# Chemodivergent dearomatization of benzene-linked O-oxime esters *via* EnT-induced radical cross-coupling†‡

**DOI:** 10.1039/d4sc07681h

**Published:** 2025-01-09

**Authors:** Guohui Zeng, Dongwen Guo, Huanfeng Jiang, Biaolin Yin

**Affiliations:** a Key Laboratory of Functional Molecular Engineering of Guangdong Province, School of Chemistry and Chemical Engineering, South China University of Technology (SCUT) Guangzhou 510640 China

## Abstract

Radical-mediated dearomatization strategies offer a blueprint for building value-added and synthetically valuable three-dimensional skeletons from readily available aromatic starting materials. Herein, we report a novel strategy by leveraging benzene-linked O-oxime esters as triply functionalized precursors to form two distinct persistent radicals under a chemodivergent pathway. These radicals then couple with a cyclohexadienyl radical for either carboamination or carbo-aminoalkylation. Remarkably, a series of 4-(2-aminoethyl)anilines derivatives featuring all-carbon quaternary centers, along with the formation of four different types of chemical bonds, are efficiently constructed through a unique rearomatization cascade in the carboamination. Importantly, employing DMPU as the hydrogen atom transfer (HAT) donor strategically diverts the reaction pathway from the C–N bond formation towards the C–C bond formation. Our mechanistic explorations support a sequential HAT/energy transfer (EnT)/HAT cascade as the key stage for carbo-aminoalkylation involving the N-center iminyl radical. Significantly, this work demonstrates the elegant expansion of divergent C–N and C–C bond formation using the imine moiety within O-oxime esters as the bifunctional reagent, and it broadens the chemical space of both benzenes and O-oxime esters in radical-mediated transformations.

## Abstract 2

Radical-mediated dearomatization strategies offer a blueprint for building value-added and synthetically valuable three-dimensional skeletons from readily available aromatic starting materials.

## Graphical abstract

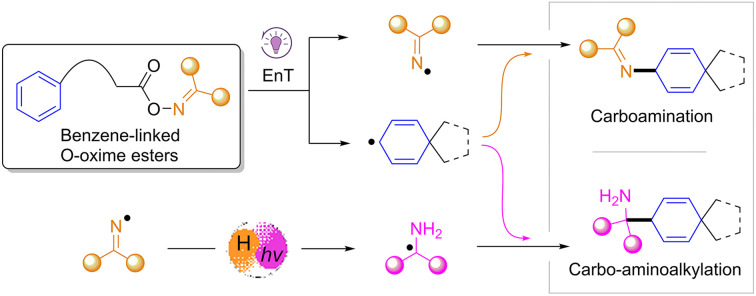

## Introduction

Phenyl rings, as readily available building blocks, undergo dearomative functionalization reactions to yield a variety of valuable skeletons that have garnered significant attention in organic synthesis.^1–3^ The chemodivergent dual-functionalization dearomatization strategy not only introduces two additional functional groups into the cyclic system but also substantially enhances the structural diversity of three-dimensional molecules.^4^ By leveraging an aromaticity disassembly-reconstruction process,^5^ peripheral editing of the benzene ring can be further achieved.^6^

**Scheme 1:**
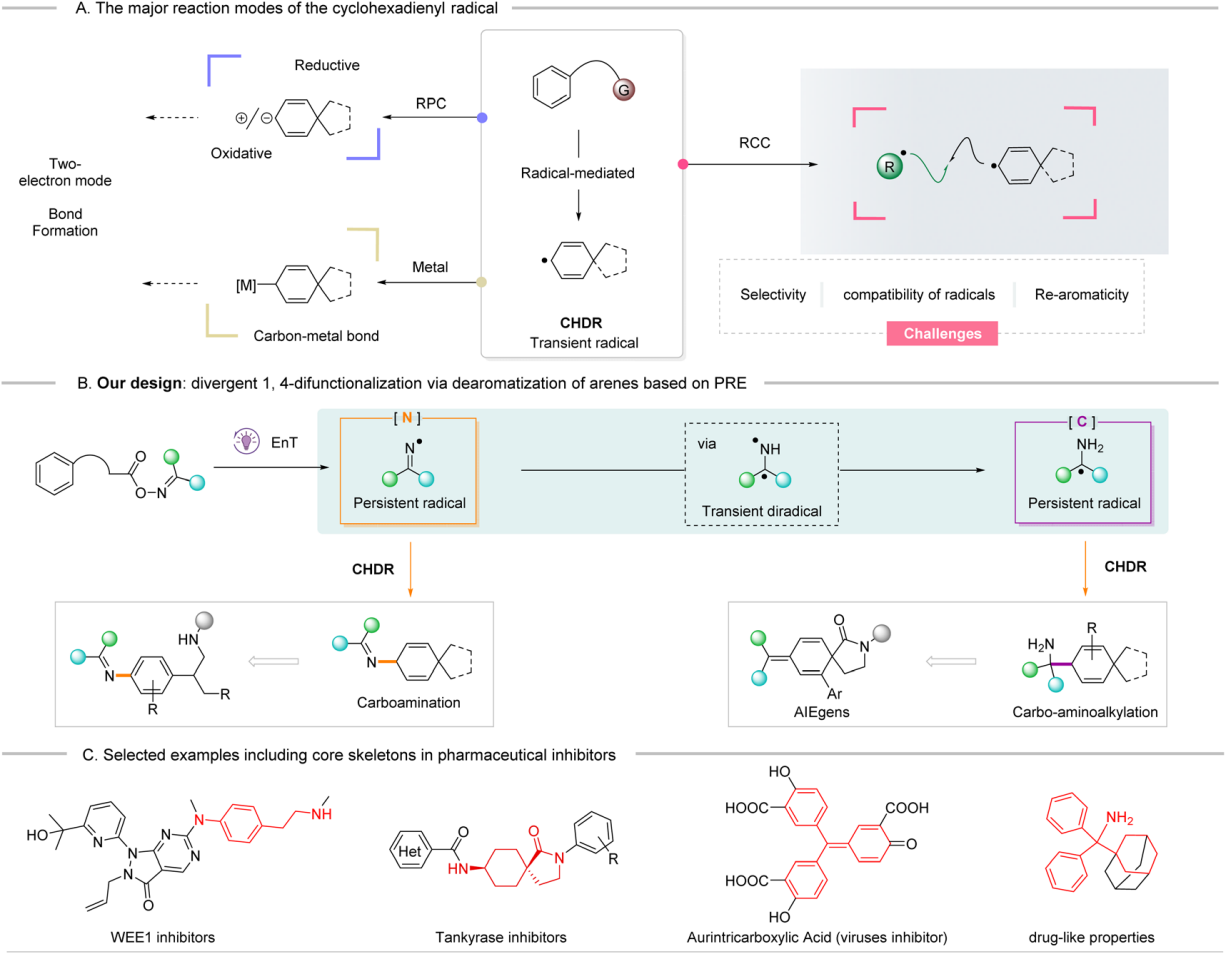
(A) Representative reaction modes of the cyclohexadienyl radical; (B) designed strategy and this work: divergent difunctionalization through dearomative radical-cross coupling of phenyl rings based on persistent radical effect; (C) selected examples including target skeletons in value-added molecules.

Compared to the traditional two-electron pathway for dearomatization of phenyl rings,^7–9^ radical-mediated dearomatizations are of interest as they tend to use mild conditions that overcome the unfavorable thermodynamics, especially for non-activated phenyl rings.^10–12^ The transience of the cyclohexadienyl radical (CHDR) intermediate during the dearomatization event, along with its high reactivity, are key factors for subsequent transformations. The radical-polar crossover (RPC)^13–15^ mechanism is well established for intercepting these radical species, leading to the irreversible generation of the corresponding ionic intermediates (Scheme 1A, upper left). Despite significant advances and their great potential, RPC strategies still have limited applications for diverse post-functionalization. For example, reductive RPC produces carbanion species, which primarily undergo protonation accompanied by few functionalizations,^16–24^ while oxidative RPC usually results in dienones *via* cation intermediates.^25–31^ Recently, a combination of CHDR and palladium catalyst to form the cyclohexadienyl Pd(ii) species^32^ may be a potential direction for expanding CHDR chemistry (Scheme 1A, lower left). Nevertheless, it is still highly desirable to continue to seek efficient transformations using CHDRs.

The radical cross-coupling (RCC) strategy has emerged as a powerful tool for difunctionalization of alkenes and other unsaturated systems.^33,34^ We envisioned that this methodology can be implemented during the dearomative process of a non-activated phenyl ring (masked cyclic triene), namely, the CHDR is terminated by another radical, to greatly expand the dearomatization chemical library (Scheme 1A, right). It is intriguing to mention that some intramolecular radical cycloaddition strategies have already been established.^35–39^ However, several challenges should be highlighted, especially in intermolecular processes, such as chemo-/regioselectivity, reactivity and compatibility of the radical species, as well as undesirable side reactions.^40–42^ Encouragingly, this pathway might be facilitated by leveraging the persistent radical effect (PRE), which relies on a kinetic mechanism to govern the RCC process. In this process two different radicals with different lifetimes are simultaneously generated.^43–45^ Therefore, potential radical partners should possess persistence, stability, and a low rate of homocoupling.

With the above concept in mind, the persistent diphenyliminyl radical was considered an excellent choice as it not only efficiently undergoes heterocoupling, but it is also easily obtained from the homolytic cleavage of oxime esters *via* visible-light mediated triplet–triplet EnT.^46–58^ On the other hand, it was hypothesized that another persistent C-centered α-amino radical could be generated *in situ* from the diphenyliminyl radical through HAT/EnT/second HAT cascade under tunable conditions within the dearomative event.^59^ Herein, as part of our ongoing research into the dearomative difunctionalization of arenes,^32,38,60^ we report a chemodivergent RCC strategy that utilizes the PRE to recombine the cyclohexadienyl radical with the diphenyliminyl *N*-radical which ultimately leads to the dearomative carboamination, or to recombine the cyclohexadienyl radical with the α-amino C-radical generated from the same substrate, which ultimately leads to carbo-aminoalkylation of non-activated phenyl rings (Scheme 1B). All these core skeletons are versatile scaffolds commonly employed in pharmaceutical molecules (Scheme 1C).

## Results and discussion

*N*-benzoyl propanoic O-oxime ester 1-1 was used as the model substrate to investigate this chemodivergent reaction. 1-1 can generate a persistent iminyl radical and a transient CHDR intermediate through an intramolecular dearomative event by the EnT catalysts cascade. The desired RCC products were successfully obtained under the screening conditions outlined in Table 1. Initially the reaction was carried out in presence of 9H-thioxanthen-9-one (TXT) with MeCN as the solvent at room temperature providing only a 34% yield of 2-1 (entry 1). Through careful analysis of the reaction products (see ESI‡ for details), we believed that additional supplementation of the iminyl radical precursor would improve the C–N bond formation process. Subsequently, dioxime oxalate A-1 was chosen as the additional iminyl radical source to further screen solvents. As expected, a significantly increased 2-1 yield was observed (60%) when MeCN was the solvent (entry 4). Additionally, the mixed solvent (MeCN/EA = 2/1, *v*/*v*) had a slightly positive effect (entry 5). THF or DMF^61^ as potential HAT precursors are commonly employed in the formation of chemical bonds.^62^ These solvents were initially evaluated in the presence of A-1 to generate the desired product 3-1. Unfortunately, these experiments failed to produce 3-1, with the reaction favoring the formation of product 2-1 in low yields (entries 7 and 8). Notably, when the reaction was conducted in the presence of γ-terpinene (A-2)^63^ in EA (entry 9), the desired product 3-1 was afforded in a 25% yield, along with approximately a 15% yield of the CHDR homodimer. Gratifyingly, DMPU^64^ exhibited a significant enhancing effect (entry 10), especially in combination with EA as the mixed solvent, affording product 3-1 in 55% yield (entry 11). Benzophenone imine (A-3), a simpler additive, increased the yield of product 3-1 to 60% (entry 12). Furthermore, increasing the loading of A-3 from 1.0 to 2.0 equiv. resulted in a significant reaction efficiency improvement (entry 13) giving the target product 3-1 in 69% yield. Control experiments confirmed that both photoirradiation and the TXT catalyst were necessary for all the desired reactions (entries 6 and 14). Nevertheless, we recognized that the low *cis*/*trans*-selectivity for both transformations might arise from lower steric control during the radical–radical recombination events. Additionally, the increased proportion of *cis*-selectivity in carbo-aminoalkylation may be ascribed to the presence of intramolecular hydrogen bonding.

**Table 1:** Optimization of reaction conditions^a^

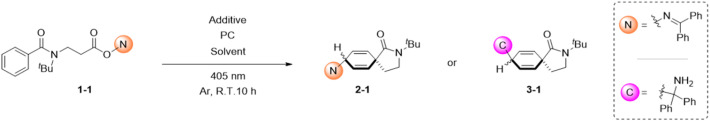						
Entry	Conditions			Yield^b^ (%)		
	Solvent	Additives	Variation	2-1	*cis*/*trans*	3-1
1	MeCN	—	PC-I	34	1 : 3.0	0
2	DCE	A-1	PC-I	35	1 : 2.6	0
3	EA	A-1	PC-I	39	1 : 3.6	0
4	MeCN	A-1	PC-I	60	1 : 2.5	0
5	MeCN/EA (2/1, *v*/*v*)	A-1	PC-I	62	1 : 2.4	0
6	MeCN/EA (2/1, *v*/*v*)	A-1	Other PC/no *hv* or PC	0		0
7	THF	A-1	PC-I	33	1 : 2.9	0
8	DMF	A-1	PC-I	24	1 : 2.7	0
9	EA	A-2	PC-I	0	2.0 : 1	25
10	DMPU	A-1	PC-I	0	1.7 : 1	37
11	DMPU/EA (1/1, *v*/*v*)	A-1	PC-I	0	1.8 : 1	55
12	DMPU/EA (1/1, *v*/*v*)	A-3	PC-I	0	1.8 : 1	60
13^c^	DMPU/EA (1/1, *v*/*v*)	A-3	PC-I	0	1.9 : 1	69
14	DMPU/EA (1/1, *v*/*v*)	A-3	Other PC/no *hv* or PC	0		0
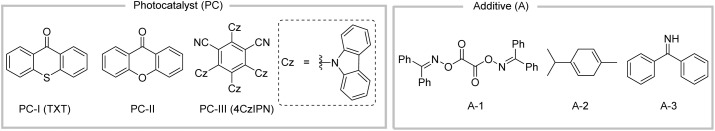						

a Standard conditions: the reactions were carried out under argon using 1-1 (0.1 mmol), Additive (1.0 equiv.), PC (5 mol%), Solvents for 2-1 (0.033 M), Solvents for 3-1 (0.05 M) at room temperature for 10 h.

b The yield of 2-1 and 3-1 were based on ^1^H NMR analysis of the crude product using CH_2_Br_2_ as the internal standard.

c Additive (2.0 equiv.). EA = ethyl acetate, DMPU = 1,3-dimethyltetrahydropyrimidin-2(1*H*)-one.

**Scheme 2:**
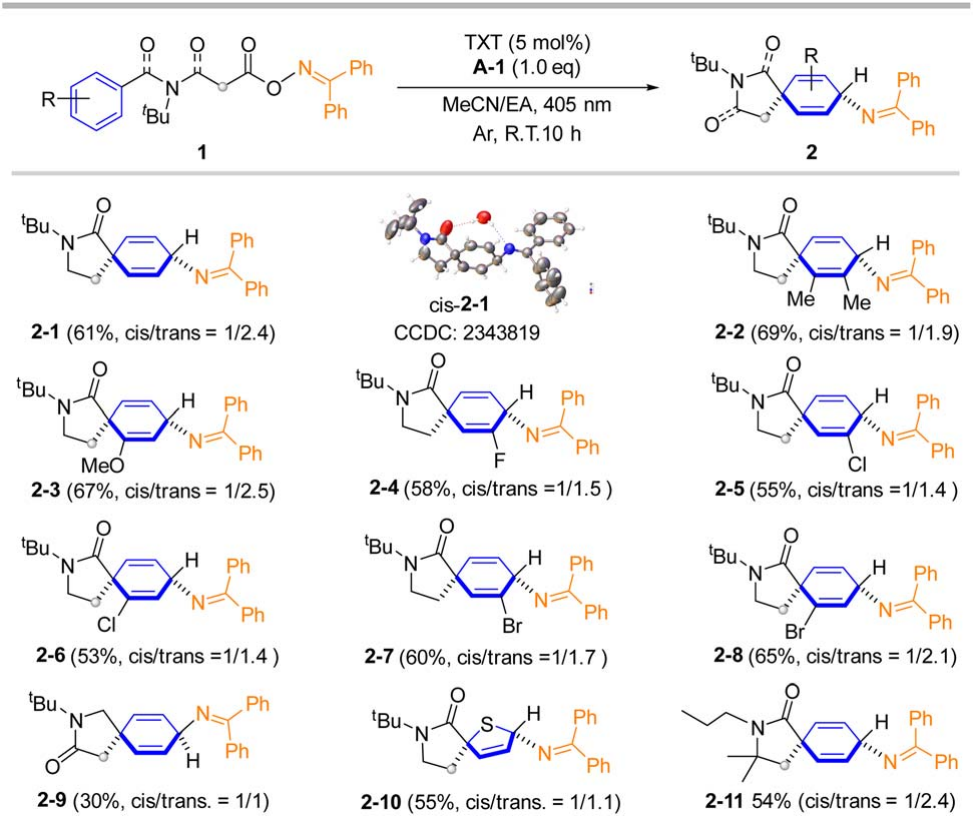
Substrate scopes for carboamination. Reaction conditions: O-oxime ester 1 (0.2 mmol), A-1 (0.2 mmol, 1.0 equiv.), TXT (5 mol%) in 6 mL of MeCN/EA (0.033 M, *v*/*v* = 2/1) under Ar atmosphere at room temperature and irradiated with visible LEDs (2 × 15 W, 405 nm) for 10 h. Isolated yields are reported as the average of four experiments.

With the optimal conditions in hand, the scope of the carboamination for non-activated phenyl substrates was investigated. As summarized in Scheme 2, various O-oxime esters derived from benzamidopropanoic acids afford the corresponding spiro-cyclohexadienyl imines in moderate to good yields. X-ray crystallographic analysis revealed the major product to be 2-1 (*trans*), with the amide carbonyl and imine moieties positioned opposite each other.^65^ The minor product, 2-1 (*cis*) was also identified. Compared to the unsubstituted phenyl product 2-1 (61% yield), the heterocoupling process showed a slight improvement in yields when substituents were present at the ortho- or meta-positions of the starting materials. Products 2-2 and 2-3 were obtained in 69% and 67% yield. Notably, it is easy to obtain cyclohexadienyl amine from 2 in the presence of weak acid conditions (see ESI‡ for detail). On the other hand, the electron-deficient groups such as F, Cl, Br on the 2- or 3-position of benzene ring were also compatible, giving corresponding products in 53% to 65% yields (2-4 to 2-8). The *N*-benzyl-derived malonate O-oxime ester also provided the dearomative product 2-9, albeit with an apparent decrease in yield, possibly due to the C–N bond is susceptible to hydrolysis, whereas steric bulk on the cyclohexadienyl amine can stabilize the C–N bond. This transformation also proceeded well when the reaction was performed on a starting material containing a thiophene ring, affording product 2-10 in a 55% yield. When *N*-propyl was used, the reaction yielded product 2-11 in 54% yield. It should be noted that the Thorpe–Ingold effect enhanced the dearomative event while there was minimal steric hindrance from the *N*-protected group (2-11). Notably, dearomative carboamination is completely inhibited by a substituent at the para-position of the phenyl ring or the 5-position of the thiophene ring (see ESI‡ for details).

**Scheme 3:**
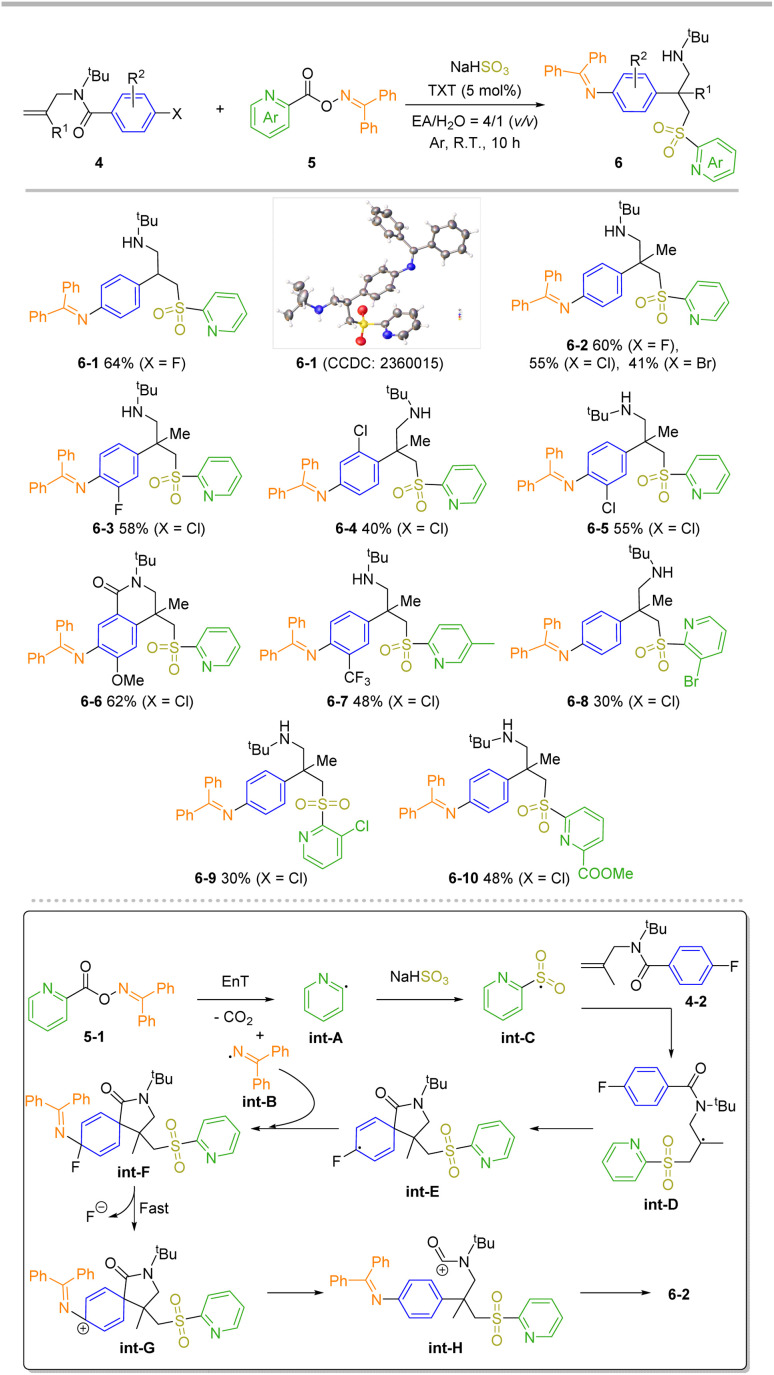
Substrate scopes for transient dearomatize-tion and plausible mechanism. Reaction conditions: *N*-allyl-*N*-(*tert*-butyl)benzamide 4 (0.2 mmol), O-oxime ester 5 (0.34 mmol, 1.7 equiv), NaHSO_3_ (0.6 mmol, 3.0 equiv.), TXT (5 mol%) in 5 mL of EA/H_2_O (*v*/*v* = 4/1) under Ar atmosphere at room temperature and irradiated with visible LEDs (2 × 15 W, 405 nm) for 10 h. Isolated yields are reported as the average of two experiments.

To make the method more useful and develop the product for new and diverse applications, we envisioned that the amine-substituted benzene ring can be achieved through a rearomative strategy,^66^ yielding 4-(2-aminoethyl)aniline derivatives. These derivatives are crucial building blocks for pharmaceuticals and liquid crystal materials. Traditionally, the pathway for introducing an amino group on the phenyl ring involves challenging nitration and reduction steps. Upon treatment of 4-bromobenzoyl-linked O-oxime ester under the standard carboamination conditions, no formation of rearomative product was observed. Interestingly, when this process was switched to an intermolecular approach, namely using 4 and 5 as starting materials, the desired product 6 was successfully obtained under modified conditions (Scheme 3). To probe this transformation's functional group tolerance, this reaction was carried with 4-1 (X = F) as the substrate. The desired product 6-1 was obtained in 64% yield, and its configuration confirmed through X-ray crystallographic analysis.^67^ It was found that the steric hindrance from the β-position of allyl (4, R^1^ = Me) is compatible, giving all-carbon quaternary center^68,69^ products in moderate to good yields (6-2, 41% to 60% yields). The effect of different halogens (X = F, Cl, Br) was also investigated. Interestingly, a decrease in the yield was observed with increasing atomic radius of the halogen. Next, several substrates 4 with different substitutions on the phenyl ring were examined. They produced the corresponding all-carbon quaternary center products in good yields (6-3 to 6-7). Significantly, rearomative cyclization product 6-6 was achieved in 62% yield, this outcome strongly indicates that a Friedel–Crafts cyclization occurred between the electron-rich phenyl ring and the amide carbocation during the cascade process. Finally, a range of O-picolinoyl oxime substrates 5 bearing different substitutions on the pyridyl ring were investigated, and the corresponding all-carbon quaternary products (6-7 to 6-10) were obtained. Note that the yields of products decreased when there was steric hindrance at the C3 position of the pyridyl ring (30% yields for both 6-8 and 6-9).

Based on the experiment outcomes, and our recently reported results,^66^ a plausible reaction mechanism for this transient dearomatization process is proposed in Scheme 3 (bottom). N–O bond cleavage occurs in the triplet state of 5-1 (generated by EnT process with excited TXT), followed by the release of CO_2_ to yield int-A and int-B. int-A quickly reacts with SO_2_ to form a sulfonyl radical int-C, which then affords the int-D through radical addition with alkene 4-2.^70,71^ The key intermediate, int-F forms *via* a radical-induced dearomatization/RCC process cascade from int-D. Then, a rapid dehalogenation process takes place *in situ* to yield int-G, which provides the 4-(2-aminoethyl)-aniline derivatives 6-2 through the rearomative cation int-H.^72^ When a more electron-rich phenyl ring is involved, a Friedel–Crafts cyclization event will take place.

**Scheme 4:**
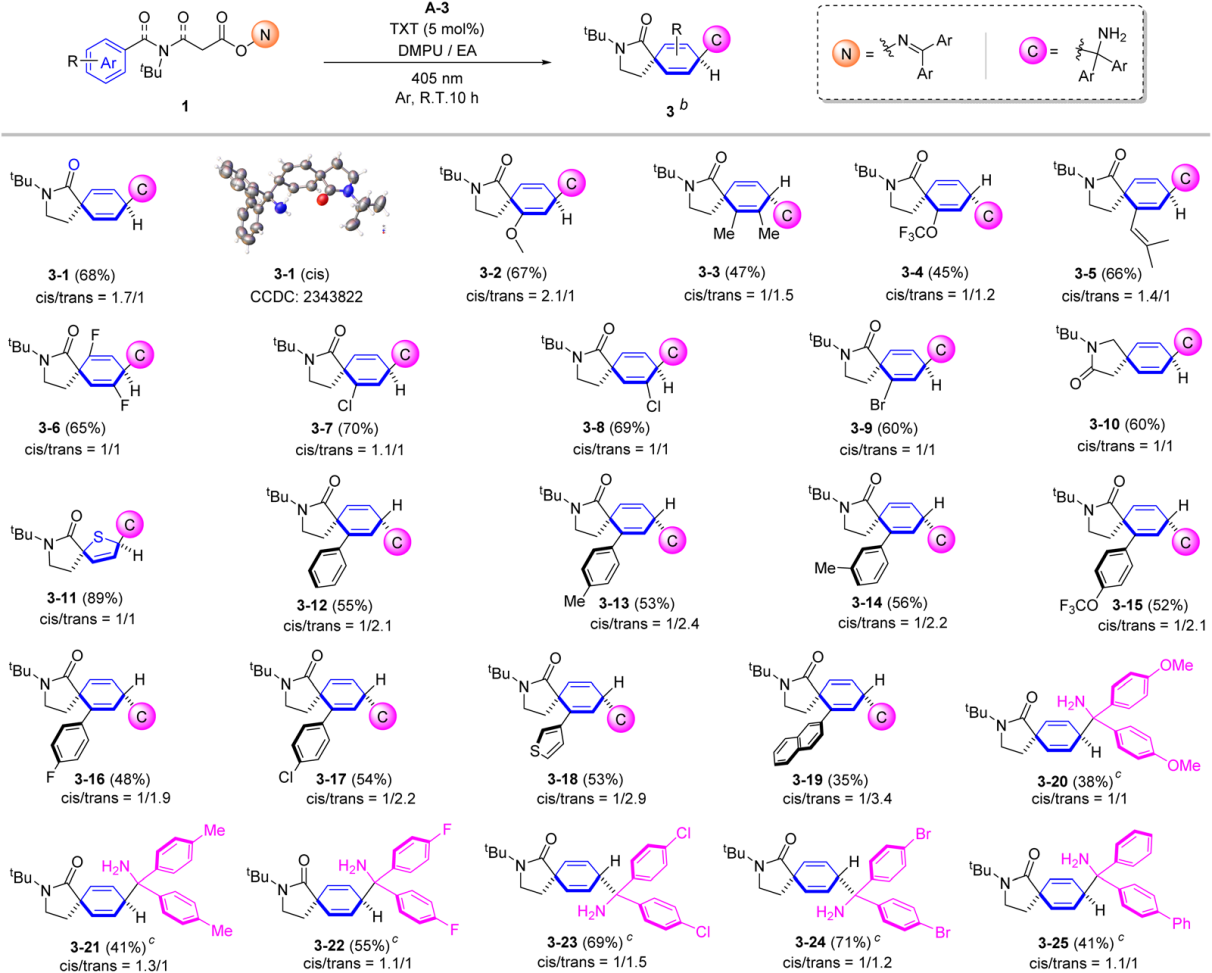
Substrate scope for carbo-aminoalkylation. Reaction conditions: ^*a*^ O-oxime ester 1 (0.2 mmol), A-3 (0.4 mmol, 2.0 equiv.), TXT (5 mol%) in 4 mL of DMPU/EA (0.05 M, *v*/*v* = 1/1) under Ar at room temperature and irradiated with visible LEDs (2 × 15 W, 405 nm) for 10 h. Isolated yields are reported as the average of four experiments. ^*b*^Major products were visualized. ^*c*^The corresponding imine additive was used instead of A-3.

Next, an investigation into the substrate's compatibility for the carbo-aminoalkylation reaction was conducted and the results summarized in Scheme 4. The standard product 3-1 was obtained in 68% yield (*cis*/*trans* = 1.7/1). X-ray crystallographic analysis confirmed a *cis* configuration for the major diastereomer which exhibits low polarity.^73^ A large variety of substituents on the phenyl ring gave the corresponding spiro carbo-aminoalkylation products (3-2 to 3-9) in moderate to good yields. Notably, different electronic properties such as methoxy (3-2), 2,3-dimethyl (3-3), trifluoromethoxy (3-4), isobutenyl (3-5) and halides (3-6 to 3-9) were well tolerated. In addition, a malonic acid derived O-oxime reagent afforded product 3-10 in 60% yield, showing the formal transposition of the carbonyl group relative to the standard product. Gratifyingly, as mentioned earlier, when employing a thiophene ring substrate, product 3-11 was obtained in excellent yield (up to 89%). *ortho*-Aryl biphenyl O-oxime ester substrates bearing a broad range of substituents on the aryl position, including methyl (3-13 and 3-14), trifluoromethoxy (3-15), and halides (3-16 and 3-17), underwent the reaction to provide the desired products in moderate yields (48% to 56%). Additionally, this reaction tolerated other aryl substituents at the *ortho*-position, such as 3-thiophenyl (3-18) and 2-naphthalenyl (3-19). However, the yield was significantly affected by the steric hindrance of the aryl moiety, with bulkier substituents leading to lower yields. Notably, the reaction exhibited a remarkable preference for the trans configuration due to the steric hindrance imposed by the *ortho*-aryl group. Furthermore, the electronic effect of the symmetrical benzene rings on the diphenyl imine radical was studied. Increasing product yield was observed as the substituents on the diphenyl ring evolved from electron-donating methoxy (3-20, 38% yield) and methyl (3-21, 41% yield) groups to electron-withdrawing fluoro (3-22, 55% yield), chloro (3-23, 69% yield), and bromo (3-24, 71% yield) groups. This observation underscores the profound impact of electronic effects on the outcomes of the reaction. Finally, the reaction successfully incorporated a non-symmetric diphenyl imine radical, affording product 3-25 in a moderate 41% yield.

**Scheme 5:**
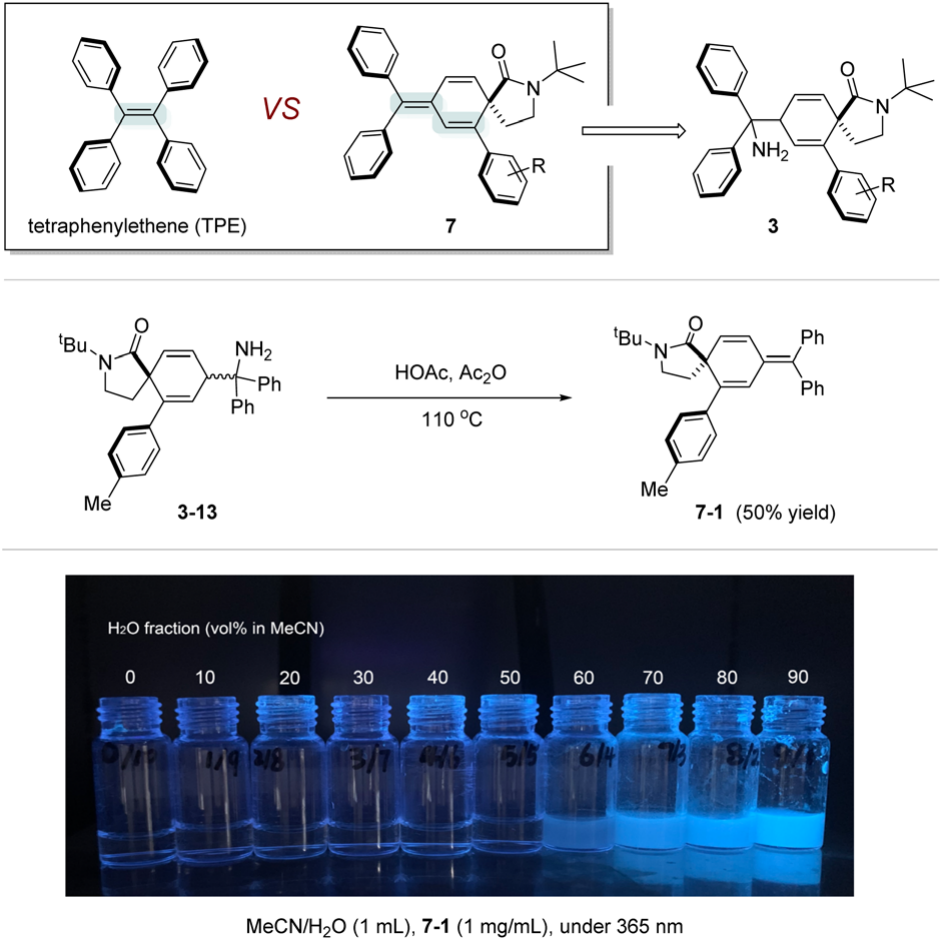
The synthesis of a novel AIEgens structure and fluorescence experiment. Reaction conditions: 3-13 (0.3 mmol), in 3 mL of HOAc/Ac_2_O (*v*/*v* = 1/2) under Ar atmosphere at 110 °C for 20 h. Isolated yield is reported.

Inspired by the well-studied tetraphenylethylene (TPE) AIE materials, we realized that the conjugated triphenylcyclo-hexadiene product 7 (Scheme 5), likely formed *via* NH_3_ elimination from 3, is expected to exhibit a similar AIE effect. To investigate this hypothesis further, a new molecule, 7-1, was synthesized. It's fluorescence image (*λ*_irr_ = 365 nm) at different compositions is shown in Scheme 5 (bottom) and supports the AIE property.

**Scheme 6:**
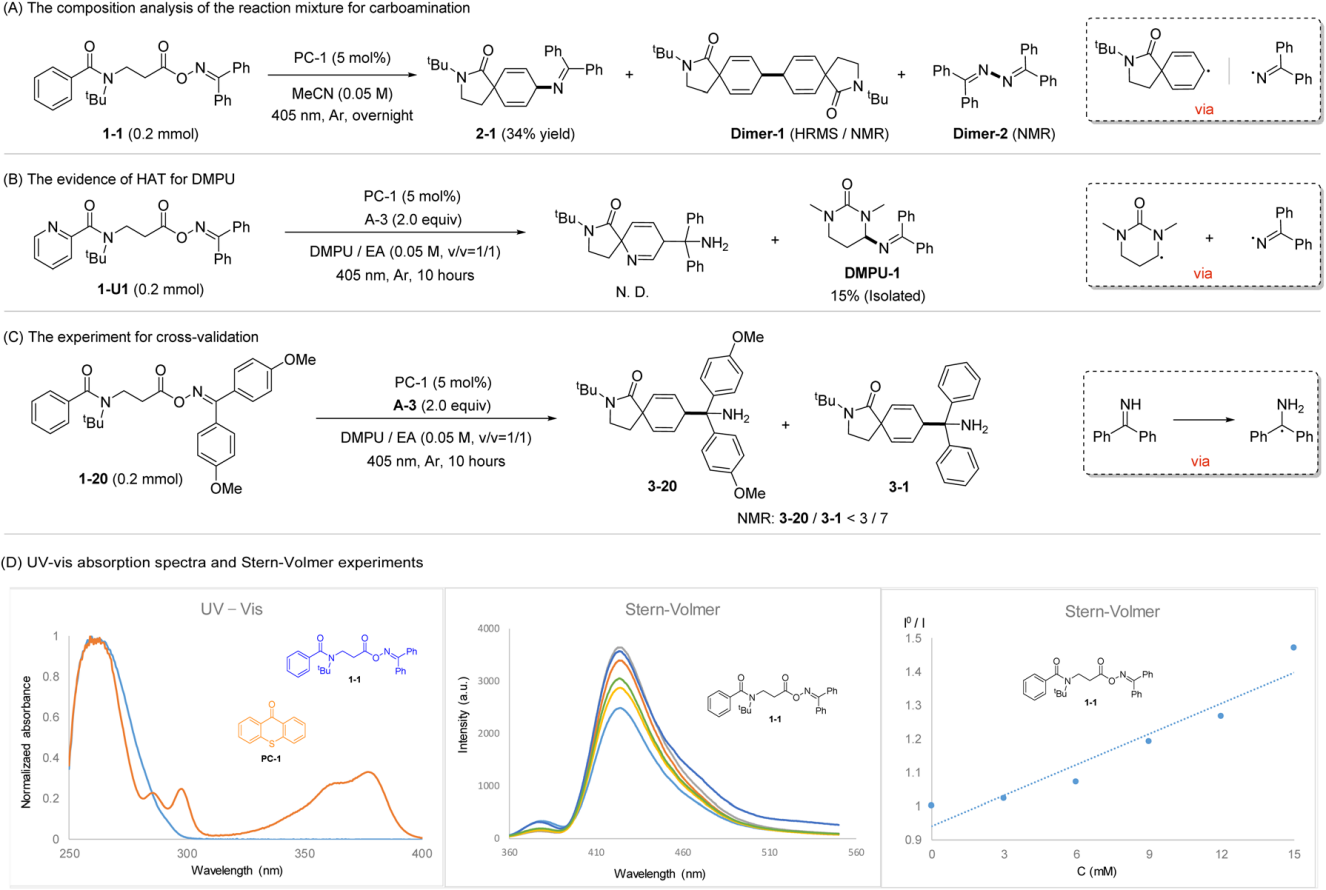
Control experiments.

To gain insights into the reaction mechanism, a series of control experiments were conducted under specific conditions, and the corresponding results are provided in Scheme 6. Careful analysis of the initial reaction mixture by HRMS and NMR shows the desired product 2-1 was present along with dimer byproducts Dimer-1 and Dimer-2 (Scheme 6A). This observation suggests the involvement of the cyclohexadienyl and the iminyl radical as reaction intermediates. Switching from benzamide O-oxime ester to picolinamide O-oxime ester did not yield the desired product, however, the reaction surprisingly afforded a key intermediate DMPU-1 in 15% yield (Scheme 6B). This result strongly implies a HAT process under the standard conditions, potentially with DMPU acting as the hydrogen atom donor or a mediator in the process. To validate our hypothesis, a cross-validation experiment, reacting 1-20 with A-3 under standard conditions, was performed. NMR analysis of the inseparable reaction products revealed 3-1 as the major component and 3-20 as a minor component (Scheme 6C). This finding illustrates that the α-amino carbon-centered radical arises from the transformation of A-3*via* a triplet EnT/HAT cascade process. UV-vis characterization of 1-1 and PC-1 revealed that 1-1 cannot be directly excited by visible-light. Furthermore, the Stern–Volmer experiments elucidated that an EnT process between 1-1 and excited-state PC-1 (Scheme 6D).

**Scheme 7:**
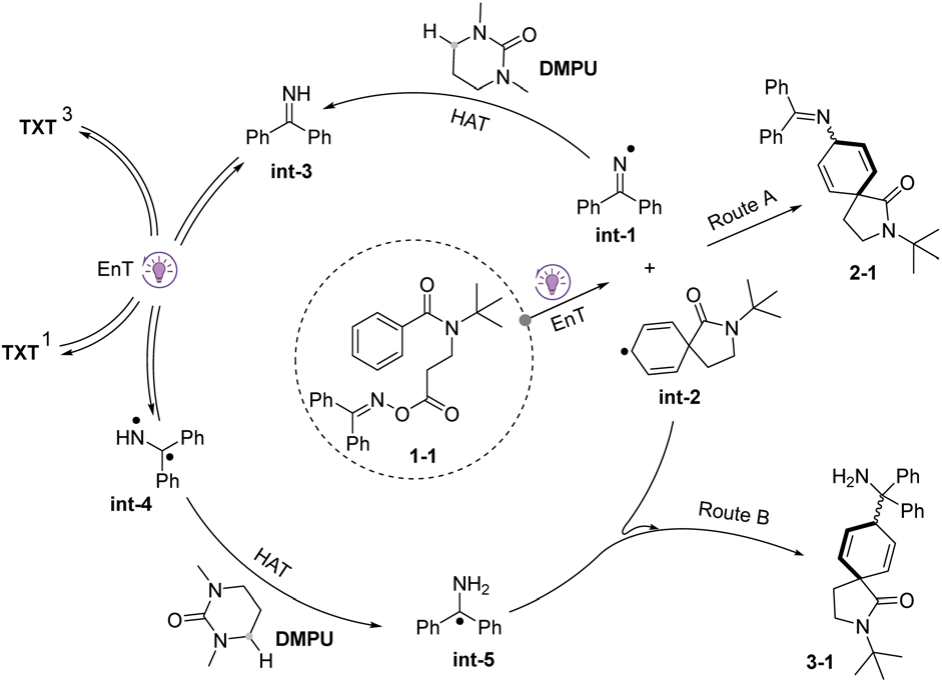
Plausible mechanism.

Based on the above experimental results, a proposed photocatalytic pathway for the chemodivergent radical cross-coupling is outlined in Scheme 7. Initially, O-oxime ester 1-1 undergoes excitation *via* an EnT process, followed by N–O bond homolysis of triplet state 1-1*. This event, coupled with releasing CO_2_ and an intramolecular dearomatization process, leads to the formation of the *N*-centered radical int-1 and the CHDR int-2. These two radicals readily couple together, leading to the formation of product 2-1 (route A). However, the presence of a suitable HAT donor, such as DMPU, disrupts this established coupling process, leading to the formation of imine int-3*via* an alternative pathway. Subsequently, imine int-3 can be directly excited by excited TXT to generate its triplet state (int-4),^74^ followed by a second HAT process with DMPU, yielding int-5. Ultimately, the radical–radical cross-coupling between int-2 and int-5 affords product 3-1 (route B), forming a new C(sp^3^)–C(sp^3^) bond instead of the above C(sp^3^)–N(sp^2^) bond.

## Conclusions

In summary, an energy transfer catalysis strategy was developed to enable chemodivergent dearomatization of benzene rings. This strategy harnesses the PRE to accomplish both carboamination and carbo-aminoalkylation through RCC. Employing this protocol, four structurally distinct molecular frameworks were synthesized *via* divergent pathways. We believe this discovery represents a groundbreaking advancement in C–C bond formation by utilizing O-oxime esters. It marks a significant departure from the traditional paradigm of C–N bond formation and holds immense potential for expanding the chemical library.

## Data availability

Supporting data or code have been included in the article's ESI‡.

## Author contributions

This work was conceptualized by G. Z. and prof. B. Y. experimentation was performed by G. Z. and D. G. The first draft of the manuscript was prepared by G. Z. and the final version was edited and revised by prof. B. Y. and prof. H. J.

## Conflicts of interest

There are no conflicts to declare.

## Supplementary Material

SC-OLF-D4SC07681H-s001
