# Supplementary material for: Chemodivergent dearomatization of benzene-linked O-oxime esters via EnT-induced radical cross-coupling
Source: Chem Sci. 2025 Jan 9;16(6):2690–9. doi: 10.1039/d4sc07681h (PMC11717118; doi:10.1039/d4sc07681h)
Supplement: SC-016-D4SC07681H-s001 [file SC-016-D4SC07681H-s001.pdf]

Supplementary Information (SI) for Chemical Science.
This journal is © The Royal Society of Chemistry 2024

Supporting Information

Chemodivergent Dearomatization of Benzene-linked O-oxime Esters via EnT-Induced Radical Cross-coupling

Guohui Zeng,[a] Dongwen Guo,[a] Huanfeng Jiang,[a] Biaolin Yin[a*]

[a]Key Laboratory of Functional Molecular Engineering of Guangdong Province, School of Chemistry and Chemical Engineering, South China University of Technology (SCUT), Guangzhou 510640, China.

[*]Corresponding Author: Biaolin Yin: *blyin@scut.edu.cn*

Table of contents

I. General information....2

II. General procedure for preparing additive and 4-halogenated materials....2

II.1 The synthesis of **A-1**....2

II.2 The synthesis of **4**....3

III. General procedure for preparing O-oxime ester materials....4

III.1 Method I....4

III.2 Method II....13

III.3 Method III....14

IV. Investigations of reaction conditions....15

V. General procedure for the chemodivergent reactions....16

V.1 Carboamination....16

V.2 Rearomatization....22

V.3 Carbo-aminoalkylation....25

V.4 The synthesis of **7-1**....38

V.5 The synthesis of cyclohexadienyl amine....39

VI. Unsuccessful substrates for chemodivergent reactions....39

VII. Control experiments....40

VIII. Crystallographic data....43

IX. References....46

X. NMR Spectra....47

I. General information

Unless specified otherwise, all experiments were performed under a N_2 atmosphere. All reagents and starting materials were purchased from commercial suppliers and used as received. FT-IR spectra were obtained with thin film samples or KBr pellets on a Bruker Vector 22 spectrometer, and data are expressed in cm^{-1}. ^{1}H, ^{13}C, ^{19}F NMR spectra were recorded on Bruker AVANCE 400 (400 MHz for ^{1}H; 100 MHz for ^{13}C; 376 MHz for ^{19}F) or a Bruker AVANCE 500 (500 MHz for ^{1}H; 126 MHz for ^{13}C; 471 MHz for ^{19}F), ^{1}H NMR and ^{13}C NMR chemical shifts were determined relative to internal standard $CDCl_3$ at 7.26 and 77.0. Chemical shifts (δ) are reported in ppm and coupling constants (*J*) are in Hertz (Hz). The following abbreviations were used to explain the multiplicities: s = singlet, d = doublet, t = triplet, q = quartet, m = multiplet, dd = doublet of doublet. The HRMS measurements were recorded on a TOF analyzer using an ESI source in the positive mode. The crystal data was collected on an Agilent Gemini E diffractometer (Mo, 50kV 40mA)and reduced by CrysAlisPro (Rigaku). Melting points were determined using a hot stage apparatus. Column chromatography was performed on silica gel (200–300 mesh) with mixtures of petroleum ether and ethyl acetate as the eluent or other solvents.

II. General procedure for preparing additive and 4-halogenated materials.

II.1 The synthesis of A-1

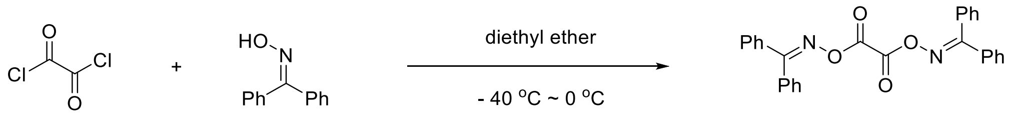

A-1

To a solution of oxalyl dichloride (10 mmol, 1.0 eq) in dry diethyl ether (20 mL), a solution of diphenylmethanone oxime (20 mmol, 2.0 eq) in dry diethyl ether (40 mL) was slowly added at -40 °C for over 10 minutes. After addition, the mixture was carefully warmed to 0 °C and stirred for another 2 hours. The reaction mixture was filtered, and the filter cake washed with diethyl ether to give the product **A-1** which was used directly in the next step without purification.

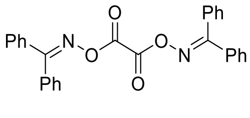

O,O'-oxalylbis(1,1-diphenylbenzophenone oxime) (**A-1**)

The title compound was obtained as a white solid (4.0 g, 89% yield).

NMR: ^{1}H NMR (400 MHz, Chloroform-*d*) δ 7.52 (d, *J* = 8.1 Hz, 1H), 7.52 – 7.44 (m, 2H), 7.43 (t, *J* = 7.3 Hz, 1H), 7.37 (t, *J* = 7.7 Hz, 1H), 7.29 (d, *J* = 7.1 Hz, 1H). ^{13}C NMR (101 MHz, Chloroform-*d*) δ 133.8, 131.4, 131.1, 130.2, 129.2, 129.1, 128.5, 128.3.

This compound is known.[1,2]

II.2 The synthesis of 4

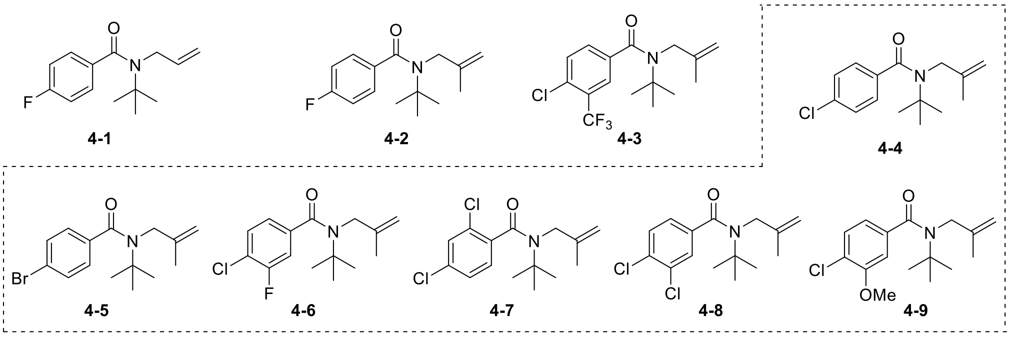

The 4-halogenated N-allyl benzamide starting materials (**4-1** to **4-9**) are prepared using our previous method.[3] **4-1**, **4-2** and **4-3** are new compounds, **4-4** to **4-9** are known compounds.

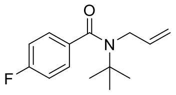

N-allyl-*N*-(tert-butyl)-4-fluorobenzamide (**4-1**)
The title compound was obtained as a colorless oil (500 mg, 86% yield).
NMR: ^{1}H NMR (400 MHz, Chloroform-*d*) δ 7.41 – 7.33 (m, 2H), 7.06 – 6.99 (m, 2H), 5.81 – 5.70 (m, 1H), 5.17 – 5.05 (m, 2H), 3.94 – 3.88 (m, 2H), 1.54 (s, 9H). ^{13}C NMR (101 MHz, Chloroform-*d*) δ 164.1, 161.6, 137.0, 135.5 (d, *J* = 3.6 Hz), 128.2 (d, *J* = 8.3 Hz), 116.2, 115.1 (d, *J* = 21.7 Hz), 57.6, 49.9, 28.6. ^{19}F NMR (376 MHz, Chloroform-*d*) δ -111.83.
HRMS (APCI): calcd for $C_{14}H_{19}FNO^+$ $[M + H]^+$: 236.1445; found: 236.1441.

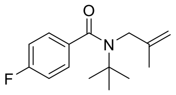

N-(tert-butyl)-4-fluoro-*N*-(2-methylallyl)benzamide (**4-2**)
The title compound was obtained as a white solid (486 mg, 85% yield).
NMR: ^{1}H NMR (400 MHz, Chloroform-*d*) δ 7.43 – 7.35 (m, 2H), 7.03 – 6.94 (m, 2H), 5.04 (s, 1H), 4.99 (m, 1H), 3.74 (s, 2H), 1.53 (s, 9H), 1.52 (s, 3H). ^{13}C NMR (101 MHz, Chloroform-*d*) δ 172.7, 162.8 (d, *J* = 248.3 Hz), 144.0, 135.4 (d, *J* = 3.6 Hz), 128.0 (d, *J* = 8.4 Hz), 115.0 (d, *J* = 21.6 Hz), 111.9, 57.9, 53.2, 28.2, 20.0. ^{19}F NMR (376 MHz, Chloroform-*d*) δ -111.9.
HRMS (APCI): calcd for $C_{15}H_{21}FNO^+$ $[M + H]^+$: 250.1602; found: 250.1659.

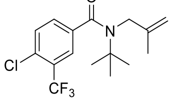

N-(tert-butyl)-4-chloro-*N*-(2-methylallyl)-3-(trifluoromethyl)benzamide (**4-3**)
The title compound was obtained as a colorless oil (510 mg, 87% yield).
NMR: ^{1}H NMR (400 MHz, Chloroform-*d*) δ 7.74 (d, *J* = 2.0 Hz, 1H), 7.52 (dd, *J* = 8.3, 2.1 Hz, 1H), 7.44 (d, *J* = 8.2 Hz, 1H), 5.06 (s, 1H), 5.05 (q, *J* = 1.5 Hz, 1H), 3.68 (s, 2H), 1.53 (s, 9H), 1.52 (s, 3H). ^{13}C NMR (101 MHz, Chloroform-*d*) δ 170.9, 143.8, 138.0, 132.7 (d, *J* = 2.0 Hz), 131.2, 130.2, 128.2 (q, *J* = 31.8 Hz), 125.3 (q, *J* = 5.3 Hz), 122.5 (d, *J* = 273.4 Hz), 112.1, 58.2, 53.0, 28.1, 19.9. ^{19}F NMR (376 MHz, Chloroform-*d*) δ -62.8.

HRMS (APCI): calcd for $C_{16}H_{20}ClF_3NO^+$ $[M + H]^+$: 334.1180; found: 334.1177.

III. General procedure for preparing O-oxime ester materials.

III.1 Method I

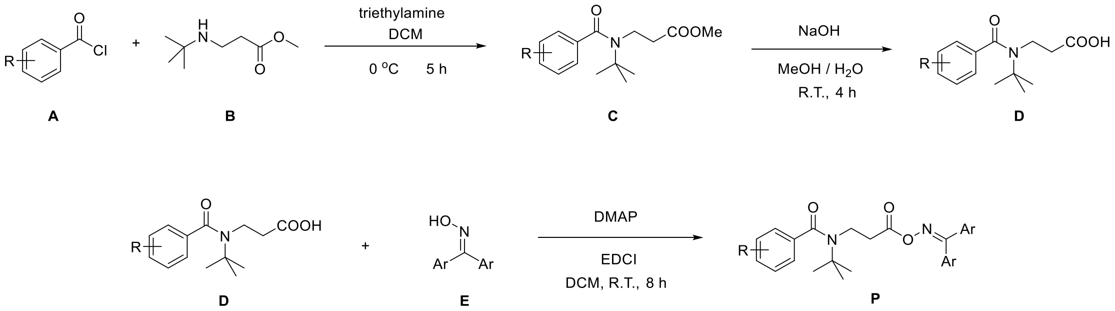

Step 1:

To a solution of methyl 3-(tert-butylamino)propanoate **B** (5 mmol, 1.0 eq) and triethylamine (7.5 mmol, 1.5 eq) in DCM (30 mL) was slowly added the corresponding benzoyl chloride **A** (5 mmol, 1.0 eq) at 0°C, then stirred at 0°C for 5 hours. Then the reaction was quenched with water and extracted with DCM for three times. The combined organic layer was dried over anhydrous Na_2SO_4 and filtered. The solvent was removed under reduced pressure to give crude product **C** which was used directly in the next step without purification.

Step 2:

To a solution of crude product **C** in MeOH and H_2O (30 mL, v/v = 4/1) was added NaOH (7.5 mmol, 1.5 eq). The solution was stirred at room temperature for 4 hours, then the reaction solution was carefully adjusted pH to 3~5 by 1M HCl(aq). After removing MeOH under reduced pressure the reaction mixture was extracted with DCM for three times. The combined organic layers were washed with brine, dried over sodium sulfate, filtered, concentrated, and purified by recrystallization from petroleum ether and EtOAc to give the corresponding acid **D**.

Step 3:

To a solution of acid **D**, oxime derivatives **E** (4.5 mmol, 0.9 eq) and DMAP (0.25 mmol, 5 mol%) in dry DCM (50 mL), was added EDCI (12.5 mmol, 2.5 eq) in portions at room temperature and stirred for 8 hours.[4] The reaction mixture was quenched with water and extracted with DCM three times. The combined organic layers were washed with brine, dried over sodium sulfate, filtered, concentrated, and purified by column chromatography on silica gel (10 : 1 petroleum ether : EtOAc) to give the oximes **P**.

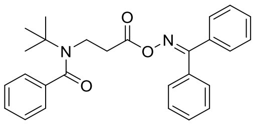

N-(tert-butyl)-N-(3-(((diphenylmethylene)amino)oxy)-3-oxopropyl)benzamide (**1-1**)

The title compound was obtained as a white solid (1.5 g, 71% yield).

NMR: ^{1}H NMR (400 MHz, Chloroform-*d*) δ 7.52 (d, *J* = 7.8 Hz, 2H), 7.49 – 7.40 (m, 4H), 7.37 – 7.31 (m, 5H), 7.30 – 7.25 (m, 2H), 7.24 – 7.17 (m, 2H), 3.60 – 3.51 (m, 2H), 2.54 – 2.45 (m, 2H), 1.47 (s,

9H). ^{13}C NMR (101 MHz, Chloroform-*d*) δ 173.3, 168.1, 165.2, 138.9, 134.2, 132.2, 130.9, 129.6, 128.9, 128.8, 128.4, 128.4, 128.3, 128.1, 125.8, 57.1, 42.4, 34.9, 28.7.

HRMS (APCI): calcd for $C_{27}H_{29}N_2O_3^+$ $[M + H]^+$: 429.2173; found: 429.2175.

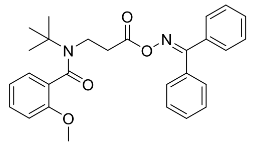

N-(tert-butyl)-N-(3-(((diphenylmethylene)amino)oxy)-3-oxopropyl)-2-methoxybenzamide (**1-2**)

The title compound was obtained as a colorless oil (1.4 g, 60% yield).

NMR: ^{1}H NMR (400 MHz, Chloroform-*d*) δ 7.57 – 7.52 (m, 2H), 7.53 – 7.40 (m, 4H), 7.39 – 7.33 (m, 2H), 7.32 – 7.21 (m, 3H), 7.09 (dd, *J* = 7.4, 1.7 Hz, 1H), 6.96 – 6.90 (m, 1H), 6.86 (dd, *J* = 8.4, 0.9 Hz, 1H), 3.75 (s, 3H), 3.60 – 3.38 (m, 2H), 2.63 – 2.36 (m, 2H), 1.51 (s, 9H). ^{13}C NMR (101 MHz, Chloroform-*d*) δ 170.4, 168.4, 165.1, 154.4, 134.3, 132.3, 130.9, 129.6, 129.5, 128.8, 128.5, 128.3, 128.1, 126.7, 120.7, 111.0, 57.2, 55.3, 42.0, 34.7, 28.8.

HRMS (APCI): calcd for $C_{28}H_{31}N_2O_4^+$ $[M + H]^+$: 459.2278; found: 459.2277.

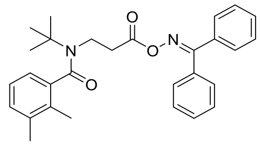

N-(tert-butyl)-N-(3-(((diphenylmethylene)amino)oxy)-3-oxopropyl)-2,3-dimethylbenzamide (**1-3**)

The title compound was obtained as colorless oil (1.3 g, 58% yield).

NMR: ^{1}H NMR (400 MHz, Chloroform-*d*) δ 7.54 – 7.51 (m, 2H), 7.50 – 7.41 (m, 4H), 7.37 – 7.32 (m, 2H), 7.25 – 7.18 (m, 2H), 7.09 (d, *J* = 6.2 Hz, 1H), 7.04 (t, *J* = 7.5 Hz, 1H), 6.91 (d, *J* = 7.5 Hz, 1H), 3.59 – 3.48 (m, 1H), 3.36 – 3.24 (m, 1H), 2.46 (t, *J* = 8.1 Hz, 2H), 2.25 (s, 3H), 2.14 (s, 3H), 1.51 (s, 9H). ^{13}C NMR (101 MHz, Chloroform-*d*) δ 172.9, 165.3, 137.5, 134.3, 131.3, 131.0, 129.8, 129.6, 128.9, 128.5, 128.3, 128.2, 125.7, 122.7, 57.3, 41.8, 34.7, 28.8, 20.0, 15.8.

HRMS (APCI): calcd for $C_{29}H_{33}N_2O_3^+$ $[M + H]^+$: 457.2486; found: 457.2488.

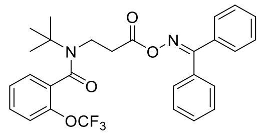

N-(tert-butyl)-N-(3-(((diphenylmethylene)amino)oxy)-3-oxopropyl)-2-(trifluoromethoxy)benzamide (**1-4**)

The title compound was obtained as a colorless oil (1.3 g, 58% yield).

NMR: ^{1}H NMR (400 MHz, Chloroform-*d*) δ 7.57 – 7.50 (m, 2H), 7.51 – 7.40 (m, 4H), 7.40 – 7.30 (m, 3H), 7.30 – 7.26 (m, 2H), 7.25 – 7.19 (m, 3H), 3.59 – 3.47 (m, 1H), 3.43 – 3.31 (m, 1H), 2.61 – 2.49 (m, 1H), 2.48 – 2.36 (m, 1H), 1.48 (s, 9H). ^{13}C NMR (101 MHz, Chloroform-*d*) δ 167.9, 167.7, 165.4, 144.2, 134.3, 132.3, 132.1, 131.0, 130.0, 129.6, 128.9, 128.4, 128.3, 128.2, 127.8, 127.0, 120.1, 57.7, 42.0, 34.8, 28.6. ^{19}F NMR (376 MHz, Chloroform-*d*) δ -56.9.

HRMS (APCI): calcd for $C_{28}H_{28}F_3N_2O_4^+$ $[M + H]^+$: 513.1996; found: 513.1998.

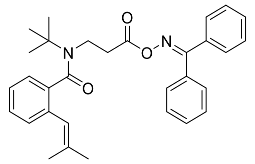

N-(tert-butyl)-N-(3-(((diphenylmethylene)amino)oxy)-3-oxopropyl)-2-(2-methylprop-1-en-1-yl)benzamide (**1-5**)

The title compound was obtained as a brown oil (1.1 g, 48% yield).

NMR: ^{1}H NMR (400 MHz, Chloroform-*d*) δ 7.54 (d, *J* = 7.7 Hz, 2H), 7.52 – 7.40 (m, 4H), 7.34 (t, *J* = 7.5 Hz, 2H), 7.28 (d, *J* = 5.3 Hz, 1H), 7.25 – 7.12 (m, 5H), 6.23 (s, 1H), 3.53 – 3.40 (m, 1H), 3.39 – 3.26 (m, 1H), 2.52 – 2.33 (m, 2H), 1.84 (s, 3H), 1.73 (s, 3H), 1.50 (s, 9H). ^{13}C NMR (101 MHz, Chloroform-*d*) δ 172.1, 168.0, 138.2, 136.9, 134.2, 134.0, 132.2, 130.9, 129.5, 129.5, 128.7, 128.3, 128.2, 128.1, 127.8, 126.2, 125.1, 122.0, 57.1, 41.6, 34.3, 28.6, 26.0, 19.1.

HRMS (APCI): calcd for $C_{31}H_{35}N_2O_3^+$ $[M + H]^+$: 483.2642; found: 483.2637.

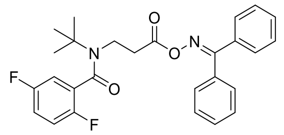

N-(tert-butyl)-N-(3-(((diphenylmethylene)amino)oxy)-3-oxopropyl)-2,5-difluorobenzamide (**1-6**)

The title compound was obtained as a white solid (1.0 g, 43% yield).

NMR: ^{1}H NMR (400 MHz, Chloroform-*d*) δ 7.53 (d, *J* = 7.8 Hz, 2H), 7.50 – 7.39 (m, 4H), 7.33 (t, *J* = 7.6 Hz, 2H), 7.22 (d, *J* = 7.3 Hz, 2H), 6.99 (t, *J* = 5.6 Hz, 2H), 6.93 (t, *J* = 6.4 Hz, 1H), 3.53 (t, *J* = 7.9 Hz, 2H), 2.51 (d, *J* = 8.0 Hz, 2H), 1.47 (s, 9H). ^{13}C NMR (101 MHz, Chloroform-*d*) δ 167.8, 166.0 (d, *J* = 1.5 Hz), 165.3, 158.4 (dd, *J* = 244.7, 2.3 Hz), 153.3 (dd, *J* = 242.3, 2.5 Hz), 134.1, 132.1, 131.0, 129.6, 128.8, 128.3, 128.3, 128.1, 117.1 (dd, *J* = 19.2, 8.4 Hz), 116.9 (dd, *J* = 18.9, 8.4 Hz), 114.5 (dd, *J* = 25.2, 4.5 Hz), 57.8, 42.1, 34.6, 28.5. ^{19}F NMR (376 MHz, Chloroform-*d*) δ -117.3 (d, *J* = 17.7 Hz), -122.6 (d, *J* = 17.6 Hz).

HRMS (APCI): calcd for $C_{27}H_{27}F_2N_2O_3^+$ $[M + H]^+$: 465.1984; found: 465.1988.

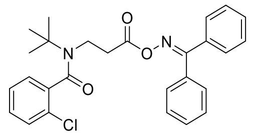

N-(tert-butyl)-2-chloro-N-(3-(((diphenylmethylene)amino)oxy)-3-oxopropyl)benzamide (**1-7**)

The title compound was obtained as a white solid (1.2 g, 51% yield).

NMR: ^{1}H NMR (400 MHz, Chloroform-*d*) δ 7.55 – 7.51 (m, 2H), 7.50 – 7.41 (m, 4H), 7.34 (t, *J* = 7.9 Hz, 3H), 7.28 – 7.17 (m, 5H), 3.61 – 3.50 (m, 1H), 3.43 – 3.28 (m, 1H), 2.64 – 2.52 (m, 1H), 2.49 – 2.37 (m, 1H), 1.50 (s, 9H). ^{13}C NMR (101 MHz, Chloroform-*d*) δ 168.9, 167.9, 165.2, 137.8, 134.2, 132.1, 130.9, 129.6, 129.6, 129.4, 128.8, 128.3, 128.2, 128.1, 127.0, 126.9, 57.6, 41.8, 34.7, 28.6.

HRMS (APCI): calcd for $C_{27}H_{28}ClN_2O_3^+$ $[M + H]^+$: 463.1783; found: 463.1781.

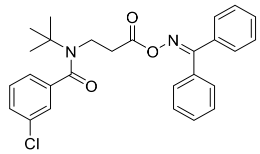

N-(tert-butyl)-3-chloro-N-(3-(((diphenylmethylene)amino)oxy)-3-oxopropyl)benzamide (**1-8**)
The title compound was obtained as a white solid (1.3 g, 56% yield).
NMR: ^{1}H NMR (400 MHz, Chloroform-*d*) δ 7.57 – 7.52 (m, 2H), 7.50 – 7.42 (m, 4H), 7.38 – 7.32 (m, 3H), 7.31 – 7.26 (m, 2H), 7.23 (dd, *J* = 7.8, 1.7 Hz, 2H), 7.19 – 7.15 (m, 1H), 3.58 – 3.53 (m, 2H), 2.53 – 2.47 (m, 2H), 1.48 (s, 9H). ^{13}C NMR (101 MHz, Chloroform-*d*) δ 171.6, 168.0, 165.4, 140.6, 134.4, 134.2, 132.2, 131.0, 129.9, 129.6, 129.1, 128.9, 128.4, 128.3, 128.2, 126.1, 124.0, 57.4, 42.4, 34.8, 28.7.
HRMS (APCI): calcd for $C_{27}H_{28}ClN_2O_3^+$ $[M + H]^+$: 463.1783; found: 463.1781.

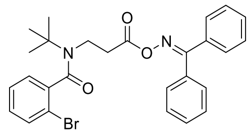

2-bromo-N-(tert-butyl)-N-(3-(((diphenylmethylene)amino)oxy)-3-oxopropyl)benzamide (**1-9**)
The title compound was obtained as a white solid (1.3 g, 50% yield).
NMR: ^{1}H NMR (400 MHz, Chloroform-*d*) δ 7.56 – 7.42 (m, 7H), 7.35 (t, *J* = 7.6 Hz, 2H), 7.29 (d, *J* = 7.7 Hz, 1H), 7.23 (d, *J* = 6.2 Hz, 2H), 7.20 – 7.14 (m, 2H), 3.64 – 3.49 (m, 1H), 3.45 – 3.28 (m, 1H), 2.69 – 2.54 (m, 1H), 2.50 – 2.35 (m, 1H), 1.52 (s, 9H). ^{13}C NMR (101 MHz, Chloroform-*d*) δ 169.6, 167.9, 165.3, 134.2, 132.8, 132.2, 131.0, 129.7, 129.6, 128.8, 128.4, 128.3, 128.1, 127.4, 127.1, 118.6, 57.7, 41.9, 34.7, 28.5.
HRMS (APCI): calcd for $C_{27}H_{28}BrN_2O_3^+$ $[M + H]^+$: 507.1278; found: 507.1276.

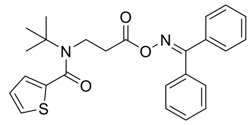

N-(tert-butyl)-N-(3-(((diphenylmethylene)amino)oxy)-3-oxopropyl)thiophene-2-carboxamide (**1-11**)
The title compound was obtained as a white solid (1.0 g, 46% yield).
NMR: ^{1}H NMR (400 MHz, Chloroform-*d*) δ 7.60 – 7.54 (m, 2H), 7.51 – 7.43 (m, 4H), 7.37 (t, *J* = 7.3 Hz, 3H), 7.30 – 7.25 (m, 2H), 7.21 (d, *J* = 3.3 Hz, 1H), 7.01 – 6.93 (m, 1H), 3.85 – 3.76 (m, 2H), 2.72 – 2.62 (m, 2H), 1.48 (s, 9H). ^{13}C NMR (101 MHz, Chloroform-*d*) δ 168.2, 166.3, 165.3, 140.0, 134.2, 132.2, 131.0, 129.6, 128.9, 128.5, 128.3, 128.2, 127.7, 127.3, 126.6, 57.9, 42.6, 35.5, 28.6.
HRMS (APCI): calcd for $C_{25}H_{27}N_2O_3S^+$ $[M + H]^+$: 435.1737; found: 435.1738.

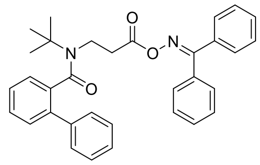

N-(tert-butyl)-N-(3-(((diphenylmethylene)amino)oxy)-3-oxopropyl)-[1,1'-biphenyl]-2-carboxamide (**1-12**)
The title compound was obtained as a colorless oil (1.0 g, 41% yield).

NMR: ^{1}H NMR (400 MHz, Chloroform-*d*) δ 7.54 (d, *J* = 7.8 Hz, 2H), 7.51 – 7.43 (m, 6H), 7.41 – 7.27 (m, 9H), 7.22 (d, *J* = 6.7 Hz, 2H), 3.26 – 3.07 (m, 2H), 2.21 (t, *J* = 8.1 Hz, 2H), 1.29 (s, 9H). ^{13}C NMR (101 MHz, Chloroform-*d*) δ 171.8, 168.0, 165.0, 139.6, 137.8, 137.4, 134.3, 132.3, 131.0, 129.6, 129.5, 129.0, 128.8, 128.7, 128.4, 128.3, 128.1, 127.4, 126.5, 57.2, 41.6, 34.3, 28.4.
HRMS (APCI): calcd for $C_{33}H_{33}N_2O_3^+$ $[M + H]^+$: 505.2486; found: 505.2484.

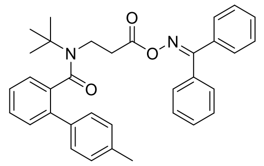

N-(tert-butyl)-N-(3-(((diphenylmethylene)amino)oxy)-3-oxopropyl)-4'-methyl-[1,1'-biphenyl]-2-carboxamide (**1-13**)
The title compound was obtained as a colorless oil (1.1 g, 42% yield).
NMR: ^{1}H NMR (400 MHz, Chloroform-*d*) δ 7.50 (d, *J* = 7.8 Hz, 2H), 7.47 – 7.39 (m, 4H), 7.37 – 7.27 (m, 7H), 7.25 – 7.16 (m, 3H), 7.12 (d, *J* = 7.7 Hz, 2H), 3.24 – 3.06 (m, 2H), 2.34 (s, 3H), 2.18 (t, *J* = 8.2 Hz, 2H), 1.28 (s, 9H). ^{13}C NMR (101 MHz, Chloroform-*d*) δ 172.0, 168.1, 165.0, 137.4, 137.1, 136.7, 134.3, 132.3, 131.0, 129.7, 129.6, 128.9, 128.8, 128.6, 128.5, 128.3, 128.1, 127.1, 126.6, 57.2, 41.6, 34.3, 28.4, 21.1.
HRMS (APCI): calcd for $C_{34}H_{35}N_2O_3^+$ $[M + H]^+$: 519.2642; found: 519.2639.

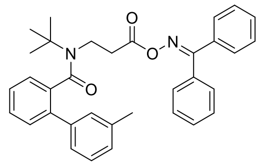

N-(tert-butyl)-N-(3-(((diphenylmethylene)amino)oxy)-3-oxopropyl)-3'-methyl-[1,1'-biphenyl]-2-carboxamide (**1-14**)
The title compound was obtained as a white solid (1.0 g, 40% yield).
NMR: ^{1}H NMR (400 MHz, Chloroform-*d*) δ 7.53 (d, *J* = 7.8 Hz, 2H), 7.49 – 7.41 (m, 4H), 7.38 – 7.29 (m, 6H), 7.26 – 7.18 (m, 5H), 7.13 (t, *J* = 4.6 Hz, 1H), 3.26 – 3.07 (m, 2H), 2.33 (s, 3H), 2.20 (t, *J* = 8.2 Hz, 2H), 1.29 (s, 9H). ^{13}C NMR (101 MHz, Chloroform-*d*) δ 171.9, 168.1, 165.0, 139.6, 137.8, 137.6, 137.5, 134.3, 132.3, 131.0, 129.8, 129.6, 129.6, 128.8, 128.6, 128.5, 128.3, 128.1, 127.3, 126.6, 126.0, 57.1, 41.6, 34.3, 28.4, 21.3.
HRMS (APCI): calcd for $C_{34}H_{35}N_2O_3^+$ $[M + H]^+$: 519.2642; found: 519.2639.

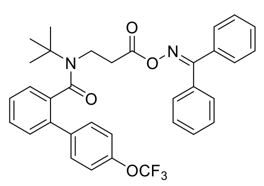

N-(tert-butyl)-N-(3-(((diphenylmethylene)amino)oxy)-3-oxopropyl)-4'-(trifluoromethoxy)-[1,1'-biphenyl]-2-carboxamide (**1-15**)
The title compound was obtained as a colorless oil (1.3 g, 45% yield).
NMR: ^{1}H NMR (400 MHz, Chloroform-*d*) δ 7.55 – 7.50 (m, 2H), 7.49 – 7.43 (m, 5H), 7.42 – 7.37 (m, 2H), 7.36 – 7.31 (m, 3H), 7.30 – 7.26 (m, 2H), 7.23 – 7.17 (m, 4H), 3.21 – 3.06 (m, 2H), 2.30 – 2.15 (m,

2H), 1.27 (s, 9H). ^{13}C NMR (101 MHz, Chloroform-*d*) δ 171.4, 167.8, 165.1, 148.5 (q, *J* = 1.8 Hz), 138.3, 135.9, 134.2, 132.2, 130.9, 130.5, 129.5, 129.5, 128.7, 128.3, 128.2, 128.1, 127.8, 126.4, 120.5, 57.2, 41.6, 34.3, 28.2. ^{19}F NMR (376 MHz, Chloroform-*d*) δ -57.8.
HRMS (APCI): calcd for $C_{34}H_{32}F_3N_2O_4^+$ $[M + H]^+$: 589.2309; found: 589.2305.

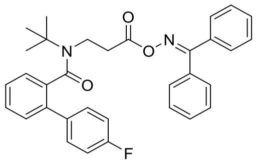

N-(tert-butyl)-N-(3-(((diphenylmethylene)amino)oxy)-3-oxopropyl)-4'-fluoro-[1,1'-biphenyl]-2-carboxamide (**1-16**)
The title compound was obtained as a white solid (1.1 g, 42% yield).
NMR: ^{1}H NMR (400 MHz, Chloroform-*d*) δ 7.52 (d, *J* = 7.1 Hz, 2H), 7.49 – 7.39 (m, 6H), 7.39 – 7.33 (m, 3H), 7.33 – 7.28 (m, 2H), 7.26 – 7.23 (m, 1H), 7.21 (d, *J* = 6.1 Hz, 2H), 7.02 (t, *J* = 8.7 Hz, 2H), 3.16 (t, *J* = 8.3 Hz, 2H), 2.21 (t, *J* = 8.1 Hz, 2H), 1.30 (s, 9H). ^{13}C NMR (101 MHz, Chloroform-*d*) δ 171.7, 168.0, 165.1, 163.5, 161.1, 137.8, 136.3, 135.7 (d, *J* = 3.4 Hz), 134.2, 132.3, 131.0, 130.8, 130.7, 129.7, 129.6, 128.8, 128.7, 128.4, 128.3, 128.2, 127.5, 126.5, 115.1, 114.9, 57.3, 41.6, 34.3, 28.4. ^{19}F NMR (376 MHz, Chloroform-*d*) δ -114.7.
HRMS (APCI): calcd for $C_{33}H_{32}FN_2O_3^+$ $[M + H]^+$: 523.2391; found: 523.2390.

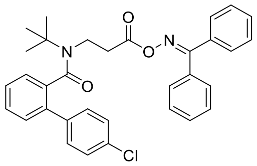

N-(tert-butyl)-4'-chloro-N-(3-(((diphenylmethylene)amino)oxy)-3-oxopropyl)-[1,1'-biphenyl]-2-carboxamide (**1-17**)
The title compound was obtained as a colorless oil (1.2 g, 46% yield).
NMR: ^{1}H NMR (400 MHz, Chloroform-*d*) δ 7.53 – 7.51 (m, 2H), 7.50 – 7.44 (m, 3H), 7.44 – 7.41 (m, 1H), 7.41 – 7.36 (m, 3H), 7.36 – 7.33 (m, 2H), 7.32 – 7.29 (m, 3H), 7.28 – 7.23 (m, 2H), 7.23 – 7.19 (m, 2H), 3.16 (t, *J* = 8.0 Hz, 2H), 2.21 (t, *J* = 8.1 Hz, 2H), 1.30 (s, 9H). ^{13}C NMR (101 MHz, Chloroform-*d*) δ 171.6, 167.9, 165.1, 138.1, 137.7, 136.0, 134.2, 133.6, 132.2, 131.0, 130.4, 129.6, 128.8, 128.8, 128.4, 128.3, 128.1, 127.7, 126.6, 57.3, 41.6, 34.3, 28.4.
HRMS (APCI): calcd for $C_{33}H_{32}ClN_2O_3^+$ $[M + H]^+$: 539.2096; found: 539.2095.

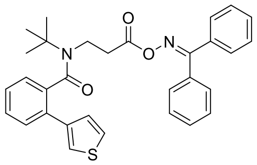

N-(tert-butyl)-N-(3-(((diphenylmethylene)amino)oxy)-3-oxopropyl)-2-(thiophen-3-yl)benzamide (**1-18**)
The title compound was obtained as a colorless oil (1.2 g, 46% yield).
NMR: ^{1}H NMR (400 MHz, Chloroform-*d*) δ 7.52 (d, *J* = 7.1 Hz, 2H), 7.50 – 7.44 (m, 3H), 7.43 – 7.39 (m, 2H), 7.38 – 7.31 (m, 4H), 7.31 – 7.27 (m, 1H), 7.26 – 7.24 (m, 1H), 7.23 – 7.18 (m, 4H), 3.21 (t, *J* = 8.1 Hz, 2H), 2.25 – 2.03 (m, 2H), 1.39 (s, 9H). ^{13}C NMR (101 MHz, Chloroform-*d*) δ 172.1, 168.0, 165.0,

140.0, 137.6, 134.2, 132.3, 132.0, 130.9, 129.5, 129.2, 128.8, 128.5, 128.4, 128.4, 128.3, 128.1, 127.3, 126.3, 125.4, 123.6, 57.3, 41.8, 34.1, 28.4.

HRMS (APCI): calcd for $C_{31}H_{31}N_2O_3S^+$ $[M + H]^+$: 511.2050; found: 511.2049.

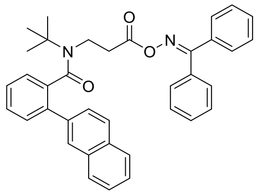

N-(tert-butyl)-N-(3-(((diphenylmethylene)amino)oxy)-3-oxopropyl)-2-(naphthalen-2-yl)benzamide (**1-19**)

The title compound was obtained as a colorless oil (0.97 g, 35% yield).

NMR: ^{1}H NMR (400 MHz, Chloroform-*d*) δ 8.00 (s, 1H), 7.88 – 7.80 (m, 3H), 7.61 (dd, *J* = 8.4, 1.8 Hz, 1H), 7.55 – 7.51 (m, 2H), 7.51 – 7.47 (m, 2H), 7.47 – 7.44 (m, 3H), 7.43 – 7.38 (m, 3H), 7.38 – 7.30 (m, 4H), 7.21 – 7.18 (m, 2H), 3.32 – 3.19 (m, 1H), 3.15 – 3.00 (m, 1H), 2.20 (t, *J* = 8.1 Hz, 2H), 1.24 (s, 9H). ^{13}C NMR (101 MHz, Chloroform-*d*) δ 172.0, 168.0, 165.1, 138.0, 137.2, 137.0, 134.3, 133.0, 132.4, 132.2, 131.0, 130.0, 129.5, 128.8, 128.8, 128.4, 128.3, 128.2, 128.1, 128.0, 127.9, 127.5, 127.5, 127.1, 126.8, 126.1, 126.0, 57.2, 41.6, 34.4, 28.4.

HRMS (APCI): calcd for $C_{37}H_{35}N_2O_3^+$ $[M + H]^+$: 555.2642; found: 555.2638.

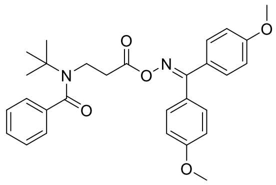

N-(3-(((bis(4-methoxyphenyl)methylene)amino)oxy)-3-oxopropyl)-N-(tert-butyl)benzamide (**1-20**)

The title compound was obtained as a white solid (1.3 g, 53% yield).

NMR: ^{1}H NMR (400 MHz, Chloroform-*d*) δ 7.47 (d, *J* = 8.8 Hz, 2H), 7.36 – 7.31 (m, 3H), 7.31 – 7.27 (m, 2H), 7.17 (d, *J* = 8.7 Hz, 2H), 6.94 (d, *J* = 8.7 Hz, 2H), 6.85 (d, *J* = 8.8 Hz, 2H), 3.87 (s, 3H), 3.80 (s, 3H), 3.62 – 3.56 (m, 2H), 2.55 – 2.48 (m, 2H), 1.50 (s, 9H). ^{13}C NMR (101 MHz, Chloroform-*d*) δ 173.3, 168.3, 164.6, 161.7, 160.4, 139.0, 130.6, 130.6, 128.9, 128.4, 127.0, 125.8, 124.4, 113.6, 113.4, 57.1, 55.2, 42.4, 34.9, 28.8.

HRMS (APCI): calcd for $C_{29}H_{33}N_2O_5^+$ $[M + H]^+$: 489.2384; found: 489.2386.

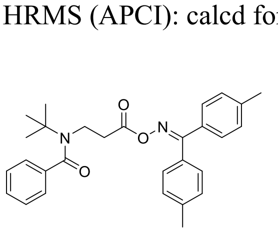

N-(tert-butyl)-N-(3-(((di-p-tolylmethylene)amino)oxy)-3-oxopropyl)benzamide (**1-21**)

The title compound was obtained as a white solid (1.2 g, 54% yield).

NMR: ^{1}H NMR (400 MHz, Chloroform-*d*) δ 7.41 (d, *J* = 8.2 Hz, 2H), 7.36 – 7.31 (m, 3H), 7.30 – 7.26 (m, 2H), 7.24 (d, *J* = 7.9 Hz, 2H), 7.14 (d, *J* = 7.9 Hz, 2H), 7.10 (d, *J* = 8.1 Hz, 2H), 3.59 – 3.53 (m, 2H), 2.52 – 2.47 (m, 2H), 2.44 (s, 3H), 2.35 (s, 3H), 1.48 (s, 9H). ^{13}C NMR (101 MHz, Chloroform-*d*) δ 173.4,

168.3, 141.3, 139.6, 139.0, 131.7, 129.4, 129.0, 128.9, 128.7, 128.6, 128.4, 125.8, 57.1, 42.4, 34.9, 28.8, 21.4, 21.3.

HRMS (APCI): calcd for $C_{29}H_{33}N_2O_3^+$ [M + H]$^+$: 457.2486; found: 457.2483.

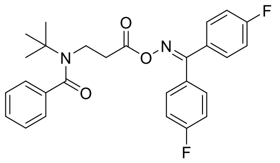

N-(3-(((bis(4-fluorophenyl)methylene)amino)oxy)-3-oxopropyl)-N-(tert-butyl)benzamide (**1-22**)

The title compound was obtained as a colorless oil (1.1 g, 50% yield).

NMR: ^{1}H NMR (400 MHz, Chloroform-*d*) δ 7.50 (dd, *J* = 8.7, 5.5 Hz, 2H), 7.35 – 7.30 (m, 3H), 7.29 – 7.24 (m, 2H), 7.21 – 7.17 (m, 2H), 7.13 (t, *J* = 8.6 Hz, 2H), 7.02 (t, *J* = 8.6 Hz, 2H), 3.60 – 3.54 (m, 2H), 2.51 – 2.46 (m, 2H), 1.47 (s, 9H). ^{13}C NMR (101 MHz, Chloroform-*d*) δ 173.3, 167.9, 165.0 (d, *J* = 132.1 Hz), 163.1, 162.5 (d, *J* = 130.5 Hz), 138.9, 130.9 (d, *J* = 8.8 Hz), 130.7 (d, *J* = 8.4 Hz), 130.3 (d, *J* = 3.3 Hz), 128.9, 128.3, 127.8 (d, *J* = 3.6 Hz), 125.8, 115.6 (d, *J* = 7.2 Hz), 115.4 (d, *J* = 7.2 Hz), 57.1, 42.4, 34.8, 28.8. ^{19}F NMR (376 MHz, Chloroform-*d*) δ -108.3, -109.5.

HRMS (APCI): calcd for $C_{27}H_{27}F_2N_2O_3^+$ [M + H]$^+$: 465.1984; found: 465.1979.

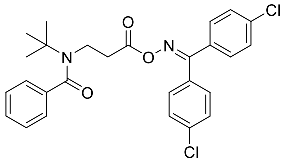

N-(3-(((bis(4-chlorophenyl)methylene)amino)oxy)-3-oxopropyl)-N-(tert-butyl)benzamide (**1-23**)

The title compound was obtained as a white solid (1.2 g, 49% yield).

NMR: ^{1}H NMR (400 MHz, Chloroform-*d*) δ 7.49 – 7.42 (m, 4H), 7.38 – 7.32 (m, 5H), 7.31 – 7.26 (m, 2H), 7.15 (d, *J* = 8.1 Hz, 2H), 3.62 – 3.55 (m, 2H), 2.56 – 2.46 (m, 2H), 1.50 (s, 9H). ^{13}C NMR (101 MHz, Chloroform-*d*) δ 173.3, 167.8, 163.0, 139.0, 137.5, 136.0, 132.4, 130.0, 129.9, 129.0, 128.7, 128.7, 128.4, 125.9, 57.1, 42.4, 34.8, 28.8.

HRMS (APCI): calcd for $C_{27}H_{27}Cl_2N_2O_3^+$ [M + H]$^+$: 497.1393; found: 497.1386.

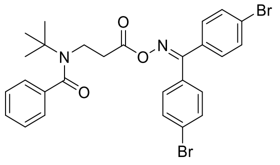

N-(3-(((bis(4-bromophenyl)methylene)amino)oxy)-3-oxopropyl)-N-(tert-butyl)benzamide (**1-24**)

The title compound was obtained as a white solid (1.3 g, 44% yield).

NMR: ^{1}H NMR (400 MHz, Chloroform-*d*) δ 7.59 (d, *J* = 8.4 Hz, 2H), 7.50 – 7.46 (m, 2H), 7.39 – 7.32 (m, 5H), 7.29 – 7.25 (m, 2H), 7.06 (d, *J* = 8.8 Hz, 2H), 3.57 (d, *J* = 7.4 Hz, 2H), 2.48 (d, *J* = 7.5 Hz, 2H), 1.48 (s, 9H). ^{13}C NMR (101 MHz, Chloroform-*d*) δ 173.2, 167.7, 163.0, 138.9, 132.7, 131.6, 131.6, 130.4, 130.1, 130.0, 128.9, 128.3, 125.9, 125.8, 124.2, 57.1, 42.3, 34.7, 28.8.

HRMS (APCI): calcd for $C_{27}H_{27}Br_2N_2O_3^+$ [M + H]$^+$: 585.0383; found: 585.0376.

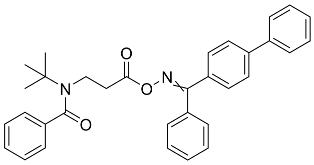

N-(3-((([1,1'-biphenyl]-4-yl(phenyl)methylene)amino)oxy)-3-oxopropyl)-N-(tert-butyl)benzamide (**1-25**)

The title compound was obtained as a colorless oil (1.0 g, 40% yield).

NMR: ^{1}H NMR (400 MHz, Chloroform-*d*) δ 7.71 – 7.67 (m, 2H), 7.65 – 7.56 (m, 4H), 7.53 – 7.48 (m, 2H), 7.44 (t, *J* = 7.9 Hz, 2H), 7.40 – 7.37 (m, 1H), 7.37 – 7.34 (m, 2H), 7.33 – 7.25 (m, 6H), 3.64 – 3.56 (m, 2H), 2.60 – 2.48 (m, 2H), 1.50 (s, 9H). ^{13}C NMR (101 MHz, Chloroform-*d*) δ 173.3, 168.2, 168.0, 164.9, 164.9, 143.7, 142.4, 139.8, 139.8, 139.0, 134.3, 133.0, 132.2, 131.0, 131.0, 129.6, 129.3, 129.2, 128.9, 128.9, 128.8, 128.4, 128.4, 128.3, 128.3, 128.2, 127.9, 127.8, 127.0, 126.9, 126.7, 125.8, 57.1, 42.4, 34.9, 28.8.

HRMS (APCI): calcd for C33H33N2O3^{+} [M + H]$^{+}$: 505.2486; found: 505.2484.

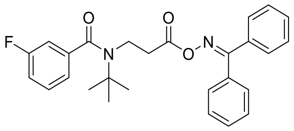

N-(tert-butyl)-N-(3-(((diphenylmethylene)amino)oxy)-3-oxopropyl)-3-fluorobenzamide (**1-26**)

The title compound was obtained as a white solid (1.1 g, 49% yield).

NMR: ^{1}H NMR (400 MHz, Chloroform-*d*) δ 7.58 – 7.53 (m, 2H), 7.52 – 7.42 (m, 4H), 7.40 – 7.30 (m, 3H), 7.26 – 7.21 (m, 2H), 7.10 – 7.04 (m, 2H), 7.03 – 6.99 (m, 1H), 3.61 – 3.54 (m, 2H), 2.55 – 2.48 (m, 2H), 1.49 (s, 9H). ^{13}C NMR (101 MHz, Chloroform-*d*) δ 171.7, 167.9, 165.4, 162.4 (d, *J* = 248.1 Hz), 140.9 (d, *J* = 6.9 Hz), 134.2, 132.2, 131.0, 130.3 (d, *J* = 8.0 Hz), 129.6, 128.8, 128.4, 128.3, 128.1, 121.5 (d, *J* = 3.1 Hz), 116.0 (d, *J* = 21.0 Hz), 113.2 (d, *J* = 22.7 Hz), 57.3, 42.3, 34.8, 28.7. ^{19}F NMR (376 MHz, Chloroform-*d*) δ -111.5.

HRMS (APCI): calcd for $C_{27}H_{28}FN_2O_3^+$ [M + H]$^{+}$: 447.2078; found: 447.2072.

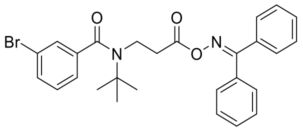

2-bromo-N-(tert-butyl)-N-(3-(((diphenylmethylene)amino)oxy)-3-oxopropyl)benzamide (**1-27**)

The title compound was obtained as a white solid (1.1 g, 45% yield).

NMR: ^{1}H NMR (400 MHz, Chloroform-*d*) δ 7.54 (d, *J* = 7.1 Hz, 2H), 7.52 – 7.40 (m, 6H), 7.35 (t, *J* = 7.5 Hz, 2H), 7.22 (t, *J* = 5.3 Hz, 4H), 3.58 – 3.51 (m, 2H), 2.52 – 2.45 (m, 2H), 1.46 (s, 9H). ^{13}C NMR (101 MHz, Chloroform-*d*) δ 171.5, 168.0, 165.4, 140.8, 134.2, 132.1, 131.0, 130.1, 129.7, 129.0, 128.9, 128.5, 128.4, 128.2, 124.5, 122.5, 57.4, 42.4, 34.8, 28.7.

HRMS (APCI): calcd for $C_{27}H_{28}BrN_2O_3^+$ [M + H]$^{+}$: 507.1278; found: 507.1273.

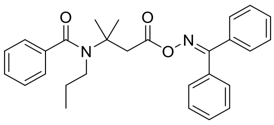

N-(4-(((diphenylmethylene)amino)oxy)-2-methyl-4-oxobutan-2-yl)-N-propylbenzamide (**1-28**)
The title compound was obtained as a colorless oil (930 mg, 42% yield).
NMR: ^{1}H NMR (400 MHz, Chloroform-*d*) δ 7.62 – 7.55 (m, 2H), 7.46 – 7.41 (m, 4H), 7.38 – 7.28 (m, 9H), 3.26 – 3.20 (m, 4H), 1.57 (s, 6H), 1.53 – 1.39 (m, 2H), 0.59 (t, J = 7.4 Hz, 3H). ^{13}C NMR (101 MHz, Chloroform-*d*) δ 173.1, 168.8, 164.8, 139.3, 134.8, 132.5, 130.7, 129.5, 129.0, 128.8, 128.4, 128.2, 128.1, 128.1, 126.0, 57.4, 49.4, 41.3, 27.4, 25.2, 10.9.
HRMS (APCI): calcd for $C_{28}H_{31}N_2O_3^+$ [M + H]$^+$: 443.2329; found: 443.2325.

III.2 Method II

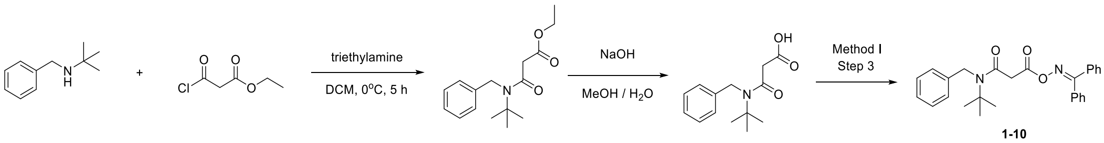

1-10

Step 1:

To a solution of N-benzyl-2-methylpropan-2-amine (5.0 mmol, 1.0 eq) and triethylamine (7.5 mmol, 1.5 eq) in dry DCM (30 mL) was slowly added ethyl 3-chloro-3-oxopropanoate (5 mmol, 1.0 eq) at 0 °C. When the addition was complete the reaction mixture was stirred at room temperature for another 8 hours. The reaction mixture was quenched with water and extracted with DCM three times. The combined organic layers were washed with brine, dried over sodium sulfate, filtered, concentrated, and used for the next step without further purification.

Step 2:

To the solution of crude product from step 1 in MeOH/H_2O (20 mL, v/v=4/1) was added NaOH (7.5 mmol, 1.5 eq) in one portion, and this reaction solution was stirred at room temperature for 4 hours. The reaction solution was carefully adjusted to pH 3~5 by 1M HCl (aq). After removing MeOH under reduced pressure the reaction mixture was extracted with DCM three times. The combined organic layers were washed with brine, dried over sodium sulfate, filtered, concentrated, and purified by recrystallization from petroleum ether and EtOAc to give 3-(benzyl(tert-butyl)amino)-3-oxopropanoic acid.

Step 3:

To a solution of the acid from step 2, diphenylmethanone oxime (4.5 mmol, 0.9 eq) and DMAP (0.25 mmol, 5 mol%) in dry DCM (30 mL), was added EDCI (12.5 mmol, 2.5 eq) in portions at room temperature and stirred for 8 hours. The reaction mixture was quenched with water and extracted with CH_2Cl_2 three times. The combined organic layers were washed with brine, dried over sodium sulfate, filtered, concentrated, and purified by column chromatography on silica gel (10 : 1 petroleum ether : EtOAc) to give the starting materials **1-10**.

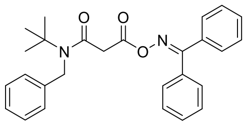

N-benzyl-N-(tert-butyl)-3-(((diphenylmethylene)amino)oxy)-3-oxopropanamide (**1-10**)
The title compound was obtained as a brown-gray solid (1.2 g, 56% yield).
NMR: ^{1}H NMR (400 MHz, Chloroform-*d*) δ 7.56 (d, J = 7.1 Hz, 2H), 7.51 – 7.41 (m, 4H), 7.40 – 7.31 (m, 4H), 7.30 – 7.20 (m, 4H), 7.11 (d, J = 6.2 Hz, 2H), 4.47 (s, 2H), 3.46 (s, 2H), 1.40 (s, 9H). 13C NMR (101 MHz, Chloroform-*d*) δ 166.7, 165.8, 164.8, 138.3, 134.6, 132.4, 130.8, 129.6, 129.0, 129.0, 128.8, 128.2, 128.1, 127.1, 125.3, 58.3, 49.0, 42.8, 28.4.

HRMS (APCI): calcd for $C_{27}H_{29}N_2O_3^+$ [M + H]$^+$: 429.2173; found: 429.2175.

III.3 Method III

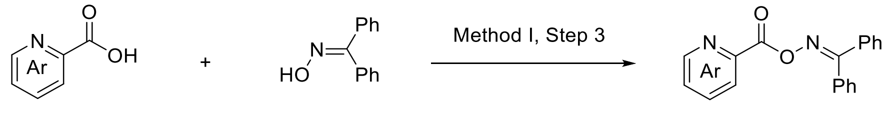

To a solution of picolinic acid (5.0 mmol), diphenylmethanone oxime (4.5 mmol, 0.9 eq) and DMAP (0.25 mmol, 5 mol%) in dry DCM (30 mL), was added EDCI (12.5 mmol, 2.5 eq) in portions at room temperature and stirred for 8 hours. The reaction mixture was quenched with water and extracted with CH_2Cl_2 three times. The combined organic layers were washed with brine, dried over sodium sulfate, filtered, concentrated, and purified by column chromatography on silica gel (4 : 1 petroleum ether : EtOAc) to give the starting materials **5**.

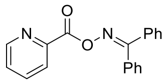

diphenylmethanone *O*-picolinoyl oxime (**5-1**)

The title compound was obtained as a white solid (1.2 g, 80% yield), it has been reported.[5]

NMR: ^{1}H NMR (400 MHz, Chloroform-*d*) δ 8.71 (d, *J* = 4.1 Hz, 1H), 7.73 – 7.62 (m, 4H), 7.49 – 7.39 (m, 7H), 7.37 (t, *J* = 7.7 Hz, 2H). ^{13}C NMR (101 MHz, Chloroform-*d*) δ 166.4, 162.1, 150.2, 146.8, 136.8, 134.4, 132.4, 131.0, 129.7, 129.1, 128.9, 128.3, 128.2, 126.9, 125.0.

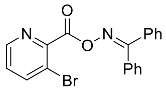

diphenylmethanone *O*-(3-bromopicolinoyl) oxime (**5-2**)

The title compound was obtained as a yellow solid (1.2 g, 65% yield), it has been reported.[5]

NMR: ^{1}H NMR (400 MHz, Chloroform-*d*) δ 8.49 (dd, *J* = 4.6, 1.4 Hz, 1H), 7.86 (dd, *J* = 8.2, 1.4 Hz, 1H), 7.62 – 7.56 (m, 2H), 7.46 – 7.42 (m, 1H), 7.39 (s, 5H), 7.35 (t, *J* = 7.6 Hz, 2H), 7.19 (dd, *J* = 8.2, 4.6 Hz, 1H). ^{13}C NMR (101 MHz, Chloroform-*d*) δ 166.3, 163.1, 149.1, 147.6, 141.1, 134.3, 131.9, 131.0, 129.7, 129.1, 129.0, 128.3, 128.0, 126.2, 118.4.

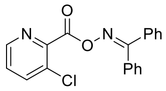

diphenylmethanone *O*-(3-chloropicolinoyl) oxime (**5-3**)

The title compound was obtained as a yellow solid (1.1 g, 68% yield).

NMR: ^{1}H NMR (400 MHz, Chloroform-*d*) δ 8.48 (dd, *J* = 4.6, 1.4 Hz, 1H), 7.70 (dd, *J* = 8.2, 1.4 Hz, 1H), 7.62 – 7.57 (m, 2H), 7.47 – 7.34 (m, 8H), 7.30 (dd, *J* = 8.2, 4.6 Hz, 1H). ^{13}C NMR (101 MHz, Chloroform-*d*) δ 166.4, 162.5, 147.3, 138.1, 134.4, 132.1, 131.1, 130.5, 129.7, 129.1, 129.0, 128.3, 128.0, 126.2.

HRMS (APCI): calcd for $C_{19}H_{14}ClN_2O_2^+$ [M + H]$^+$: 337.0738; found: 337.0733.

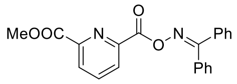

methyl 6-((((diphenylmethylene)amino)oxy)carbonyl)picolinate (**5-4**)

The title compound was obtained as a beige solid (1.2 g, 70% yield), it has been reported.[5]

NMR: ^{1}H NMR (400 MHz, Chloroform-*d*) δ 8.22 (dd, *J* = 7.6, 1.4 Hz, 1H), 7.95 (dd, *J* = 7.8, 1.4 Hz, 1H), 7.90 (t, *J* = 7.7 Hz, 1H), 7.66 – 7.59 (m, 2H), 7.48 (s, 5H), 7.44 (d, *J* = 7.4 Hz, 1H), 7.36 (t, *J* = 7.5 Hz, 2H), 3.96 (s, 3H). ^{13}C NMR (101 MHz, Chloroform-*d*) δ 166.3, 164.9, 161.5, 148.3, 147.1, 138.2, 134.4, 132.1, 131.0, 129.8, 129.3, 129.2, 128.3, 128.1, 127.8, 127.8, 52.8.

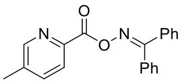

diphenylmethanone *O*-(5-methylpicolinoyl) oxime (**5-5**)

The title compound was obtained as a yellow solid (1.3 g, 82% yield).

NMR: ^{1}H NMR (400 MHz, Chloroform-*d*) δ 8.48 (s, 1H), 7.59 (d, *J* = 7.7 Hz, 2H), 7.53 (d, *J* = 8.0 Hz, 1H), 7.47 – 7.39 (m, 5H), 7.39 – 7.35 (m, 2H), 7.31 (t, *J* = 7.6 Hz, 2H), 2.29 (s, 3H). ^{13}C NMR (101 MHz, Chloroform-*d*) δ 166.0, 162.0, 150.5, 143.8, 137.3, 136.8, 134.2, 132.2, 130.8, 129.5, 128.9, 128.6, 128.1, 127.9, 124.4, 18.3.

HRMS (APCI): calcd for $C_{20}H_{17}N_2O_2^+$ [M + H]$^+$: 317.1285; found: 317.1279.

IV. Investigations of reaction conditions

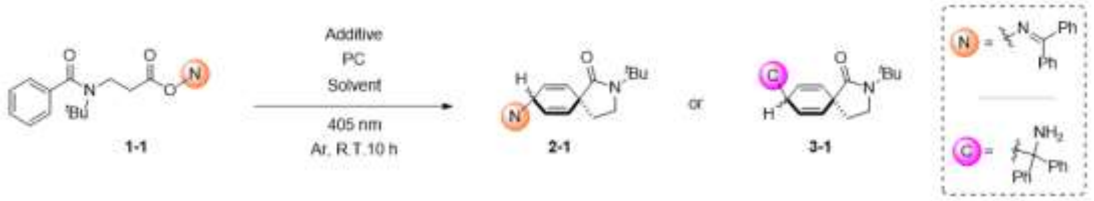

Entry	Solvent	Additives	Variation	2-1	cis/trans	3-1
	Conditions			[b]Yield (%)		
1	MeCN	-	PC-I	34	1 : 3.0	0
2	DCE	A-1	PC-I	35	1 : 2.6	0
3	EA	A-1	PC-I	39	1 : 3.6	0
4	MeCN	A-1	PC-I	60	1 : 2.5	0
5	MeCN / EA (2/1, v/v)	A-1	PC-I	62	1 : 2.4	0
6	MeCN / EA (2/1, v/v)	A-1	other PC / no *hv* or PC	0		0
7	THF	A-1	PC-I	33	1 : 2.9	0
8	DMF	A-1	PC-I	24	1 : 2.7	0
9	EA	A-2	PC-I	0	2.0 : 1	25
10	DMPU	A-1	PC-I	0	1.7 : 1	37
11	DMPU / EA (1/1, v/v)	A-1	PC-I	0	1.8 : 1	55
12	DMPU / EA (1/1, v/v)	A-3	PC-I	0	1.8 : 1	60
[c]13	DMPU / EA (1/1, v/v)	A-3	PC-I	0	1.9 : 1	69
14	DMPU / EA (1/1, v/v)	A-3	other PC / no *hv* or PC	0		0

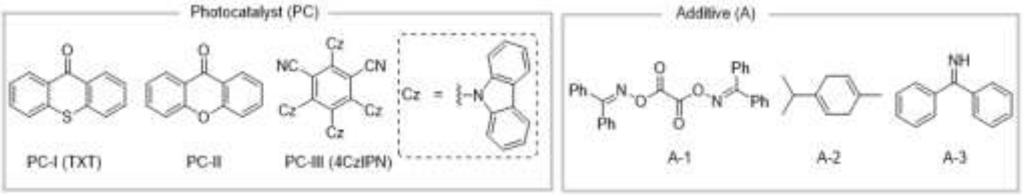

Standard conditions: The reactions were carried out under argon using **1-1** (0.1 mmol), Additive (1.0 equiv), PC (5 mol%), Solvents for **2-1** (0.033 M), Solvents for **3-1** (0.05 M) at room temperature for 10 hours. [b]The yield of **2-1** and **3-1** were based on ^{1}H NMR analysis of the crude product using

CH_2Br_2 as the internal standard. [c]Additive (2.0 equiv). EA = ethyl acetate, DMPU = 1,3-dimethyltetrahydropyrimidin-2(1H)-one.

Notes: For product **3-1**, various potential HAT solvents, including NMP, cyclohexane, 1,4-dioxane, CHCl3 and 1,3-dimethylimidazolidin-2-one were explored. However, C-C coupling product formation was not observed in any of these solvents.

V. General procedure for the chemodivergent reactions.

V.1 Carboamination

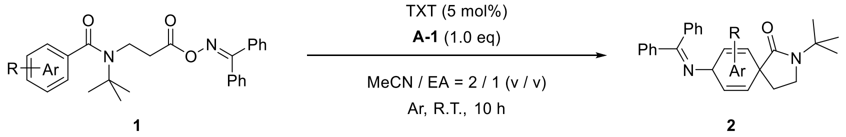

Standard Conditions: O-oxime ester **1** (0.2 mmol), **A-1** (0.2 mmol, 1.0 equiv), **TXT** (5 mol%) in 6 mL of MeCN/EA (0.033 M, *v*/*v* = 2/1) under an Ar atmosphere at R.T. and irradiated with visible LEDs (2 x 15 W, 405 nm) for 10 h.

Workup: After removing the solvent under reduced pressure, the crude product was purified by column chromatography on pre-basified silica gel using a PE/EtOAc eluent system containing 1% (v/v) triethylamine (TEA) to isolate all isomers of the target product **2**. Isolated yields were reported as the average of four experiments.

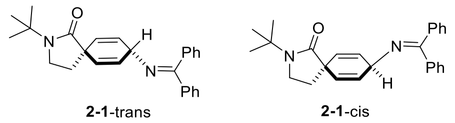

2-(tert-butyl)-8-((diphenylmethylene)amino)-2-azaspiro[4.5]deca-6,9-dien-1-one (**2-1**)

2-1-trans was obtained (132 mg, 43% yield) as a brown oil.

2-1-cis was obtained (55 mg, 18% yield) as a brown oil, which can become solid under lower temperature.

d.r. (cis:trans) = 1:2.4

NMR:

[trans]: ^{1}H NMR (400 MHz, Chloroform-*d*) δ 7.64 – 7.60 (m, 2H), 7.45 – 7.40 (m, 3H), 7.35 – 7.27 (m, 3H), 7.20 – 7.14 (m, 2H), 5.75 (dd, *J* = 10.1, 2.7 Hz, 2H), 5.69 (dd, *J* = 10.1, 1.9 Hz, 2H), 4.70 – 4.66 (m, 1H), 3.48 (t, *J* = 6.8 Hz, 2H), 2.11 (t, *J* = 6.8 Hz, 2H), 1.37 (s, 9H). ^{13}C NMR (101 MHz, Chloroform-*d*) δ 174.7, 169.2, 139.5, 136.5, 130.0, 128.8, 128.6, 128.6, 128.4, 127.8, 127.4, 127.4, 55.4, 54.1, 49.1, 42.1, 32.5, 27.4.

[cis]: ^{1}H NMR (400 MHz, Chloroform-*d*) δ 7.64 (d, *J* = 7.2 Hz, 2H), 7.50 – 7.39 (m, 3H), 7.37 – 7.25 (m, 3H), 7.22 – 7.15 (m, 2H), 5.71 (s, 4H), 4.45 (s, 1H), 3.44 (t, *J* = 6.8 Hz, 2H), 1.90 (t, *J* = 6.8 Hz, 2H), 1.40 (s, 9H). ^{13}C NMR (101 MHz, Chloroform-*d*) δ 174.8, 169.2, 139.1, 136.8, 130.2, 128.8, 128.6, 128.4, 128.3, 128.0, 128.0, 127.5, 55.6, 54.3, 48.6, 42.1, 33.4, 27.6.

HRMS (APCI): calcd for $C_{26}H_{29}N_2O^+$ $[M + H]^+$: 385.2274; found: 385.2270.

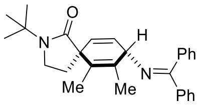

2-2-trans

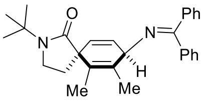

2-2-cis

2-(tert-butyl)-8-((diphenylmethylene)amino)-6,7-dimethyl-2-azaspiro[4.5]deca-6,9-dien-1-one (**2-2**)

2-2-trans was obtained (149 mg, 45% yield) as a brown oil.

2-2-cis was obtained (79 mg, 24% yield) as a brown oil.

d.r. (cis:trans) = 1:1.9

NMR:

[trans]: ^{1}H NMR (400 MHz, Chloroform-*d*) δ 7.66 – 7.60 (m, 2H), 7.44 – 7.39 (m, 3H), 7.35 – 7.27 (m, 3H), 7.21 – 7.15 (m, 2H), 5.70 (dd, *J* = 9.8, 1.7 Hz, 1H), 5.57 (dd, *J* = 9.8, 3.4 Hz, 1H), 4.50 (s, 1H), 3.52 – 3.45 (m, 2H), 2.37 – 2.27 (m, 1H), 2.02 – 1.95 (m, 1H), 1.68 (s, 3H), 1.61 (s, 3H), 1.39 (s, 9H). ^{13}C NMR (101 MHz, Chloroform-*d*) δ 175.4, 169.2, 139.7, 136.9, 129.9, 129.5, 129.1, 128.5, 128.5, 128.3, 127.8, 127.5, 126.8, 126.3, 60.7, 54.2, 53.3, 42.8, 30.3, 27.4, 16.7, 14.6.

[cis]: ^{1}H NMR (400 MHz, Chloroform-*d*) δ 7.68 (d, *J* = 7.5 Hz, 2H), 7.49 – 7.40 (m, 3H), 7.37 – 7.26 (m, 3H), 7.20 (d, *J* = 7.2 Hz, 2H), 5.70 (d, *J* = 9.9 Hz, 1H), 5.51 (dd, *J* = 9.9, 3.4 Hz, 1H), 4.29 (s, 1H), 3.50 – 3.40 (m, 2H), 2.19 – 2.09 (m, 1H), 1.78 – 1.71 (m, 1H), 1.67 (s, 3H), 1.58 (s, 3H), 1.42 (s, 9H). ^{13}C NMR (101 MHz, Chloroform-*d*) δ 175.0, 167.9, 139.5, 137.2, 129.8, 129.1, 128.8, 128.5, 128.1, 127.8, 127.6, 126.5, 126.2, 60.5, 54.2, 53.0, 42.6, 30.7, 27.5, 16.6, 14.6.

HRMS (APCI): calcd for $C_{28}H_{33}N_2O^+$ $[M + H]^+$: 413.2587; found: 413.2582.

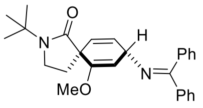

2-3-trans

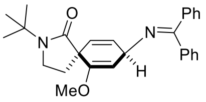

2-3-cis

2-(tert-butyl)-8-((diphenylmethylene)amino)-6-methoxy-2-azaspiro[4.5]deca-6,9-dien-1-one (**2-3**)

2-3-trans was obtained (159 mg, 48% yield) as a brown oil.

2-3-cis was obtained (64 mg, 19% yield) as a brown oil.

d.r. (cis:trans) = 1:2.5

NMR:

[trans]: ^{1}H NMR (400 MHz, Chloroform-*d*) δ 7.66 – 7.58 (m, 2H), 7.44 – 7.38 (m, 3H), 7.35 – 7.27 (m, 3H), 7.19 – 7.15 (m, 2H), 5.69 – 5.63 (m, 1H), 5.58 (dd, *J* = 9.6, 1.7 Hz, 1H), 4.89 – 4.84 (m, 1H), 4.65 (dd, *J* = 3.6, 1.4 Hz, 1H), 3.55 (s, 3H), 3.53 – 3.46 (m, 1H), 3.45 – 3.38 (m, 1H), 2.52 – 2.44 (m, 1H), 2.01 – 1.92 (m, 1H), 1.36 (s, 9H). ^{13}C NMR (101 MHz, Chloroform-*d*) δ 174.2, 168.5, 155.7, 139.7, 136.7, 129.9, 128.6, 128.5, 128.3, 128.3, 128.2, 127.8, 127.4, 96.3, 57.4, 54.2, 54.2, 51.5, 43.4, 29.9, 27.3.

[cis]: ^{1}H NMR (400 MHz, Chloroform-*d*) δ 7.64 (d, *J* = 6.9 Hz, 2H), 7.48 – 7.41 (m, 3H), 7.34 – 7.27 (m, 3H), 7.19 (d, *J* = 6.6 Hz, 2H), 5.65 – 5.56 (m, 2H), 4.65 – 4.59 (m, 2H), 3.54 (s, 3H), 3.50 – 3.44 (m, 1H), 3.42 – 3.36 (m, 1H), 2.26 – 2.18 (m, 1H), 1.83 – 1.75 (m, 1H), 1.39 (s, 9H). ^{13}C NMR (101 MHz, Chloroform-*d*) δ 174.0, 168.3, 156.5, 139.4, 137.1, 130.2, 128.9, 128.6, 128.6, 128.4, 128.0, 127.6, 127.6, 96.3, 57.5, 54.5, 54.5, 51.3, 43.4, 31.0, 27.5.

HRMS (APCI): calcd for $C_{27}H_{31}N_2O_2^+$ $[M + H]^+$: 415.2380; found: 415.2374.

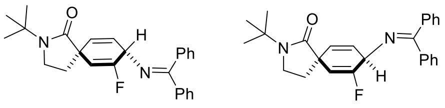

2-4-trans **2-4**-cis

2-(tert-butyl)-8-((diphenylmethylene)amino)-7-fluoro-2-azaspiro[4.5]deca-6,9-dien-1-one (**2-4**)
2-4-trans was obtained (116 mg, 36% yield) as a brown oil.
2-4-cis was obtained (74 mg, 23% yield) as a brown oil.
d.r. (cis:trans) = 1:1.5
NMR:
[trans]: ^{1}H NMR (400 MHz, Chloroform-*d*) δ 7.64 (d, *J* = 7.6 Hz, 2H), 7.46 – 7.39 (m, 3H), 7.35 (d, *J* = 7.1 Hz, 1H), 7.29 (t, *J* = 7.4 Hz, 2H), 7.19 (d, *J* = 7.6 Hz, 2H), 5.67 – 5.60 (m, 1H), 5.60 – 5.52 (m, 1H), 5.25 (d, *J* = 15.6 Hz, 1H), 4.86 (s, 1H), 3.45 (t, *J* = 6.8 Hz, 2H), 2.19 – 2.06 (m, 2H), 1.36 (s, 9H). ^{13}C NMR (101 MHz, Chloroform-*d*) δ 174.1 (d, *J* = 3.1 Hz), 171.7, 159.1 (d, *J* = 259.4 Hz), 139.2, 136.1, 130.2, 128.7, 128.5, 128.5, 127.9, 127.7 (d, *J* = 2.4 Hz), 127.6, 127.0 (d, *J* = 7.2 Hz), 104.5 (d, *J* = 17.0 Hz), 56.2 (d, *J* = 24.1 Hz), 54.2, 51.1 (d, *J* = 6.9 Hz), 42.1, 32.4, 27.4. ^{19}F NMR (376 MHz, Chloroform-*d*) δ -111.4.
[cis]: ^{1}H NMR (400 MHz, Chloroform-*d*) δ 7.66 (d, *J* = 7.2 Hz, 2H), 7.48 – 7.41 (m, 3H), 7.36 – 7.32 (m, 1H), 7.31 – 7.25 (m, 2H), 7.20 (d, *J* = 7.8 Hz, 2H), 5.67 – 5.61 (m, 1H), 5.54 – 5.48 (m, 1H), 5.26 (d, *J* = 15.7 Hz, 1H), 4.65 (s, 1H), 3.42 (t, *J* = 6.7 Hz, 2H), 1.97 – 1.90 (m, 2H), 1.39 (s, 9H). ^{13}C NMR (101 MHz, Chloroform-*d*) δ 173.8 (d, *J* = 2.0 Hz), 171.4, 159.2 (d, *J* = 259.8 Hz), 138.9, 136.3, 130.3, 128.9, 128.5, 128.4, 128.2 (d, *J* = 2.2 Hz), 127.8, 127.7, 126.4 (d, *J* = 7.7 Hz), 104.9 (d, *J* = 17.2 Hz), 56.2 (d, *J* = 24.4 Hz), 54.3, 50.8 (d, *J* = 6.9 Hz), 41.9, 33.2, 27.5. ^{19}F NMR (376 MHz, Chloroform-*d*) δ -111.7.
HRMS (APCI): calcd for $C_{26}H_{28}FN_2O^+$ $[M + H]^+$: 403.2180; found: 403.2184.

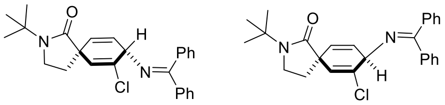

2-5-trans **2-5**-cis

2-(tert-butyl)-7-chloro-8-((diphenylmethylene)amino)-2-azaspiro[4.5]deca-6,9-dien-1-one (**2-5**)
2-5-trans was obtained (107 mg, 32% yield) as a brown oil.
2-5-cis was obtained (77 mg, 23% yield) as a brown oil.
d.r. (cis:trans) = 1:1.4
NMR:
[trans]: ^{1}H NMR (400 MHz, Chloroform-*d*) δ 7.65 (d, *J* = 7.2 Hz, 2H), 7.47 – 7.41 (m, 3H), 7.37 (d, *J* = 7.1 Hz, 1H), 7.31 (t, *J* = 7.4 Hz, 2H), 7.23 (d, *J* = 7.8 Hz, 2H), 5.83 (s, 1H), 5.67 (dd, *J* = 9.9, 2.0 Hz, 1H), 5.60 (dd, *J* = 9.8, 3.2 Hz, 1H), 4.73 (s, 1H), 3.47 (t, *J* = 6.8 Hz, 2H), 2.19 – 2.10 (m, 2H), 1.38 (s, 9H). ^{13}C NMR (101 MHz, Chloroform-*d*) δ 173.6, 171.2, 139.4, 136.3, 134.5, 130.3, 128.8, 128.6, 128.6, 128.0, 127.7, 127.6, 127.3, 125.0, 59.8, 54.4, 52.2, 42.2, 32.2, 27.5.
[cis]: ^{1}H NMR (400 MHz, Chloroform-*d*) δ 7.71 (d, *J* = 7.2 Hz, 2H), 7.53 – 7.42 (m, 3H), 7.39 – 7.36 (m, 1H), 7.35 – 7.30 (m, 2H), 7.27 (d, *J* = 7.2 Hz, 2H), 5.86 (s, 1H), 5.70 (d, *J* = 9.9 Hz, 1H), 5.58 (dd, *J* = 9.8, 3.6 Hz, 1H), 4.56 (s, 1H), 3.47 (t, *J* = 6.8 Hz, 2H), 1.98 (t, *J* = 6.7 Hz, 2H), 1.43 (s, 9H). ^{13}C NMR (101 MHz, Chloroform-*d*) δ 173.1, 170.7, 139.0, 136.5, 134.4, 130.3, 129.0, 128.5, 128.4, 127.9, 127.7, 127.7, 127.1, 125.4, 60.1, 54.5, 51.9, 41.9, 33.1, 27.6.

HRMS (APCI): calcd for $C_{26}H_{28}ClN_2O^+$ $[M + H]^+$: 419.1885; found: 419.1880.

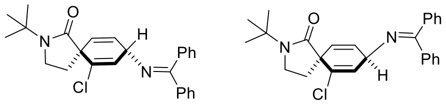

2-6-trans **2-6**-cis

2-(tert-butyl)-8-((diphenylmethylene)amino)-7-fluoro-2-azaspiro[4.5]deca-6,9-dien-1-one (**2-6**)
2-6-trans was obtained (116 mg, 36% yield) as a brown oil.
2-6-cis was obtained (74 mg, 23% yield) as a brown oil.
d.r. (cis:trans) = 1:1.4
NMR:
[trans]: ^{1}H NMR (400 MHz, Chloroform-*d*) δ 7.61 (d, *J* = 7.2 Hz, 2H), 7.43 – 7.39 (m, 3H), 7.36 – 7.34 (m, 1H), 7.31 – 7.28 (m, 2H), 7.14 (d, *J* = 7.5 Hz, 2H), 5.85 (d, *J* = 3.7 Hz, 1H), 5.66 (s, 2H), 4.81 (d, *J* = 3.1 Hz, 1H), 3.61 – 3.55 (m, 1H), 3.51 – 3.45 (m, 1H), 2.61 – 2.53 (m, 1H), 2.10 – 2.03 (m, 1H), 1.38 (s, 9H). ^{13}C NMR (101 MHz, Chloroform-*d*) δ 172.2, 170.0, 139.2, 136.2, 133.9, 130.2, 128.7, 128.6, 128.5, 128.3, 127.9, 127.2, 127.2, 127.0, 57.9, 54.5, 53.9, 43.1, 29.7, 27.2.
[cis]: ^{1}H NMR (400 MHz, Chloroform-*d*) δ 7.6 (d, *J* = 7.2 Hz, 2H), 7.5 – 7.4 (m, 3H), 7.4 – 7.3 (m, 1H), 7.3 – 7.3 (m, 2H), 7.2 (d, *J* = 7.9 Hz, 2H), 5.8 – 5.8 (m, 1H), 5.7 – 5.7 (m, 1H), 5.7 – 5.6 (m, 1H), 4.6 (t, *J* = 3.5 Hz, 1H), 3.6 – 3.5 (m, 1H), 3.5 – 3.4 (m, 1H), 2.4 – 2.3 (m, 1H), 1.9 – 1.8 (m, 1H), 1.4 (s, 9H). ^{13}C NMR (101 MHz, Chloroform-*d*) δ 171.9, 169.6, 138.9, 136.4, 134.2, 130.3, 128.8, 128.7, 128.5, 128.5, 127.9, 127.3, 127.1, 126.6, 57.9, 54.7, 53.6, 42.9, 30.5, 27.3.
HRMS (APCI): calcd for $C_{26}H_{28}ClN_2O^+$ $[M + H]^+$: 419.1885; found: 419.1880.

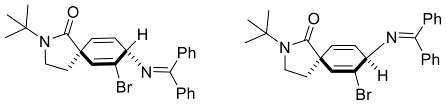

2-7-trans **2-7**-cis

2-(tert-butyl)-8-((diphenylmethylene)amino)-7-fluoro-2-azaspiro[4.5]deca-6,9-dien-1-one (**2-7**)
2-7-trans was obtained (141 mg, 38% yield) as a brown oil.
2-7-cis was obtained (82 mg, 22% yield) as a brown oil.
d.r. (cis:trans) = 1:1.7
NMR:
[trans]: ^{1}H NMR (400 MHz, Chloroform-*d*) δ 7.65 (d, *J* = 7.0 Hz, 2H), 7.47 – 7.36 (m, 3H), 7.37 – 7.33 (m, 1H), 7.33 – 7.25 (m, 3H), 7.22 (d, *J* = 5.7 Hz, 2H), 6.07 (s, 1H), 5.71 – 5.65 (m, 1H), 5.59 (dd, *J* = 9.7, 3.3 Hz, 1H), 4.80 – 4.74 (m, 1H), 3.43 (t, *J* = 6.8 Hz, 2H), 2.16 – 2.04 (m, 2H), 1.35 (s, 9H). ^{13}C NMR (101 MHz, Chloroform-*d*) δ 173.0, 170.6, 139.2, 136.1, 130.1, 128.7, 128.6, 128.4, 127.8, 127.4, 127.3, 127.1, 125.8, 60.5, 54.2, 52.7, 41.9, 31.8, 27.3.
[cis]: ^{1}H NMR (400 MHz, Chloroform-*d*) δ 7.68 (d, *J* = 7.3 Hz, 2H), 7.47 – 7.41 (m, 3H), 7.35 – 7.32 (m, 1H), 7.29 (d, *J* = 7.9 Hz, 2H), 7.24 (d, *J* = 8.0 Hz, 2H), 6.07 (s, 1H), 5.71 – 5.67 (m, 1H), 5.53 (dd, *J* = 9.8, 3.6 Hz, 1H), 4.60 – 4.57 (m, 1H), 3.43 (t, *J* = 6.8 Hz, 2H), 1.94 (t, *J* = 6.5 Hz, 2H), 1.39 (s, 9H). ^{13}C NMR (101 MHz, Chloroform-*d*) δ 172.8, 170.6, 138.9, 136.3, 130.3, 129.3, 128.9, 128.4, 128.4, 127.8, 127.6, 127.5, 127.0, 125.6, 61.0, 54.4, 52.5, 41.8, 32.7, 27.5.
HRMS (APCI): calcd for $C_{26}H_{28}BrN_2O^+$ $[M + H]^+$: 463.1380; found: 463.1374.

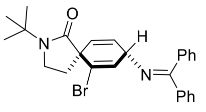 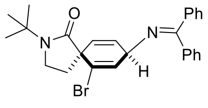

2-8-trans **2-8**-cis

6-bromo-2-(tert-butyl)-8-((diphenylmethylene)amino)-2-azaspiro[4.5]deca-6,9-dien-1-one (**2-4**)

2-4-trans was obtained (163 mg, 44% yield) as a brown oil.

2-4-cis was obtained (78 mg, 21% yield) as a brown oil.

d.r. (cis:trans) = 1:2.1

NMR:

[trans]: ^{1}H NMR (400 MHz, Chloroform-*d*) δ 7.62 (d, *J* = 7.0 Hz, 2H), 7.42 (d, *J* = 7.1 Hz, 3H), 7.30 (t, *J* = 7.4 Hz, 3H), 7.14 (dd, *J* = 7.5, 1.9 Hz, 2H), 6.11 (d, *J* = 3.6 Hz, 1H), 5.69 (s, 2H), 4.76 (dd, *J* = 3.6, 1.3 Hz, 1H), 3.63 – 3.57 (m, 1H), 3.50 – 3.44 (m, 1H), 2.61 – 2.53 (m, 1H), 2.10 – 2.03 (m, 1H), 1.39 (s, 9H). ^{13}C NMR (101 MHz, Chloroform-*d*) δ 172.3, 170.2, 139.2, 136.1, 131.4, 130.3, 128.7, 128.6, 128.2, 127.9, 127.2, 126.8, 125.7, 58.5, 54.6, 43.0, 30.6, 27.2.

[cis]: ^{1}H NMR (400 MHz, Chloroform-*d*) δ 7.66 (d, *J* = 7.2 Hz, 2H), 7.49 – 7.43 (m, 3H), 7.37 – 7.33 (m, 1H), 7.29 (d, *J* = 7.7 Hz, 2H), 7.17 (d, *J* = 6.1 Hz, 2H), 6.07 (d, *J* = 2.1 Hz, 1H), 5.71 (d, *J* = 10.1 Hz, 1H), 5.65 (d, *J* = 11.1 Hz, 1H), 4.52 (t, *J* = 3.7 Hz, 1H), 3.59 – 3.53 (m, 1H), 3.48 – 3.42 (m, 1H), 2.39 – 2.32 (m, 1H), 1.91 – 1.83 (m, 1H), 1.43 (s, 9H). ^{13}C NMR (101 MHz, Chloroform-*d*) δ 172.0, 169.3, 139.0, 136.5, 131.4, 130.3, 128.8, 128.7, 128.4, 128.4, 127.9, 127.3, 126.5, 125.7, 58.5, 54.6, 54.3, 42.8, 31.3, 27.4.

HRMS (APCI): calcd for $C_{26}H_{28}BrN_2O^+$ $[M + H]^+$: 463.1380; found: 463.1374.

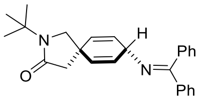 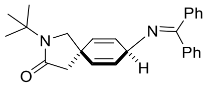

2-9-trans **2-9**-cis

2-(tert-butyl)-8-((diphenylmethylene)amino)-2-azaspiro[4.5]deca-6,9-dien-3-one (**2-9**)

2-9-trans was obtained (46 mg, 15% yield) as a brown oil.

2-9-cis was obtained (46 mg, 15% yield) as a brown oil.

d.r. (cis:trans) = 1:1

NMR:

[trans]: ^{1}H NMR (400 MHz, Chloroform-*d*) δ 7.62 (d, *J* = 7.2 Hz, 2H), 7.51 – 7.43 (m, 3H), 7.40 – 7.35 (m, 1H), 7.33 – 7.28 (m, 2H), 7.19 (d, *J* = 6.0 Hz, 2H), 5.86 (dd, *J* = 10.1, 1.8 Hz, 2H), 5.60 (dd, *J* = 10.0, 3.2 Hz, 2H), 4.49 – 4.41 (m, 1H), 3.43 (s, 2H), 2.28 (s, 2H), 1.40 (s, 9H). ^{13}C NMR (101 MHz, Chloroform-*d*) δ 173.0, 169.4, 139.5, 136.7, 131.3, 130.2, 128.7, 128.6, 128.5, 128.0, 127.5, 127.1, 57.3, 55.9, 54.0, 46.7, 36.6, 27.7.

[cis]: ^{1}H NMR (400 MHz, Chloroform-*d*) δ 7.61 (d, *J* = 7.6 Hz, 2H), 7.50 – 7.43 (m, 3H), 7.39 – 7.34 (m, 1H), 7.30 (t, *J* = 7.5 Hz, 2H), 7.20 (d, *J* = 7.0 Hz, 2H), 5.85 (d, *J* = 9.8 Hz, 2H), 5.61 (dd, *J* = 10.0, 3.2 Hz, 2H), 4.44 (s, 1H), 3.24 (s, 2H), 2.46 (s, 2H), 1.36 (s, 9H). ^{13}C NMR (101 MHz, Chloroform-*d*) δ 173.3, 169.3, 139.4, 136.7, 131.4, 130.2, 128.6, 128.4, 128.0, 127.5, 127.2, 57.5, 55.7, 53.9, 46.4, 36.6, 27.6.

HRMS (APCI): calcd for $C_{26}H_{29}N_2O^+$ $[M + H]^+$: 385.2274; found: 385.2270.

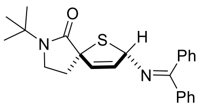

2-10-trans

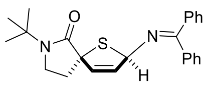

2-10-cis

7-(tert-butyl)-2-((diphenylmethylene)amino)-1-thia-7-azaspiro[4.4]non-3-en-6-one (**2-10**)

2-10-trans was obtained (91 mg, 29% yield) as a brown oil.

2-10-cis was obtained (82 mg, 26% yield) as a brown oil.

d.r. (cis:trans) = 1:1.1

NMR:

[trans]: ^{1}H NMR (400 MHz, Chloroform-*d*) δ 7.61 (d, *J* = 7.6 Hz, 2H), 7.44 – 7.33 (m, 4H), 7.29 (t, *J* = 7.6 Hz, 2H), 7.12 (d, *J* = 6.8 Hz, 2H), 6.05 (s, 1H), 5.89 (dd, *J* = 6.1, 2.5 Hz, 1H), 5.73 (d, *J* = 6.0 Hz, 1H), 3.45 – 3.32 (m, 2H), 2.54 – 2.40 (m, 2H), 1.36 (s, 9H). ^{13}C NMR (101 MHz, Chloroform-*d*) δ 173.6, 170.1, 139.1, 135.8, 134.3, 133.3, 130.4, 128.9, 128.7, 128.6, 127.9, 127.6, 75.1, 69.6, 54.4, 42.8, 35.3, 27.3.

[cis]: ^{1}H NMR (400 MHz, Chloroform-*d*) δ 7.63 (d, *J* = 7.1 Hz, 2H), 7.49 – 7.39 (m, 3H), 7.34 (t, *J* = 7.2 Hz, 1H), 7.30 – 7.24 (m, 2H), 7.14 (d, *J* = 6.5 Hz, 2H), 5.92 (s, 1H), 5.88 (dd, *J* = 6.1, 2.4 Hz, 1H), 5.73 (d, *J* = 5.6 Hz, 1H), 3.41 – 3.35 (m, 1H), 3.35 – 3.28 (m, 1H), 2.32 – 2.25 (m, 1H), 2.21 – 2.14 (m, 1H), 1.38 (s, 9H). ^{13}C NMR (101 MHz, Chloroform-*d*) δ 173.2, 170.3, 138.7, 136.1, 134.0, 133.2, 130.5, 129.0, 128.6, 128.5, 127.8, 127.7, 74.7, 69.2, 54.5, 42.7, 35.2, 27.4.

HRMS (APCI): calcd for $C_{24}H_{27}N_2OS^+$ [M + H]$^+$: 391.1839; found: 391.1833.

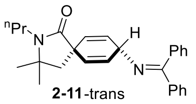

2-11-trans

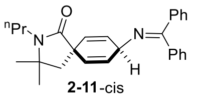

2-11-cis

8-((diphenylmethylene)amino)-3,3-dimethyl-2-propyl-2-azaspiro[4.5]deca-6,9-dien-1-one (**2-11**)

2-11-trans was obtained (118 mg, 38% yield) as a yellow solid.

2-11-cis was obtained (49 mg, 16% yield) as a yellow solid.

d.r. (cis:trans) = 1:2.4

NMR:

[trans]: ^{1}H NMR (400 MHz, Chloroform-*d*) δ 7.64 – 7.58 (m, 2H), 7.44 – 7.37 (m, 3H), 7.37 – 7.27 (m, 3H), 7.18 – 7.12 (m, 2H), 5.73 (dd, *J* = 10.2, 2.6 Hz, 2H), 5.68 (dd, *J* = 10.1, 1.7 Hz, 2H), 4.66 (p, *J* = 2.1 Hz, 1H), 3.10 – 3.03 (m, 2H), 2.11 (s, 2H), 1.62 – 1.51 (m, 2H), 1.30 (s, 6H), 0.86 (t, *J* = 7.4 Hz, 3H). ^{13}C NMR (101 MHz, Chloroform-*d*) δ 173.9, 169.3, 139.5, 136.4, 130.0, 129.4, 128.6, 128.5, 128.5, 128.4, 127.8, 127.4, 58.0, 54.9, 48.4, 47.3, 41.9, 29.2, 22.4, 11.5.

[cis]: ^{1}H NMR (400 MHz, Chloroform-*d*) δ 7.67 – 7.61 (m, 2H), 7.49 – 7.42 (m, 3H), 7.36 – 7.27 (m, 3H), 7.21 – 7.17 (m, 2H), 5.75 – 5.67 (m, 4H), 4.43 (q, *J* = 1.8 Hz, 1H), 3.14 – 3.10 (m, 2H), 1.92 (s, 2H), 1.66 – 1.59 (m, 2H), 1.29 (s, 6H), 0.91 (t, *J* = 7.4 Hz, 3H). ^{13}C NMR (101 MHz, Chloroform-*d*) δ 174.0, 169.4, 139.1, 136.8, 130.3, 129.7, 128.8, 128.6, 128.4, 128.0, 128.0, 127.5, 58.1, 55.1, 49.2, 46.9, 42.0, 29.3, 22.5, 11.6.

HRMS (APCI): calcd for $C_{27}H_{31}N_2O^+$ [M + H]$^+$: 399.2431; found: 399.2426.

V.2 Rearomatization

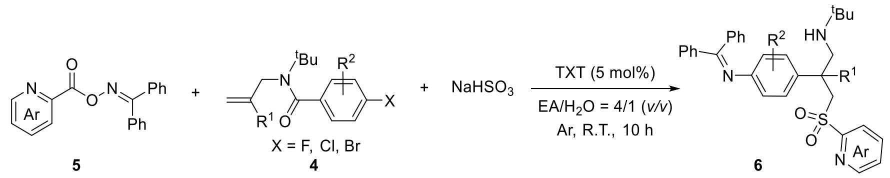

Standard Conditions: N-allyl-N-(tert-butyl)benzamide **4** (0.2 mmol), O-oxime ester **5** (0.34 mmol, 1.7 equiv), $NaHSO_3$ (0.6 mmol, 3.0 equiv.), TXT (5 mol%) in 5 mL of EA/H_2O (*v*/*v* = 4/1) under Ar atmosphere at R.T. and irradiated with visible LEDs (2 x 15 W, 405 nm) for 10h.
Workup: The reaction mixture was washed with brine (saturated NaCl(aq)) and extracted with EA multiple times. The combined organic layers were then dried over Na_2SO_4, concentrated under reduced pressure, and purified by column chromatography to isolate all isomers of target product **6**. Isolated yields were reported as the average of two parallel experiments.

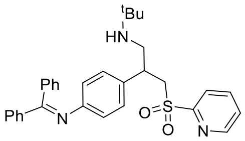

N-(tert-butyl)-2-(4-((diphenylmethylene)amino)phenyl)-3-(pyridin-2-ylsulfonyl)propan-1-amine (**6-1**)
The title compound was obtained as a beige solid (131 mg, 64% yield).
NMR: ^{1}H NMR (400 MHz, Chloroform-*d*) δ 8.57 (d, *J* = 4.7 Hz, 1H), 7.78 (dd, *J* = 6.8, 1.7 Hz, 2H), 7.71 (d, *J* = 7.1 Hz, 2H), 7.47 – 7.41 (m, 2H), 7.37 (t, *J* = 7.6 Hz, 2H), 7.27 – 7.18 (m, 3H), 7.04 (dd, *J* = 7.4, 2.0 Hz, 2H), 6.79 (d, *J* = 8.0 Hz, 2H), 6.49 (d, *J* = 8.0 Hz, 2H), 3.79 (d, *J* = 6.7 Hz, 2H), 3.19 (p, *J* = 6.8 Hz, 1H), 2.80 – 2.67 (m, 2H), 0.92 (s, 9H). ^{13}C NMR (101 MHz, Chloroform-*d*) δ 168.3, 157.1, 150.1, 149.8, 139.2, 137.7, 135.9, 134.4, 130.6, 129.2, 129.1, 128.4, 128.0, 127.7, 127.7, 126.8, 122.0, 120.9, 55.4, 49.8, 47.3, 40.6, 28.7. HRMS (APCI): calcd for $C_{19}H_{14}ClN_2O_2^+$ [M + H]$^+$: 337.0738; found: 337.0733.
HRMS (APCI): calcd for $C_{31}H_{34}N_3O_2S^+$ [M + H]$^+$: 512.2366; found: 512.2361.

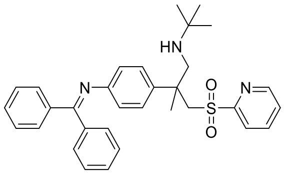

N-(tert-butyl)-2-(4-((diphenylmethylene)amino)phenyl)-2-methyl-3-(pyridin-2-ylsulfonyl)-propan-1-amine (**6-2**)
The title compound was obtained as a brown oil (126 mg, 60% yield).
NMR: ^{1}H NMR (400 MHz, Chloroform-*d*) δ 8.62 (d, *J* = 4.7 Hz, 1H), 7.80 – 7.74 (m, 1H), 7.71 (d, *J* = 7.3 Hz, 2H), 7.64 (d, *J* = 7.8 Hz, 1H), 7.48 – 7.45 (m, 1H), 7.44 – 7.36 (m, 3H), 7.22 (q, *J* = 6.7 Hz, 3H), 7.04 (d, *J* = 6.4 Hz, 2H), 6.92 (d, *J* = 8.2 Hz, 2H), 6.44 (d, *J* = 8.2 Hz, 2H), 4.00 (d, *J* = 14.9 Hz, 1H), 3.81 (d, *J* = 15.0 Hz, 1H), 2.83 – 2.74 (m, 2H), 1.57 (s, 3H), 0.94 (s, 9H). ^{13}C NMR (101 MHz, Chloroform-*d*) δ 168.3, 158.2, 149.7, 149.5, 139.4, 137.9, 137.2, 136.0, 130.7, 129.4, 129.2, 128.5, 128.1, 127.8, 126.6, 126.5, 121.7, 120.5, 60.2, 52.6, 50.2, 41.0, 28.8, 22.7.

HRMS (APCI): calcd for $C_{32}H_{36}N_3O_2S^+$ $[M + H]^+$: 526.2523; found: 526.2521.

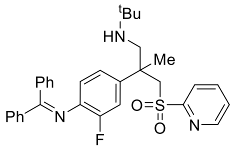

N-(tert-butyl)-2-(4-((diphenylmethylene)amino)-3-fluorophenyl)-2-methyl-3-(pyridin-2-ylsulfonyl)propan-1-amine (**6-3**)

The title compound was obtained as a brown oil (126 mg, 58% yield).

NMR: ^{1}H NMR (400 MHz, Chloroform-*d*) δ 8.63 (d, *J* = 4.0 Hz, 1H), 7.81 – 7.76 (m, 1H), 7.75 – 7.70 (m, 2H), 7.65 (d, *J* = 7.8 Hz, 1H), 7.49 – 7.43 (m, 2H), 7.39 (t, *J* = 7.4 Hz, 2H), 7.26 – 7.19 (m, 3H), 7.11 – 7.04 (m, 2H), 6.74 (dd, *J* = 8.3, 2.1 Hz, 1H), 6.66 (dd, *J* = 12.4, 2.0 Hz, 1H), 6.48 (t, *J* = 8.4 Hz, 1H), 3.98 (d, *J* = 14.9 Hz, 1H), 3.78 (d, *J* = 14.9 Hz, 1H), 2.77 – 2.70 (m, 2H), 1.54 (s, 3H), 0.93 (s, 9H). ^{13}C NMR (101 MHz, Chloroform-*d*) δ 171.2, 157.9, 151.4 (d, *J* = 244.4 Hz), 149.8, 139.4 (d, *J* = 5.9 Hz), 138.7, 137.8, 137.6 (d, *J* = 12.7 Hz), 136.1, 131.0, 129.3, 128.8, 128.5, 128.1, 127.8, 126.7, 121.9, 121.9, 121.6, 114.0 (d, *J* = 21.1 Hz), 59.9, 52.6, 50.2, 40.9, 28.7, 22.6. ^{19}F NMR (376 MHz, Chloroform-*d*) δ -124.8.

HRMS (APCI): calcd for $C_{32}H_{34}FN_3O_2S^+$ $[M + H]^+$: 543.2350; found: 543.2347.

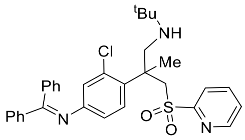

N-(tert-butyl)-2-(2-chloro-4-((diphenylmethylene)amino)phenyl)-2-methyl-3-(pyridin-2-ylsulfonyl)propan-1-amine (**6-4**)

The title compound was obtained as a brown oil (90 mg, 40% yield).

NMR: ^{1}H NMR (400 MHz, Chloroform-*d*) δ 8.64 (d, *J* = 3.3 Hz, 1H), 7.74 – 7.67 (m, 3H), 7.49 (dd, *J* = 18.4, 7.5 Hz, 2H), 7.44 – 7.37 (m, 3H), 7.29 – 7.25 (m, 3H), 7.14 (d, *J* = 8.5 Hz, 1H), 7.07 (dd, *J* = 7.6, 2.0 Hz, 2H), 6.50 (dd, *J* = 8.5, 2.3 Hz, 1H), 6.37 (d, *J* = 2.3 Hz, 1H), 4.50 (d, *J* = 15.1 Hz, 1H), 3.89 (d, *J* = 15.1 Hz, 1H), 3.07 (d, *J* = 11.7 Hz, 1H), 2.98 (d, *J* = 11.7 Hz, 1H), 1.68 (s, 3H), 0.96 (s, 9H). ^{13}C NMR (101 MHz, Chloroform-*d*) δ 157.6, 151.0, 149.9, 139.0, 137.6, 135.5, 132.8, 132.5, 131.0, 130.6, 129.3, 128.9, 128.2, 128.0, 126.6, 123.7, 121.8, 119.1, 57.6, 50.3, 49.7, 42.2, 29.0, 24.2.

HRMS (APCI): calcd for $C_{32}H_{35}ClN_3O_2S^+$ $[M + H]^+$: 560.2133; found: 560.2128.

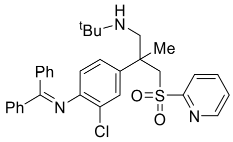

N-(tert-butyl)-2-(3-chloro-4-((diphenylmethylene)amino)phenyl)-2-methyl-3-(pyridin-2-ylsulfonyl)propan-1-amine (**6-5**)

The title compound was obtained as a brown oil (122 mg, 55% yield).

NMR: ^{1}H NMR (400 MHz, Chloroform-*d*) δ 8.62 (d, *J* = 4.7 Hz, 1H), 7.78 – 7.72 (m, 3H), 7.61 (d, *J* = 7.8 Hz, 1H), 7.50 – 7.36 (m, 5H), 7.22 (d, *J* = 7.0 Hz, 2H), 7.08 (d, *J* = 5.3 Hz, 2H), 6.97 (d, *J* = 2.1 Hz, 1H), 6.80 (dd, *J* = 8.3, 2.1 Hz, 1H), 6.29 (d, *J* = 8.3 Hz, 1H), 3.99 (d, *J* = 15.0 Hz, 1H), 3.76 (d, *J* = 14.9

Hz, 1H), 2.73 (s, 2H), 1.54 (s, 3H), 0.93 (s, 9H). ^{13}C NMR (101 MHz, Chloroform-*d*) δ 169.9, 157.7, 149.8, 147.1, 138.5, 138.5, 137.7, 135.9, 131.0, 129.4, 128.8, 128.5, 128.1, 127.7, 127.6, 126.8, 125.0, 124.2, 121.6, 120.6, 59.9, 52.5, 50.2, 40.8, 28.8, 22.5.

HRMS (APCI): calcd for $C_{32}H_{34}ClN_3O_2S^+$ [M + H]$^+$: 559.2055; found: 559.2051.

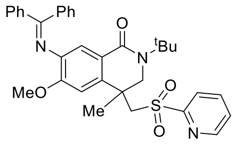

2-(tert-butyl)-7-((diphenylmethylene)amino)-6-methoxy-4-methyl-4-((pyridin-2-ylsulfonyl)-methyl)-3,4-dihydroisoquinolin-1(2H)-one (**6-6**)

The title compound was obtained as a brown oil (145 mg, 62% yield).

NMR: ^{1}H NMR (400 MHz, Chloroform-*d*) δ 8.60 (d, *J* = 4.7 Hz, 1H), 7.89 – 7.83 (m, 2H), 7.66 (d, *J* = 7.2 Hz, 2H), 7.47 – 7.44 (m, 1H), 7.40 (d, *J* = 7.0 Hz, 1H), 7.34 (d, *J* = 7.6 Hz, 2H), 7.21 – 7.14 (m, 4H), 7.11 – 7.04 (m, 2H), 6.57 (s, 1H), 4.07 (d, *J* = 13.0 Hz, 1H), 3.69 (s, 3H), 3.64 (d, *J* = 14.4 Hz, 1H), 3.58 (d, *J* = 14.4 Hz, 1H), 3.22 (d, *J* = 12.9 Hz, 1H), 1.67 (s, 3H), 1.53 (s, 9H), 1.50 (s, 9H). ^{13}C NMR (101 MHz, Chloroform-*d*) δ 170.1, 164.5, 157.7, 151.9, 150.0, 140.4, 139.1, 138.9, 138.0, 136.4, 130.6, 129.3, 128.5, 128.4, 127.9, 127.5, 127.1, 123.0, 120.8, 120.8, 105.5, 57.3, 55.7, 55.4, 51.3, 37.1, 28.5, 21.6.

HRMS (APCI): calcd for $C_{34}H_{36}N_3O_4S^+$ [M + H]$^+$: 582.2421; found: 582.2416.

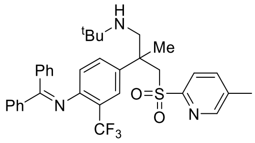

N-(tert-butyl)-2-(4-((diphenylmethylene)amino)-3-(trifluoromethyl)phenyl)-2-methyl-3-((5-methylpyridin-2-yl)sulfonyl)propan-1-amine (**6-7**)

The title compound was obtained as a brown oil (115 mg, 48% yield).

NMR: ^{1}H NMR (400 MHz, Chloroform-*d*) δ 8.39 (s, 1H), 7.75 (d, *J* = 7.7 Hz, 2H), 7.48 – 7.38 (m, 5H), 7.27 – 7.22 (m, 4H), 7.14 – 7.06 (m, 2H), 7.01 (d, *J* = 8.5 Hz, 1H), 6.20 (d, *J* = 8.4 Hz, 1H), 4.02 (d, *J* = 15.0 Hz, 1H), 3.74 (d, *J* = 15.0 Hz, 1H), 2.72 (s, 2H), 2.39 (s, 3H), 1.56 (s, 3H), 0.93 (s, 9H). ^{13}C NMR (101 MHz, Chloroform-*d*) δ 168.4, 154.9, 150.3, 147.5, 138.6, 137.8, 137.7, 137.2, 135.5, 131.0, 130.2, 129.3, 128.8, 128.1, 127.8, 124.1 (q, *J* = 5.4 Hz), 123.8 (d, *J* = 273.4 Hz), 121.5, 120.6, 120.0 (q, *J* = 29.7 Hz), 60.1, 52.7, 50.1, 40.8, 28.8, 22.5, 18.4. ^{19}F NMR (376 MHz, Chloroform-*d*) δ -61.3.

HRMS (APCI): calcd for $C_{34}H_{37}F_3N_3O_2S^+$ [M + H]$^+$: 608.2553; found: 608.2548.

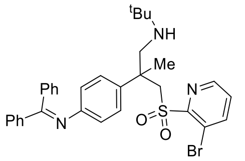

3-((3-bromopyridin-2-yl)sulfonyl)-*N*-(tert-butyl)-2-(4-((diphenylmethylene)amino)phenyl)-2-methylpropan-1-amine (**6-8**)

The title compound was obtained as a brown oil (72 mg, 30% yield).

NMR: ^{1}H NMR (400 MHz, Chloroform-*d*) δ 8.51 (d, *J* = 4.6 Hz, 1H), 7.94 (d, *J* = 8.1 Hz, 1H), 7.71 (d, *J* = 7.7 Hz, 2H), 7.46 (t, *J* = 7.1 Hz, 1H), 7.39 (t, *J* = 7.5 Hz, 2H), 7.31 – 7.22 (m, 4H), 7.06 (t, *J* = 6.7

Hz, 4H), 6.53 (d, *J* = 8.1 Hz, 2H), 4.09 (d, *J* = 14.9 Hz, 1H), 4.04 (d, *J* = 14.6 Hz, 1H), 2.85 (s, 2H), 1.61 (s, 3H), 0.96 (s, 9H). ^{13}C NMR (101 MHz, Chloroform-*d*) δ 168.4, 155.2, 149.5, 147.1, 143.9, 139.5, 137.7, 136.0, 130.7, 129.4, 129.2, 128.6, 128.1, 127.8, 127.4, 126.3, 120.7, 117.6, 60.0, 52.5, 50.4, 41.2, 28.8, 22.7.

HRMS (APCI): calcd for $C_{32}H_{35}BrN_3O_2S^+$ $[M + H]^+$: 604.1628; found: 604.1624.

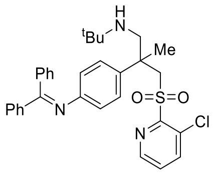

N-(tert-butyl)-3-((3-chloropyridin-2-yl)sulfonyl)-2-(4-((diphenylmethylene)amino)phenyl)-2-methylpropan-1-amine (**6-9**)

The title compound was obtained as a brown oil (67 mg, 30% yield).

NMR: ^{1}H NMR (400 MHz, Chloroform-*d*) δ 8.51 (d, *J* = 4.5 Hz, 1H), 7.74 (t, *J* = 8.1 Hz, 3H), 7.48 (t, *J* = 7.3 Hz, 1H), 7.44 – 7.39 (m, 3H), 7.27 – 7.22 (m, 3H), 7.11 – 7.02 (m, 4H), 6.53 (d, *J* = 8.1 Hz, 2H), 4.10 – 4.01 (m, 2H), 2.86 (s, 2H), 1.63 (s, 3H), 0.98 (s, 9H). ^{13}C NMR (101 MHz, Chloroform-*d*) δ 168.4, 153.8, 149.5, 146.7, 140.4, 139.5, 137.6, 136.0, 130.7, 130.2, 129.4, 129.2, 128.6, 128.1, 127.8, 127.6, 126.3, 120.7, 60.5, 52.5, 50.4, 41.2, 28.8, 22.7.

HRMS (APCI): calcd for $C_{32}H_{35}ClN_3O_2S^+$ $[M + H]^+$: 560.2133; found: 560.2128.

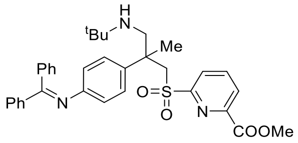

methyl 6-((3-(tert-butylamino)-2-(4-((diphenylmethylene)amino)phenyl)-2-methylpropyl)sulfonyl)picolinate (**6-10**)

The title compound was obtained as a brown oil (112 mg, 48% yield).

NMR: ^{1}H NMR (400 MHz, Chloroform-*d*) δ 8.18 (dd, *J* = 7.8, 1.1 Hz, 1H), 7.89 (t, *J* = 7.9 Hz, 1H), 7.73 (dd, *J* = 7.8, 1.1 Hz, 1H), 7.71 – 7.66 (m, 2H), 7.47 – 7.43 (m, 1H), 7.39 (t, *J* = 7.4 Hz, 2H), 7.23 – 7.17 (m, 3H), 7.02 (dd, *J* = 7.9, 1.7 Hz, 2H), 6.88 (d, *J* = 8.6 Hz, 2H), 6.35 (d, *J* = 8.5 Hz, 2H), 4.11 (d, *J* = 15.0 Hz, 1H), 3.99 (s, 3H), 3.86 (d, *J* = 15.1 Hz, 1H), 2.75 (s, 2H), 1.56 (s, 3H), 0.94 (s, 9H). ^{13}C NMR (101 MHz, Chloroform-*d*) δ 168.3, 164.3, 158.2, 149.5, 148.0, 139.4, 139.1, 136.8, 136.0, 130.7, 129.4, 129.2, 128.5, 128.1, 127.8, 127.4, 126.7, 124.3, 120.3, 60.1, 53.0, 52.6, 50.0, 40.9, 28.9, 22.7.

HRMS (APCI): calcd for $C_{34}H_{38}N_3O_4S^+$ $[M + H]^+$: 584.2578; found: 584.2573.

V.3 Carbo-aminoalkylation

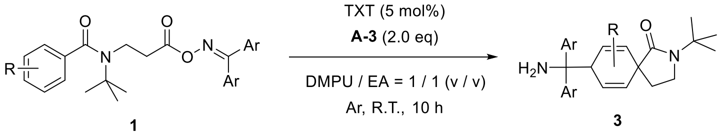

Standard Conditions: O-oxime ester **1** (0.2 mmol), **A-3** (0.4 mmol, 2.0 equiv), **TXT** (5 mol%) in 4 mL of DMPU/EA (0.05 M, *v*/*v* = 1/1) under Ar atmosphere at R.T. and irradiated with visible LEDs (2 x 15 W, 405 nm) for 10 h.

Workup: The reaction mixture was washed with brine (saturated NaCl(aq)) and extracted with EA multiple times. The combined organic layers were then dried over Na_2SO_4, concentrated under reduced pressure, and purified by column chromatography to isolate all isomers of target product **3**. Isolated yields were reported as the average of four experiments.

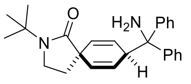

3-1-cis

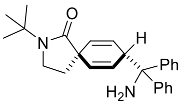

3-1-trans

8-(aminodiphenylmethyl)-2-(tert-butyl)-2-azaspiro[4.5]deca-6,9-dien-1-one (**3-1**)
3-1-cis was obtained (132 mg, 43% yield) as a brown oi, which can slowly solidify.
3-1-trans was obtained (78 mg, 25% yield) as a brown oil.
d.r. (cis:trans) = 1.7:1
NMR:
[cis]: ^{1}H NMR (400 MHz, Chloroform-*d*) δ 7.67 (d, *J* = 7.8 Hz, 4H), 7.27 (t, *J* = 7.5 Hz, 4H), 7.13 (t, *J* = 7.3 Hz, 2H), 5.64 (d, *J* = 10.1 Hz, 2H), 5.56 (dd, *J* = 10.3, 3.4 Hz, 2H), 4.24 (s, 1H), 3.43 (t, *J* = 6.7 Hz, 2H), 2.13 (s, 2H), 1.95 (t, *J* = 6.7 Hz, 2H), 1.38 (s, 9H). ^{13}C NMR (101 MHz, Chloroform-*d*) δ 174.9, 146.7, 130.5, 128.2, 126.9, 126.2, 125.9, 62.3, 54.2, 49.4, 44.7, 42.0, 33.7, 27.6.
[trans]: ^{1}H NMR (400 MHz, Chloroform-*d*) δ 7.59 (d, *J* = 6.9 Hz, 4H), 7.28 (t, *J* = 7.6 Hz, 4H), 7.15 (t, *J* = 7.3 Hz, 2H), 5.73 – 5.66 (m, 4H), 4.33 (s, 1H), 3.42 (t, *J* = 6.8 Hz, 2H), 1.92 (t, *J* = 6.8 Hz, 2H), 1.80 (s, 2H), 1.38 (s, 9H). ^{13}C NMR (101 MHz, Chloroform-*d*) δ 175.1, 146.3, 130.4, 128.3, 127.5, 126.3, 126.2, 62.8, 54.3, 49.4, 44.1, 42.2, 33.5, 27.6.
HRMS (APCI): calcd for $C_{26}H_{31}N_2O^+$ [M + H]$^+$: 387.2431; found: 387.2430.

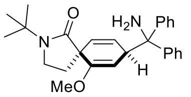

3-2-cis

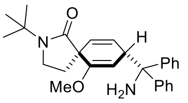

3-2-trans

8-(aminodiphenylmethyl)-2-(tert-butyl)-6-methoxy-2-azaspiro[4.5]deca-6,9-dien-1-one (**3-2**)
3-2-cis was obtained (150 mg, 45% yield) as a brown solid.
3-2-trans was obtained (73 mg, 22% yield) as a brown oil.
d.r. (cis:trans) = 2.1:1
NMR:
[cis]: ^{1}H NMR (400 MHz, Chloroform-*d*) δ 7.66 (dd, *J* = 12.9, 7.8 Hz, 4H), 7.26 (t, *J* = 15.0 Hz, 4H), 7.12 (t, *J* = 7.5 Hz, 2H), 5.57 – 5.48 (m, 2H), 4.36 (s, 2H), 3.49 – 3.35 (m, 2H), 3.30 (s, 3H), 2.42 – 2.29 (m, 1H), 1.87 – 1.72 (m, 1H), 1.37 (s, 9H). ^{13}C NMR (101 MHz, Chloroform-*d*) δ 174.4, 157.7, 147.1, 146.9, 130.9, 128.2, 128.1, 126.4, 126.2, 125.9, 125.8, 93.5, 62.6, 54.4, 54.2, 51.8, 45.8, 43.3, 30.7, 27.5.
[trans]: ^{1}H NMR (400 MHz, Chloroform-*d*) δ 7.59 (t, *J* = 8.5 Hz, 4H), 7.29 (t, *J* = 7.6 Hz, 4H), 7.15 (t, *J* = 7.4 Hz, 2H), 5.68 – 5.61 (m, 1H), 5.61 – 5.54 (m, 1H), 4.53 – 4.44 (m, 2H), 3.51 – 3.40 (m, 1H), 3.41 – 3.36 (m, 1H), 3.35 (s, 3H), 2.27 – 2.17 (m, 1H), 1.84 – 1.77 (m, 1H), 1.37 (s, 9H). ^{13}C NMR (101 MHz, Chloroform-*d*) δ 174.3, 157.8, 146.6, 146.3, 131.0, 128.2, 128.2, 126.9, 126.2, 126.1, 126.1, 93.5, 63.0, 54.4, 54.2, 51.6, 45.5, 43.4, 31.1, 27.4.
HRMS (APCI): calcd for $C_{27}H_{33}N_2O_2{}^+$ [M + H]$^+$: 417.2537; found: 417.2539.

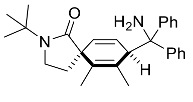
3-3-cis

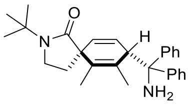
3-3-trans

8-(aminodiphenylmethyl)-2-(tert-butyl)-6,7-dimethyl-2-azaspiro[4.5]deca-6,9-dien-1-one (**3-3**)
3-3-cis was obtained (63 mg, 19% yield) as a beige solid.
3-3-trans was obtained (93 mg, 28% yield) as an off-white solid.
d.r. (cis:trans) = 1:1.5
NMR:
[cis]: ^{1}H NMR (400 MHz, Chloroform-*d*) δ 7.70 (d, *J* = 7.2 Hz, 2H), 7.54 (d, *J* = 7.2 Hz, 2H), 7.30 (t, *J* = 7.7 Hz, 2H), 7.22 (t, *J* = 7.7 Hz, 2H), 7.16 (t, *J* = 7.3 Hz, 2H), 7.11 (t, *J* = 7.3 Hz, 1H), 5.91 (dd, *J* = 10.1, 4.3 Hz, 1H), 5.67 (d, *J* = 10.0 Hz, 1H), 4.19 (d, *J* = 4.3 Hz, 1H), 3.47 – 3.41 (m, 2H), 2.20 – 2.11 (m, 1H), 1.83 (s, 2H), 1.79 – 1.75 (m, 1H), 1.58 (s, 3H), 1.39 (s, 9H), 0.98 (s, 3H). ^{13}C NMR (101 MHz, Chloroform-*d*) δ 175.3, 146.9, 146.7, 132.3, 130.8, 128.3, 128.2, 127.8, 126.6, 126.4, 126.2, 126.0, 63.4, 54.3, 53.4, 52.0, 42.5, 31.5, 27.4, 20.4, 15.5.
[trans]: ^{1}H NMR (400 MHz, Chloroform-*d*) δ 7.78 (d, *J* = 7.9 Hz, 2H), 7.69 (d, *J* = 7.8 Hz, 2H), 7.27 (t, *J* = 7.8 Hz, 2H), 7.20 (t, *J* = 7.6 Hz, 2H), 7.16 – 7.04 (m, 2H), 5.69 (dd, *J* = 10.1, 4.1 Hz, 1H), 5.58 (d, *J* = 10.1 Hz, 1H), 4.22 (d, *J* = 4.2 Hz, 1H), 3.49 – 3.39 (m, 2H), 2.32 – 2.19 (m, 3H), 1.76 – 1.68 (m, 1H), 1.56 (s, 3H), 1.39 (s, 9H), 0.92 (s, 3H). ^{13}C NMR (101 MHz, Chloroform-*d*) δ 175.4, 147.9, 147.8, 131.2, 129.5, 129.1, 128.1, 127.7, 126.8, 126.8, 126.7, 125.8, 125.7, 62.8, 54.3, 53.8, 51.5, 42.4, 31.0, 27.6, 20.4, 16.2.
HRMS (APCI): calcd for $C_{28}H_{35}N_2O^+$ $[M + H]^+$: 415.2744; found: 415.2742.

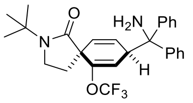
3-4-cis

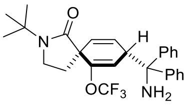
3-4-trans

8-(aminodiphenylmethyl)-2-(tert-butyl)-6-(trifluoromethoxy)-2-azaspiro[4.5]deca-6,9-dien-1-one (**3-4**)
3-4-cis was obtained (75 mg, 20% yield) as a brown oil.
3-4-trans was obtained (94 mg, 25% yield) as a brown oil.
d.r. (cis:trans) = 1:1.2
NMR:
[cis]: ^{1}H NMR (400 MHz, Chloroform-*d*) δ 7.67 (d, *J* = 7.4 Hz, 2H), 7.59 (d, *J* = 7.2 Hz, 2H), 7.31 – 7.24 (m, 4H), 7.17 – 7.12 (m, 2H), 5.55 (s, 2H), 5.24 (s, 1H), 4.42 (dd, *J* = 4.0, 1.9 Hz, 1H), 3.49 – 3.40 (m, 2H), 2.38 – 2.30 (m, 1H), 2.12 (s, 2H), 1.96 – 1.88 (m, 1H), 1.39 (s, 9H). ^{13}C NMR (101 MHz, Chloroform-*d*) δ 172.1, 148.7, 146.2 (d, *J* = 14.1 Hz), 130.8, 128.3 (d, *J* = 2.6 Hz), 126.2, 126.2, 125.9, 125.9, 110.2, 62.6, 54.8, 51.4, 46.5, 43.0, 30.1, 27.3. ^{19}F NMR (376 MHz, Chloroform-*d*) δ -56.4.
[trans]: ^{1}H NMR (400 MHz, Chloroform-*d*) δ 7.55 (d, *J* = 7.1 Hz, 2H), 7.49 (d, *J* = 7.4 Hz, 2H), 7.32 – 7.25 (m, 4H), 7.20 – 7.14 (m, 2H), 5.76 – 5.70 (m, 1H), 5.57 (dd, *J* = 10.1, 1.8 Hz, 1H), 5.50 – 5.44 (m, 1H), 4.50 – 4.44 (m, 1H), 3.44 – 3.36 (m, 2H), 2.16 – 2.07 (m, 1H), 1.85 – 1.77 (m, 1H), 1.71 (s, 2H), 1.37 (s, 9H). ^{13}C NMR (101 MHz, Chloroform-*d*) δ 172.0, 148.4, 145.7, 145.5, 130.4, 128.2 (d, *J* = 11.0

Hz), 126.5 (d, J = 1.3 Hz), 126.2, 126.1 (d, J = 38.1 Hz), 110.4 (d, J = 2.1 Hz), 63.3, 54.7, 51.2, 46.0, 42.9, 30.1, 27.2. ^{19}F NMR (376 MHz, Chloroform-*d*) δ -56.3.

HRMS (APCI): calcd for $C_{27}H_{30}F_3N_2O_2^+$ [M + H]$^+$: 471.2254; found: 471.2259.

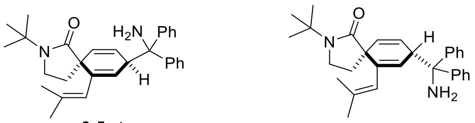

3-5-cis **3-5**-trans

8-(aminodiphenylmethyl)-2-(tert-butyl)-6-(2-methylprop-1-en-1-yl)-2-azaspiro[4.5]deca-6,9-dien-1-one (**3-5**)

3-5-cis was obtained (134 mg, 38% yield) as a beige solid.

3-5-trans was obtained (99 mg, 28% yield) as a yellow solid.

d.r. (cis:trans) = 1.4:1

NMR:

[cis]: ^{1}H NMR (400 MHz, Chloroform-*d*) δ 7.72 – 7.67 (m, 4H), 7.31 – 7.26 (m, 3H), 7.25 (d, J = 1.6 Hz, 1H), 7.15 – 7.10 (m, 2H), 5.64 (dd, J = 10.2, 1.6 Hz, 1H), 5.58 – 5.52 (m, 1H), 5.51 – 5.47 (m, 1H), 5.27 – 5.22 (m, 1H), 4.38 – 4.32 (m, 1H), 3.46 – 3.39 (m, 1H), 3.37 – 3.30 (m, 1H), 2.21 (s, 2H), 2.17 – 2.11 (m, 1H), 1.88 – 1.81 (m, 1H), 1.68 (s, 3H), 1.56 (s, 3H), 1.38 (s, 9H). ^{13}C NMR (101 MHz, Chloroform-*d*) δ 174.8, 147.0, 146.9, 138.9, 136.3, 132.3, 128.1, 128.1, 126.1, 126.1, 125.8, 125.8, 124.9, 122.9, 62.4, 54.2, 51.6, 45.2, 43.2, 31.4, 27.4, 25.9, 19.3.

[trans]: ^{1}H NMR (400 MHz, Chloroform-*d*) δ 7.61 (dd, J = 13.5, 7.8 Hz, 4H), 7.27 (q, J = 7.6 Hz, 4H), 7.14 (q, J = 7.1 Hz, 2H), 5.69 (s, 2H), 5.45 (s, 1H), 5.33 (s, 1H), 4.43 (t, J = 2.7 Hz, 1H), 3.43 – 3.37 (m, 1H), 3.37 – 3.30 (m, 1H), 2.08 – 1.99 (m, 1H), 1.86 – 1.77 (m, 3H), 1.67 (s, 3H), 1.50 (s, 3H), 1.38 (s, 9H). ^{13}C NMR (101 MHz, Chloroform-*d*) δ 174.7, 146.5, 146.2, 138.6, 136.3, 131.8, 128.2, 128.1, 126.1, 126.1, 126.0, 125.9, 125.3, 123.1, 62.8, 54.2, 51.5, 45.2, 42.9, 31.5, 27.4, 26.0, 19.5.

HRMS (APCI): calcd for $C_{30}H_{37}N_2O^+$ [M + H]$^+$: 441.2900; found: 441.2896.

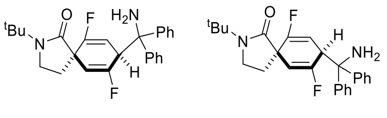

3-6-cis **3-6**-trans

8-(aminodiphenylmethyl)-2-(tert-butyl)-6,9-difluoro-2-azaspiro[4.5]deca-6,9-dien-1-one (**3-6**)

3-6-cis was obtained (110 mg, 32.5% yield) as a white solid.

3-6-trans was obtained (110 mg, 32.5% yield) as a white solid.

d.r. (cis:trans) = 1:1

NMR:

[cis]: ^{1}H NMR (400 MHz, Chloroform-*d*) δ 7.74 (d, J = 7.8 Hz, 2H), 7.68 (d, J = 7.8 Hz, 2H), 7.35 – 7.25 (m, 4H), 7.21 – 7.12 (m, 2H), 5.22 (dd, J = 15.9, 7.1 Hz, 1H), 5.06 – 4.94 (m, 1H), 4.69 (q, J = 3.8 Hz, 1H), 3.55 – 3.38 (m, 2H), 2.49 – 2.38 (m, 1H), 2.28 (s, 2H), 2.00 – 1.88 (m, 1H), 1.43 (s, 9H). ^{13}C NMR (101 MHz, Chloroform-*d*) δ 171.6, 146.4, 146.3, 128.4, 127.7, 126.2, 126.1, 126.0, 125.9, 125.9, 108.1 (dd, J = 22.1, 8.0 Hz), 102.7 (dd, J = 21.2, 9.5 Hz), 62.2, 54.9, 51.0 (dd, J = 28.0, 8.5 Hz), 46.6 (dd, J = 21.8, 7.4 Hz), 42.6, 29.7, 27.4. ^{19}F NMR (376 MHz, Chloroform-*d*) δ -102.3 (d, J = 7.1 Hz), -117.7 (d, J = 6.8 Hz).

[trans]: ^{1}H NMR (400 MHz, Chloroform-*d*) δ 7.48 (t, *J* = 6.7 Hz, 4H), 7.28 (q, *J* = 7.7 Hz, 4H), 7.20 (t, *J* = 7.1 Hz, 2H), 5.29 – 5.20 (m, 1H), 5.10 (dd, *J* = 16.6, 7.1 Hz, 1H), 4.58 (s, 1H), 3.41 – 3.24 (m, 2H), 2.07 – 2.00 (m, 1H), 1.96 (s, 2H), 1.65 – 1.55 (m, 1H), 1.36 (s, 9H). ^{13}C NMR (101 MHz, Chloroform-*d*) δ 171.4, 145.6, 144.8, 127.9, 127.8, 126.7, 126.6, 126.5, 107.5 (dd, *J* = 22.4, 8.4 Hz), 103.4 (dd, *J* = 21.5, 9.3 Hz), 64.0, 54.7, 50.7 (dd, *J* = 27.9, 8.5 Hz), 46.7 (dd, *J* = 21.6, 7.1 Hz), 42.4, 29.4, 27.3. ^{19}F NMR (376 MHz, Chloroform-*d*) δ -103.0 (d, *J* = 7.3 Hz), -117.7 (d, *J* = 7.2 Hz).
HRMS (APCI): calcd for $C_{26}H_{29}F_2N_2O^+$ [M + H]$^+$: 423.2242; found: 423.2245.

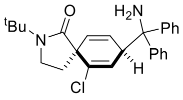

3-7-cis

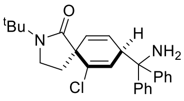

3-7-trans

8-(aminodiphenylmethyl)-2-(tert-butyl)-6-chloro-2-azaspiro[4.5]deca-6,9-dien-3-one (**3-7**)
3-7-cis was obtained (123 mg, 37% yield) as a white solid.
3-7-trans was obtained (112 mg, 33% yield) as a white solid.
d.r. (cis:trans) = 1.1:1
NMR:
[cis]: ^{1}H NMR (400 MHz, Chloroform-*d*) δ 7.68 (dd, *J* = 7.7, 4.1 Hz, 4H), 7.31 (q, *J* = 7.9 Hz, 4H), 7.17 (q, *J* = 7.5 Hz, 2H), 5.74 (dd, *J* = 3.7, 1.7 Hz, 1H), 5.65 (d, *J* = 10.1 Hz, 1H), 5.54 – 5.47 (m, 1H), 4.42 (t, *J* = 3.9 Hz, 1H), 3.61 – 3.54 (m, 1H), 3.52 – 3.44 (m, 1H), 2.54 – 2.46 (m, 1H), 2.05 (s, 2H), 1.98 – 1.90 (m, 1H), 1.44 (s, 9H). ^{13}C NMR (101 MHz, Chloroform-*d*) δ 172.4, 146.4, 146.2, 135.4, 131.2, 128.4, 128.3, 126.2, 126.1, 126.0, 125.9, 125.2, 62.5, 54.7, 54.2, 47.5, 43.0, 30.8, 27.4.
[trans]: ^{1}H NMR (400 MHz, Chloroform-*d*) δ 7.57 (dd, *J* = 7.2, 3.4 Hz, 4H), 7.31 (q, *J* = 7.7 Hz, 4H), 7.20 (q, *J* = 7.1 Hz, 2H), 5.89 (d, *J* = 3.4 Hz, 1H), 5.67 (s, 2H), 4.50 (d, *J* = 3.5 Hz, 1H), 3.59 – 3.52 (m, 1H), 3.49 – 3.42 (m, 1H), 2.39 – 2.31 (m, 1H), 1.89 – 1.81 (m, 1H), 1.68 (s, 2H), 1.42 (s, 9H). ^{13}C NMR (101 MHz, Chloroform-*d*) δ 172.4, 145.8, 145.6, 135.3, 131.0, 128.4, 128.3, 126.5, 126.4, 126.1, 126.0, 125.7, 63.1, 54.7, 54.1, 47.2, 43.0, 30.7, 27.3.
HRMS (APCI): calcd for $C_{26}H_{30}ClN_2O^+$ [M + H]$^+$: 421.2041; found: 421.2044.

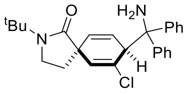

3-8-cis

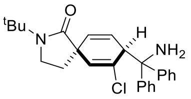

3-8-trans

8-(aminodiphenylmethyl)-2-(tert-butyl)-7-chloro-2-azaspiro[4.5]deca-6,9-dien-3-one (**3-8**)
3-8-cis was obtained (116.5 mg, 34.5% yield) as a white solid.
3-8-trans was obtained (116.5 mg, 34.5% yield) as a white solid.
d.r. (cis:trans) = 1:1
NMR:
[cis]: ^{1}H NMR (400 MHz, Chloroform-*d*) δ 7.76 (d, *J* = 7.2 Hz, 2H), 7.72 (d, *J* = 7.3 Hz, 2H), 7.27 (t, *J* = 7.6 Hz, 2H), 7.23 (t, *J* = 8.0 Hz, 2H), 7.16 – 7.07 (m, 2H), 5.83 (d, *J* = 1.8 Hz, 1H), 5.57 (dd, *J* = 10.0, 3.9 Hz, 1H), 5.52 (dd, *J* = 10.3, 1.8 Hz, 1H), 4.54 (d, *J* = 3.9 Hz, 1H), 3.43 (t, *J* = 6.7 Hz, 2H), 2.27 (s, 2H), 1.98 (t, *J* = 6.7 Hz, 2H), 1.39 (s, 9H). ^{13}C NMR (101 MHz, Chloroform-*d*) δ 173.5, 146.8, 146.6, 133.0, 129.9, 128.7, 128.2, 127.6, 126.6, 126.4, 126.0, 126.0, 62.7, 54.6, 52.4, 51.3, 41.9, 33.7, 27.6.

[trans]: ^{1}H NMR (400 MHz, Chloroform-*d*) δ 7.70 – 7.46 (m, 4H), 7.36 – 7.24 (m, 4H), 7.23 – 7.17 (m, 2H), 5.86 (s, 2H), 5.62 (d, *J* = 10.1 Hz, 1H), 4.47 (s, 1H), 3.38 (t, *J* = 7.0 Hz, 2H), 1.97 (s, 2H), 1.78 (s, 2H), 1.39 (s, 9H). ^{13}C NMR (101 MHz, Chloroform-*d*) δ 174.0, 145.9, 145.5, 133.0, 129.8, 128.9, 128.5, 127.9, 127.8, 126.7, 126.4, 64.3, 54.3, 51.8, 51.1, 42.0, 32.6, 27.5.

HRMS (APCI): calcd for $C_{26}H_{30}ClN_2O^+$ $[M + H]^+$: 421.2041; found: 421.2044.

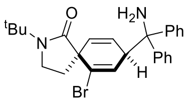

3-9-cis

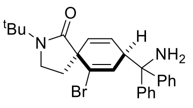

3-9-trans

8-(aminodiphenylmethyl)-6-bromo-2-(tert-butyl)-2-azaspiro[4.5]deca-6,9-dien-1-one (**3-9**)

3-9-cis was obtained (112 mg, 30% yield) as a white solid.

3-9-trans was obtained (112 mg, 30% yield) as a white solid.

d.r. (cis:trans) = 1:1

NMR:

[cis]: ^{1}H NMR (400 MHz, Chloroform-*d*) δ 7.64 (t, *J* = 6.6 Hz, 4H), 7.32 – 7.21 (m, 4H), 7.13 (q, *J* = 7.7 Hz, 2H), 5.95 (s, 1H), 5.63 (d, *J* = 10.0 Hz, 1H), 5.49 (d, *J* = 10.3 Hz, 1H), 4.33 (s, 1H), 3.62 – 3.50 (m, 1H), 3.48 – 3.37 (m, 1H), 2.52 – 2.40 (m, 1H), 2.05 (s, 2H), 1.95 – 1.85 (m, 1H), 1.40 (s, 9H). ^{13}C NMR (101 MHz, Chloroform-*d*) δ 172.4, 146.3, 146.1, 131.1, 130.2, 128.4, 128.2, 127.1, 126.2, 126.1, 126.0, 126.0, 124.9, 62.3, 54.9, 54.7, 48.3, 42.9, 31.7, 27.4.

[trans]: ^{1}H NMR (400 MHz, Chloroform-*d*) δ 7.54 (dd, *J* = 7.7, 4.8 Hz, 4H), 7.29 (q, *J* = 7.4 Hz, 4H), 7.17 (q, *J* = 6.9 Hz, 2H), 6.11 (d, *J* = 3.5 Hz, 1H), 5.72 – 5.61 (m, 2H), 4.42 (s, 1H), 3.59 – 3.50 (m, 1H), 3.45 – 3.36 (m, 1H), 2.36 – 2.27 (m, 1H), 1.87 – 1.73 (m, 3H), 1.40 (s, 9H). ^{13}C NMR (101 MHz, Chloroform-*d*) δ 172.5, 145.7, 145.5, 130.8, 130.5, 128.3, 128.3, 126.8, 126.5, 126.4, 126.0, 125.4, 63.0, 54.8, 54.7, 47.9, 42.9, 31.4, 27.3.

HRMS (APCI): calcd for $C_{26}H_{30}BrN_2O^+$ $[M + H]^+$: 465.1536; found: 465.1530.

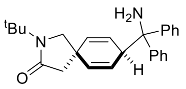

3-10-cis

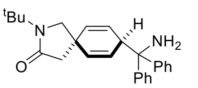

3-10-trans

8-(aminodiphenylmethyl)-2-(tert-butyl)-2-azaspiro[4.5]deca-6,9-dien-3-one (**3-10**)

3-10-cis was obtained (101 mg, 30% yield) as a brown oil.

3-10-trans was obtained (101 mg, 30% yield) as a brown oil.

d.r. (cis:trans) = 1:1

NMR:

[cis]: ^{1}H NMR (400 MHz, Chloroform-*d*) δ 7.55 (d, *J* = 7.8 Hz, 4H), 7.29 (t, *J* = 7.6 Hz, 4H), 7.17 (t, *J* = 7.3 Hz, 2H), 5.80 (d, *J* = 10.2 Hz, 2H), 5.61 (dd, *J* = 10.3, 3.1 Hz, 2H), 4.12 (s, 1H), 3.10 (s, 2H), 2.29 (s, 2H), 1.83 (s, 2H), 1.35 (s, 9H). ^{13}C NMR (101 MHz, Chloroform-*d*) δ 173.0, 146.0, 133.7, 128.2, 126.3, 126.3, 125.6, 63.0, 57.8, 53.9, 47.1, 44.4, 36.8, 27.6.

[trans]: ^{1}H NMR (400 MHz, Chloroform-*d*) δ 7.57 (d, *J* = 7.2 Hz, 4H), 7.28 (t, *J* = 7.7 Hz, 4H), 7.16 (t, *J* = 7.3 Hz, 2H), 5.82 (dd, *J* = 10.4, 1.9 Hz, 2H), 5.57 (dd, *J* = 10.3, 3.1 Hz, 2H), 4.15 – 4.11 (m, 1H), 3.27 (s, 2H), 2.23 (s, 2H), 1.86 (s, 2H), 1.36 (s, 9H). ^{13}C NMR (101 MHz, Chloroform-*d*) δ 172.9, 146.0, 133.7, 128.2, 126.3, 126.1, 125.7, 62.9, 57.8, 53.9, 47.1, 44.6, 36.9, 27.6.

HRMS (APCI): calcd for $C_{26}H_{31}N_2O^+$ $[M + H]^+$: 387.2431; found: 387.2430.

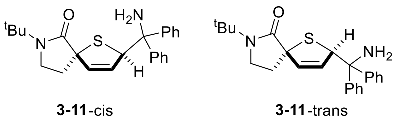

2-(aminodiphenylmethyl)-7-(tert-butyl)-1-thia-7-azaspiro[4.4]non-3-en-6-one (**3-11**)

3-11-cis was obtained (140 mg, 44.5% yield) as a brown oil.

3-11-trans was obtained (140 mg, 44.5% yield) as a brown oil.

d.r. (cis:trans) = 1:1

NMR:

[cis]: ^{1}H NMR (400 MHz, Chloroform-*d*) δ 7.53 (d, *J* = 7.3 Hz, 4H), 7.30 – 7.23 (m, 5H), 7.15 (t, *J* = 7.3 Hz, 2H), 5.76 (t, *J* = 2.2 Hz, 1H), 5.62 (dd, *J* = 6.3, 2.0 Hz, 1H), 5.41 (dd, *J* = 6.3, 2.4 Hz, 1H), 3.41 – 3.27 (m, 2H), 2.38 – 2.32 (m, 3H), 2.29 – 2.21 (m, 1H), 1.36 (s, 9H). ^{13}C NMR (101 MHz, Chloroform-*d*) δ 173.9, 147.9, 146.4, 134.6, 132.0, 128.2, 128.0, 126.4, 126.4, 126.3, 126.1, 68.2, 67.9, 63.6, 54.4, 42.7, 35.5, 27.4.

[trans]: ^{1}H NMR (400 MHz, Chloroform-*d*) δ 7.51 (dd, *J* = 15.8, 7.1 Hz, 4H), 7.30 – 7.24 (m, 4H), 7.16 (t, *J* = 7.3 Hz, 2H), 5.88 (t, *J* = 2.2 Hz, 1H), 5.65 (dd, *J* = 6.3, 2.0 Hz, 1H), 5.51 (dd, *J* = 6.3, 2.3 Hz, 1H), 3.40 – 3.28 (m, 2H), 2.40 – 2.32 (m, 1H), 2.20 – 2.13 (m, 1H), 2.03 (s, 2H), 1.38 (s, 9H). ^{13}C NMR (101 MHz, Chloroform-*d*) δ 173.8, 147.1, 145.7, 134.4, 132.3, 128.3, 128.1, 126.7, 126.6, 126.4, 126.0, 68.6, 67.8, 63.2, 54.5, 42.7, 35.4, 27.4.

HRMS (APCI): calcd for $C_{24}H_{29}N_2OS^+$ $[M + H]^+$: 393.1995; found: 393.1997.

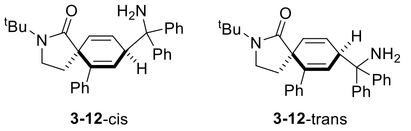

8-(aminodiphenylmethyl)-2-(tert-butyl)-6-phenyl-2-azaspiro[4.5]deca-6,9-dien-1-one (**3-12**)

3-12-cis was obtained (66 mg, 18% yield) as a brown oil.

3-12-trans was obtained (138 mg, 37% yield) as a brown oil.

d.r. (cis:trans) = 1:2.1

NMR:

[cis]: ^{1}H NMR (400 MHz, Chloroform-*d*) δ 7.74 (d, *J* = 8.0 Hz, 4H), 7.38 – 7.26 (m, 6H), 7.24 – 7.08 (m, 5H), 5.75 (d, *J* = 10.0 Hz, 1H), 5.65 – 5.55 (m, 2H), 4.49 (s, 1H), 3.32 (q, *J* = 8.0 Hz, 1H), 2.93 – 2.80 (m, 1H), 2.39 (s, 2H), 2.16 – 2.06 (m, 1H), 1.88 – 1.77 (m, 1H), 1.41 (s, 9H). ^{13}C NMR (101 MHz, Chloroform-*d*) δ 174.4, 147.0, 146.7, 142.5, 141.3, 131.8, 128.6, 128.2, 128.2, 127.9, 127.1, 127.0, 126.2, 126.2, 125.9, 125.9, 125.3, 62.5, 54.3, 52.4, 45.7, 42.4, 31.1, 27.4.

[trans]: ^{1}H NMR (400 MHz, Chloroform-*d*) δ 7.62 (d, *J* = 7.8 Hz, 4H), 7.33 – 7.27 (m, 4H), 7.24 – 7.11 (m, 7H), 5.81 – 5.69 (m, 3H), 4.48 (s, 1H), 3.31 – 3.23 (m, 1H), 2.87 – 2.79 (m, 1H), 2.04 – 1.94 (m, 1H), 1.82 (s, 2H), 1.77 – 1.70 (m, 1H), 1.35 (s, 9H). ^{13}C NMR (101 MHz, Chloroform-*d*) δ 174.3, 146.3, 146.2, 141.9, 131.6, 128.6, 128.2, 128.1, 127.8, 127.5, 127.0, 126.2, 126.2, 125.8, 63.2, 54.3, 52.3, 44.6, 42.4, 30.8, 27.3.

HRMS (APCI): calcd for $C_{32}H_{35}N_2O^+$ $[M + H]^+$: 463.2744; found: 463.2735.

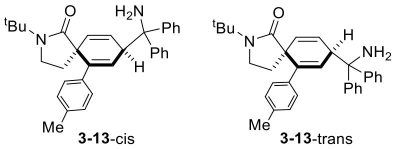

3-13-cis **3-13**-trans

8-(aminodiphenylmethyl)-2-(tert-butyl)-6-(p-tolyl)-2-azaspiro[4.5]deca-6,9-dien-1-one (**3-13**)

3-13-cis was obtained (60 mg, 16% yield) as a brown oil.

3-13-trans was obtained (143 mg, 37% yield) as a brown oil.

d.r. (cis:trans) = 1:2.4

NMR:

[cis]: ^{1}H NMR (400 MHz, Chloroform-*d*) δ 7.71 (t, *J* = 6.5 Hz, 4H), 7.30 (t, *J* = 7.5 Hz, 2H), 7.23 (t, *J* = 7.7 Hz, 2H), 7.15 (t, *J* = 7.4 Hz, 1H), 7.11 – 7.03 (m, 5H), 5.73 (d, *J* = 10.1 Hz, 1H), 5.63 – 5.51 (m, 2H), 4.45 (s, 1H), 3.31 (q, *J* = 8.1 Hz, 1H), 2.95 – 2.84 (m, 1H), 2.48 (s, 2H), 2.32 (s, 3H), 2.16 – 2.04 (m, 1H), 1.84 – 1.73 (m, 1H), 1.40 (s, 9H). ^{13}C NMR (101 MHz, Chloroform-*d*) δ 174.5, 146.9, 142.4, 138.4, 136.6, 131.8, 128.5, 128.4, 128.2, 128.1, 126.9, 126.2, 126.1, 125.9, 125.8, 125.3, 62.5, 54.3, 52.4, 45.7, 42.4, 31.1, 27.5, 21.0.

[trans]: ^{1}H NMR (400 MHz, Chloroform-*d*) δ 7.57 (d, *J* = 7.8 Hz, 4H), 7.25 – 7.18 (m, 4H), 7.11 (t, *J* = 7.2 Hz, 1H), 7.06 (t, *J* = 7.3 Hz, 1H), 7.03 – 6.94 (m, 4H), 5.74 – 5.67 (m, 2H), 5.64 (s, 1H), 4.43 (s, 1H), 3.25 – 3.17 (m, 1H), 2.85 – 2.76 (m, 1H), 2.24 (s, 3H), 1.99 – 1.91 (m, 1H), 1.86 (s, 2H), 1.70 – 1.62 (m, 1H), 1.31 (s, 9H). ^{13}C NMR (101 MHz, Chloroform-*d*) δ 174.3, 146.2, 141.8, 138.2, 136.5, 131.5, 128.4, 128.3, 128.1, 128.0, 127.2, 126.1, 126.1, 125.7, 63.1, 54.1, 52.2, 44.6, 42.3, 30.7, 27.2, 20.9.

HRMS (APCI): calcd for $C_{33}H_{37}N_2O^+$ $[M + H]^+$: 477.2900; found: 477.2895.

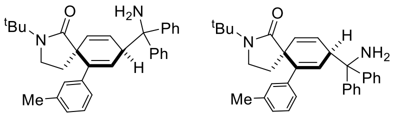

3-14-cis **3-14**-trans

8-(aminodiphenylmethyl)-2-(tert-butyl)-6-(m-tolyl)-2-azaspiro[4.5]deca-6,9-dien-1-one (**3-14**)

3-14-cis was obtained (67 mg, 17.5% yield) as a brown oil.

3-14-trans was obtained (147 mg, 38.5% yield) as a brown oil.

d.r. (cis:trans) = 1:2.2

NMR:

[cis]: ^{1}H NMR (400 MHz, Chloroform-*d*) δ 7.76 (d, *J* = 7.8 Hz, 4H), 7.34 (t, *J* = 7.6 Hz, 2H), 7.29 (d, *J* = 5.3 Hz, 2H), 7.21 – 7.16 (m, 2H), 7.13 (d, *J* = 7.0 Hz, 1H), 7.09 (d, *J* = 8.1 Hz, 1H), 7.06 – 7.00 (m, 2H), 5.77 (d, *J* = 10.1 Hz, 1H), 5.66 – 5.56 (m, 2H), 4.50 (t, *J* = 3.7 Hz, 1H), 3.38 – 3.29 (m, 1H), 2.92 – 2.85 (m, 1H), 2.55 (s, 2H), 2.33 (s, 3H), 2.19 – 2.09 (m, 1H), 1.87 – 1.79 (m, 1H), 1.43 (s, 9H). ^{13}C NMR (101 MHz, Chloroform-*d*) δ 174.4, 147.0, 146.7, 142.6, 141.3, 137.3, 131.8, 129.2, 128.2, 128.1, 127.8, 127.7, 126.8, 126.1, 125.8, 125.8, 125.7, 125.2, 62.5, 54.3, 52.3, 45.6, 42.3, 31.1, 27.5, 21.3.

[trans]: ^{1}H NMR (400 MHz, Chloroform-*d*) δ 7.62 (d, *J* = 7.9 Hz, 4H), 7.33 – 7.26 (m, 4H), 7.19 (d, *J* = 7.3 Hz, 1H), 7.15 – 7.10 (m, 2H), 7.04 (d, *J* = 7.5 Hz, 1H), 6.96 (s, 1H), 6.92 (d, *J* = 7.5 Hz, 1H), 5.79 – 5.72 (m, 2H), 5.69 (s, 1H), 4.48 (s, 1H), 3.30 – 3.23 (m, 1H), 2.87 – 2.79 (m, 1H), 2.28 (s, 3H), 2.11 (s, 2H), 2.04 – 1.96 (m, 1H), 1.78 – 1.70 (m, 1H), 1.36 (s, 9H). ^{13}C NMR (101 MHz, Chloroform-*d*) δ 174.3, 146.2, 142.0, 141.1, 137.2, 131.7, 129.2, 128.1, 128.1, 127.7, 127.7, 127.2, 126.2, 126.2, 126.1, 125.8, 125.7, 63.2, 54.2, 52.3, 44.5, 42.4, 30.8, 27.3, 21.2.

HRMS (APCI): calcd for $C_{33}H_{37}N_2O^+$ $[M + H]^+$: 477.2900; found: 477.2895.

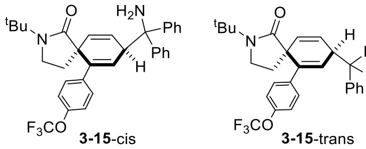

8-(aminodiphenylmethyl)-2-(tert-butyl)-6-(4-(trifluoromethoxy)phenyl)-2-azaspiro[4.5]deca-6,9-dien-1-one (**3-15**)

3-15-cis was obtained (74 mg, 17% yield) as a brown oil.

3-15-trans was obtained (154 mg, 35% yield) as a brown oil.

d.r. (cis:trans) = 1:2.1

NMR:

[cis]: ^{1}H NMR (400 MHz, Chloroform-*d*) δ 7.71 (d, *J* = 7.9 Hz, 4H), 7.35 – 7.27 (m, 3H), 7.25 – 7.15 (m, 4H), 7.13 – 7.05 (m, 3H), 5.74 (d, *J* = 10.2 Hz, 1H), 5.64 – 5.54 (m, 2H), 4.46 (s, 1H), 3.35 (q, *J* = 8.2 Hz, 1H), 3.00 – 2.87 (m, 1H), 2.39 (s, 2H), 2.09 – 2.00 (m, 1H), 1.86 – 1.75 (m, 1H), 1.39 (s, 9H). ^{13}C NMR (101 MHz, Chloroform-*d*) δ 174.2, 148.3 (d, *J* = 2.7 Hz), 146.8, 146.7, 141.3, 140.0, 131.2, 130.0, 128.3, 128.2, 126.2, 126.1, 126.0, 126.0, 125.3, 120.4, 62.6, 54.4, 52.4, 45.7, 42.3, 30.9, 27.4. ^{19}F NMR (376 MHz, Chloroform-*d*) δ -57.8.

[trans]: ^{1}H NMR (400 MHz, Chloroform-*d*) δ 7.61 (d, *J* = 7.2 Hz, 4H), 7.29 (q, *J* = 7.7 Hz, 4H), 7.22 – 7.13 (m, 4H), 7.08 (d, *J* = 8.3 Hz, 2H), 5.82 – 5.77 (m, 1H), 5.76 – 5.71 (m, 2H), 4.45 (q, *J* = 2.6 Hz, 1H), 3.34 – 3.24 (m, 1H), 2.93 – 2.85 (m, 1H), 1.95 – 1.88 (m, 1H), 1.82 (s, 2H), 1.74 – 1.66 (m, 1H), 1.34 (s, 9H). ^{13}C NMR (101 MHz, Chloroform-*d*) δ 174.1, 148.2 (d, *J* = 1.8 Hz), 146.2, 146.0, 140.5, 139.8, 131.0, 130.0, 128.6, 128.2, 128.1, 126.3, 126.2, 126.2, 125.7, 120.3 (d, *J* = 257.2 Hz), 120.3, 63.3, 54.3, 52.2, 44.7, 42.3, 30.7, 27.2. ^{19}F NMR (376 MHz, Chloroform-*d*) δ -57.8.

HRMS (APCI): calcd for $C_{33}H_{34}F_3N_2O_2^+$ [M + H]$^+$: 547.2567; found: 547.2561.

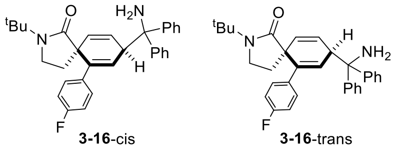

8-(aminodiphenylmethyl)-2-(tert-butyl)-6-(4-fluorophenyl)-2-azaspiro[4.5]deca-6,9-dien-1-one (**3-16**)

3-16-cis was obtained (63 mg, 17% yield) as a brown oil.

3-16-trans was obtained (121 mg, 31% yield) as a brown oil.

d.r. (cis:trans) = 1:1.9

NMR:

[cis]: ^{1}H NMR (400 MHz, Chloroform-*d*) δ 7.76 – 7.68 (m, 4H), 7.30 (t, *J* = 7.6 Hz, 2H), 7.25 (t, *J* = 7.7 Hz, 2H), 7.18 – 7.13 (m, 3H), 7.09 (t, *J* = 7.2 Hz, 1H), 6.93 (t, *J* = 8.7 Hz, 2H), 5.74 (d, *J* = 10.0 Hz, 1H), 5.63 – 5.54 (m, 2H), 4.50 – 4.42 (m, 1H), 3.38 – 3.27 (m, 1H), 2.94 – 2.87 (m, 1H), 2.24 (s, 2H), 2.10 – 2.02 (m, 1H), 1.84 – 1.76 (m, 1H), 1.39 (s, 9H). ^{13}C NMR (101 MHz, Chloroform-*d*) δ 174.3, 161.9 (d, *J* = 246.2 Hz), 146.8 (d, *J* = 14.1 Hz), 141.5, 137.2 (d, *J* = 3.1 Hz), 131.4, 130.2, 130.1, 128.2, 128.1, 127.7, 126.1, 126.1, 125.9, 125.2, 114.8, 114.6, 62.4, 54.3, 52.4, 45.7, 42.3, 30.9, 27.4. ^{19}F NMR (376 MHz, Chloroform-*d*) δ -115.3.

[trans]: ^{1}H NMR (400 MHz, Chloroform-*d*) δ 7.63 – 7.58 (m, 4H), 7.32 – 7.24 (m, 4H), 7.20 – 7.06 (m, 4H), 6.91 (t, *J* = 8.7 Hz, 2H), 5.81 – 5.76 (m, 1H), 5.75 – 5.70 (m, 2H), 4.45 (q, *J* = 2.5 Hz, 1H), 3.30 –

3.22 (m, 1H), 2.89 – 2.81 (m, 1H), 1.97 – 1.88 (m, 1H), 1.82 (s, 2H), 1.74 – 1.65 (m, 1H), 1.34 (s, 9H). ^{13}C NMR (101 MHz, Chloroform-*d*) δ 174.1, 161.7 (d, *J* = 246.0 Hz), 146.1 (d, *J* = 17.3 Hz), 140.7, 137.0 (d, *J* = 3.4 Hz), 131.1, 130.1, 130.1, 128.0, 128.0, 127.9, 126.1, 126.1, 126.0, 125.6, 114.6, 114.4, 63.2, 54.1, 52.2, 44.5, 42.2, 30.6, 27.2. ^{19}F NMR (376 MHz, Chloroform-*d*) δ -115.2.
HRMS (APCI): calcd for $C_{32}H_{34}FN_2O^+$ $[M + H]^+$: 481.2650; found: 481.2645.

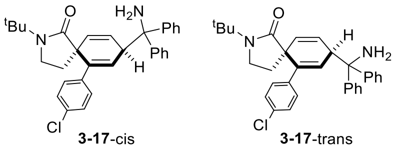

3-17-cis **3-17**-trans

8-(aminodiphenylmethyl)-2-(tert-butyl)-6-(4-chlorophenyl)-2-azaspiro[4.5]deca-6,9-dien-1-one (**3-17**)
3-17-cis was obtained (67 mg, 17% yield) as a brown oil.
3-17-trans was obtained (148 mg, 37% yield) as a brown oil.
d.r. (cis:trans) = 1:2.2
NMR:
[cis]: ^{1}H NMR (400 MHz, Chloroform-*d*) δ 7.73 (t, *J* = 7.9 Hz, 4H), 7.32 (t, *J* = 7.6 Hz, 2H), 7.28 – 7.21 (m, 4H), 7.19 – 7.08 (m, 4H), 5.75 (d, *J* = 10.1 Hz, 1H), 5.66 – 5.53 (m, 2H), 4.47 (s, 1H), 3.36 (q, *J* = 8.0 Hz, 1H), 3.02 – 2.94 (m, 1H), 2.23 (s, 2H), 2.11 – 1.99 (m, 1H), 1.85 – 1.77 (m, 1H), 1.41 (s, 9H). ^{13}C NMR (101 MHz, Chloroform-*d*) δ 146.8, 146.7, 141.4, 139.8, 131.2, 129.9, 128.2, 128.2, 128.0, 126.1, 126.1, 125.9, 125.2, 62.5, 54.4, 52.3, 45.7, 42.2, 30.9, 27.4.
[trans]: ^{1}H NMR (400 MHz, Chloroform-*d*) δ 7.64 – 7.53 (m, 4H), 7.27 (q, *J* = 8.0 Hz, 4H), 7.21 – 7.10 (m, 4H), 7.04 (d, *J* = 8.1 Hz, 2H), 5.77 (d, *J* = 10.3 Hz, 1H), 5.71 (d, *J* = 10.4 Hz, 2H), 4.43 (s, 1H), 3.28 (q, *J* = 8.2 Hz, 1H), 2.96 – 2.84 (m, 1H), 1.96 – 1.84 (m, 1H), 1.77 – 1.62 (m, 3H), 1.34 (s, 9H). ^{13}C NMR (101 MHz, Chloroform-*d*) δ 174.1, 146.2, 146.0, 140.7, 139.6, 132.9, 131.0, 129.9, 128.4, 128.2, 128.1, 128.0, 126.3, 126.2, 126.1, 125.7, 63.3, 54.3, 52.2, 44.6, 42.3, 30.6, 27.3.
HRMS (APCI): calcd for $C_{32}H_{34}ClN_2O^+$ $[M + H]^+$: 497.2354; found: 497.2349.

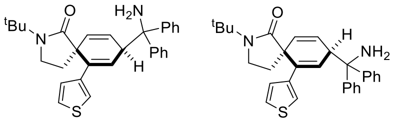

3-18-cis **3-18**-trans

8-(aminodiphenylmethyl)-2-(tert-butyl)-6-(thiophen-3-yl)-2-azaspiro[4.5]deca-6,9-dien-1-one (**3-18**)
3-18-cis was obtained (51 mg, 14% yield) as a brown oil.
3-18-trans was obtained (148 mg, 39% yield) as a brown oil.
d.r. (cis:trans) = 1:2.9
NMR:
[cis]: ^{1}H NMR (400 MHz, Chloroform-*d*) δ 7.73 (d, *J* = 7.3 Hz, 4H), 7.36 – 7.25 (m, 4H), 7.21 – 7.12 (m, 3H), 7.03 (dd, *J* = 3.0, 1.3 Hz, 1H), 6.95 (dd, *J* = 5.0, 1.3 Hz, 1H), 5.80 – 5.74 (m, 2H), 5.62 – 5.55 (m, 1H), 4.46 (t, *J* = 4.1 Hz, 1H), 3.48 – 3.37 (m, 1H), 3.22 – 3.15 (m, 1H), 2.27 – 2.18 (m, 3H), 1.85 – 1.77 (m, 1H), 1.45 (s, 9H). ^{13}C NMR (101 MHz, Chloroform-*d*) δ 174.6, 146.9, 146.7, 141.5, 137.3, 130.8, 128.2, 128.2, 127.9, 127.2, 126.1, 125.9, 125.9, 125.0, 124.8, 121.7, 62.6, 54.3, 52.2, 45.7, 42.2, 31.0, 27.4.

[trans]: ^{1}H NMR (400 MHz, Chloroform-*d*) δ 7.62 (dd, *J* = 7.9, 4.9 Hz, 4H), 7.35 – 7.25 (m, 4H), 7.20 – 7.13 (m, 3H), 6.99 (d, *J* = 3.5 Hz, 1H), 6.89 (d, *J* = 4.9 Hz, 1H), 5.90 (d, *J* = 3.2 Hz, 1H), 5.77 (s, 2H), 4.47 (d, *J* = 3.3 Hz, 1H), 3.37 (q, *J* = 8.4 Hz, 1H), 3.20 – 3.11 (m, 1H), 2.16 – 2.06 (m, 1H), 1.79 (s, 2H), 1.71 – 1.63 (m, 1H), 1.41 (s, 9H). ^{13}C NMR (101 MHz, Chloroform-*d*) δ 174.5, 146.1, 146.0, 141.0, 136.5, 130.5, 128.0, 128.0, 127.7, 127.4, 126.6, 126.1, 126.1, 126.0, 125.3, 124.6, 121.5, 63.2, 54.1, 51.9, 44.7, 42.1, 30.8, 27.2.
HRMS (APCI): calcd for $C_{30}H_{33}N_2OS^+$ $[M + H]^+$: 469.2308; found: 469.2303.

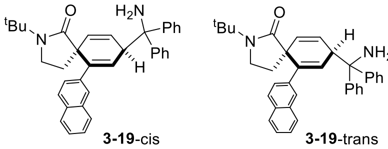

3-19-cis **3-19**-trans

8-(aminodiphenylmethyl)-2-(tert-butyl)-6-(naphthalen-2-yl)-2-azaspiro[4.5]deca-6,9-dien-1-one (**3-19**)
3-19-cis was obtained (33 mg, 8% yield) as a brown oil.
3-19-trans was obtained (111 mg, 27% yield) as a brown oil.
d.r. (cis:trans) = 1:3.4
NMR:
[cis]: ^{1}H NMR (400 MHz, Chloroform-*d*) δ 7.81 – 7.78 (m, 1H), 7.75 (d, *J* = 8.8 Hz, 3H), 7.73 – 7.69 (m, 3H), 7.66 (s, 1H), 7.47 – 7.43 (m, 2H), 7.36 – 7.33 (m, 1H), 7.31 (d, *J* = 8.0 Hz, 2H), 7.22 (t, *J* = 7.6 Hz, 2H), 7.17 (t, *J* = 7.3 Hz, 1H), 7.06 (t, *J* = 7.3 Hz, 1H), 5.78 (d, *J* = 10.1 Hz, 1H), 5.68 (dd, *J* = 3.8, 1.8 Hz, 1H), 5.66 – 5.60 (m, 1H), 4.54 – 4.48 (m, 1H), 3.36 – 3.25 (m, 1H), 2.89 – 2.82 (m, 1H), 2.31 (s, 2H), 2.16 – 2.08 (m, 1H), 1.85 – 1.78 (m, 1H), 1.41 (s, 9H). ^{13}C NMR (101 MHz, Chloroform-*d*) δ 174.5, 147.0, 146.7, 142.5, 138.9, 132.9, 132.3, 131.6, 128.2, 128.2, 127.9, 127.8, 127.4, 127.4, 127.1, 127.1, 126.2, 126.1, 126.1, 125.9, 125.7, 125.3, 62.5, 54.4, 52.6, 45.9, 42.4, 31.1, 27.5.
[trans]: ^{1}H NMR (400 MHz, Chloroform-*d*) δ 7.81 – 7.75 (m, 2H), 7.73 (d, *J* = 8.5 Hz, 1H), 7.67 (d, *J* = 7.8 Hz, 4H), 7.62 (s, 1H), 7.48 – 7.40 (m, 2H), 7.38 – 7.25 (m, 5H), 7.21 (t, *J* = 7.3 Hz, 1H), 7.14 (t, *J* = 7.3 Hz, 1H), 5.84 (d, *J* = 12.9 Hz, 2H), 5.80 (d, *J* = 10.1 Hz, 1H), 4.54 (s, 1H), 3.30 – 3.24 (m, 1H), 2.87 – 2.79 (m, 1H), 2.11 – 1.99 (m, 1H), 1.97 (s, 2H), 1.80 – 1.71 (m, 1H), 1.38 (s, 9H). ^{13}C NMR (101 MHz, Chloroform-*d*) δ 174.4, 146.2, 146.2, 141.8, 138.7, 132.8, 132.2, 131.4, 128.2, 128.2, 128.1, 127.8, 127.4, 127.1, 127.0, 126.2, 126.2, 126.2, 126.0, 125.8, 125.7, 63.3, 54.3, 52.5, 44.7, 42.4, 30.8, 27.3.
HRMS (APCI): calcd for $C_{36}H_{37}N_2O^+$ $[M + H]^+$: 513.2900; found: 513.2896.

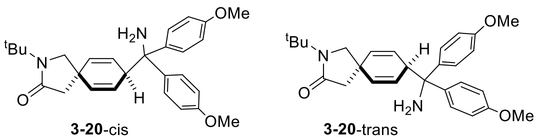

3-20-cis **3-20**-trans

8-(aminobis(4-methoxyphenyl)methyl)-2-(tert-butyl)-2-azaspiro[4.5]deca-6,9-dien-3-one (**3-20**)
3-20-cis was obtained (72 mg, 19% yield) as a brown oil.
3-20-trans was obtained (72 mg, 19% yield) as a brown oil.
d.r. (cis:trans) = 1:1
NMR:
[cis]: ^{1}H NMR (400 MHz, Chloroform-*d*) δ 7.55 (d, *J* = 8.9 Hz, 4H), 6.80 (d, *J* = 8.8 Hz, 4H), 5.63 (d, *J* = 10.4 Hz, 2H), 5.56 (dd, *J* = 10.4, 3.3 Hz, 2H), 4.18 – 4.11 (m, 1H), 3.74 (s, 6H), 3.43 (t, *J* = 6.7 Hz,

2H), 2.14 (s, 2H), 1.95 (t, *J* = 6.8 Hz, 2H), 1.38 (s, 9H). ^{13}C NMR (101 MHz, Chloroform-*d*) δ 175.0, 157.6, 139.4, 130.3, 127.2, 127.1, 113.5, 61.4, 55.1, 54.2, 49.4, 44.8, 41.9, 33.7, 27.6.

[trans]: ^{1}H NMR (400 MHz, Chloroform-*d*) δ 7.47 (d, *J* = 8.9 Hz, 4H), 6.81 (d, *J* = 8.9 Hz, 4H), 5.75 – 5.64 (m, 4H), 4.23 (s, 1H), 3.74 (s, 6H), 3.42 (t, *J* = 6.8 Hz, 2H), 1.91 (t, *J* = 6.8 Hz, 2H), 1.85 (s, 2H), 1.38 (s, 9H). ^{13}C NMR (101 MHz, Chloroform-*d*) δ 175.0, 157.7, 138.8, 130.1, 127.5, 127.0, 113.5, 61.9, 55.1, 54.2, 49.3, 44.1, 42.0, 33.4, 27.5.

HRMS (APCI): calcd for $C_{28}H_{35}N_2O_3^+$ [M + H]$^+$: 447.2642; found: 447.2638.

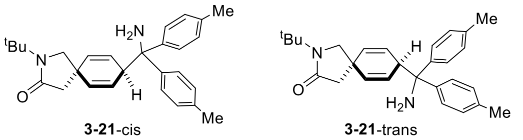

3-21-cis **3-21**-trans

8-(aminodi-p-tolylmethyl)-2-(tert-butyl)-2-azaspiro[4.5]deca-6,9-dien-3-one (**3-21**)

3-21-cis was obtained (77 mg, 23% yield) as a brown oil.

3-21-trans was obtained (59 mg, 18% yield) as a brown oil.

d.r. (cis:trans) = 1.3:1

NMR:

[cis]: ^{1}H NMR (400 MHz, Chloroform-*d*) δ 7.55 (d, *J* = 7.9 Hz, 4H), 7.08 (d, *J* = 7.9 Hz, 4H), 5.64 (d, *J* = 10.4 Hz, 2H), 5.61 – 5.56 (m, 2H), 4.21 (s, 1H), 3.43 (t, *J* = 6.8 Hz, 2H), 2.27 (s, 6H), 1.95 (t, *J* = 6.8 Hz, 2H), 1.41 (s, 2H), 1.39 (s, 9H). ^{13}C NMR (101 MHz, Chloroform-*d*) δ 174.9, 144.0, 135.2, 130.2, 128.8, 127.0, 125.9, 61.8, 54.1, 49.3, 44.6, 41.8, 33.7, 27.5, 20.8.

[trans]: ^{1}H NMR (400 MHz, Chloroform-*d*) δ 7.46 (d, *J* = 8.0 Hz, 4H), 7.08 (d, *J* = 7.9 Hz, 4H), 5.75 – 5.64 (m, 4H), 4.29 (s, 1H), 3.43 (t, *J* = 6.8 Hz, 2H), 2.27 (s, 6H), 1.93 (t, *J* = 6.9 Hz, 2H), 1.75 (s, 2H), 1.38 (s, 9H). ^{13}C NMR (101 MHz, Chloroform-*d*) δ 175.1, 143.6, 135.6, 130.1, 129.0, 127.7, 125.8, 62.3, 54.2, 49.3, 44.0, 42.1, 33.5, 27.6, 20.8.

HRMS (APCI): calcd for $C_{28}H_{35}N_2O^+$ [M + H]$^+$: 415.2744; found: 415.2737.

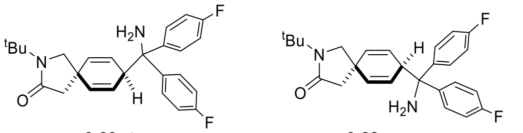

3-22-cis **3-22**-trans

8-(aminobis(4-fluorophenyl)methyl)-2-(tert-butyl)-2-azaspiro[4.5]deca-6,9-dien-3-one (**3-22**)

3-22-cis was obtained (97 mg, 29% yield) as a white solid.

3-22-trans was obtained (89 mg, 26% yield) as a white solid.

d.r. (cis:trans) = 1.1:1

NMR:

[cis]: ^{1}H NMR (400 MHz, Chloroform-*d*) δ 7.65 – 7.56 (m, 4H), 6.94 (t, *J* = 8.7 Hz, 4H), 5.66 (d, *J* = 10.3 Hz, 2H), 5.51 (dd, *J* = 10.3, 3.5 Hz, 2H), 4.20 – 4.14 (m, 1H), 3.43 (t, *J* = 6.7 Hz, 2H), 2.07 (s, 2H), 1.95 (t, *J* = 6.7 Hz, 2H), 1.37 (s, 9H). ^{13}C NMR (101 MHz, Chloroform-*d*) δ 174.7, 162.3, 159.8, 142.5, 142.5, 130.8, 127.7, 127.7, 126.3, 115.0, 114.7, 61.5, 54.2, 49.4, 44.6, 41.9, 33.5, 27.5. ^{19}F NMR (376 MHz, Chloroform-*d*) δ -117.2.

[trans]: ^{1}H NMR (400 MHz, Chloroform-*d*) δ 7.56 – 7.48 (m, 4H), 6.95 (t, *J* = 8.7 Hz, 4H), 5.70 (dd, *J* = 10.2, 1.7 Hz, 2H), 5.65 (dd, *J* = 10.5, 2.5 Hz, 2H), 4.28 – 4.20 (m, 1H), 3.42 (t, *J* = 6.8 Hz, 2H), 1.90 (t, *J* = 6.7 Hz, 2H), 1.62 (s, 2H), 1.37 (s, 9H). ^{13}C NMR (101 MHz, Chloroform-*d*) δ 174.7, 162.4, 159.9,

141.9, 141.9, 130.7, 127.7, 127.6, 126.8, 115.1, 114.9, 62.1, 54.2, 49.3, 44.0, 42.0, 33.3, 27.5. ^{19}F NMR (376 MHz, Chloroform-*d*) δ -116.7.

HRMS (APCI): calcd for $C_{26}H_{29}F_2N_2O^+$ [M + H]$^+$: 423.2242; found: 423.2237.

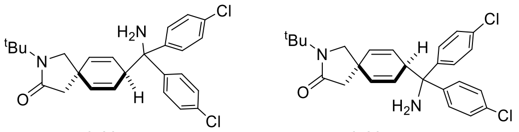

3-23-cis 3-23-trans

8-(aminobis(4-chlorophenyl)methyl)-2-(tert-butyl)-2-azaspiro[4.5]deca-6,9-dien-3-one (**3-23**)

3-23-cis was obtained (100 mg, 28% yield) as a white solid.

3-23-trans was obtained (151 mg, 41% yield) as a white solid.

d.r. (cis:trans) = 1:1.5

NMR:

[cis]: ^{1}H NMR (400 MHz, Chloroform-*d*) δ 7.58 (d, *J* = 8.7 Hz, 4H), 7.24 (d, *J* = 8.7 Hz, 4H), 5.66 (dd, *J* = 10.3, 1.5 Hz, 2H), 5.51 (dd, *J* = 10.2, 3.5 Hz, 2H), 4.18 – 4.14 (m, 1H), 3.44 (t, *J* = 6.8 Hz, 2H), 2.04 (s, 2H), 1.96 (t, *J* = 6.8 Hz, 2H), 1.38 (s, 9H). ^{13}C NMR (101 MHz, Chloroform-*d*) δ 174.7, 145.0, 132.0, 131.0, 128.4, 127.6, 126.1, 61.7, 54.3, 49.4, 44.3, 42.0, 33.5, 27.6.

[trans]: ^{1}H NMR (400 MHz, Chloroform-*d*) δ 7.50 (d, *J* = 8.2 Hz, 4H), 7.24 (d, *J* = 8.1 Hz, 4H), 5.71 (d, *J* = 10.0 Hz, 2H), 5.63 (dd, *J* = 10.5, 2.7 Hz, 2H), 4.24 (s, 1H), 3.43 (t, *J* = 6.8 Hz, 2H), 1.91 (t, *J* = 6.8 Hz, 2H), 1.62 (s, 2H), 1.37 (s, 9H). ^{13}C NMR (101 MHz, Chloroform-*d*) δ 174.6, 144.5, 132.2, 130.9, 128.4, 127.5, 126.6, 62.2, 54.2, 49.3, 43.6, 42.0, 33.2, 27.5.

HRMS (APCI): calcd for $C_{26}H_{29}Cl_2N_2O^+$ [M + H]$^+$: 455.1651; found: 455.1646.

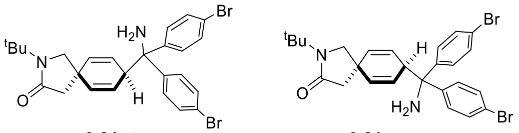

3-24-cis 3-24-trans

8-(aminobis(4-bromophenyl)methyl)-2-(tert-butyl)-2-azaspiro[4.5]deca-6,9-dien-3-one (**3-24**)

3-24-cis was obtained (141 mg, 32% yield) as an off-white solid.

3-24-trans was obtained (169 mg, 39% yield) as an off-white solid.

d.r. (cis:trans) = 1:1.2

NMR:

[cis]: ^{1}H NMR (400 MHz, Chloroform-*d*) δ 7.52 (d, *J* = 8.4 Hz, 4H), 7.39 (d, *J* = 8.3 Hz, 4H), 5.66 (d, *J* = 10.2 Hz, 2H), 5.50 (dd, *J* = 10.3, 3.5 Hz, 2H), 4.16 (s, 1H), 3.44 (t, *J* = 6.7 Hz, 2H), 2.01 (s, 2H), 1.96 (t, *J* = 6.8 Hz, 2H), 1.38 (s, 9H). ^{13}C NMR (101 MHz, Chloroform-*d*) δ 174.7, 145.5, 131.3, 131.1, 128.0, 126.1, 120.2, 61.8, 54.3, 49.4, 44.2, 42.0, 33.5, 27.6.

[trans]: ^{1}H NMR (400 MHz, Chloroform-*d*) δ 7.44 (d, *J* = 8.8 Hz, 4H), 7.38 (d, *J* = 8.9 Hz, 4H), 5.70 (dd, *J* = 10.3, 1.9 Hz, 2H), 5.62 (dd, *J* = 10.3, 2.7 Hz, 2H), 4.24 – 4.20 (m, 1H), 3.42 (t, *J* = 6.8 Hz, 2H), 1.90 (t, *J* = 6.8 Hz, 2H), 1.62 (s, 2H), 1.37 (s, 9H). ^{13}C NMR (101 MHz, Chloroform-*d*) δ 174.6, 144.9, 131.5, 131.4, 130.9, 128.4, 127.8, 126.5, 120.4, 62.3, 54.2, 49.3, 43.5, 42.0, 33.2, 27.5.

HRMS (APCI): calcd for $C_{26}H_{29}Br_2N_2O^+$ [M + H]$^+$: 543.0641; found: 543.0638.

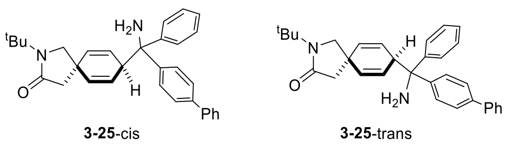

8-([1,1'-biphenyl]-4-yl(amino)(phenyl)methyl)-2-(tert-butyl)-2-azaspiro[4.5]deca-6,9-dien-3-one (**3-25**)

3-25-cis was obtained (80 mg, 21% yield) as a brown oil.

3-25-trans was obtained (72 mg, 20% yield) as a brown oil.

d.r. (cis:trans) = 1.1:1

NMR:

[cis]: ^{1}H NMR (400 MHz, Chloroform-*d*) δ 7.78 (dd, *J* = 11.6, 8.0 Hz, 4H), 7.57 (dd, *J* = 14.4, 7.9 Hz, 4H), 7.43 (t, *J* = 7.5 Hz, 2H), 7.34 (t, *J* = 7.7 Hz, 3H), 7.19 (t, *J* = 7.3 Hz, 1H), 5.75 – 5.58 (m, 4H), 4.37 – 4.28 (m, 1H), 3.47 (t, *J* = 6.7 Hz, 2H), 2.18 (s, 2H), 2.00 (t, *J* = 6.7 Hz, 2H), 1.43 (s, 9H). ^{13}C NMR (101 MHz, Chloroform-*d*) δ 174.9, 146.7, 145.9, 140.7, 138.6, 130.6, 130.5, 128.6, 128.2, 126.9, 126.8, 126.8, 126.6, 126.1, 126.0, 62.1, 54.2, 49.4, 44.7, 41.9, 33.7, 27.6.

[trans]: ^{1}H NMR (400 MHz, Chloroform-*d*) δ 7.67 (dd, *J* = 13.0, 8.0 Hz, 4H), 7.54 (dd, *J* = 12.2, 7.9 Hz, 4H), 7.40 (t, *J* = 7.6 Hz, 2H), 7.31 (t, *J* = 7.0 Hz, 3H), 7.18 (t, *J* = 7.3 Hz, 1H), 5.82 – 5.69 (m, 4H), 4.38 (s, 1H), 3.43 (t, *J* = 6.7 Hz, 2H), 1.93 (t, *J* = 6.8 Hz, 2H), 1.72 (s, 2H), 1.39 (s, 9H). ^{13}C NMR (101 MHz, Chloroform-*d*) δ 174.9, 146.2, 145.3, 140.5, 138.8, 130.4, 130.3, 128.6, 128.3, 127.3, 127.3, 127.0, 126.9, 126.8, 126.5, 126.2, 126.0, 62.6, 54.2, 49.3, 44.0, 42.0, 33.4, 27.5.

HRMS (APCI): calcd for $C_{32}H_{35}N_2O^+$ $[M + H]^+$: 463.2744; found: 463.2737.

V.4 The synthesis of 7-1

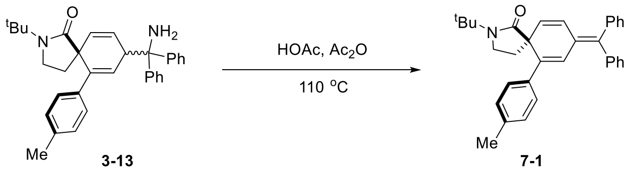

Standard Conditions: 3-13 (0.3 mmol), in 3 mL of HOAc/Ac_2O (*v*/*v* = 1/2) under Ar atmosphere at 110 °C for 20 h.

Workup: The reaction mixture was carefully adjusted pH value to about 7 with K_2CO_3 (aq), then it was washed with brine (saturated NaCl(aq)) and extracted with EA multiple times. The combined organic layers were then dried over Na_2SO_4, concentrated under reduced pressure, and purified by column chromatography to isolate target product **7-1**.

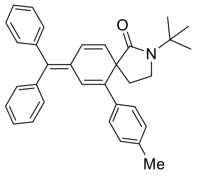

2-(tert-butyl)-8-(diphenylmethylene)-6-(p-tolyl)-2-azaspiro[4.5]deca-6,9-dien-1-one (**7-1**)

The title compound was obtained as a beige solid (69 mg, 50% yield).

NMR: ^{1}H NMR (400 MHz, Chloroform-*d*) δ 7.36 – 7.21 (m, 10H), 7.19 (d, *J* = 7.7 Hz, 2H), 7.10 (d, *J* = 7.8 Hz, 2H), 6.69 (s, 1H), 6.65 (d, *J* = 9.9 Hz, 1H), 5.76 (d, *J* = 9.9 Hz, 1H), 3.39 (q, *J* = 8.2 Hz, 1H),

3.09 – 3.00 (m, 1H), 2.34 (s, 3H), 2.26 – 2.14 (m, 1H), 1.95 – 1.86 (m, 1H), 1.44 (s, 9H). ^{13}C NMR (101 MHz, Chloroform-*d*) δ 141.8, 141.5, 140.7, 139.8, 138.1, 136.7, 130.6, 130.6, 128.6, 128.2, 128.1, 127.8, 127.7, 126.9, 126.9, 125.8, 54.9, 54.4, 42.3, 31.3, 27.4, 21.0.

HRMS (APCI): calcd for $C_{33}H_{34}NO^+$ $[M + H]^+$: 460.2635; found: 460.2632.

V.5 The synthesis of cyclohexadienyl amine

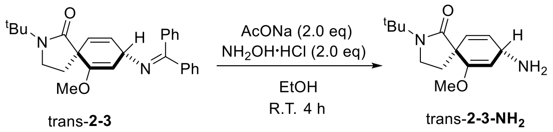

trans-**2-3** → trans-**2-3-NH2**

To a solution of trans-**2-3** (125 mg, 0.3 mmol) in EtOH (10 mL), AcONa (50 mg, 2.0 equiv) and $NH_2OH{\cdot}HCl$ (42 mg, 2.0 equiv) were added. The reaction mixture was stirred at R. T. for 4 hours, then the solvent was removed under reduced pressure, and 10 mL of water was added. The mixture was adjusted to pH about 10 with 1 M NaOH, extracted with DCM three times, The combined organic layers was dried over Na_2SO_4, concentrated under reduced pressure, and purified by column chromatography to isolate the target product, trans-**2-3-NH$_2$** (59 mg, 78% yield).

NMR: ^1H NMR (400 MHz, Chloroform-*d*) δ 5.80 – 5.72 (m, 1H), 5.36 (dd, *J* = 9.8, 1.6 Hz, 1H), 4.77 (dd, *J* = 3.8, 1.5 Hz, 1H), 4.06 – 4.01 (m, 1H), 3.42 (s, 3H), 3.39 – 3.33 (m, 1H), 3.31 – 3.26 (m, 1H), 3.16 – 3.04 (m, 2H), 2.25 – 2.15 (m, 1H), 1.80 – 1.72 (m, 1H), 1.24 (s, 9H). ^{13}C NMR (101 MHz, Chloroform-*d*) δ 173.7, 155.5, 130.0, 127.4, 98.0, 54.1, 51.1 (spiro C), 51.1 (OMe), 46.6, 43.1, 29.9, 27.1.

VI. Unsuccessful substrates for chemodivergent reactions

VI. 1 The following compounds were used in carboamination under the corresponding standard conditions, but no desired products were formed.

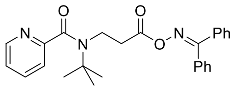
1-U1

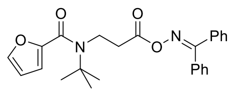
1-U2

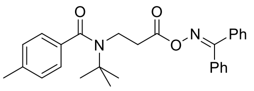
1-U3

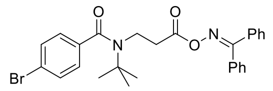
1-U4

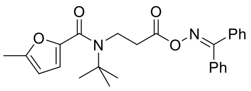
1-U5

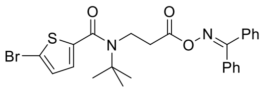
1-U6

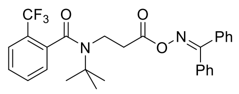
1-U7

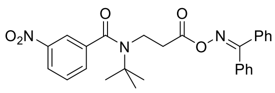
1-U8

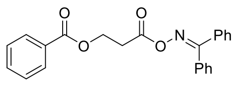
1-U9

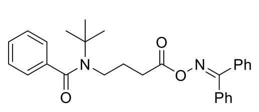
1-U10

VI. 2 The following compounds were used in rearomatization with compound **4-1** under the corresponding standard conditions, but no desired products were formed.

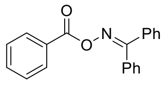

5-U1 (ND)

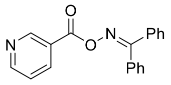

5-U2 (ND)

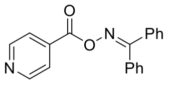

5-U3 (ND)

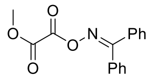

5-U4 (ND)

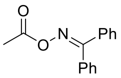

5-U5 (trace)

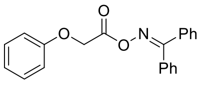

5-U6 (trace)

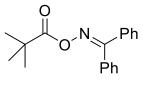

5-U7 (trace)

VI. 3 The following compounds were used in carbo-aminoalkylation under the corresponding standard conditions, but no desired products were formed.

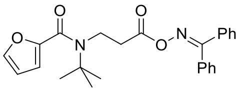

1-U2

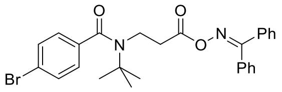

1-U4

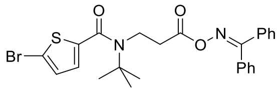

1-U6

VII. Control experiments

(A) The composition analysis of the reaction mixture for carboamination

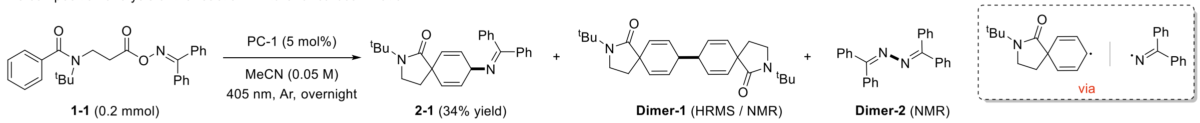

(B) The evidence of HAT for DMPU

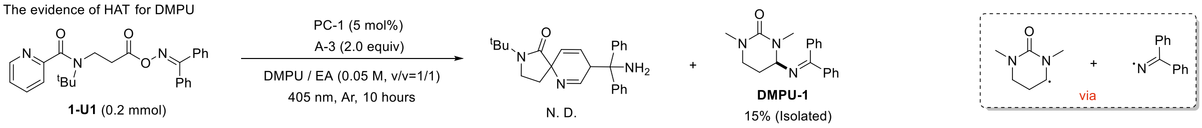

(C) The experiment for cross-validation

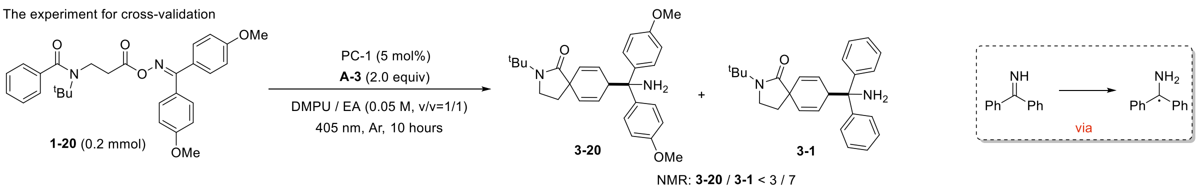

(D) UV-vis absorption spectra and Stern-Volmer experiments

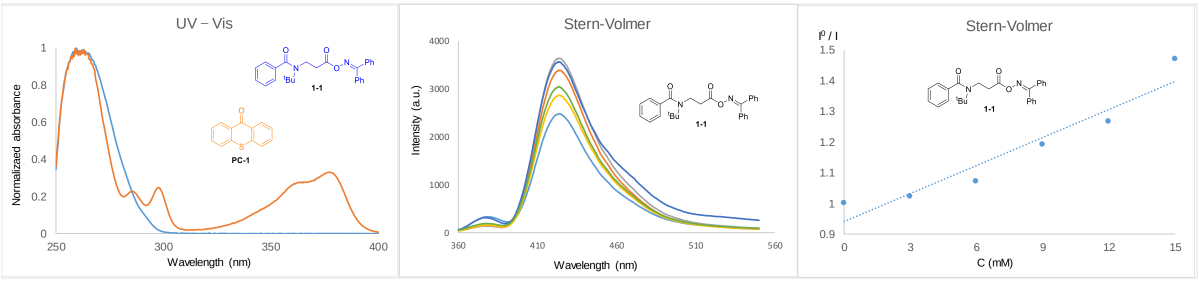

The corresponding experimental data is listed below.

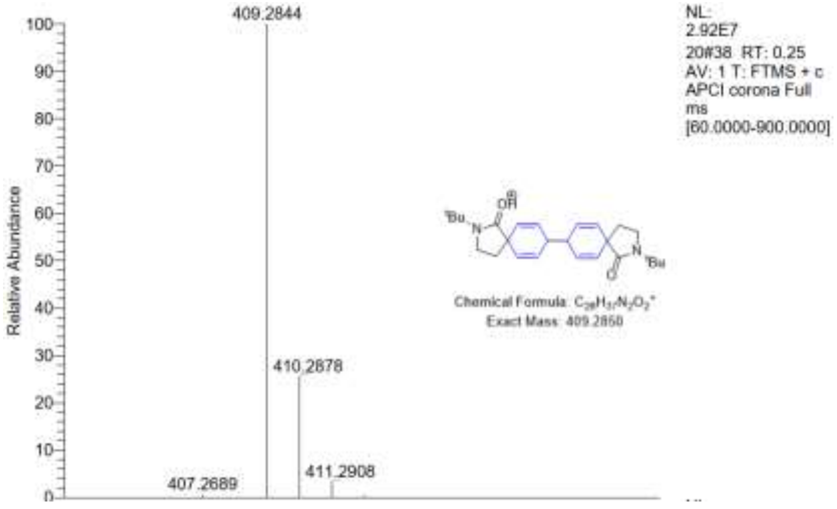

HRMS for **Dimer-1**

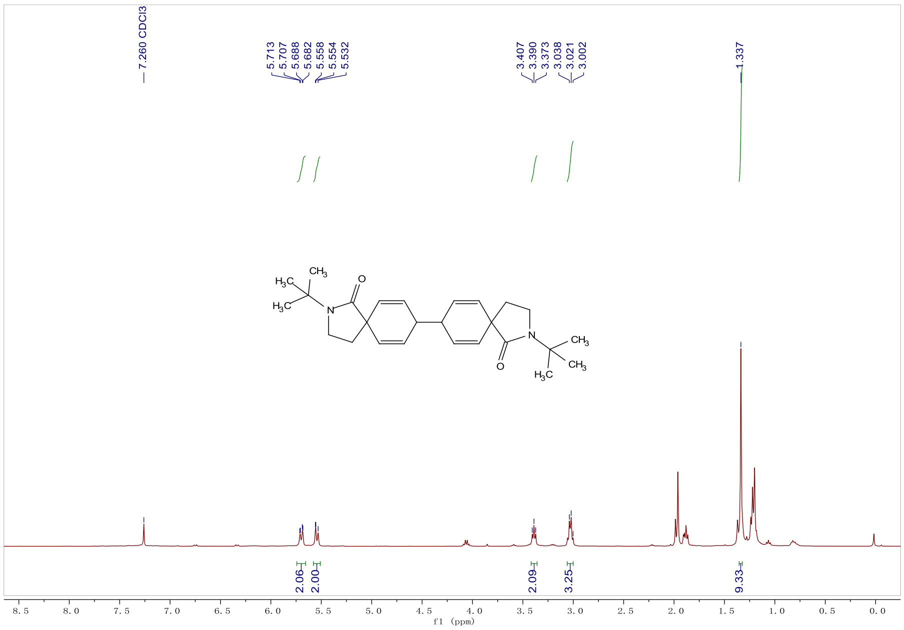

^{1}H-NMR for **Dimer-1**

NMR: ^{1}H NMR (400 MHz, Chloroform-*d*) δ 5.70 (dd, *J* = 10.1, 2.3 Hz, 2H), 5.58 – 5.51 (m, 2H), 3.39 (t, *J* = 6.8 Hz, 2H), 3.04 – 3.00 (m, 3H), 1.34 (s, 9H).

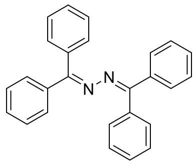

1,2-bis(diphenylmethylene)hydrazine (**dimer-2**)

The title compound was obtained as a yellow solid. This compound is known.[6]

NMR: ^{1}H NMR (400 MHz, Chloroform-*d*) δ 7.52 – 7.45 (m, 2H), 7.45 – 7.38 (m, 3H), 7.37 – 7.32 (m, 3H), 7.32 – 7.27 (m, 2H). ^{13}C NMR (101 MHz, Chloroform-*d*) δ 158.9, 138.2, 135.5, 129.6, 129.3, 128.6, 128.6, 128.0, 127.8.

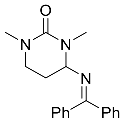

4-((diphenylmethylene)amino)-1,3-dimethyltetrahydropyrimidin-2(1*H*)-one (**DMPU-1**)

The title compound was obtained as a brown oil (34 mg, 15% yield).

NMR: ^{1}H NMR (400 MHz, Chloroform-*d*) δ 7.64 (d, *J* = 7.0 Hz, 2H), 7.54 – 7.44 (m, 3H), 7.40 (t, *J* = 7.3 Hz, 1H), 7.33 (t, *J* = 7.4 Hz, 2H), 7.16 (dd, *J* = 7.3, 2.1 Hz, 2H), 4.61 (t, *J* = 3.6 Hz, 1H), 3.89 – 3.79 (m, 1H), 3.14 – 3.08 (m, 1H), 3.03 (s, 3H), 2.72 (s, 3H), 2.17 – 2.07 (m, 1H), 1.81 – 1.73 (m, 1H). ^{13}C NMR (101 MHz, Chloroform-*d*) δ 167.8, 156.6, 138.8, 136.0, 130.4, 128.6, 128.6, 127.9, 127.3, 72.7, 44.2, 35.7, 33.4, 29.0.

VIII. Crystallographic data

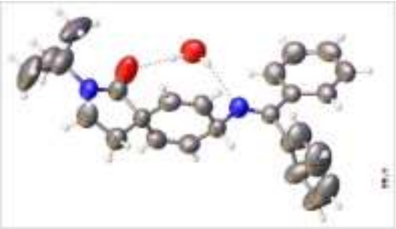

cis-**2-1** (CCDC: 2343819)

Identification code	exp_13627_autored
Empirical formula	$C_{26}H_{30}N_2O_2$
Formula weight	402.52
Temperature/K	293(2)
Crystal system	monoclinic
Space group	$P2_1/n$
a/Å	9.8065(7)
b/Å	16.1017(12)
c/Å	15.4868(15)
α/°	90
β/°	100.404(8)
γ/°	90
Volume/Å^3	2405.2(3)
Z	4
ρ_{calc}g/cm^3	1.112
μ/mm^{-1}	0.551
F(000)	864.0
Crystal size/mm^3	0.18 × 0.12 × 0.09
Radiation	Cu Kα (λ = 1.54184)
2Θ range for data collection/°	7.99 to 129.992
Index ranges	$-11 \leq h \leq 11$, $-17 \leq k \leq 18$, $-18 \leq l \leq 16$
Reflections collected	15383
Independent reflections	3943 [R_{int} = 0.1557, R_{sigma} = 0.0940]
Data/restraints/parameters	3943/0/271
Goodness-of-fit on F^2	1.040
Final R indexes [I>=2σ (I)]	R_1 = 0.1017, wR_2 = 0.2718
Final R indexes [all data]	R_1 = 0.1382, wR_2 = 0.3232
Largest diff. peak/hole / e Å^{-3}	0.35/-0.44

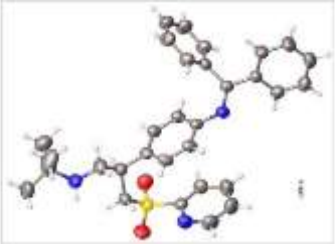

6-1 (CCDC: 2360015)

Crystal data and structure refinement

Identification code	exp_14260_auto
Empirical formula	$C_{31}H_{34}N_3O_2S$
Formula weight	512.67
Temperature/K	293(2)
Crystal system	monoclinic
Space group	$P2_1/n$
a/Å	9.0152(4)
b/Å	19.9619(10)
c/Å	15.0973(7)
α/°	90
β/°	92.014(5)
γ/°	90
Volume/Å^3	2715.2(2)
Z	4
ρ_{calc}g/cm^3	1.254
μ/mm^{-1}	0.152
F(000)	1092.0
Crystal size/mm^3	0.25 × 0.12 × 0.11
Radiation	Mo Kα (λ = 0.71073)
2Θ range for data collection/°	4.894 to 53.99
Index ranges	$-11 \leq h \leq 11, -25 \leq k \leq 25, -19 \leq l \leq 19$
Reflections collected	21642
Independent reflections	5895 [R_{int} = 0.0439, R_{sigma} = 0.0502]
Data/restraints/parameters	5895/0/337
Goodness-of-fit on F^2	1.032
Final R indexes [I>=2σ (I)]	R_1 = 0.0545, wR_2 = 0.1173
Final R indexes [all data]	R_1 = 0.0907, wR_2 = 0.1370
Largest diff. peak/hole / e Å^{-3}	0.22/-0.34

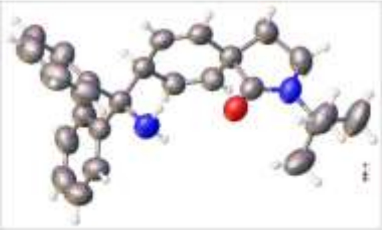

cis-**3-1**（CCDC：2343822）

Identification code	exp_13658_auto
Empirical formula	$C_{26}H_{30}N_2O$
Formula weight	386.52
Temperature/K	293(2)
Crystal system	monoclinic
Space group	$P2_1/n$
a/Å	11.3911(5)
b/Å	9.5082(4)
c/Å	20.4932(10)
α/°	90
β/°	99.690(5)
γ/°	90
Volume/Å^3	2187.93(17)
Z	4
ρ_{calc}g/cm^3	1.173
μ/mm^{-1}	0.550
F(000)	832.0
Crystal size/mm^3	0.25 × 0.17 × 0.12
Radiation	Cu Kα (λ = 1.54184)
2Θ range for data collection/°	8.34 to 132.996
Index ranges	$-13 \le h \le 13$, $-8 \le k \le 11$, $-24 \le l \le 24$
Reflections collected	14319
Independent reflections	3836 [R_{int} = 0.0610, R_{sigma} = 0.0531]
Data/restraints/parameters	3836/0/272
Goodness-of-fit on F^2	1.024
Final R indexes [I>=2σ (I)]	R_1 = 0.0653, wR_2 = 0.1592
Final R indexes [all data]	R_1 = 0.1015, wR_2 = 0.2004
Largest diff. peak/hole / e Å^{-3}	0.17/-0.22

IX. References

(1) Scanlan E. M.; Walton J. C. Preparation of oxime oxalate amides and their use in free-radical mediated syntheses of lactams. *Chem. Commun.* **2002**, (18), 2086-2087.

(2) Portela-Cubillo F.; Scanlan E. M.; Scott J. S.; Walton J. C. From dioxime oxalates to dihydropyrroles and phenanthridines via iminyl radicals. *Chem. Commun.* **2008**, 4189-4191.

(3) Cai J.; Zeng G.; Jiang K.; Luo H.; Yin B. Intramolecular cobalt/visible light cocatalyzed reductive coupling of unactivated arenes with unactivated alkenes. *Org. Lett.* **2024**, *26* (1), 327-331.

(4) Patra T.; Mukherjee S.; Ma J.; Strieth-Kalthoff F.; Glorius F. Visible-light-photosensitized aryl and alkyl decarboxylative functionalization reactions. *Angew. Chem. Int. Ed.* **2019**, *58* (31), 10514-10520.

(5) Qi X.-K.; Zheng M.-J.; Yang C.; Zhao Y.; Guo L.; Xia W. Metal-free amino(hetero)arylation and aminosulfonylation of alkenes enabled by photoinduced energy transfer. *J. Am. Chem. Soc.* **2023**, *145* (30), 16630-16641.

(6) Xu Y.; Wang B.; Wang J.; Zhou X.; Chen J.; Guo X.; Deng G.-J.; Shao W. Regioselective synthesis of unsymmetrical vicinal diamines via azidoimination of alkenes with $TMSN_3$ and ketimines. *Org. Lett.* **2023**, *25* (48), 8716-8721.

X. NMR Spectra

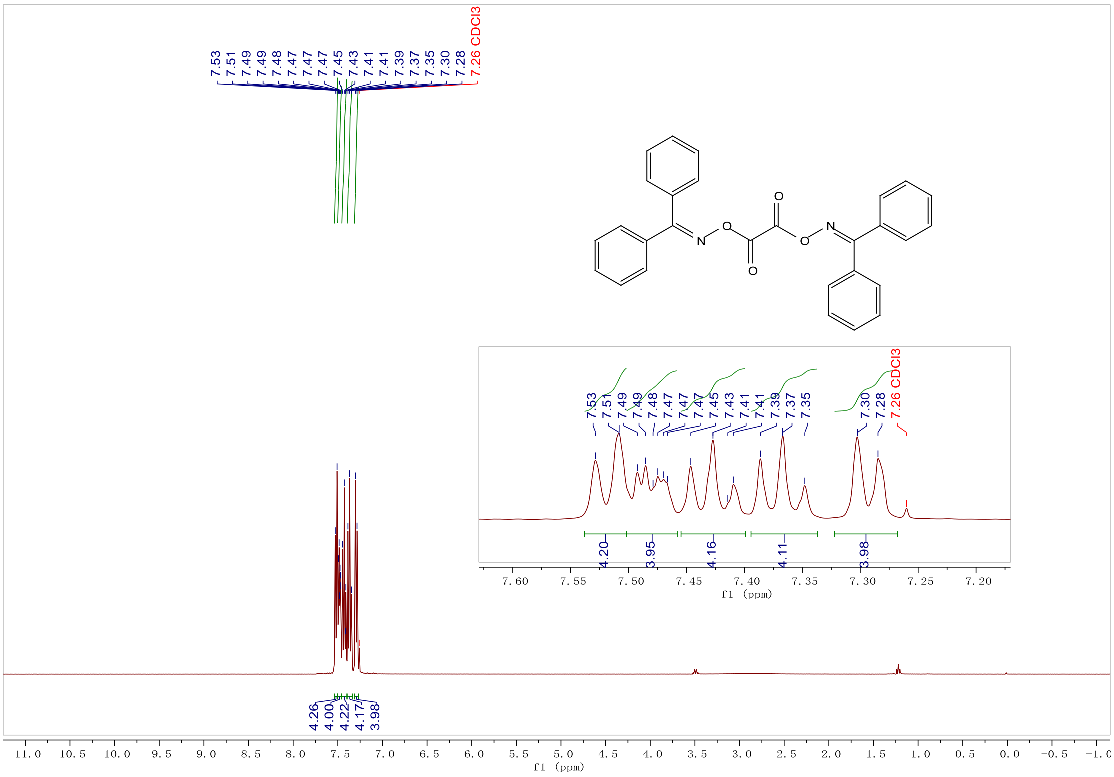

^{1}H NMR of **A-1**

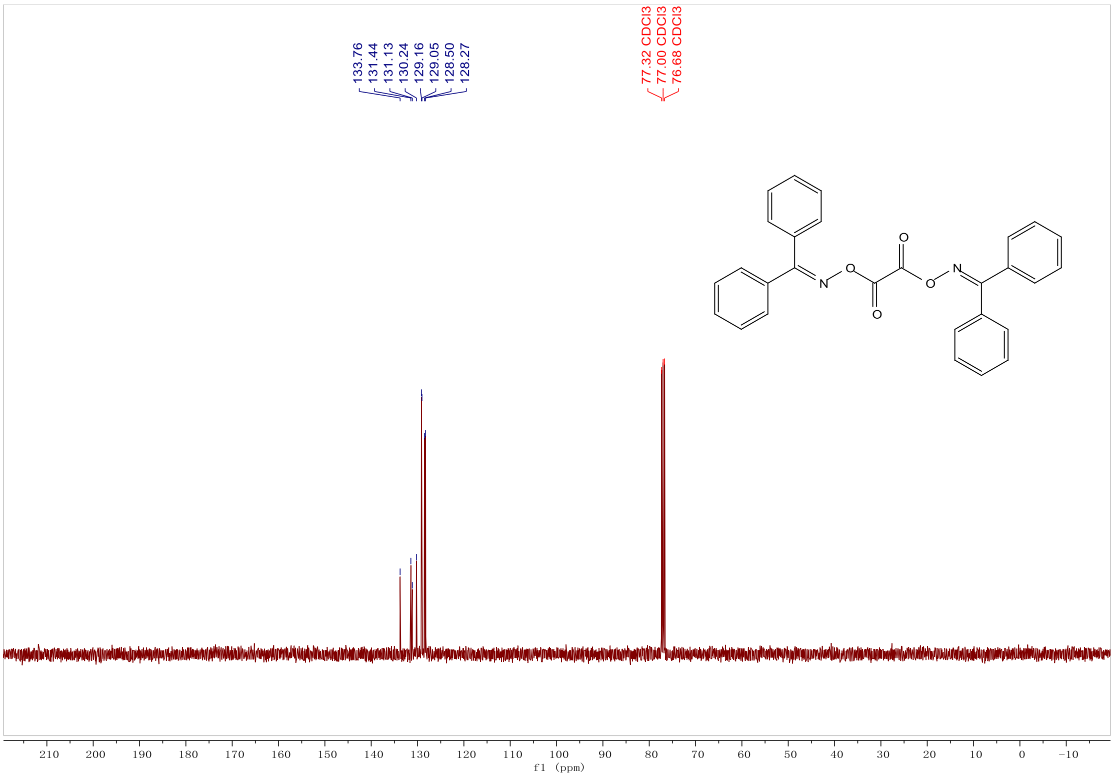

^{13}C NMR of **A-1**

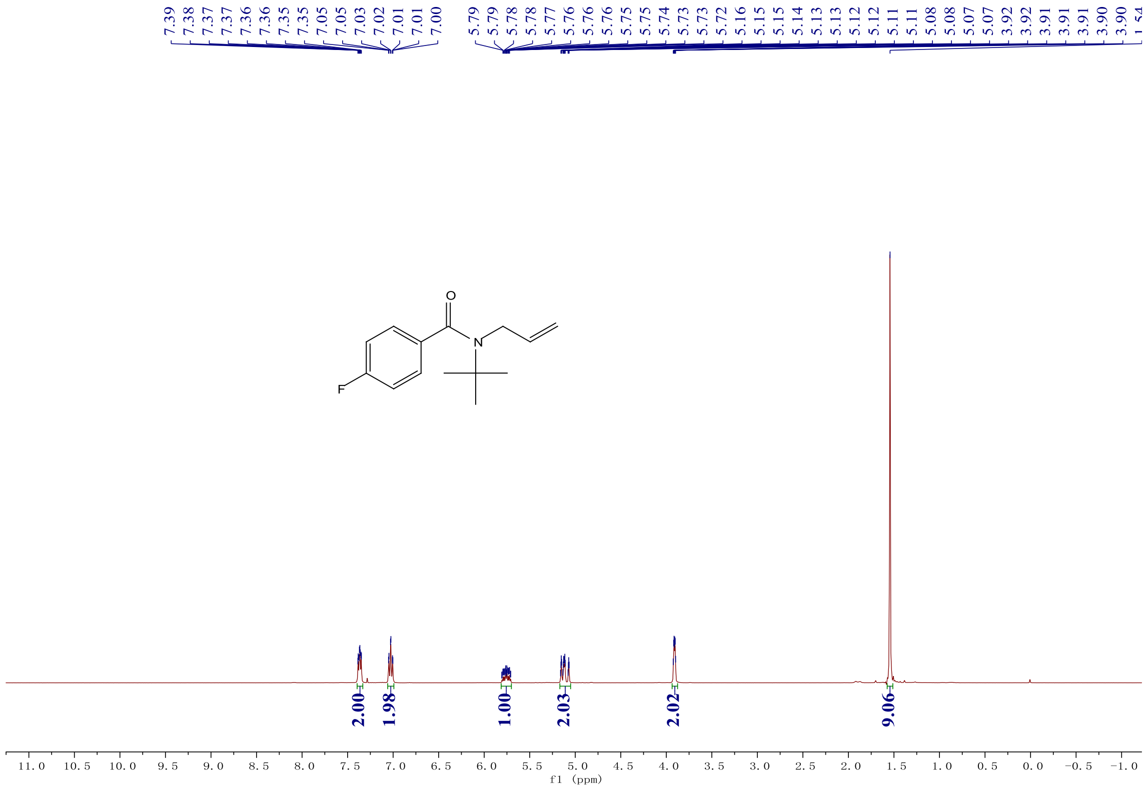

^{1}H-NMR for **4-1**

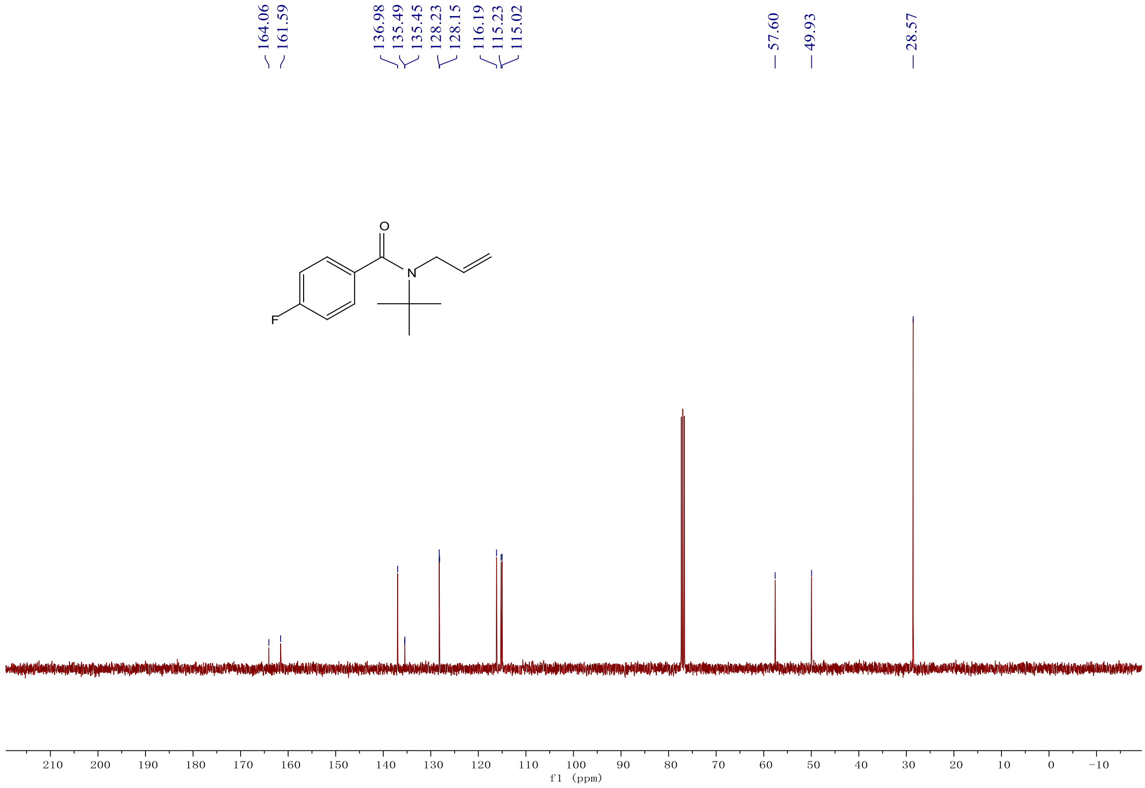

^{13}C−NMR for **4-1**

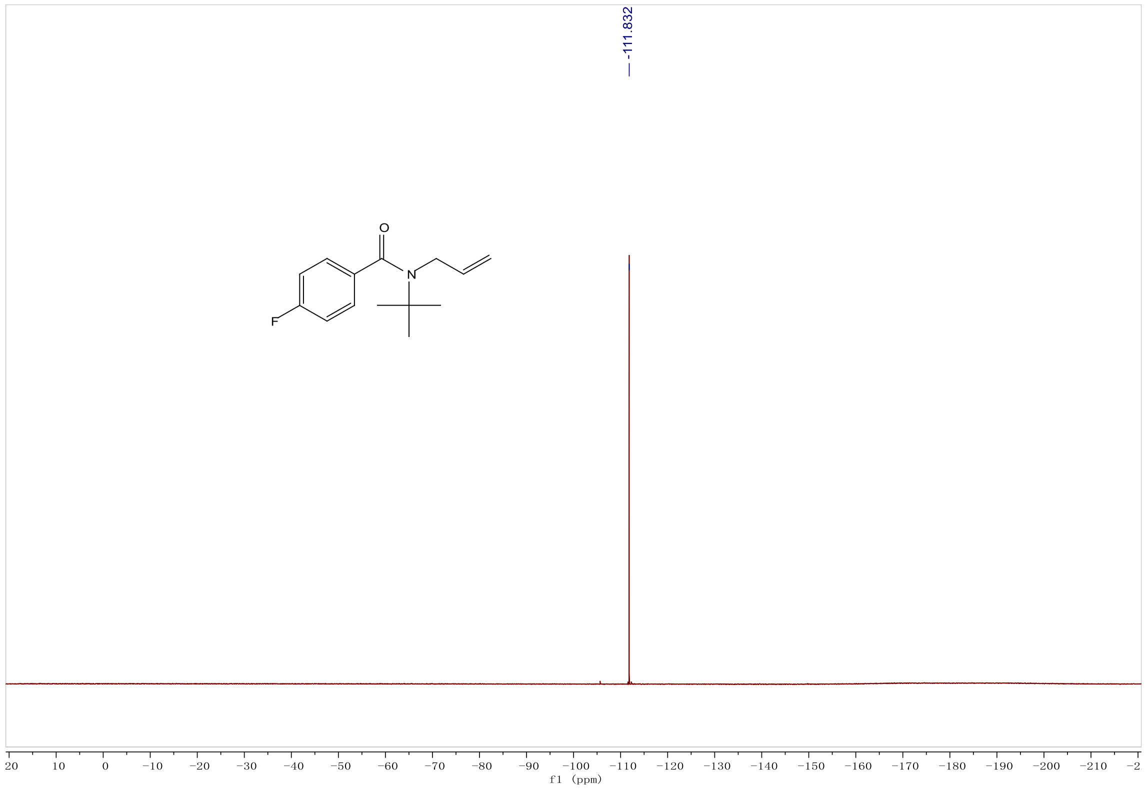

^{19}F-NMR for **4-1**

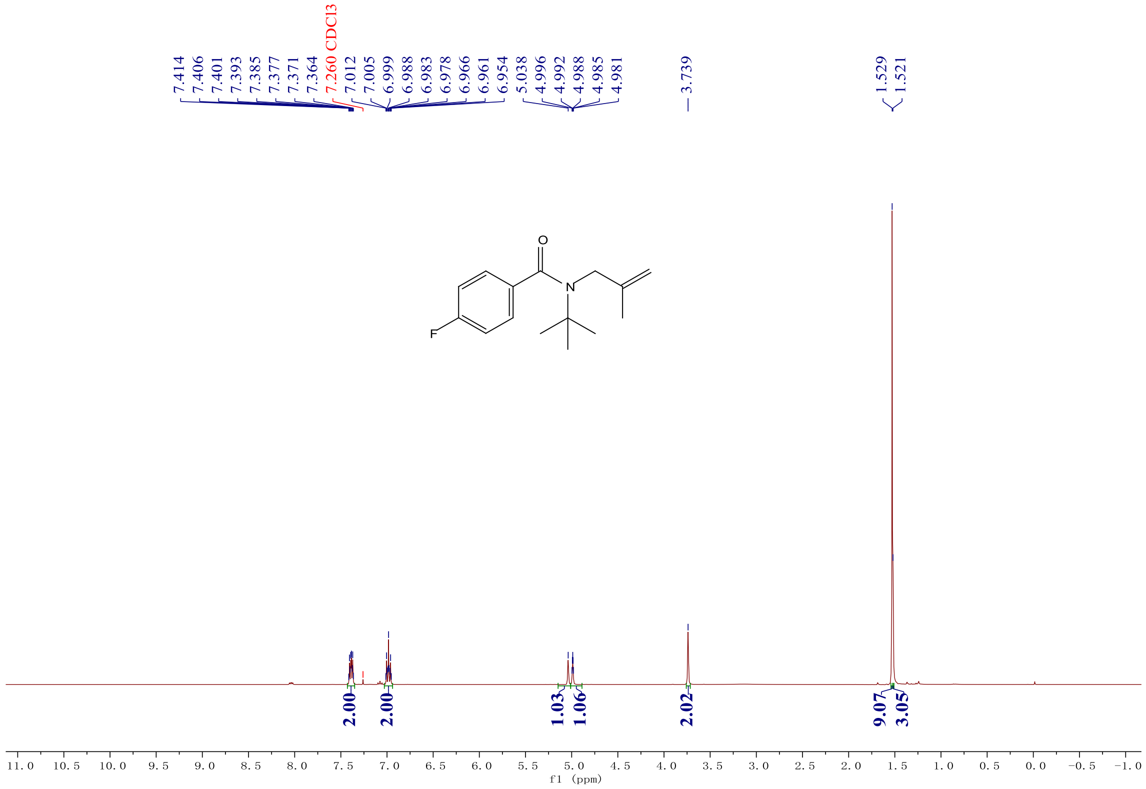

^{1}H-NMR for **4-2**

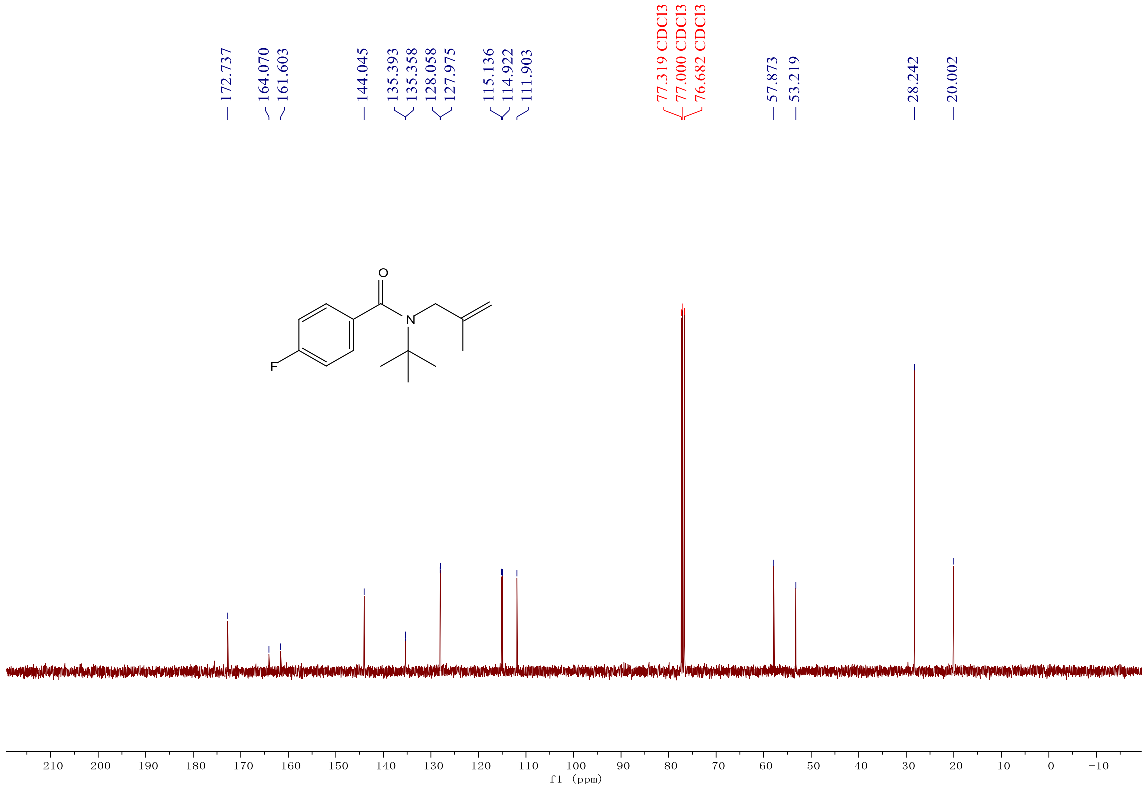

^{13}C−NMR for **4-2**

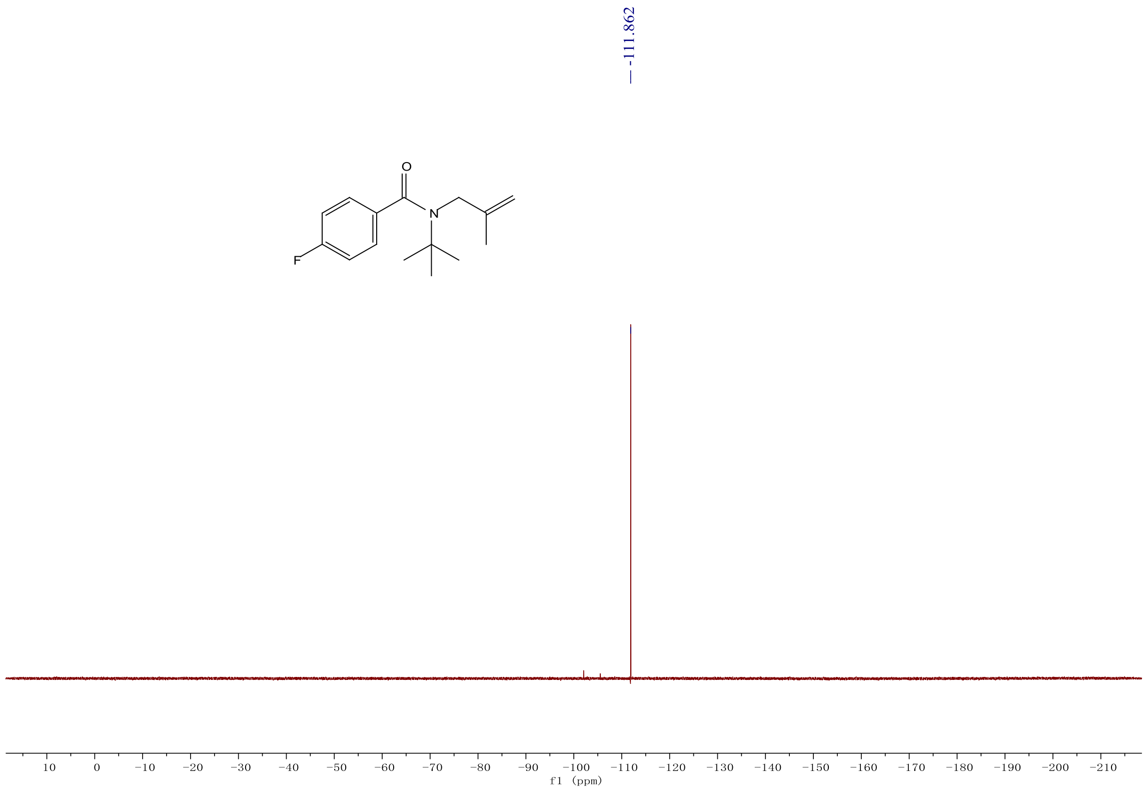

^{19}F-NMR for **4-2**

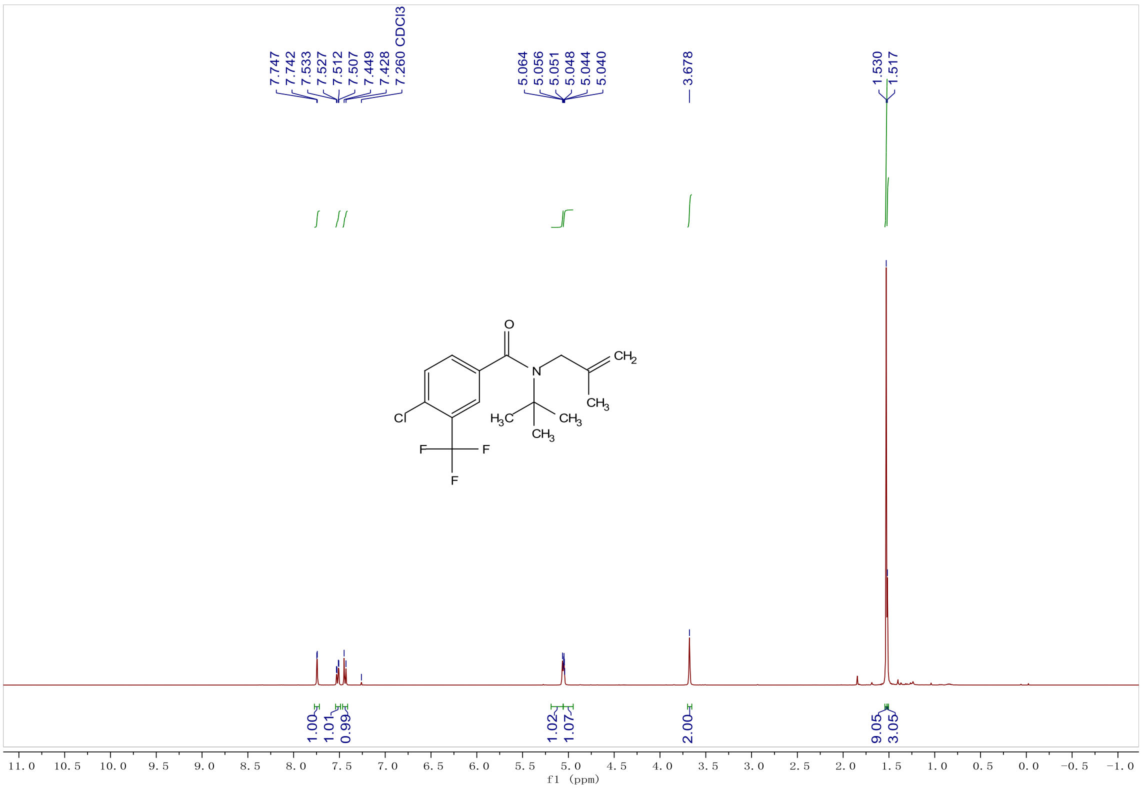

^{1}H-NMR for **4-3**

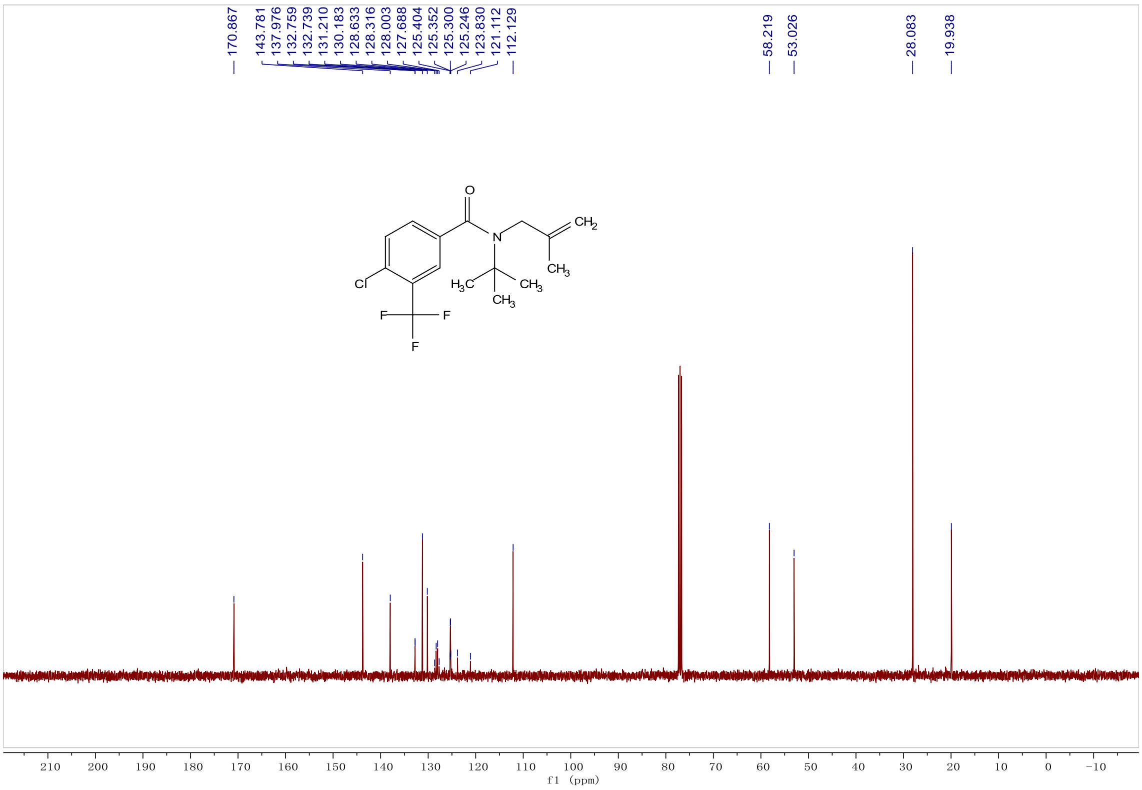

^{13}C−NMR for **4-3**

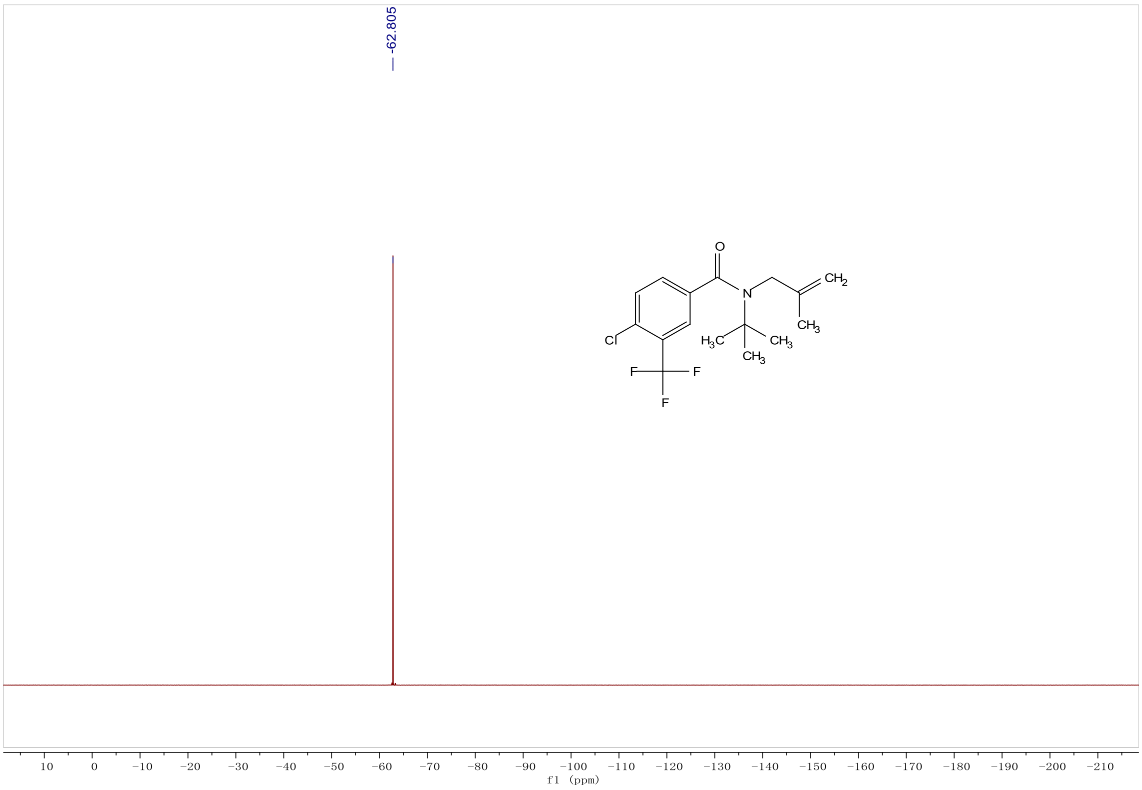

^{19}C−NMR for **4-3**

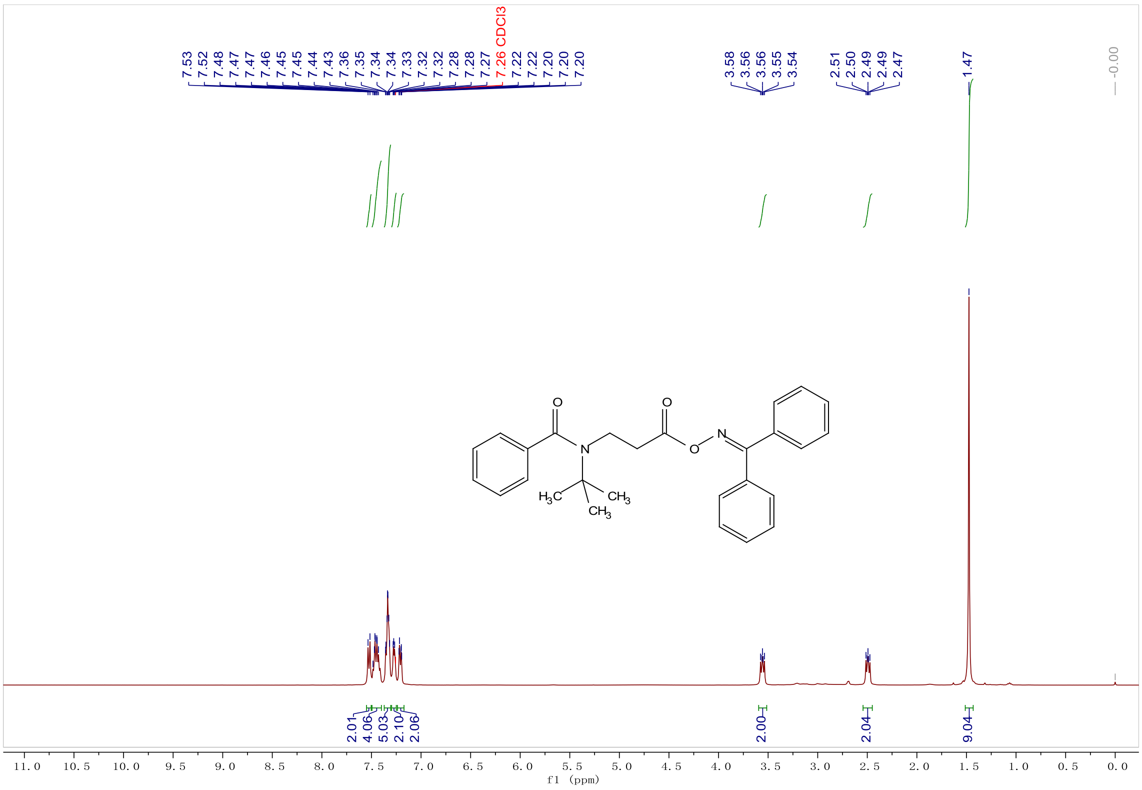

^{1}H-NMR for **1-1**

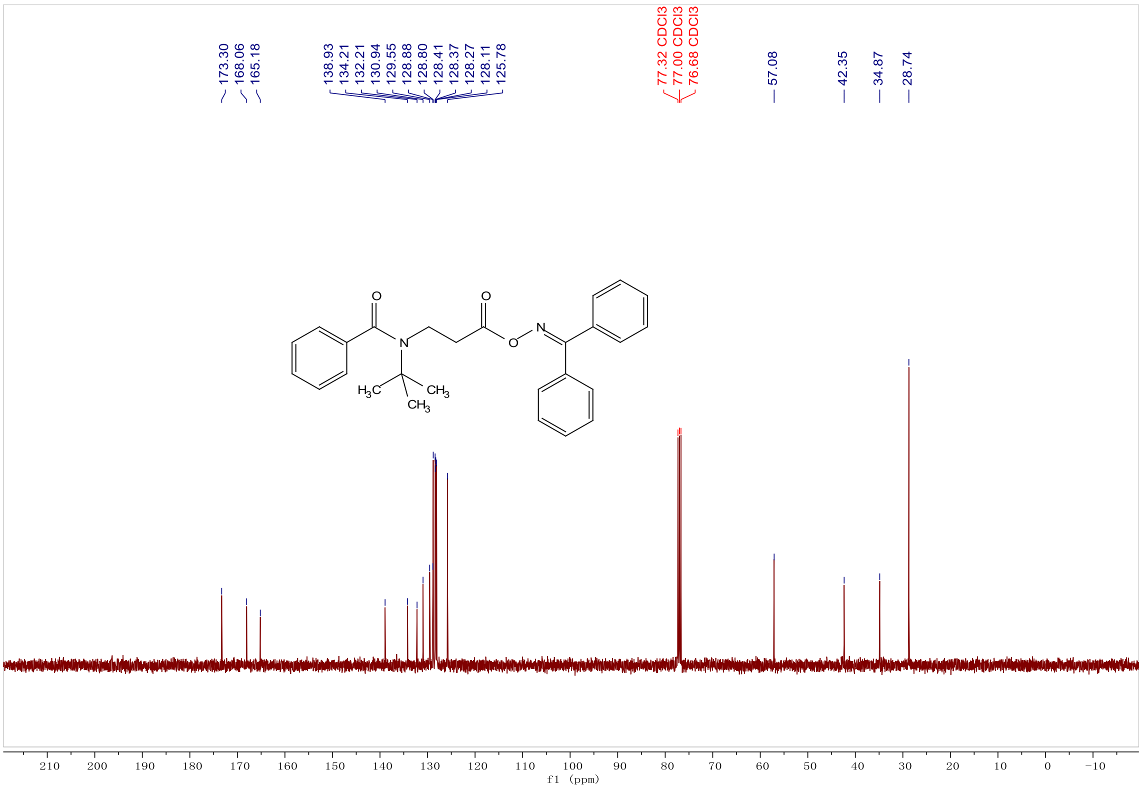

$^{13}C-NMR$ for **1-1**

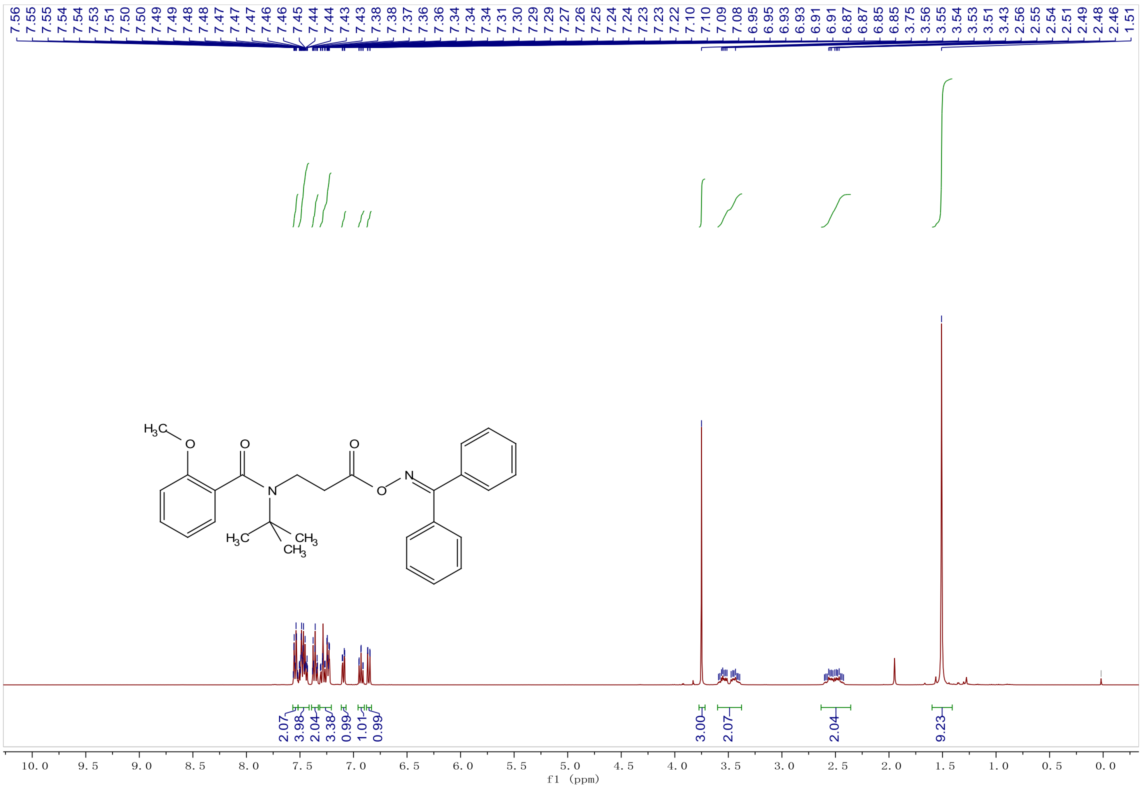

$^{1}H-NMR$ for **1-2**

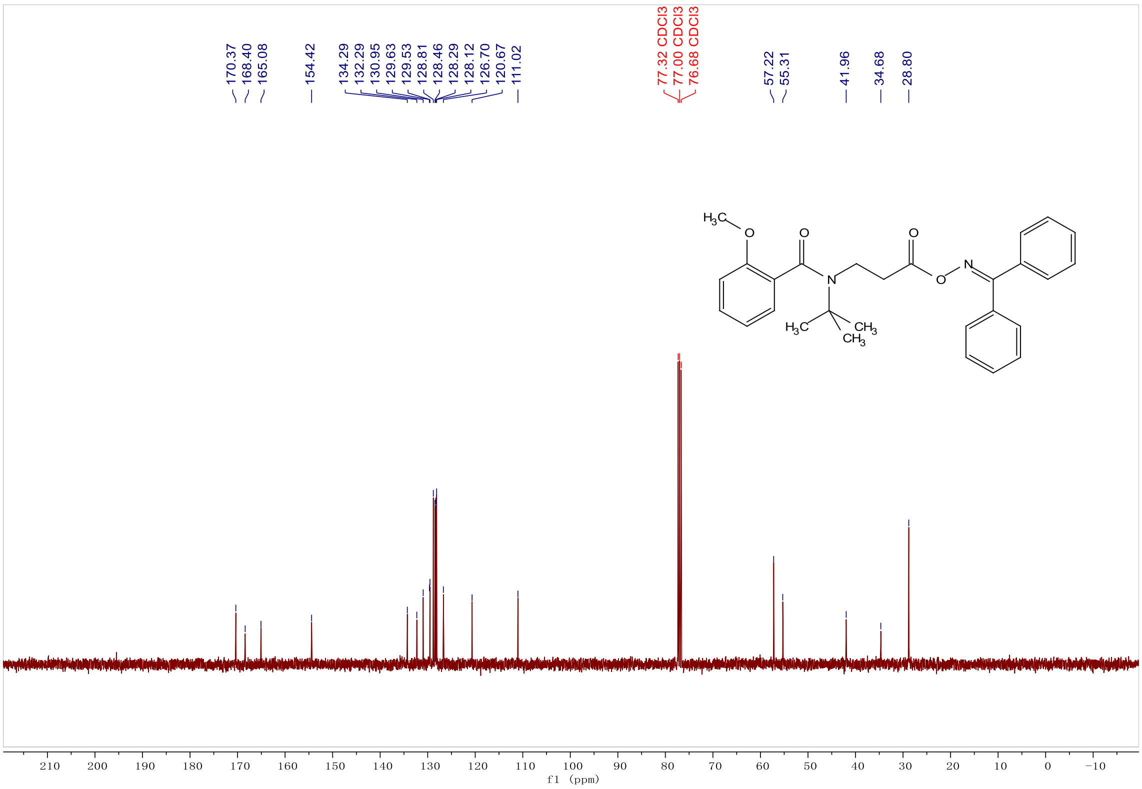

^{13}C−NMR for **1-2**

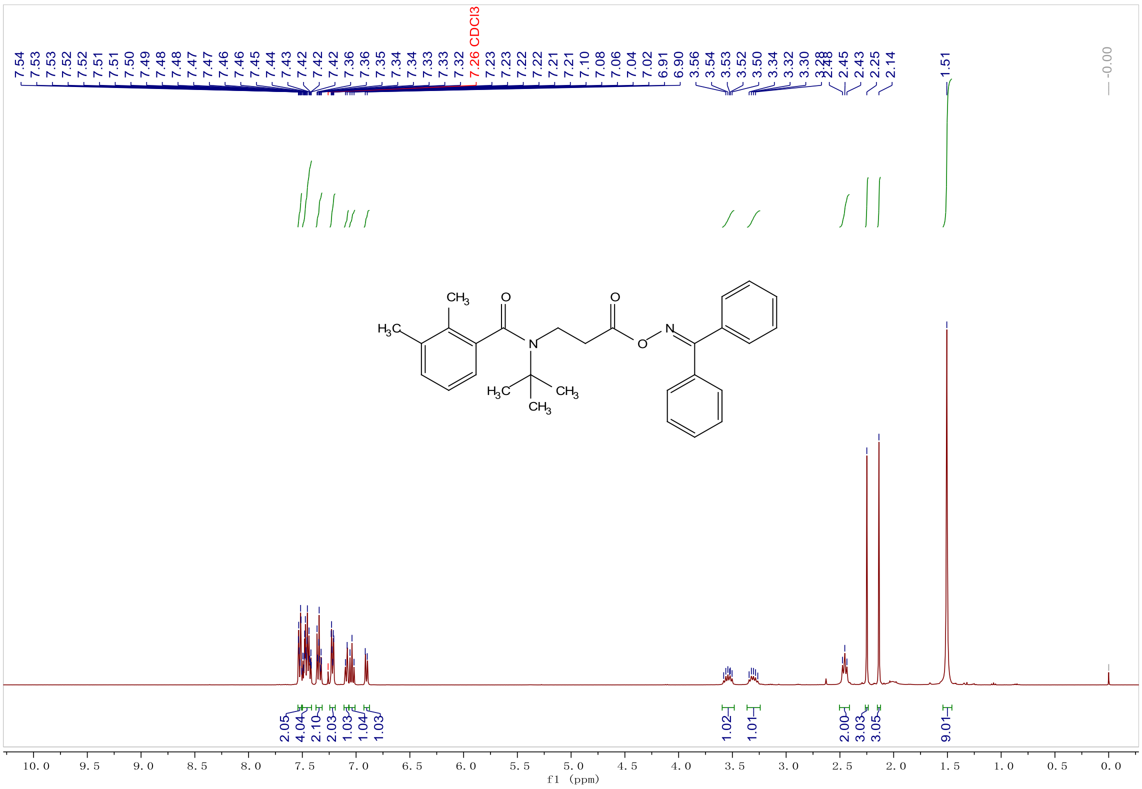

^{1}H-NMR for **1-3**

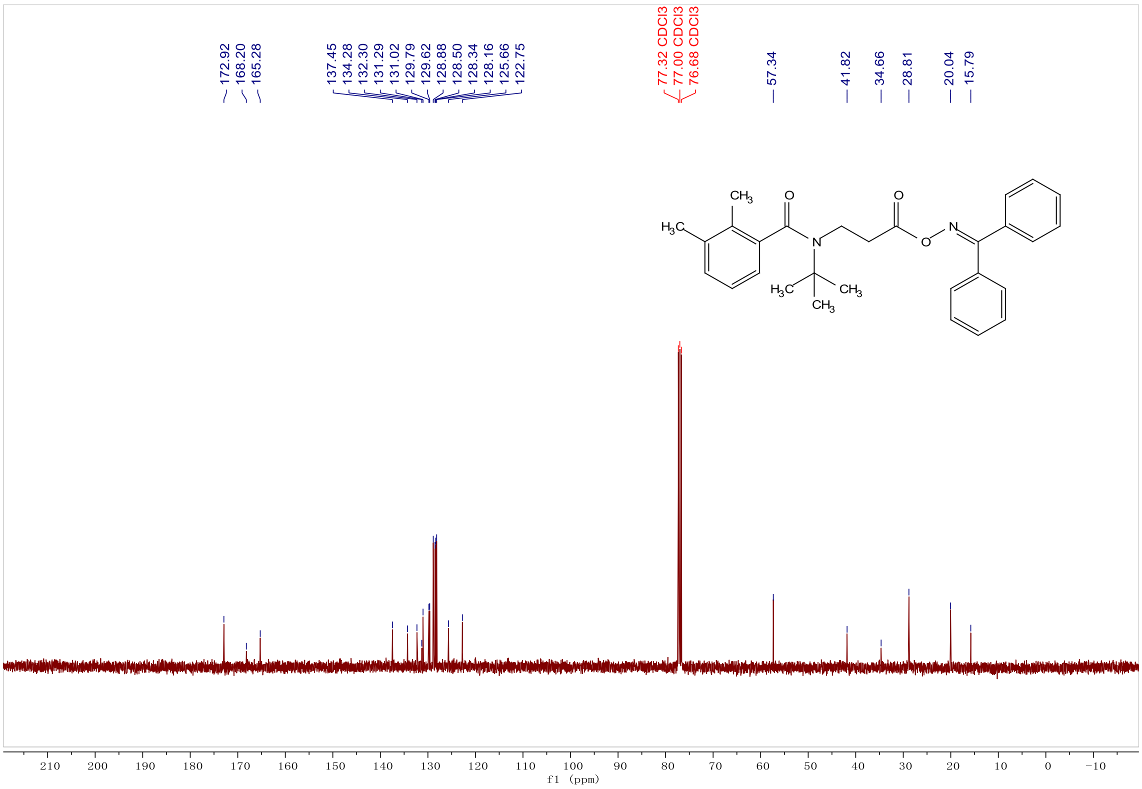

^{13}C−NMR for **1-3**

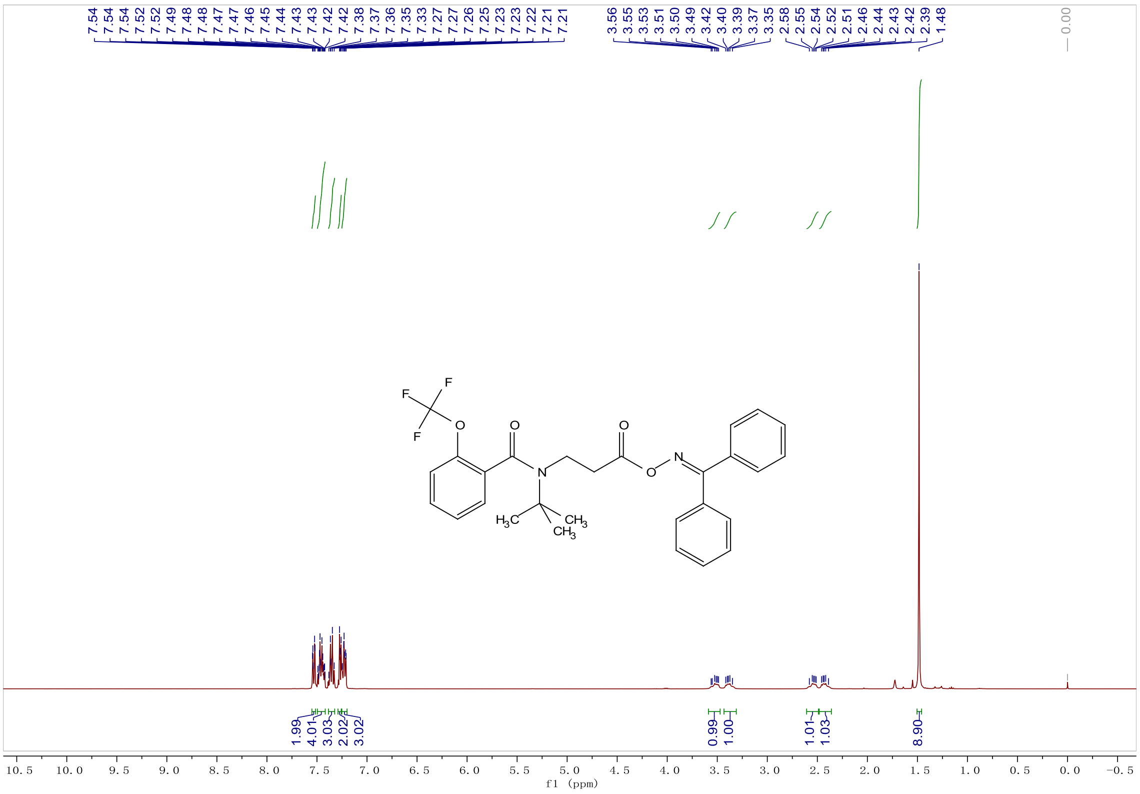

^{1}H-NMR for **1-4**

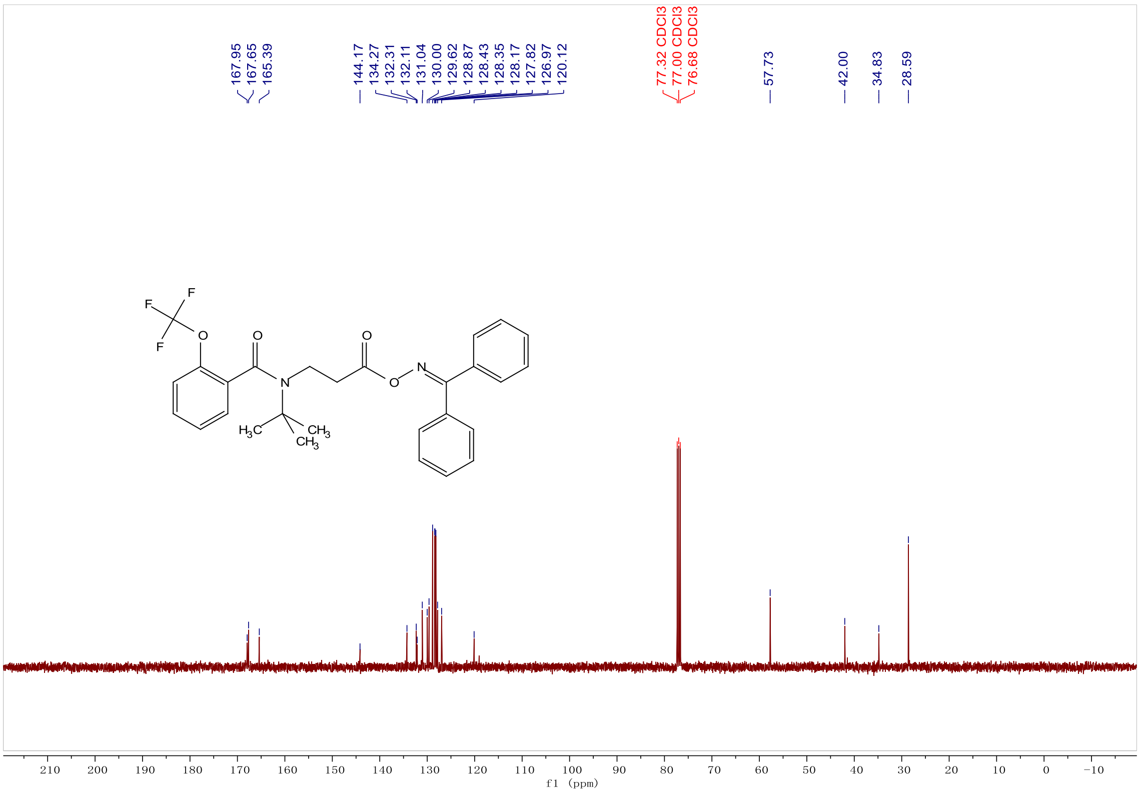

^{13}C−NMR for **1-4**

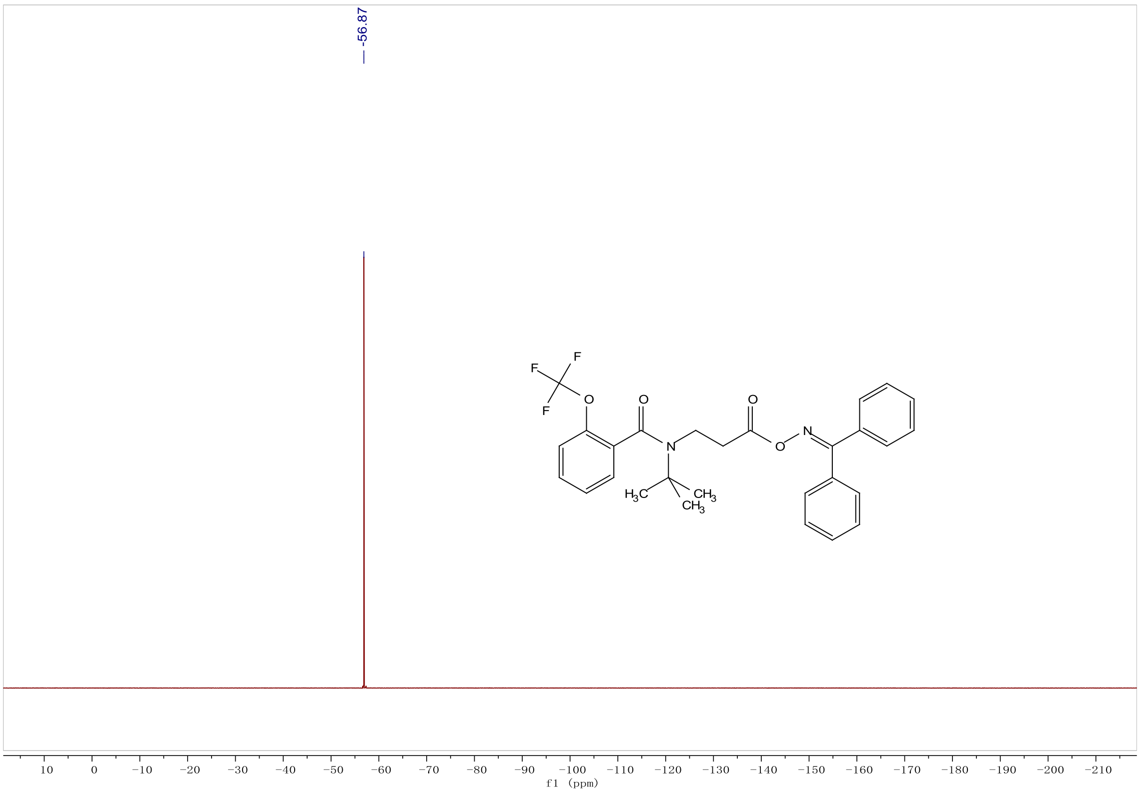

^{19}F-NMR for **1-4**

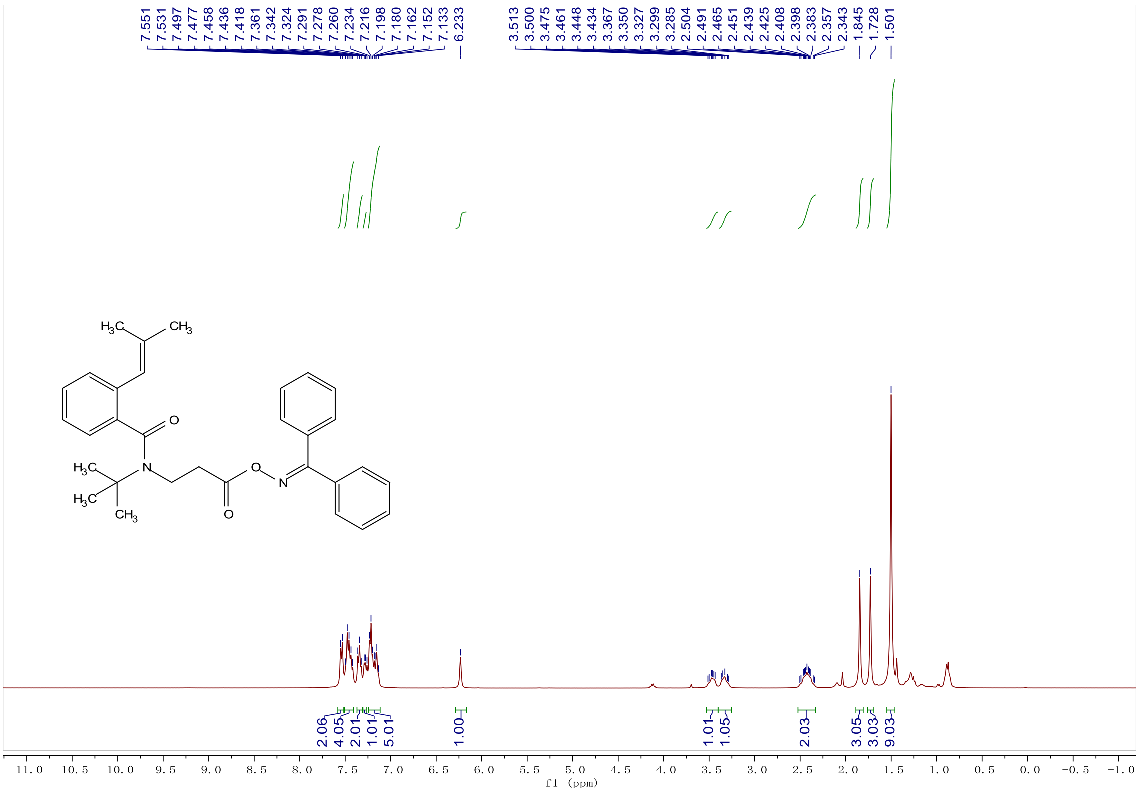

^{1}H-NMR for **1-5**

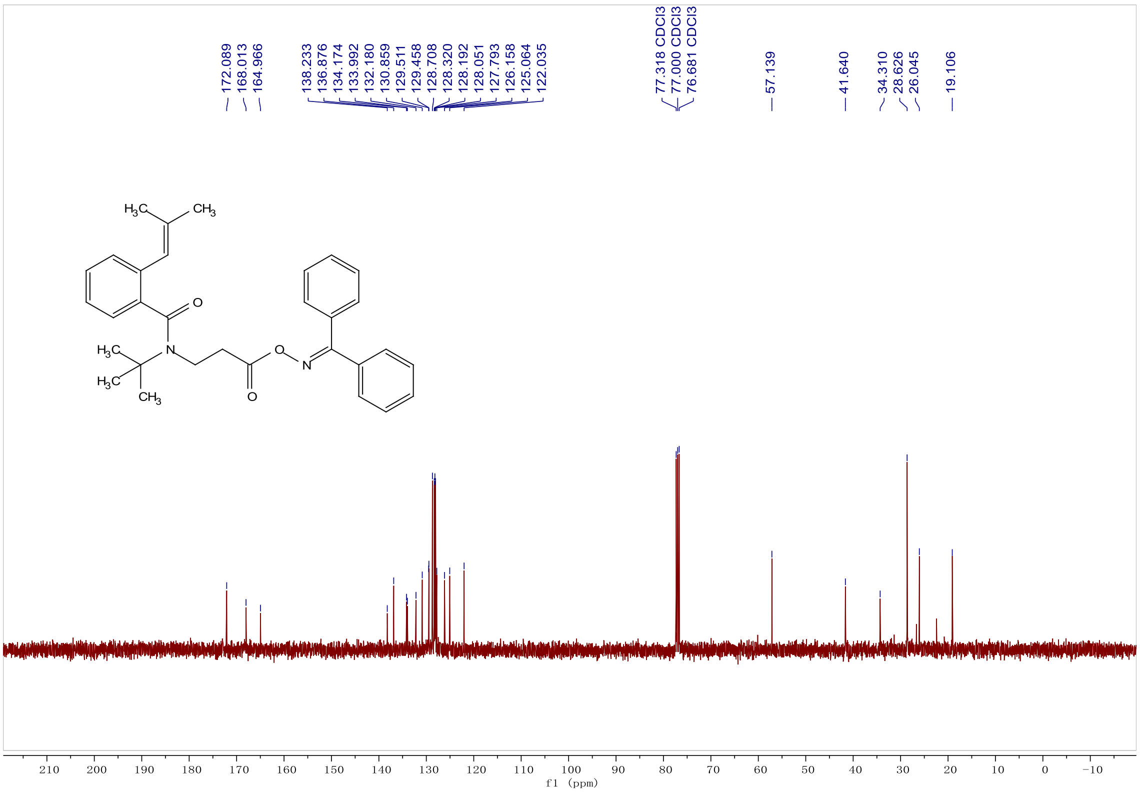

^{13}C−NMR for **1-5**

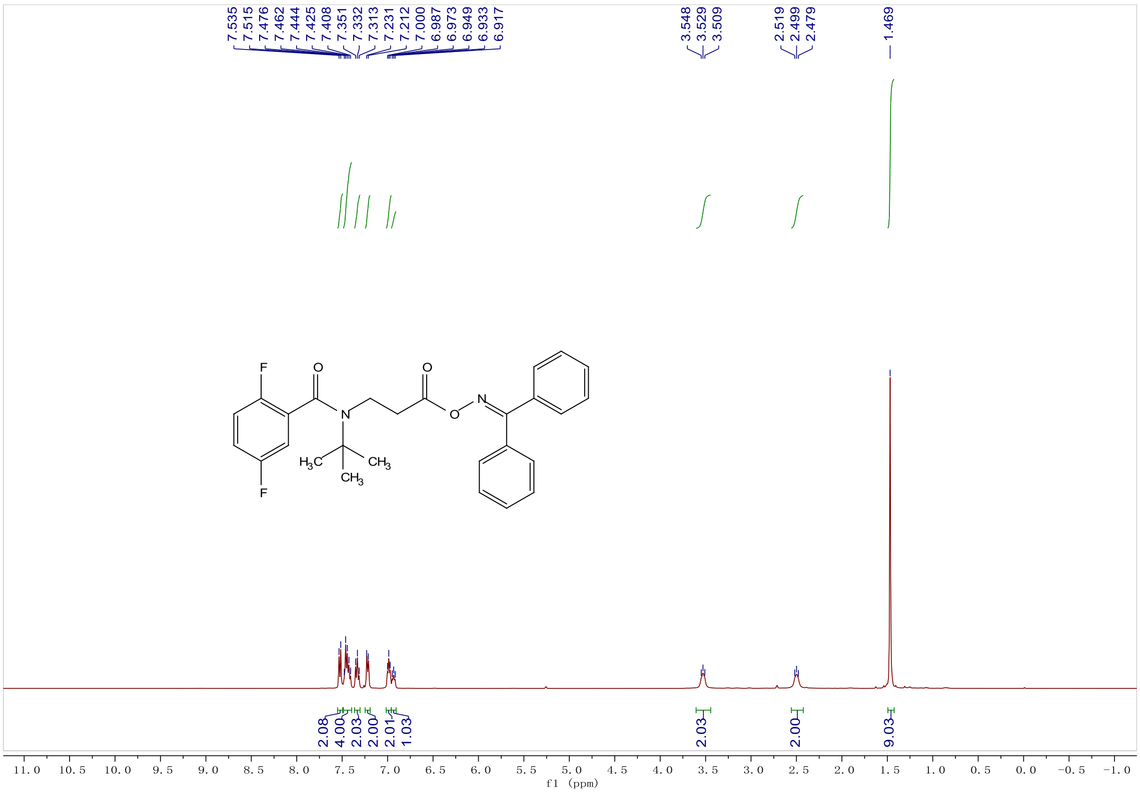

^{1}H-NMR for **1-6**

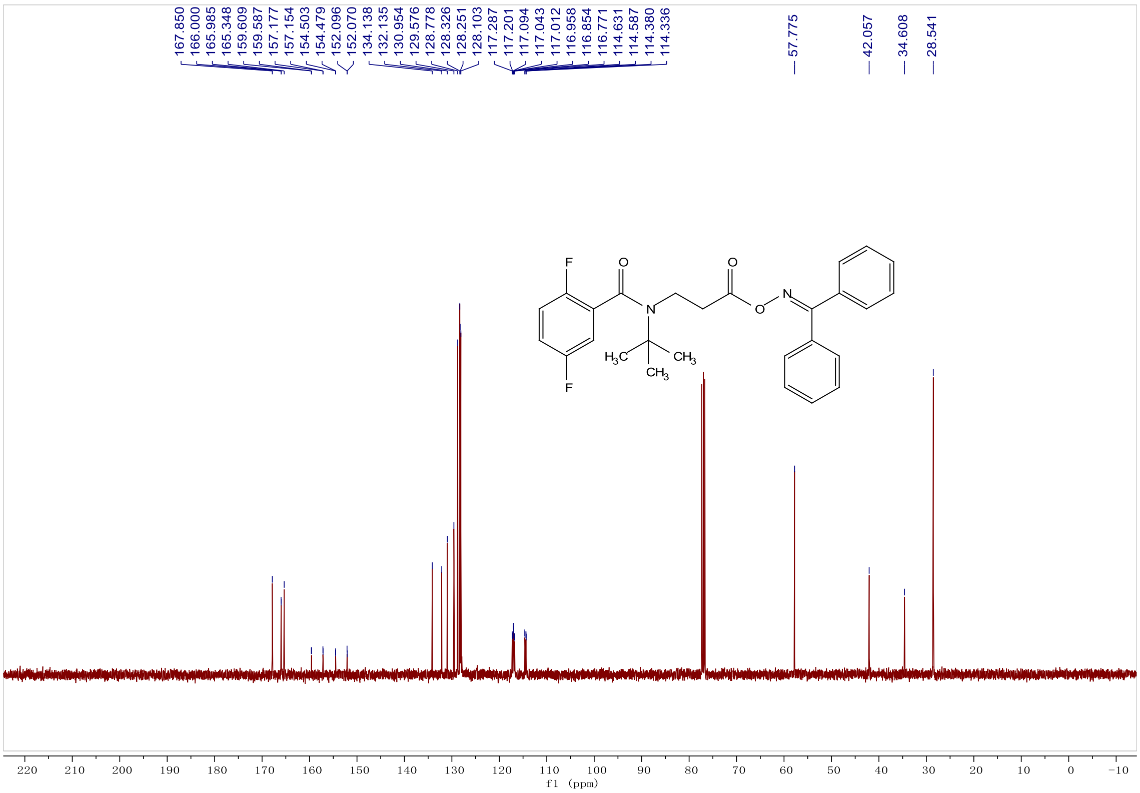

^{13}C−NMR for **1-6**

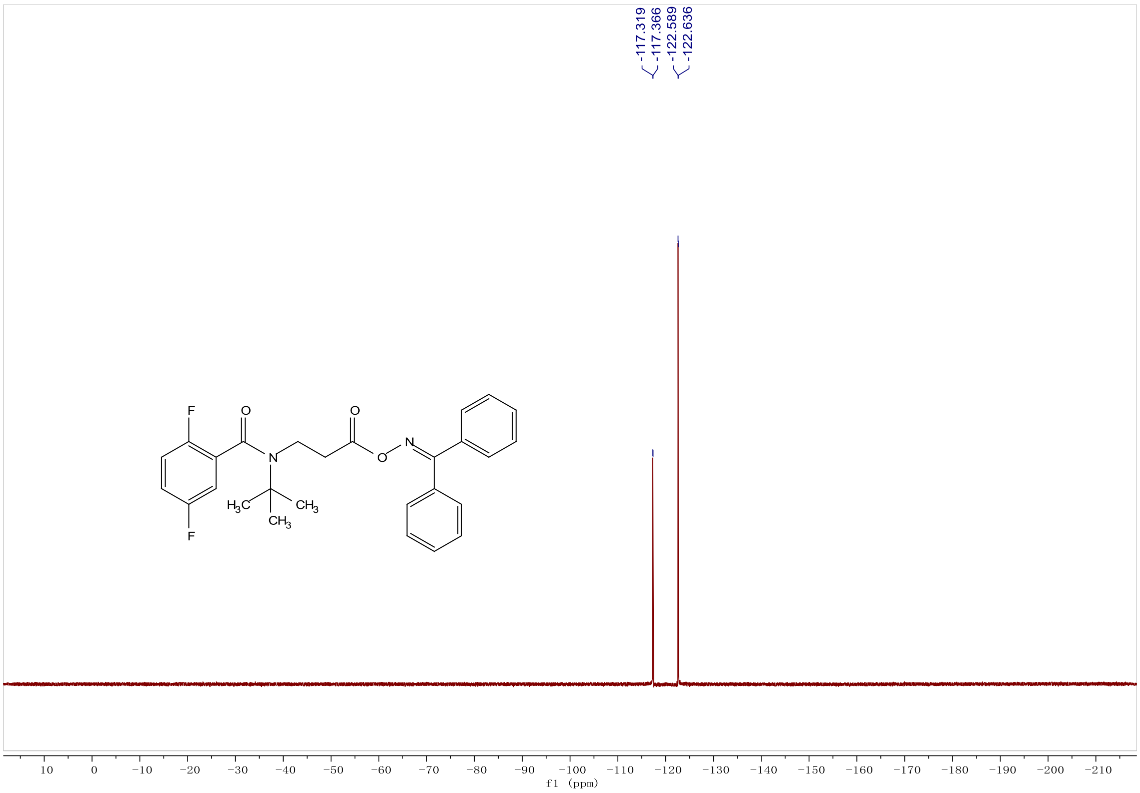

^{19}F-NMR for **1-6**

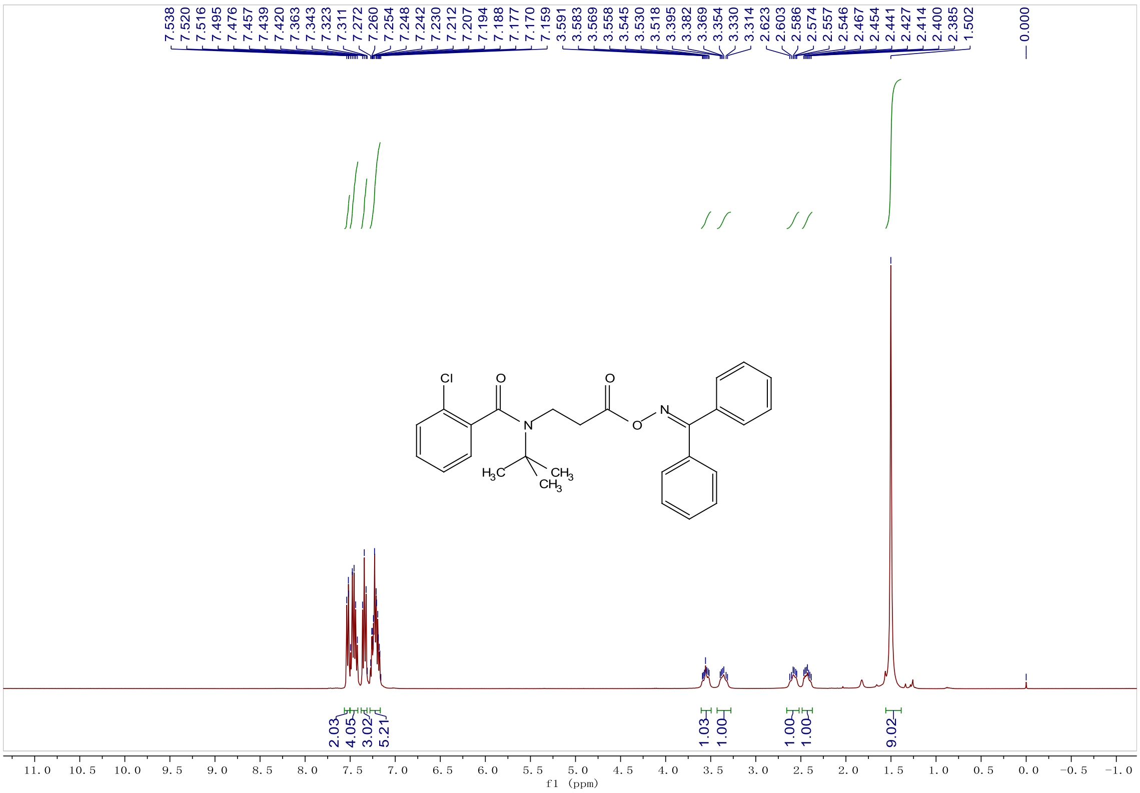

^{1}H-NMR for **1-7**

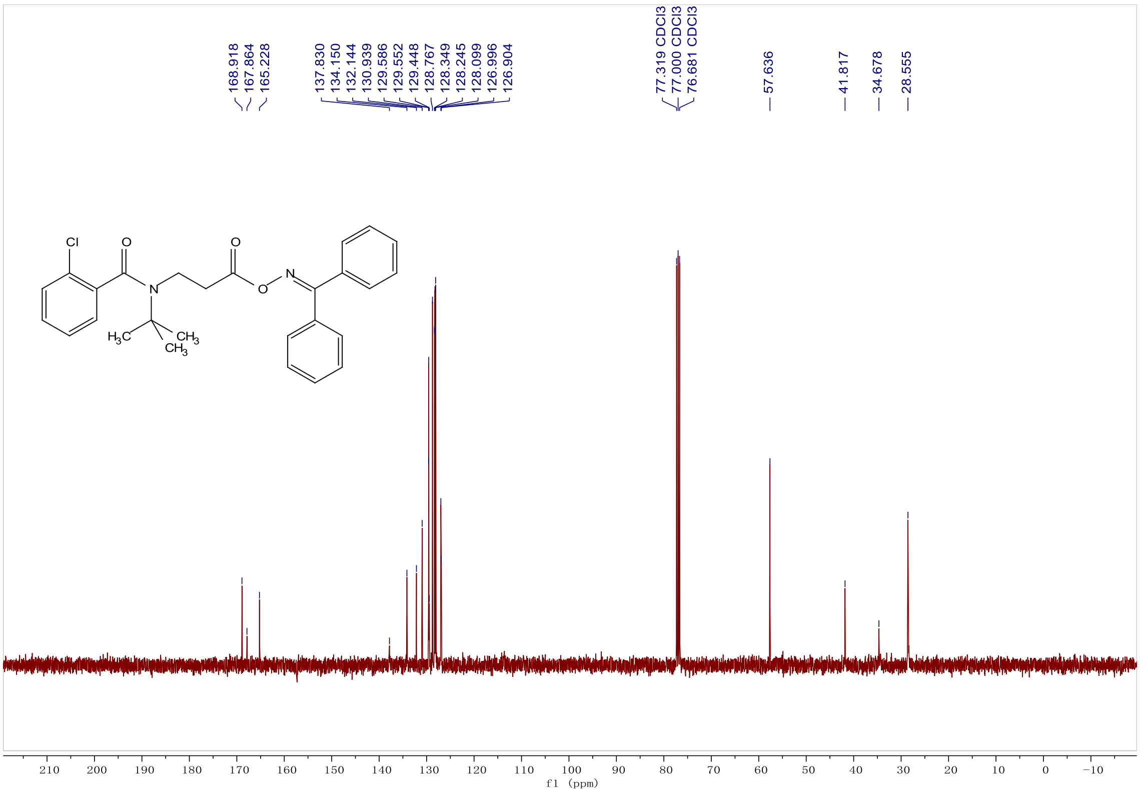

^{13}C−NMR for **1-7**

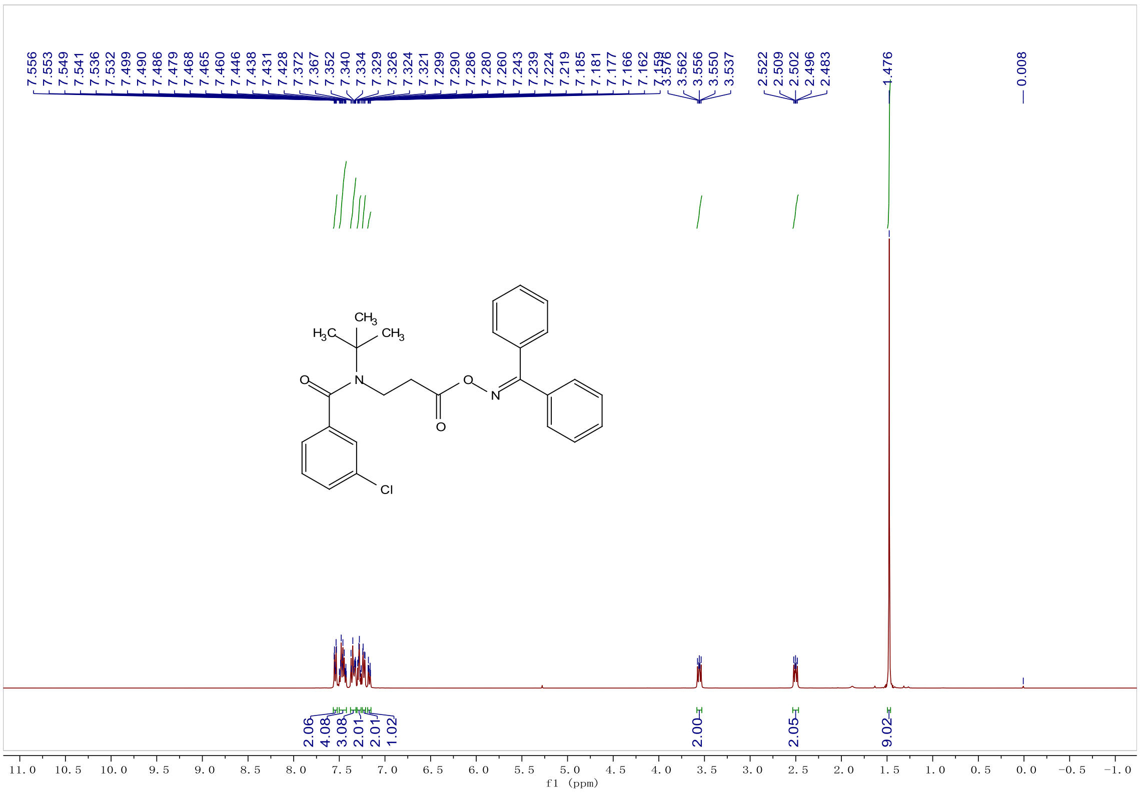

^{1}H-NMR for **1-8**

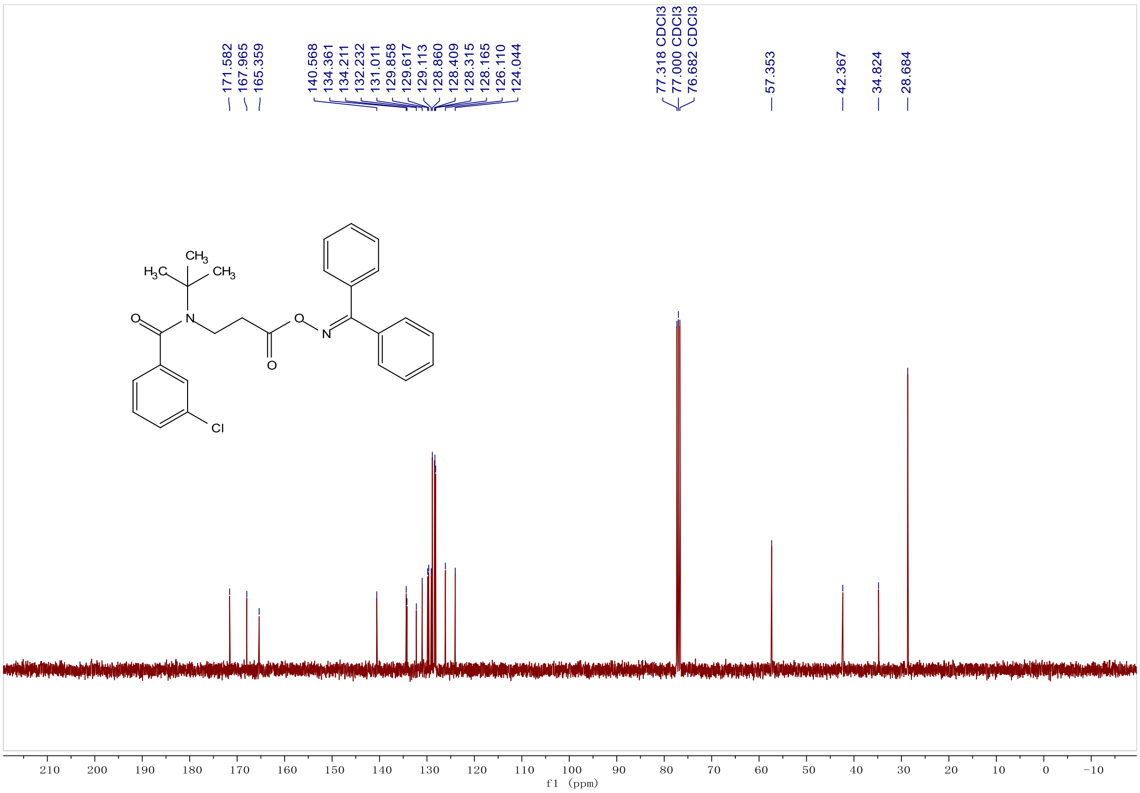

^{13}C−NMR for **1-8**

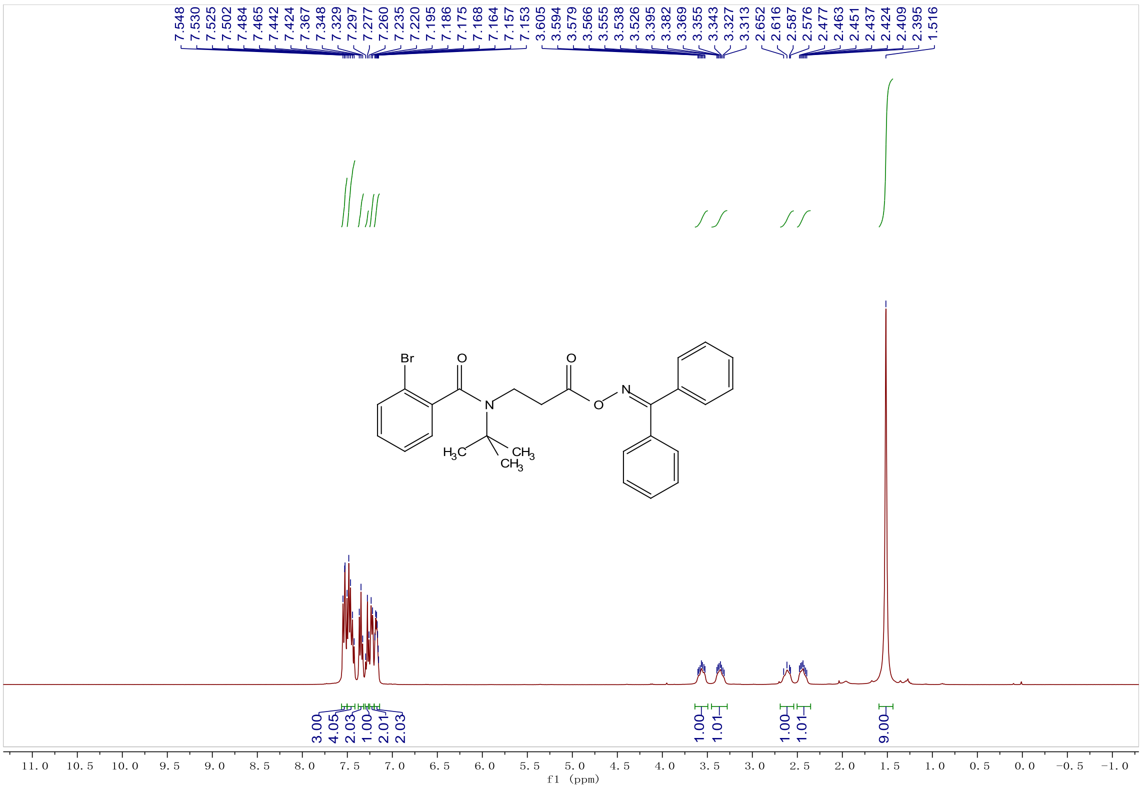

^{1}H-NMR for **1-9**

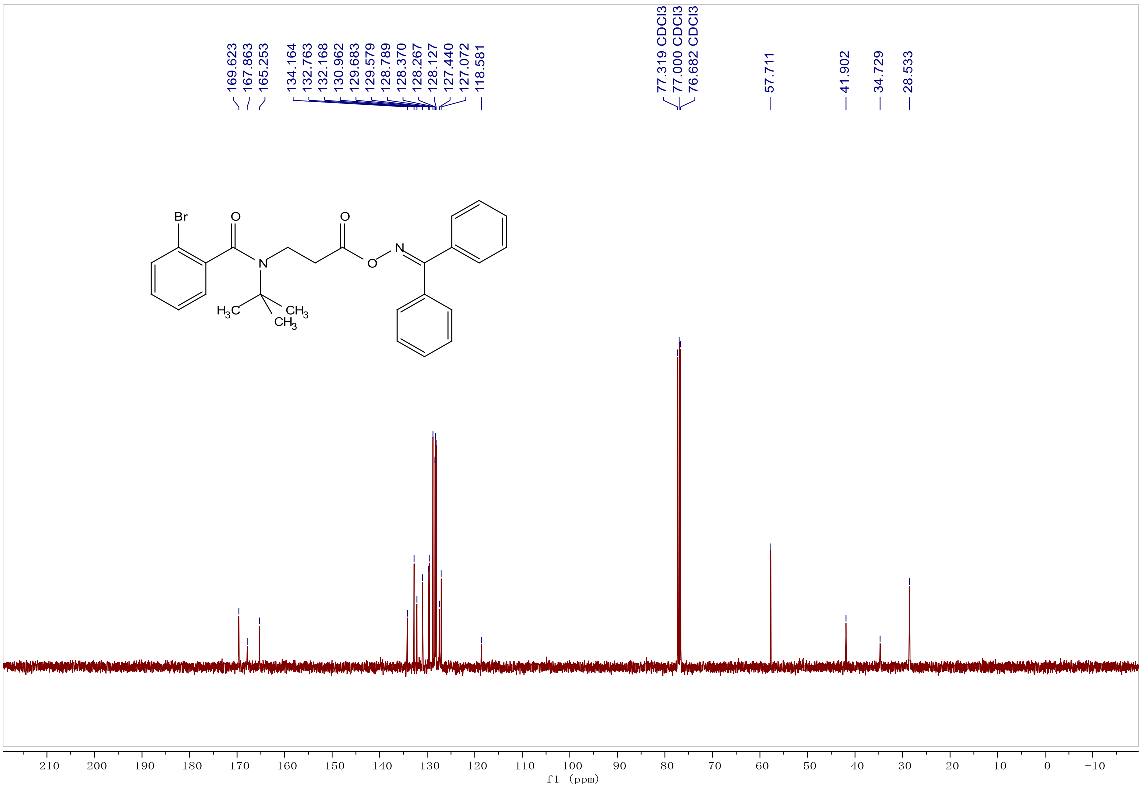

^{13}C−NMR for **1-9**

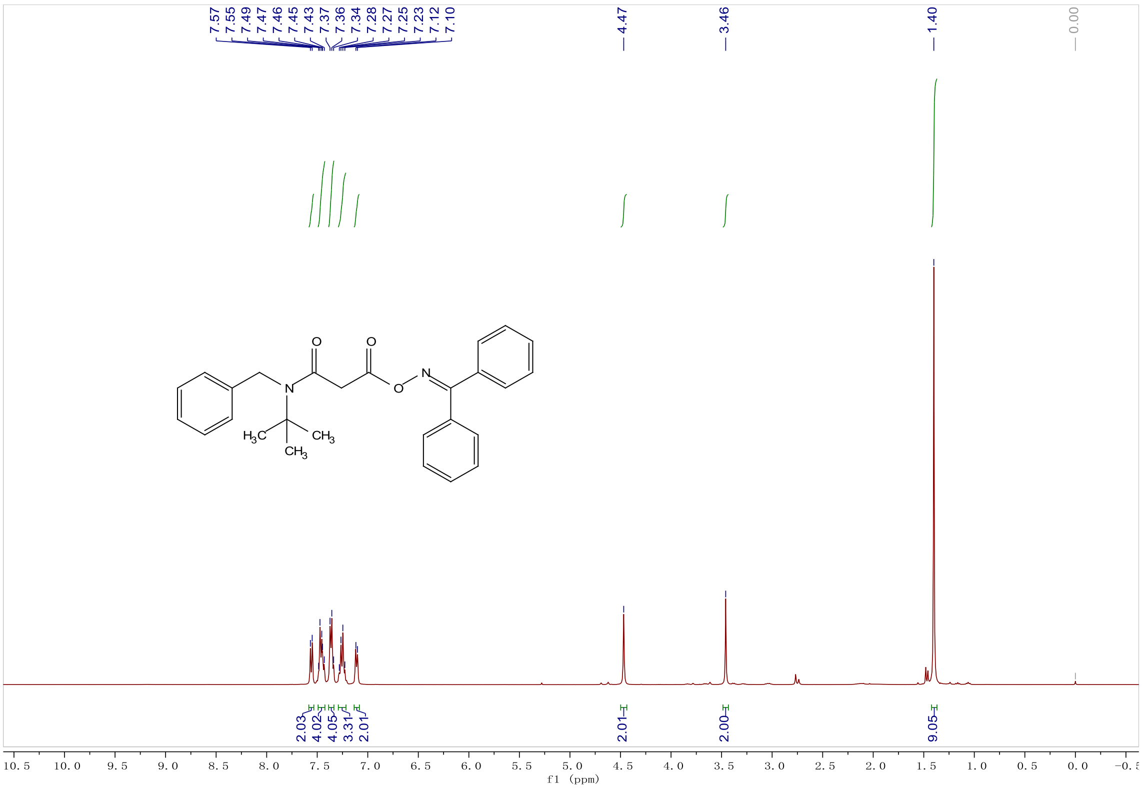

^{1}H-NMR for **1-10**

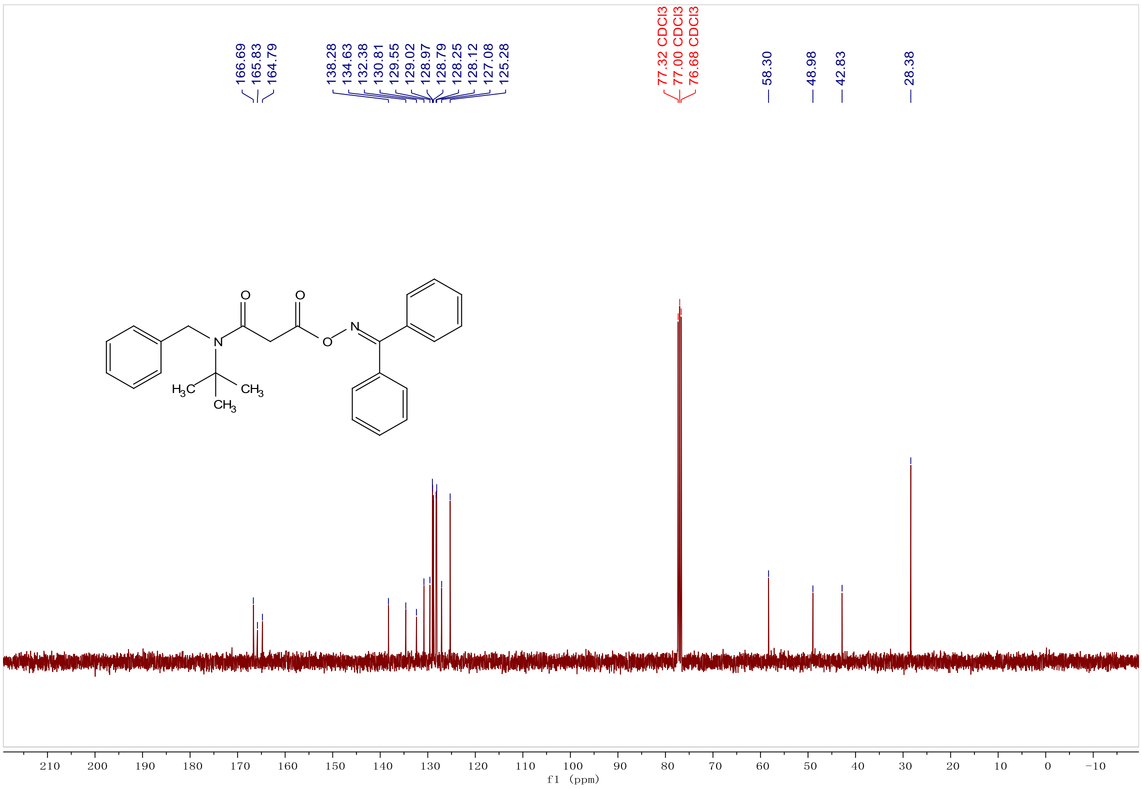

^{13}C−NMR for **1-10**

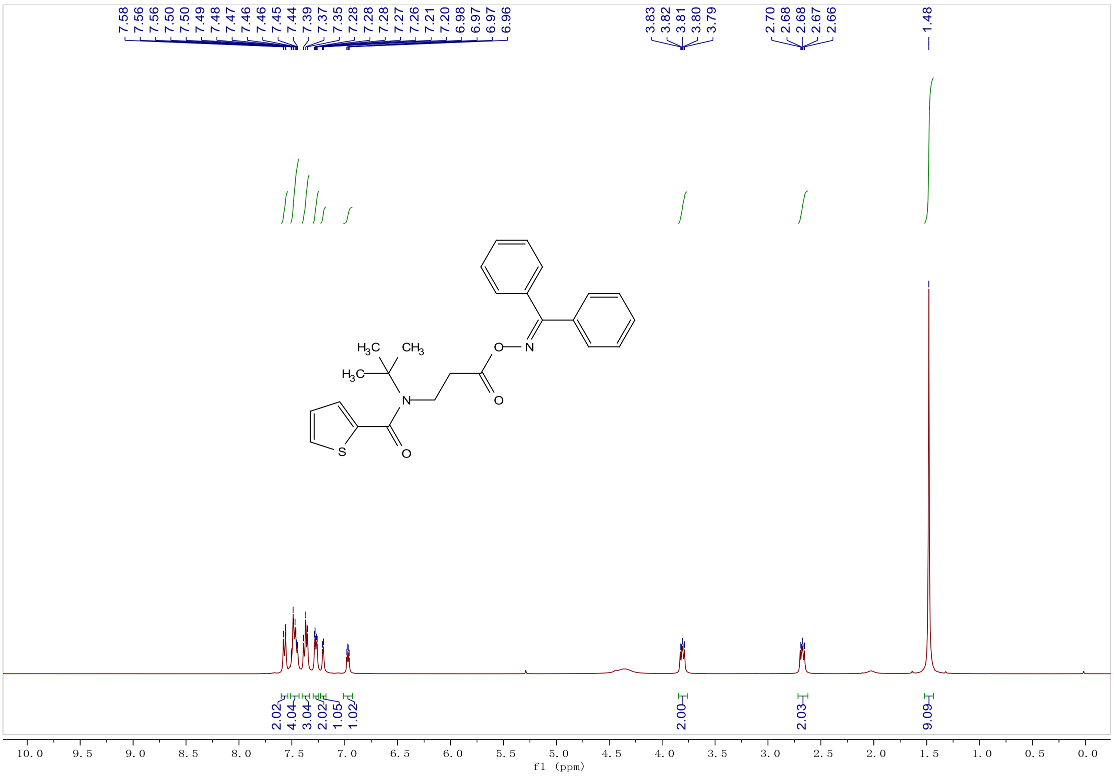

^{1}H NMR of **1-11**

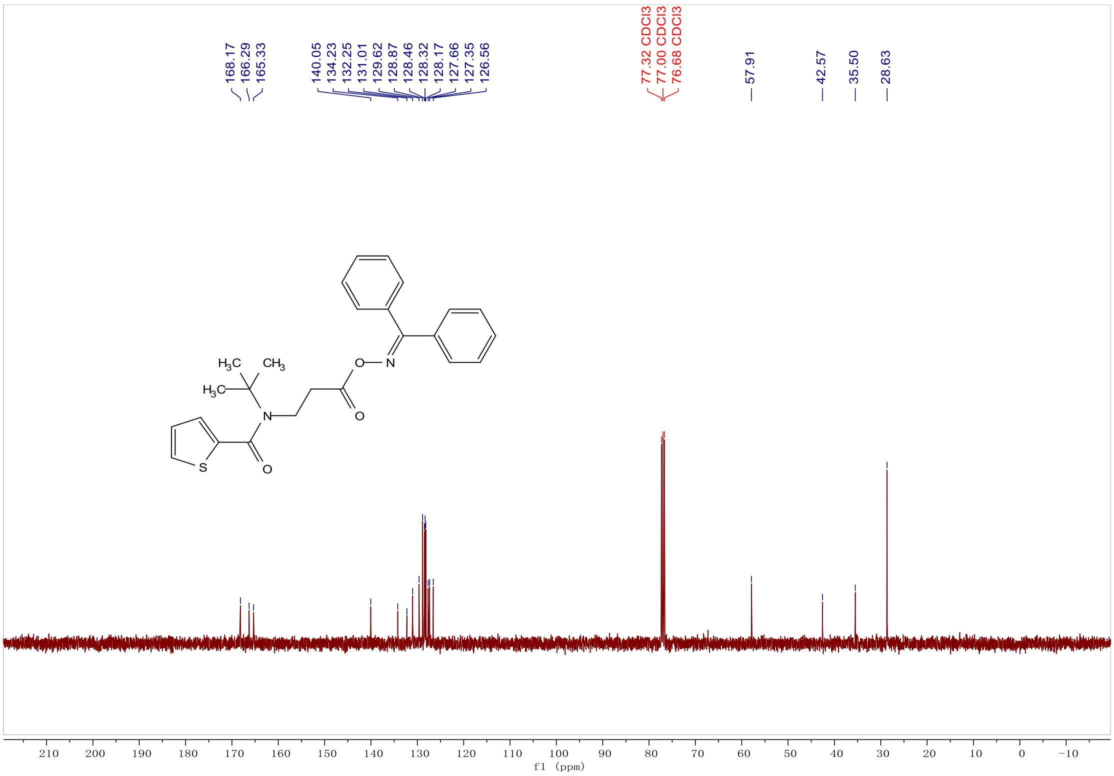

^{13}C NMR of **1-11**

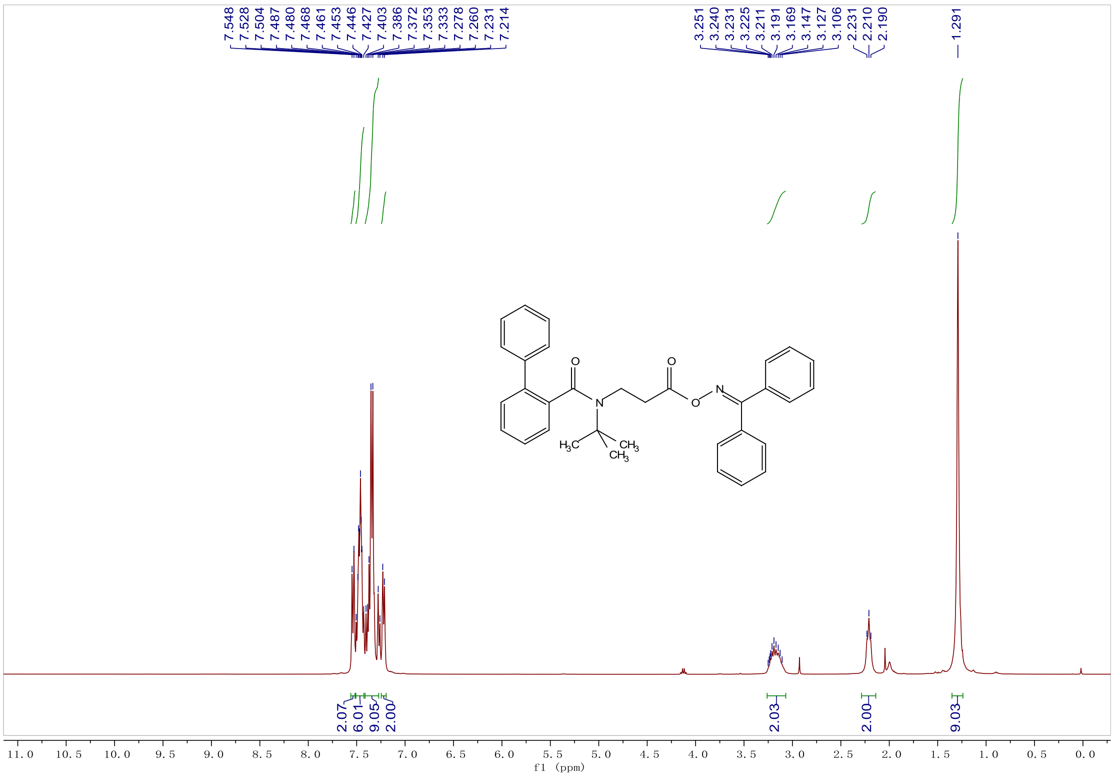

^{1}H NMR of **1-12**

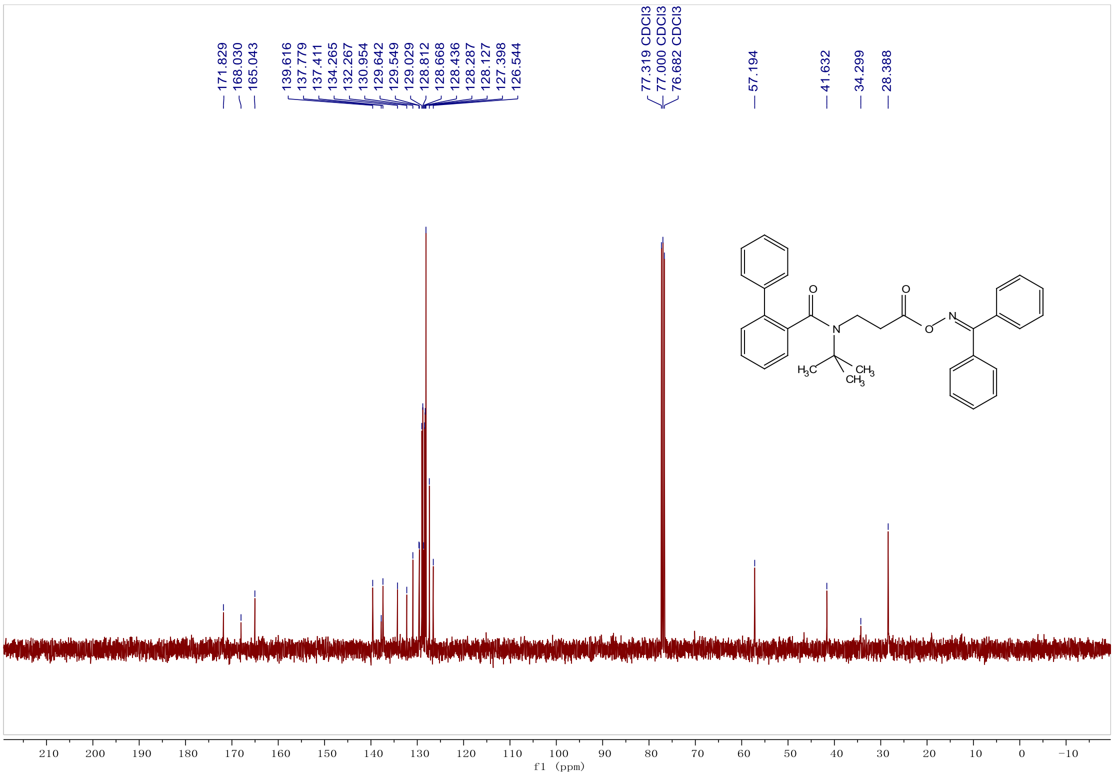

^{13}C NMR of **1-12**

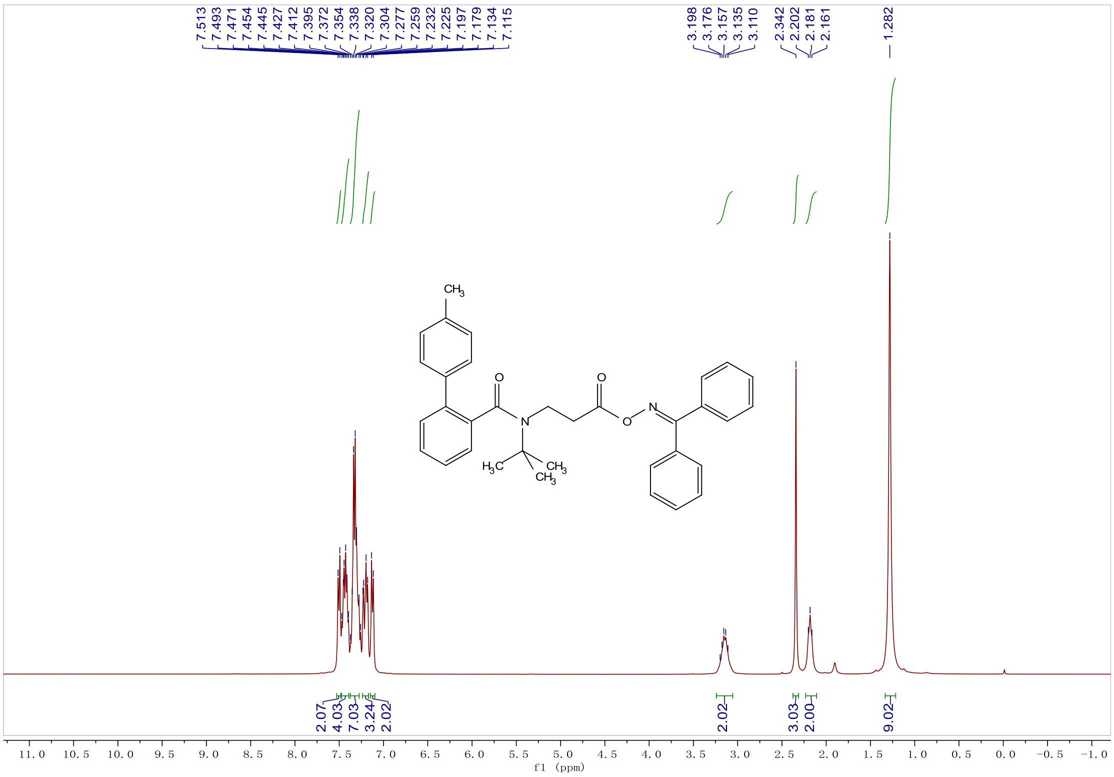

^{1}H NMR of **1-13**

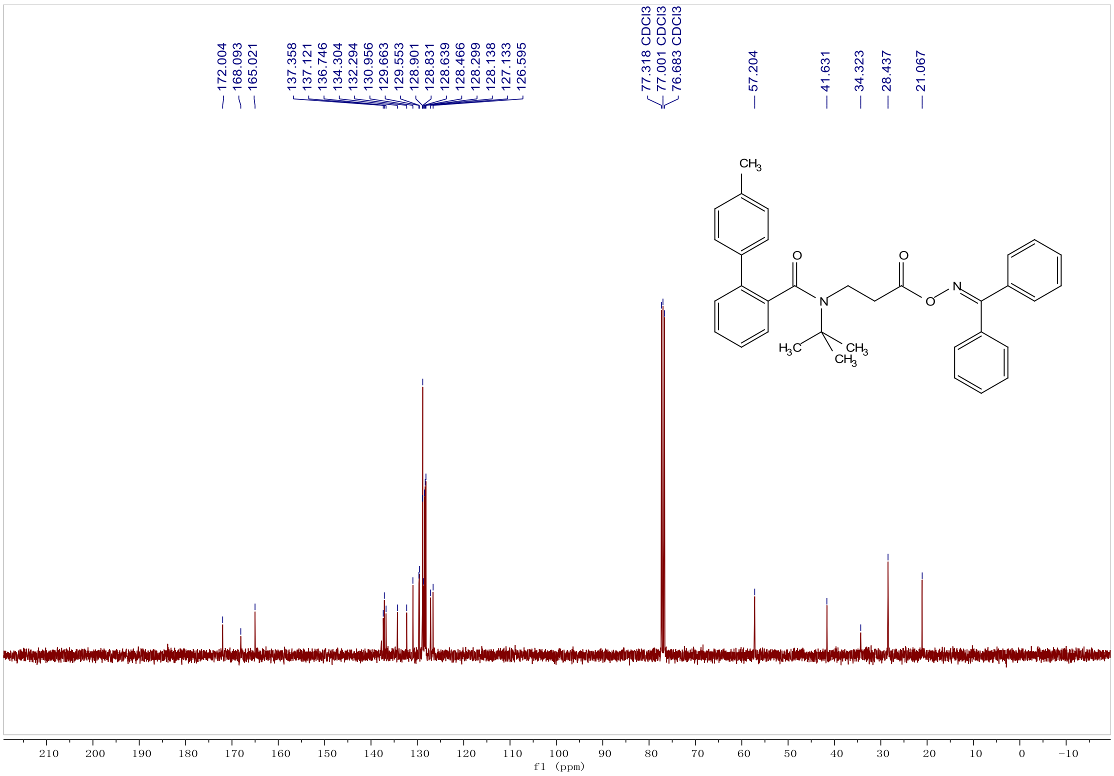

^{13}C NMR of **1-13**

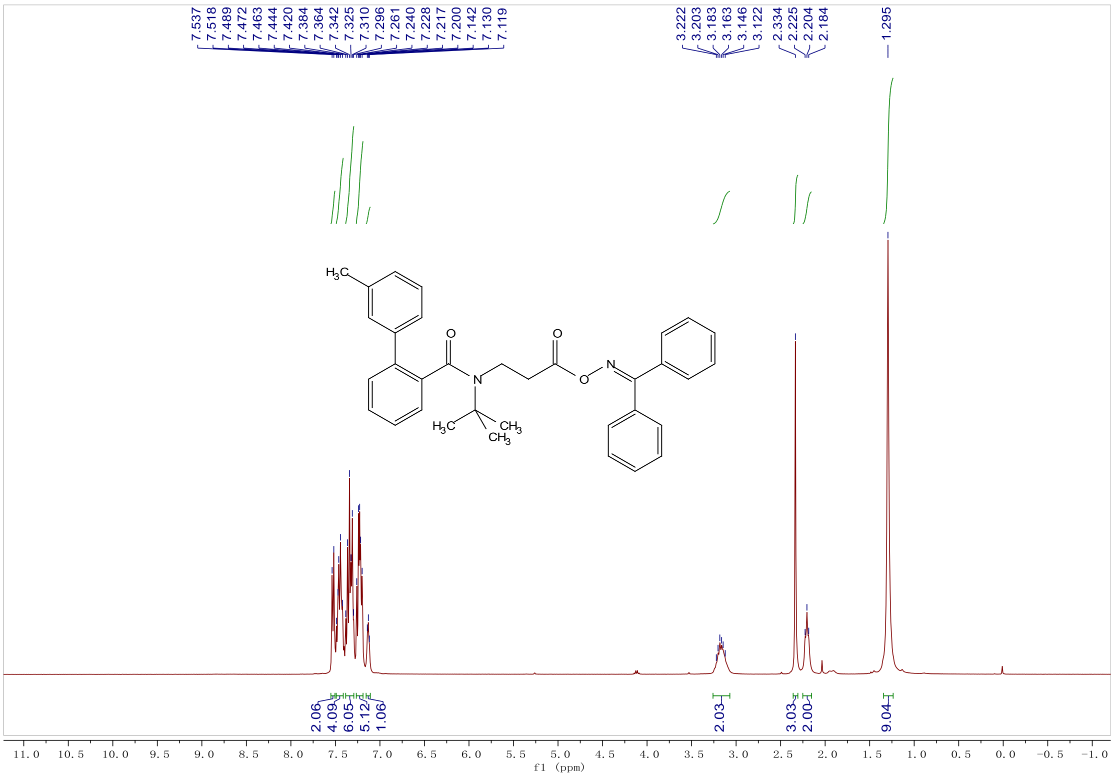

^{1}H NMR of **1-14**

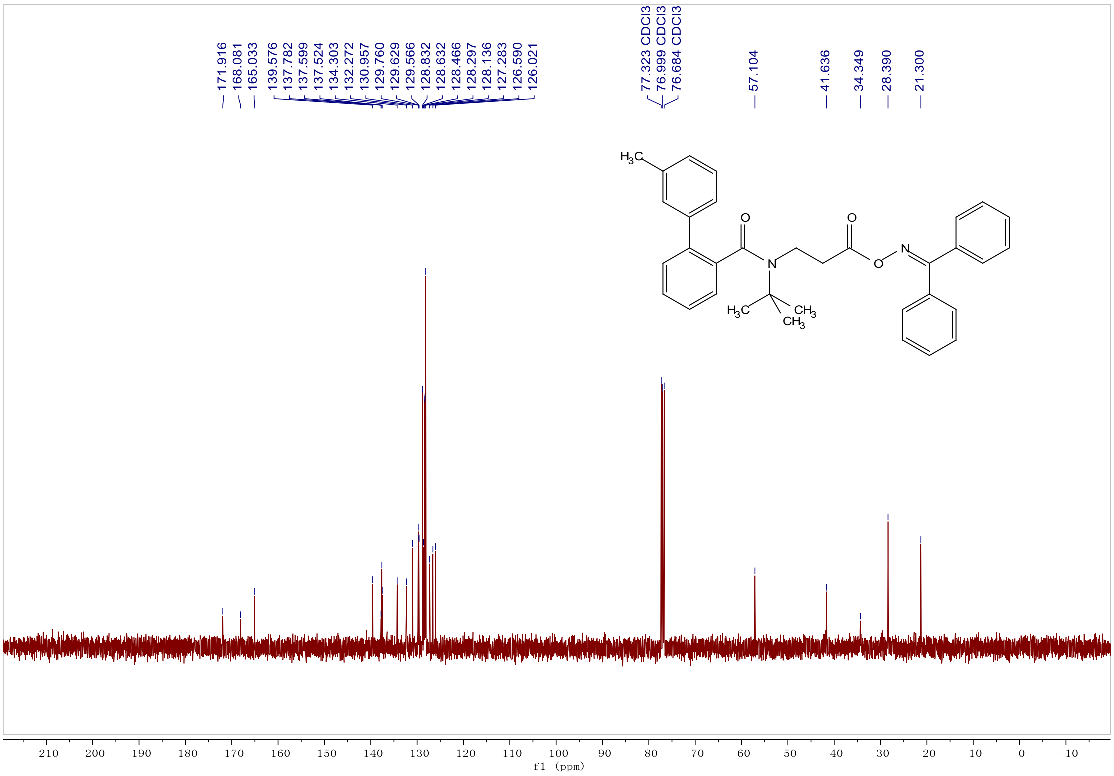

^{13}C NMR of **1-14**

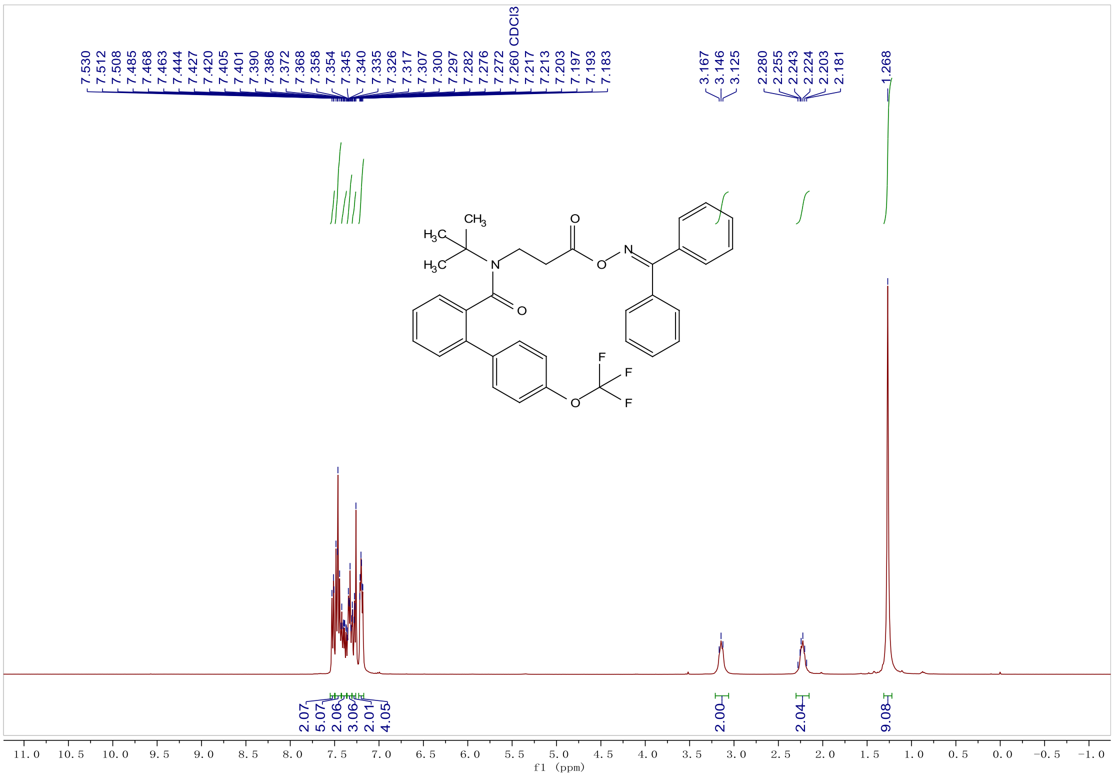

^{1}H NMR of **1-15**

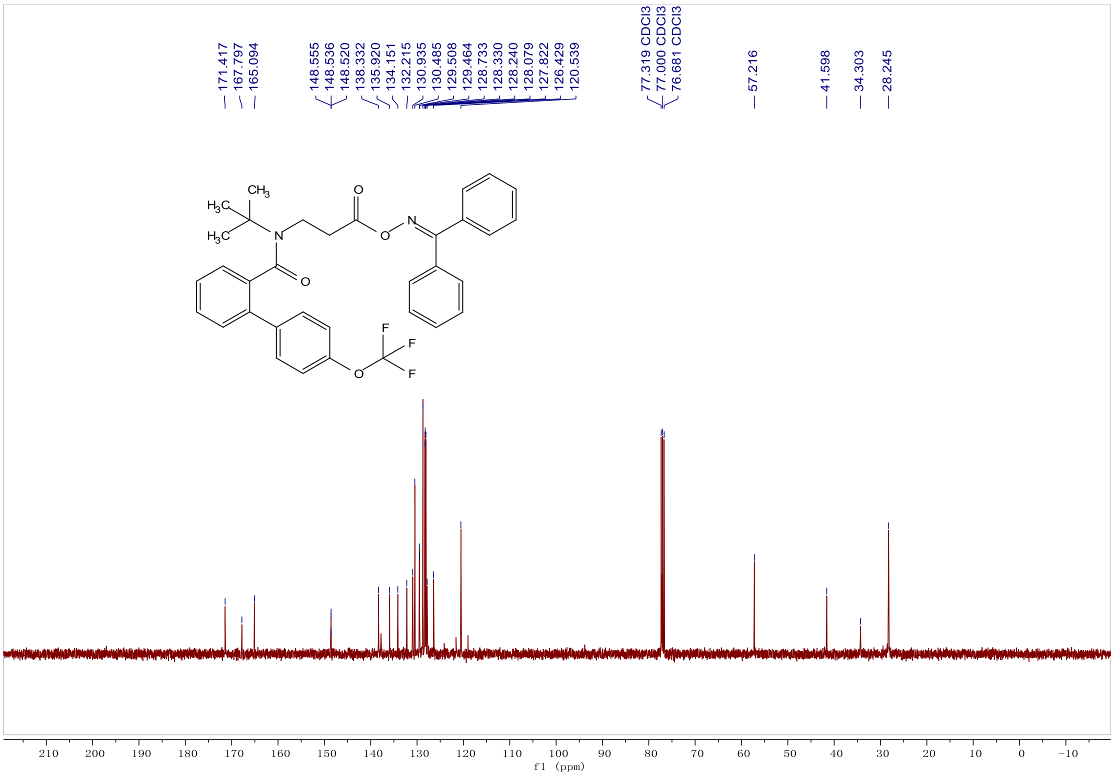

^{13}C NMR of **1-15**

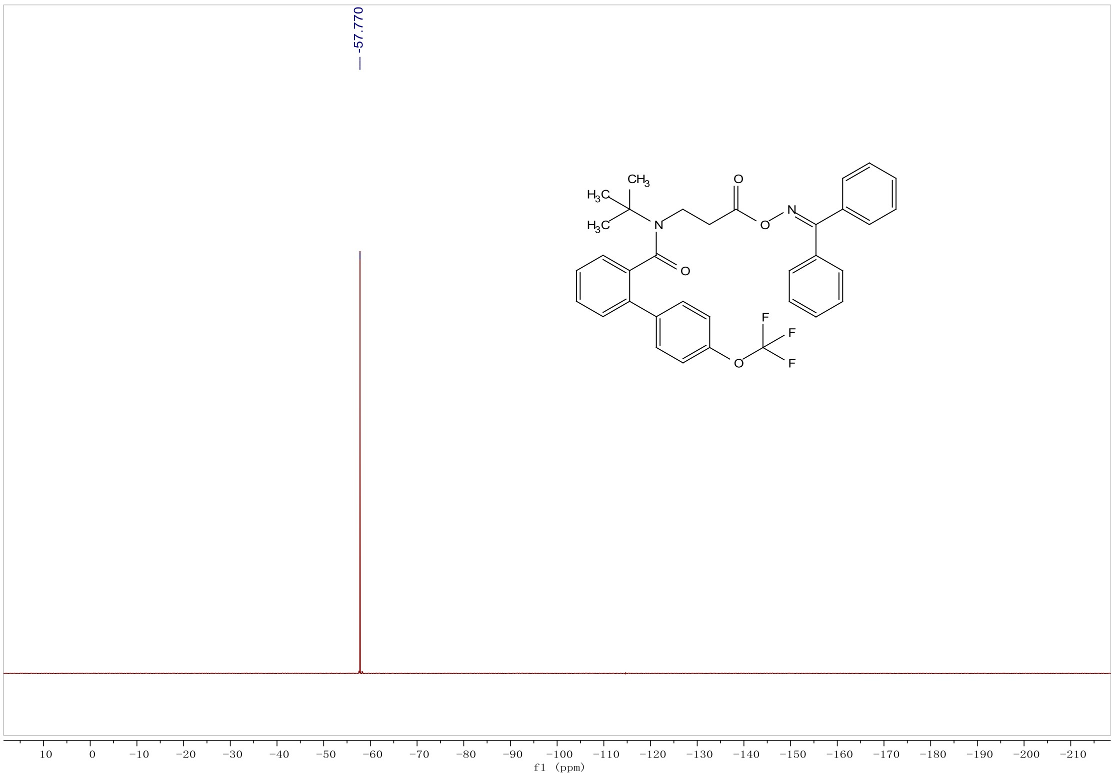

^{19}F NMR of **1-15**

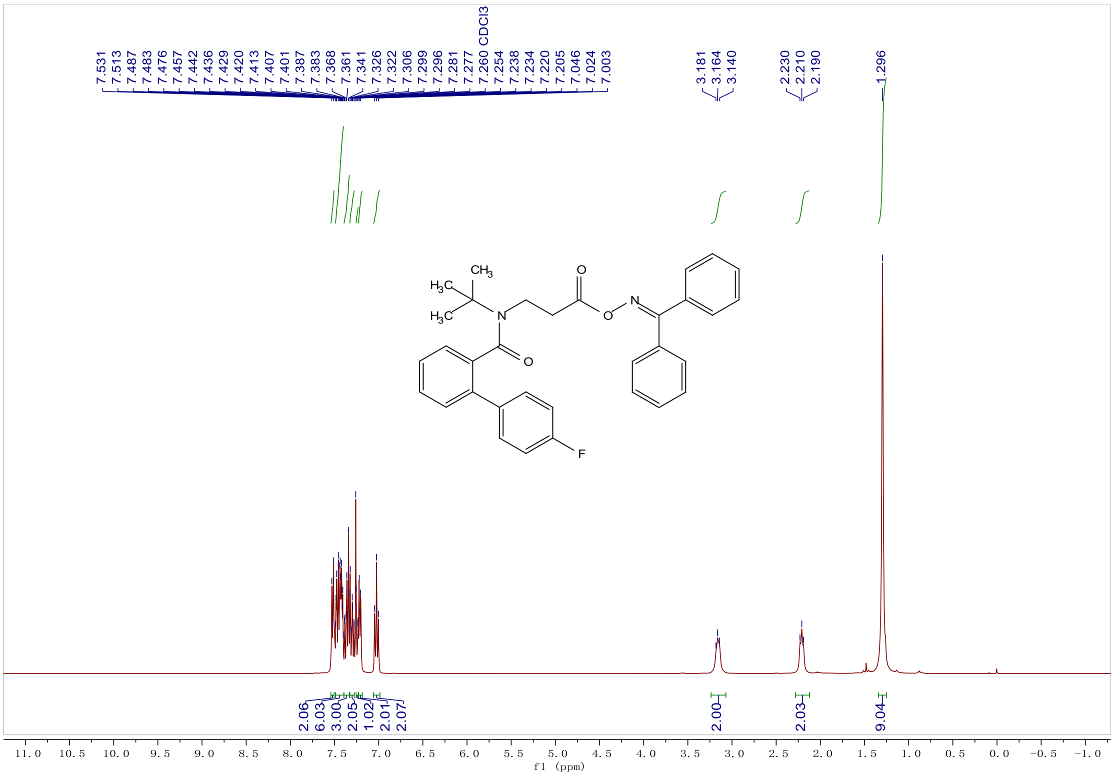

^{1}H NMR of **1-16**

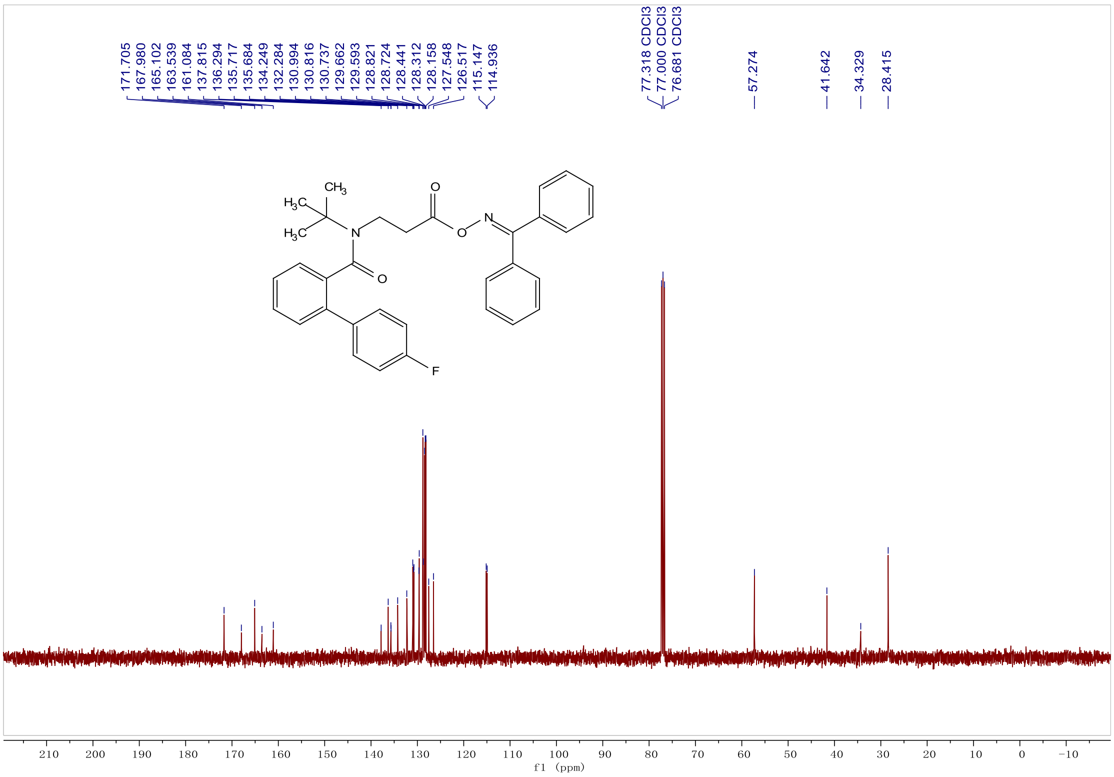

^{13}C NMR of **1-16**

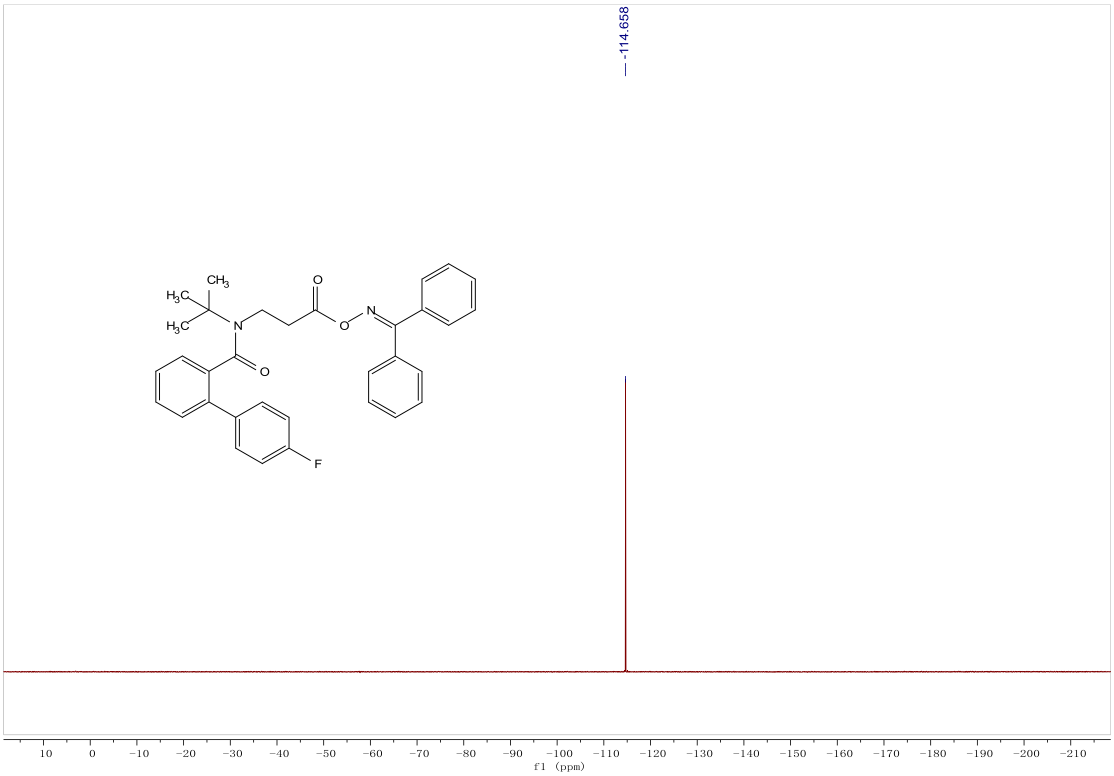

^{19}F NMR of **1-16**

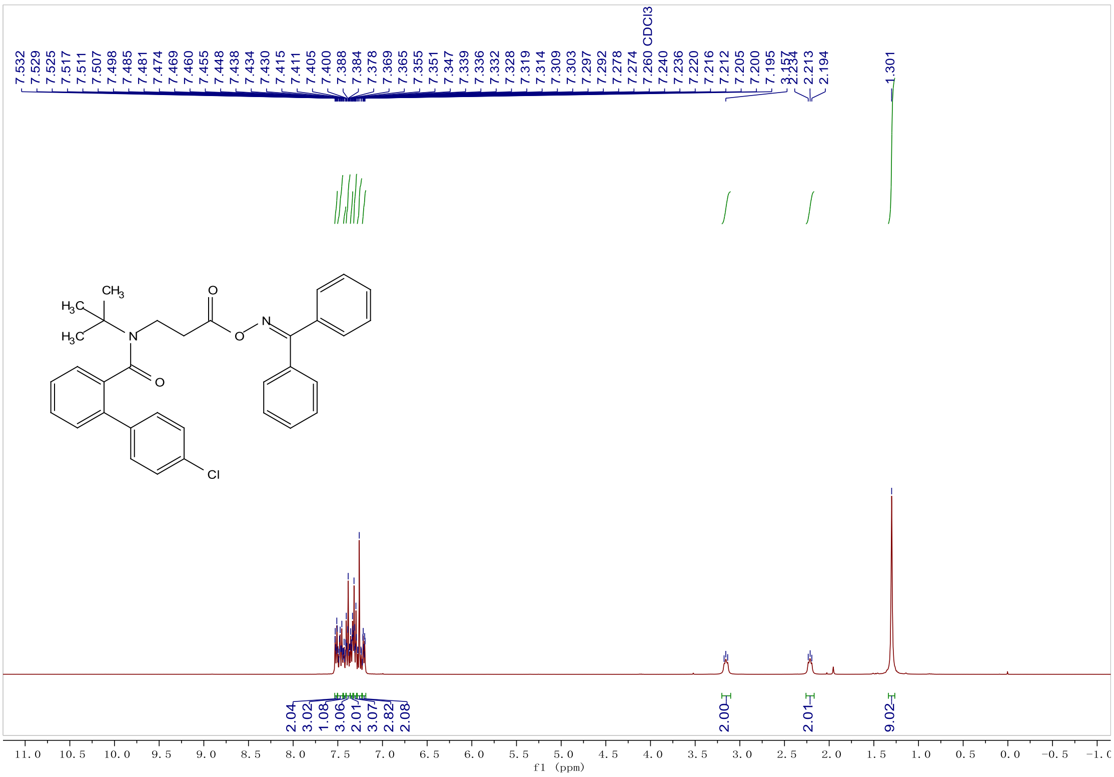

^{1}H NMR of **1-17**

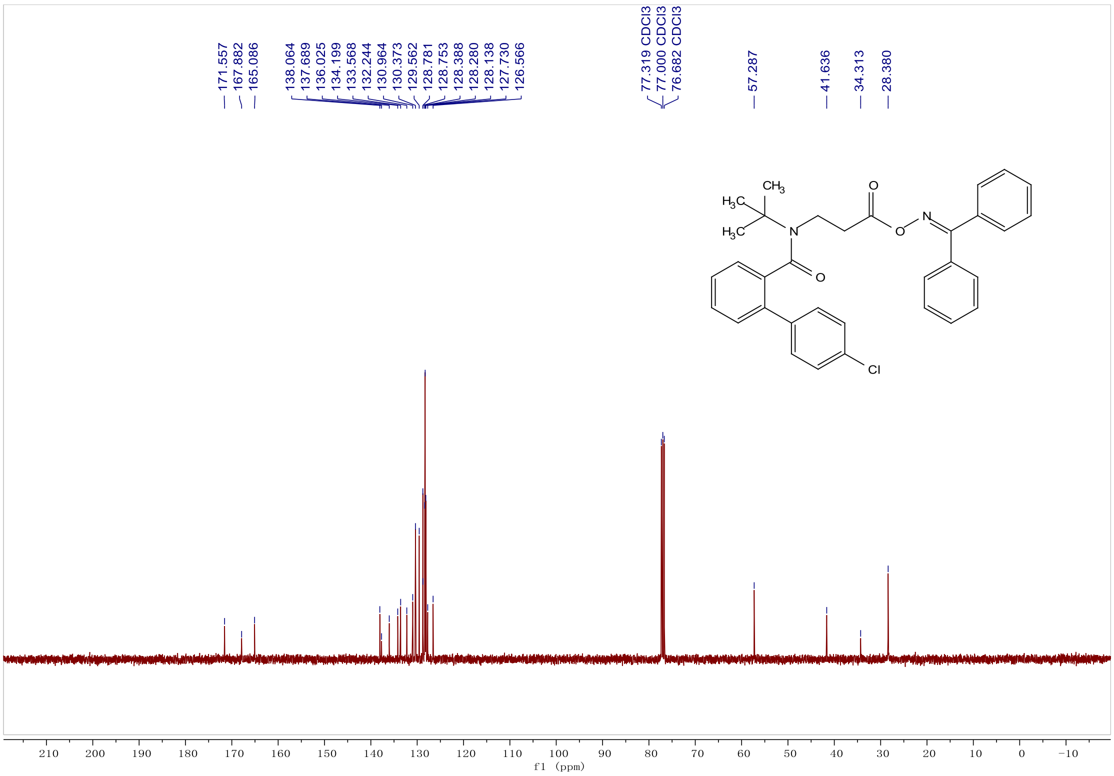

^{13}C NMR of **1-17**

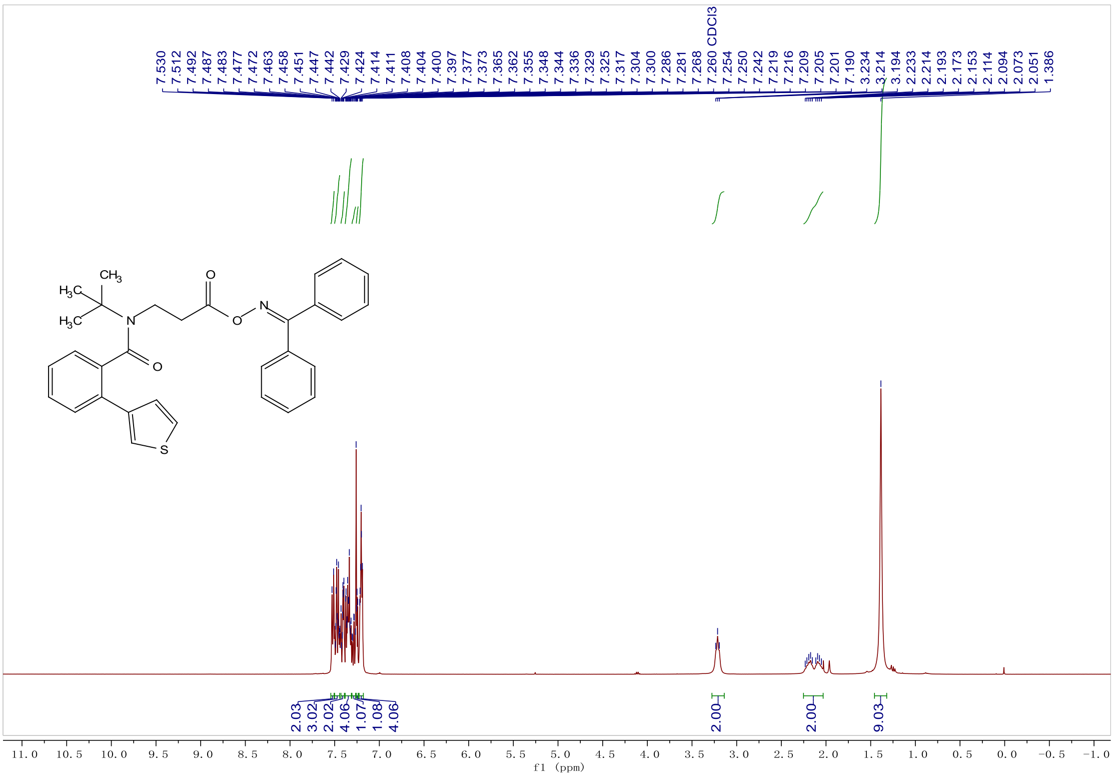

^{1}H NMR of **1-18**

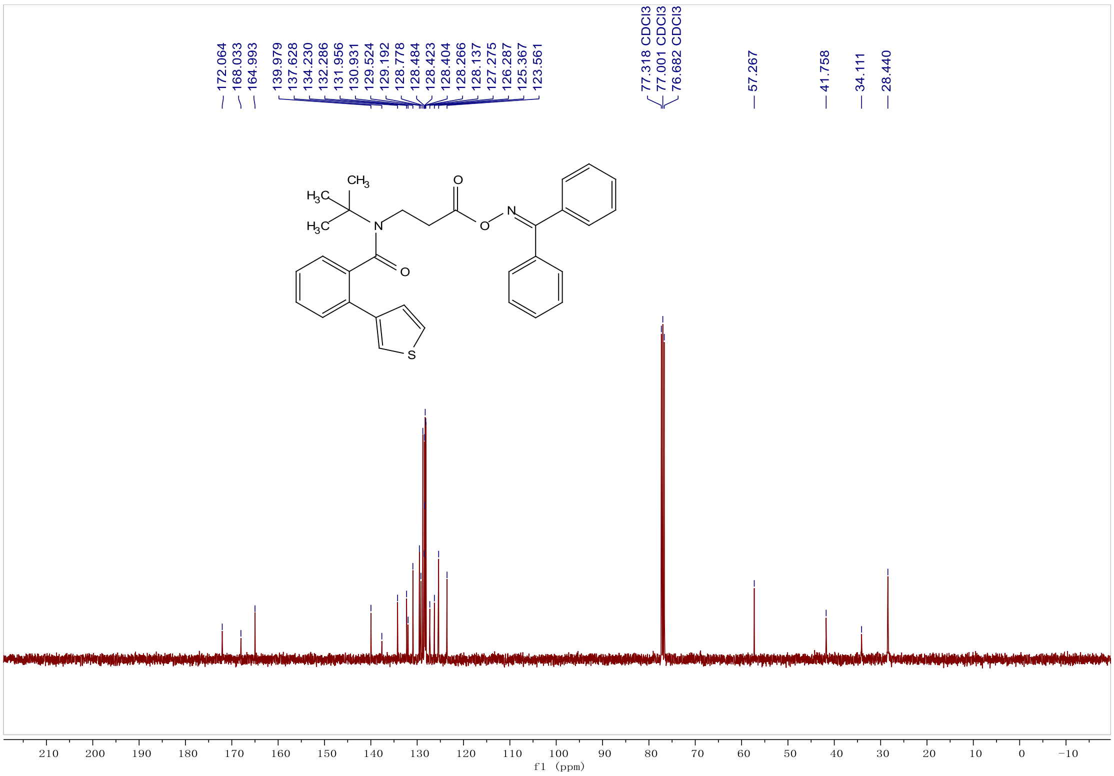

^{13}C NMR of **1-18**

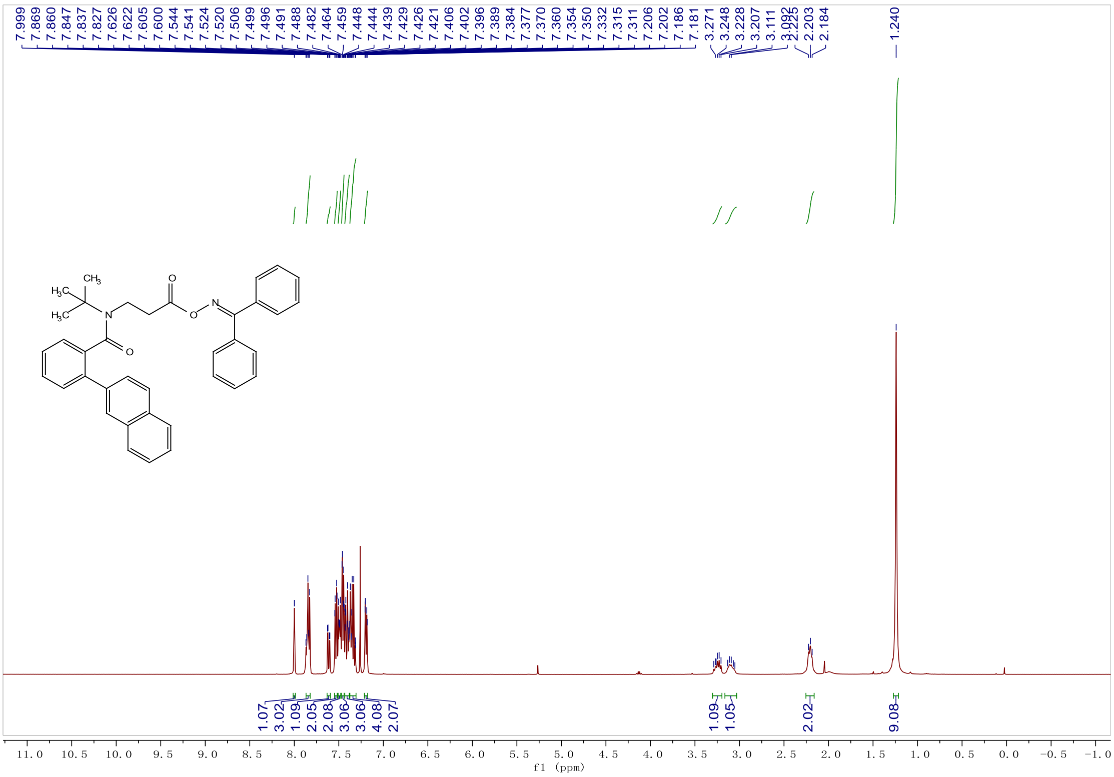

^{1}H NMR of **1-19**

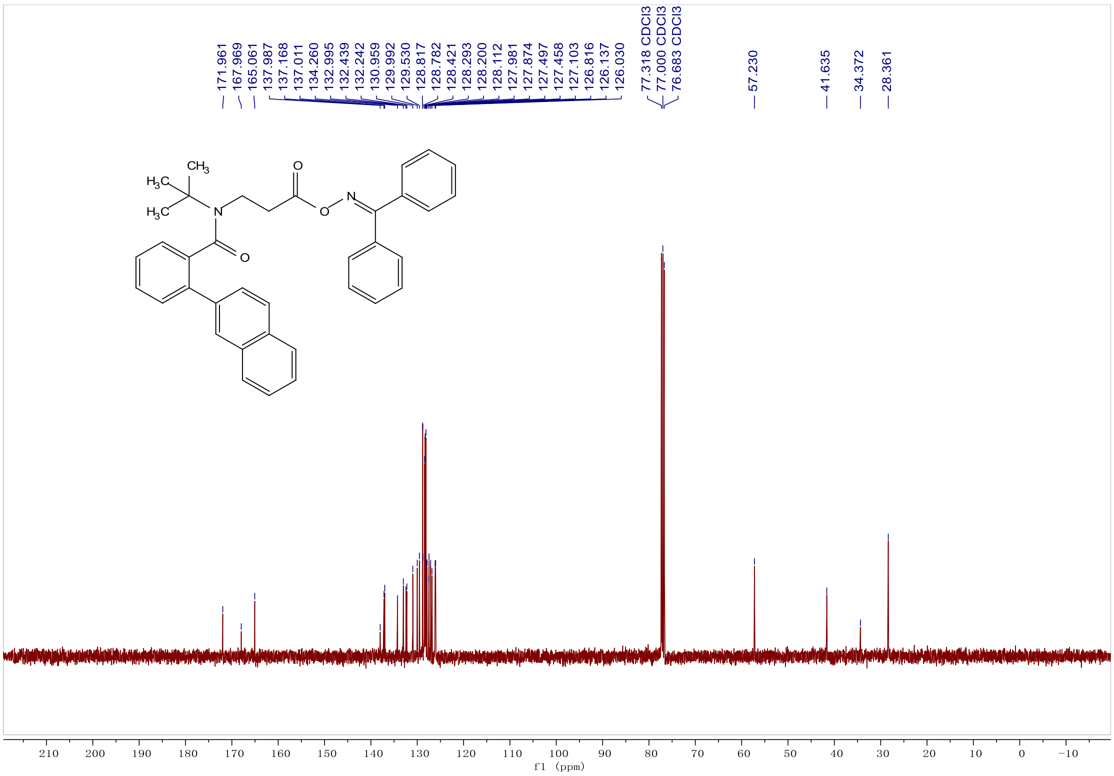

^{13}C NMR of **1-19**

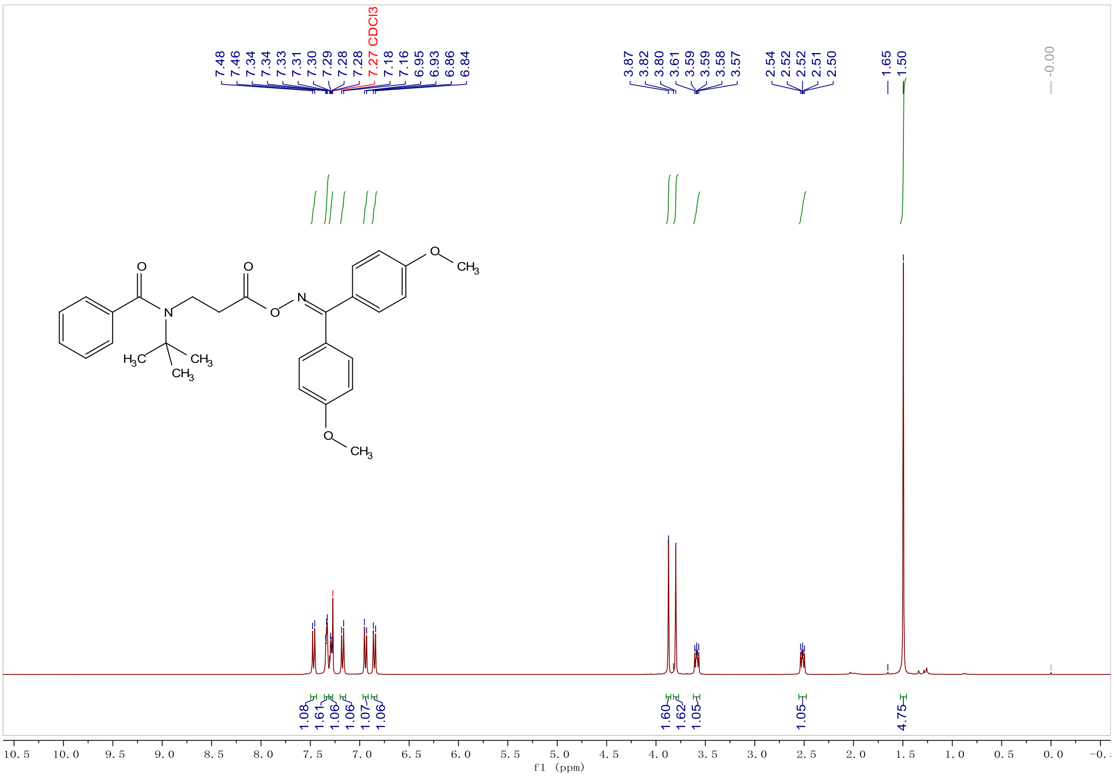

^{1}H NMR of **1-20**

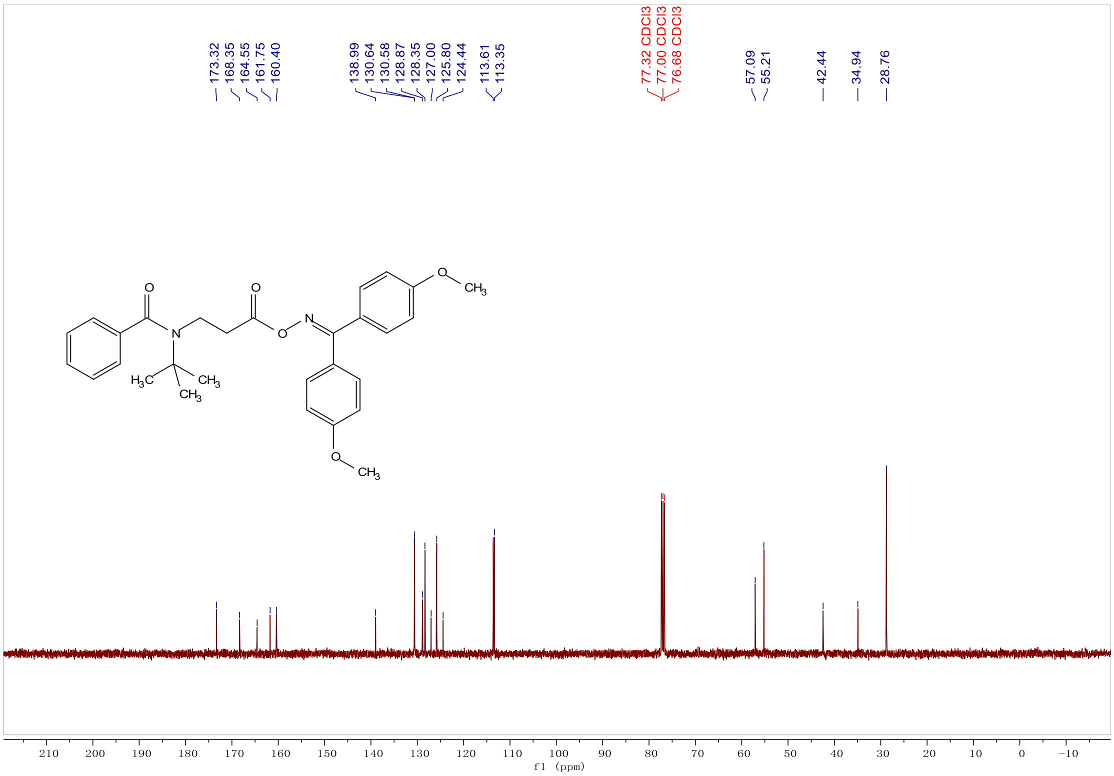

^{13}C NMR of **1-20**

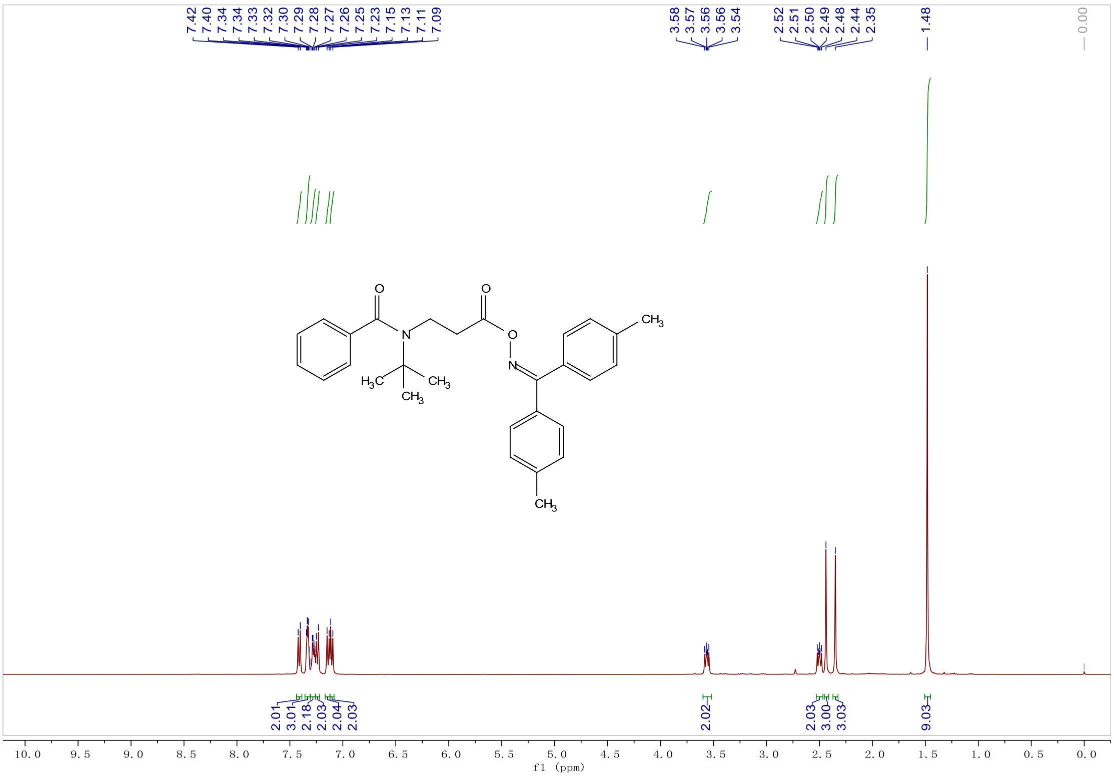

^{1}H NMR of **1-21**

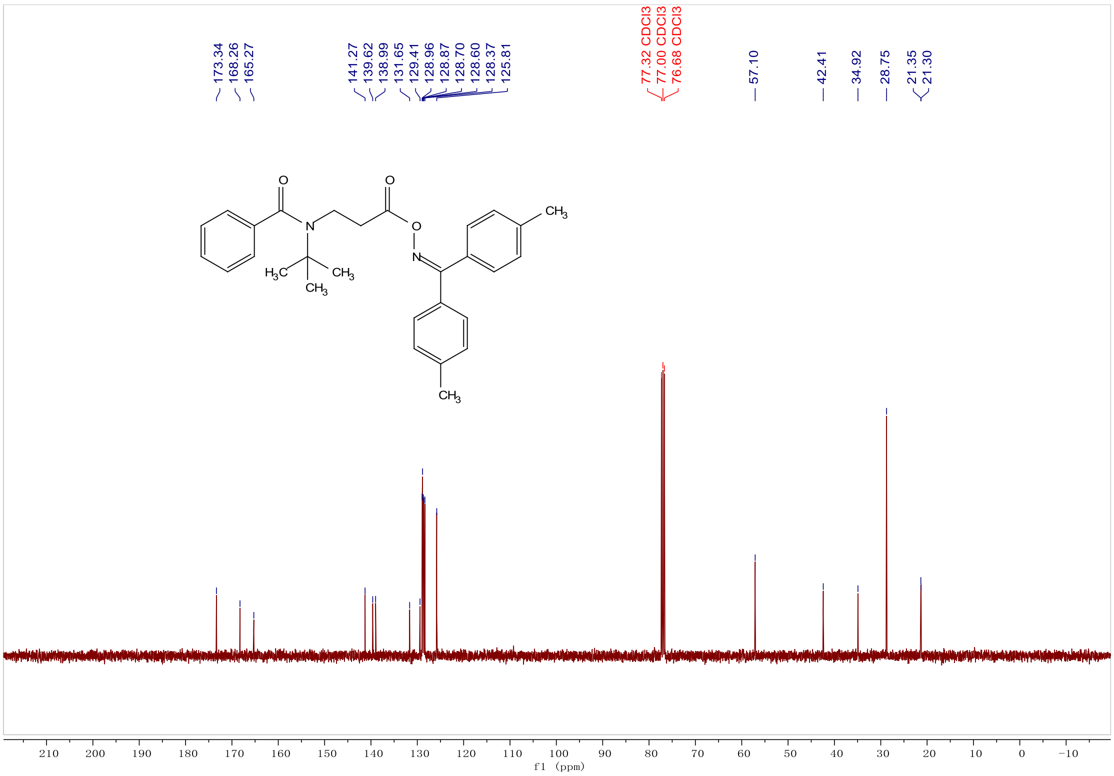

^{13}C NMR of **1-21**

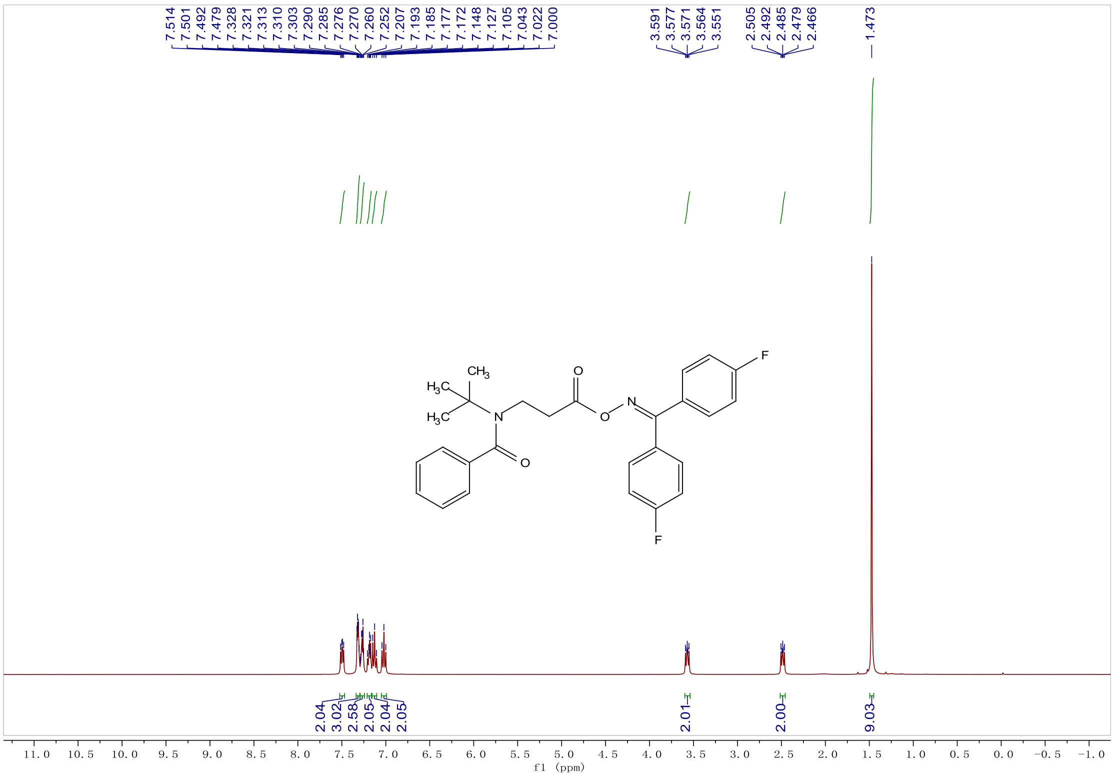

^{1}H NMR of **1-22**

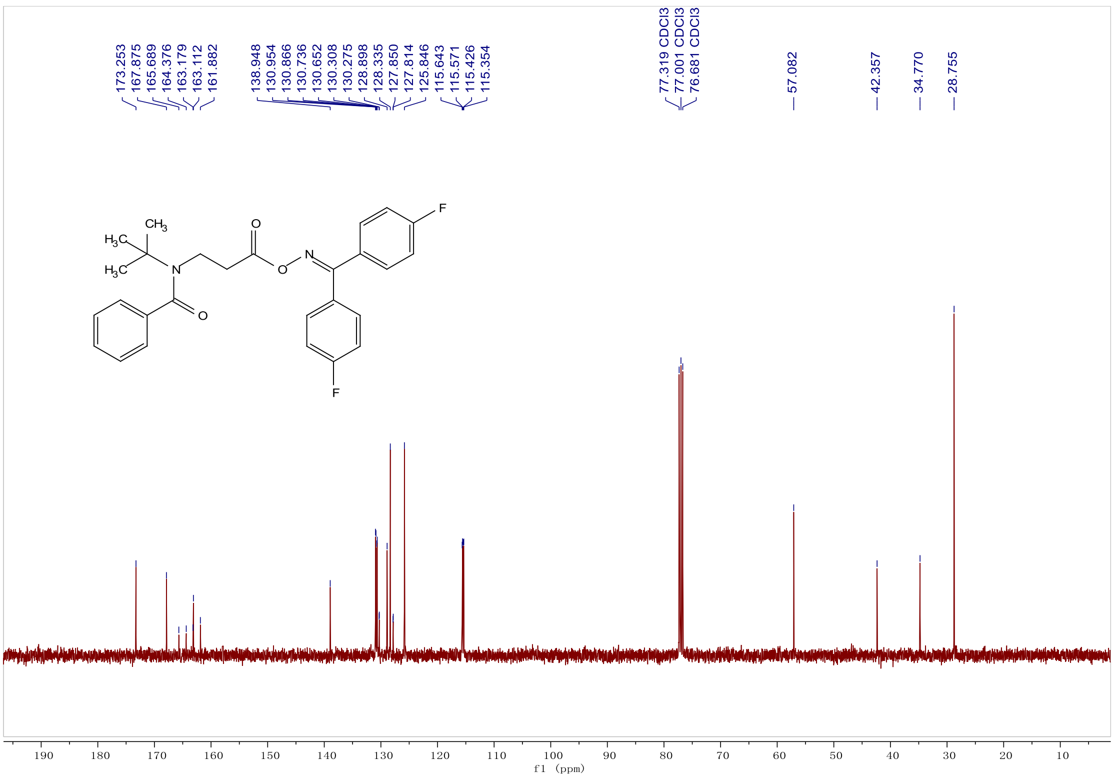

^{13}C NMR of **1-22**

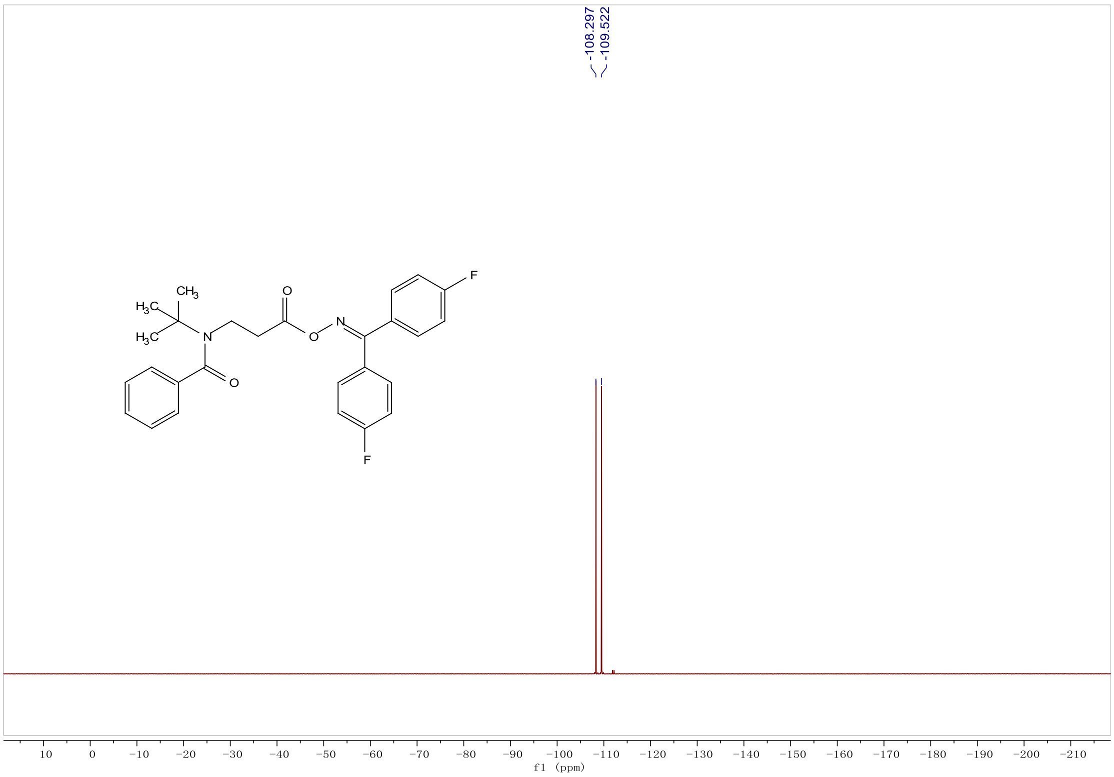

^{19}F NMR of **1-22**

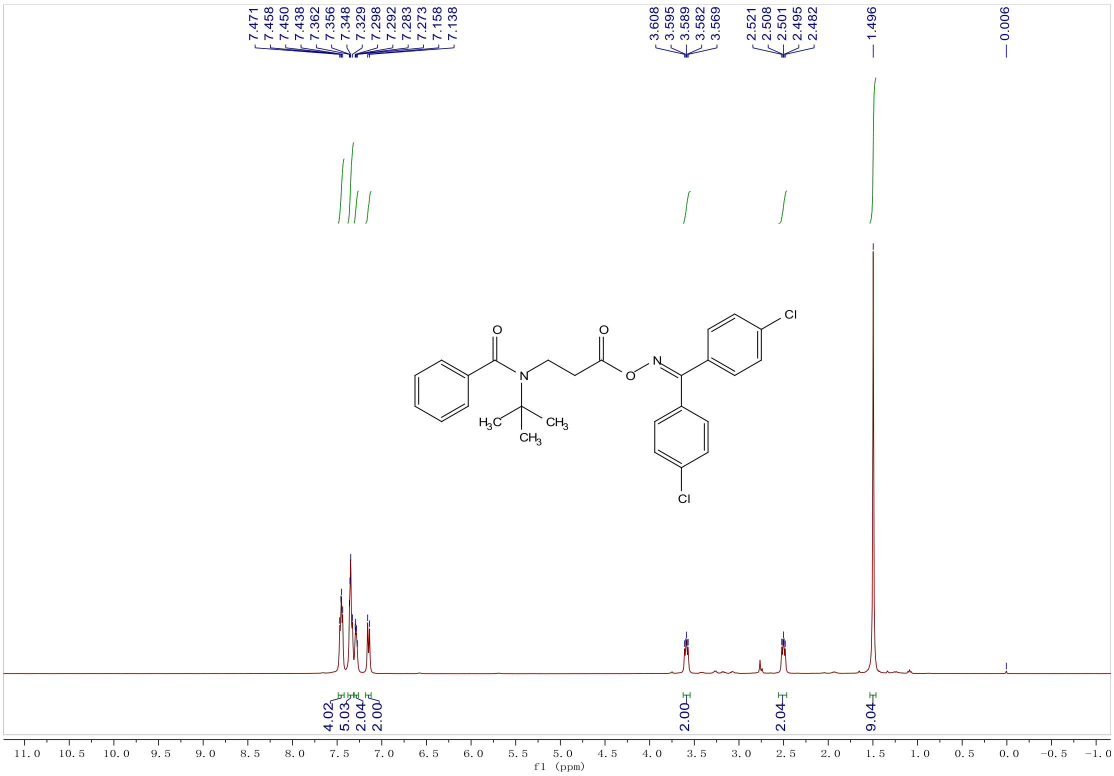

^{1}H NMR of **1-23**

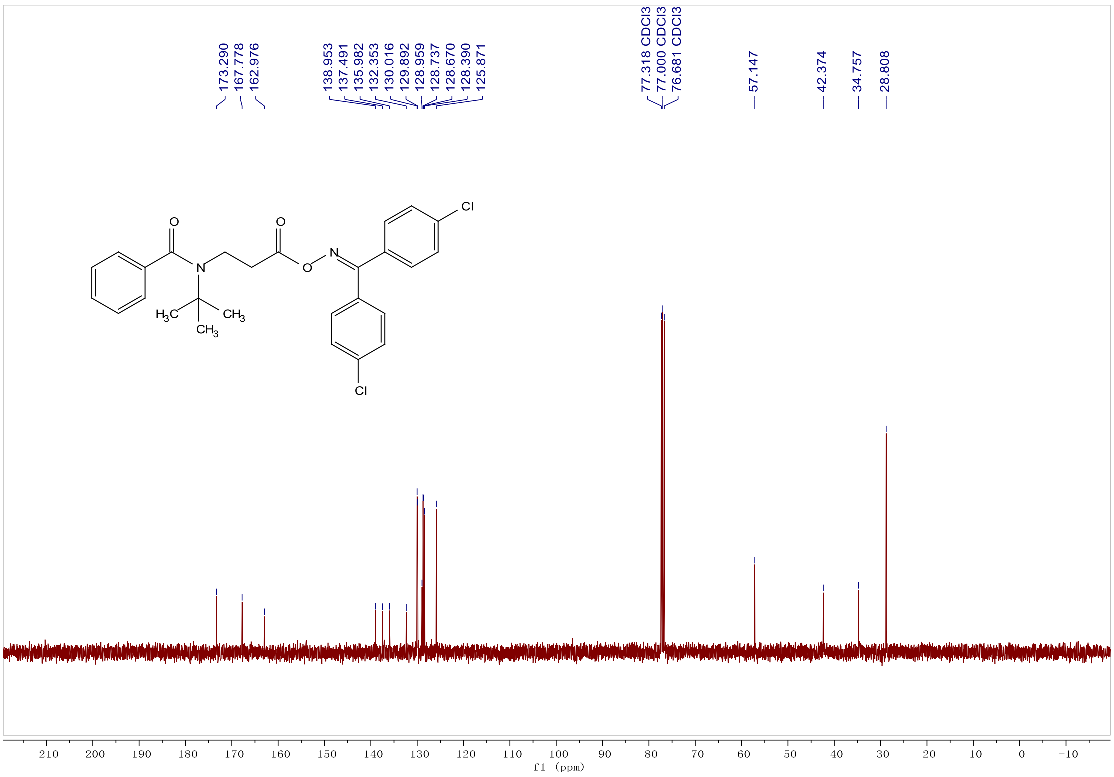

^{13}C NMR of **1-23**

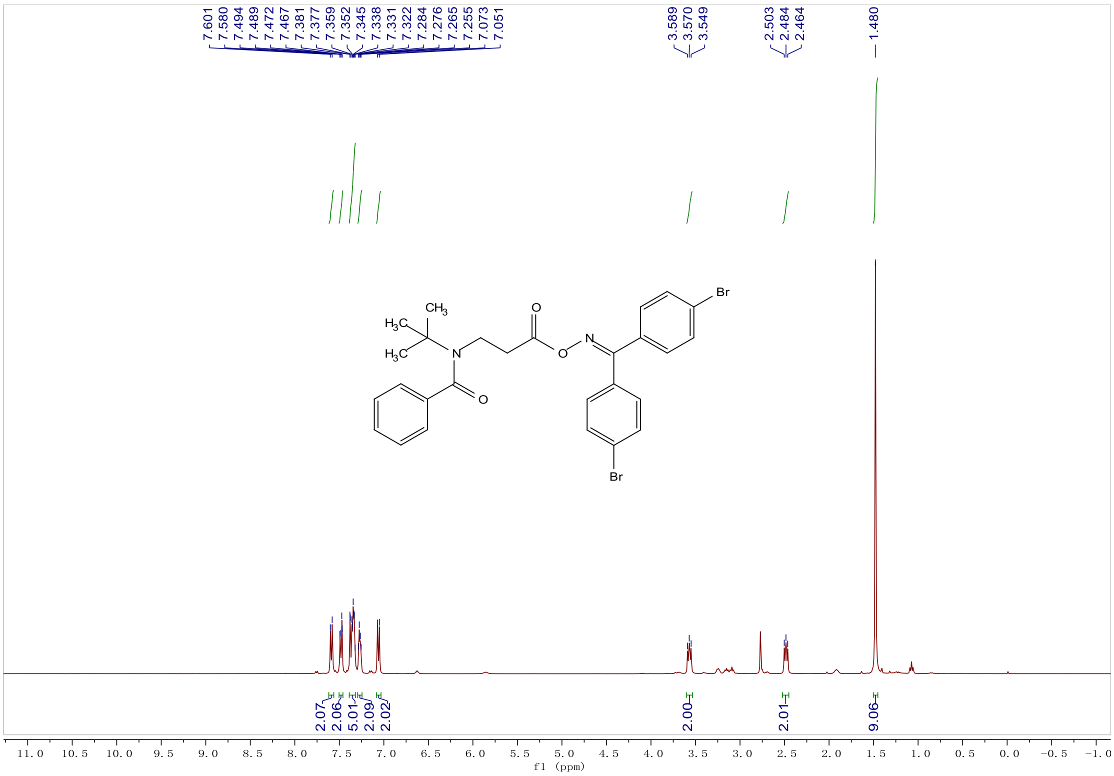

^{1}H NMR of **1-24**

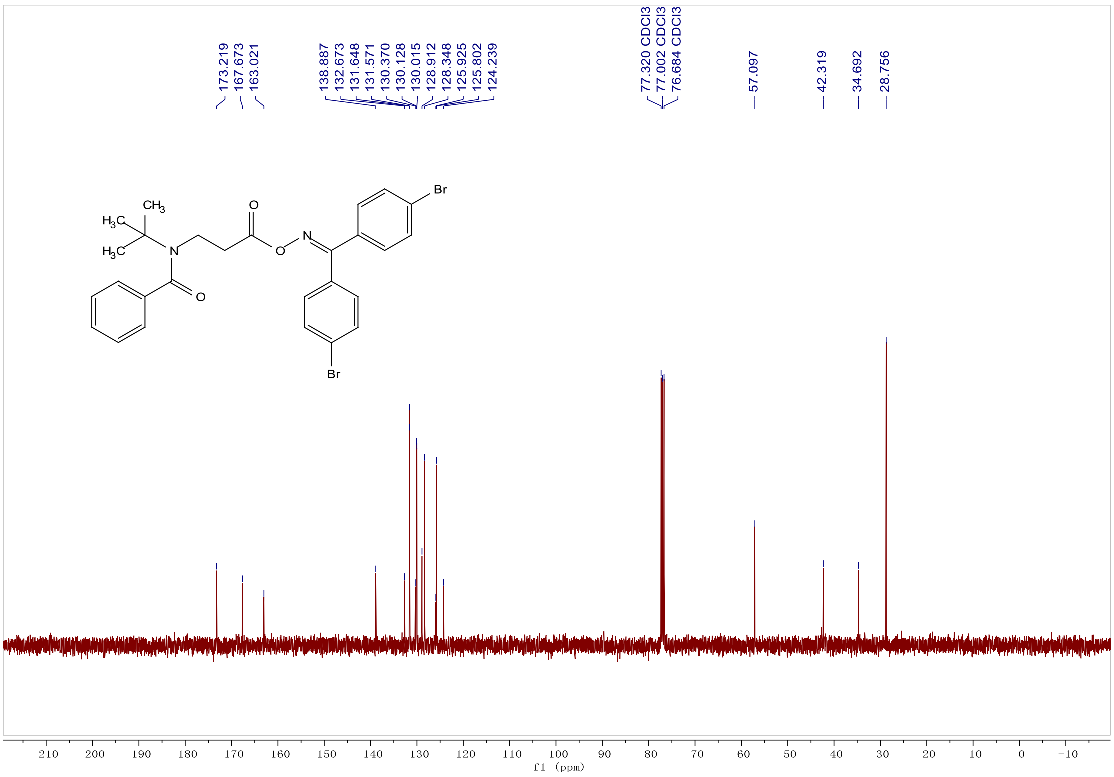

^{13}C NMR of **1-24**

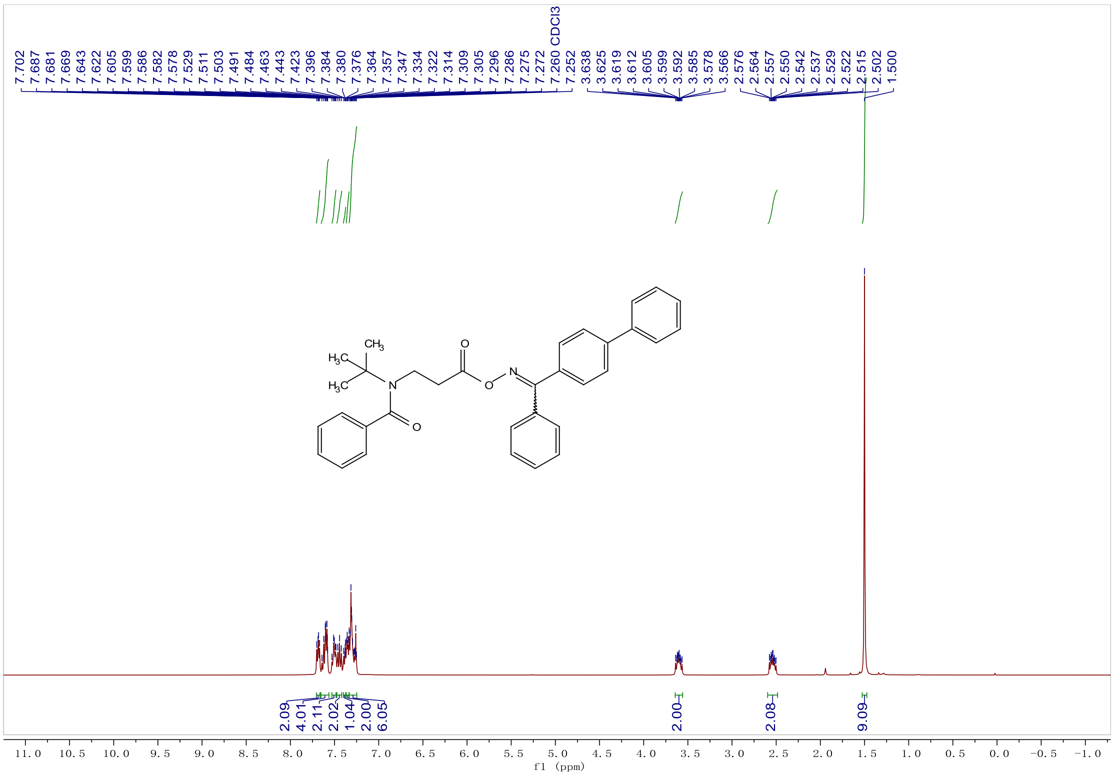

^{1}H NMR of **1-25**

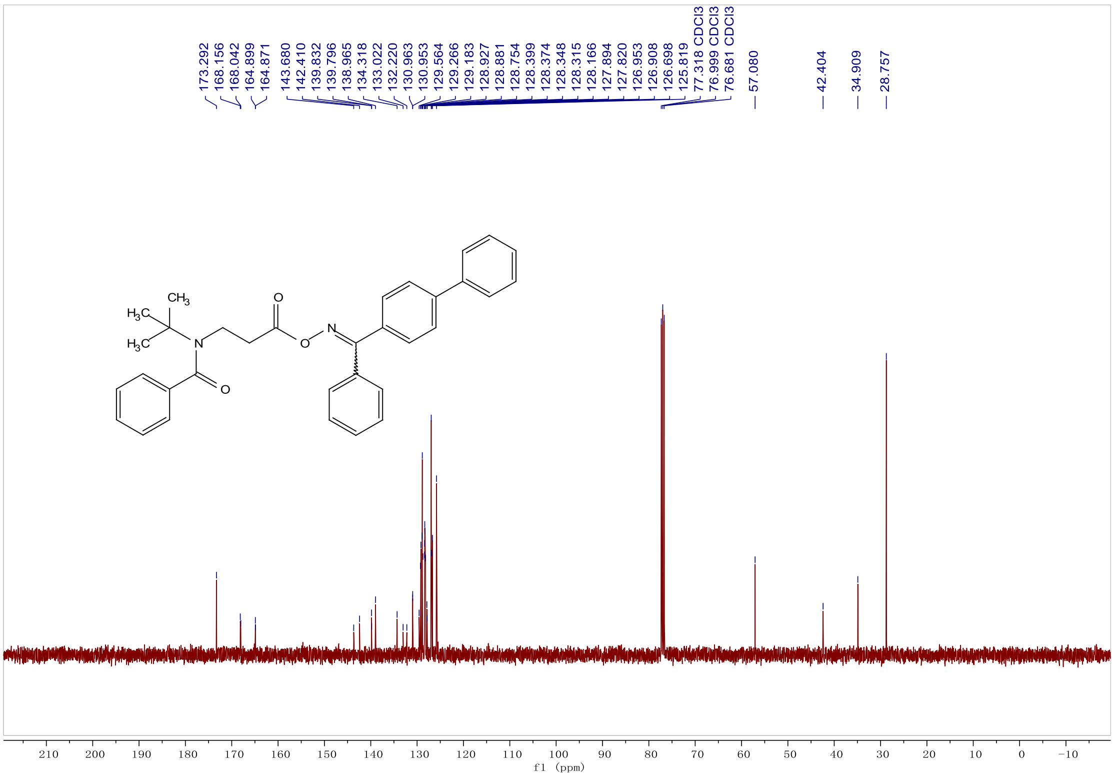

^{13}C NMR of **1-25**

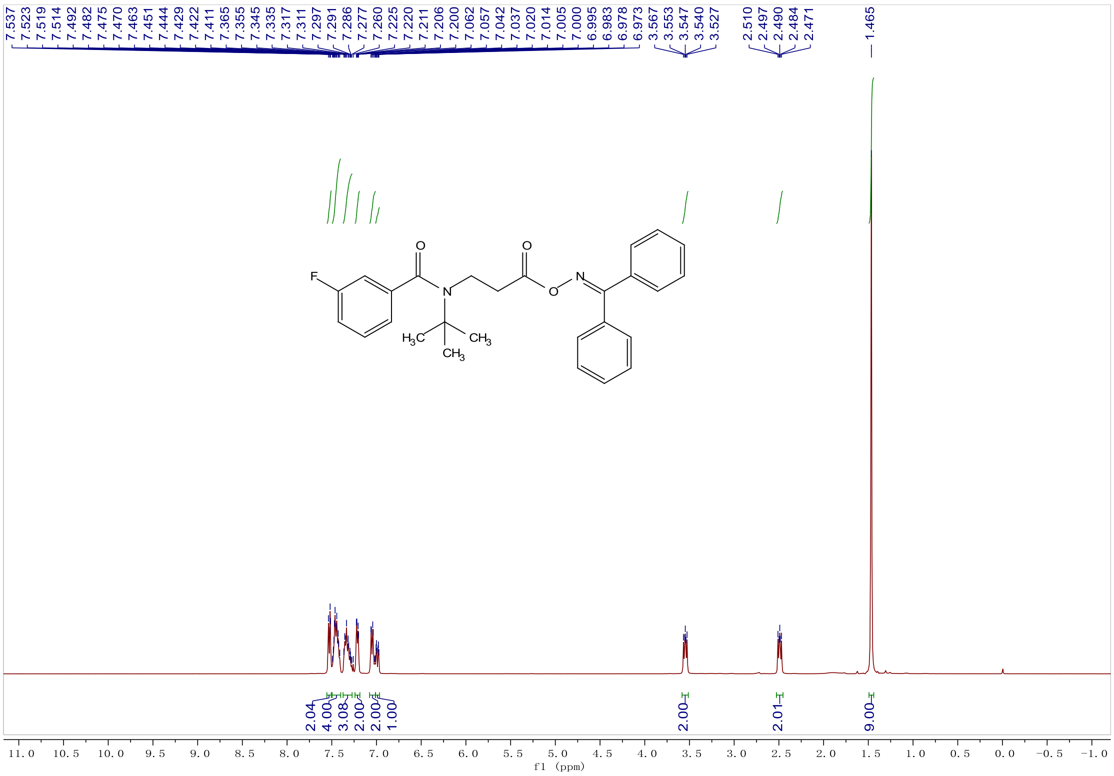

^{1}H-NMR for **1-26**

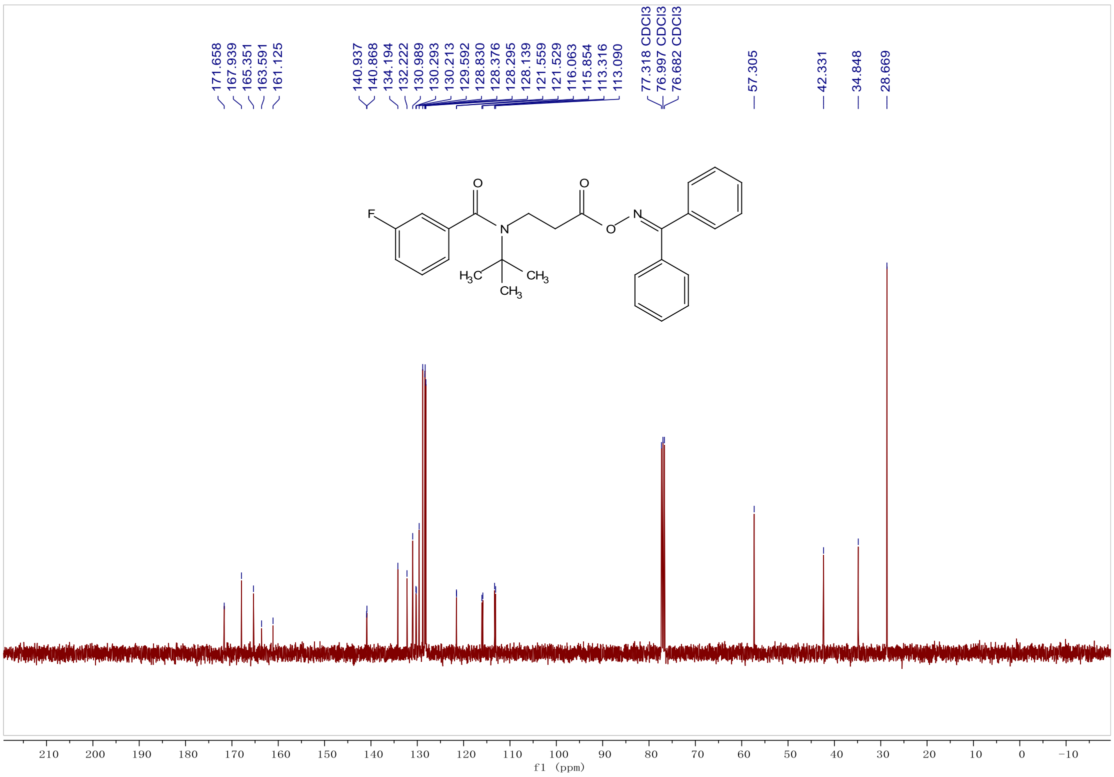

^{13}C−NMR for **1-26**

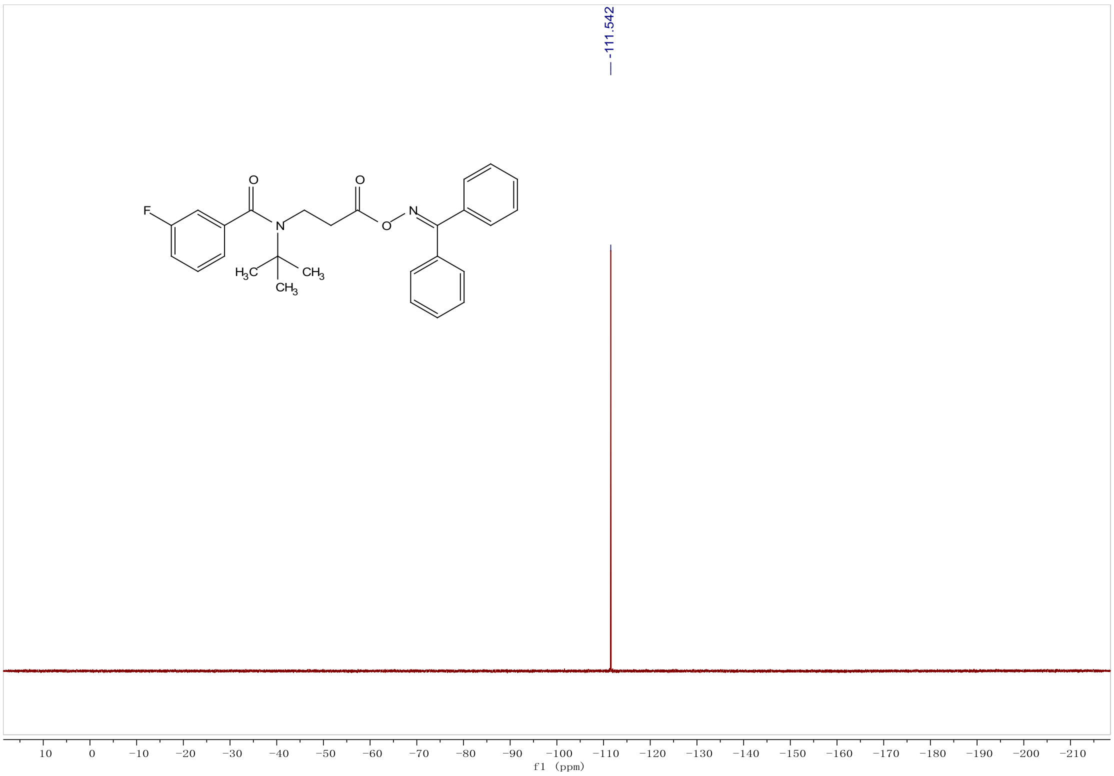

^{19}F−NMR for **1-26**

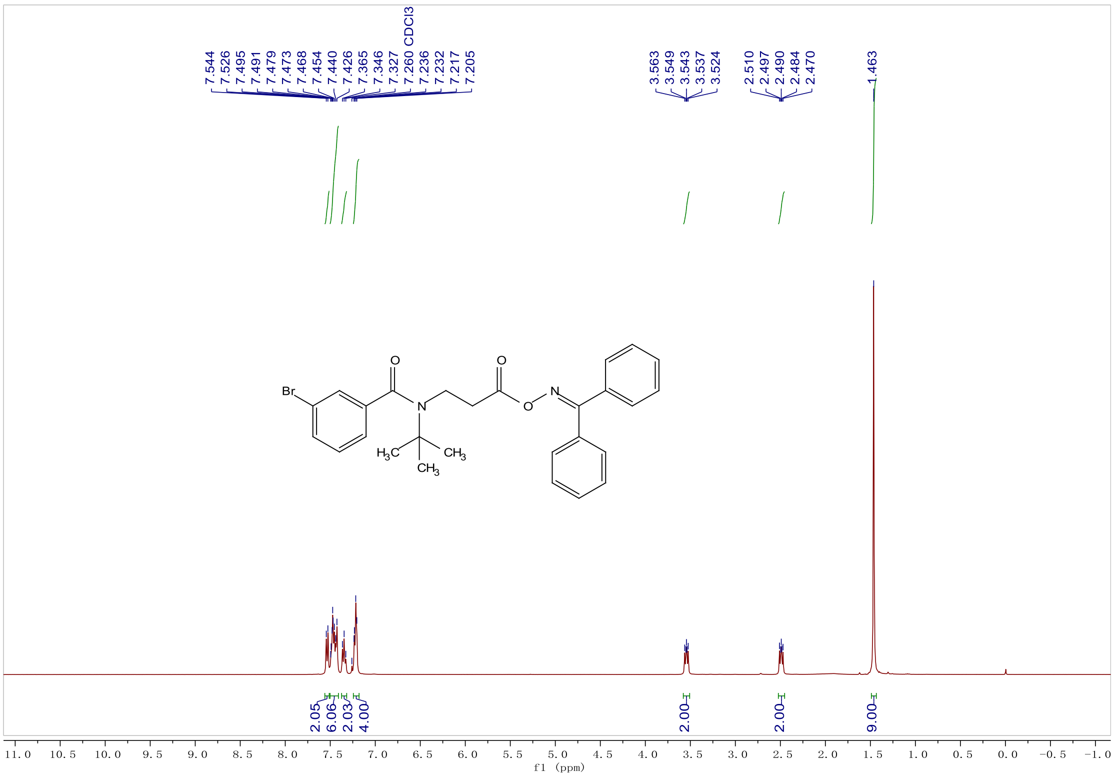

^{1}H-NMR for **1-27**

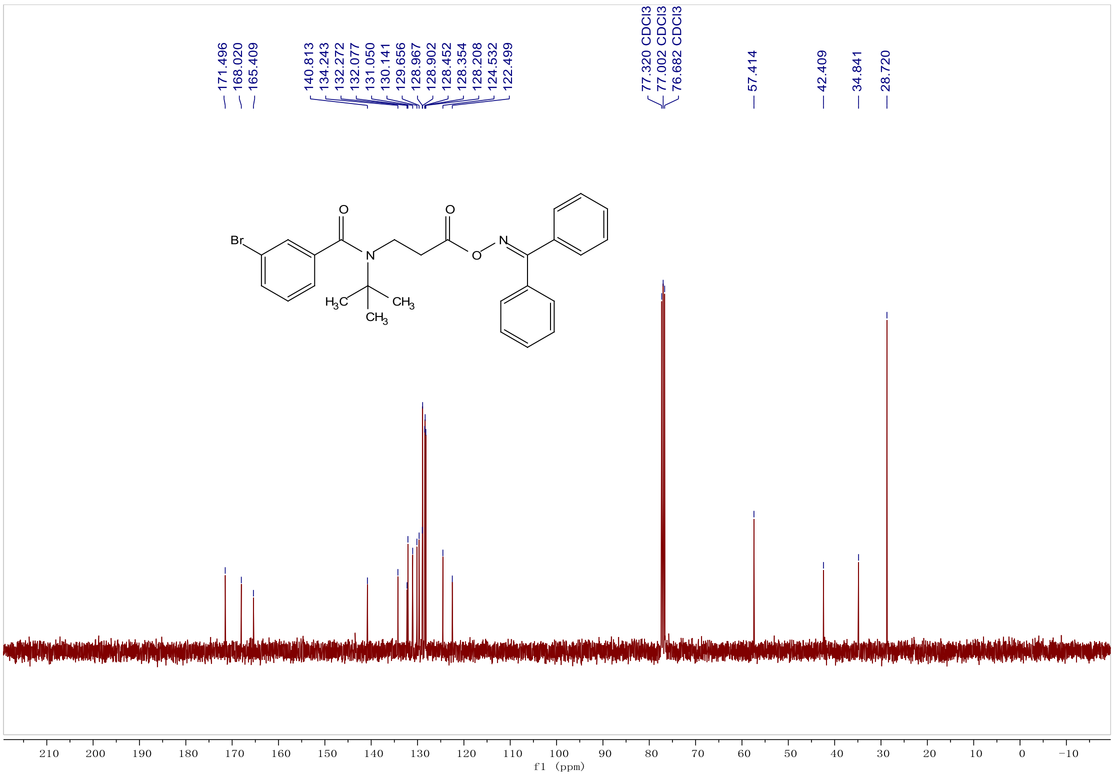

^{13}C-NMR for **1-27**

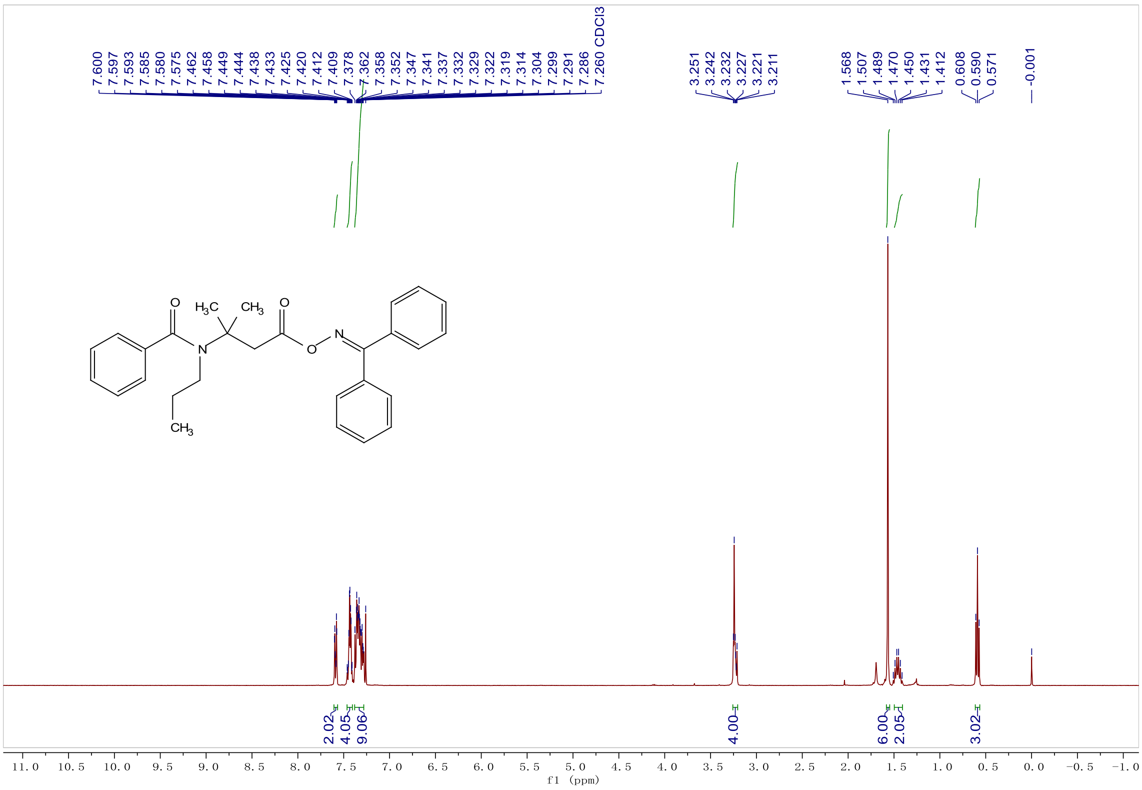

^{1}H-NMR for **1-28**

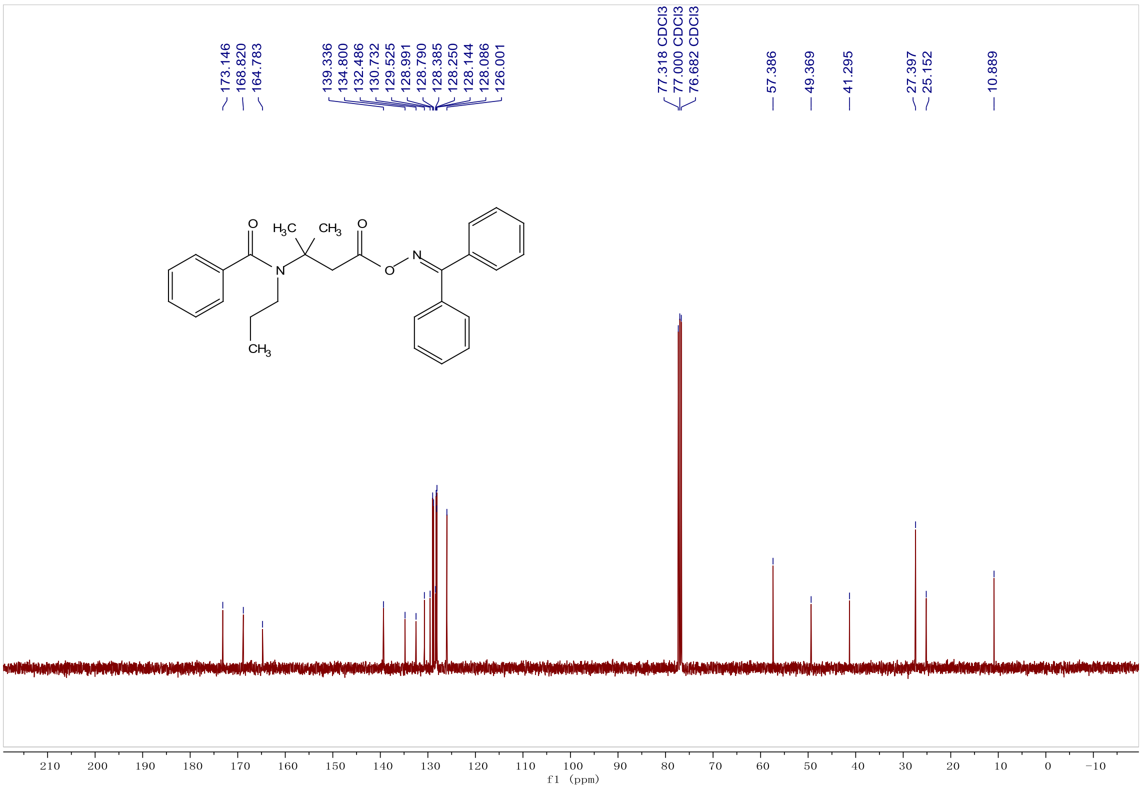

^{13}C−NMR for **1-28**

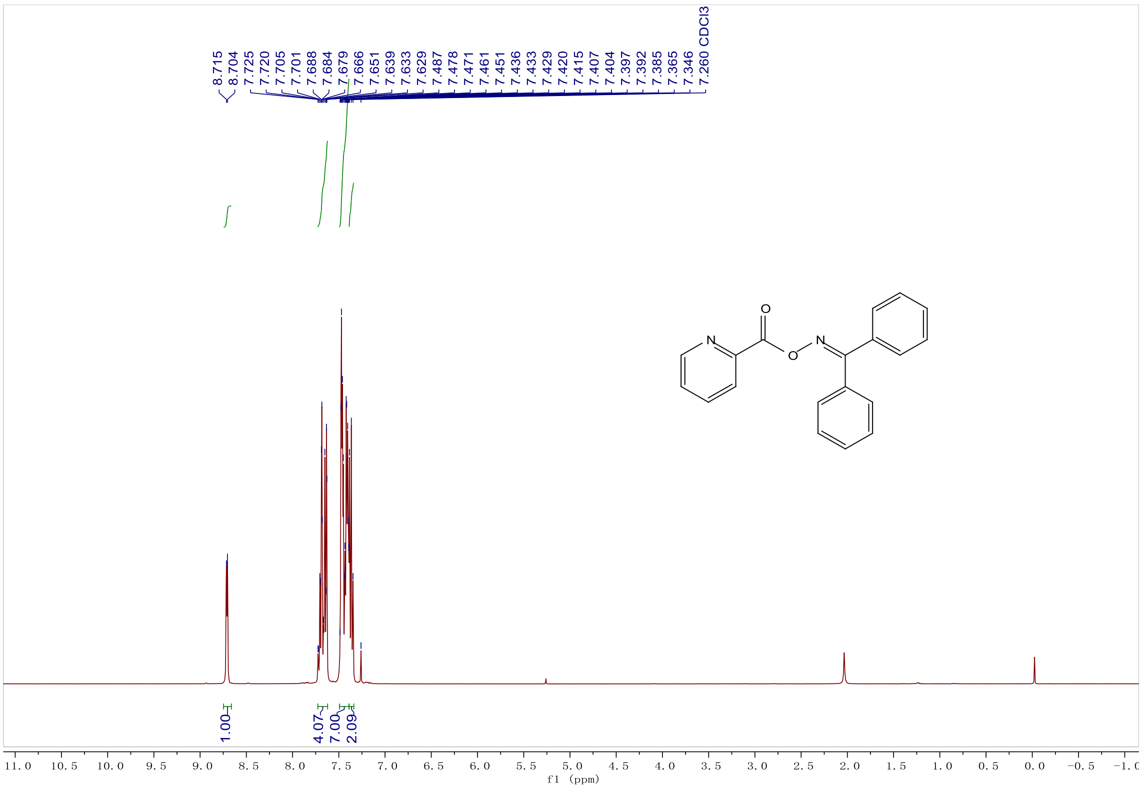

^{1}H-NMR for **5-1**

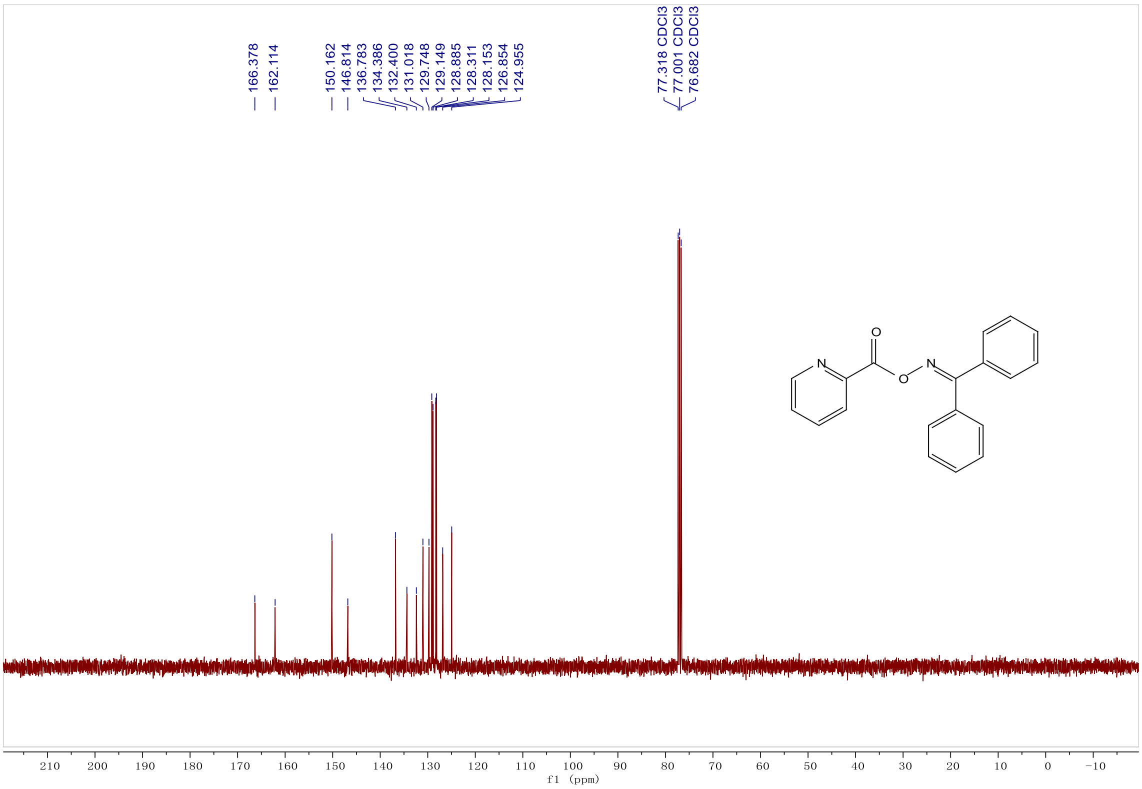

^{13}C−NMR for **5-1**

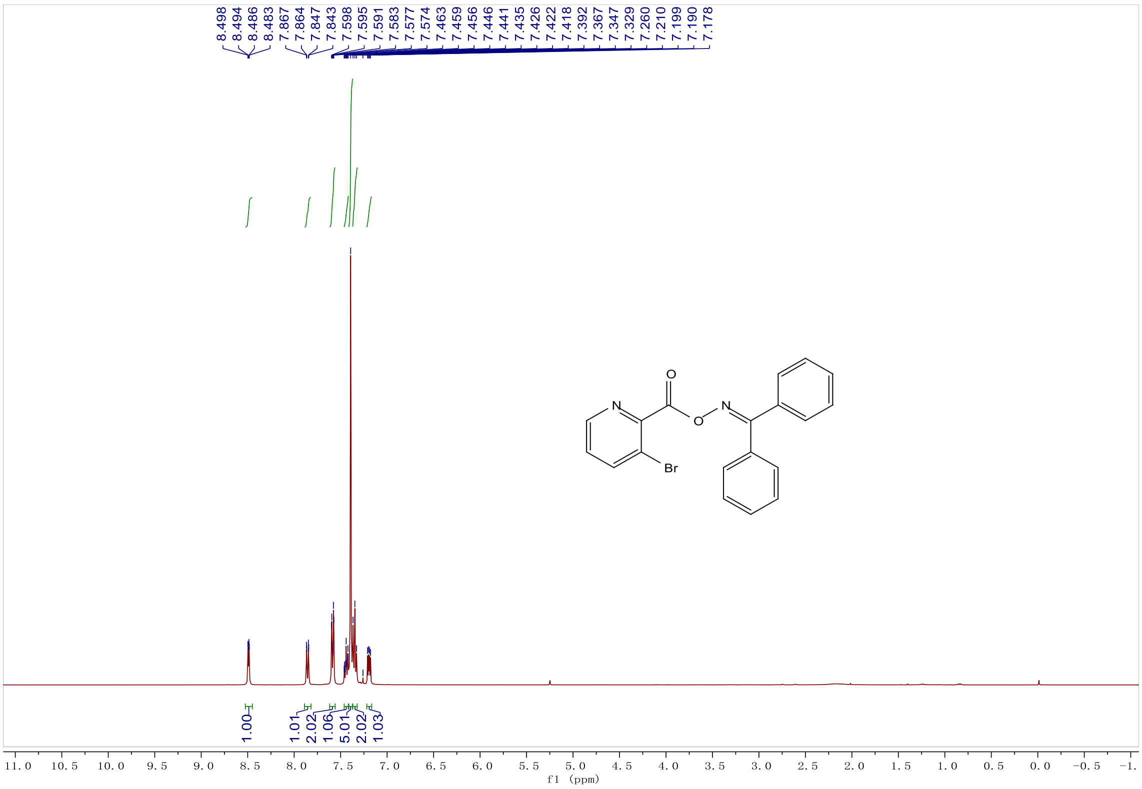

^{1}H-NMR for **5-2**

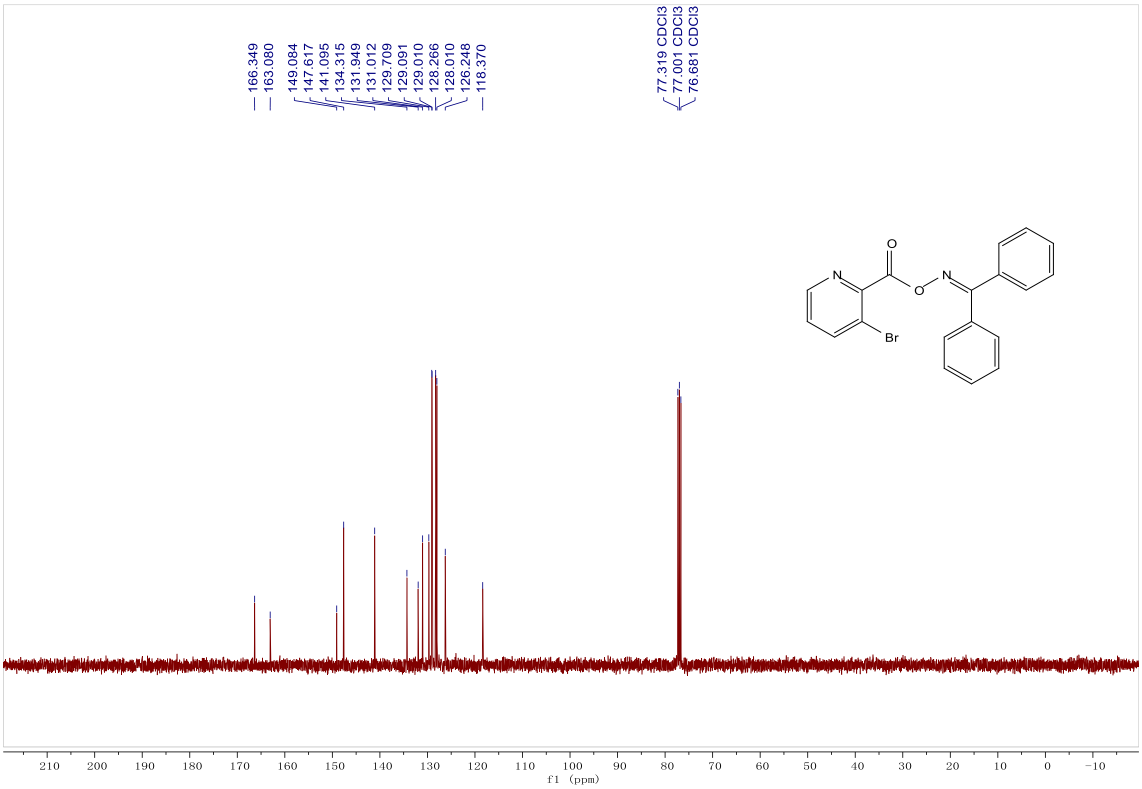

^{13}C−NMR for **5-2**

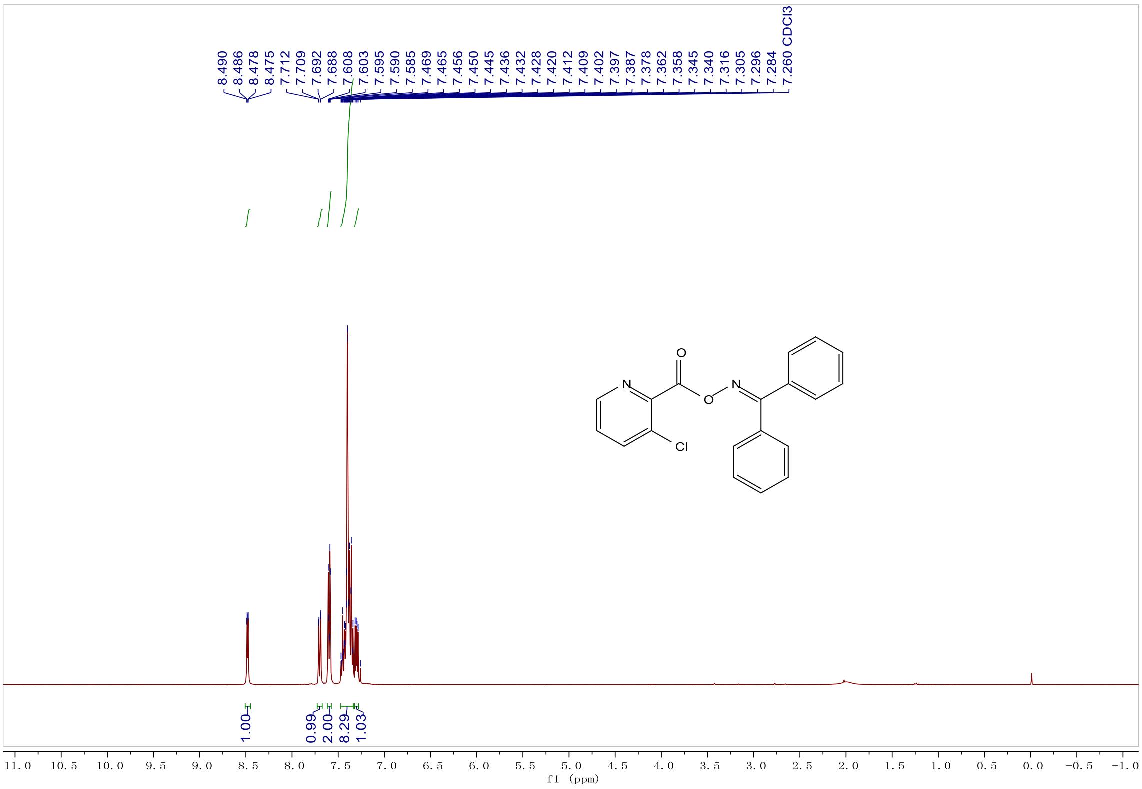

^{1}H-NMR for **5-3**

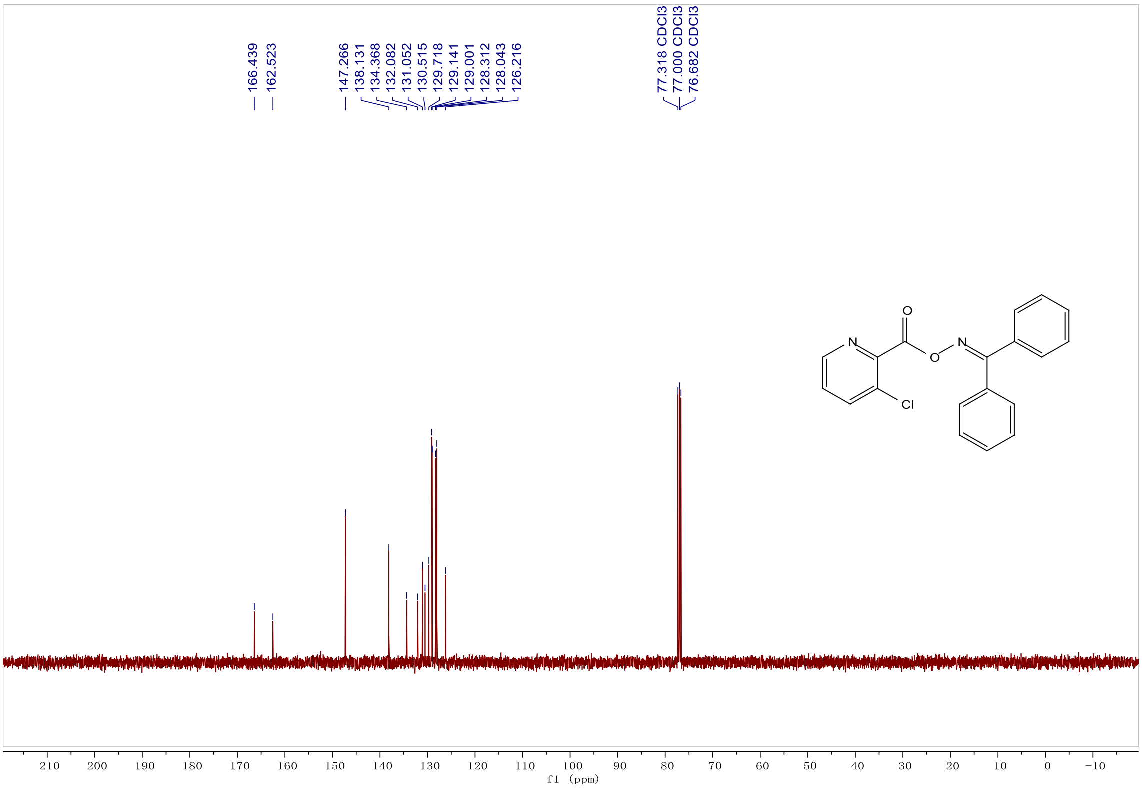

^{13}C−NMR for **5-3**

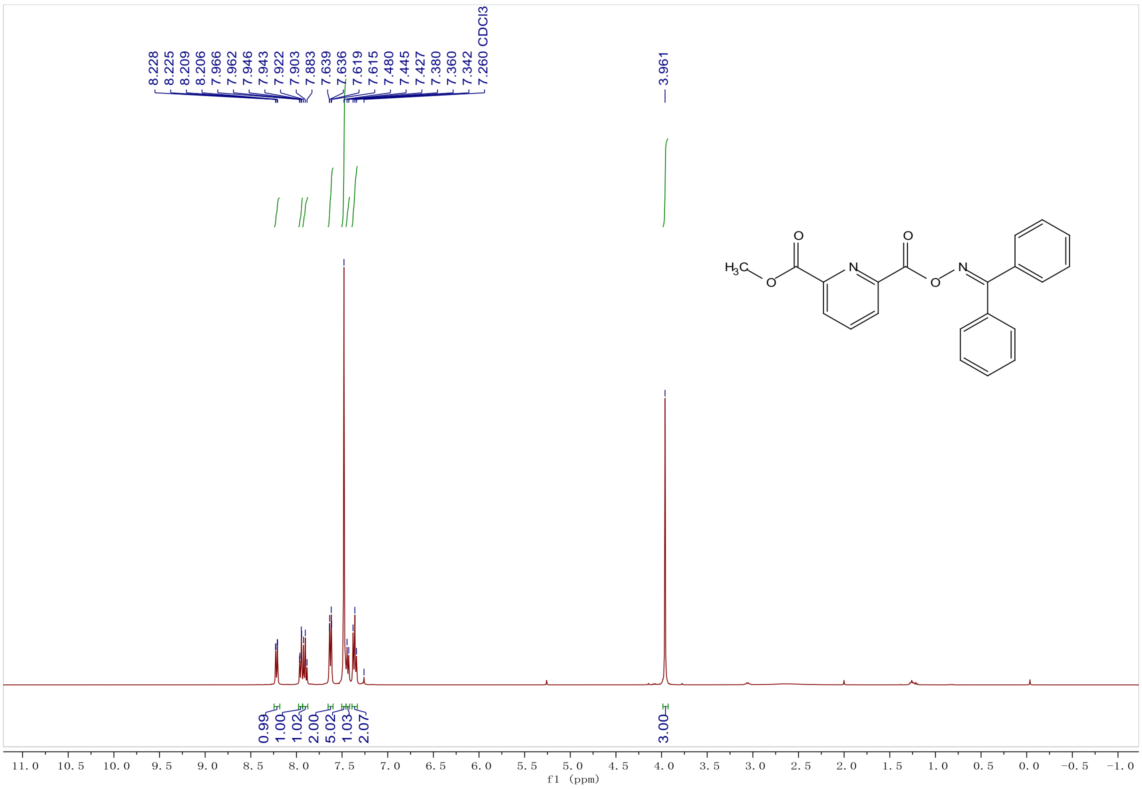

^{1}H-NMR for **5-4**

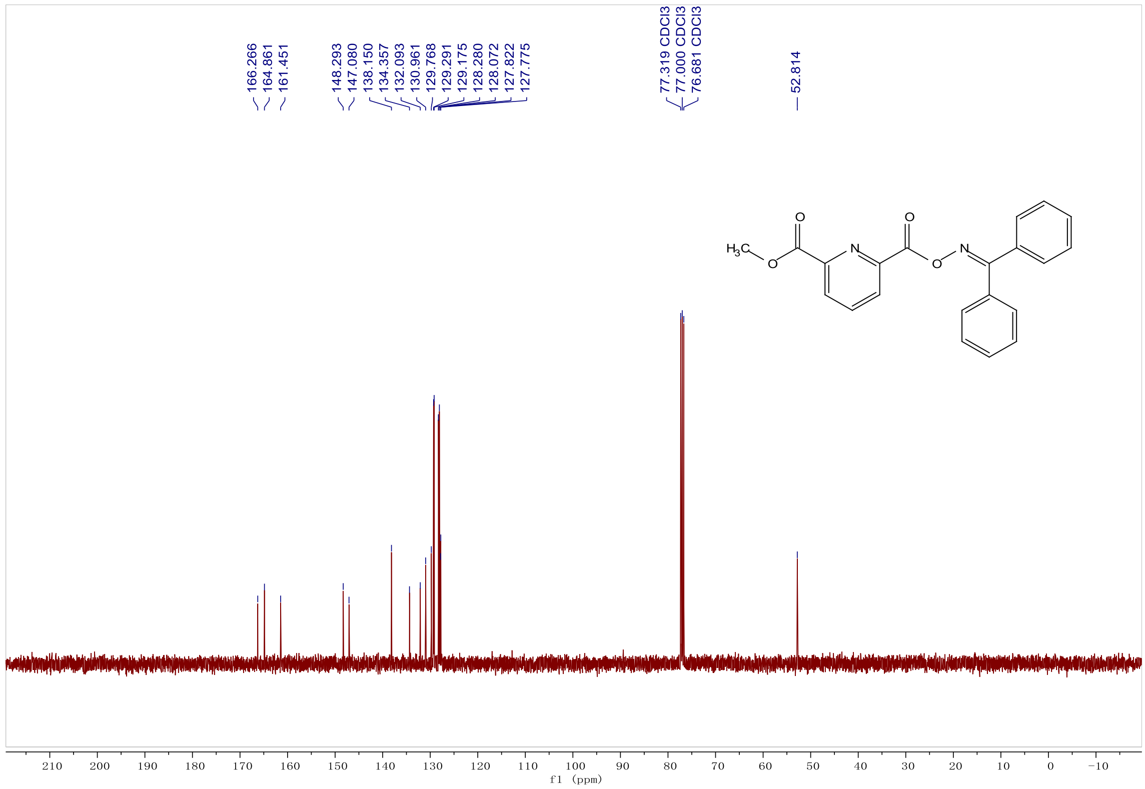

^{13}C−NMR for **5-4**

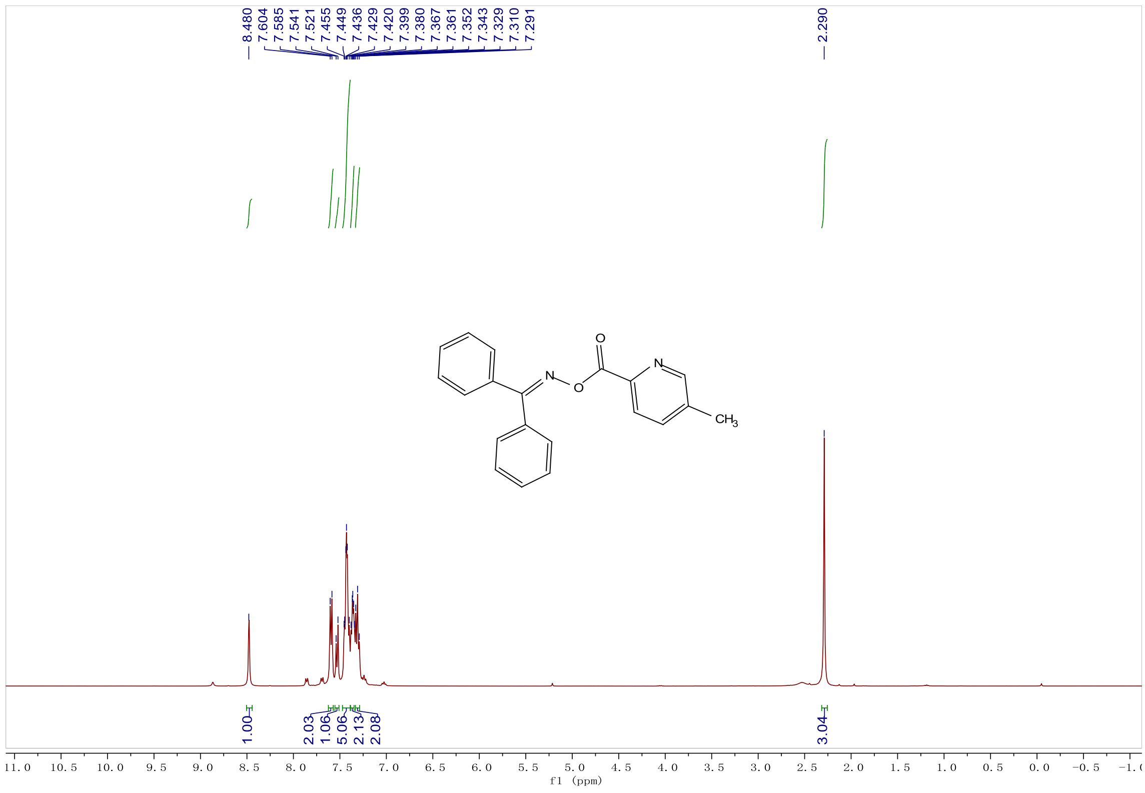

^{1}H-NMR for **5-5**

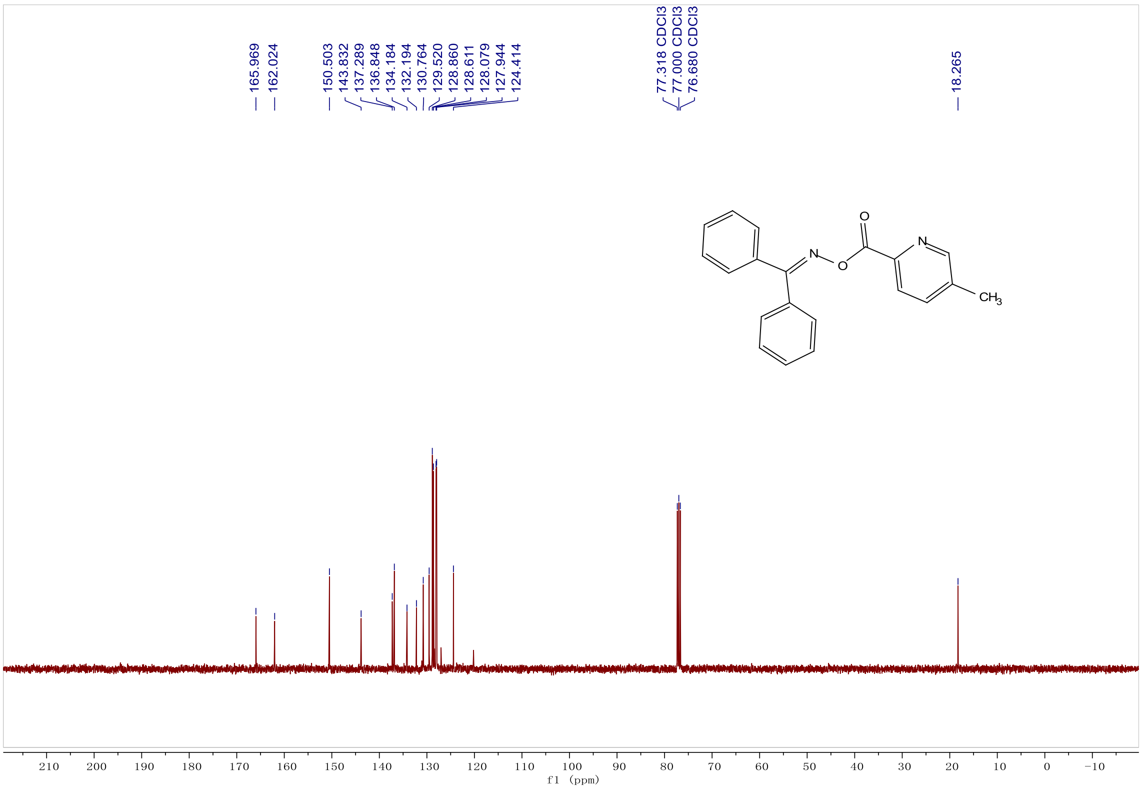

^{13}C−NMR for **5-5**

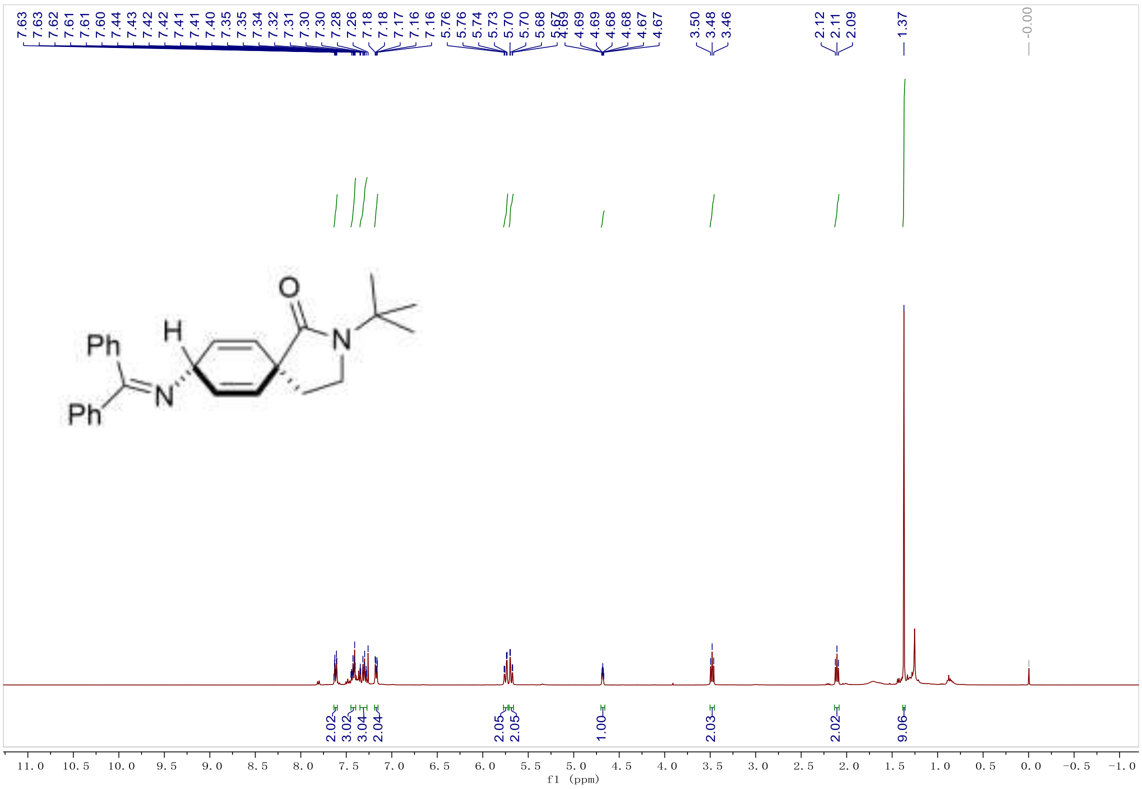

^{1}H-NMR for trans-**2-1**

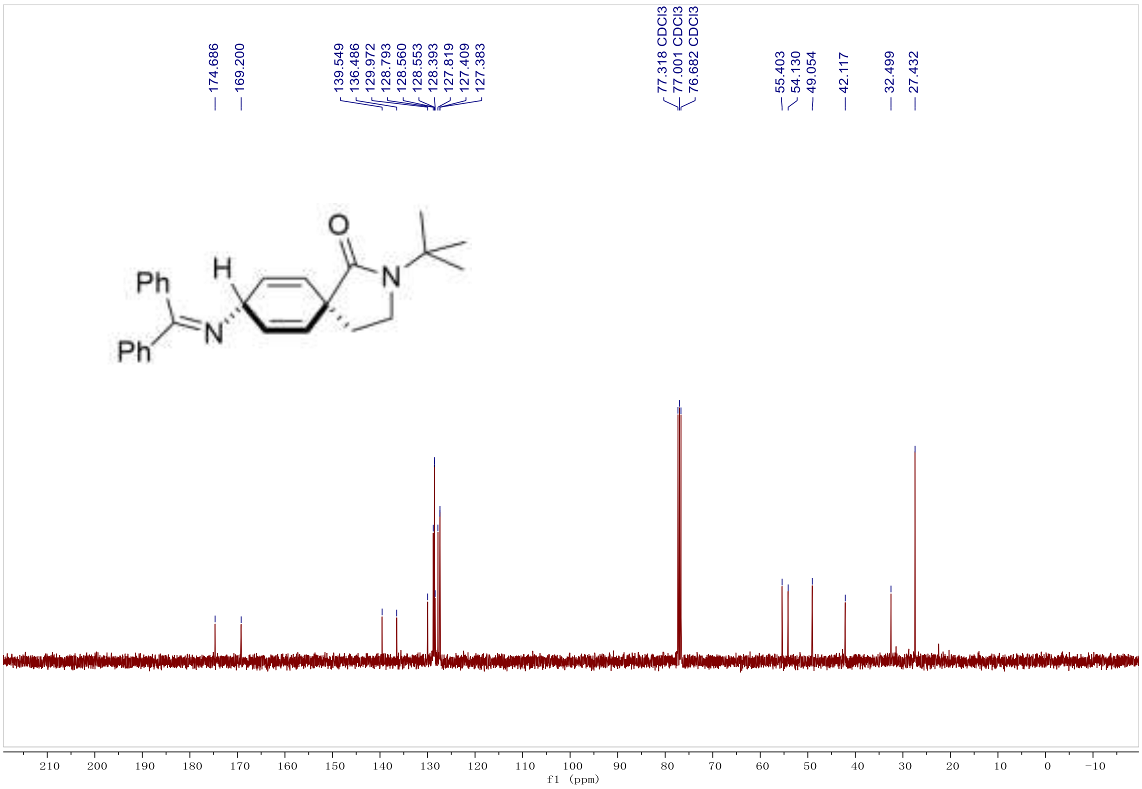

^{13}C−NMR for trans-**2-1**

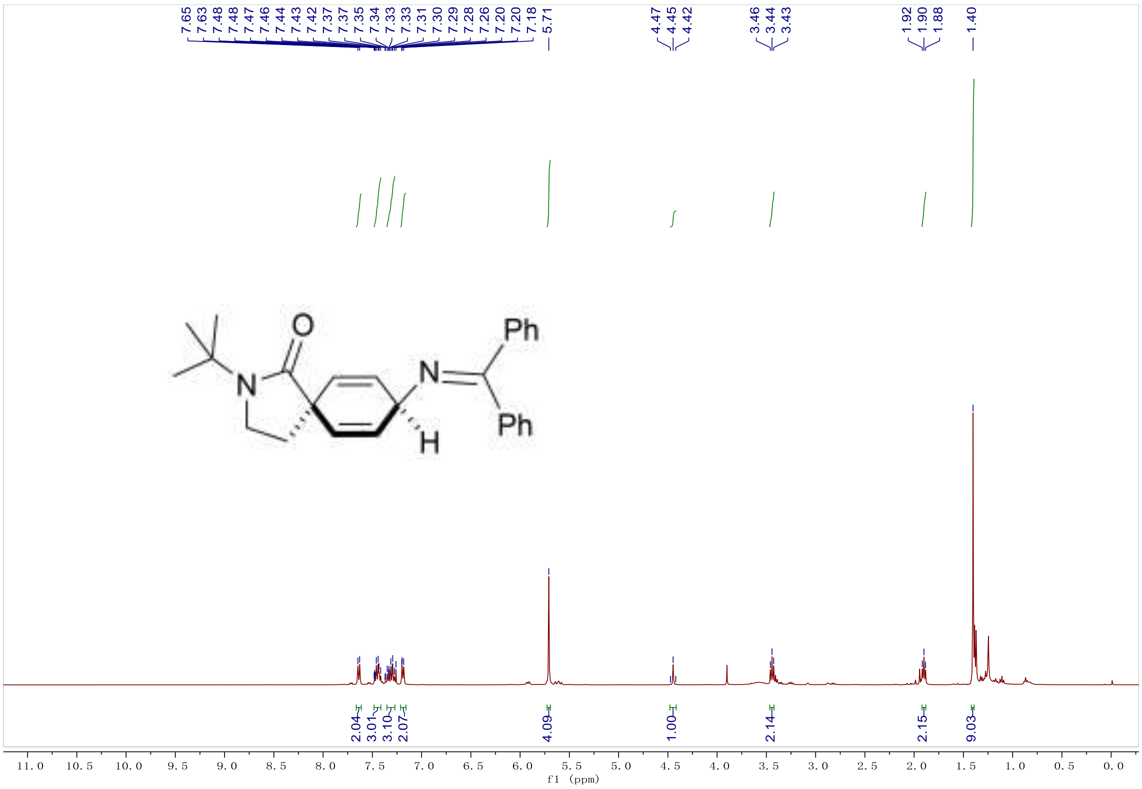

^{1}H-NMR for cis-**2-1**

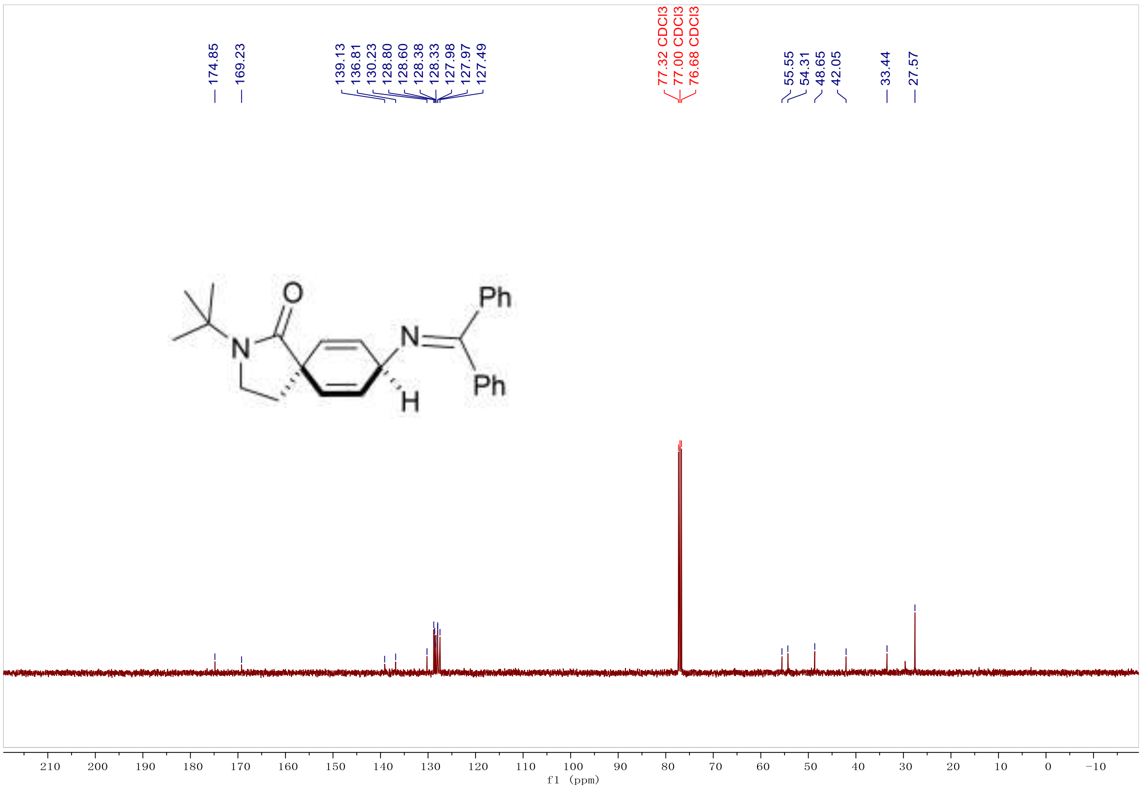

^{13}C−NMR for cis-**2-1**

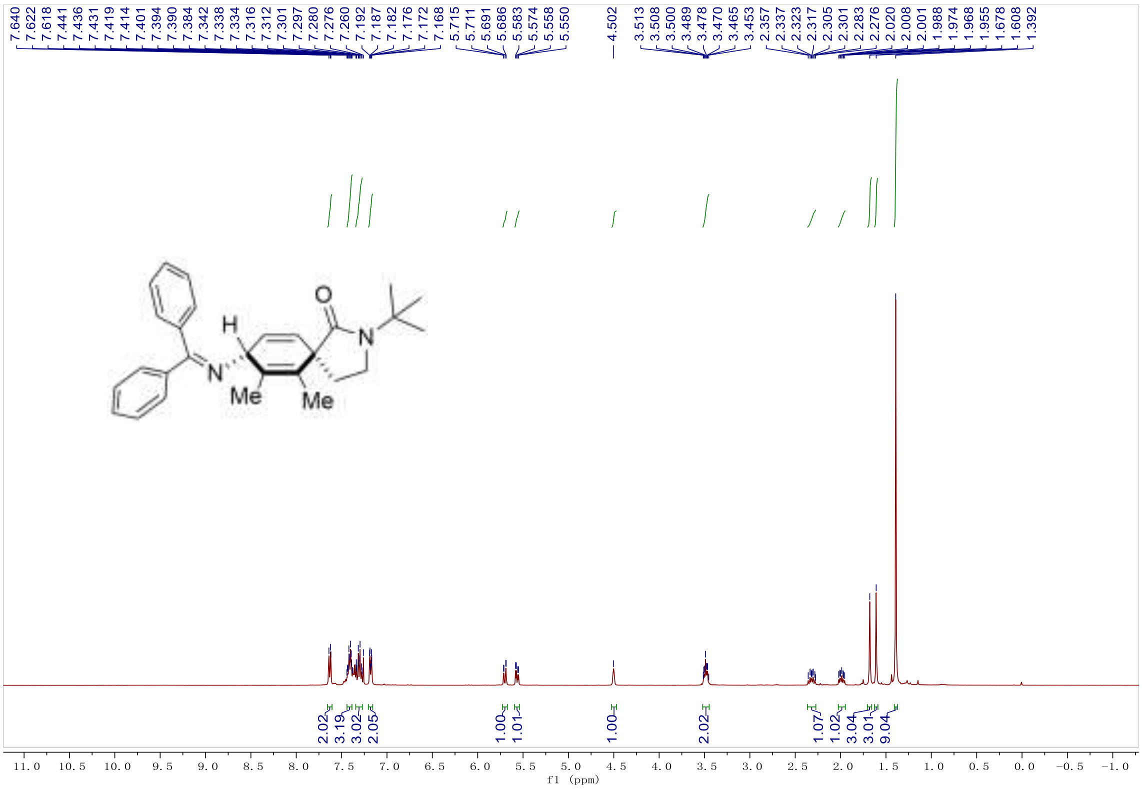

^{1}H-NMR for trans-**2-2**

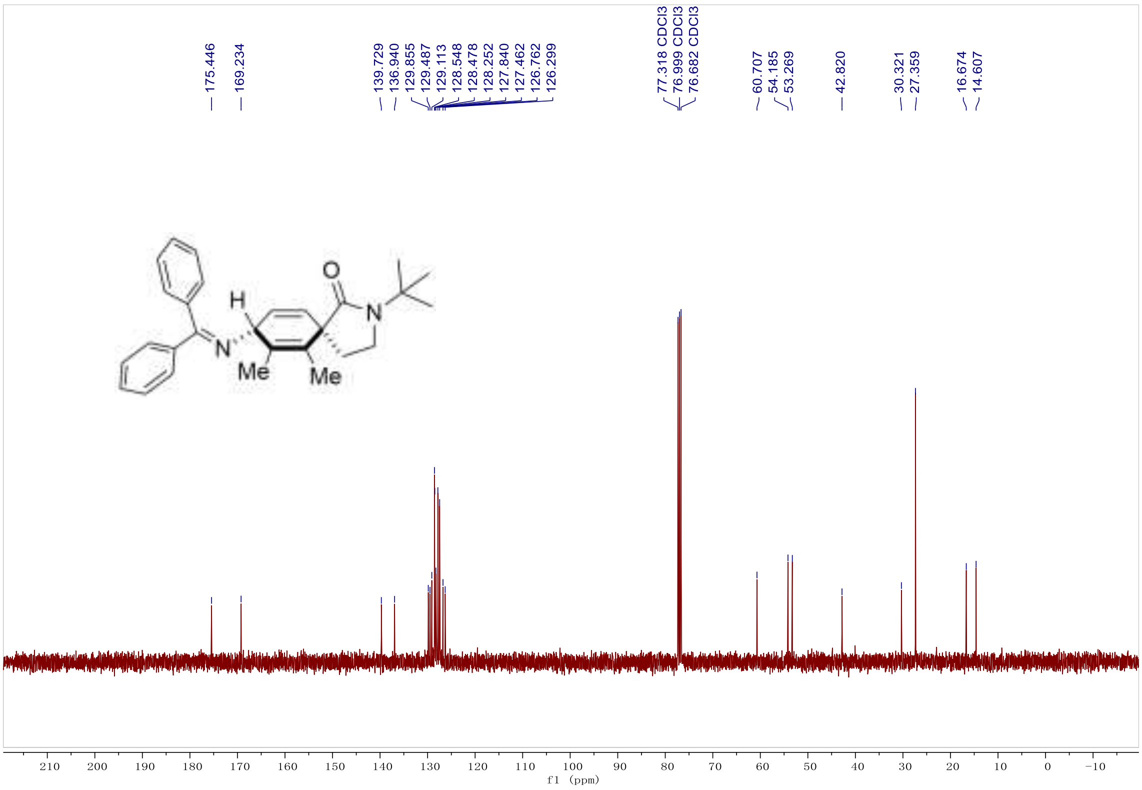

^{13}C−NMR for trans-**2-2**

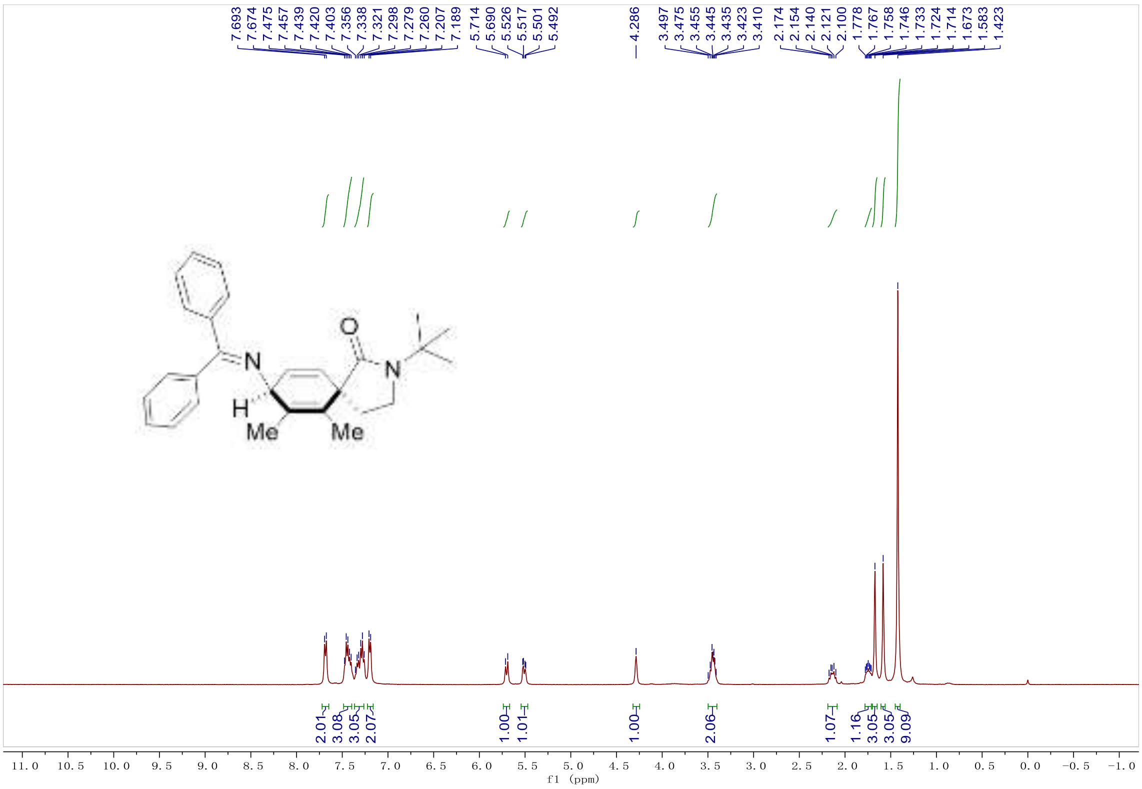

^{1}H-NMR for cis-**2-2**

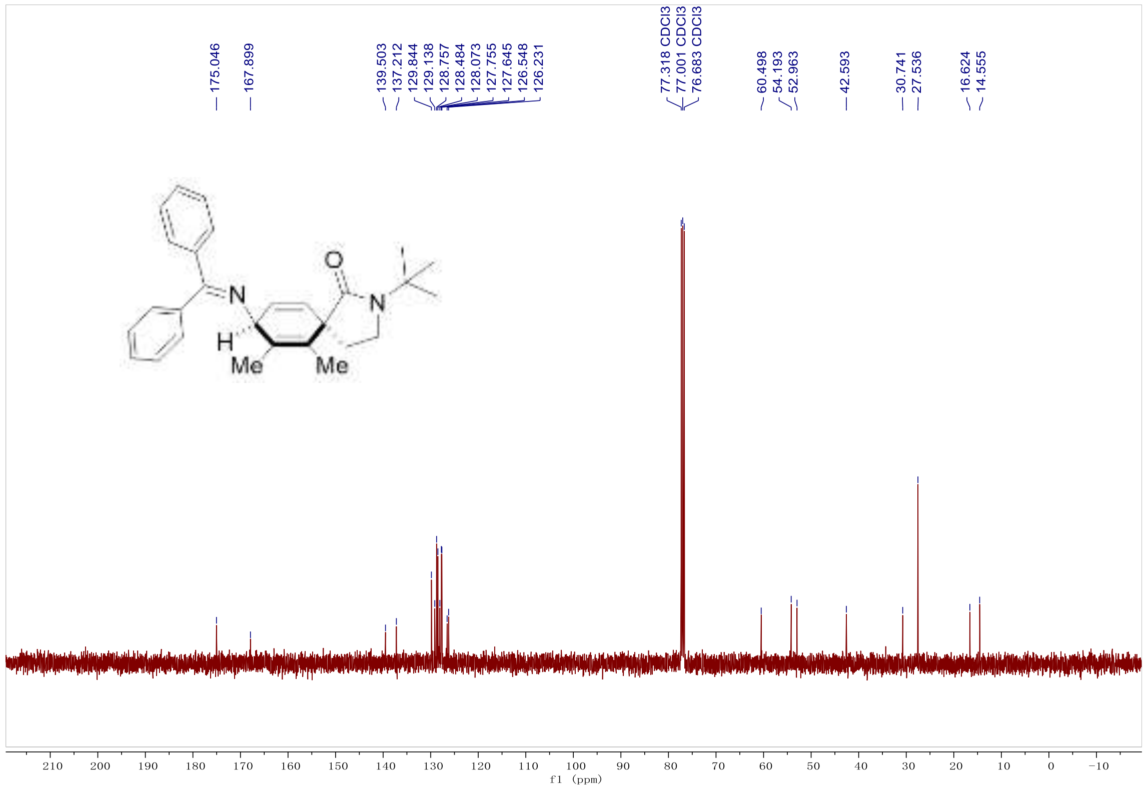

^{13}C−NMR for cis-**2-2**

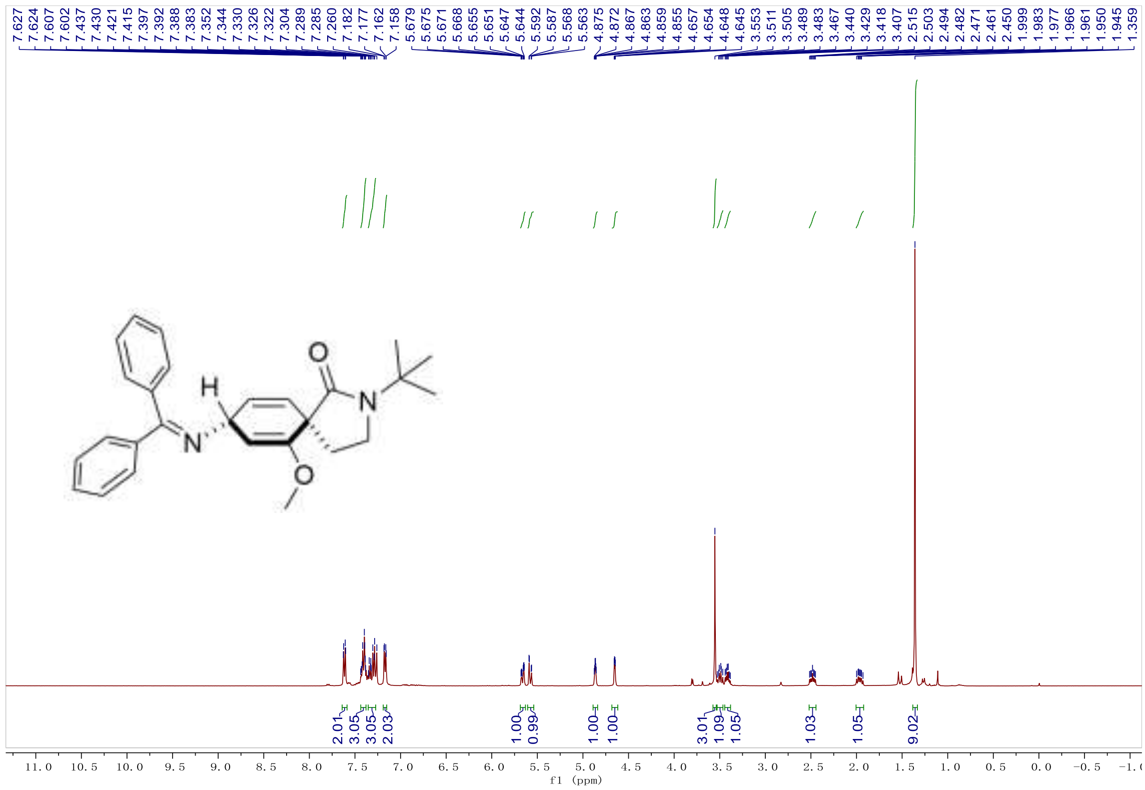

^{1}H-NMR for trans-**2-3**

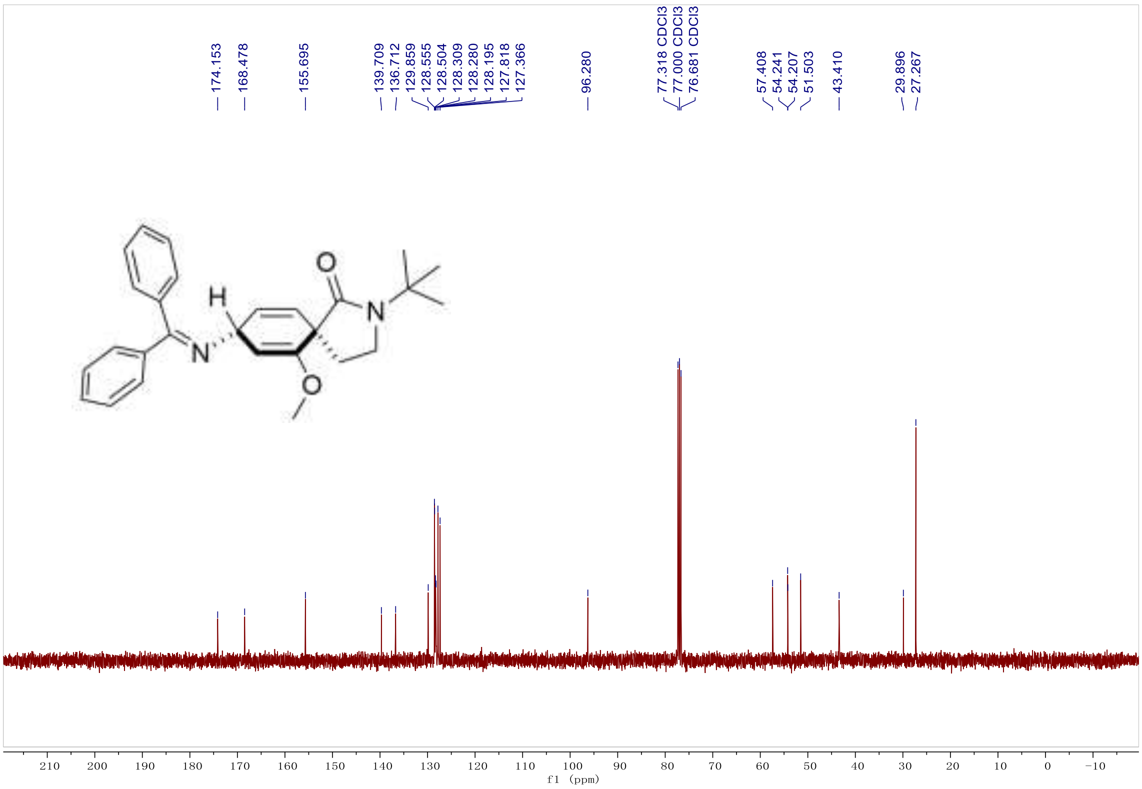

$^{13}C-NMR$ for trans-**2-3**

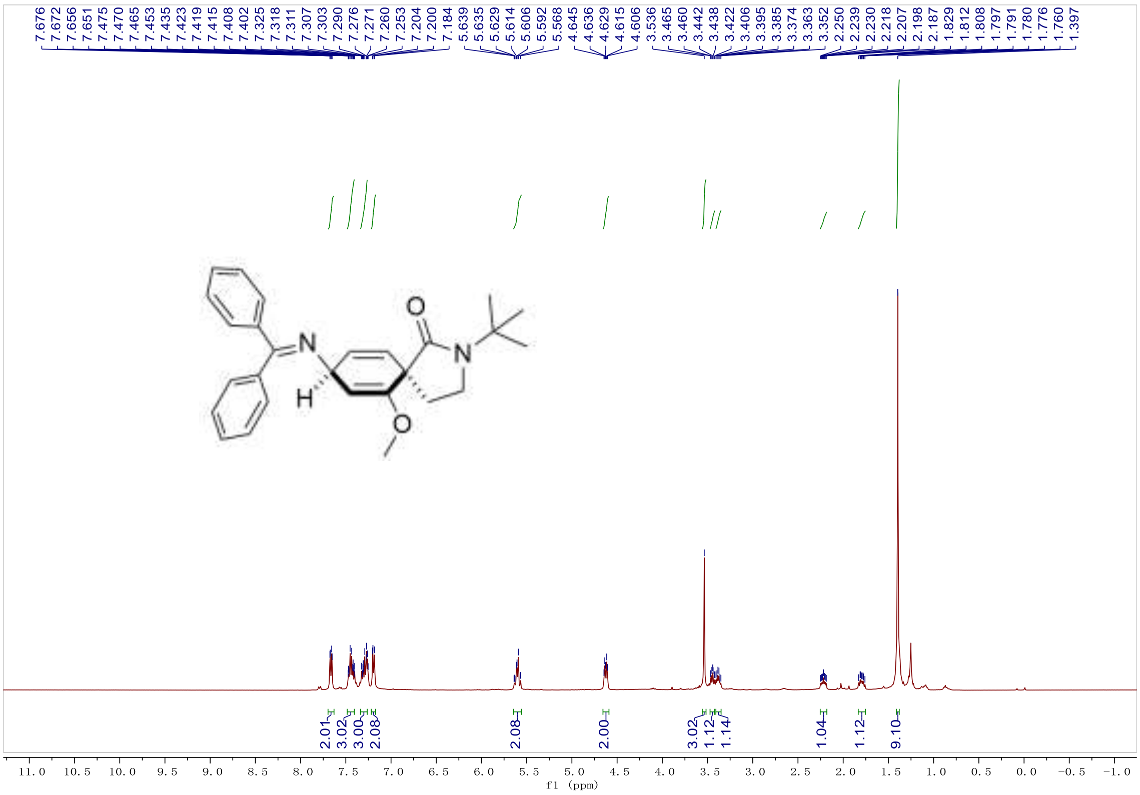

$^{1}H-NMR$ for cis-**2-3**

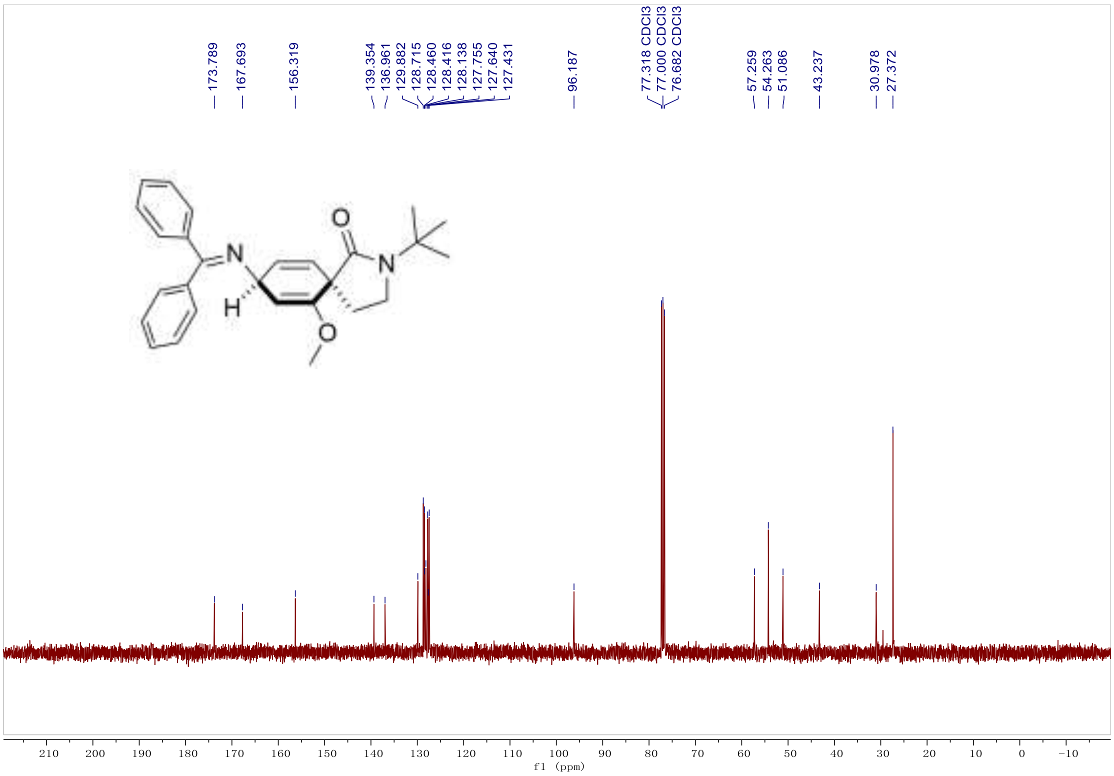

^{13}C−NMR for cis-**2-3**

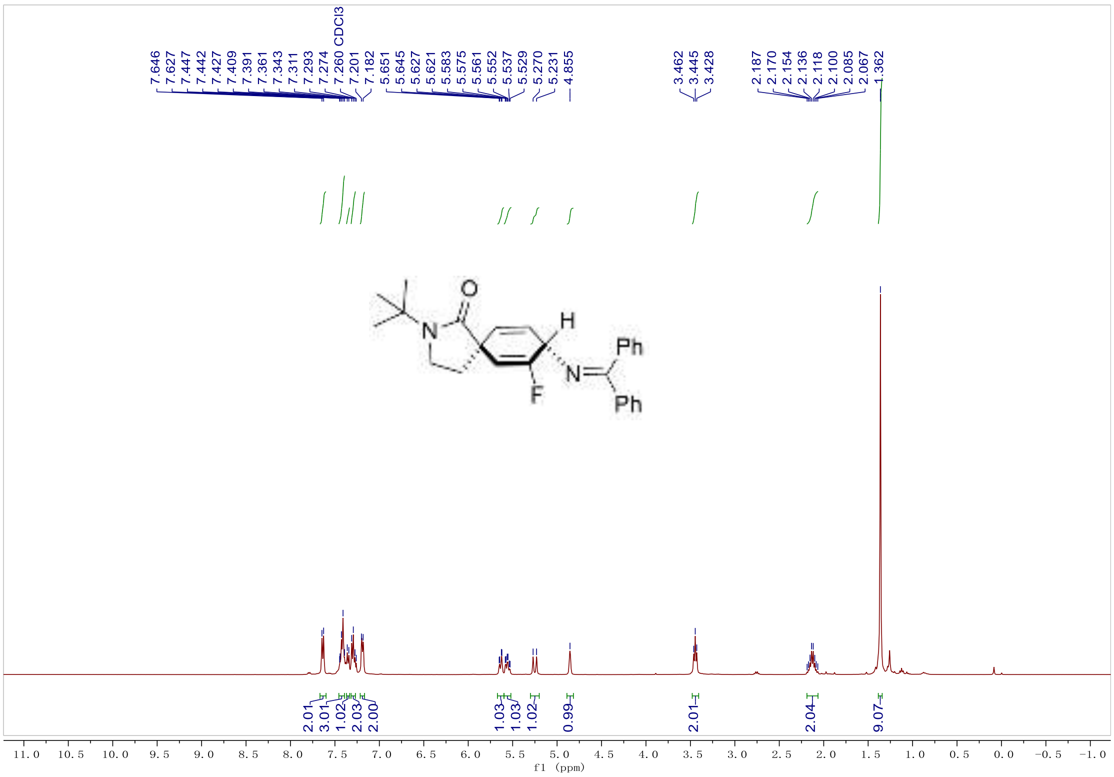

^{1}H-NMR for trans-**2-4**

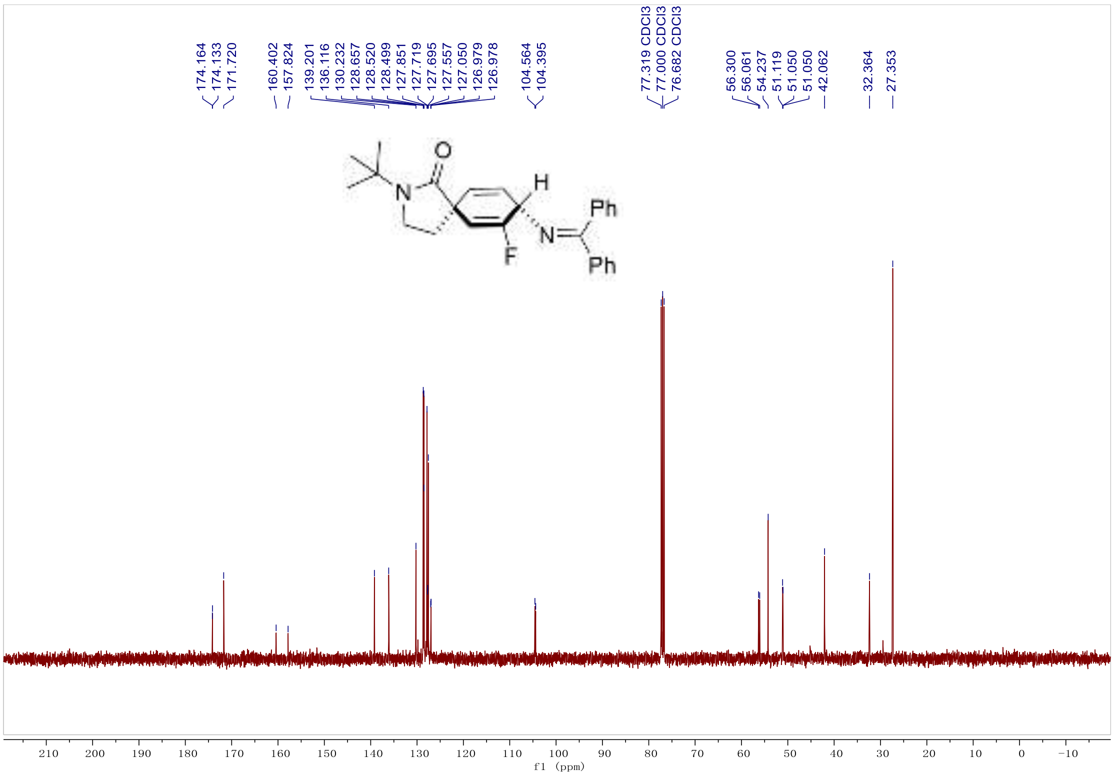

^{13}C-NMR for trans-**2-4**

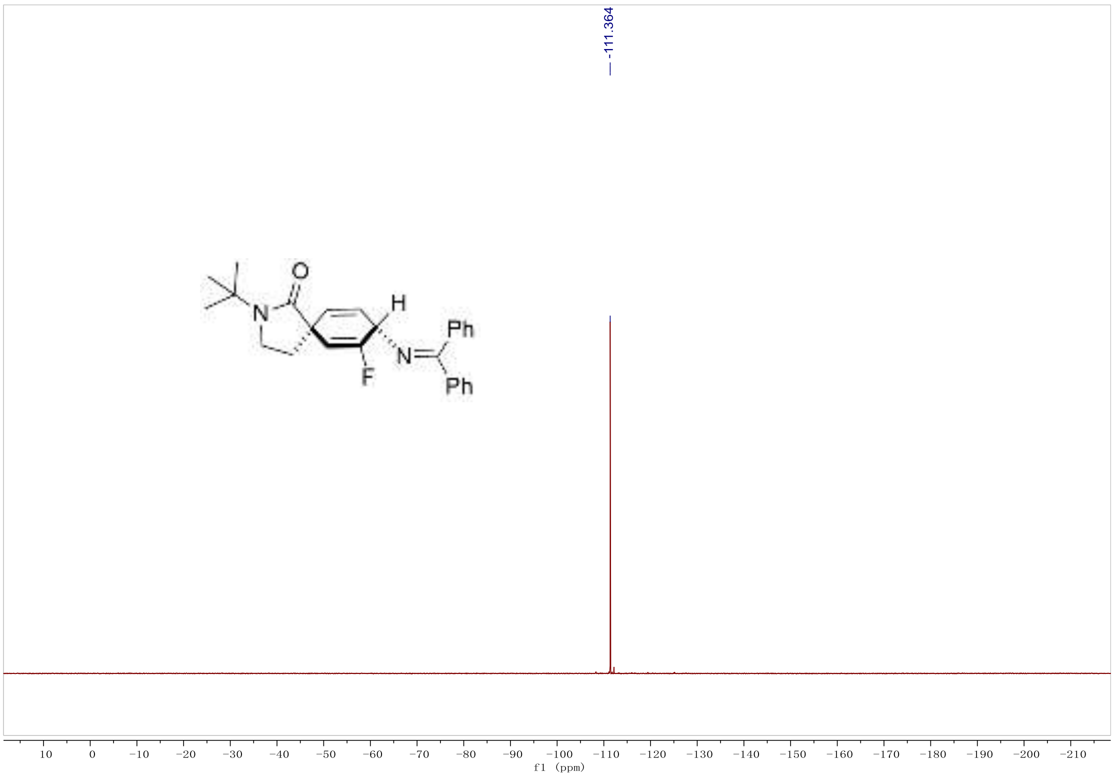

^{19}F-NMR for trans-**2-4**

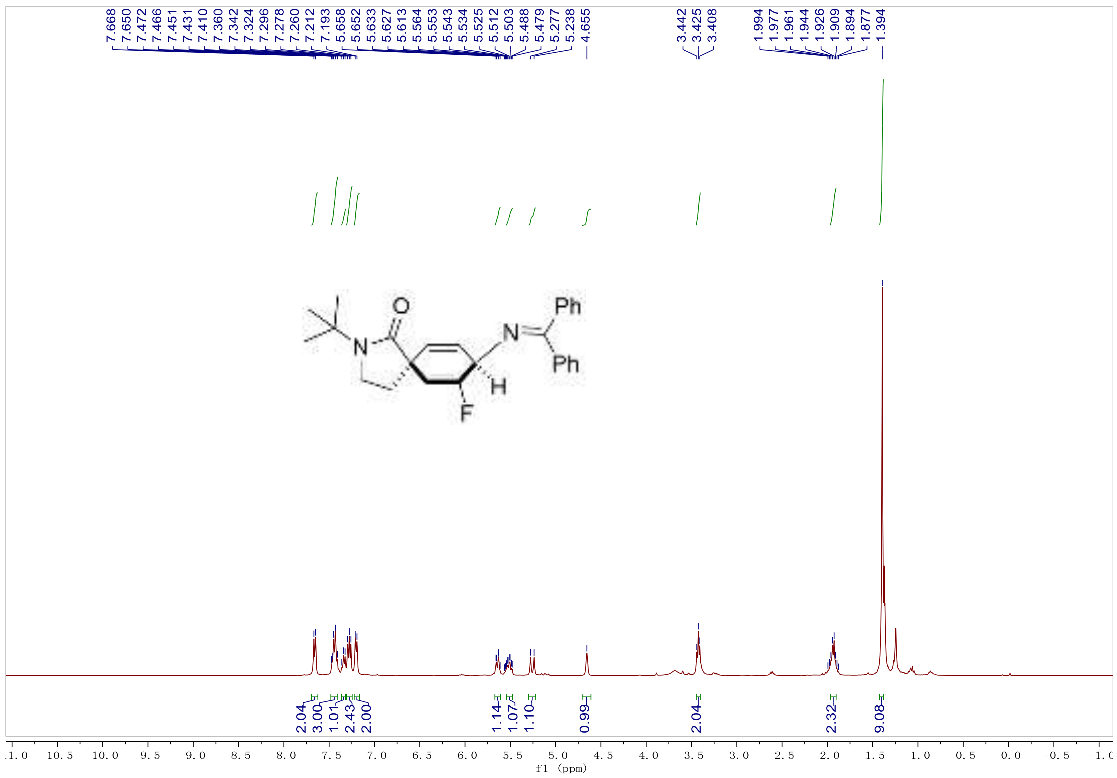

^{1}H-NMR for cis-**2-4**

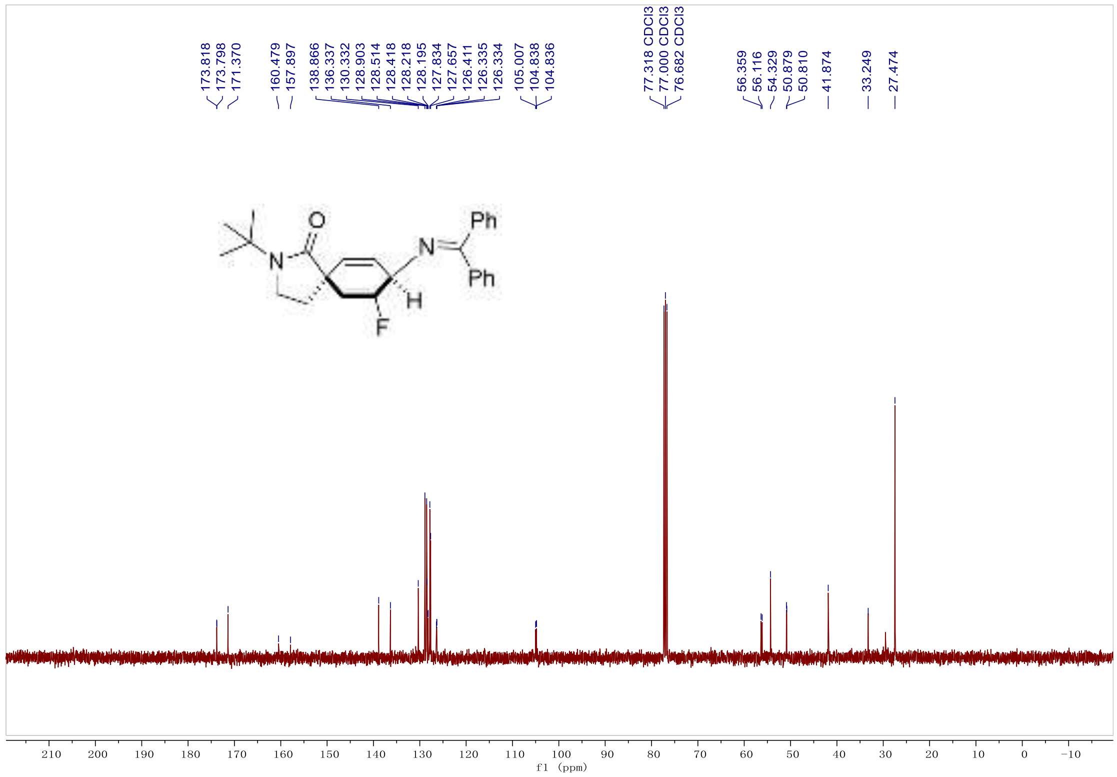

^{13}C-NMR for cis-**2-4**

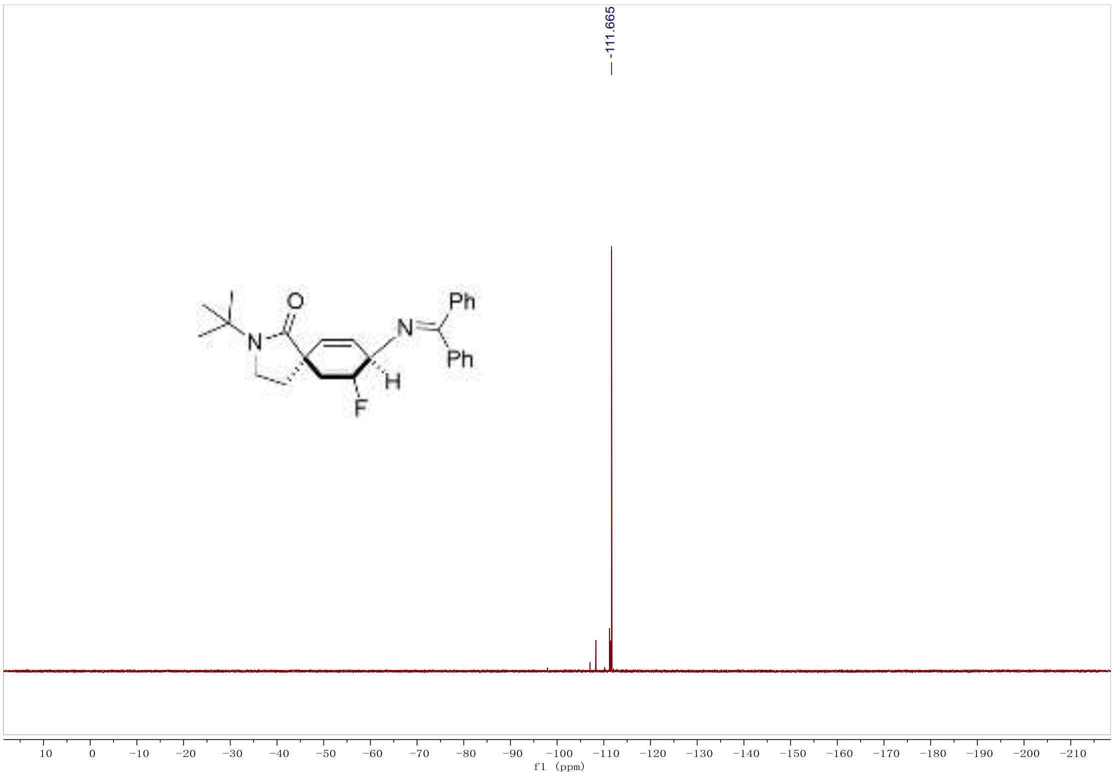

^{19}F-NMR for cis-**2-4**

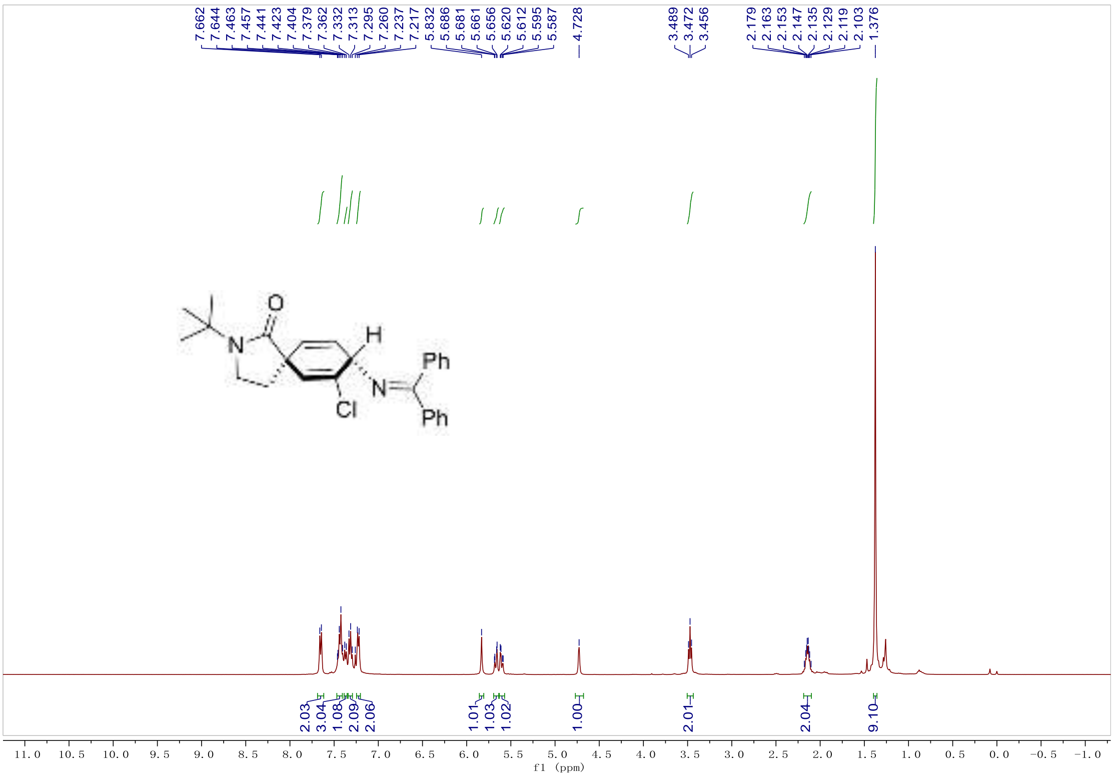

^{1}H-NMR for trans-**2-5**

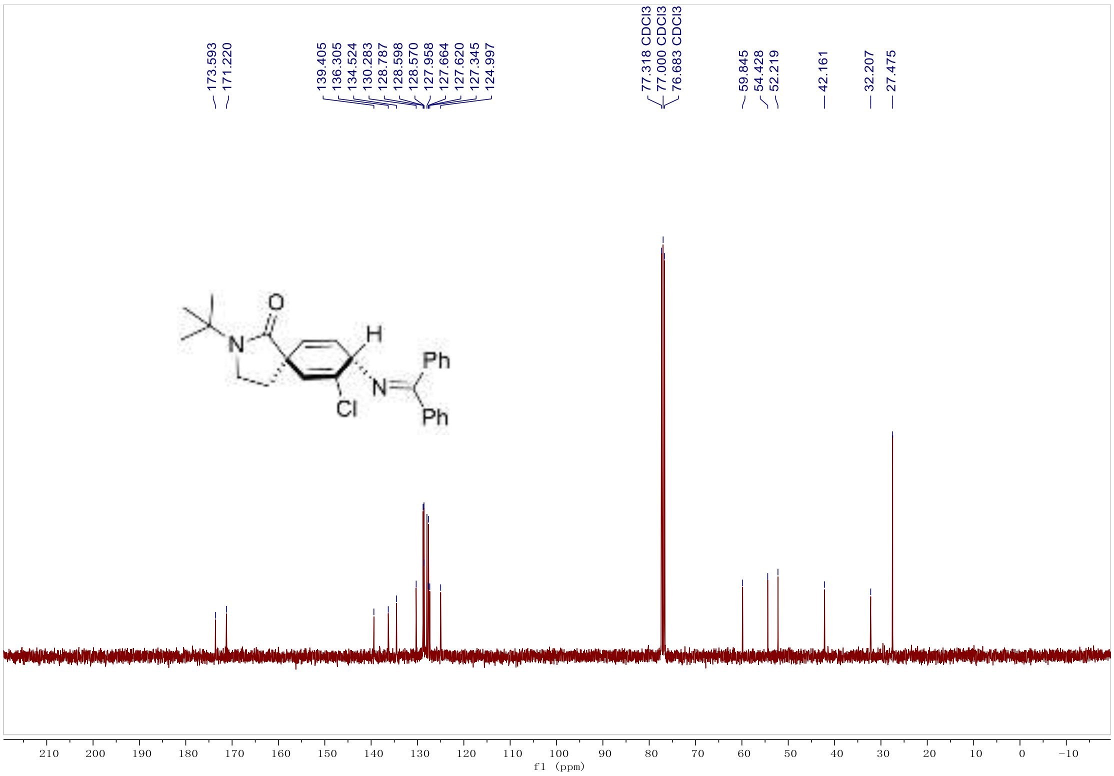

^{13}C-NMR for trans-**2-5**

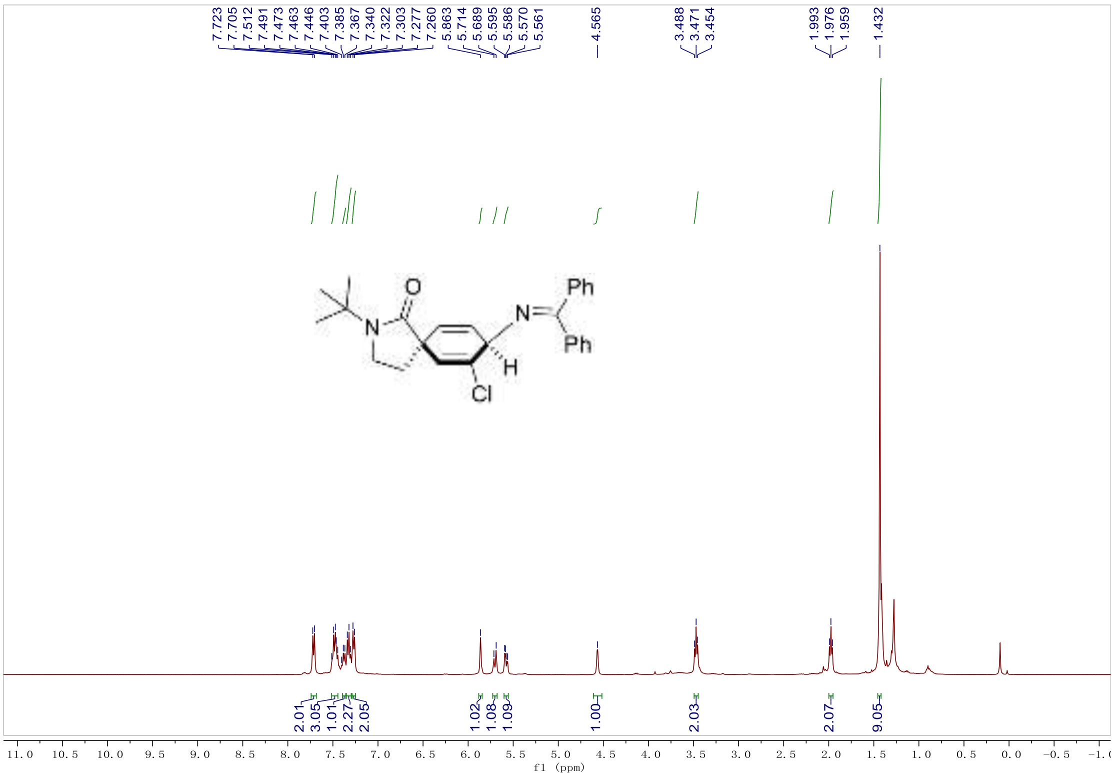

^{1}H-NMR for cis-**2-5**

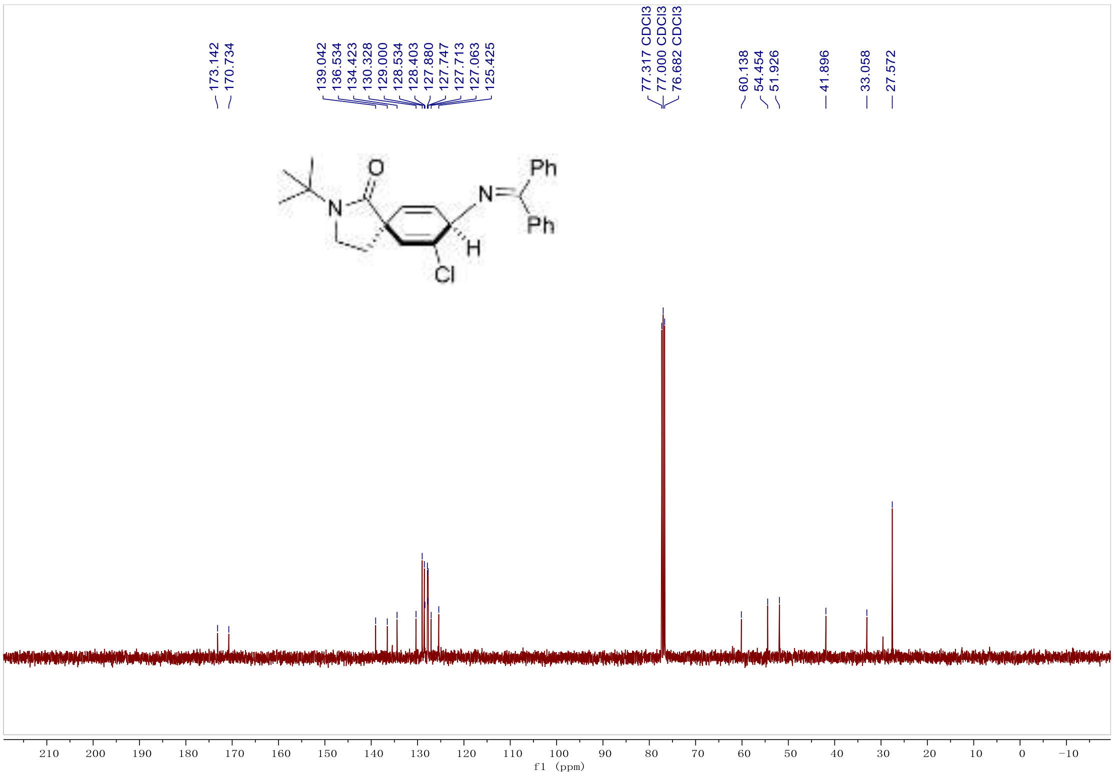

^{13}C-NMR for cis-**2-5**

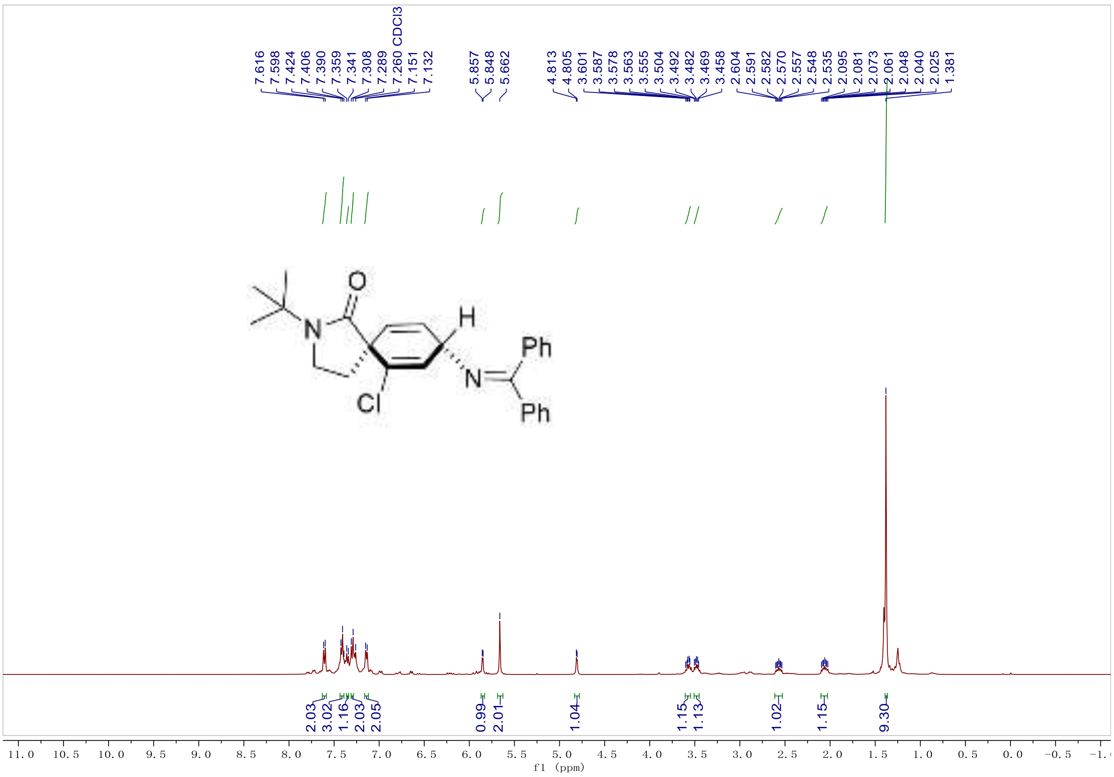

^{1}H-NMR for trans-**2-6**

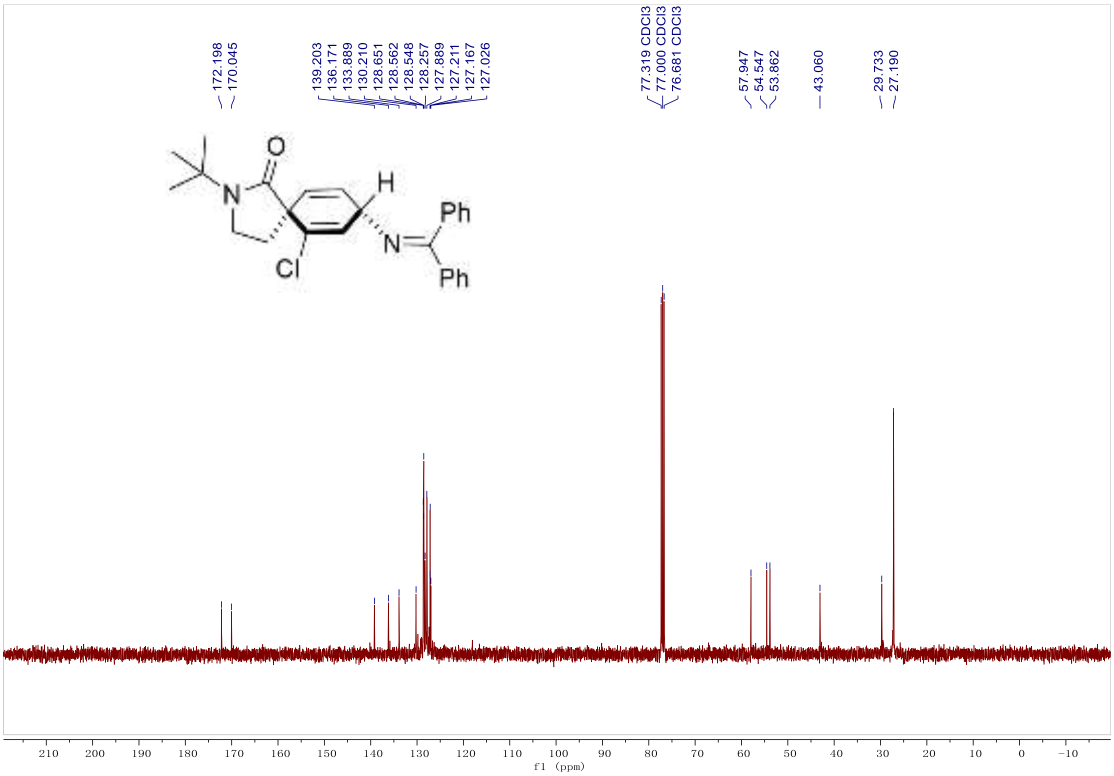

^{13}C-NMR for trans-**2-6**

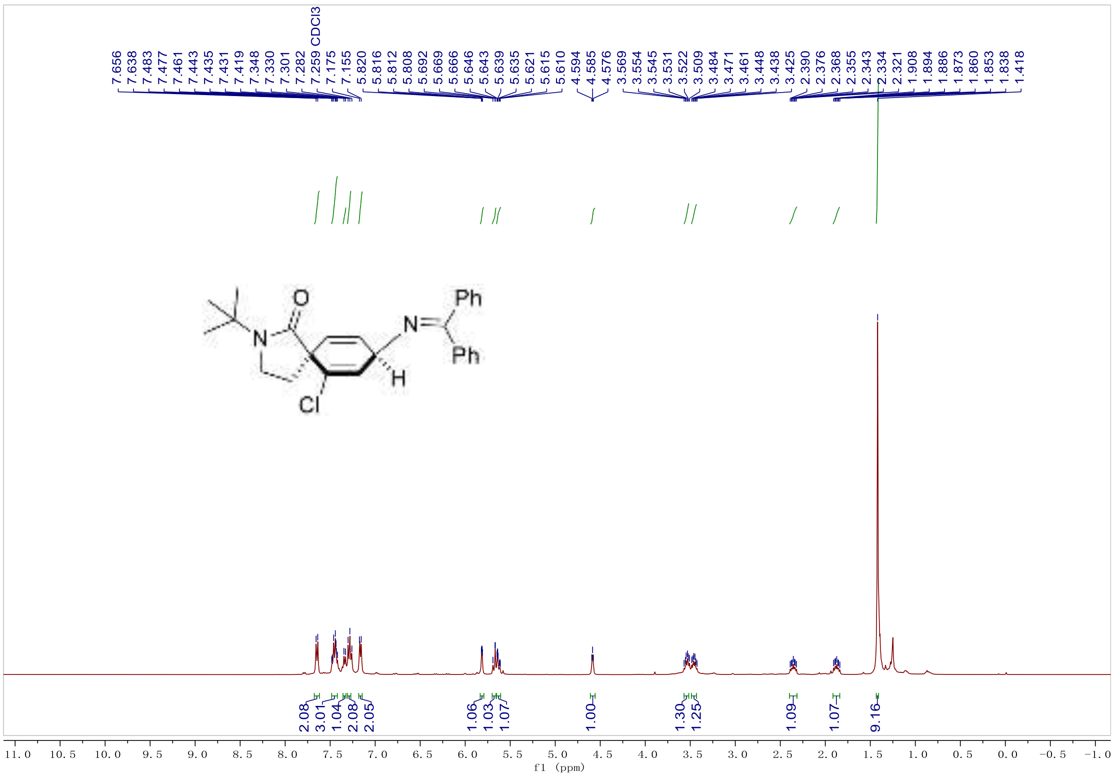

^{1}H-NMR for cis-**2-6**

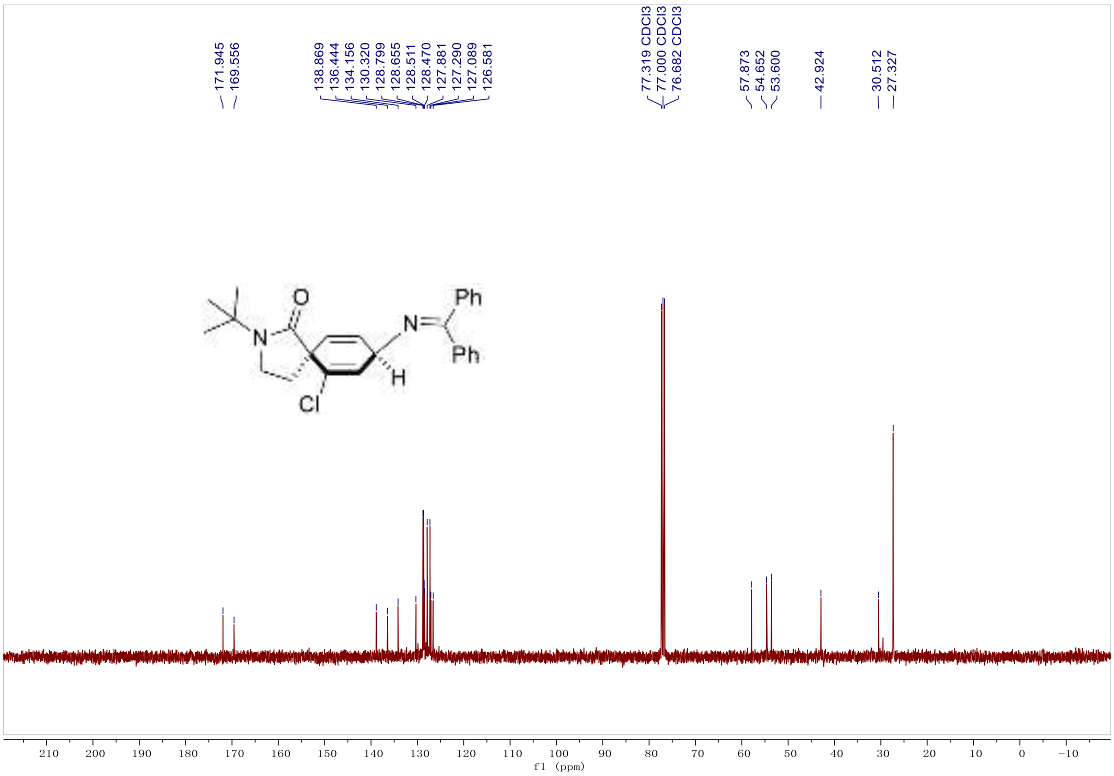

^{13}C-NMR for cis-**2-6**

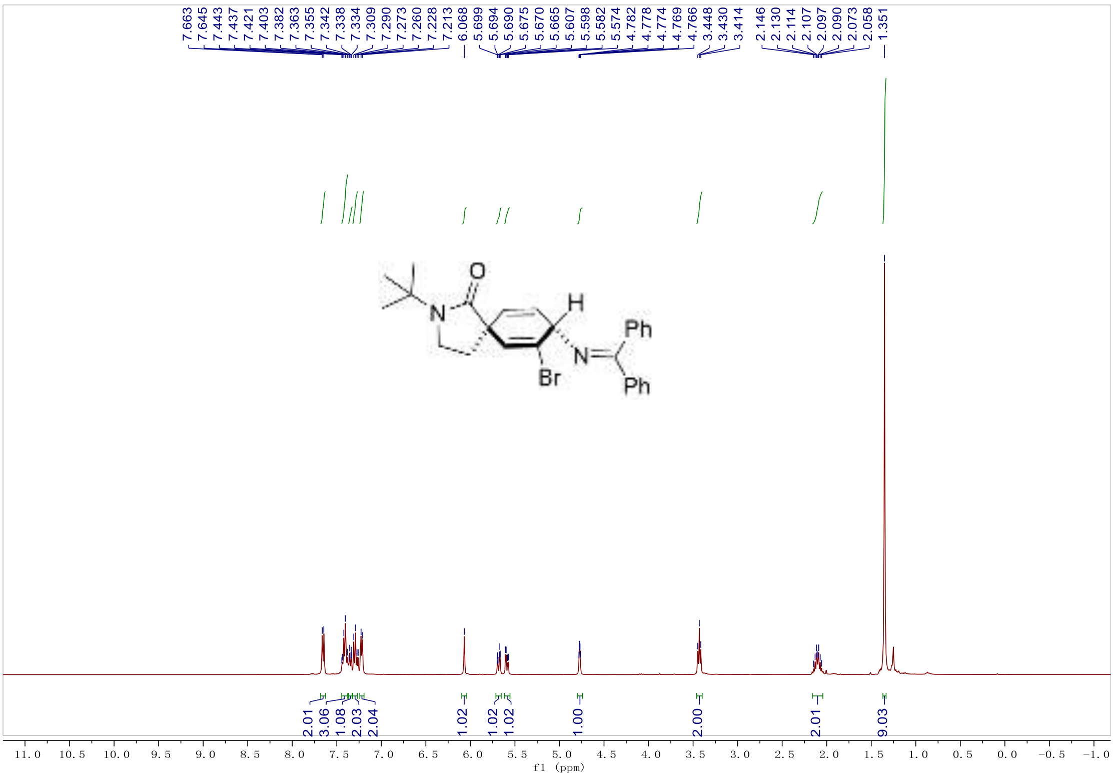

^{1}H-NMR for trans-**2-7**

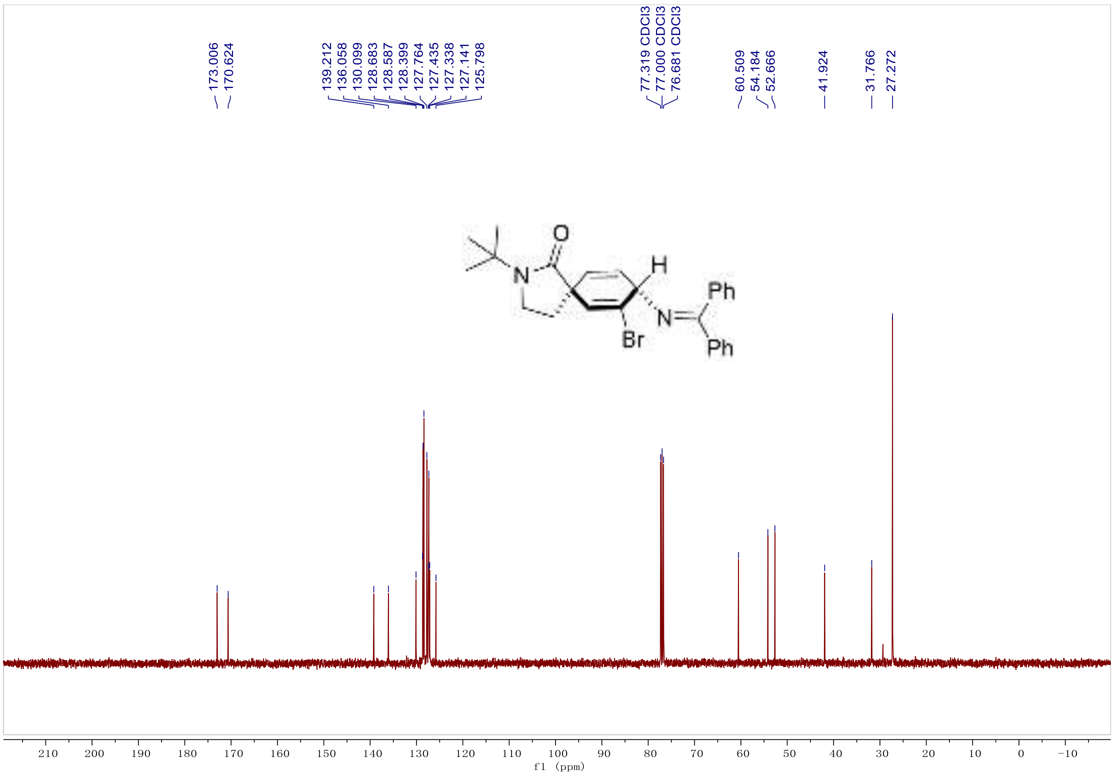

^{13}C-NMR for trans-**2-7**

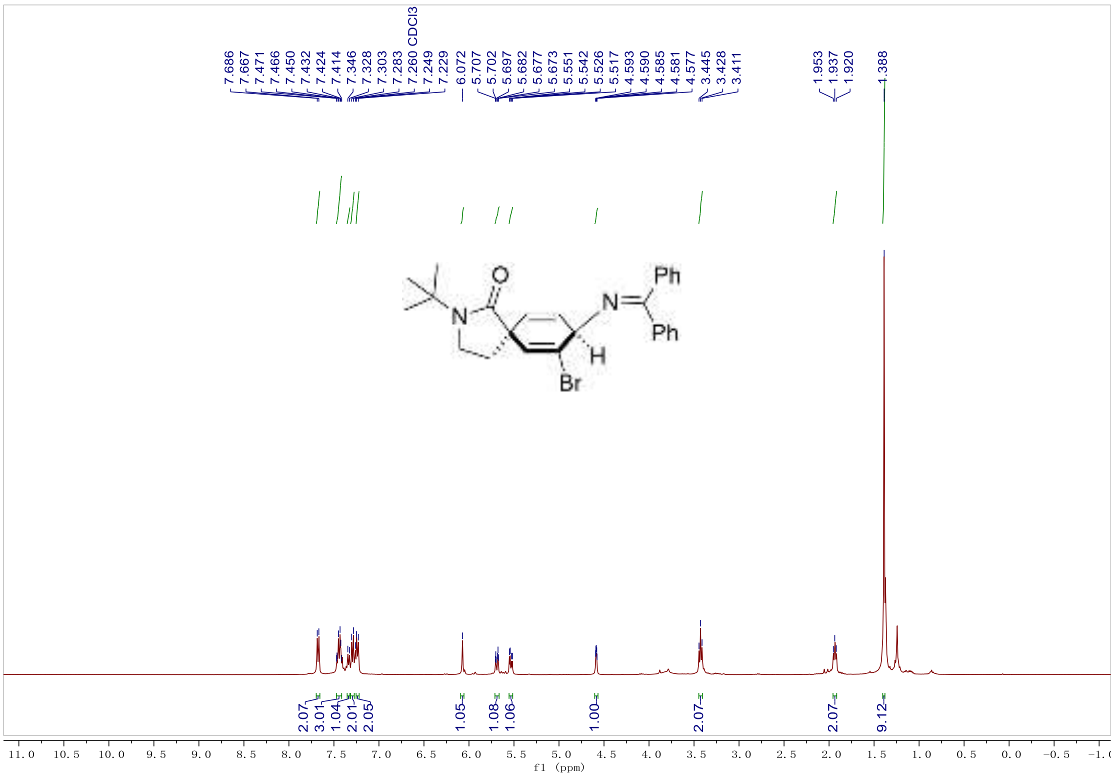

^{1}H-NMR for cis-**2-7**

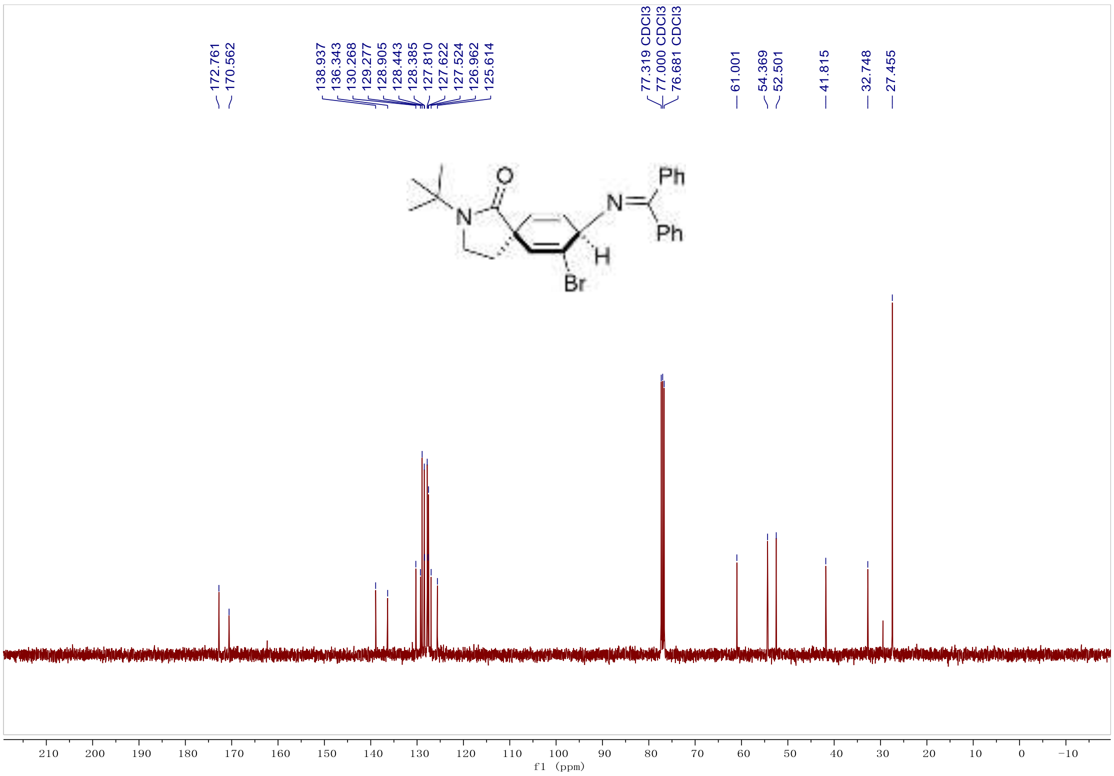

^{13}C-NMR for cis-**2-7**

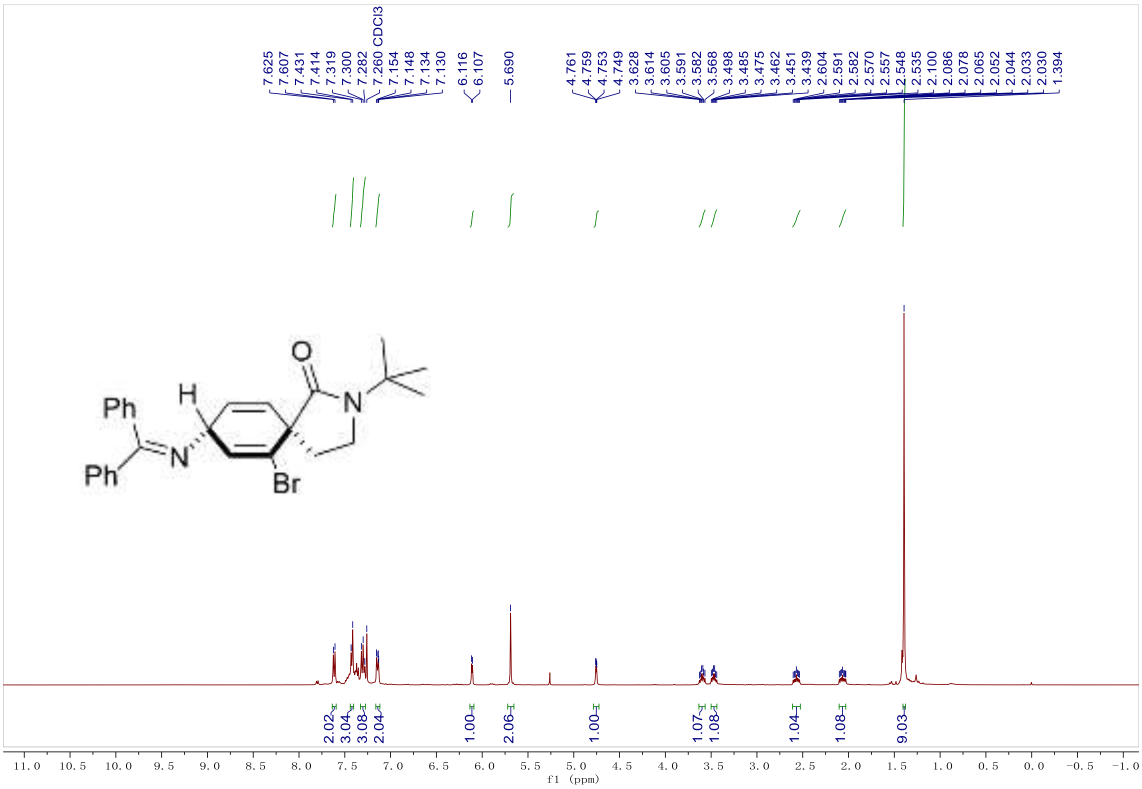

^{1}H-NMR for trans-**2-8**

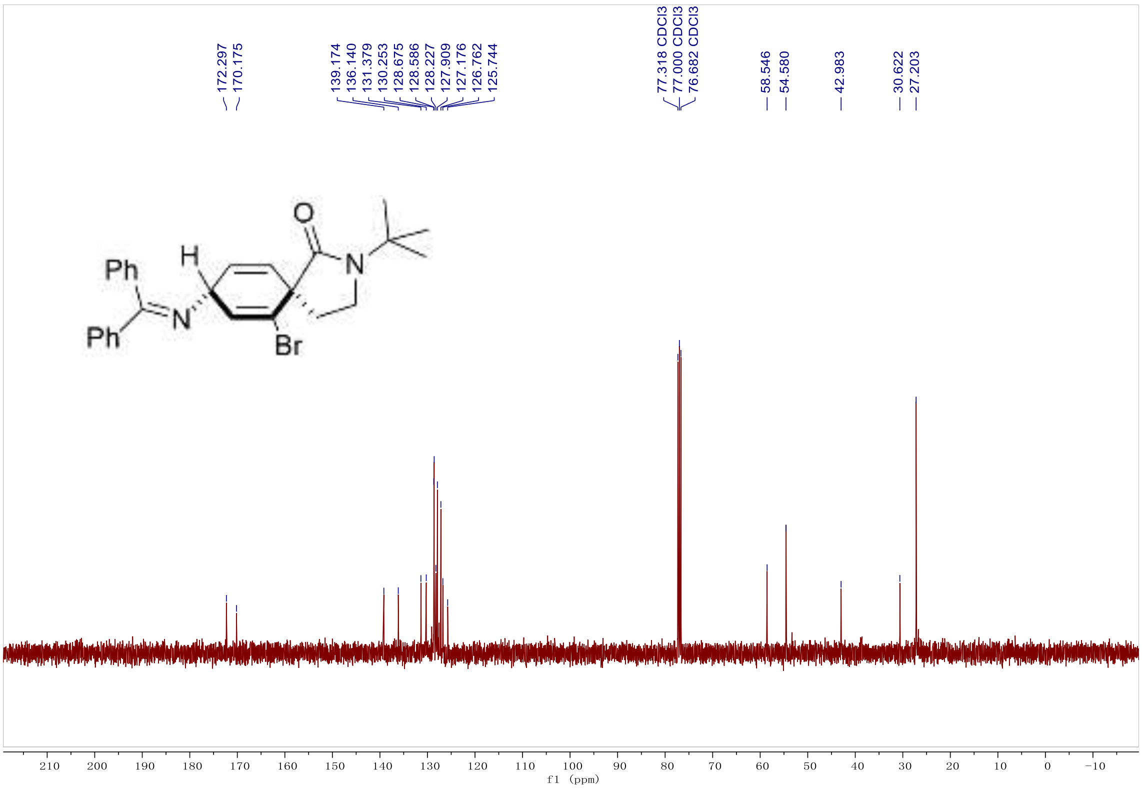

^{13}C−NMR for trans-**2-8**

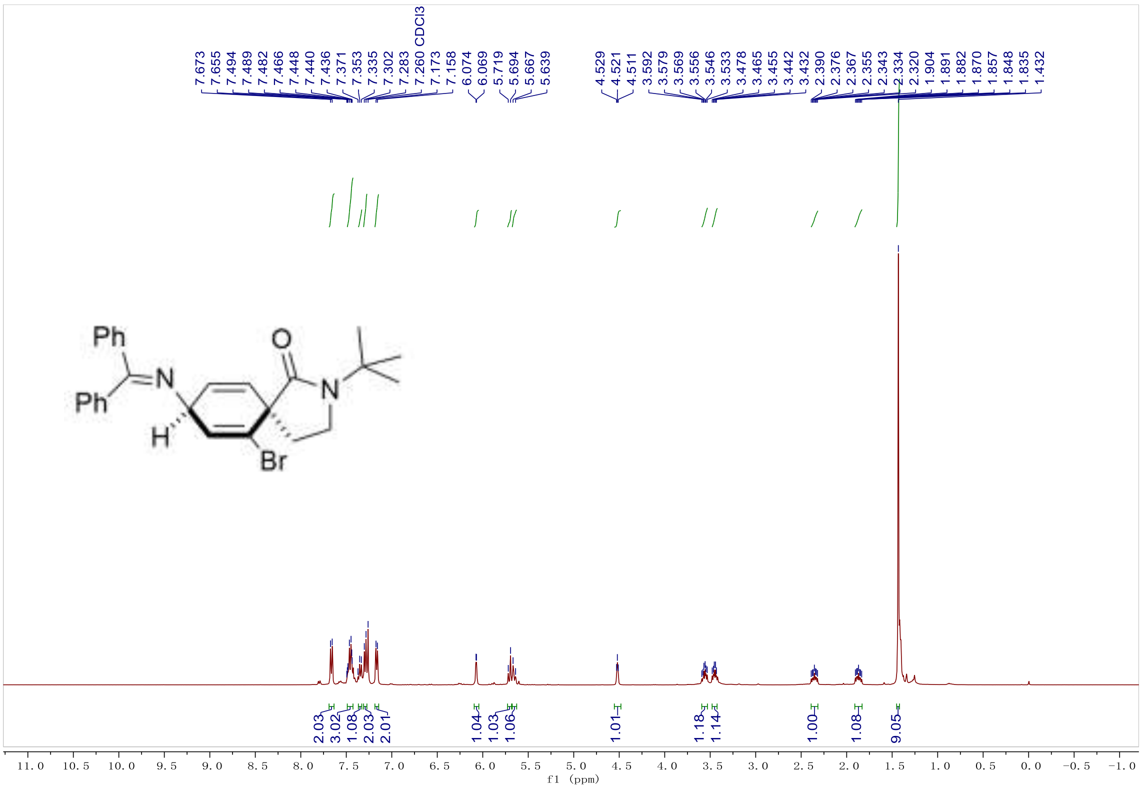

^{1}H-NMR for cis-**2-8**

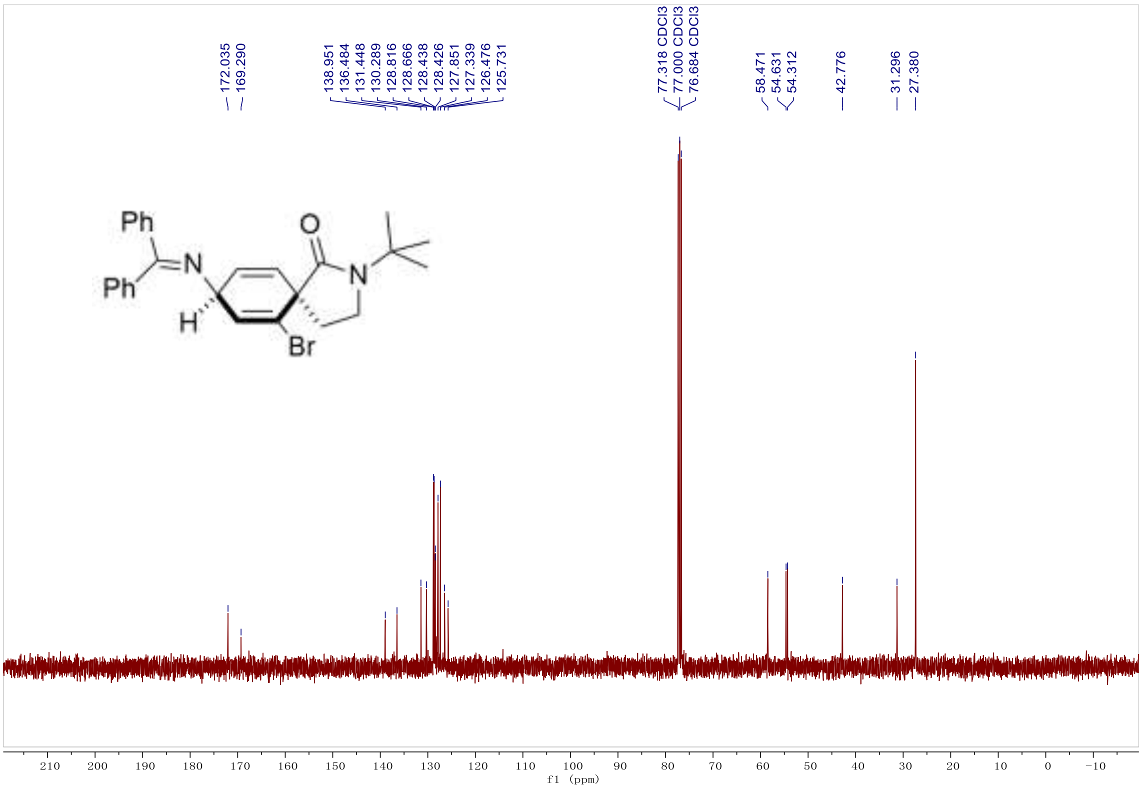

^{13}C−NMR for cis-**2-8**

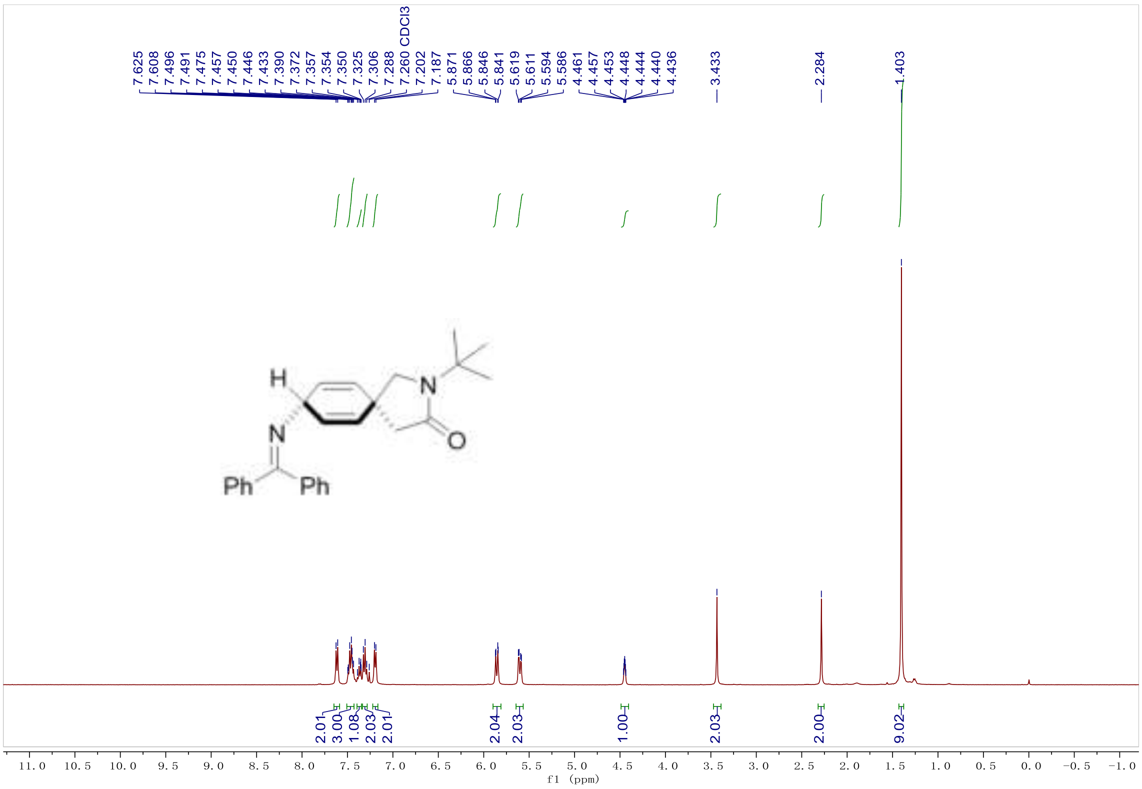

^{1}H-NMR for trans-**2-9**

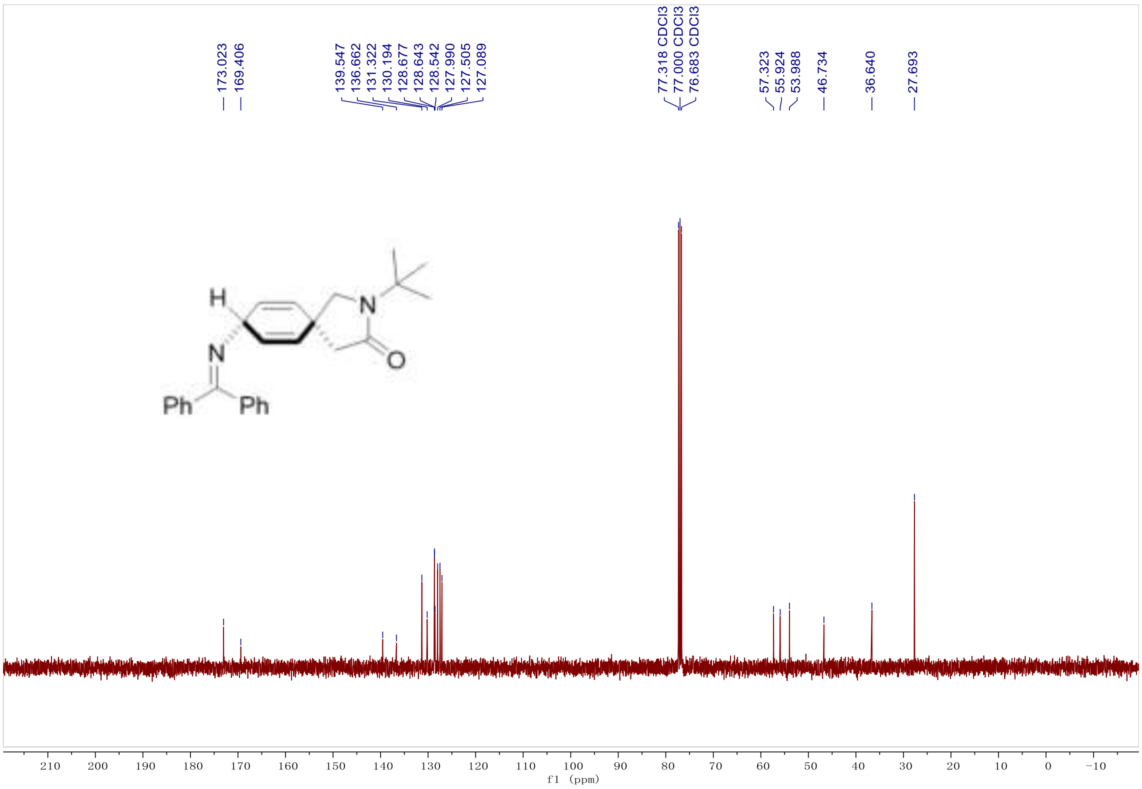

^{13}C−NMR for trans-**2-9**

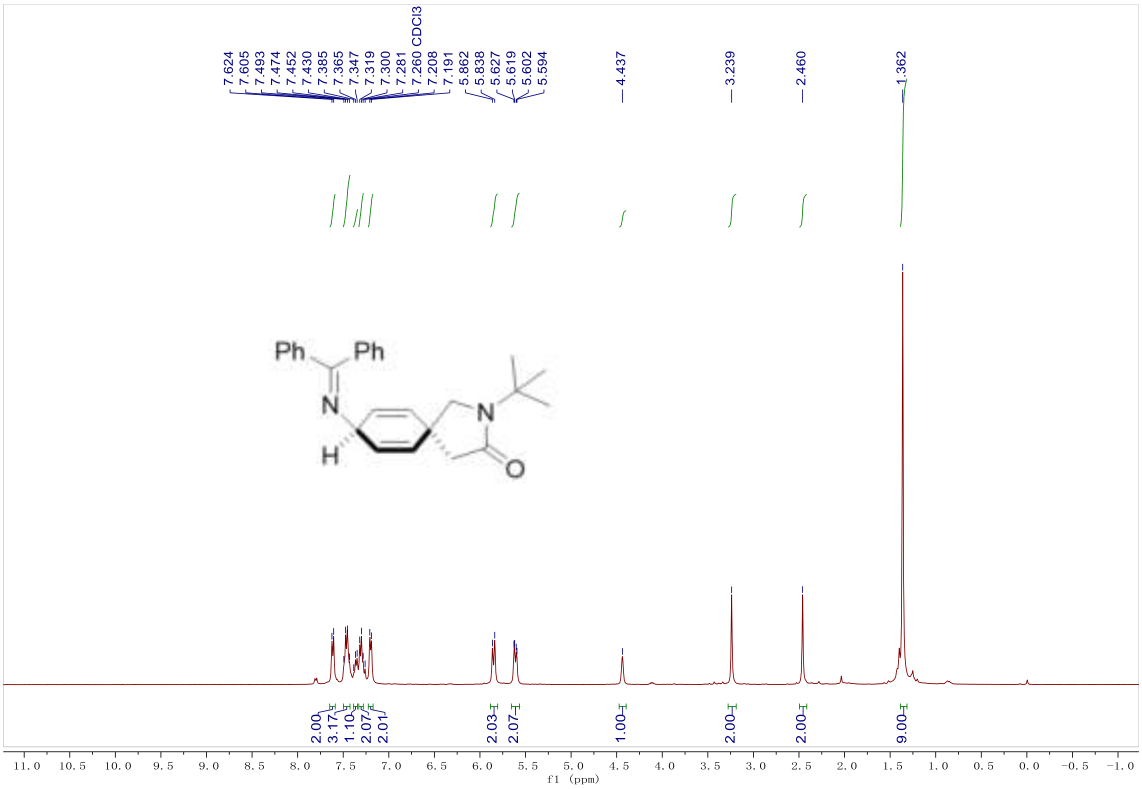

^{1}H-NMR for cis-**2-9**

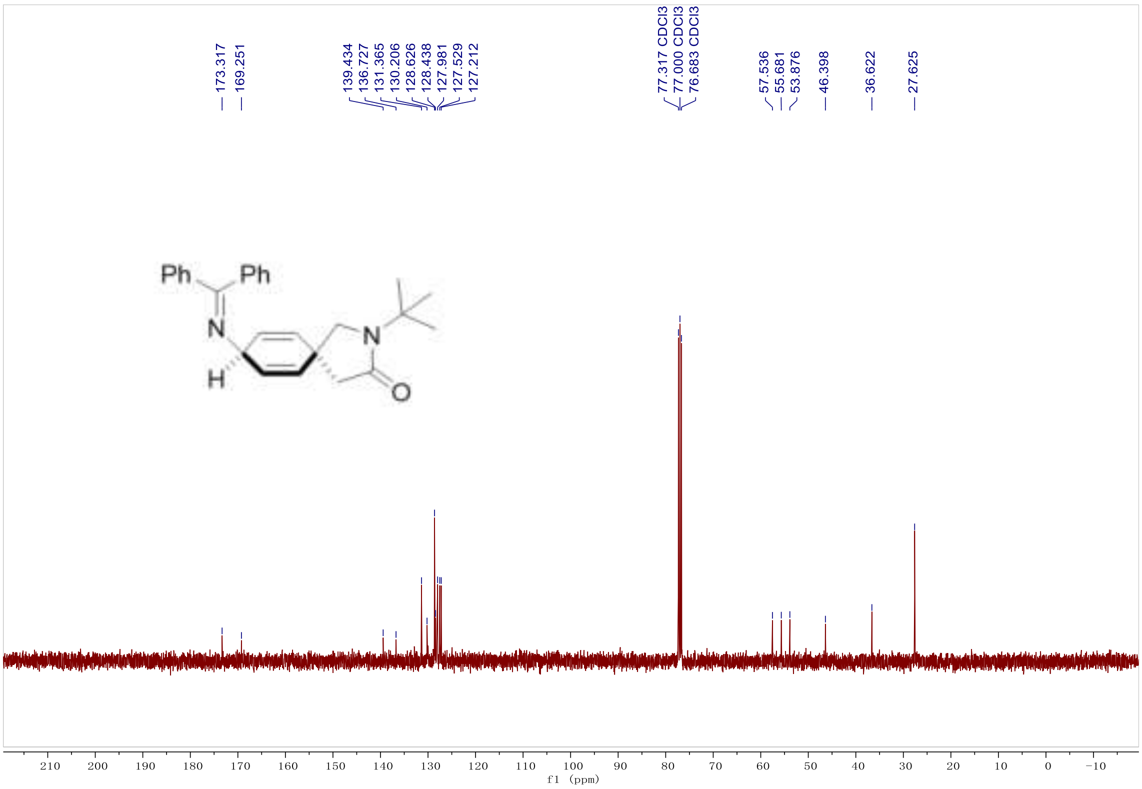

^{13}C−NMR for cis-**2-9**

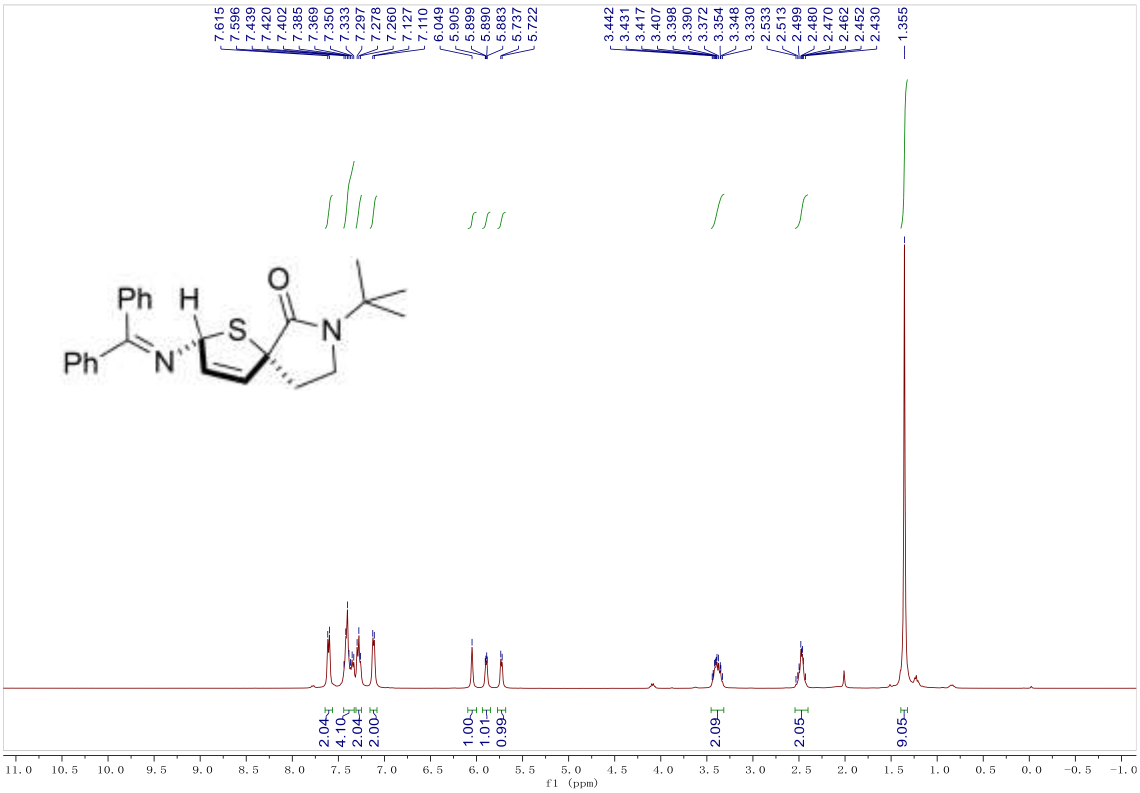

^{1}H-NMR for trans-**2-10**

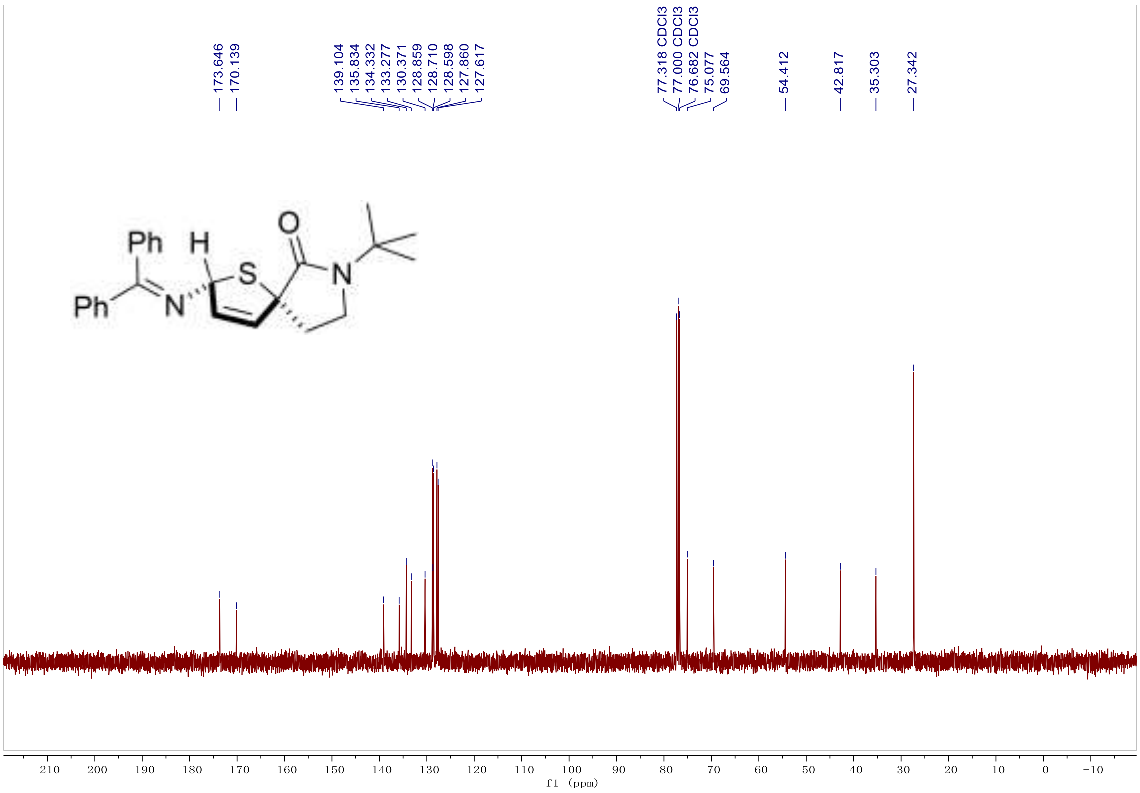

^{13}C−NMR for trans-**2-10**

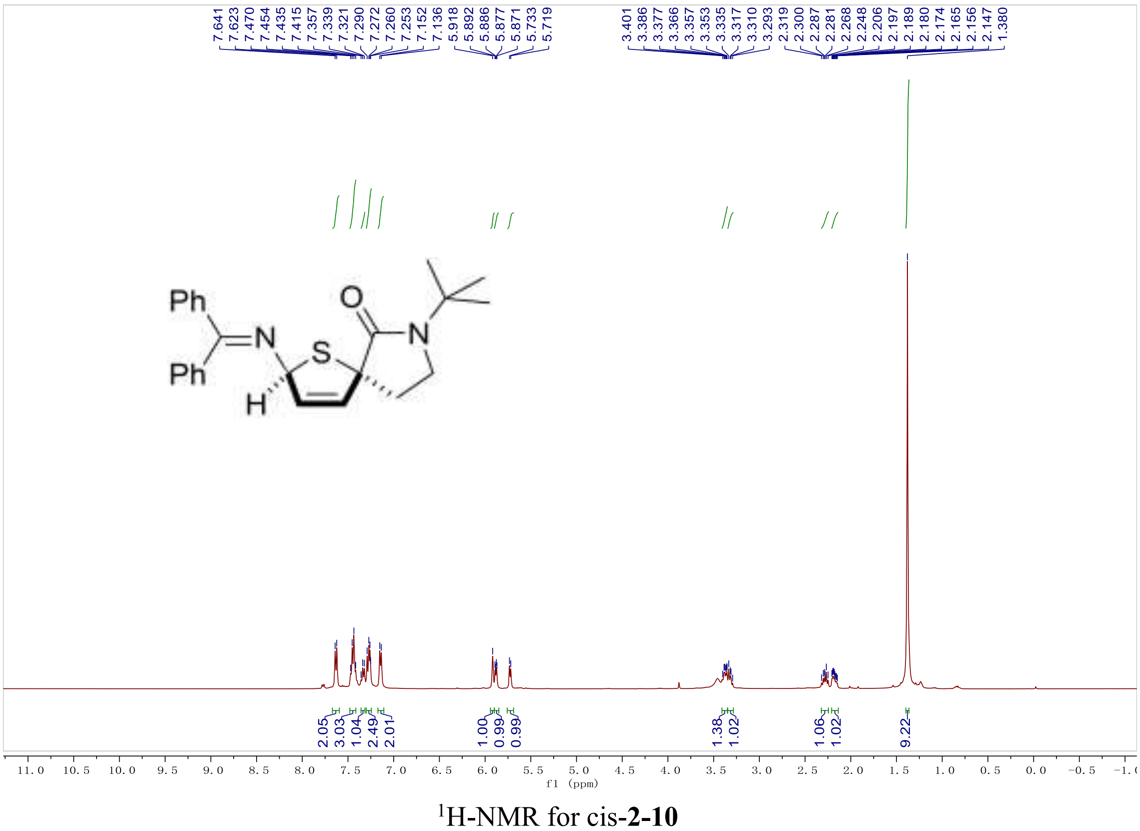

^{1}H-NMR for cis-**2-10**

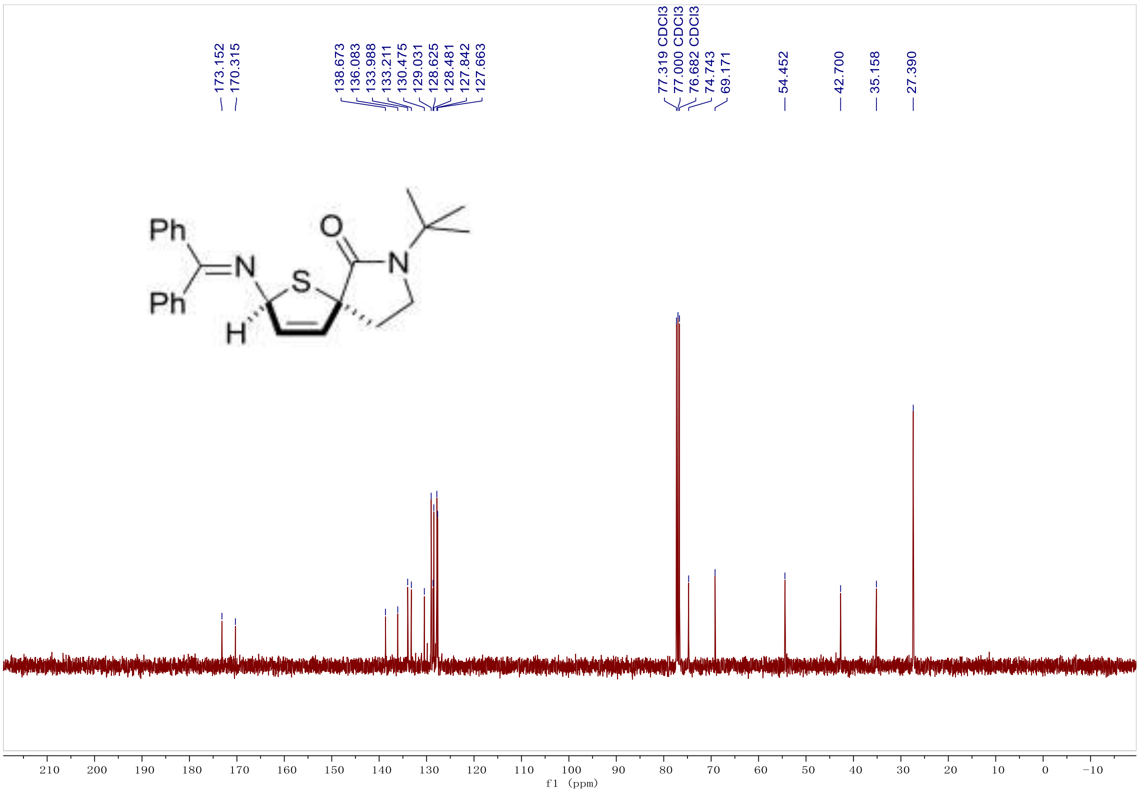

^{13}C−NMR for cis-**2-10**

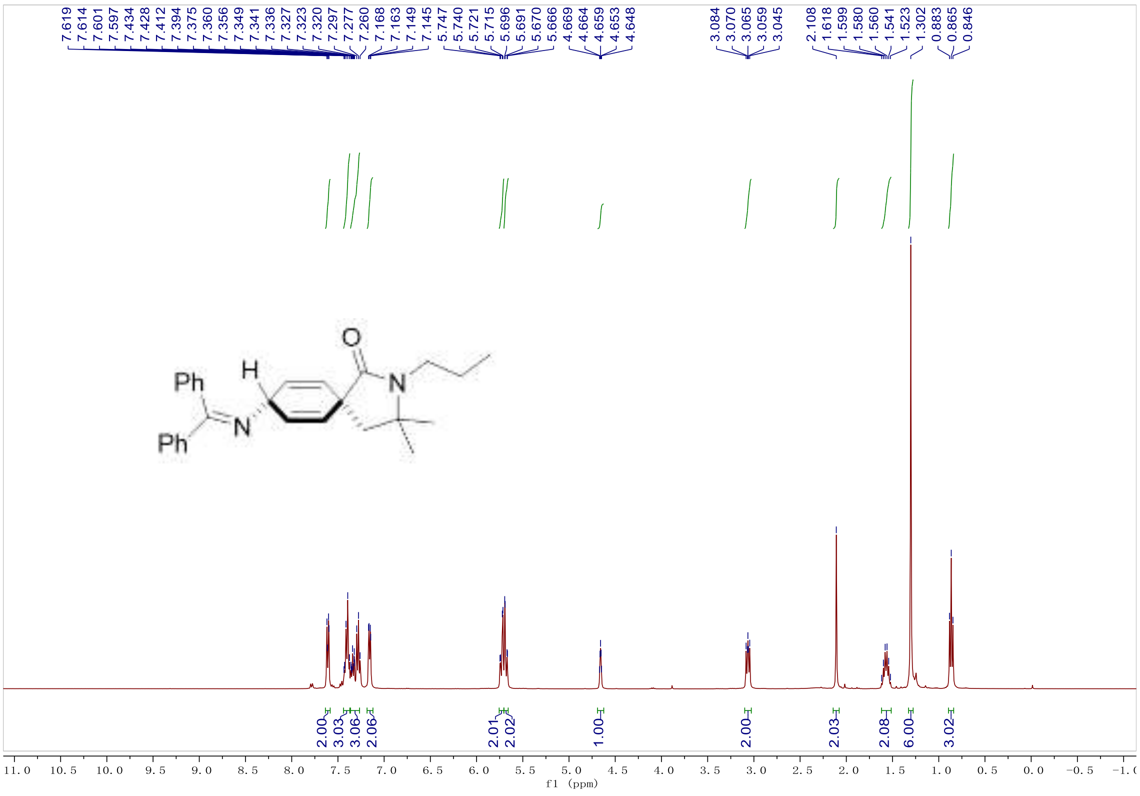

^{1}H-NMR for trans-**2-11**

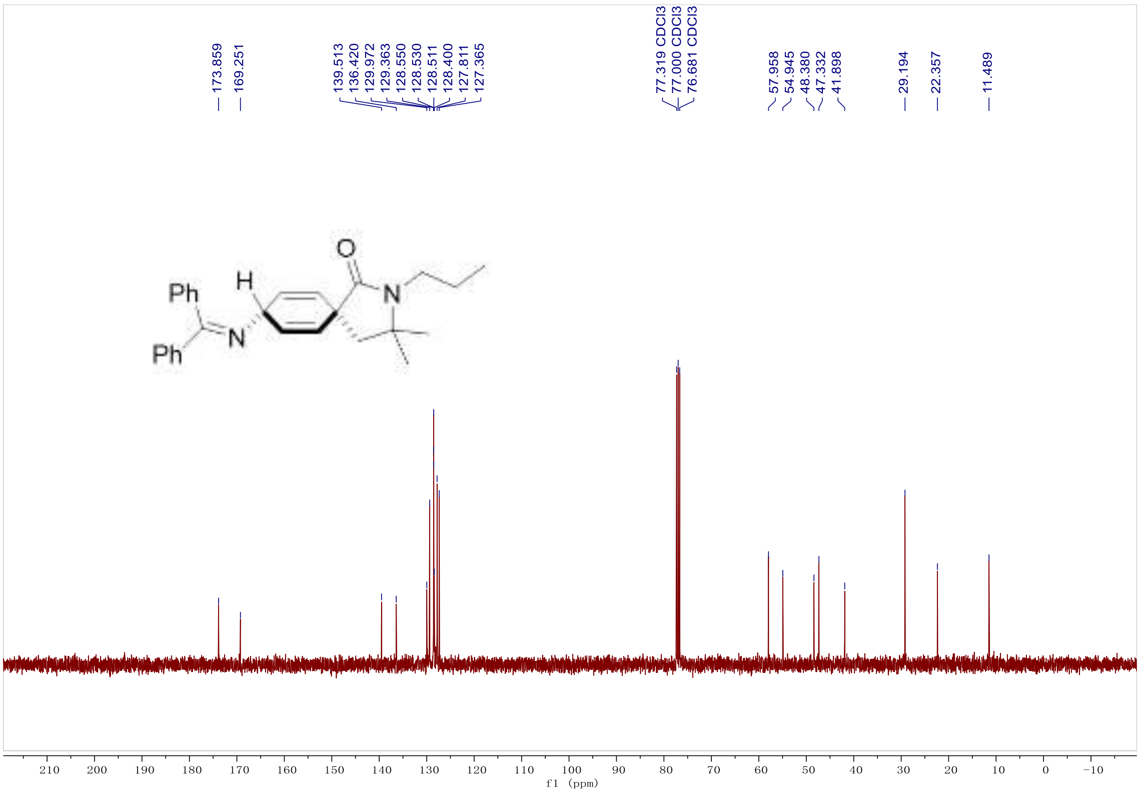

^{13}C−NMR for trans-**2-11**

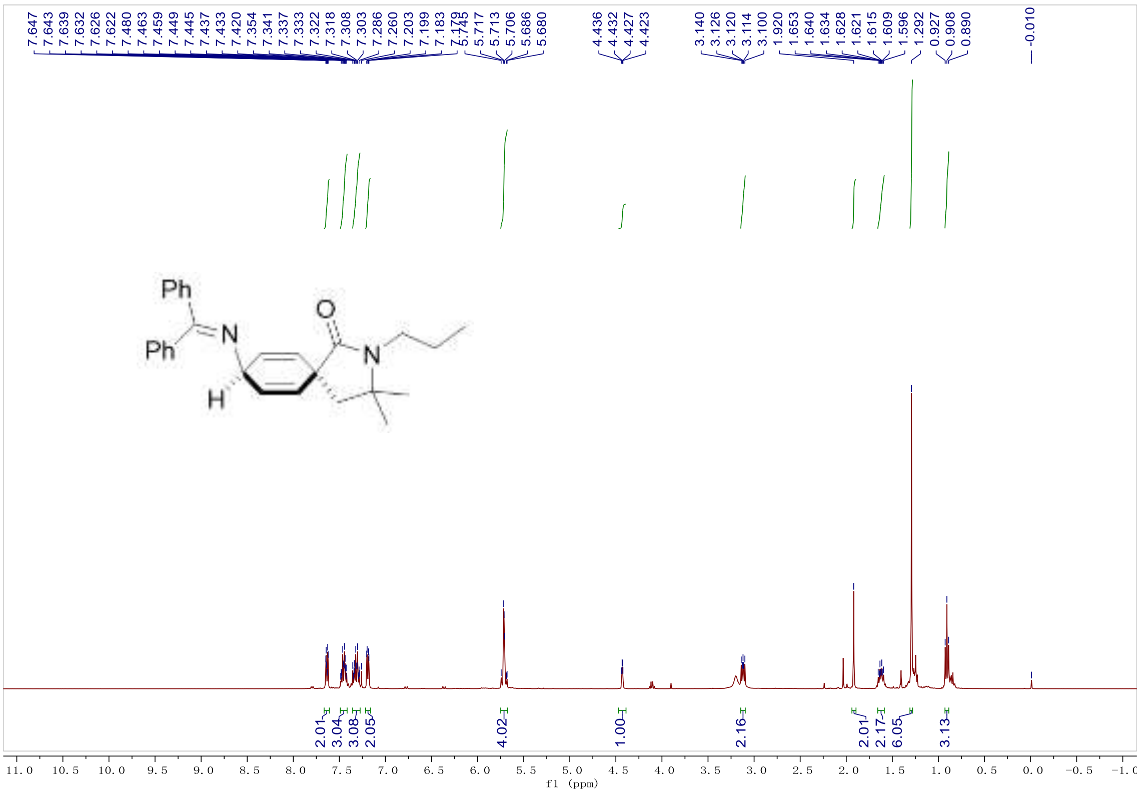

^{1}H-NMR for cis-**2-11**

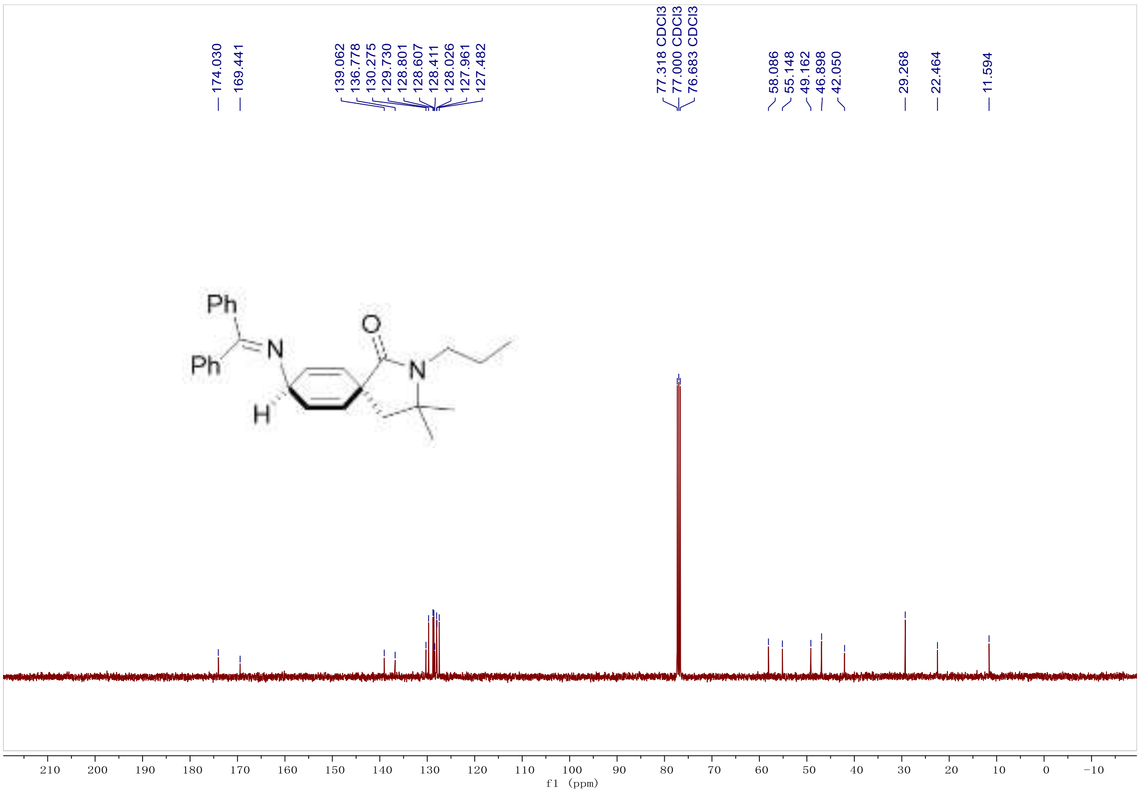

^{13}C−NMR for cis-**2-11**

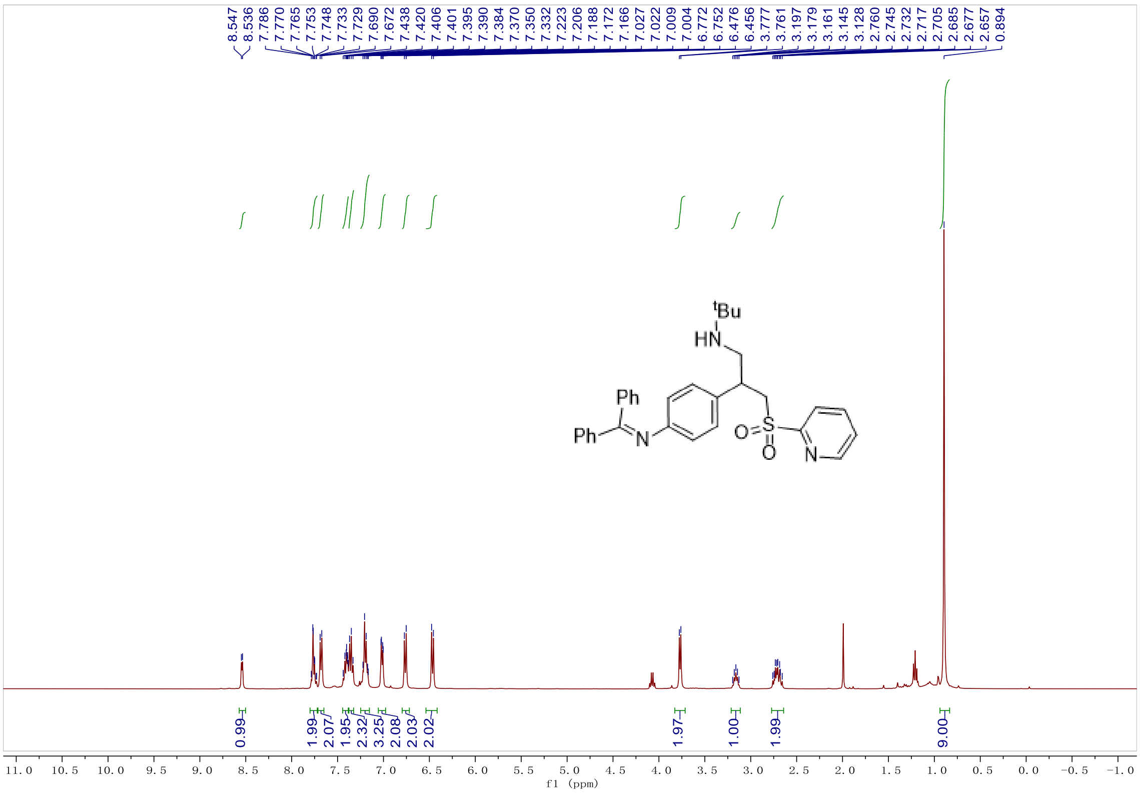

^{1}H-NMR for **6-1**

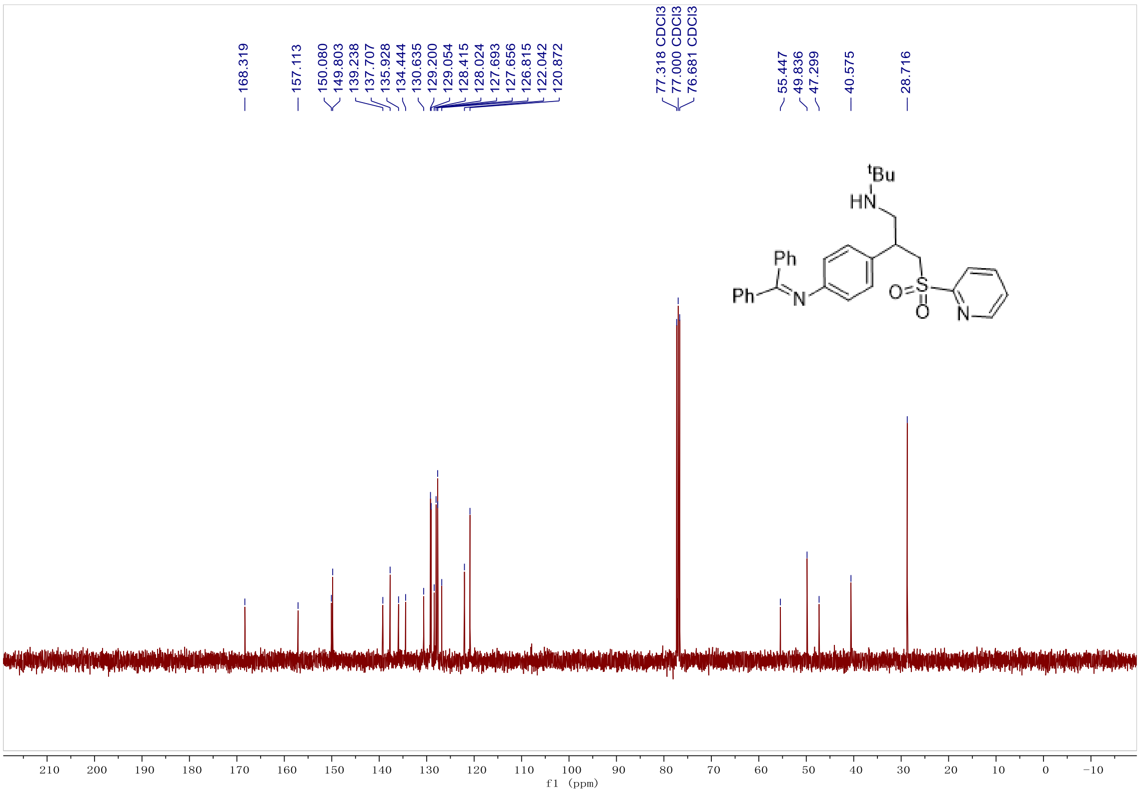

^{13}C−NMR for **6-1**

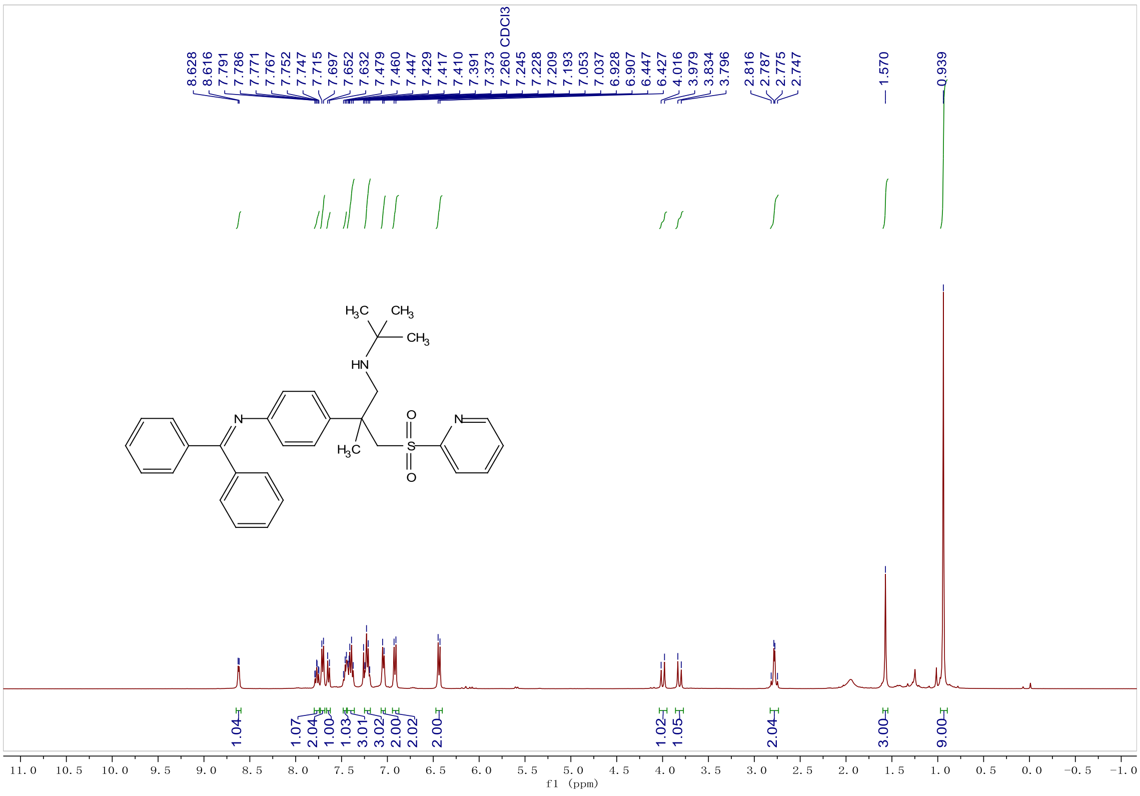

^{1}H-NMR for **6-2**

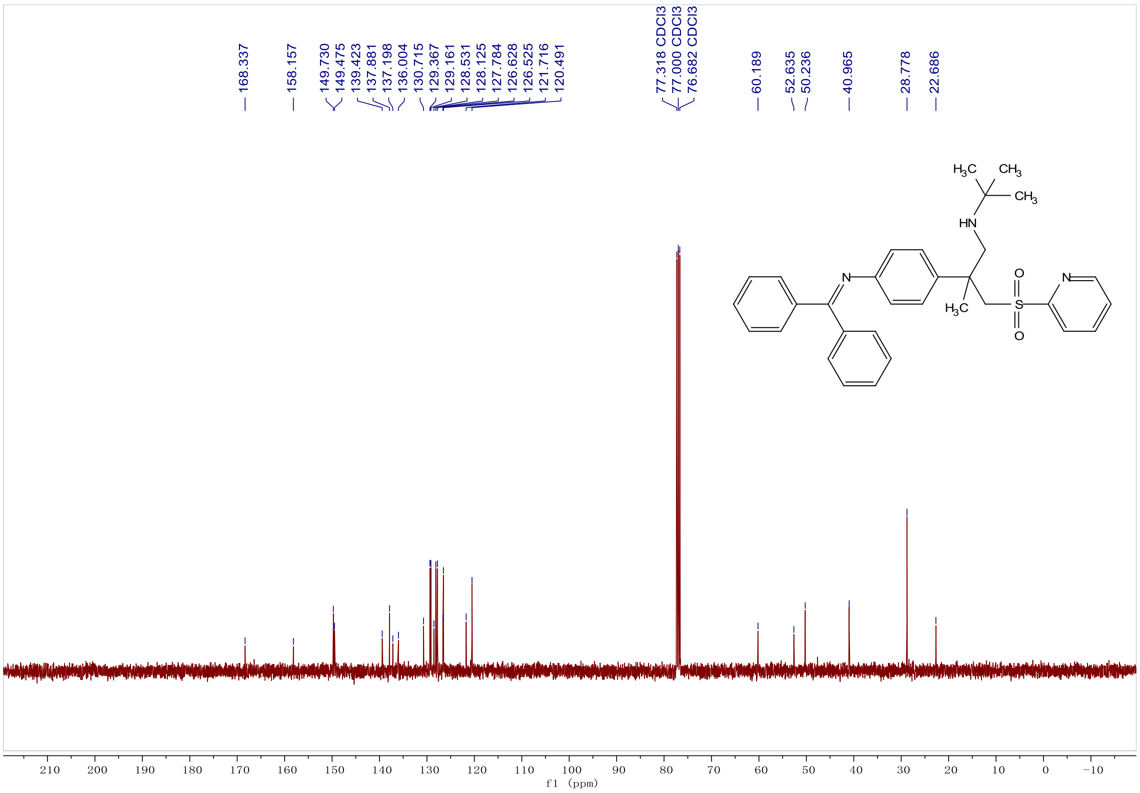

^{13}C−NMR for **6-2**

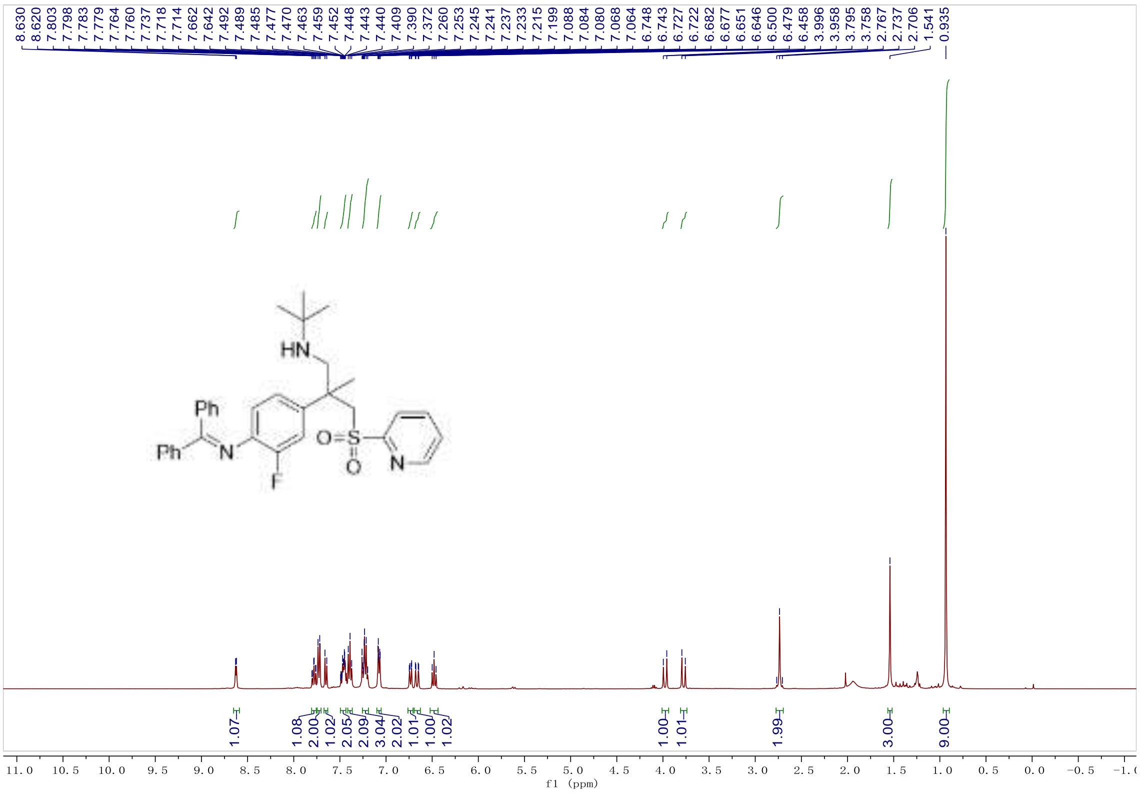

^{1}H-NMR for **6-3**

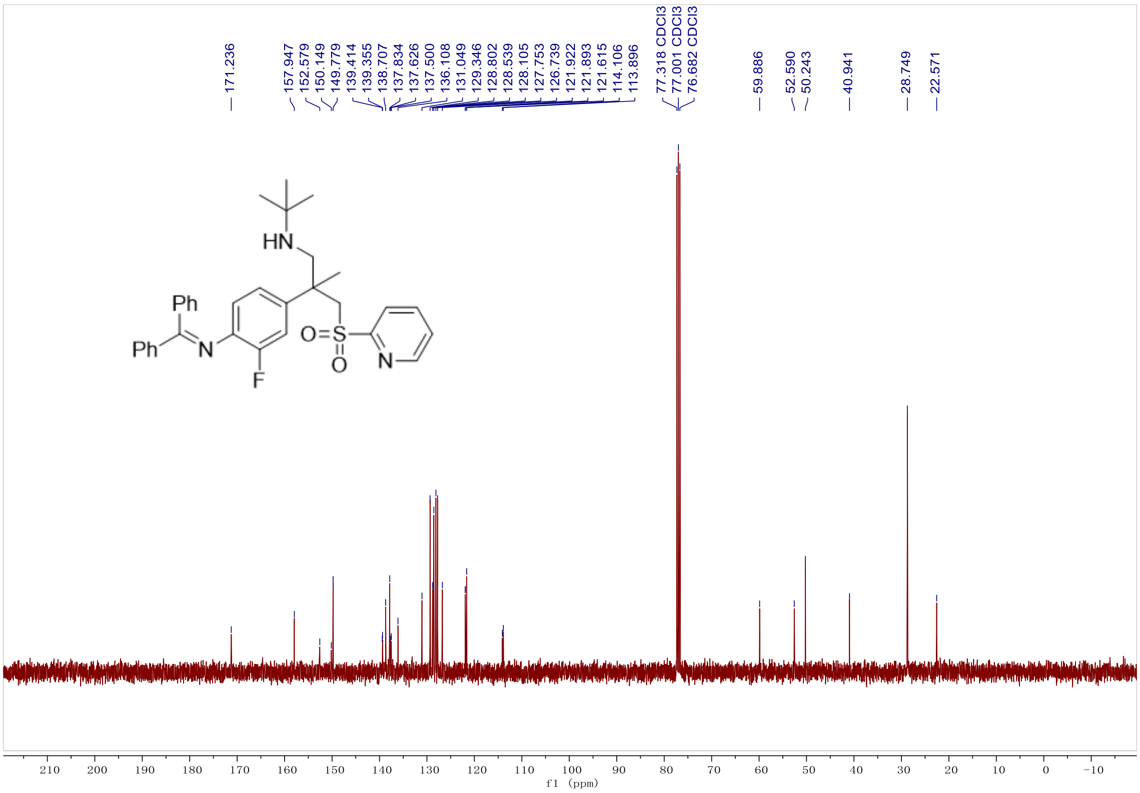

^{13}C−NMR for **6-3**

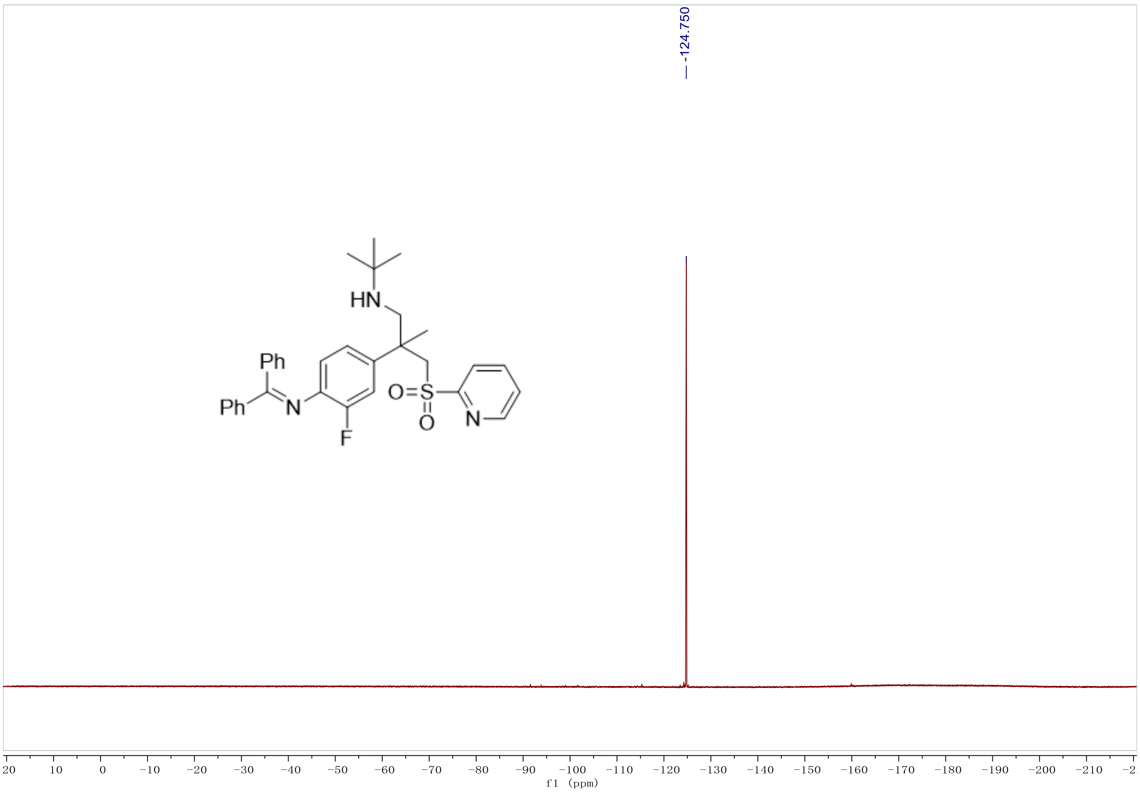

^{19}F-NMR for **6-3**

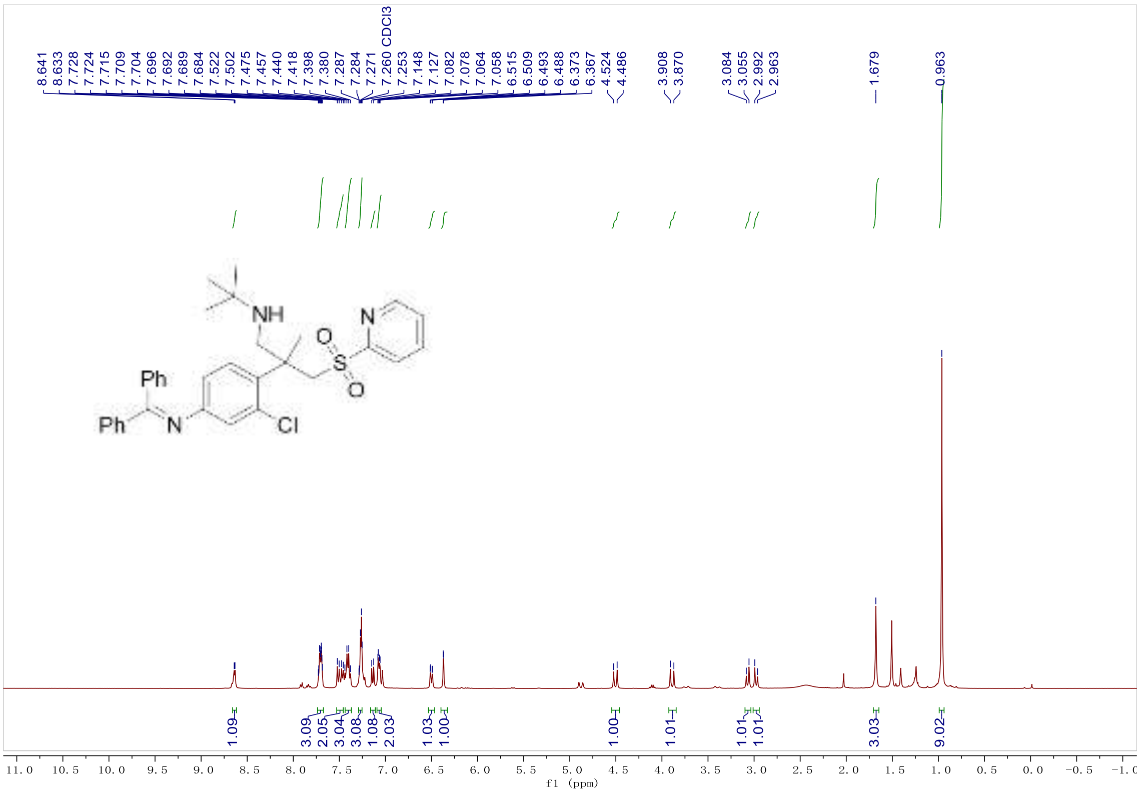

^{1}H-NMR for **6-4**

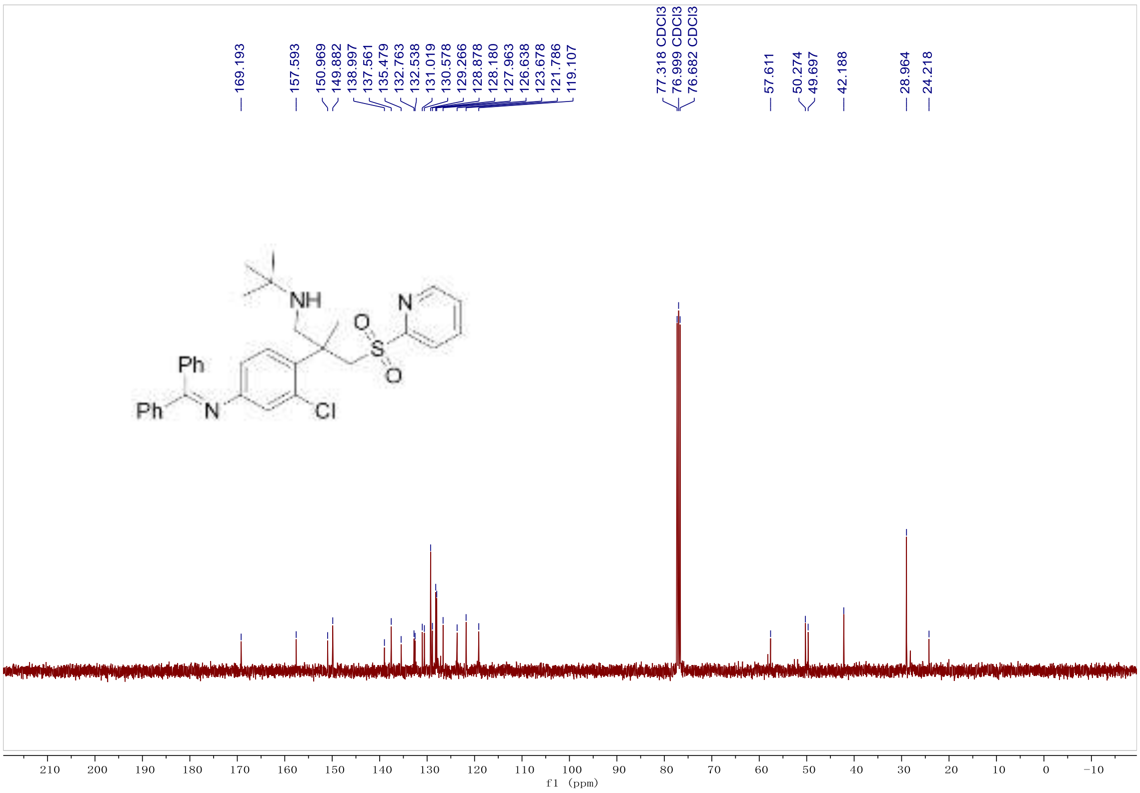

^{13}C−NMR for **6-4**

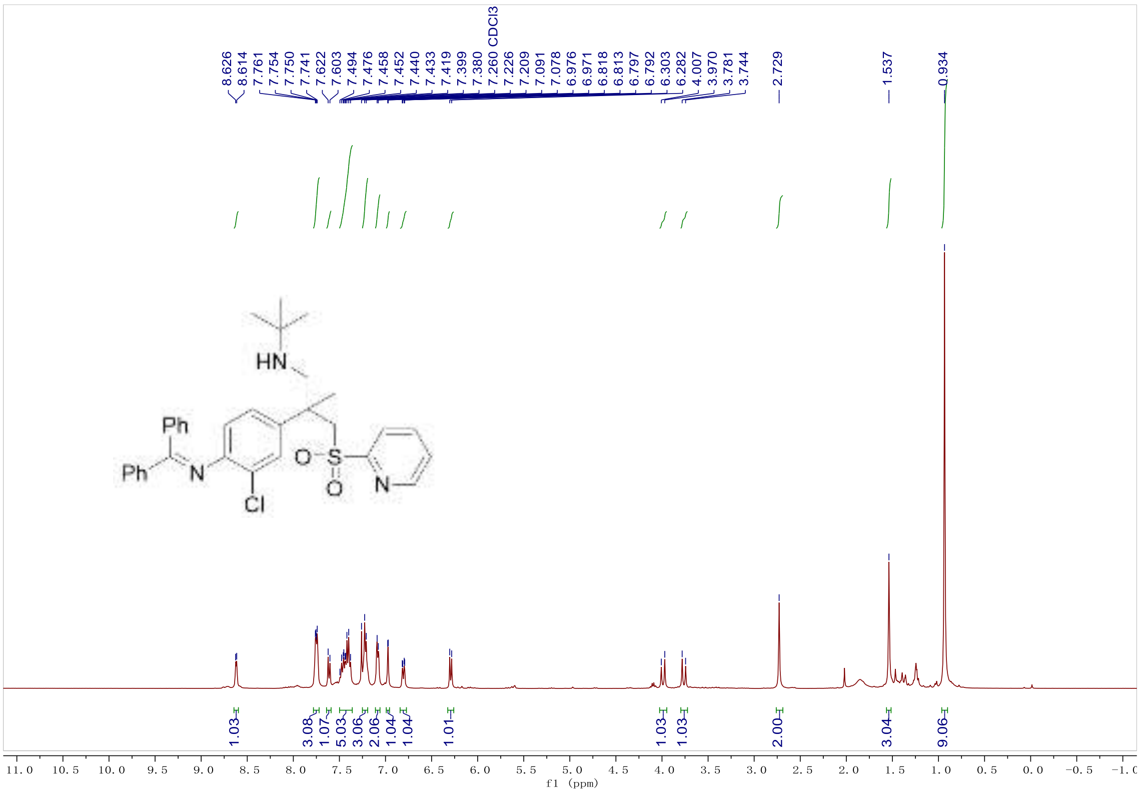

^{1}H-NMR for **6-5**

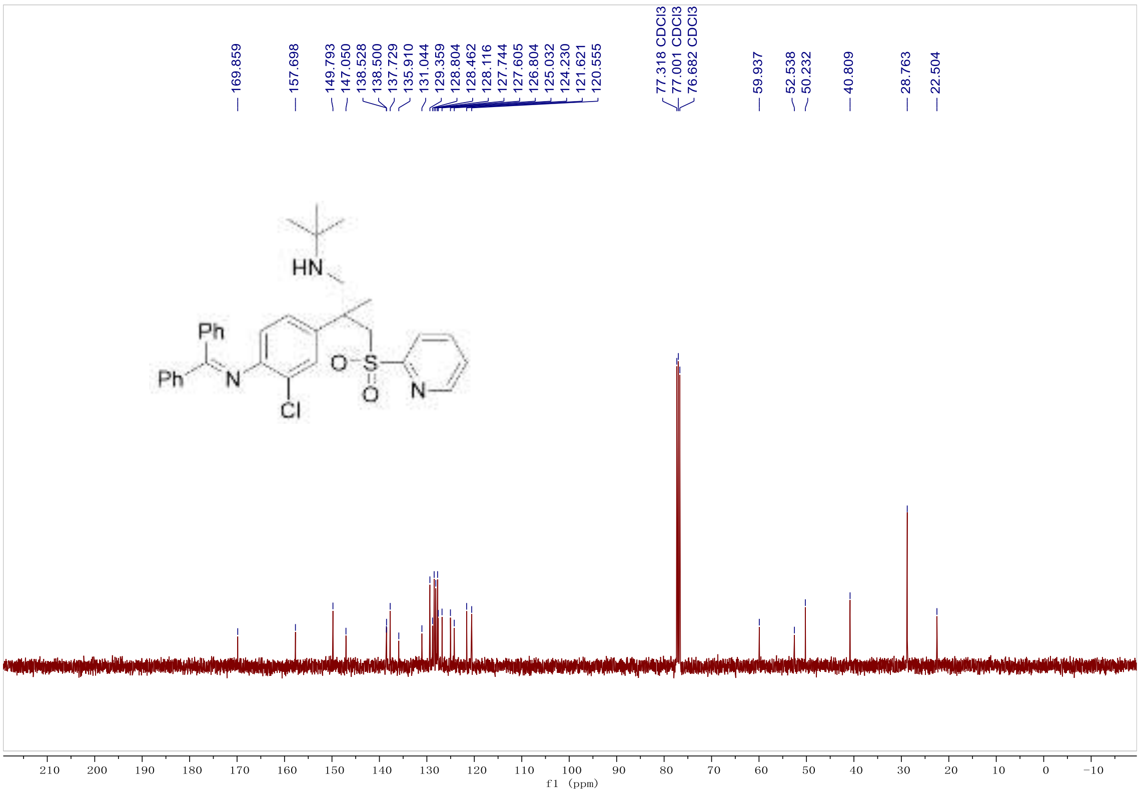

^{13}C−NMR for **6-5**

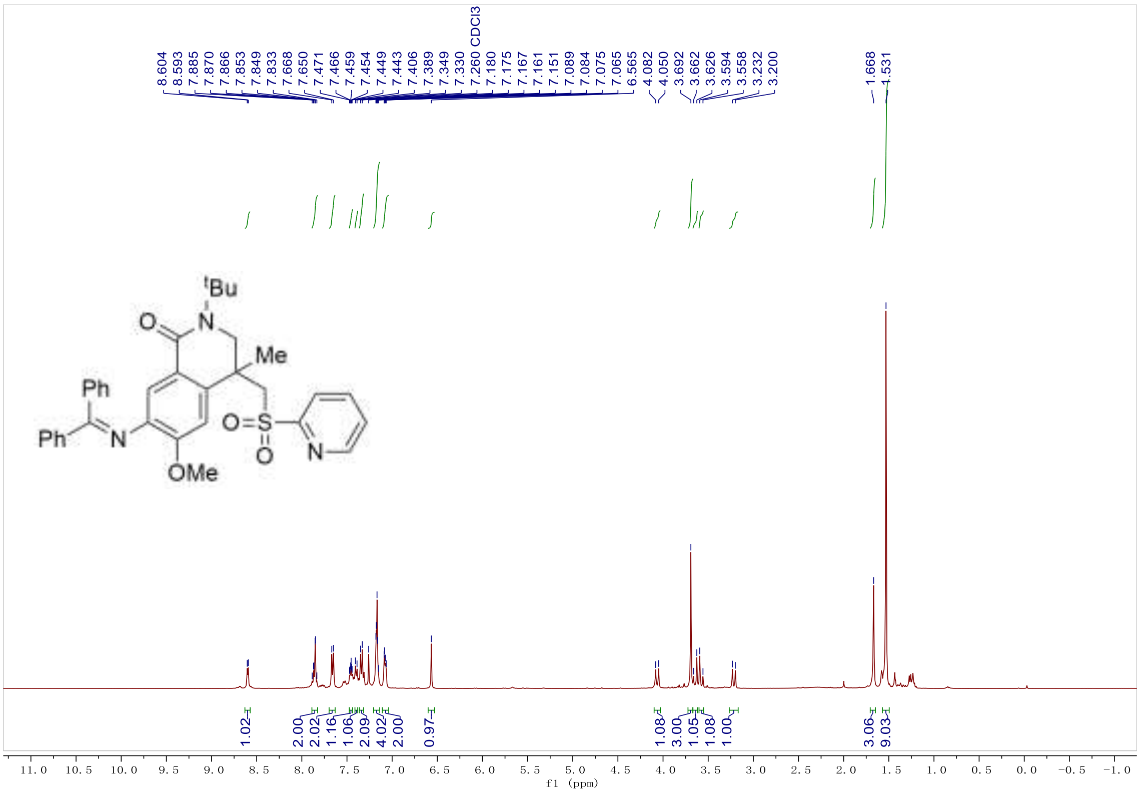

^{1}H-NMR for **6-6**

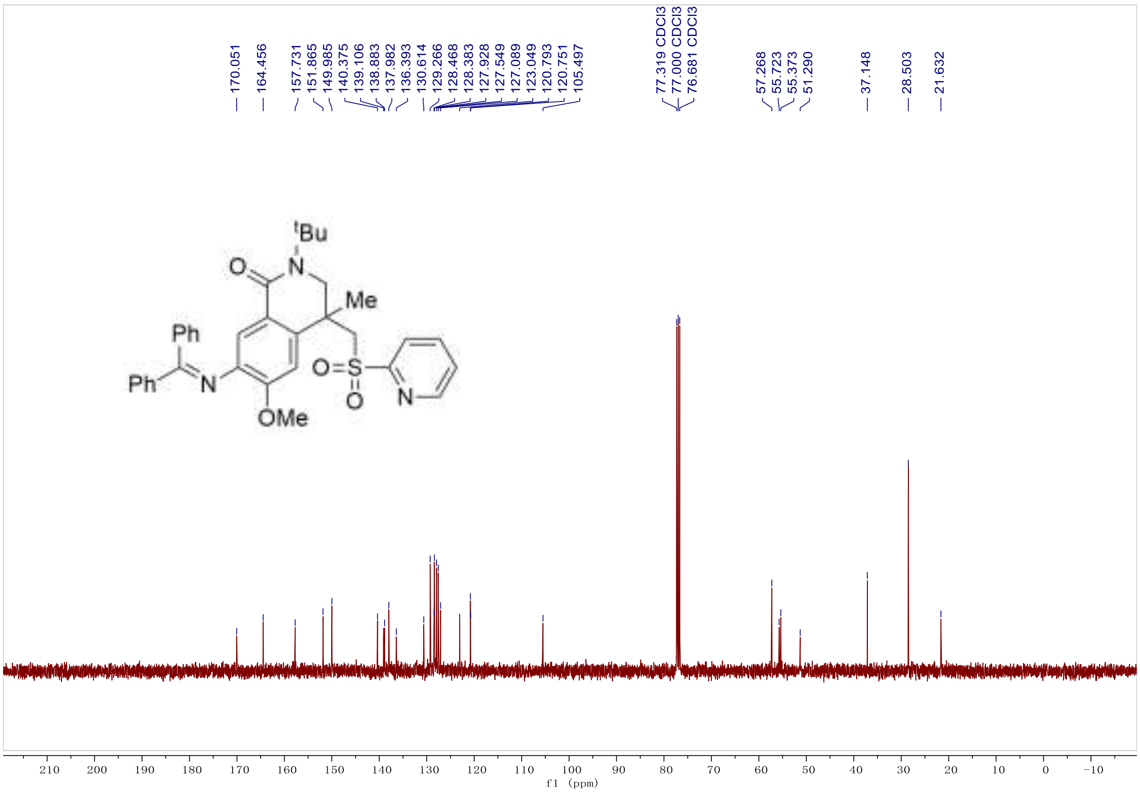

^{13}C−NMR for **6-6**

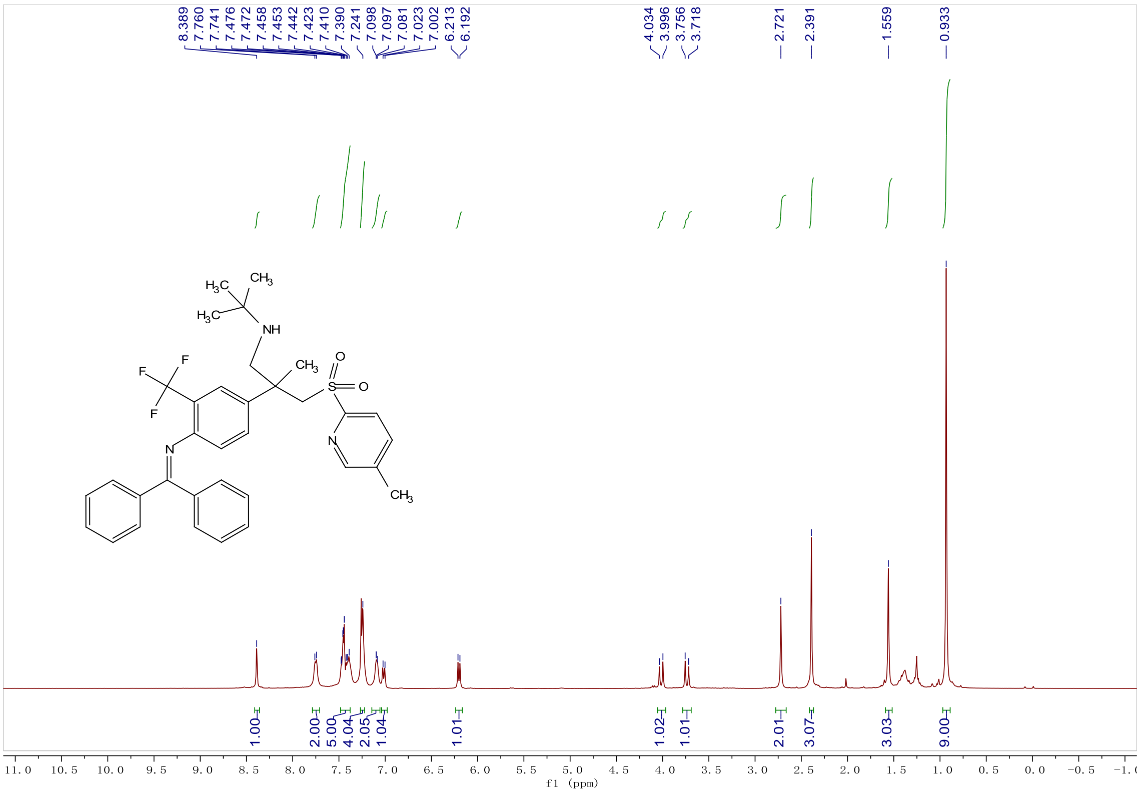

^{1}H-NMR for **6-7**

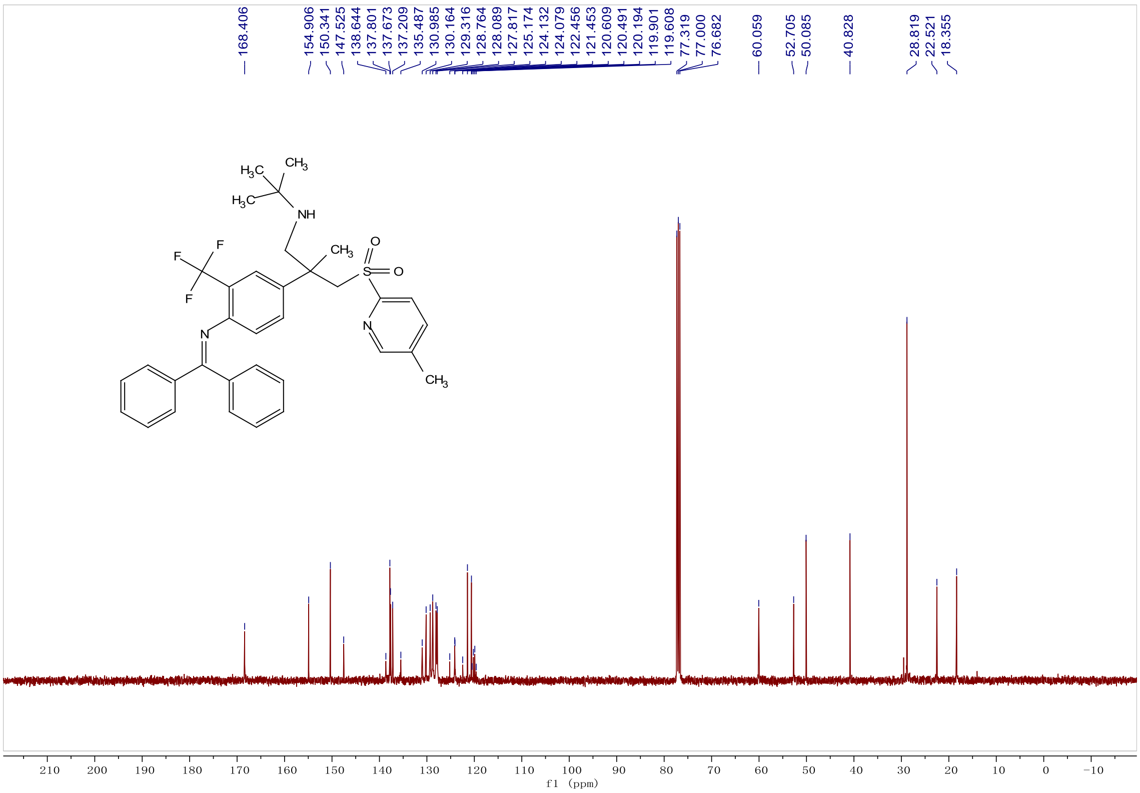

^{13}C−NMR for **6-7**

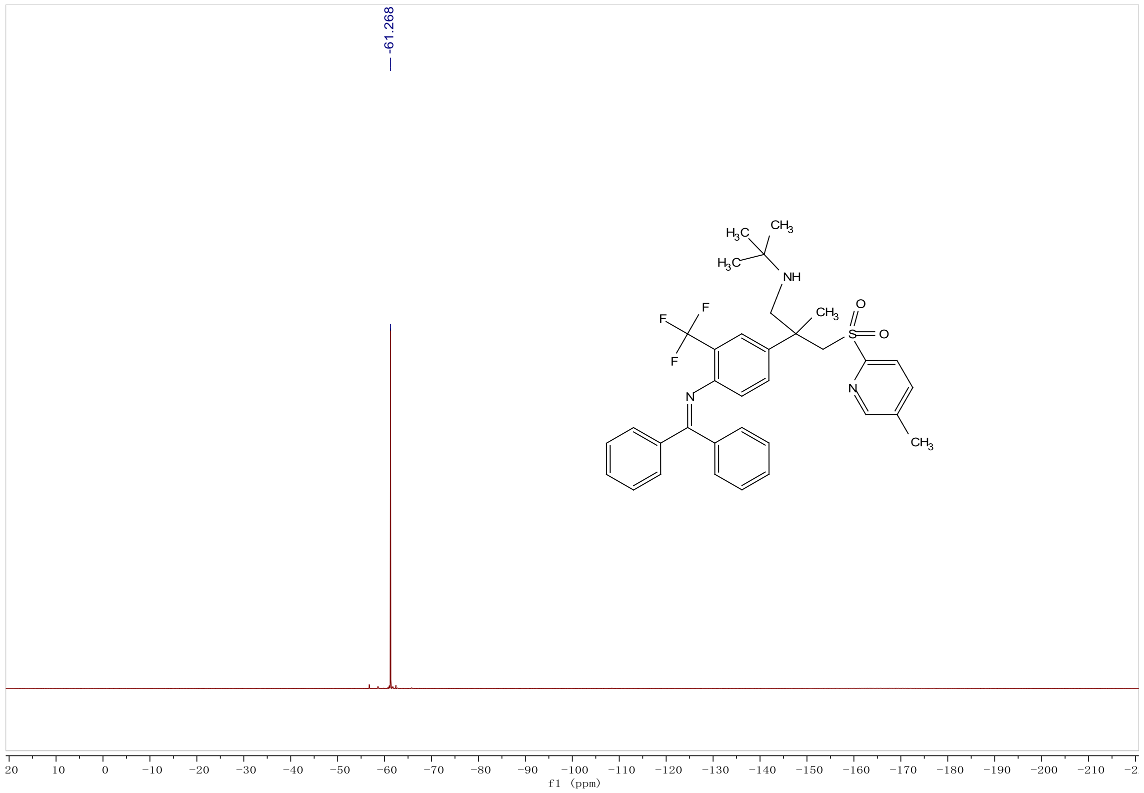

^{19}F-NMR for **6-7**

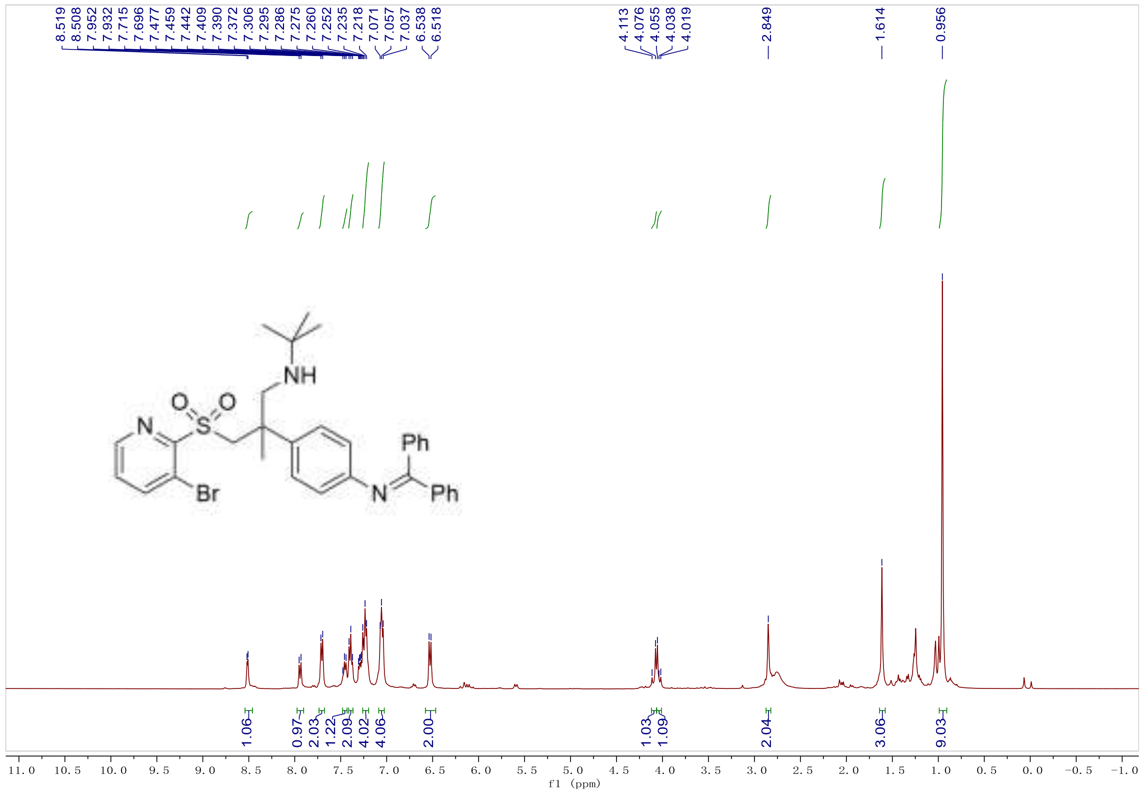

^{1}H-NMR for **6-8**

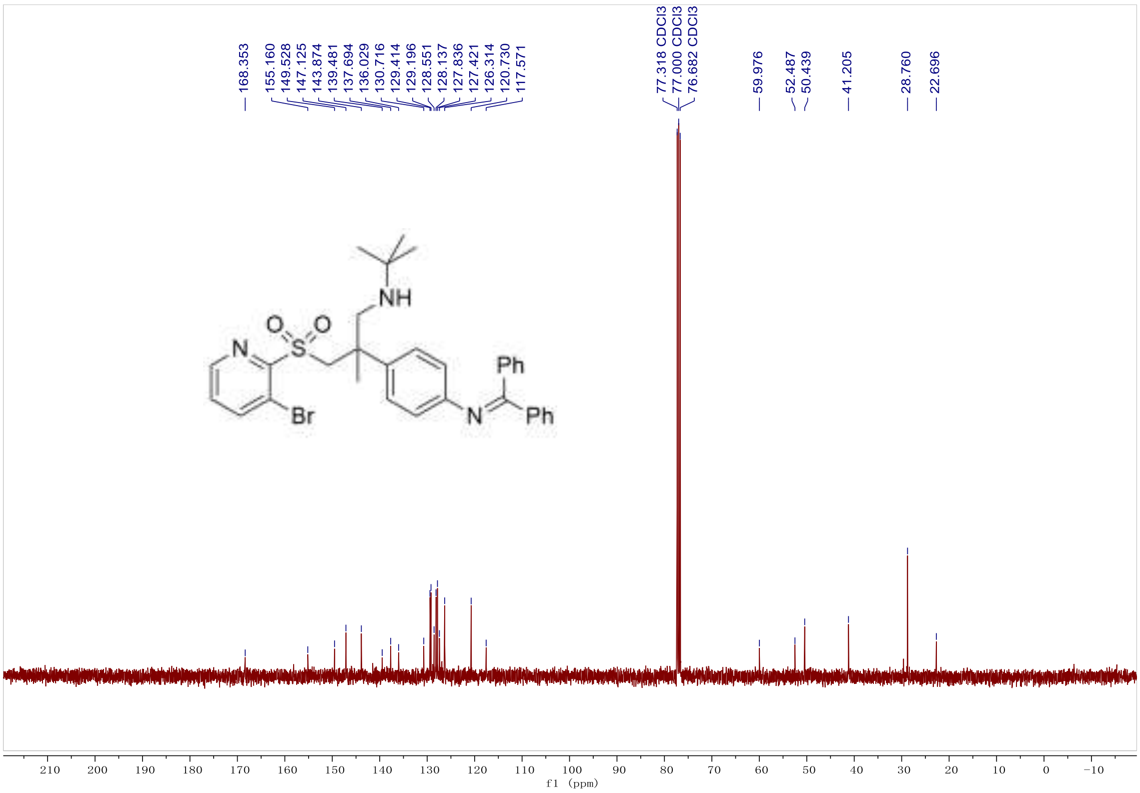

^{13}C−NMR for **6-8**

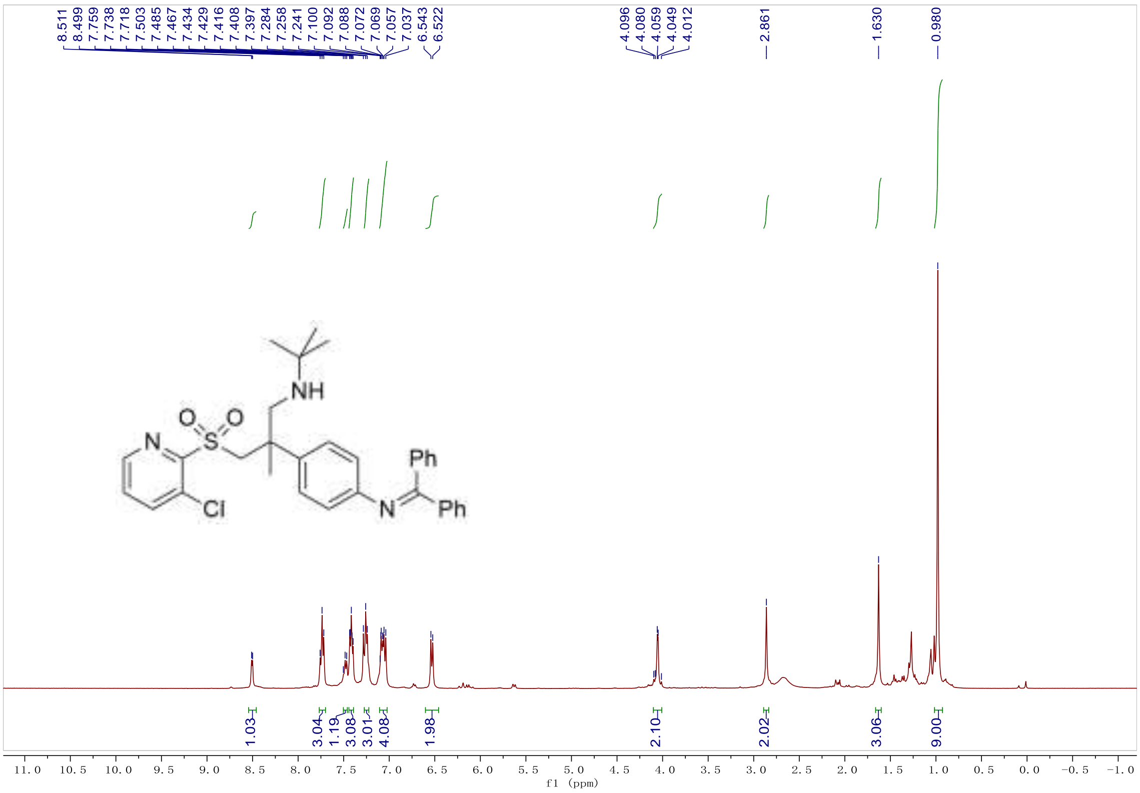

^{1}H-NMR for **6-9**

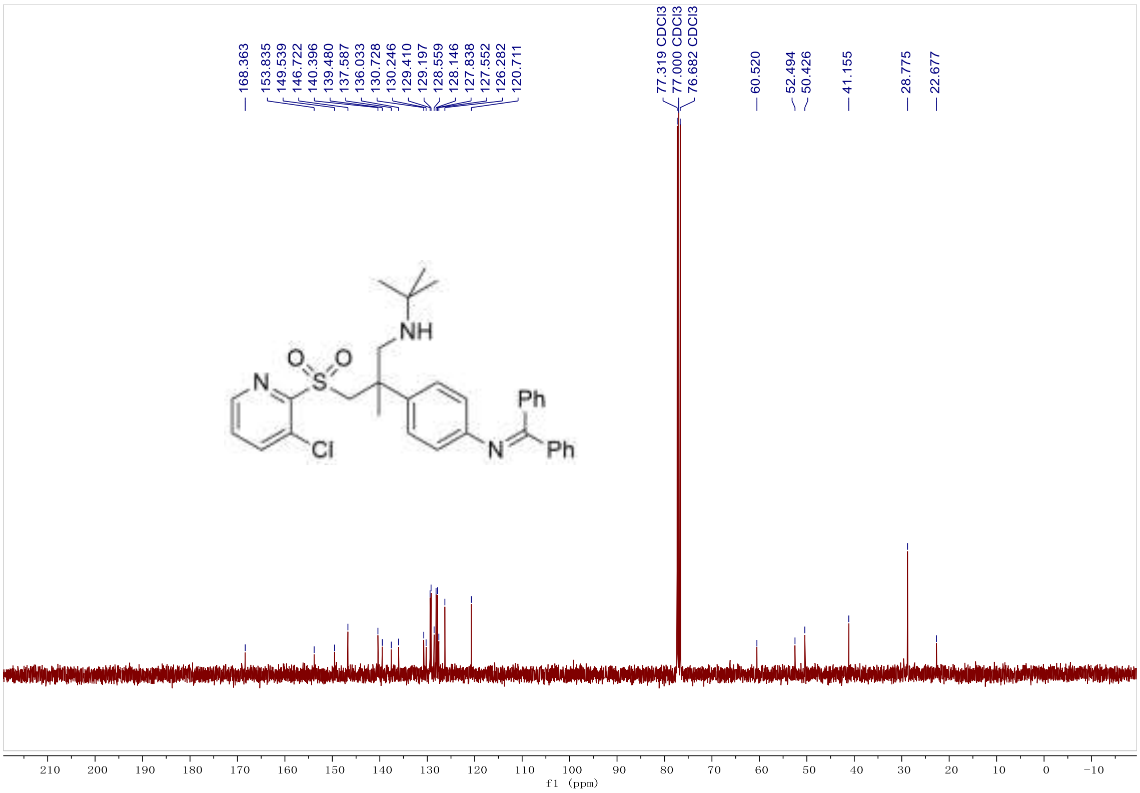

^{13}C−NMR for **6-9**

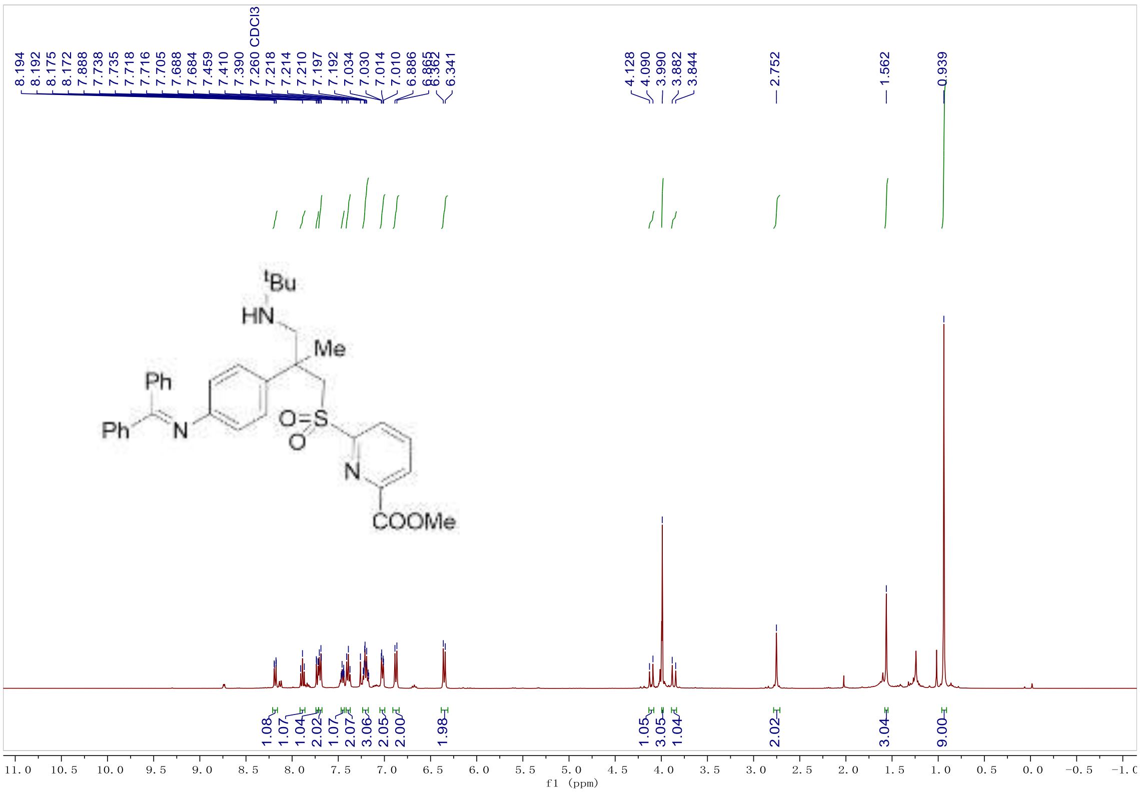

^{1}H-NMR for **6-10**

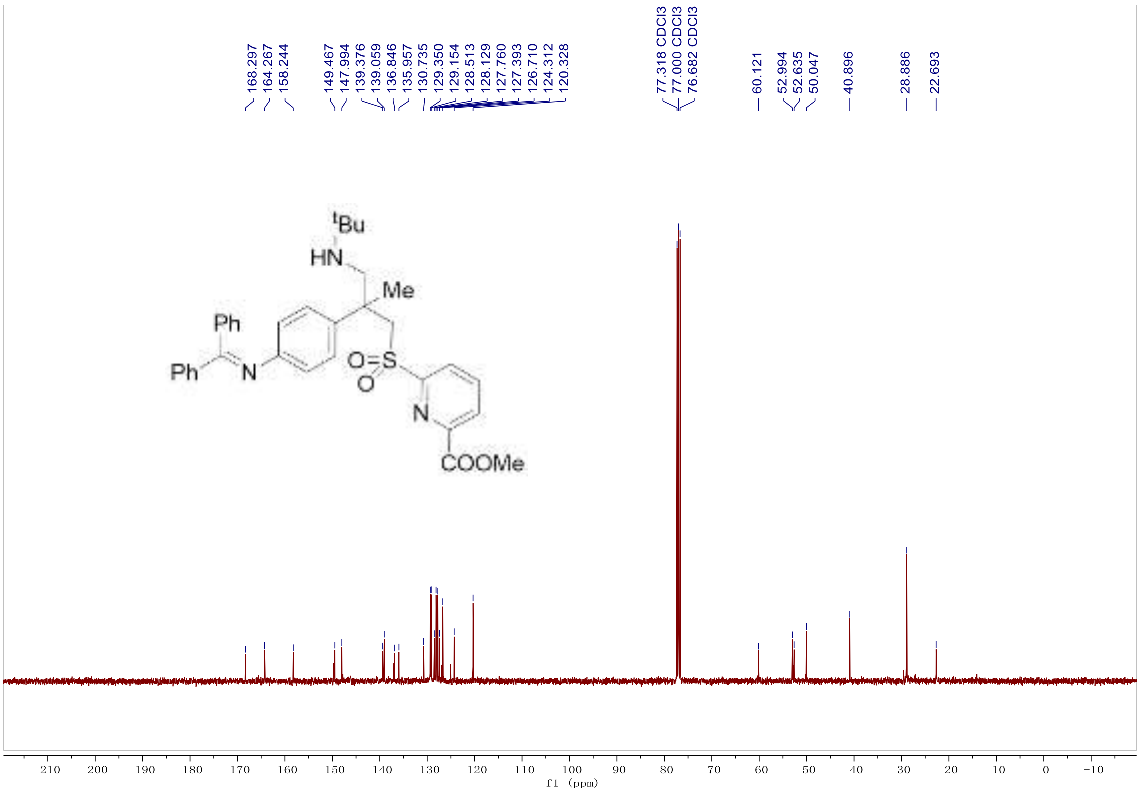

^{13}C−NMR for **6-10**

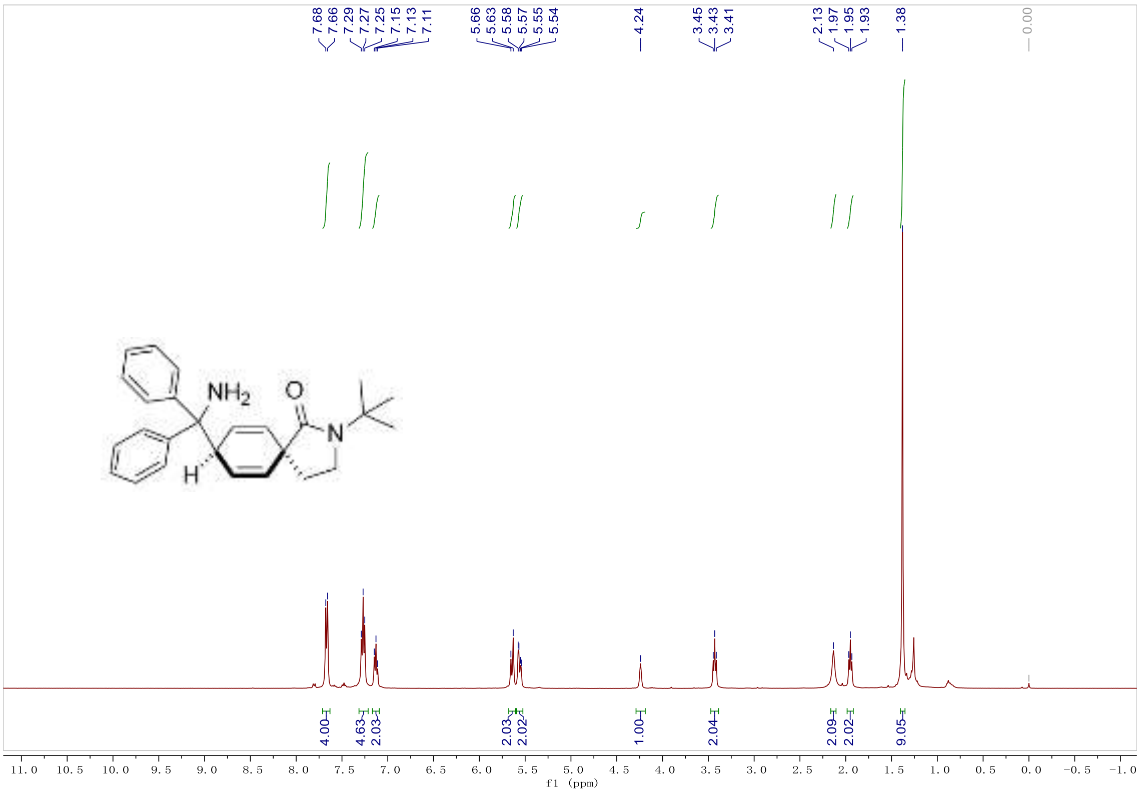

^{1}H-NMR for cis-**3-1**

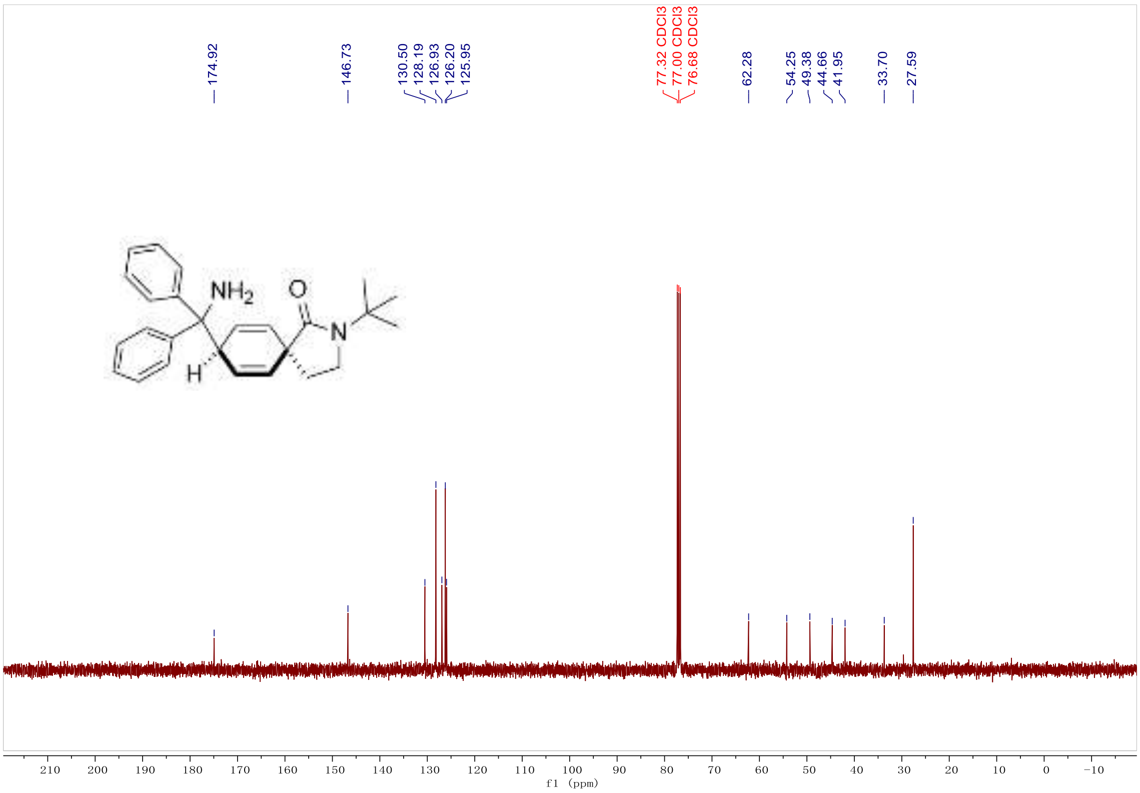

^{13}C−NMR for cis-**3-1**

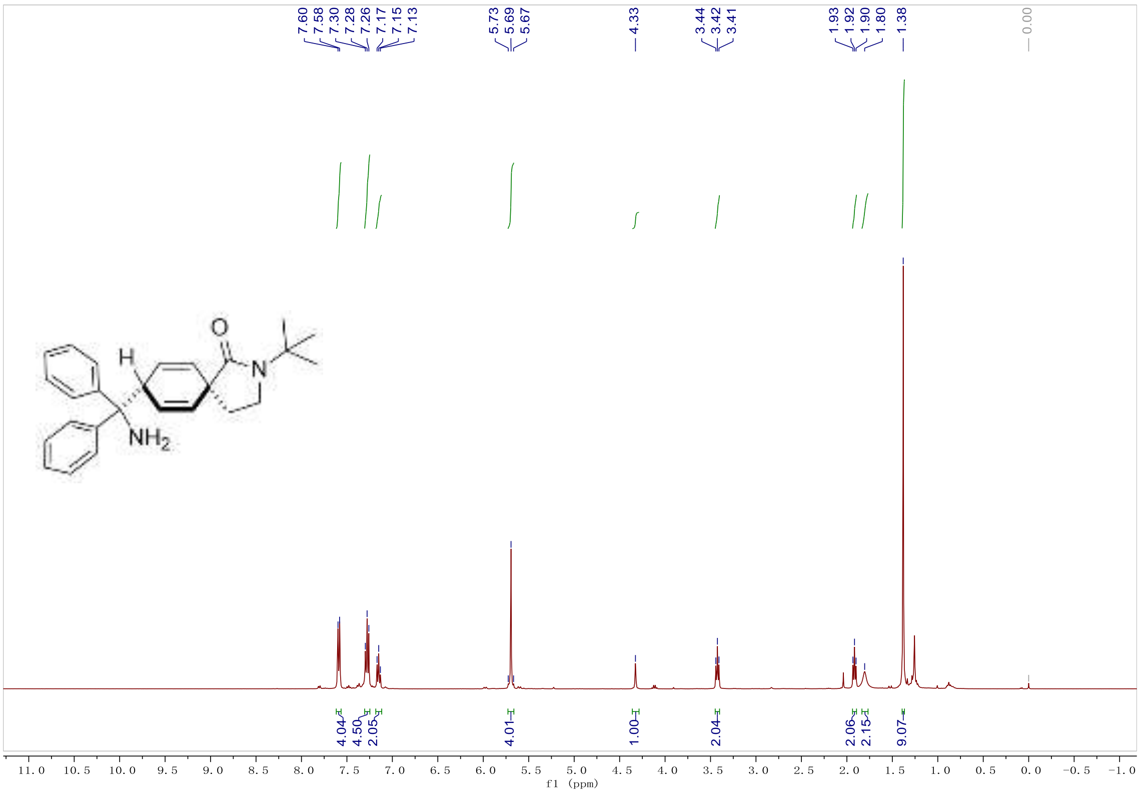

^{1}H-NMR for trans-**3-1**

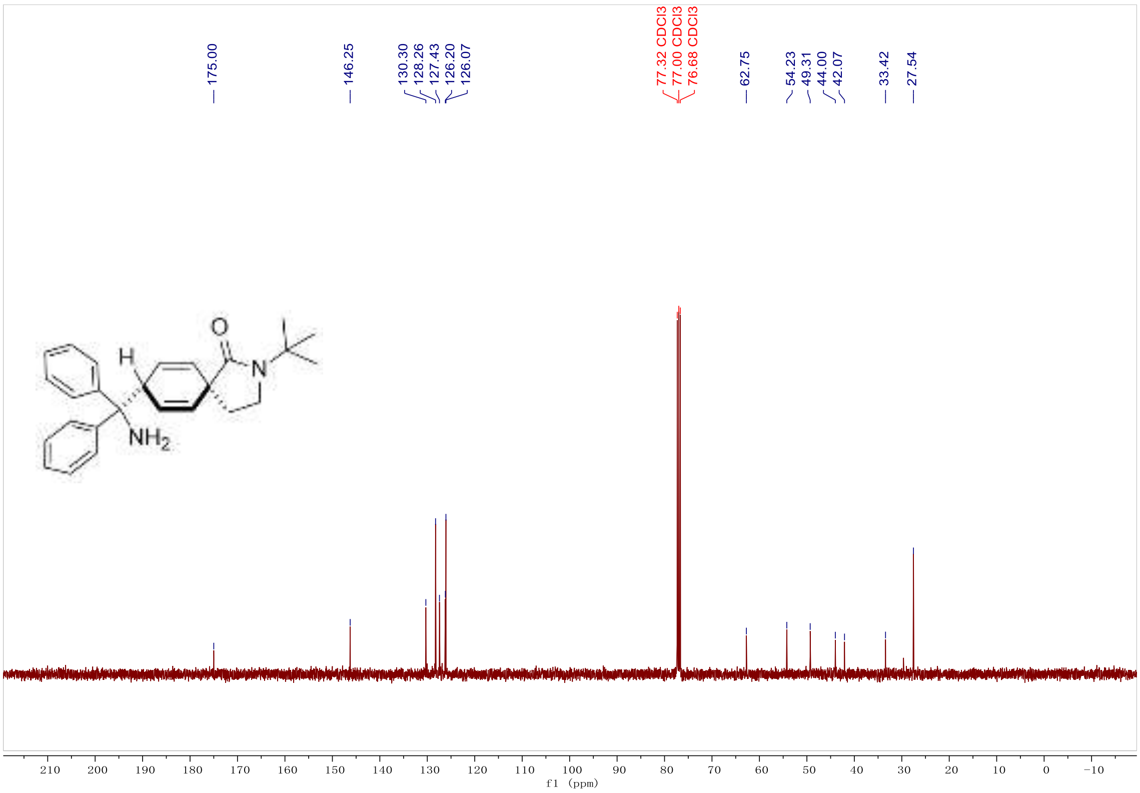

^{13}C−NMR for trans-**3-1**

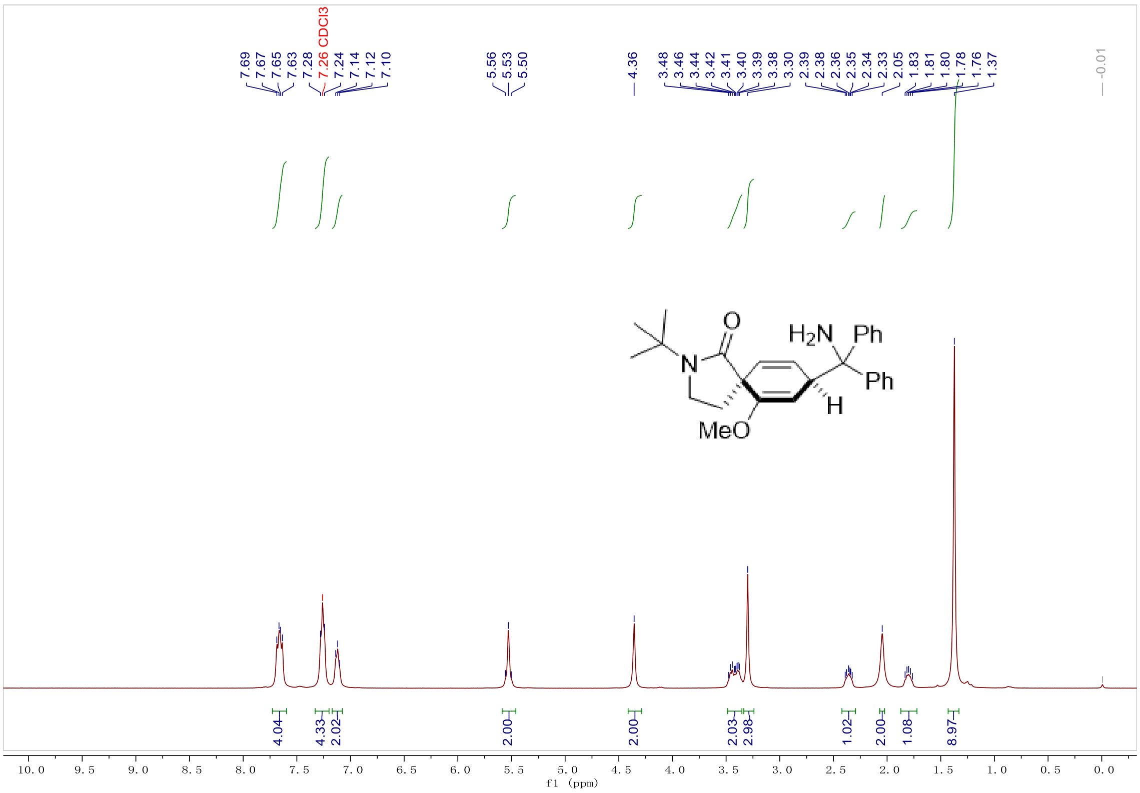

^{1}H-NMR for cis-**3-2**

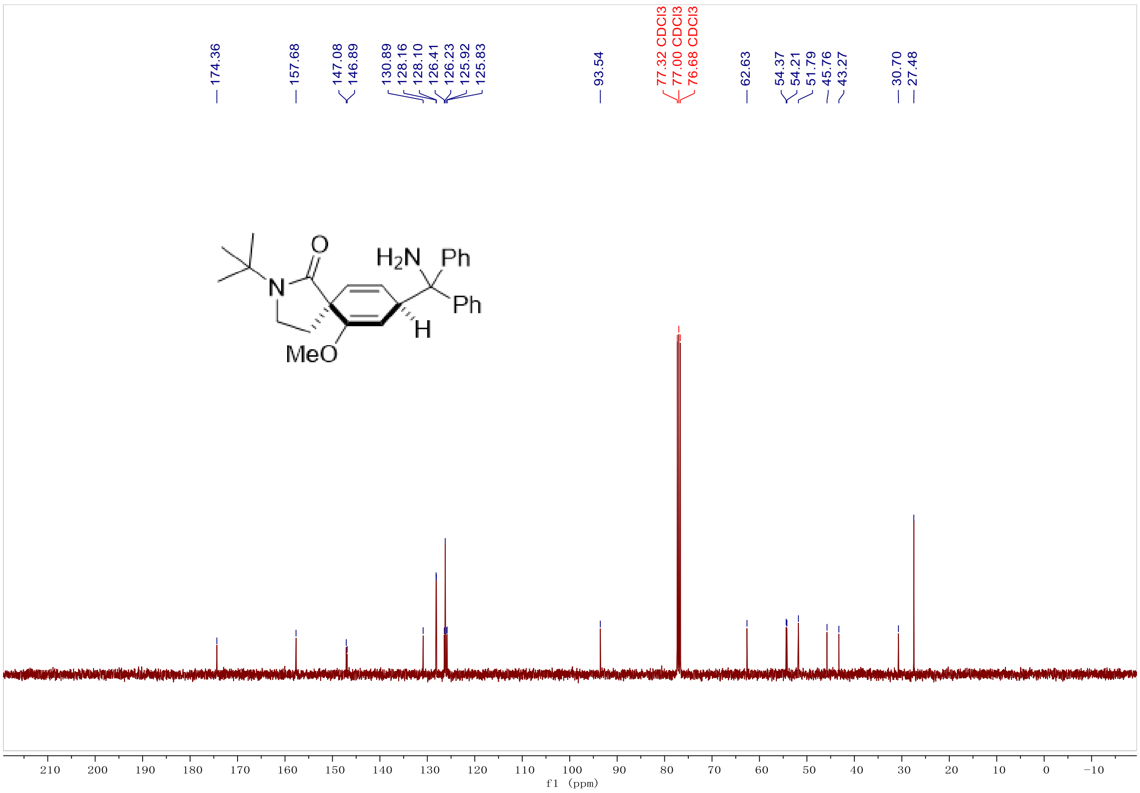

$^{13}C-NMR$ for cis-**3-2**

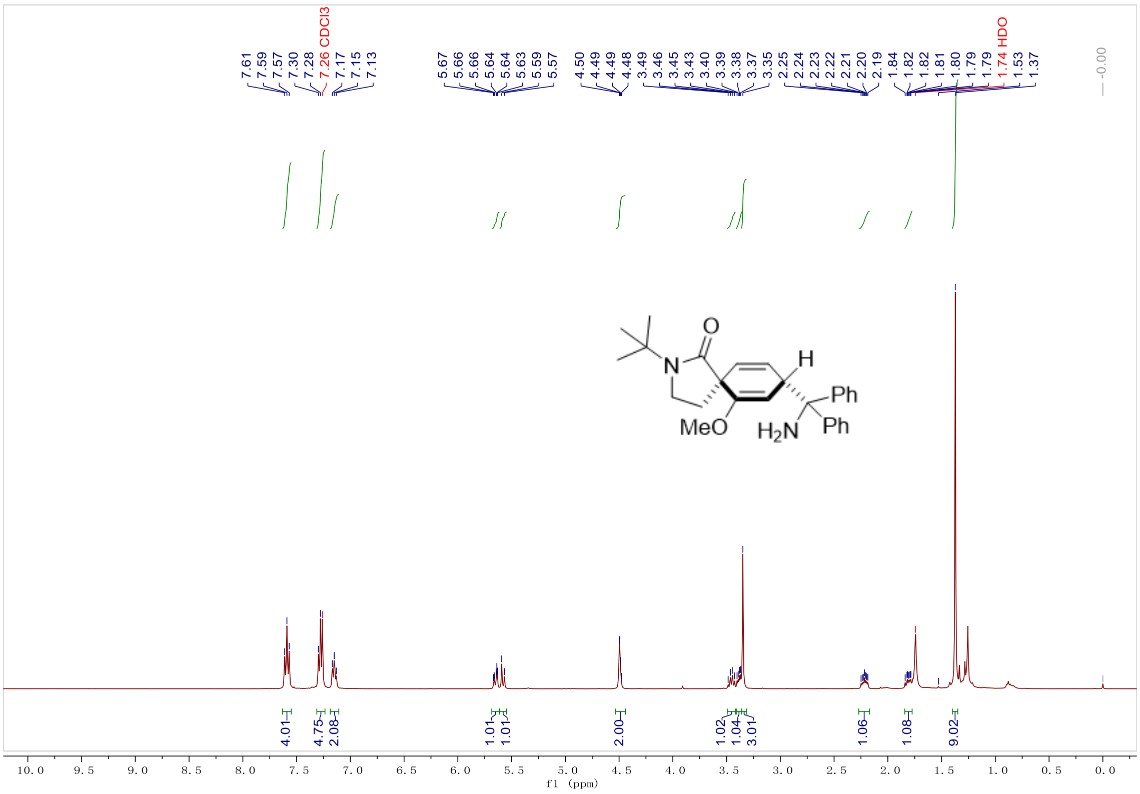

^{1}H-NMR for trans-**3-2**

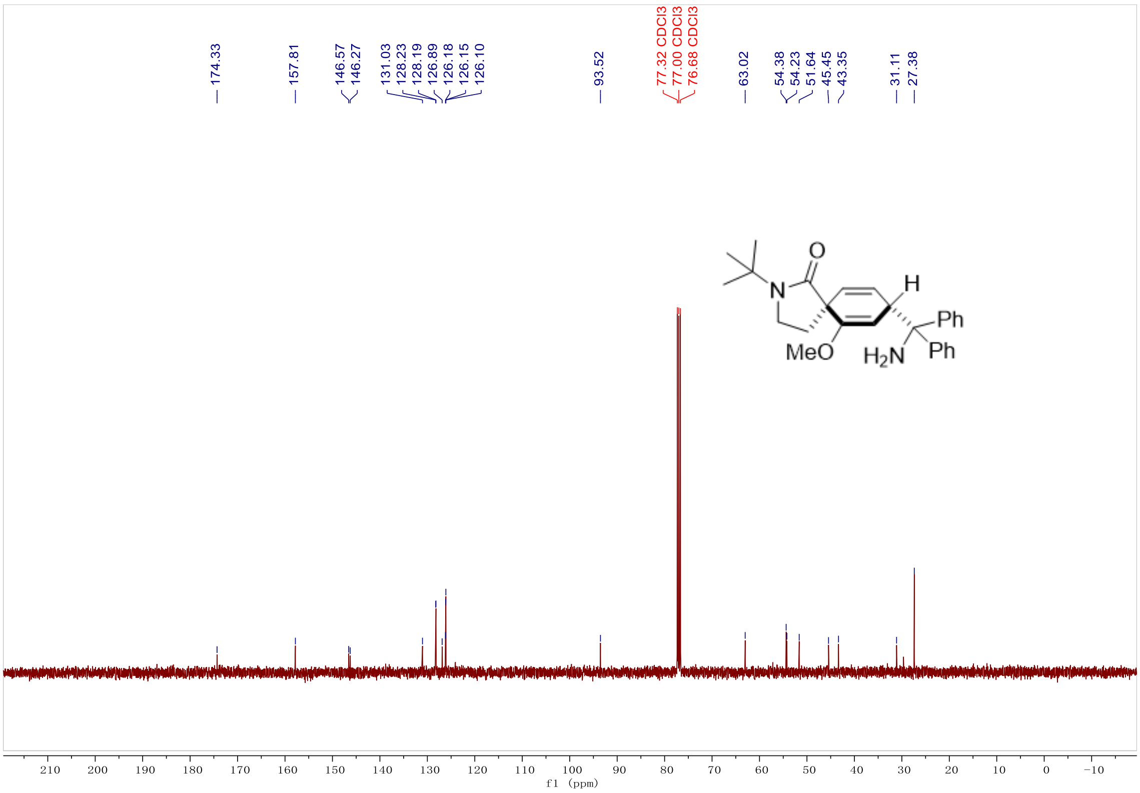

^{13}C−NMR for trans-**3-2**

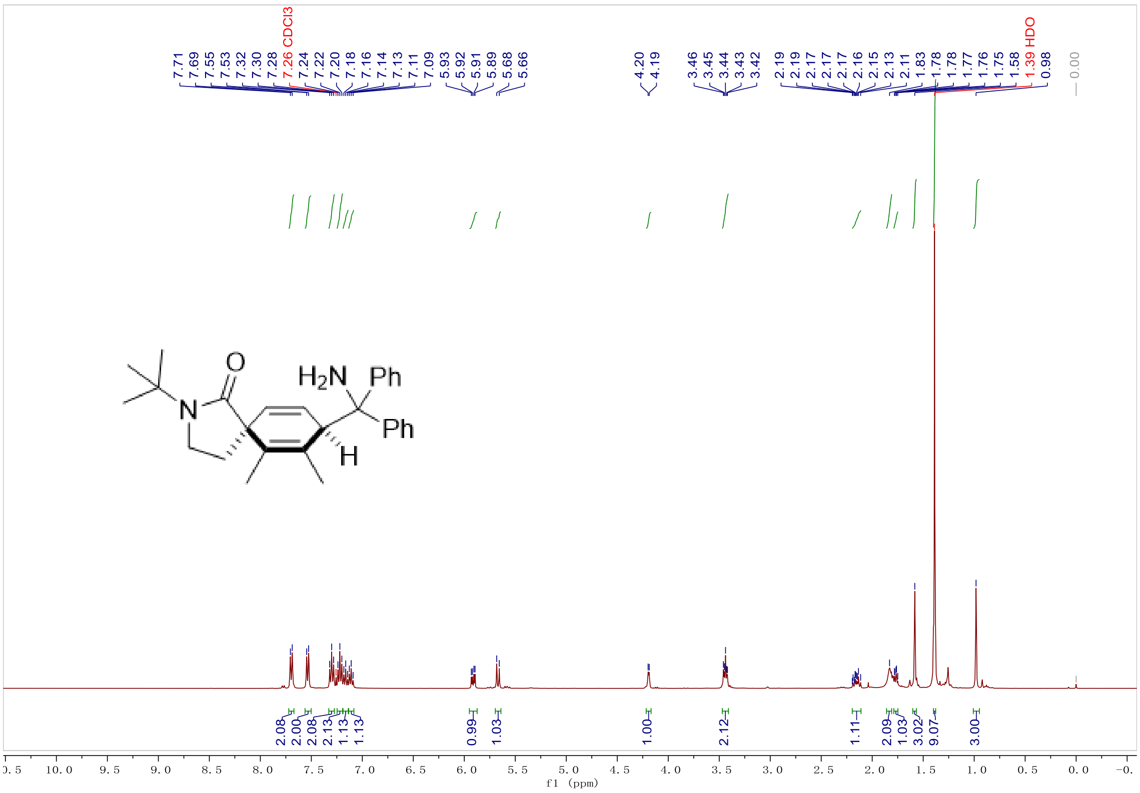

^{1}H-NMR for cis-**3-3**

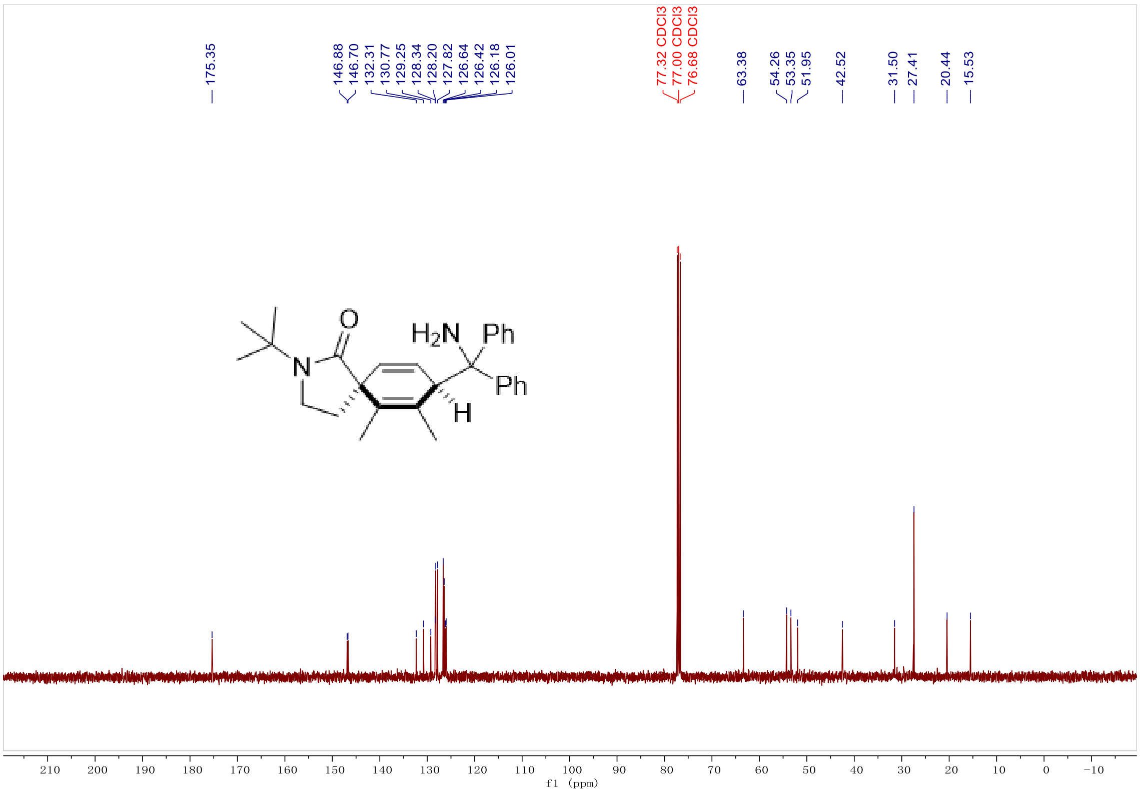

^{13}C−NMR for cis-**3-3**

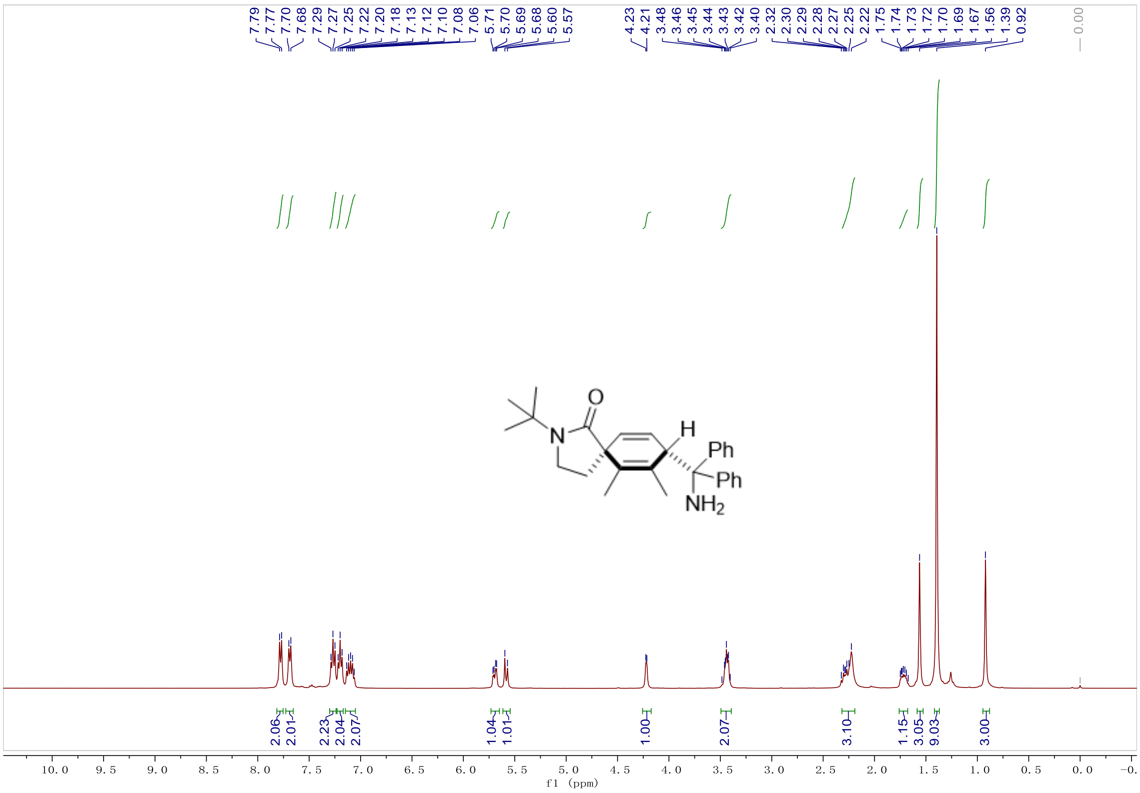

^{1}H-NMR for trans-**3-3**

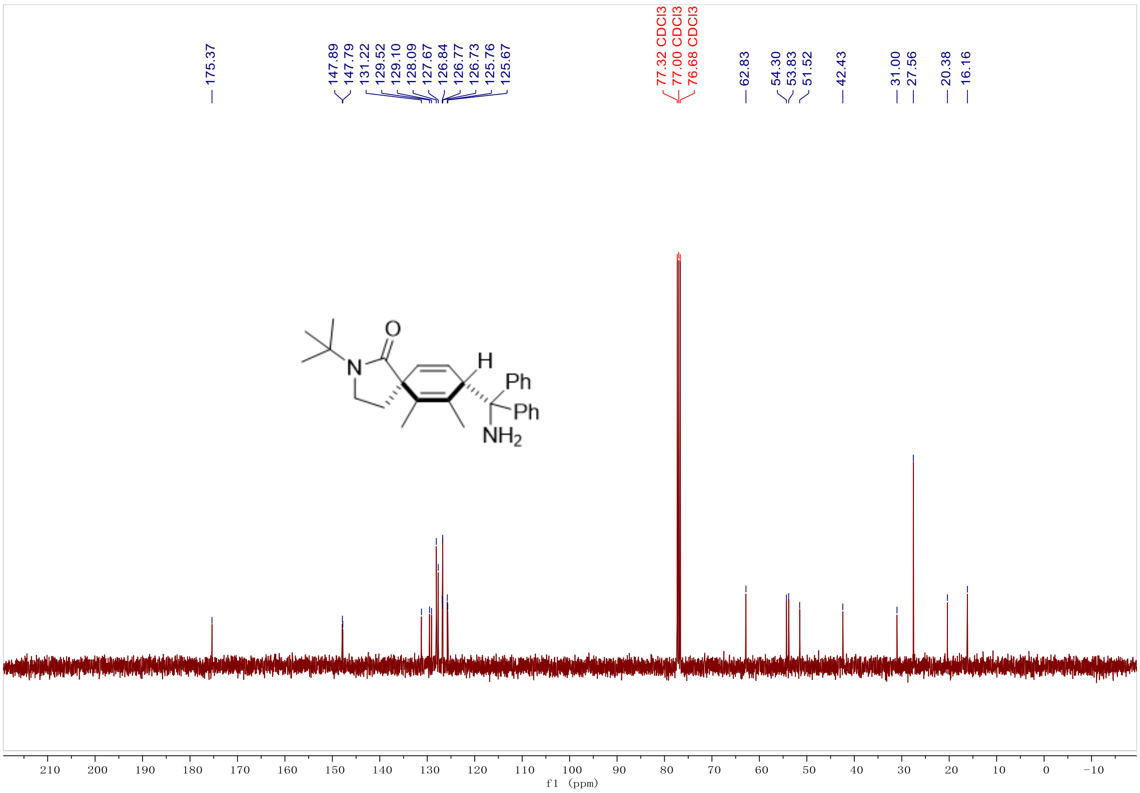

^{13}C−NMR for trans-**3-3**

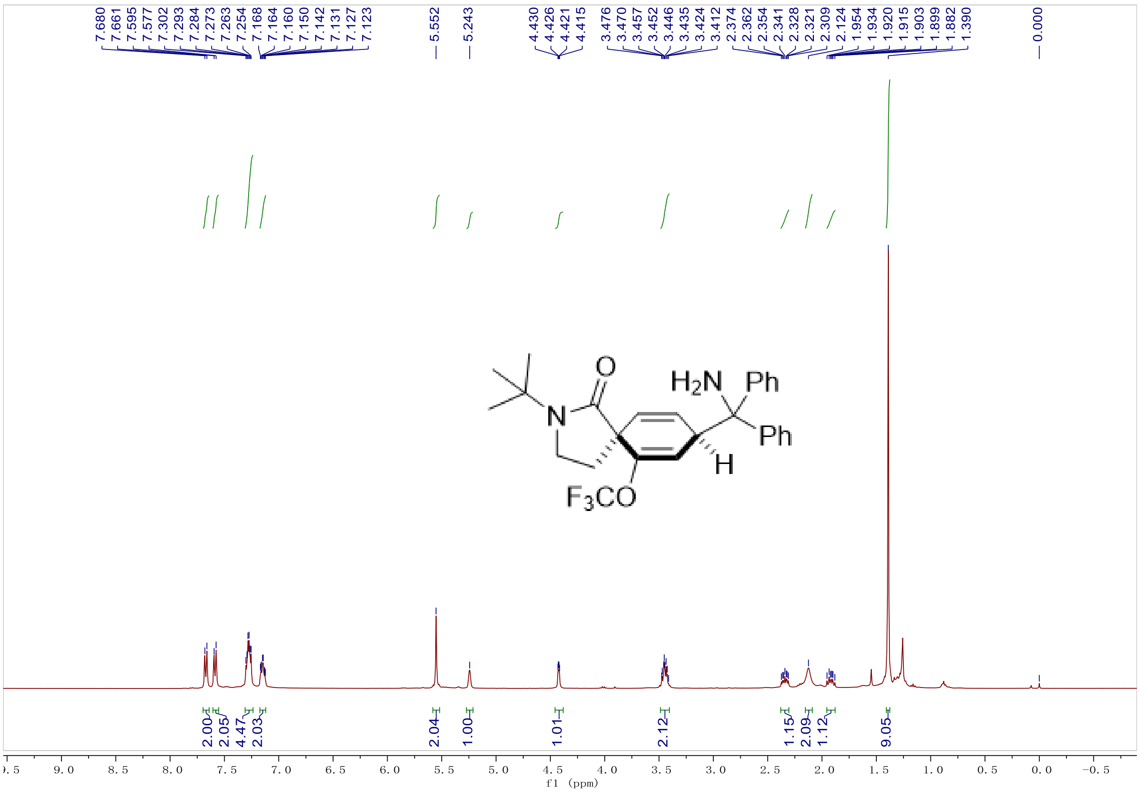

^{1}H-NMR for cis-**3-4**

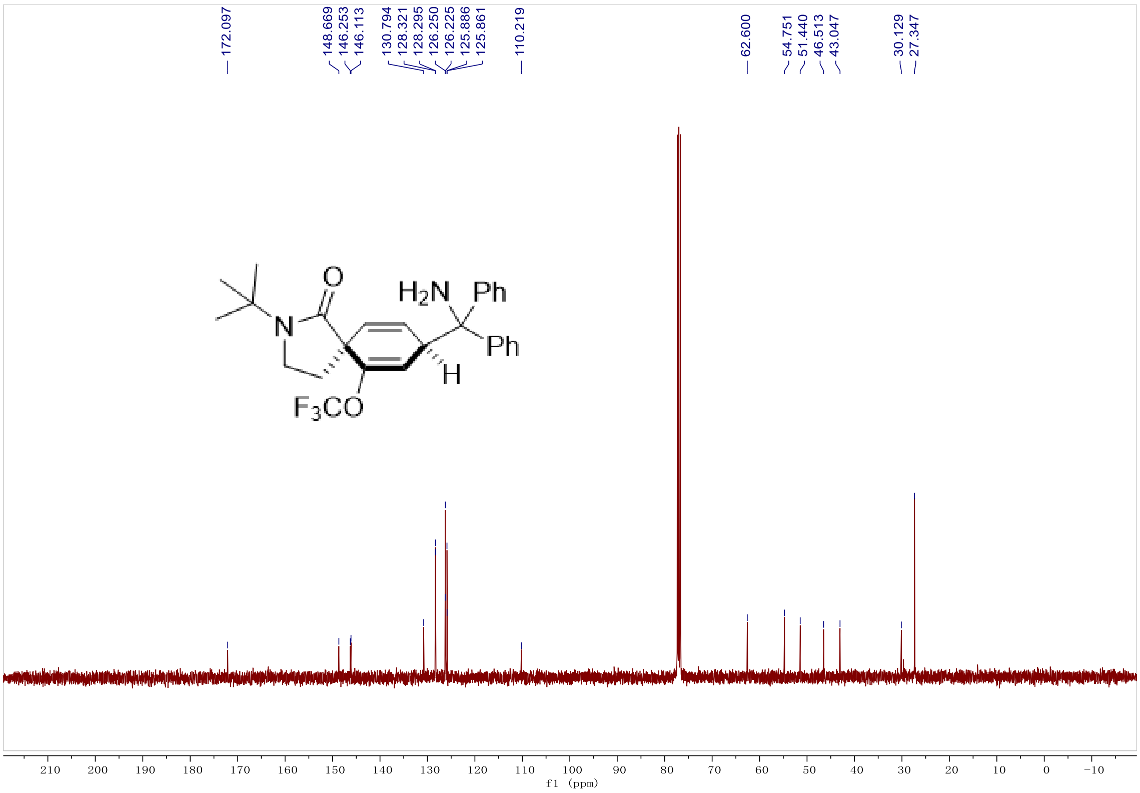

^{13}C−NMR for cis-**3-4**

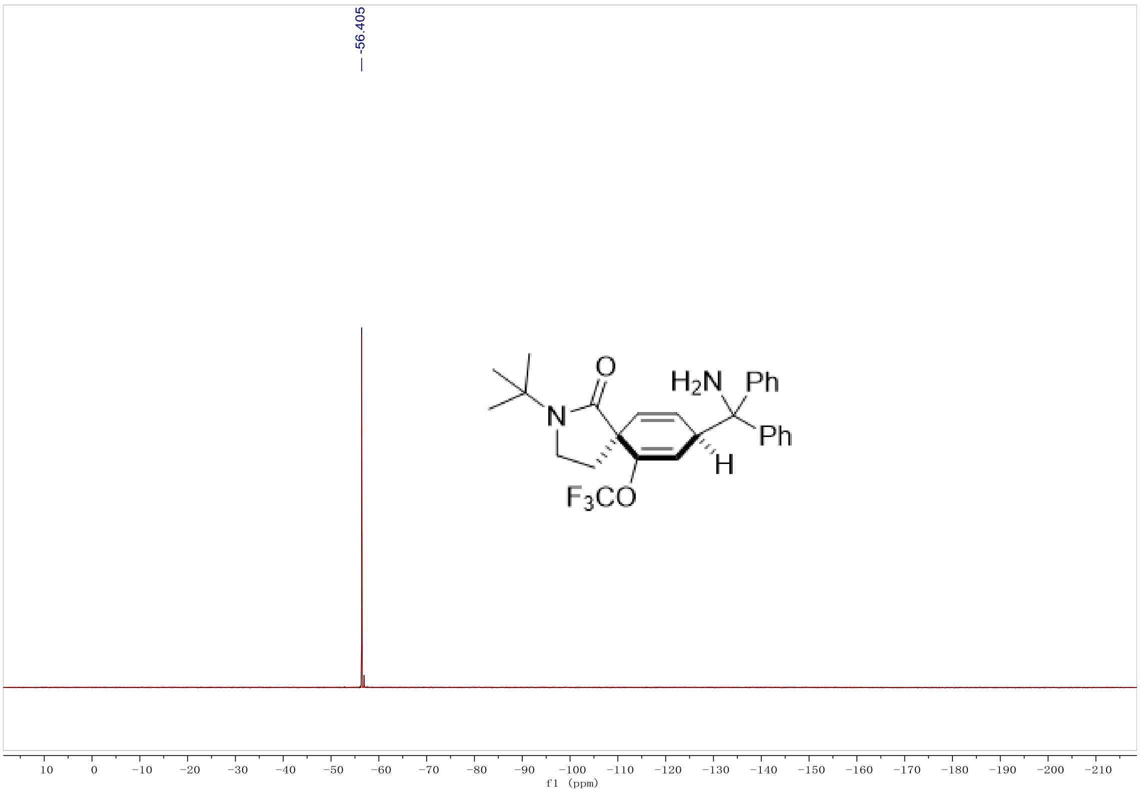

^{19}F-NMR for cis-**3-4**

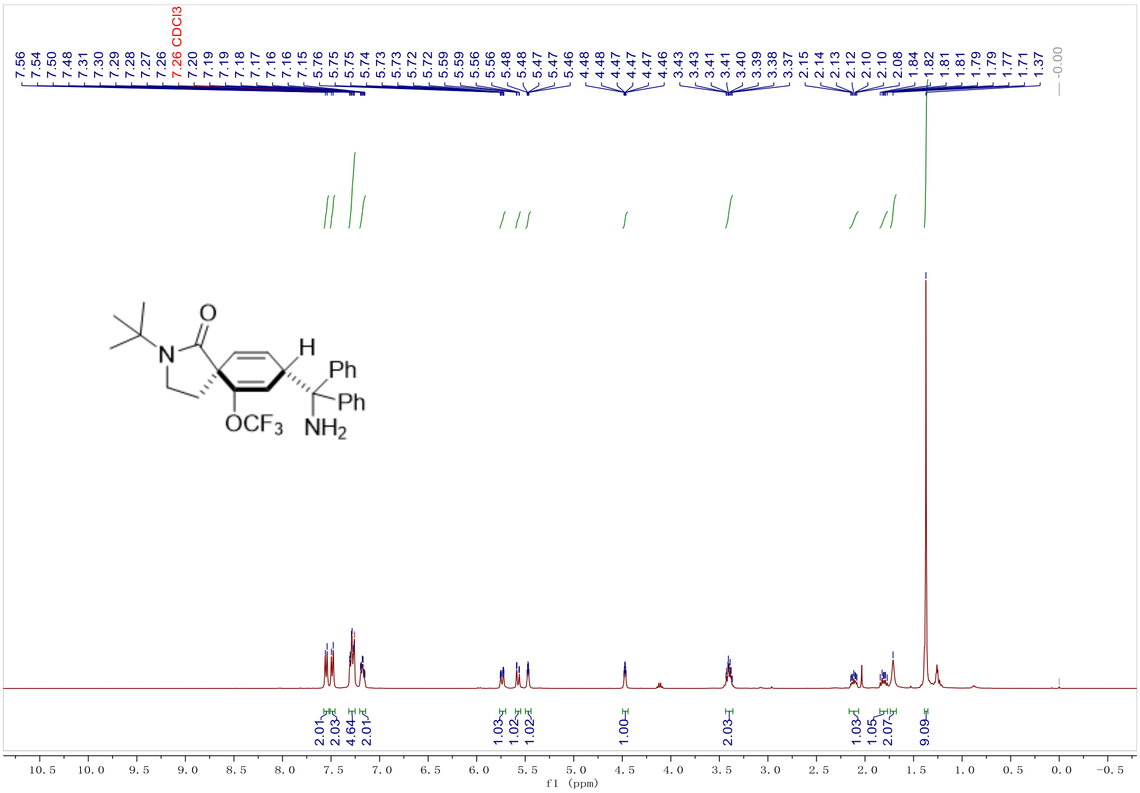

^{1}H-NMR for trans-**3-4**

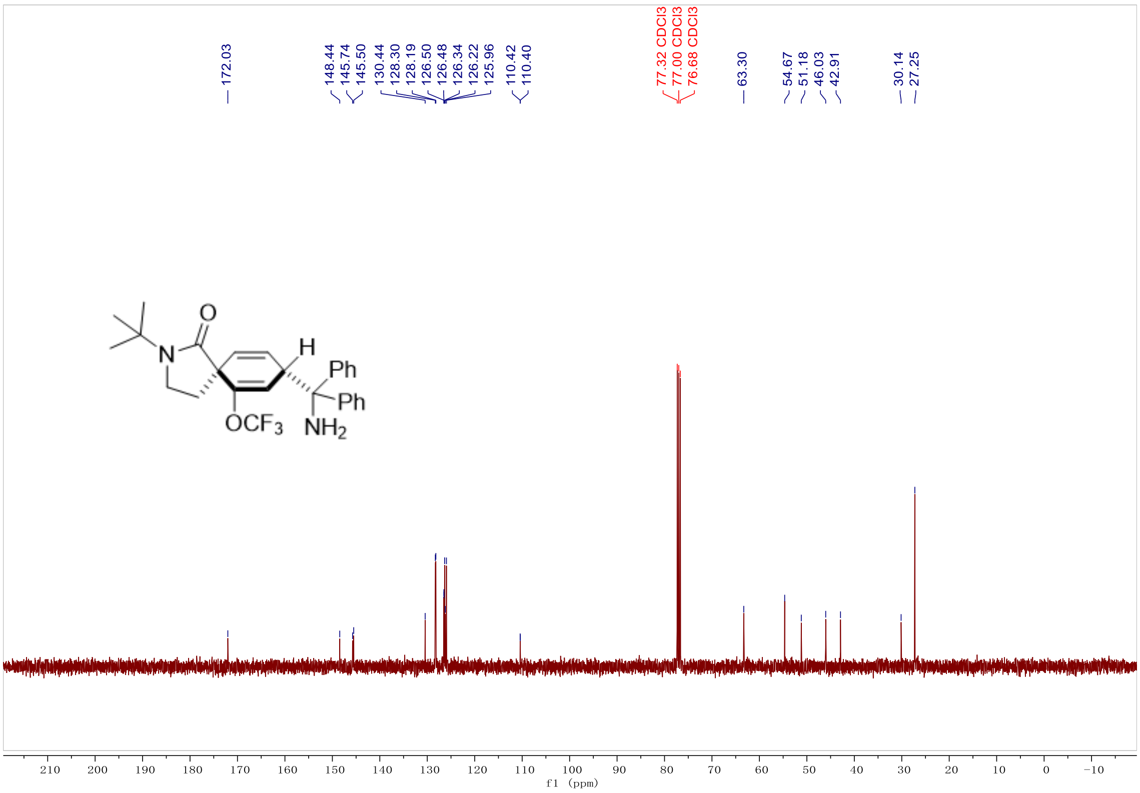

^{13}C−NMR for trans-**3-4**

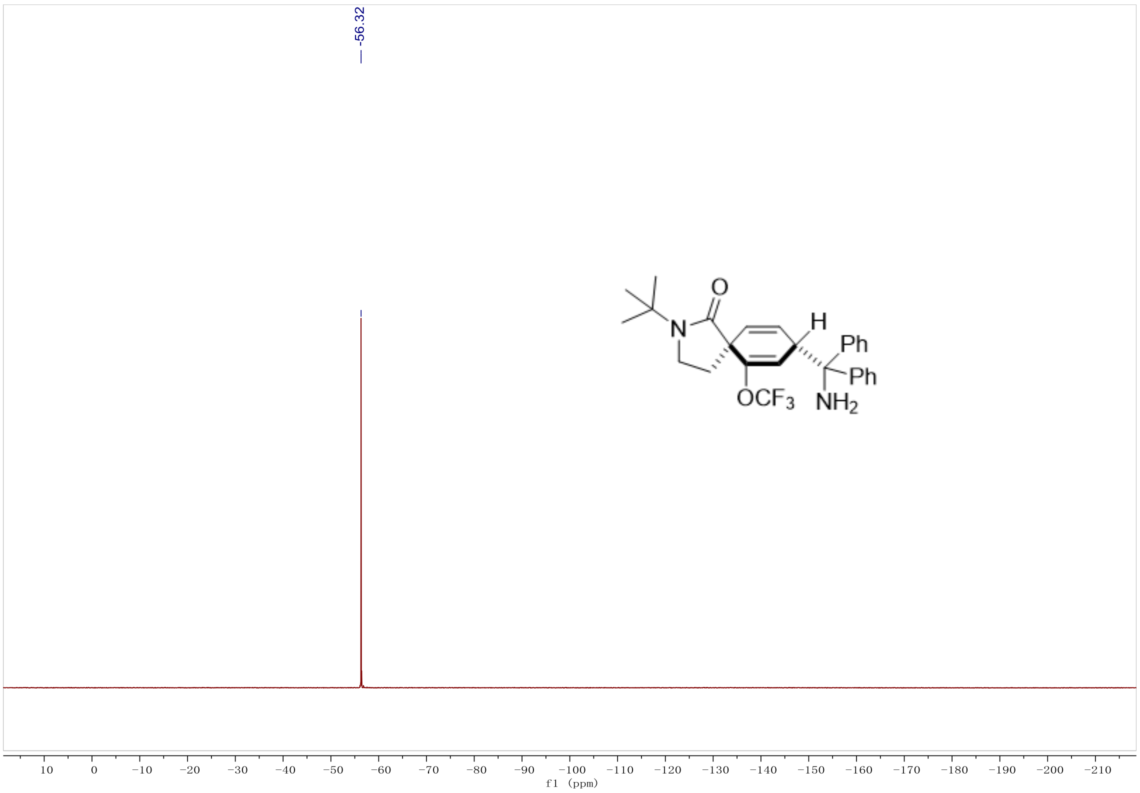

^{19}F-NMR for trans-**3-4**

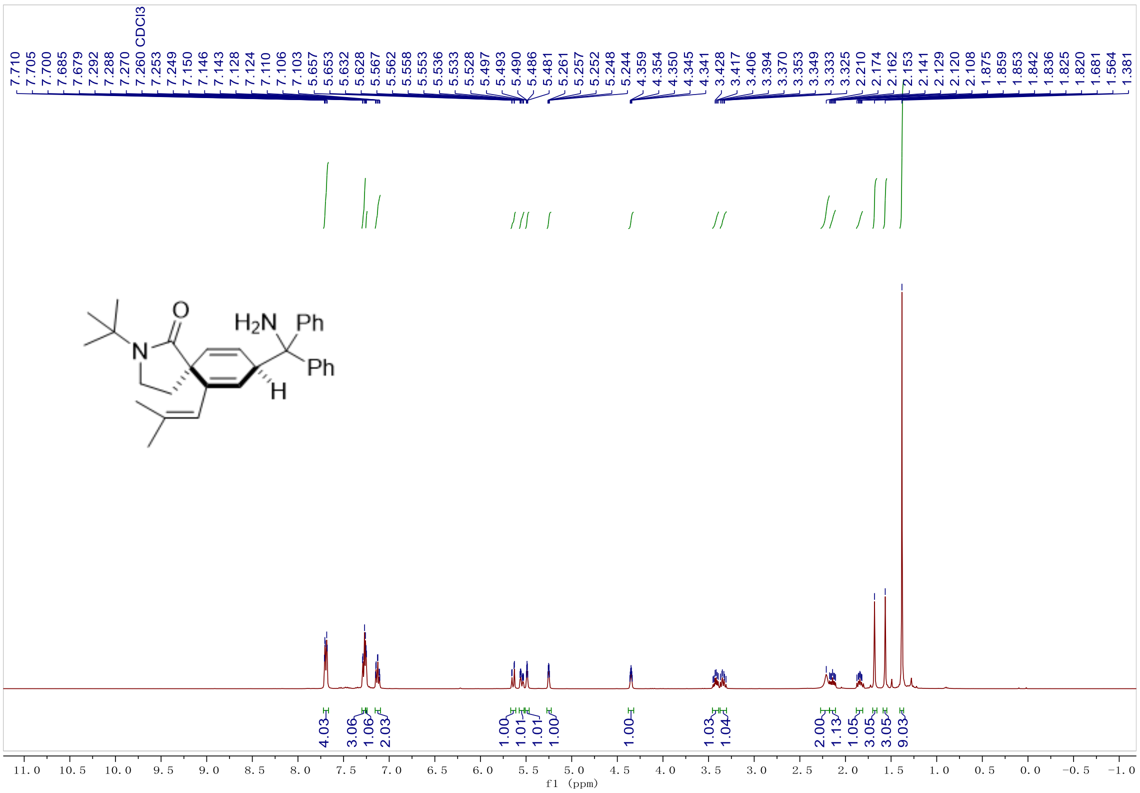

^{1}H-NMR for cis-**3-5**

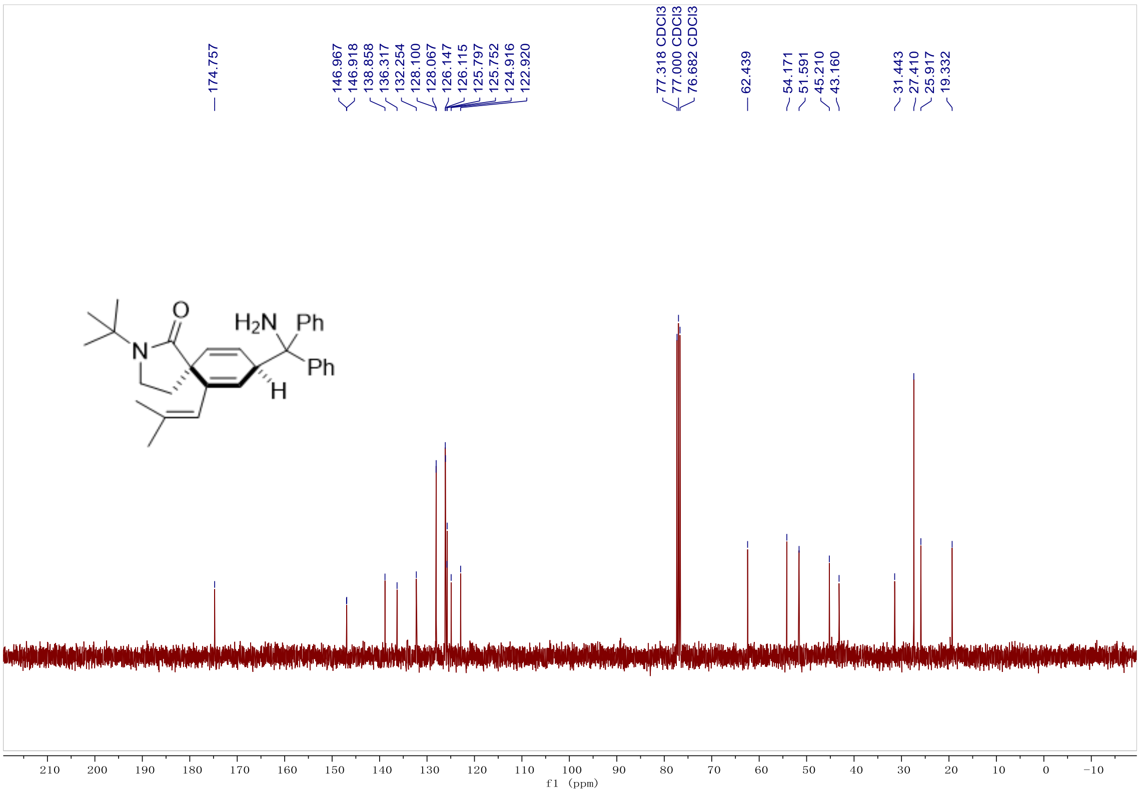

^{13}C−NMR for cis-**3-5**

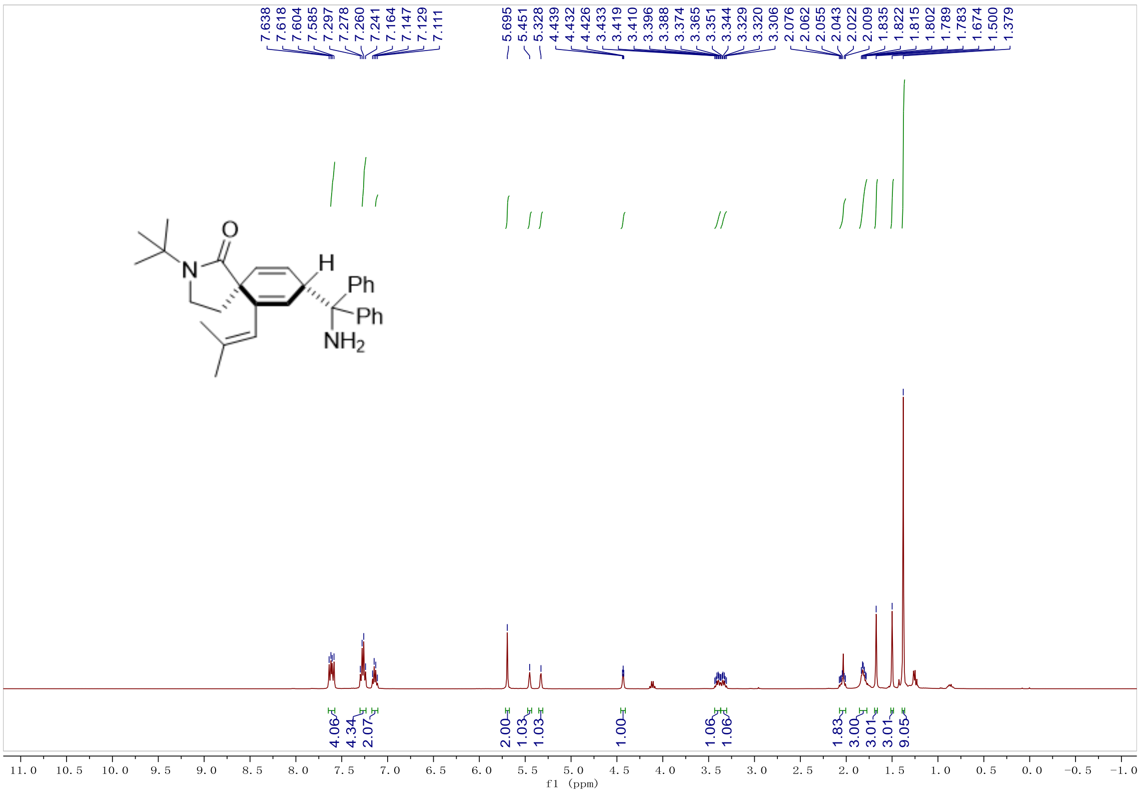

^{1}H-NMR for trans-**3-5**

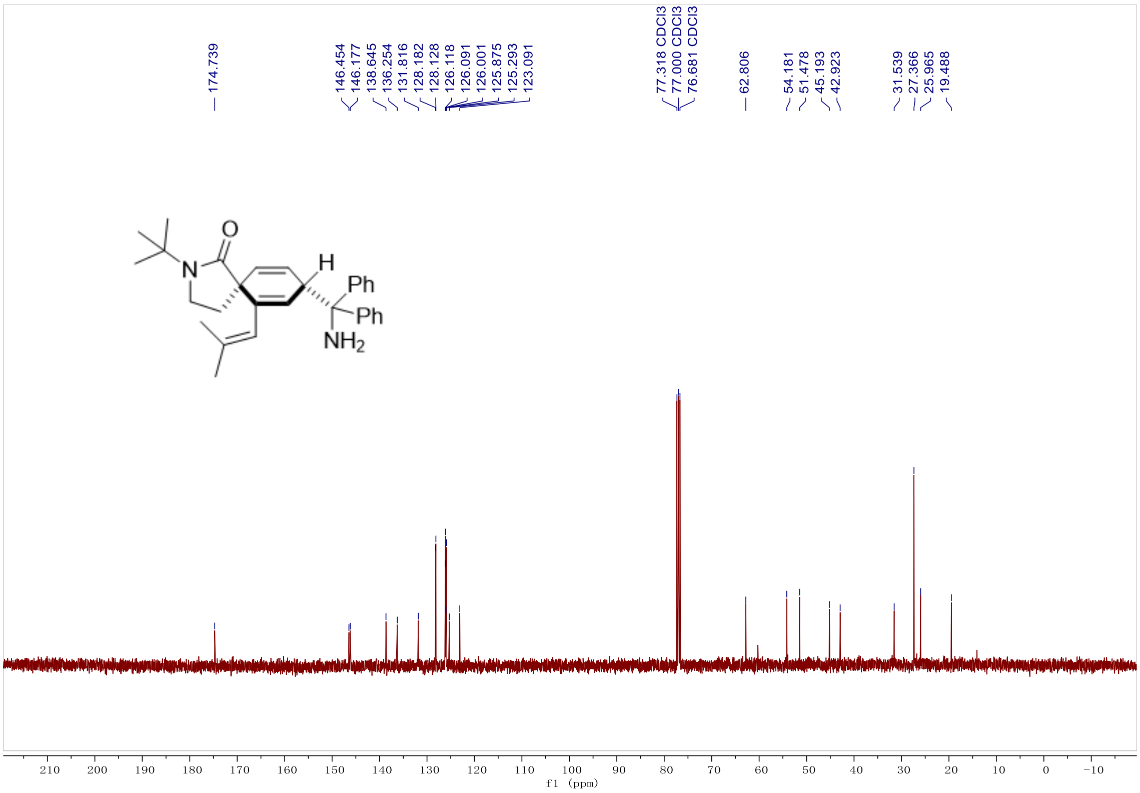

^{13}C−NMR for trans-**3-5**

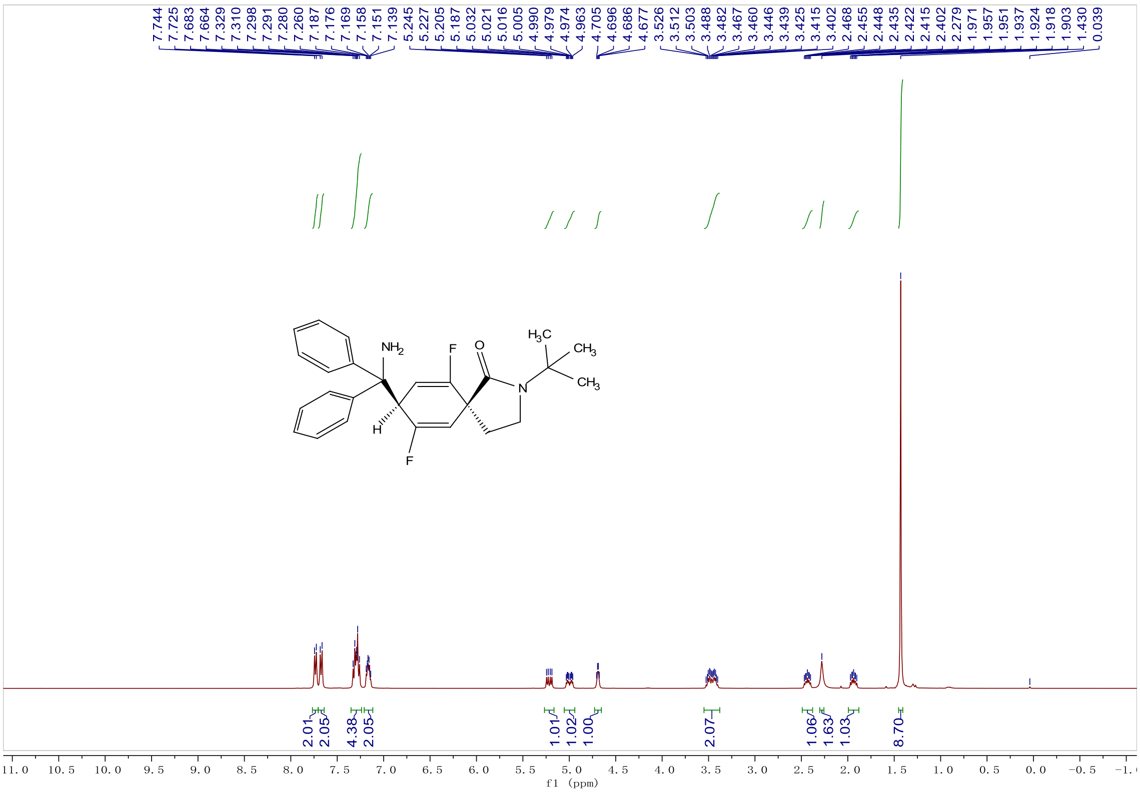

^{1}H-NMR for cis-**3-6**

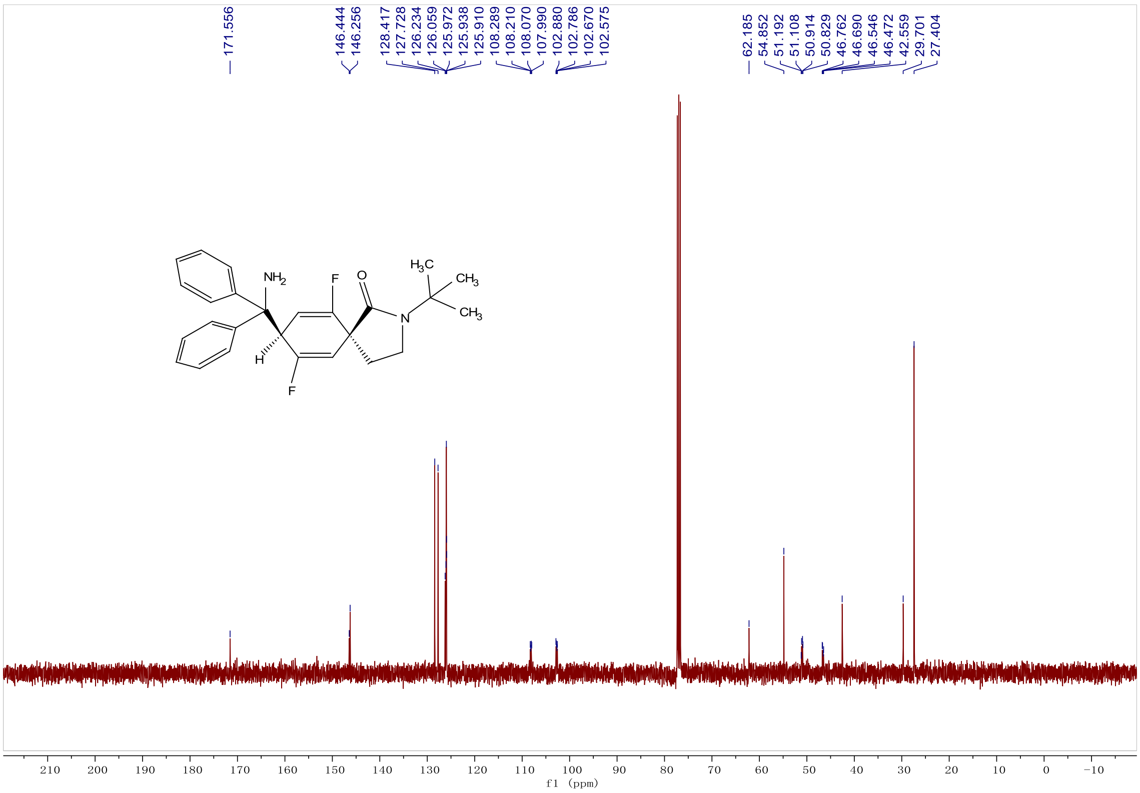

^{13}C−NMR for cis-**3-6**

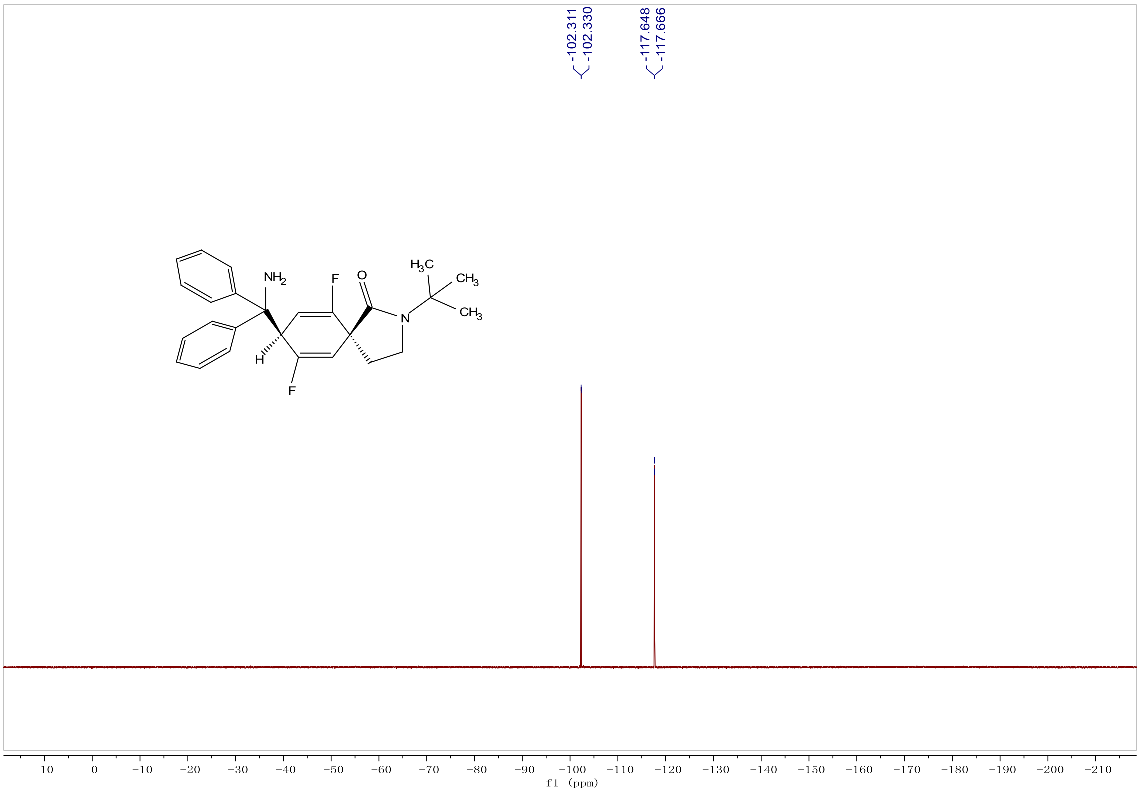

^{19}F-NMR for cis-**3-6**

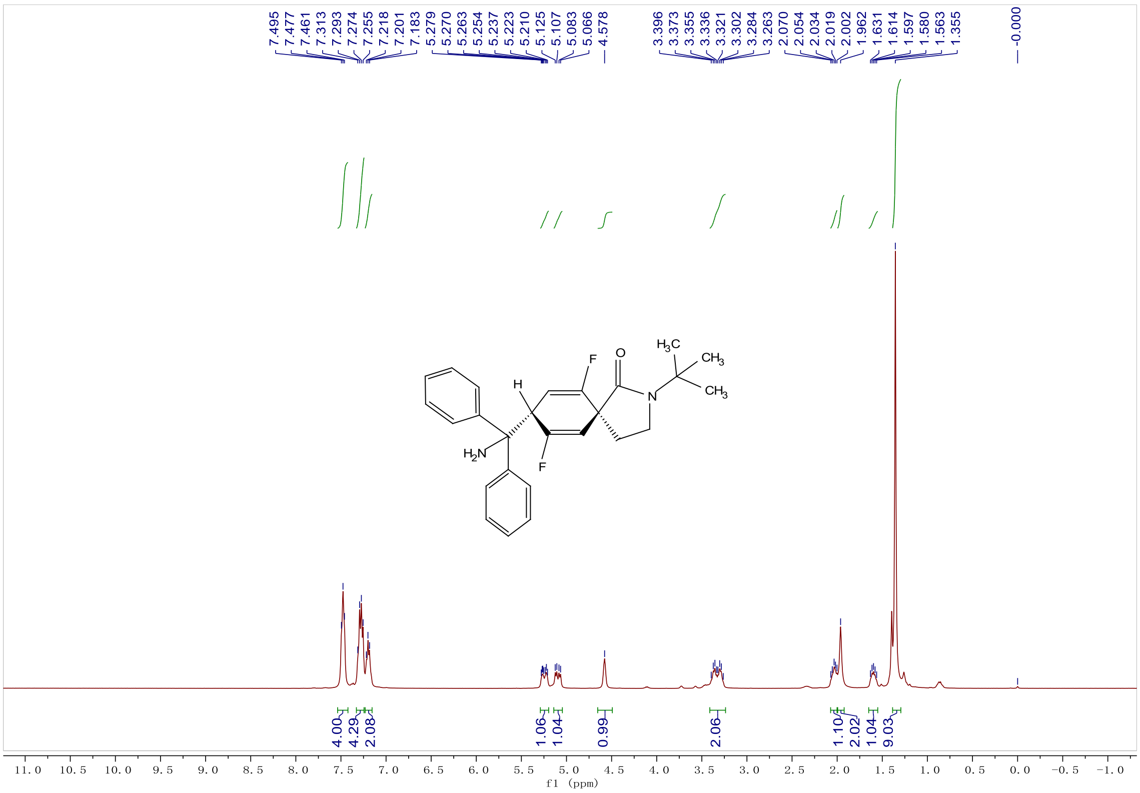

^{1}H-NMR for trans-**3-6**

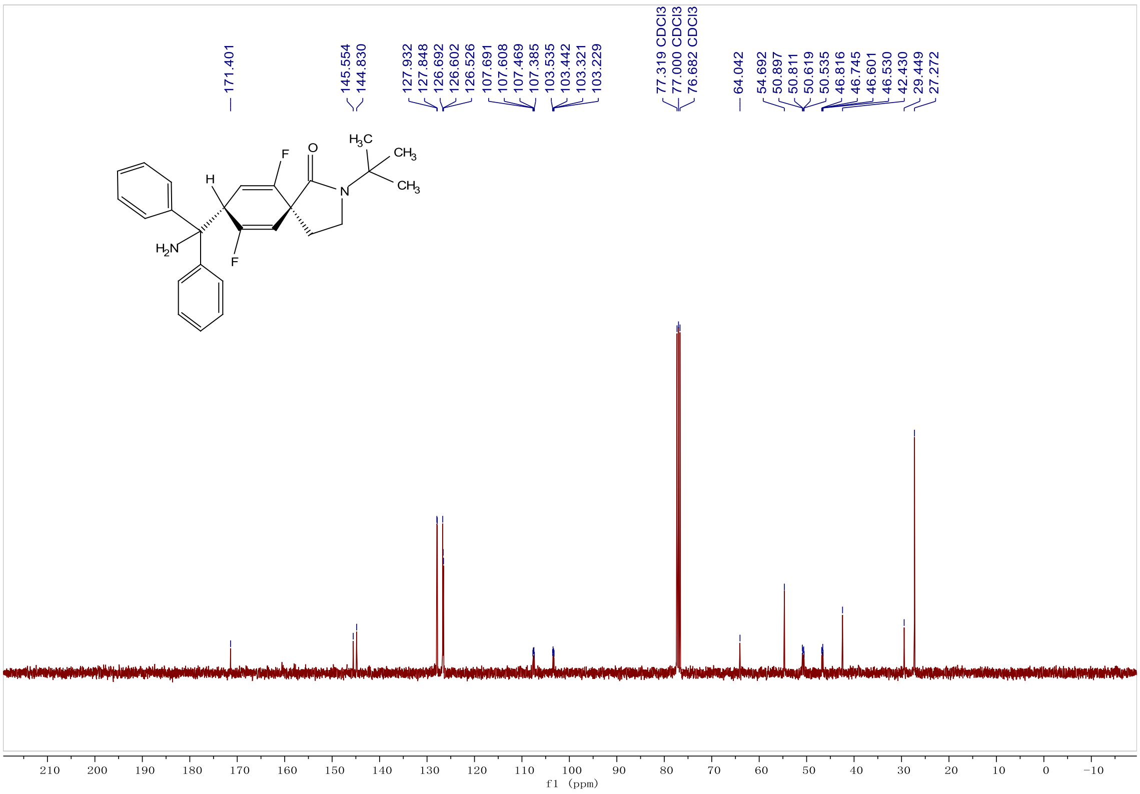

^{13}C−NMR for trans-**3-6**

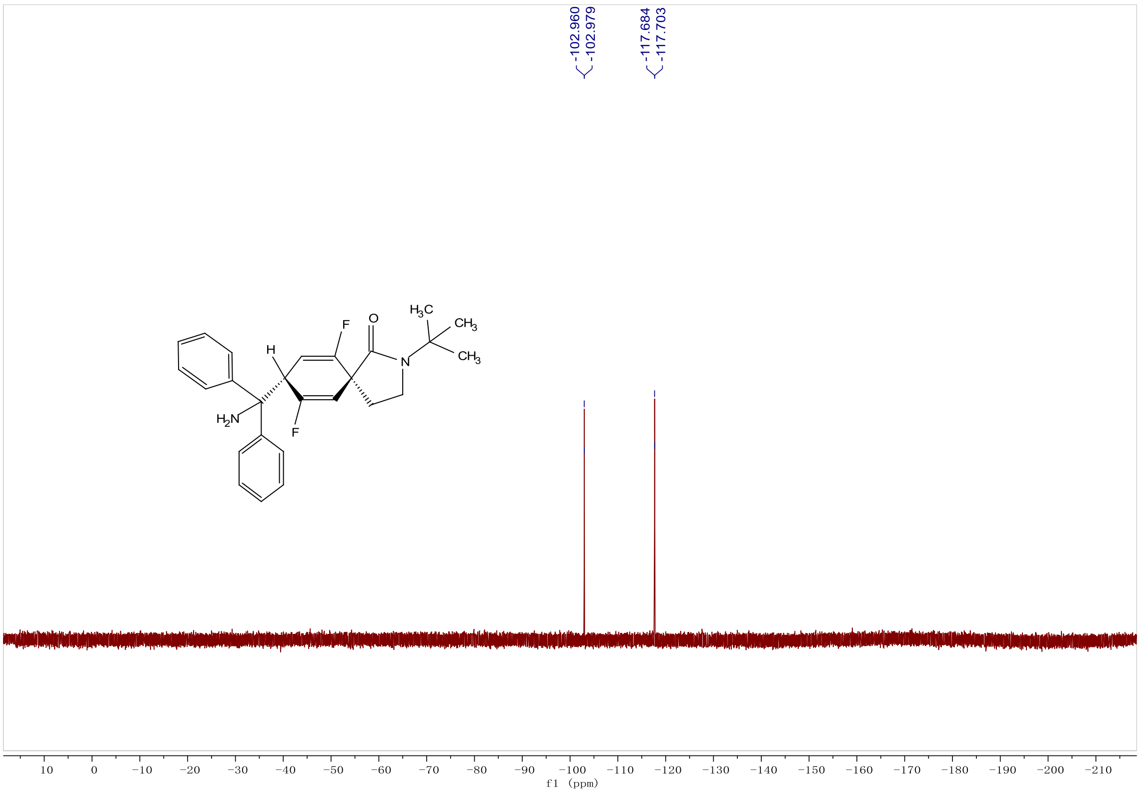

^{19}F-NMR for trans-**3-6**

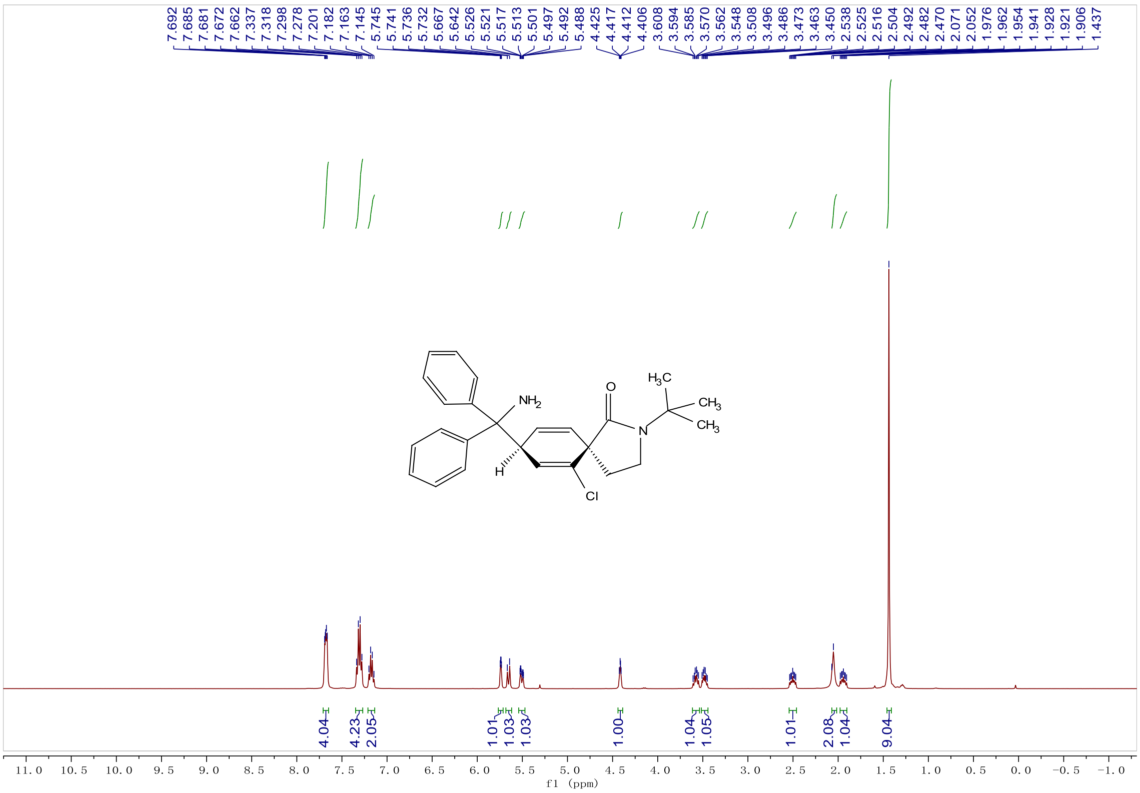

^{1}H-NMR for cis-**3-7**

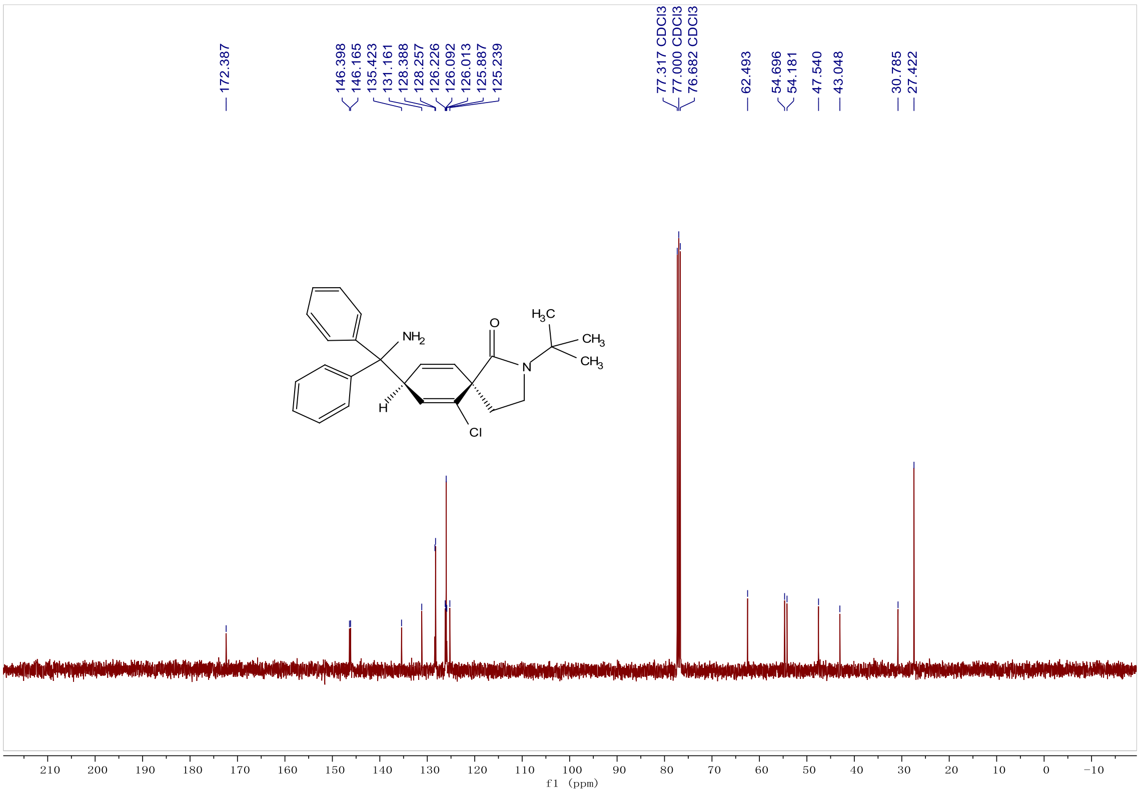

^{13}C−NMR for cis-**3-7**

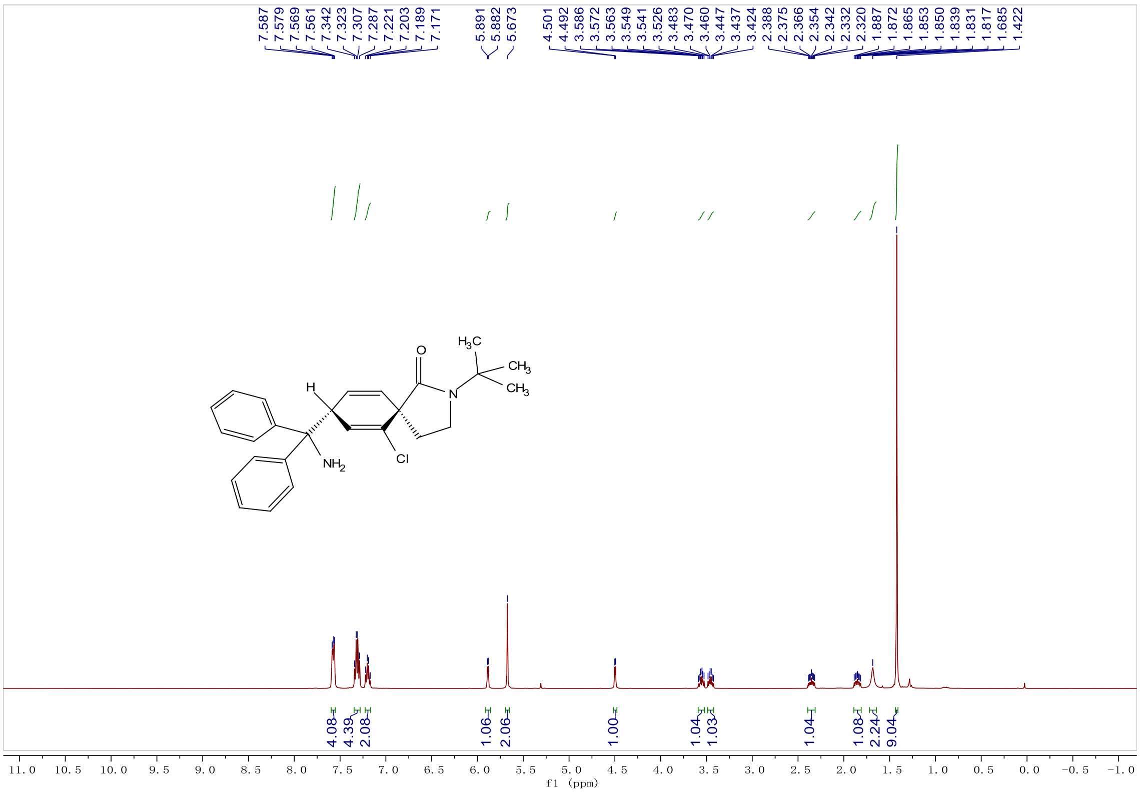

^{1}H-NMR for trans-**3-7**

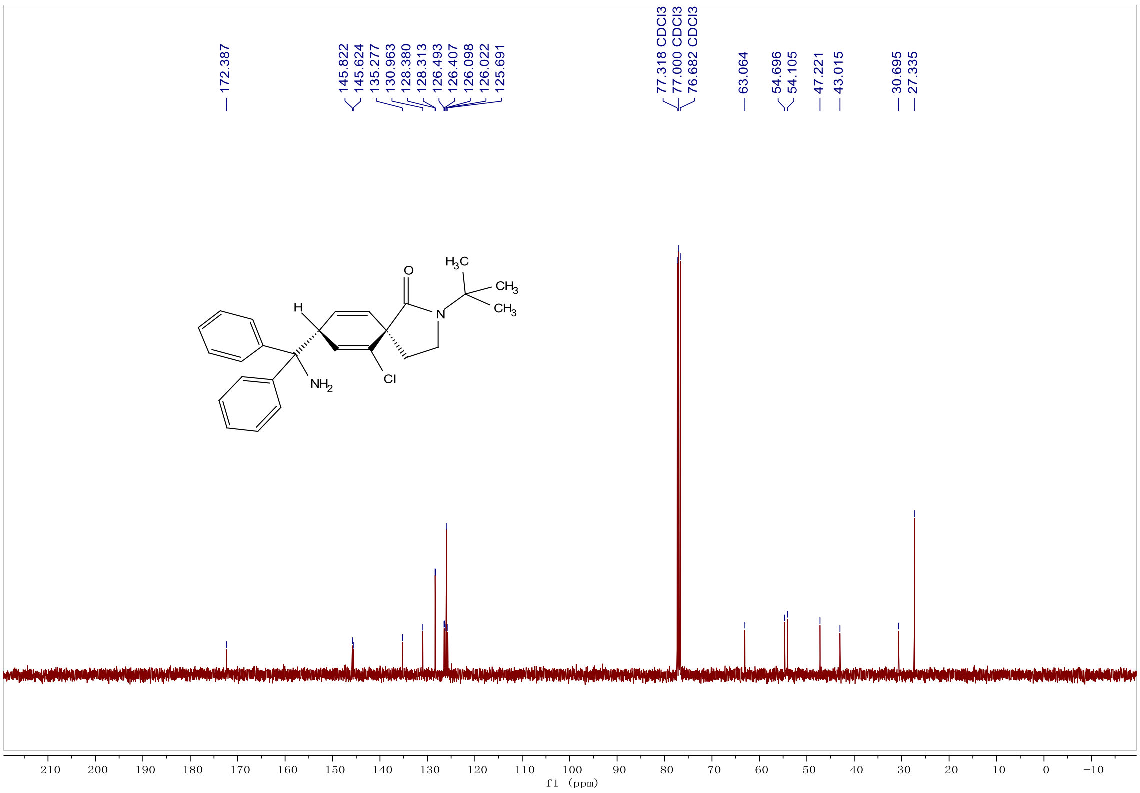

^{13}C−NMR for trans-**3-7**

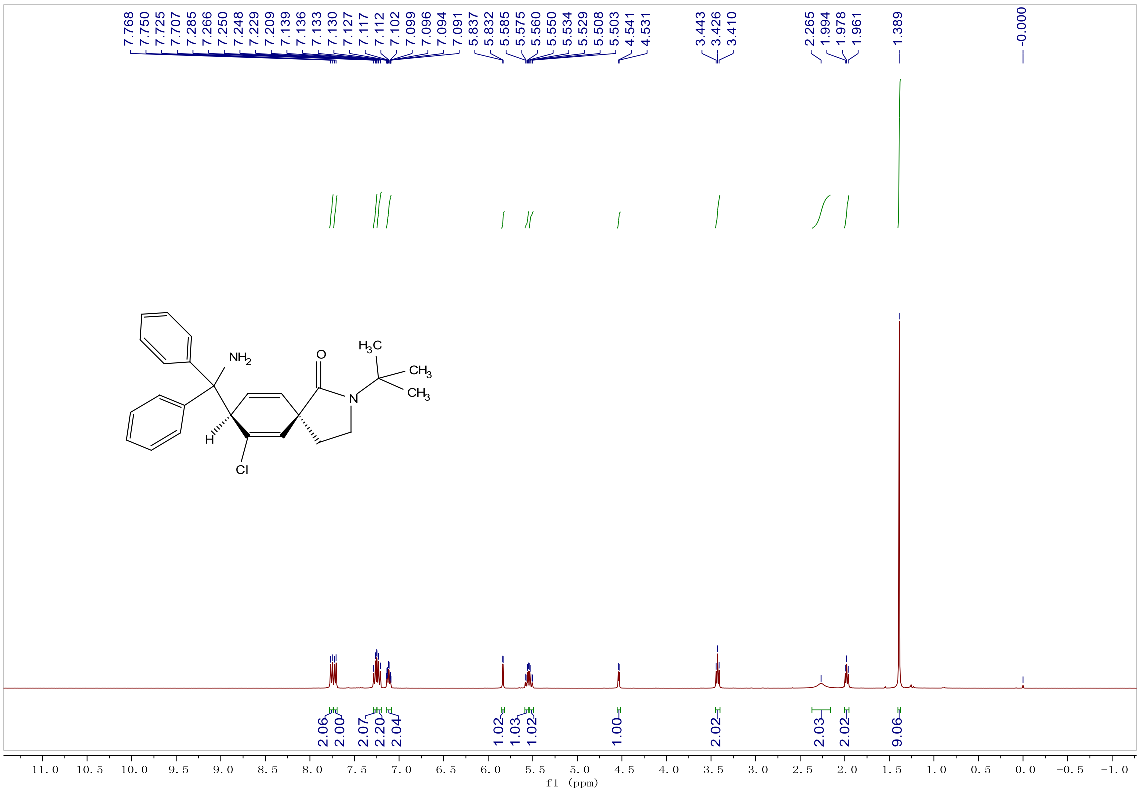

^{1}H-NMR for cis-**3-8**

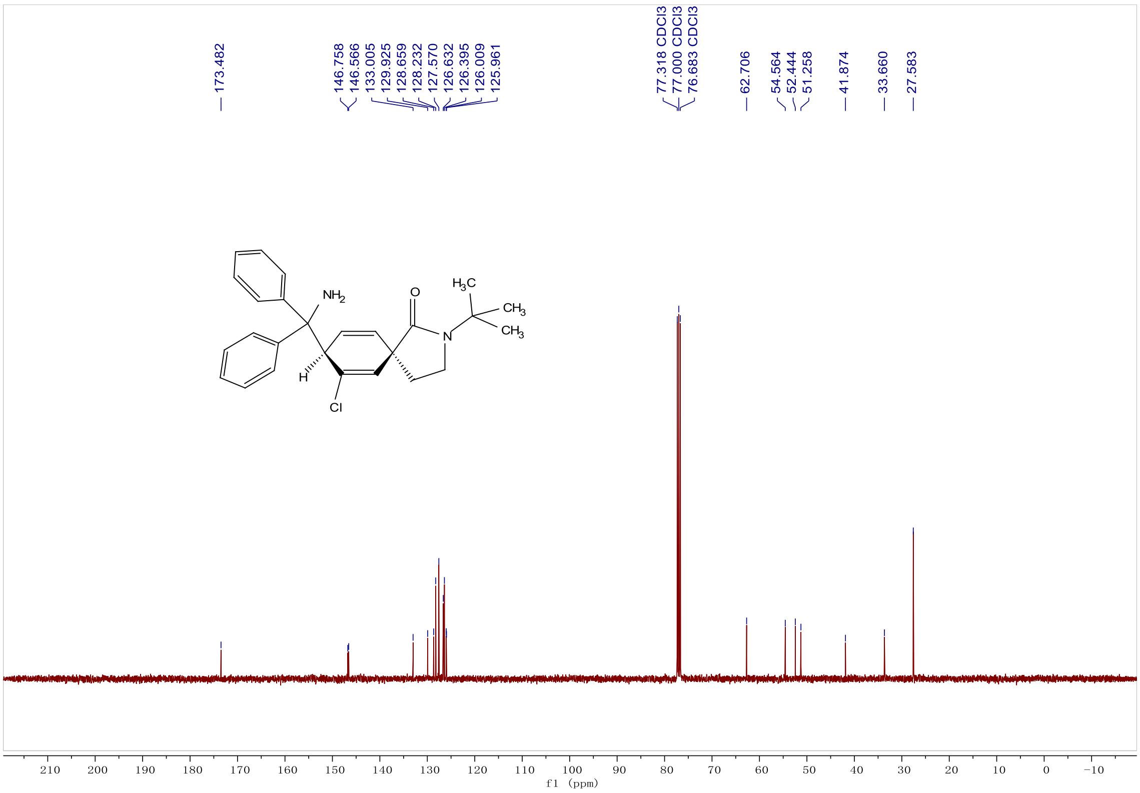

^{13}C−NMR for cis-**3-8**

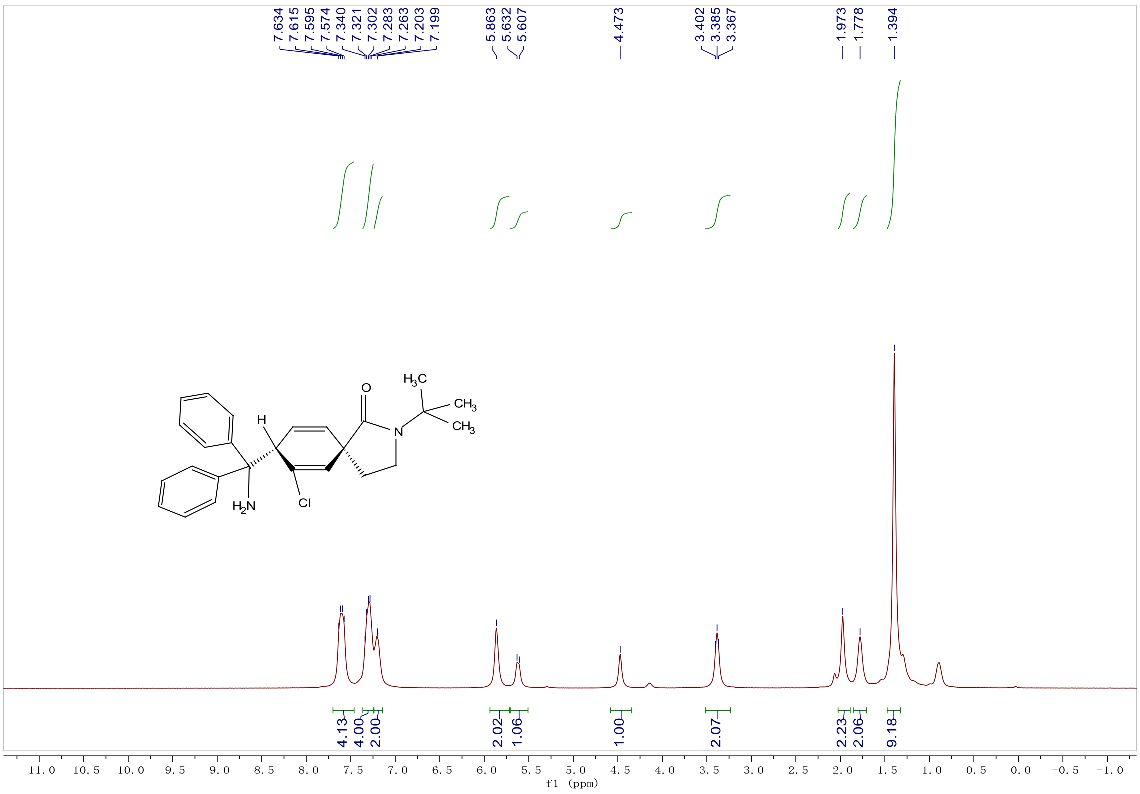

^{1}H-NMR for trans-**3-8**

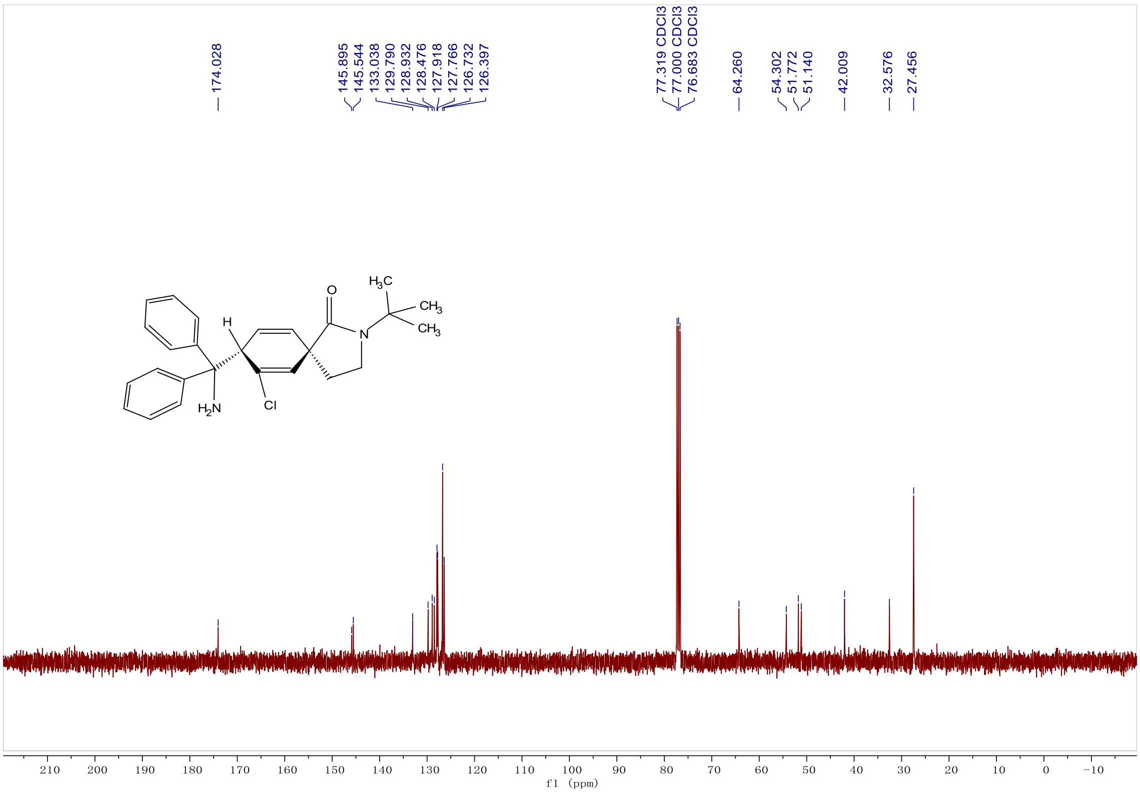

^{13}C−NMR for trans-**3-8**

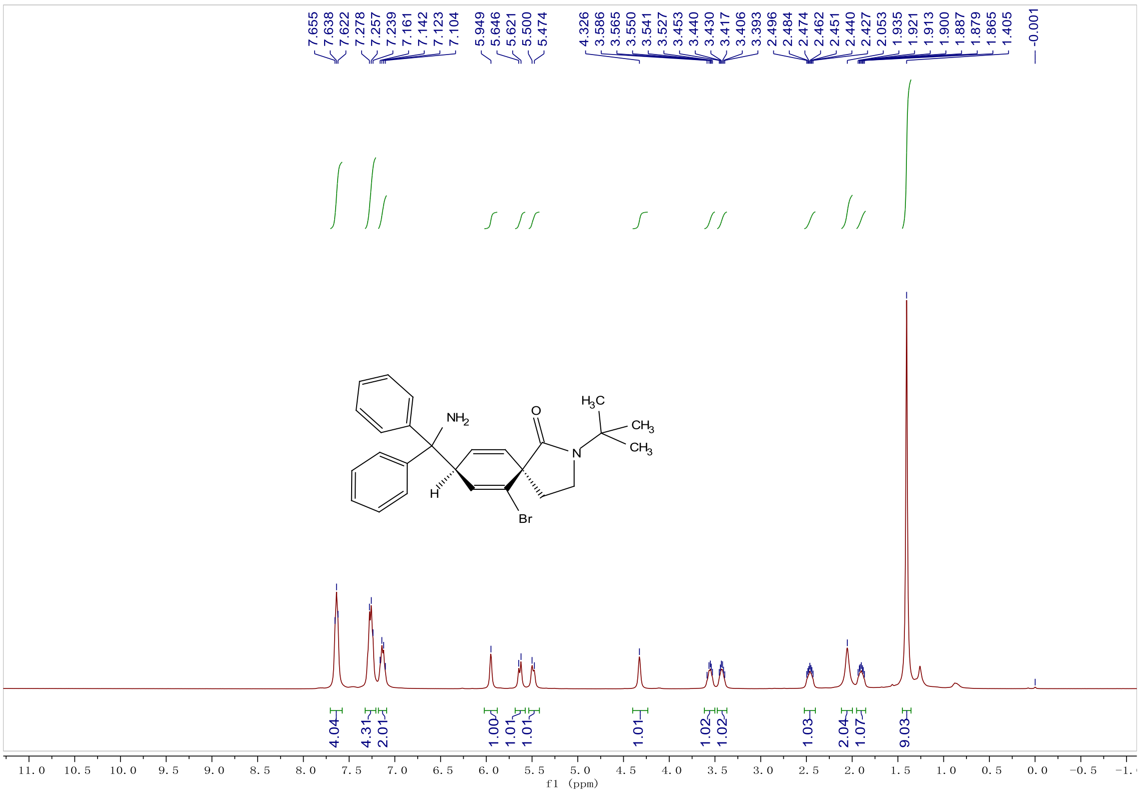

^{1}H-NMR for cis-**3-9**

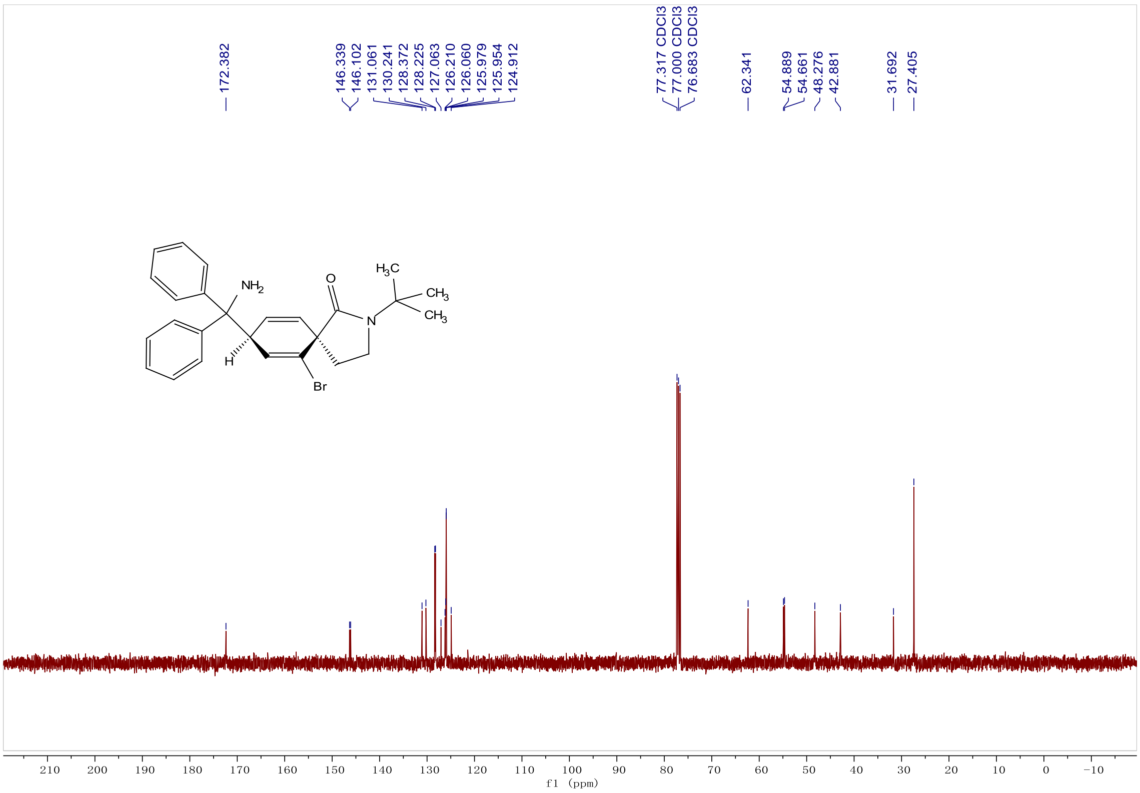

^{13}C−NMR for cis-**3-9**

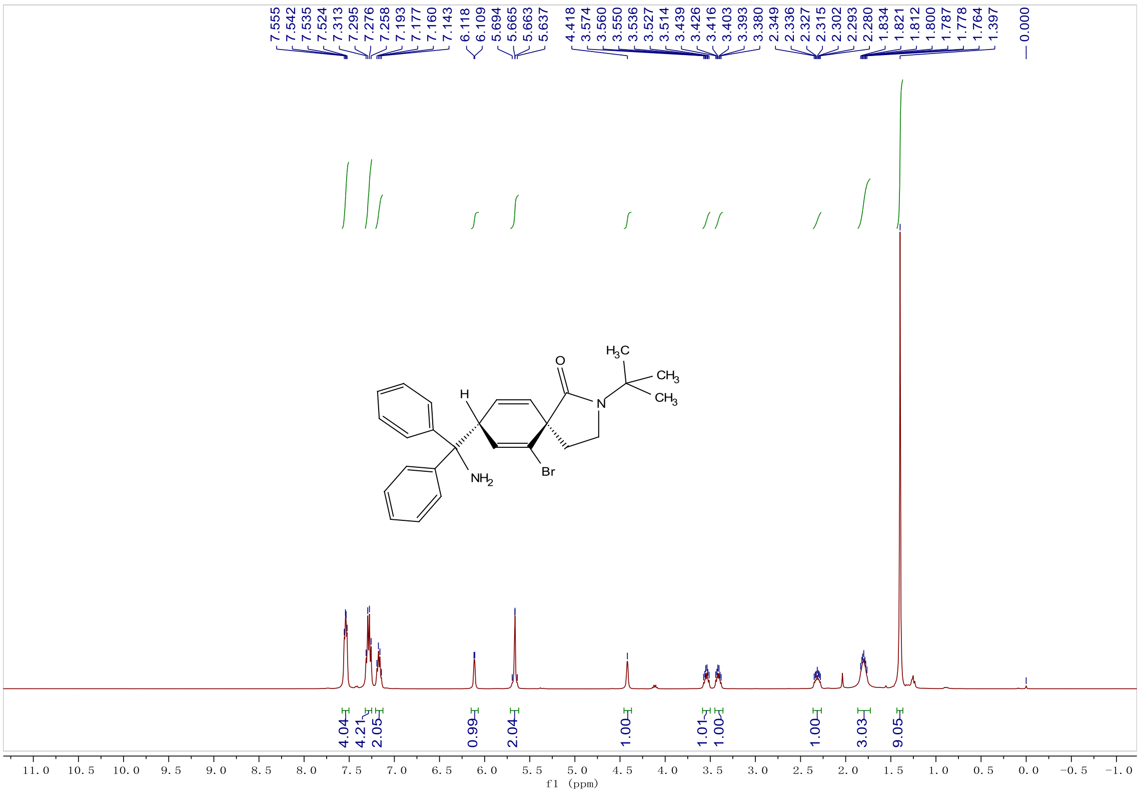

^{1}H-NMR for trans-**3-9**

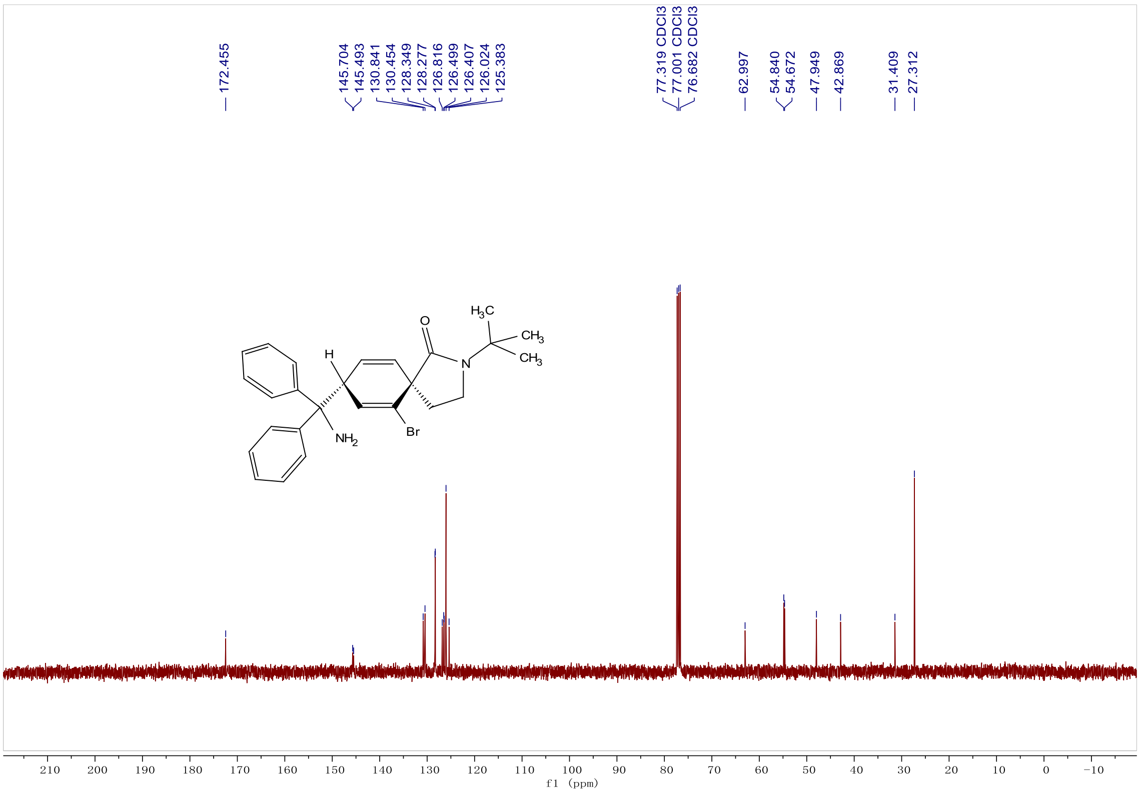

^{13}C−NMR for trans-**3-9**

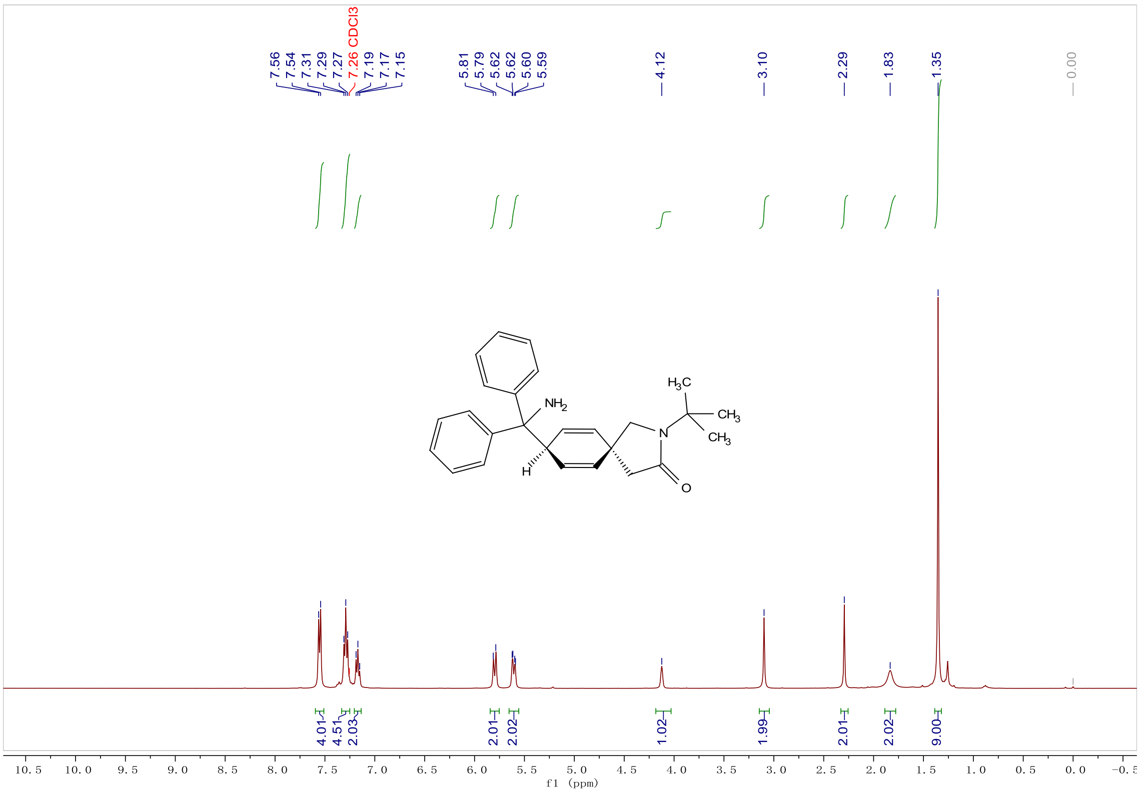

^{1}H-NMR for cis-**3-10**

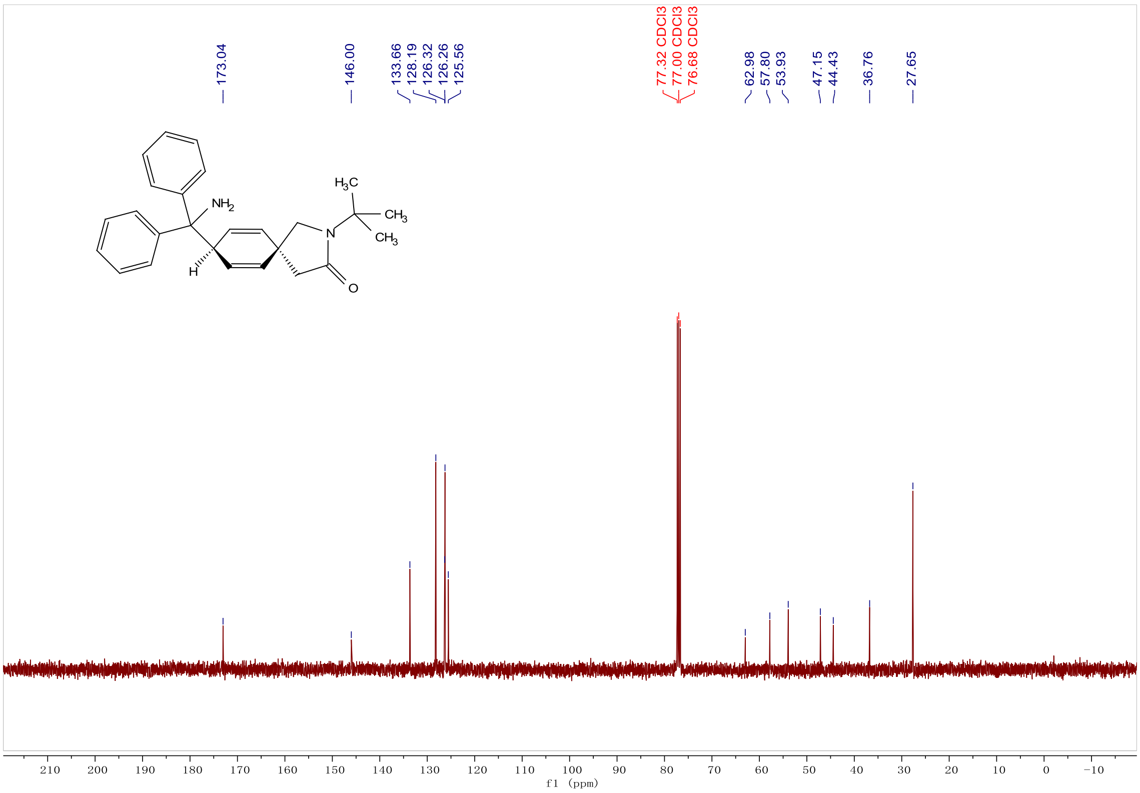

^{13}C−NMR for cis-**3-10**

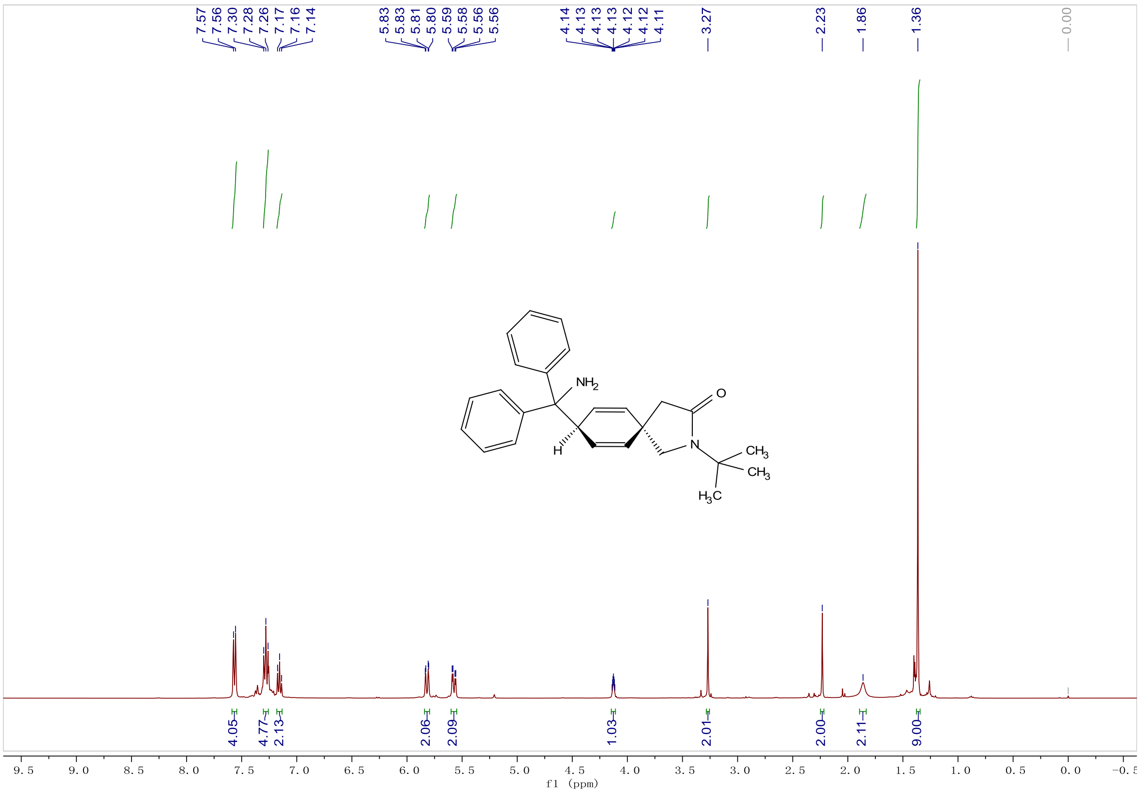

^{1}H-NMR for trans-**3-10**

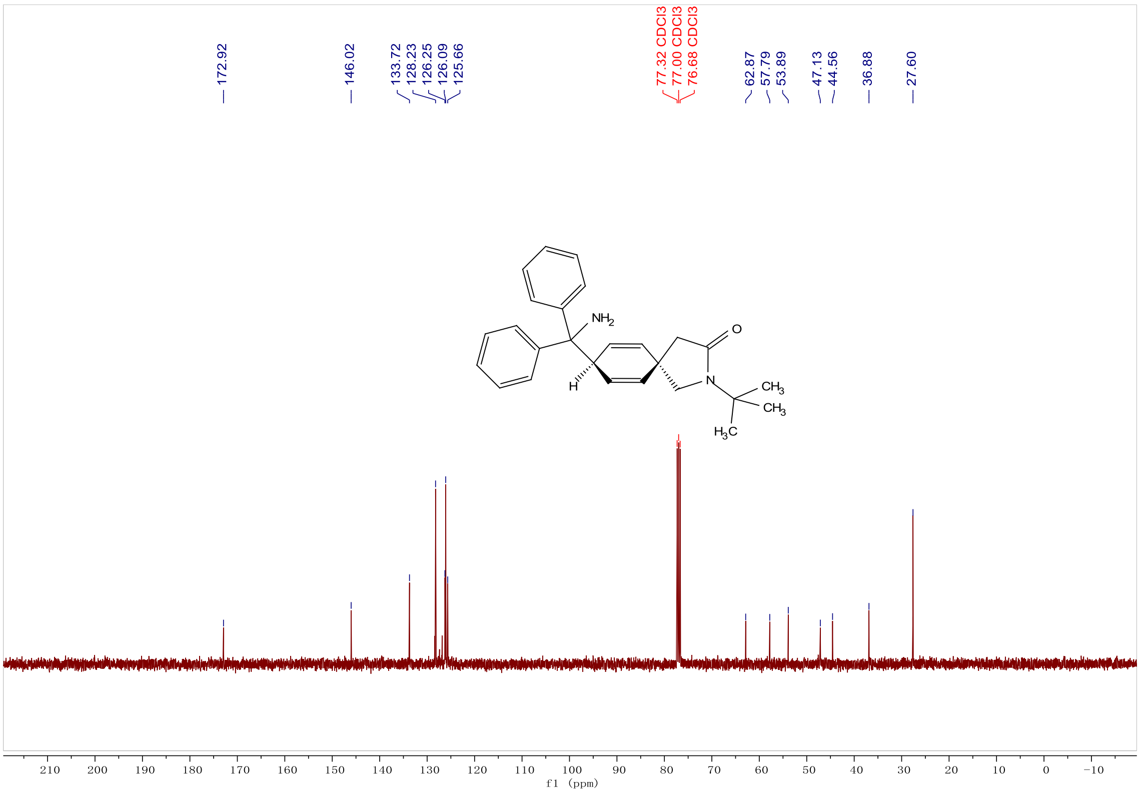

^{13}C−NMR for trans-**3-10**

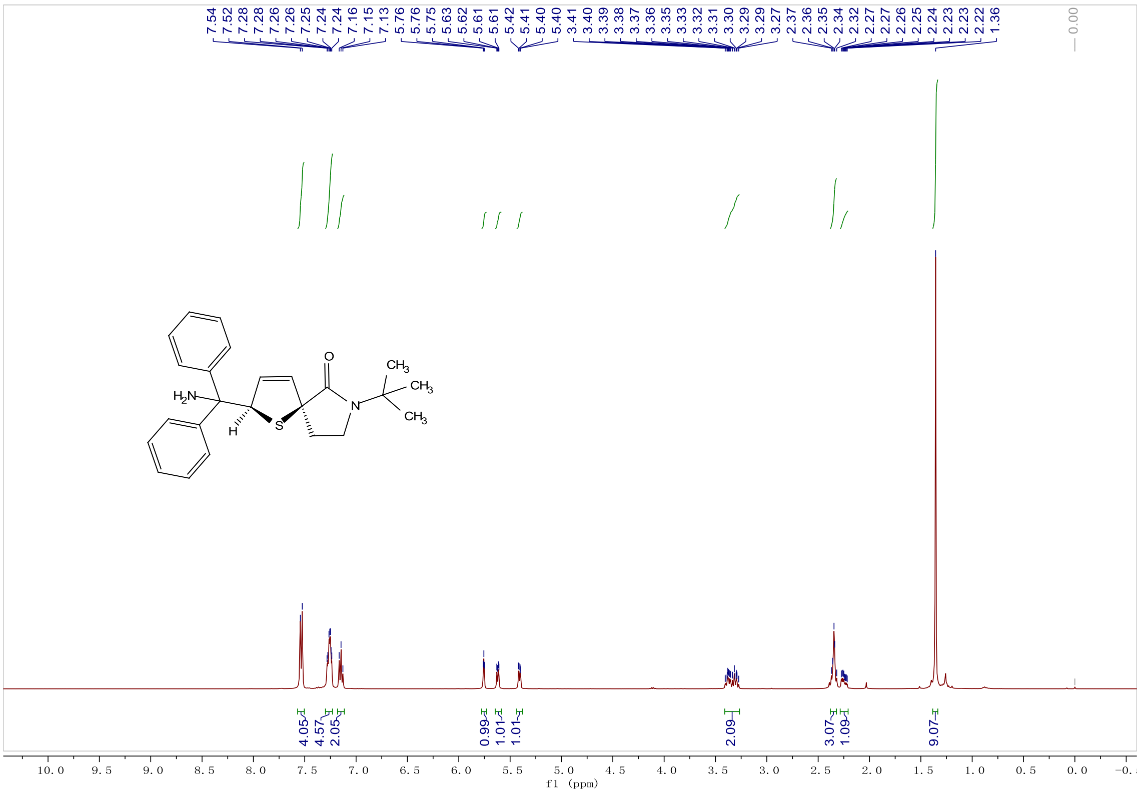

^{1}H-NMR for cis-**3-11**

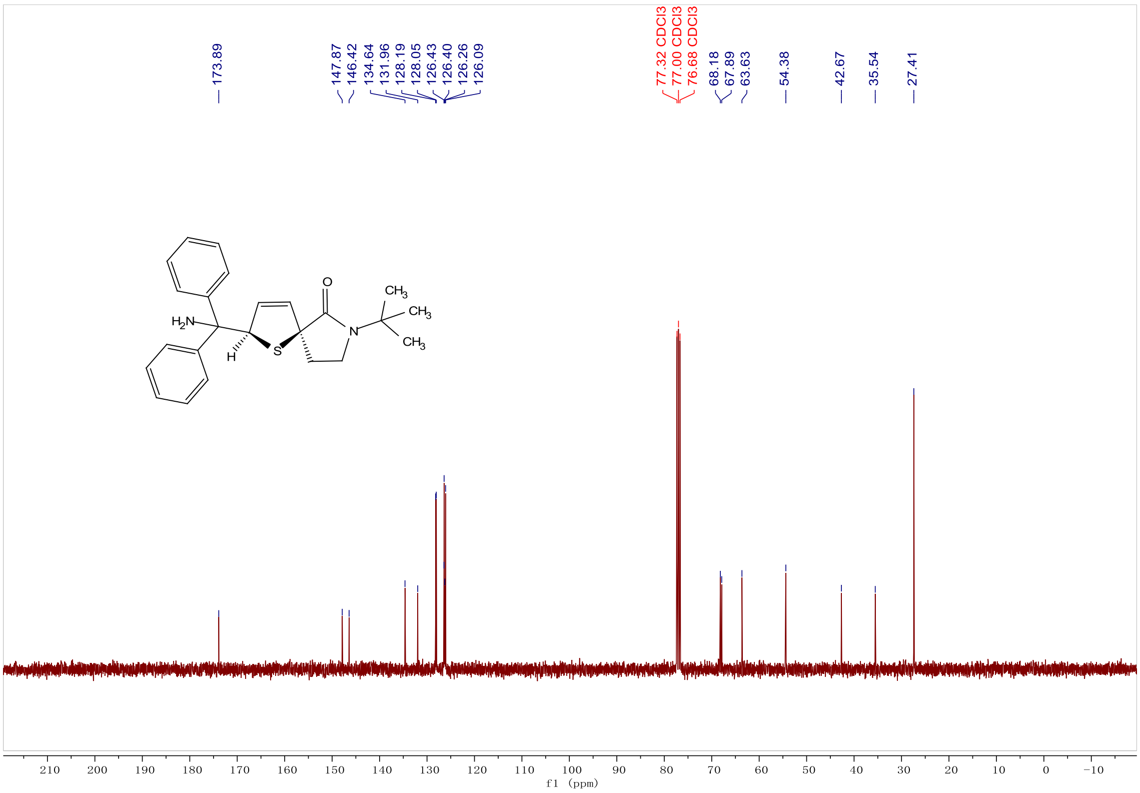

^{13}C−NMR for cis-**3-11**

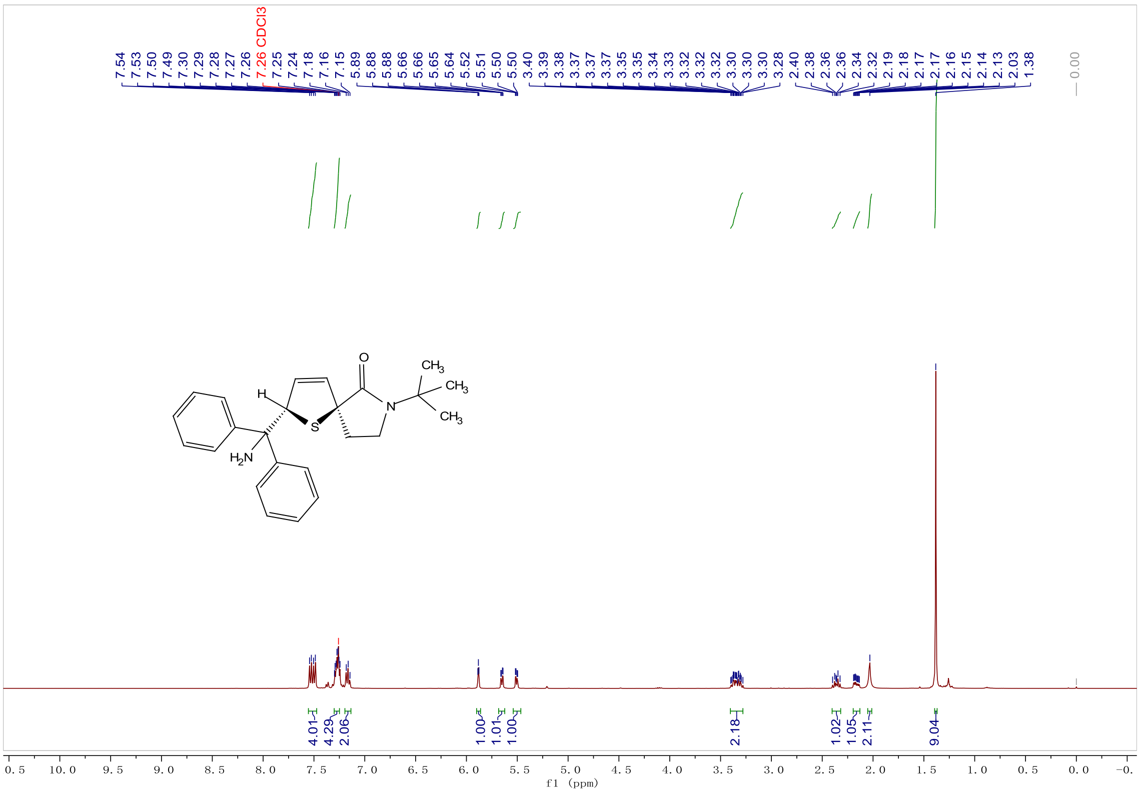

^{1}H-NMR for trans-**3-11**

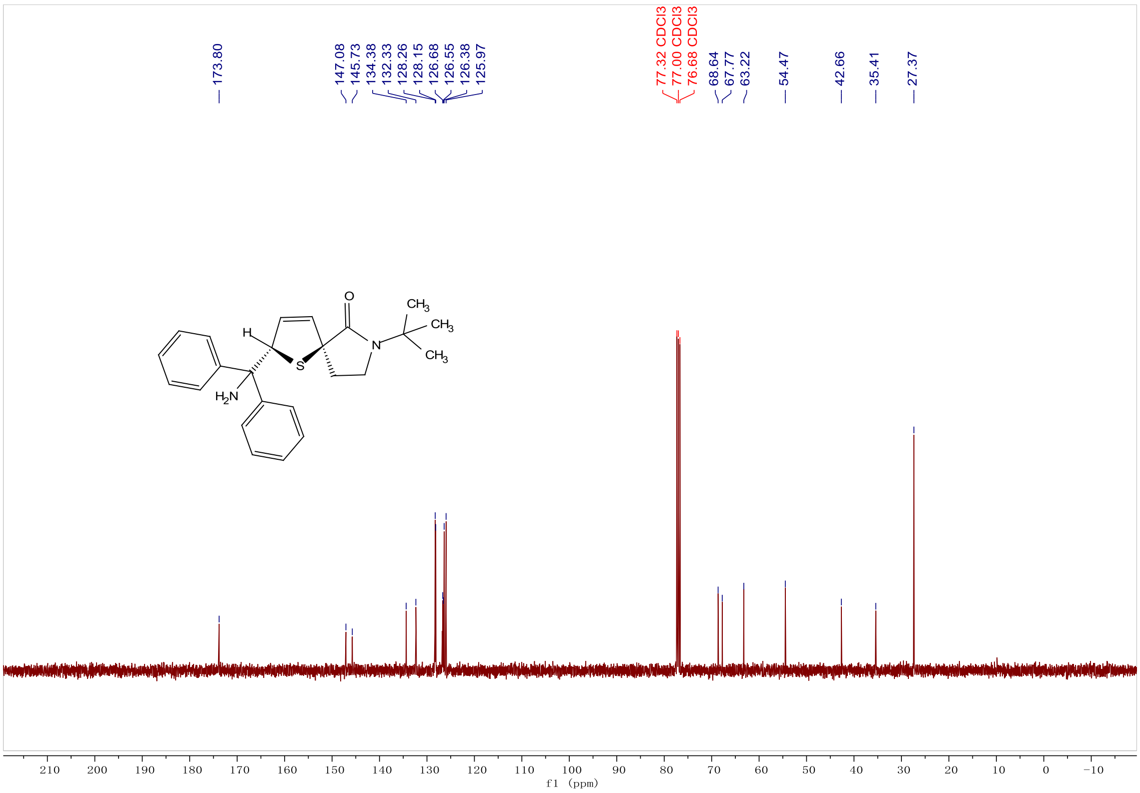

^{13}C−NMR for trans-**3-11**

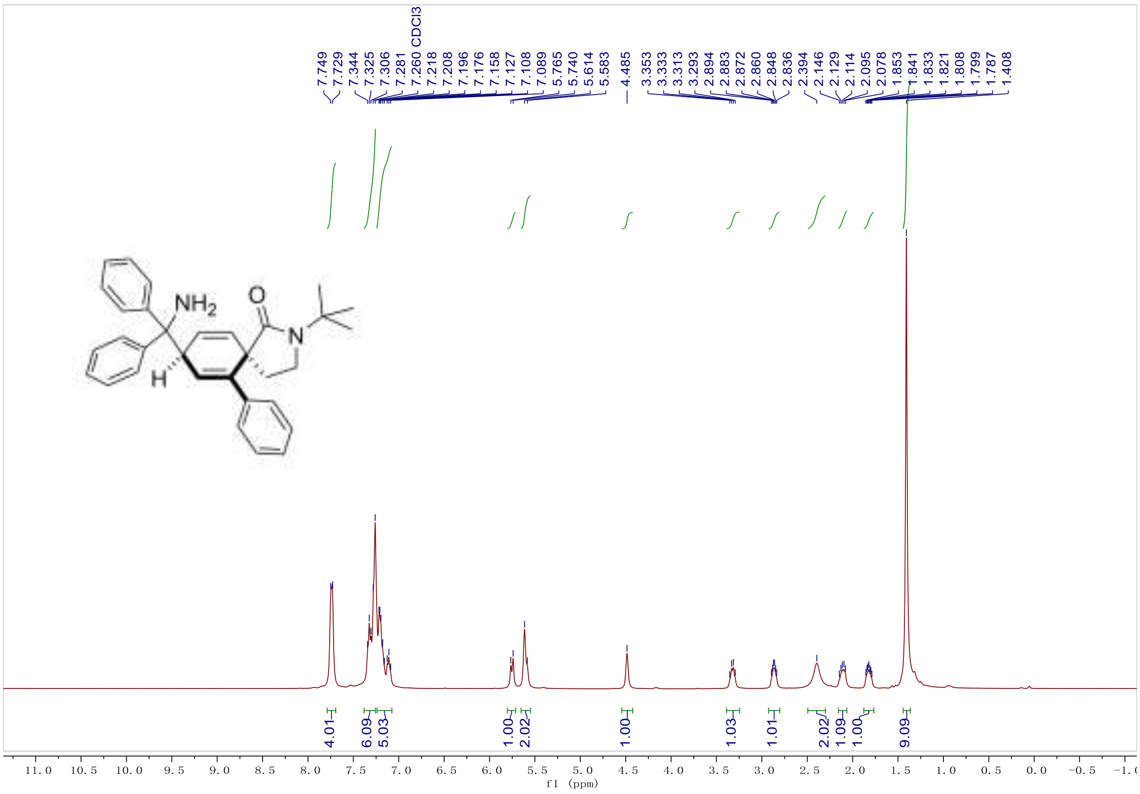

^{1}H-NMR for cis-**3-12**

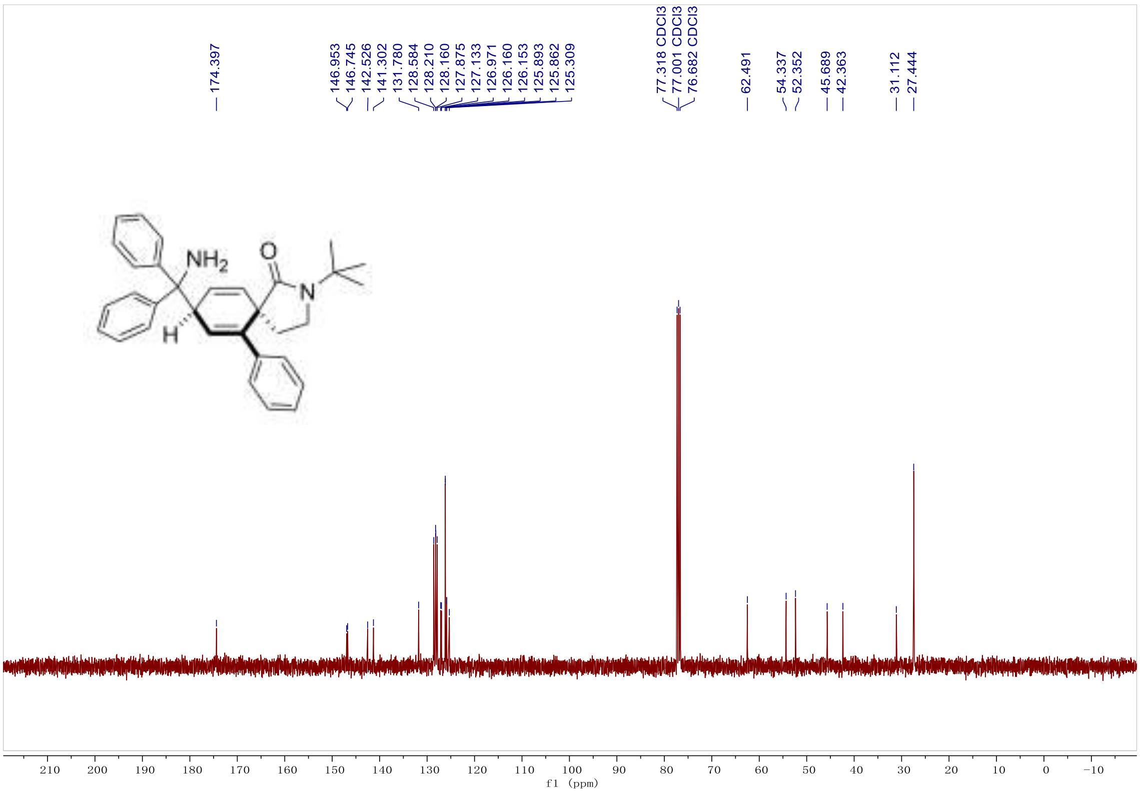

$^{13}C-NMR$ for cis-**3-12**

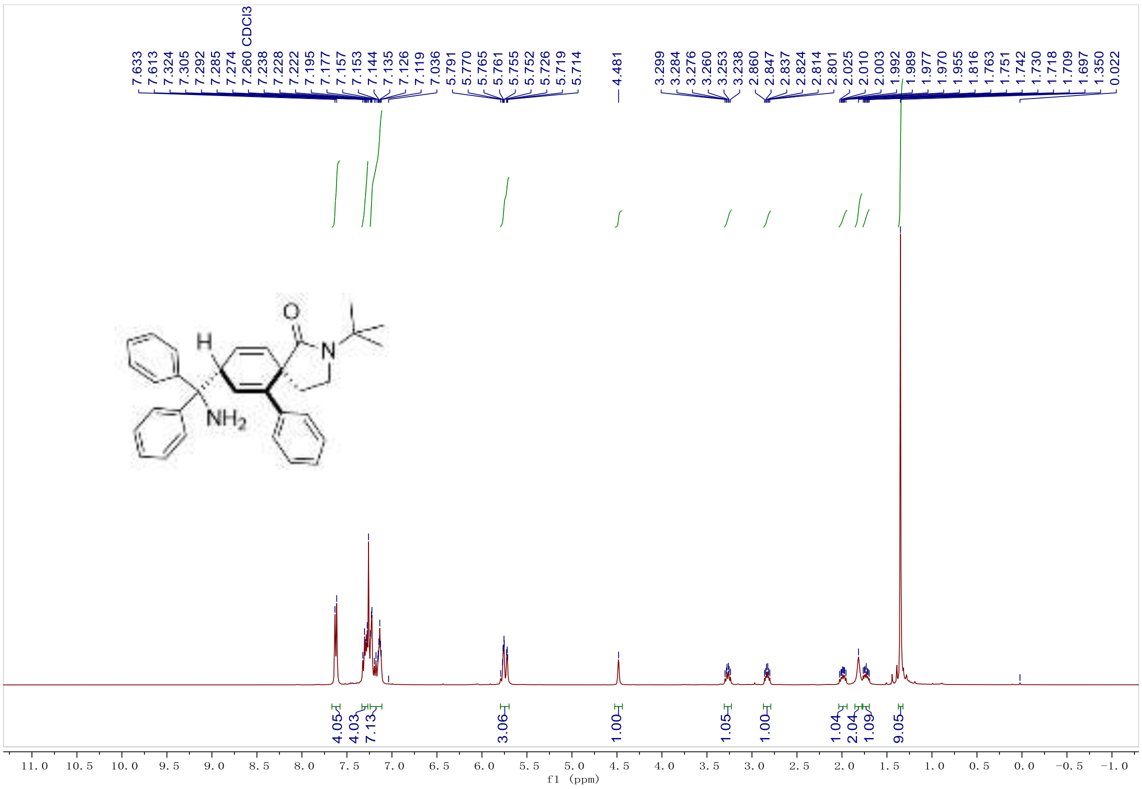

$^{1}H-NMR$ for trans-**3-12**

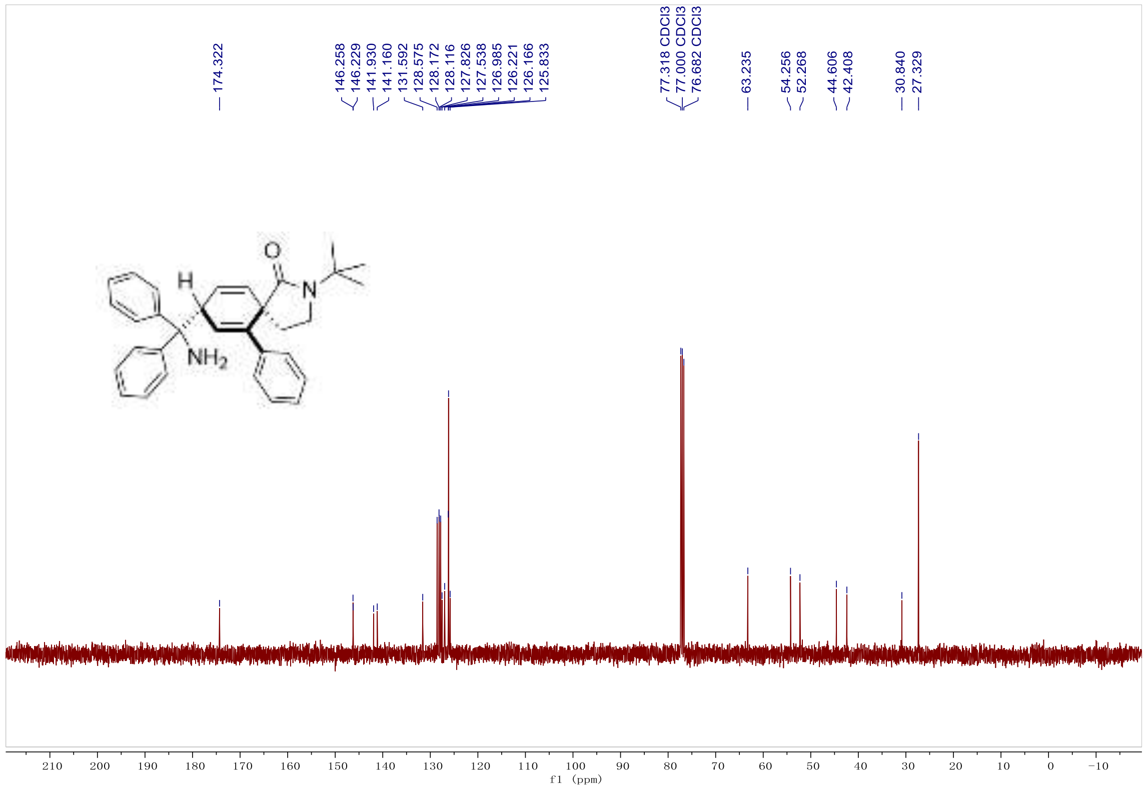

$^{13}C-NMR$ for trans-**3-12**

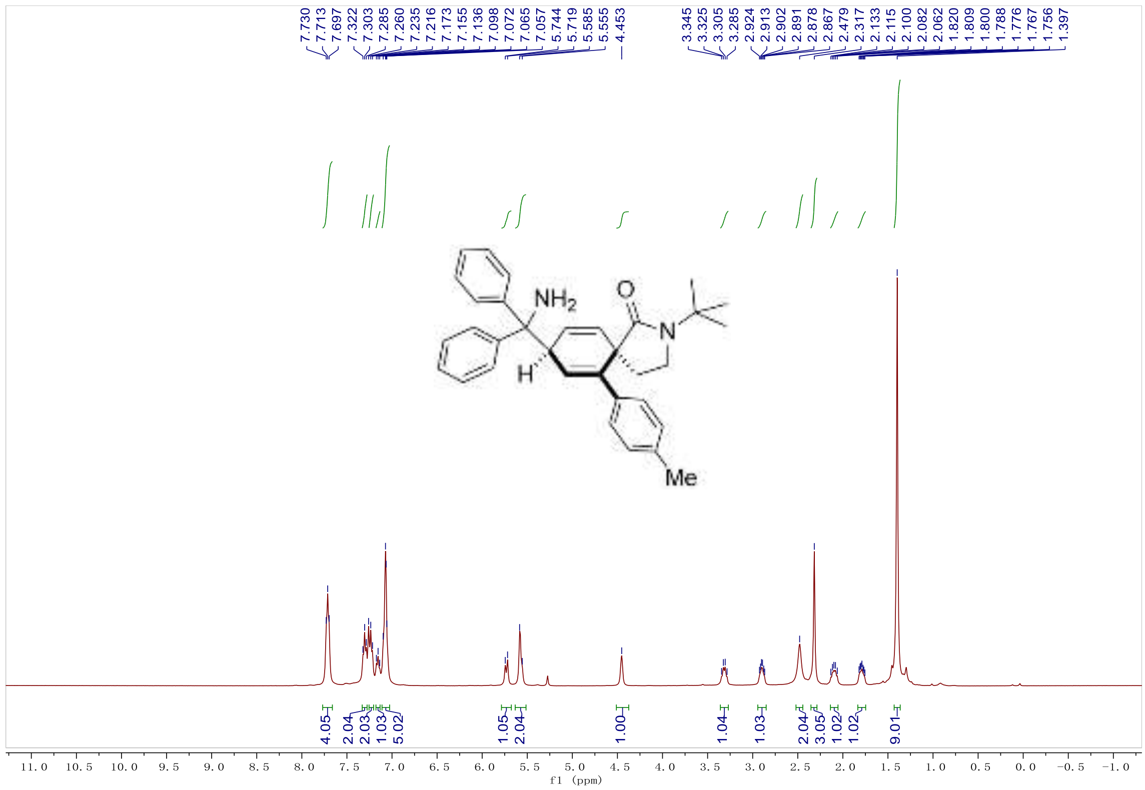

$^{1}H-NMR$ for cis-**3-13**

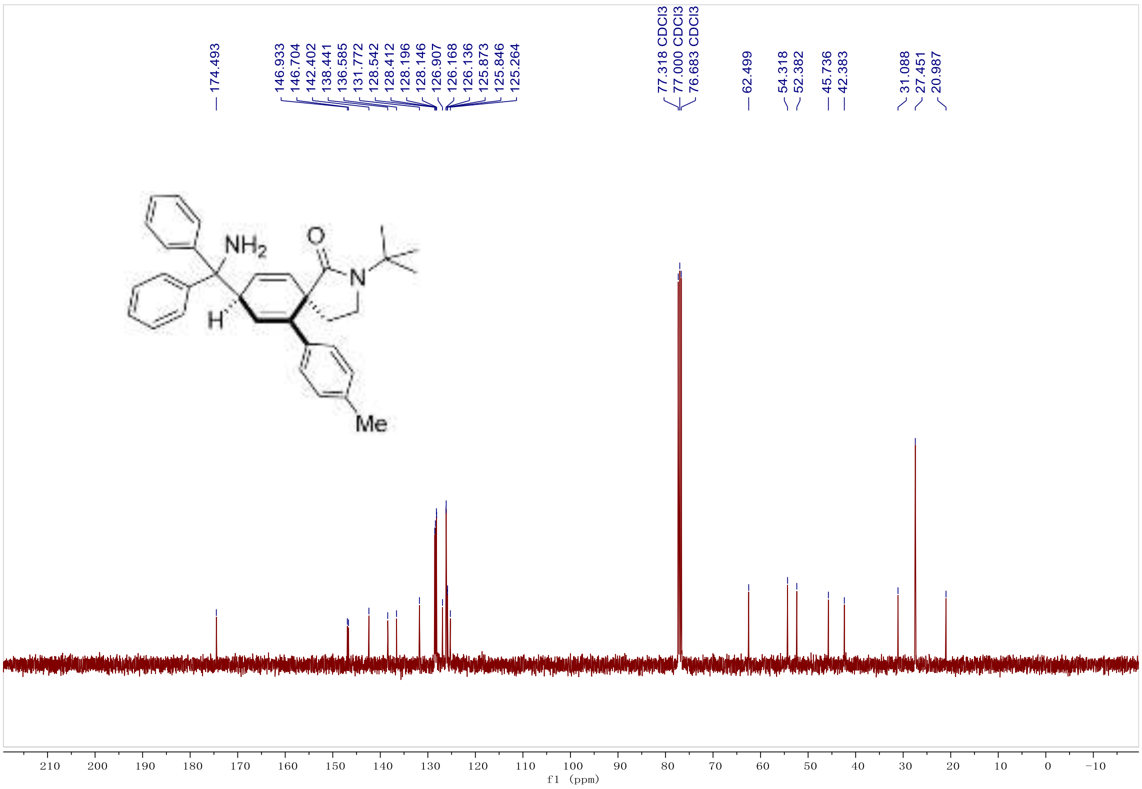

^{13}C−NMR for cis-**3-13**

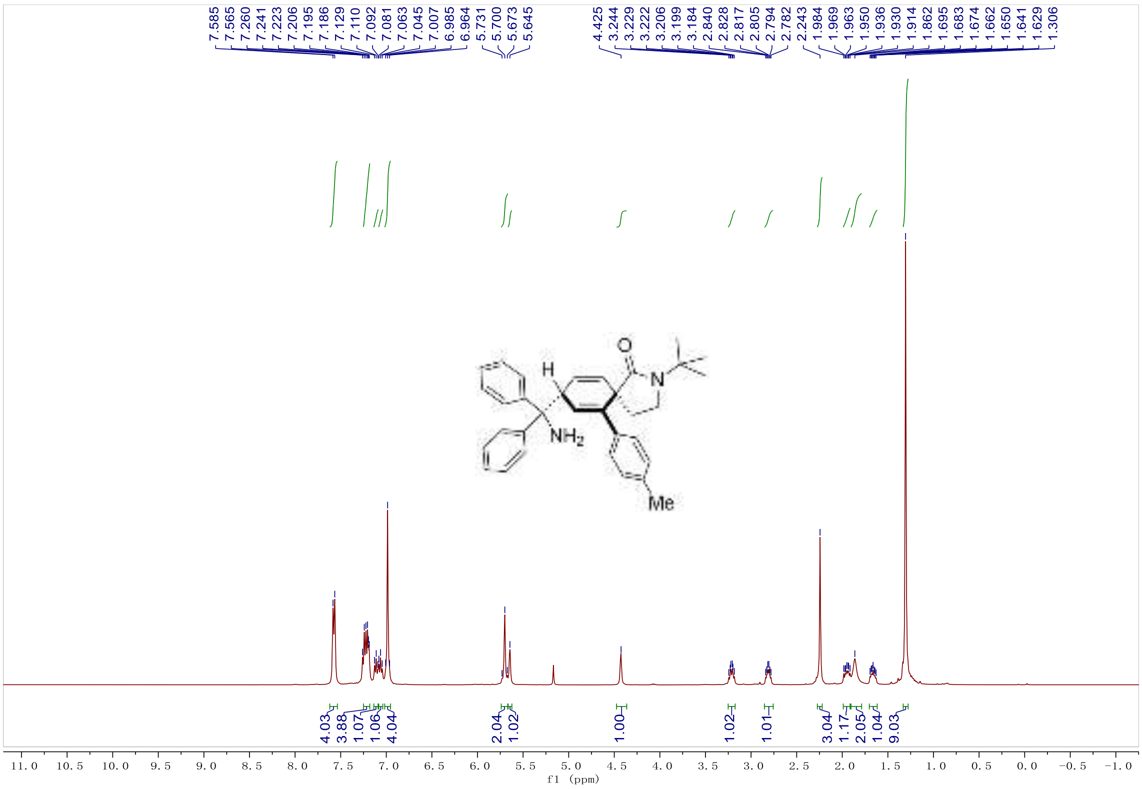

^{1}H-NMR for trans-**3-13**

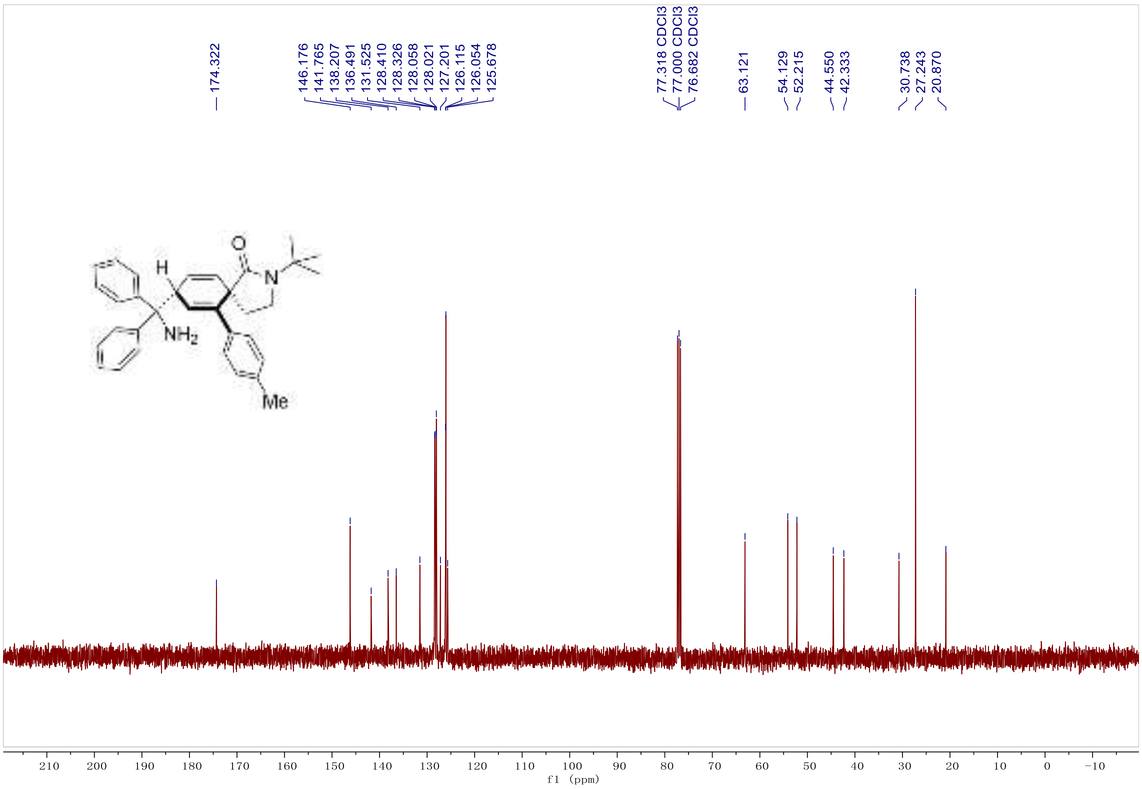

^{13}C−NMR for trans-**3-13**

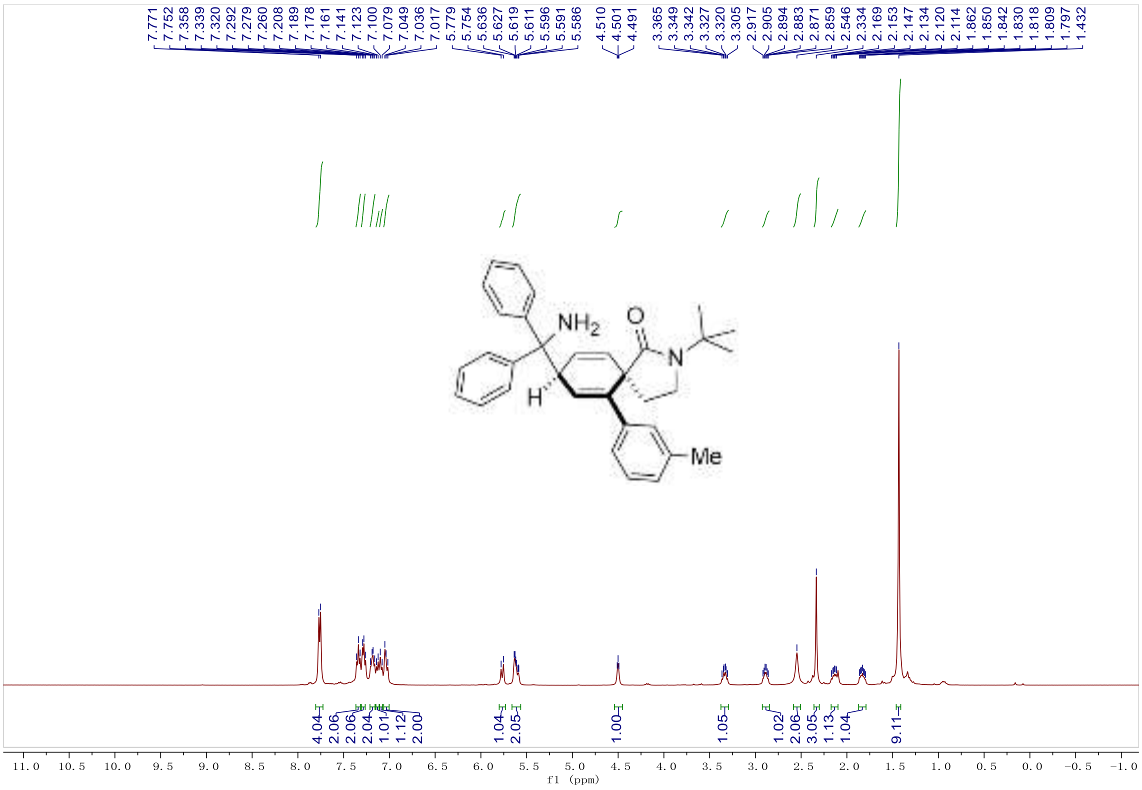

^{1}H-NMR for cis-**3-14**

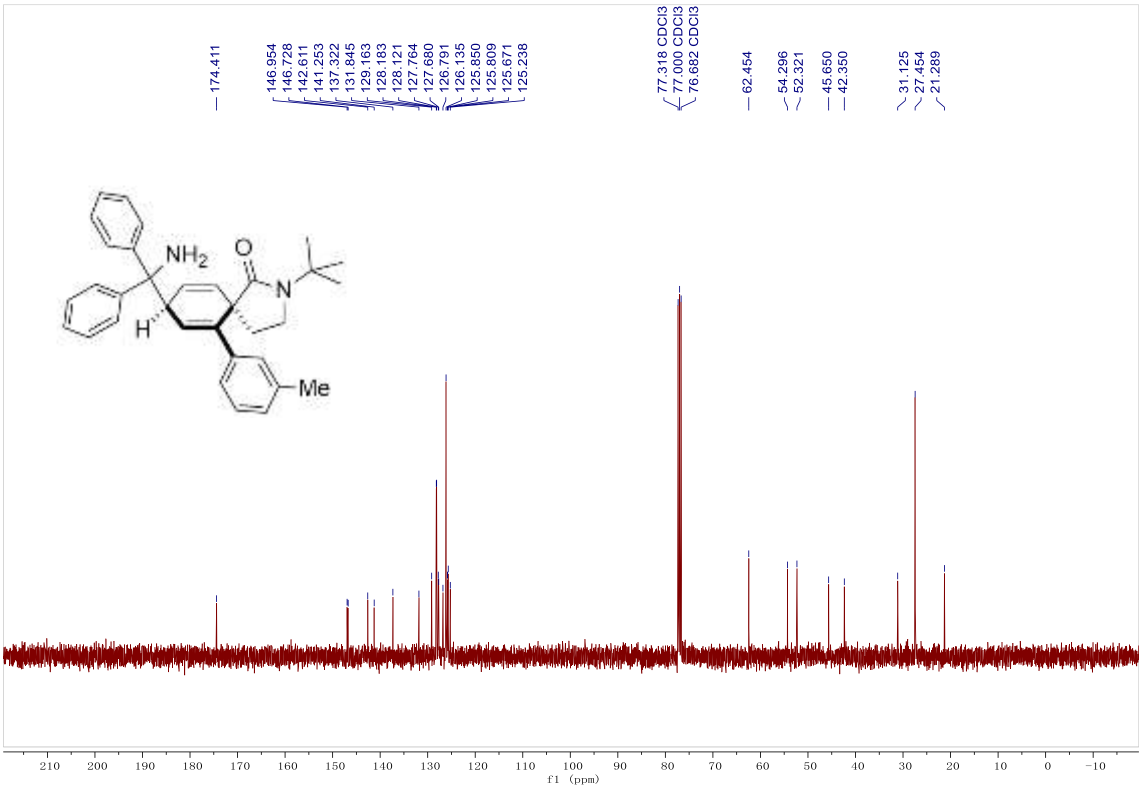

^{13}C−NMR for cis-**3-14**

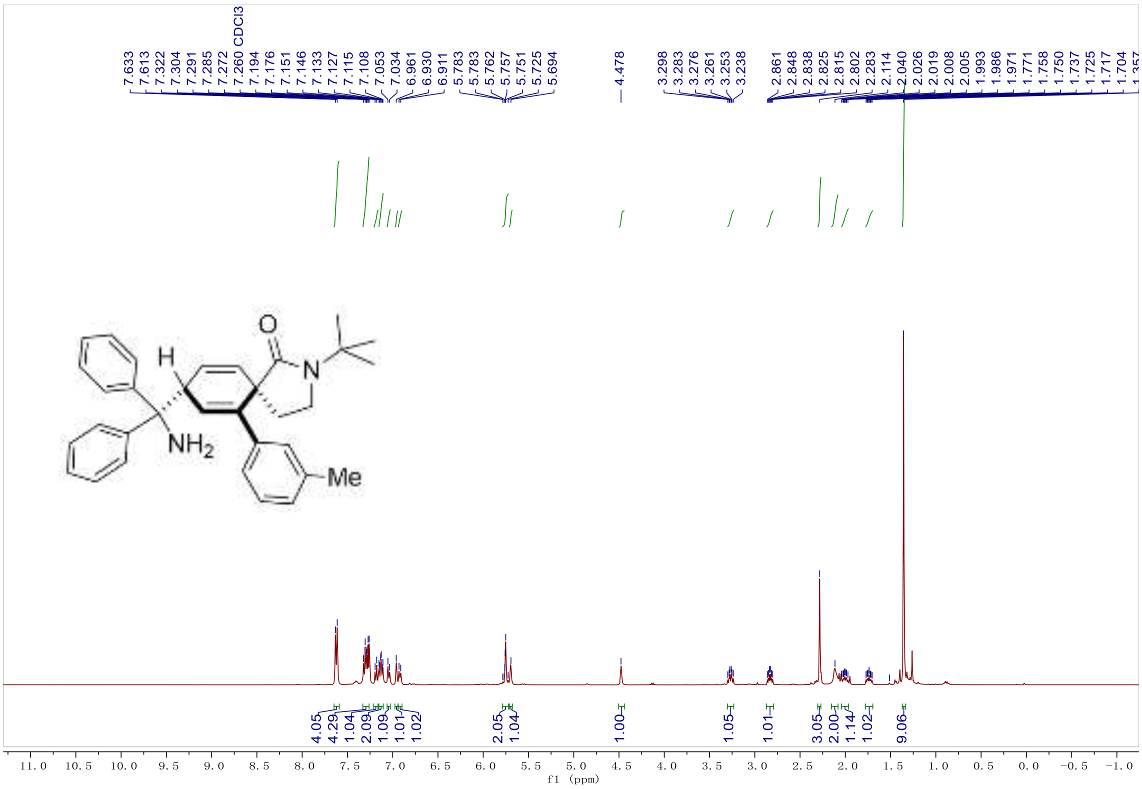

^{1}H-NMR for trans-**3-14**

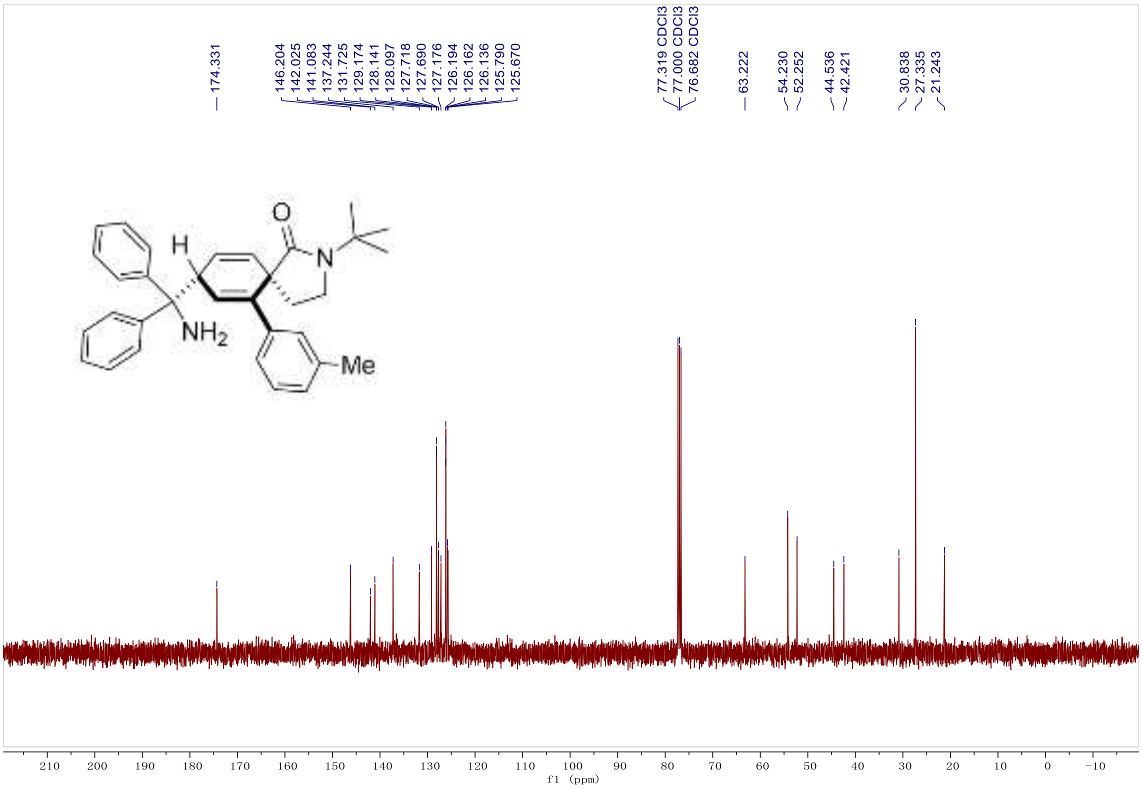

$^{13}C-NMR$ for trans-**3-14**

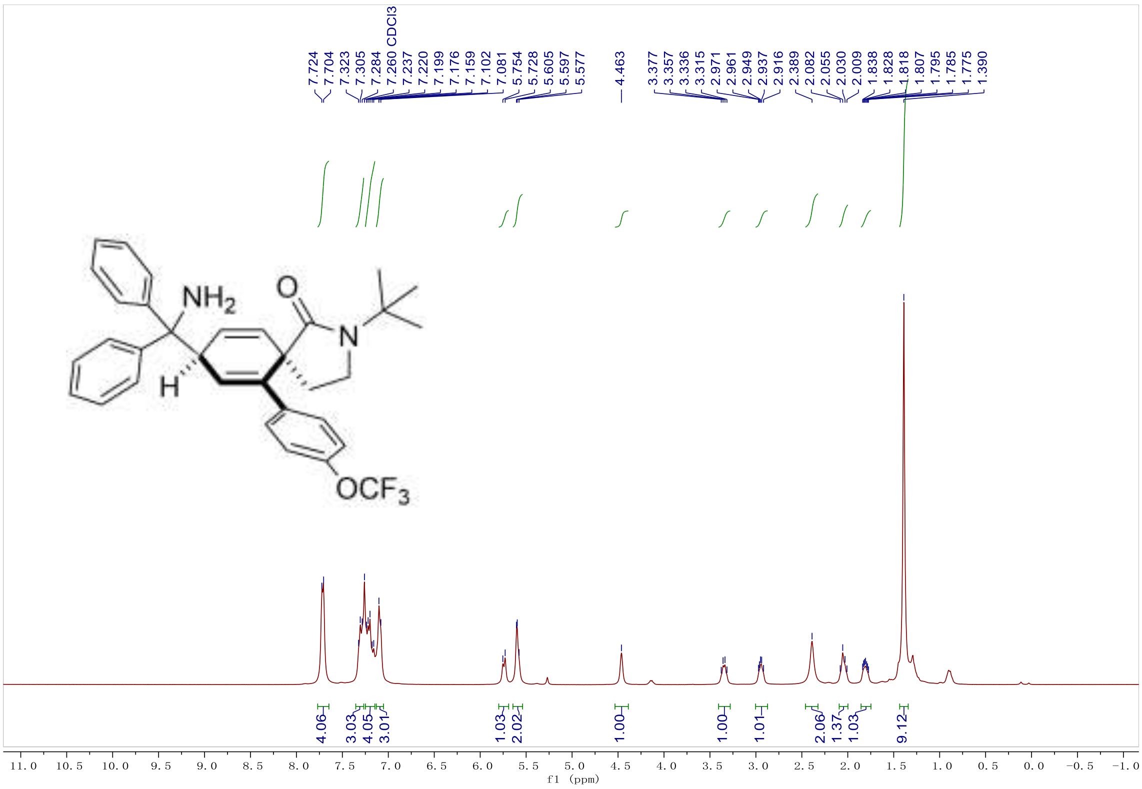

$^{1}H-NMR$ for cis-**3-15**

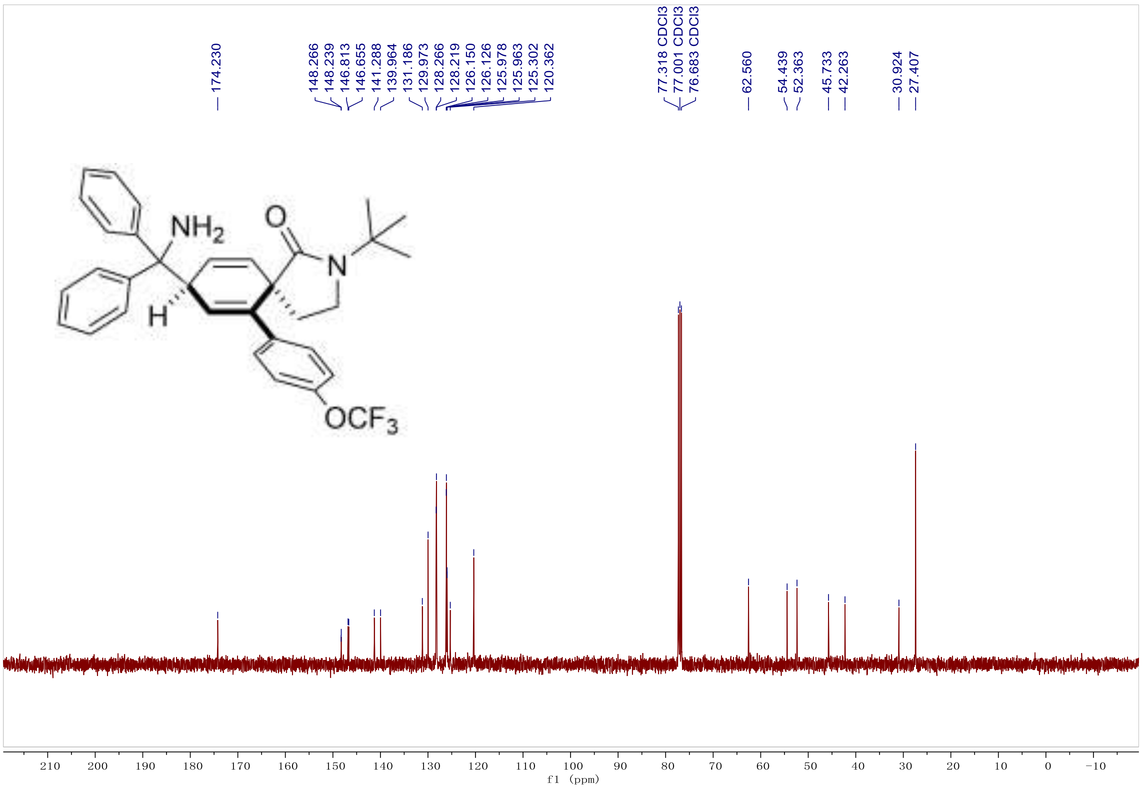

^{13}C−NMR for cis-**3-15**

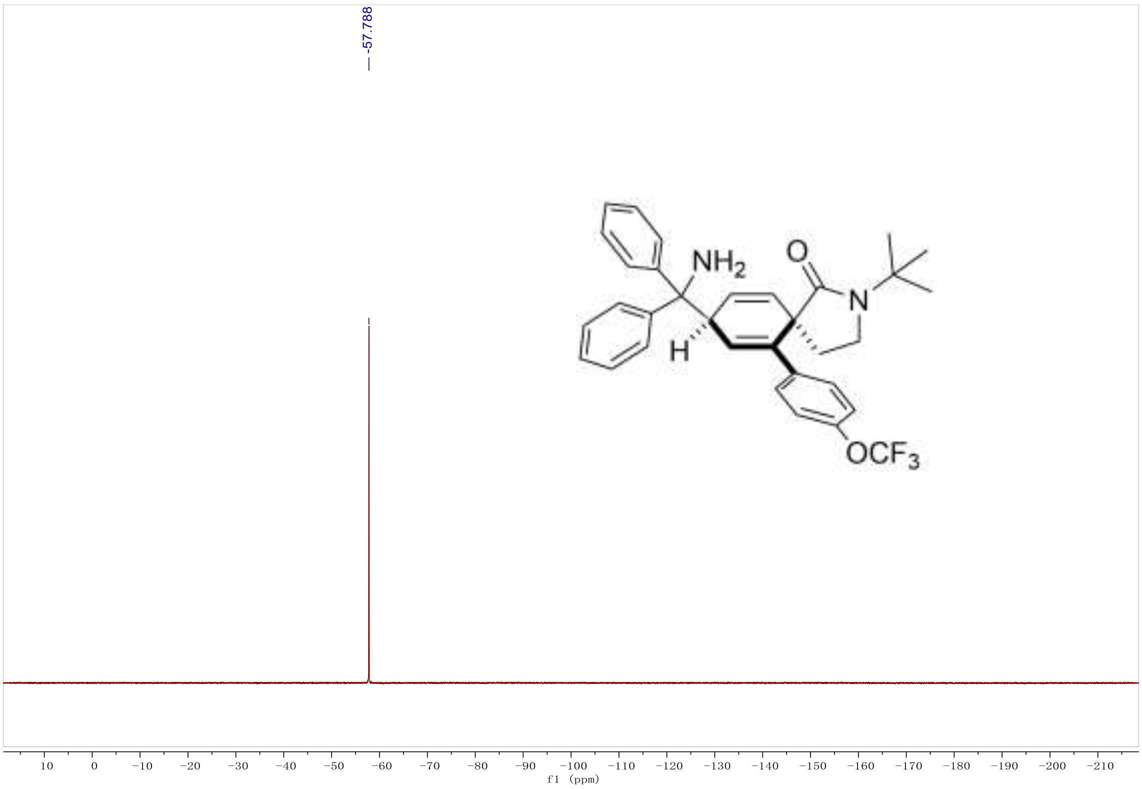

^{19}F-NMR for cis-**3-15**

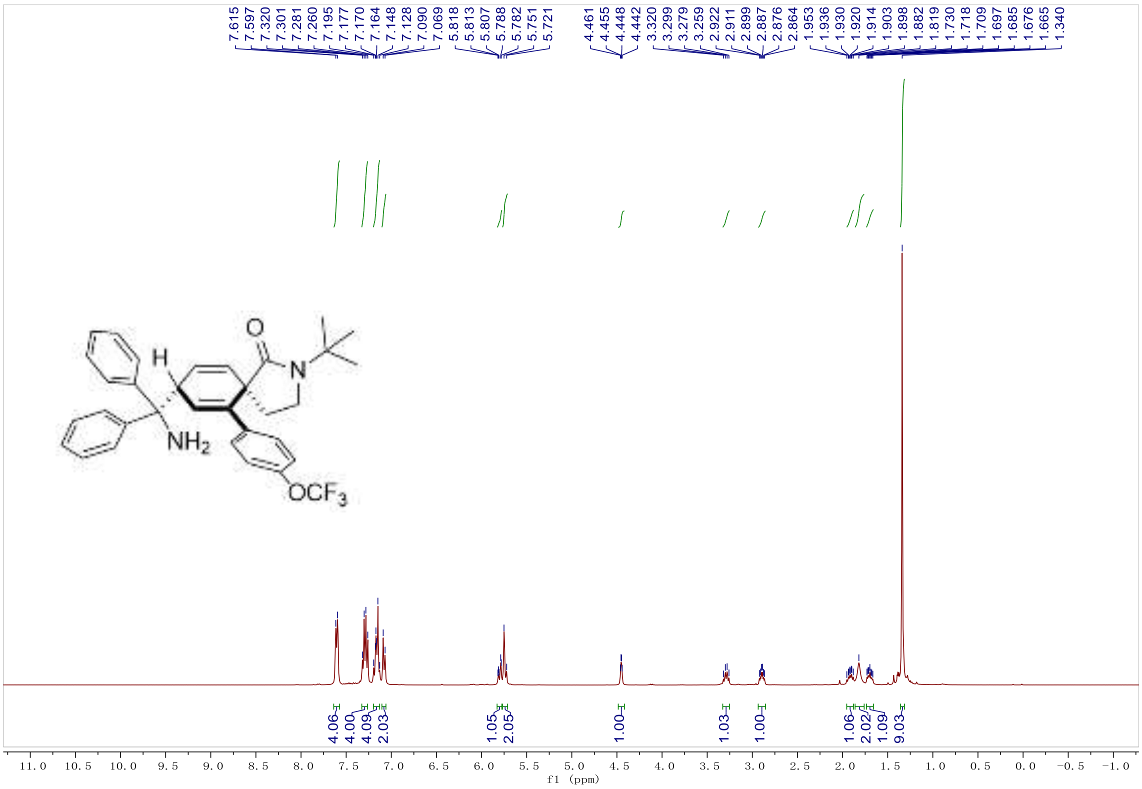

^{1}H-NMR for trans-**3-15**

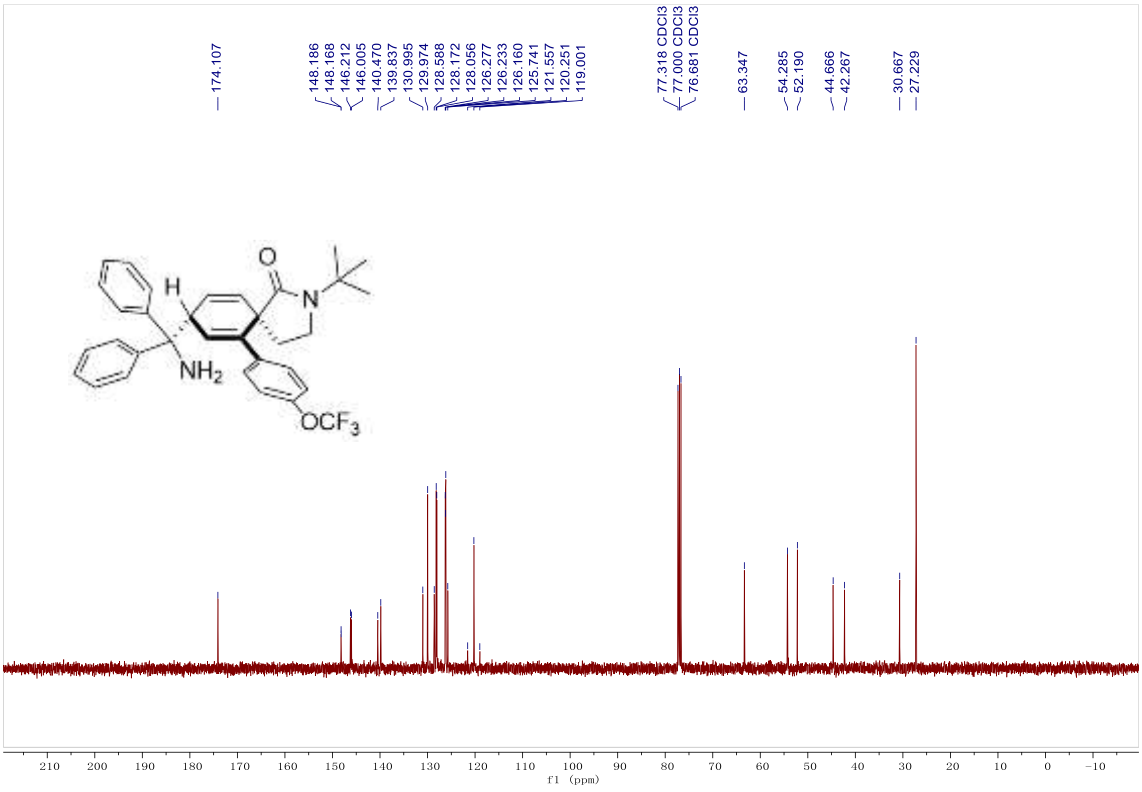

^{13}C−NMR for trans-**3-15**

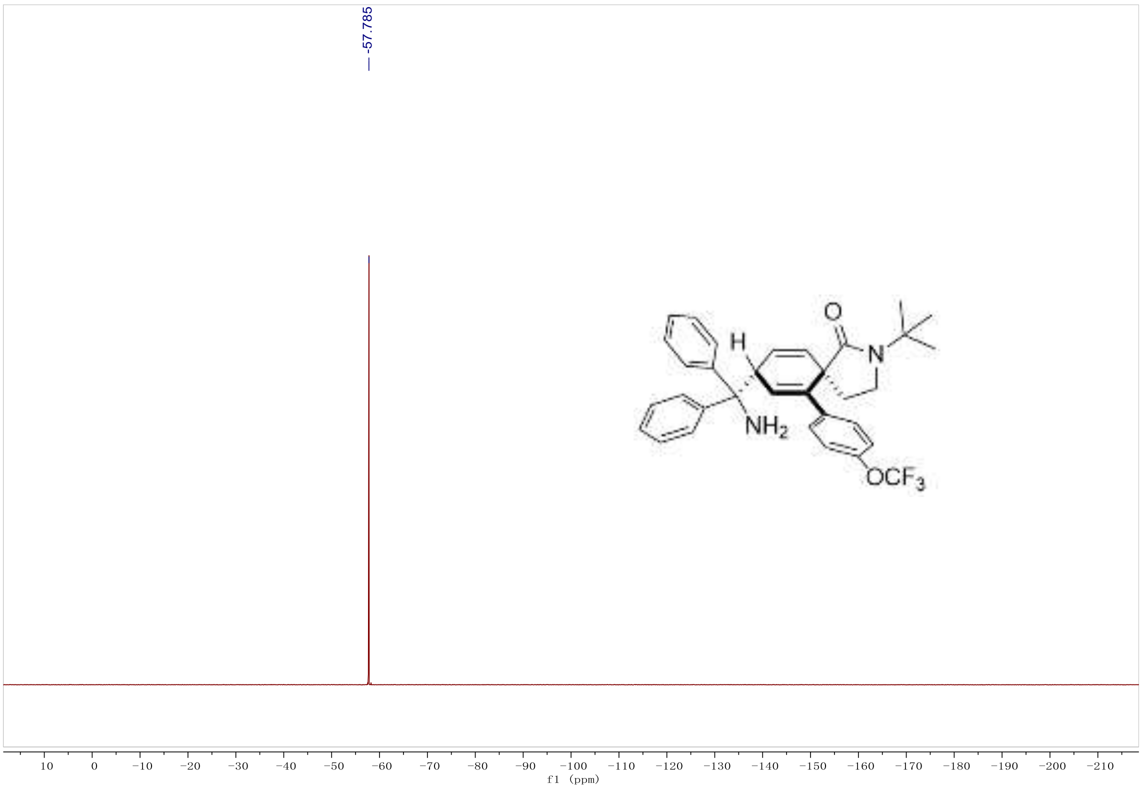

^{19}F-NMR for trans-**3-15**

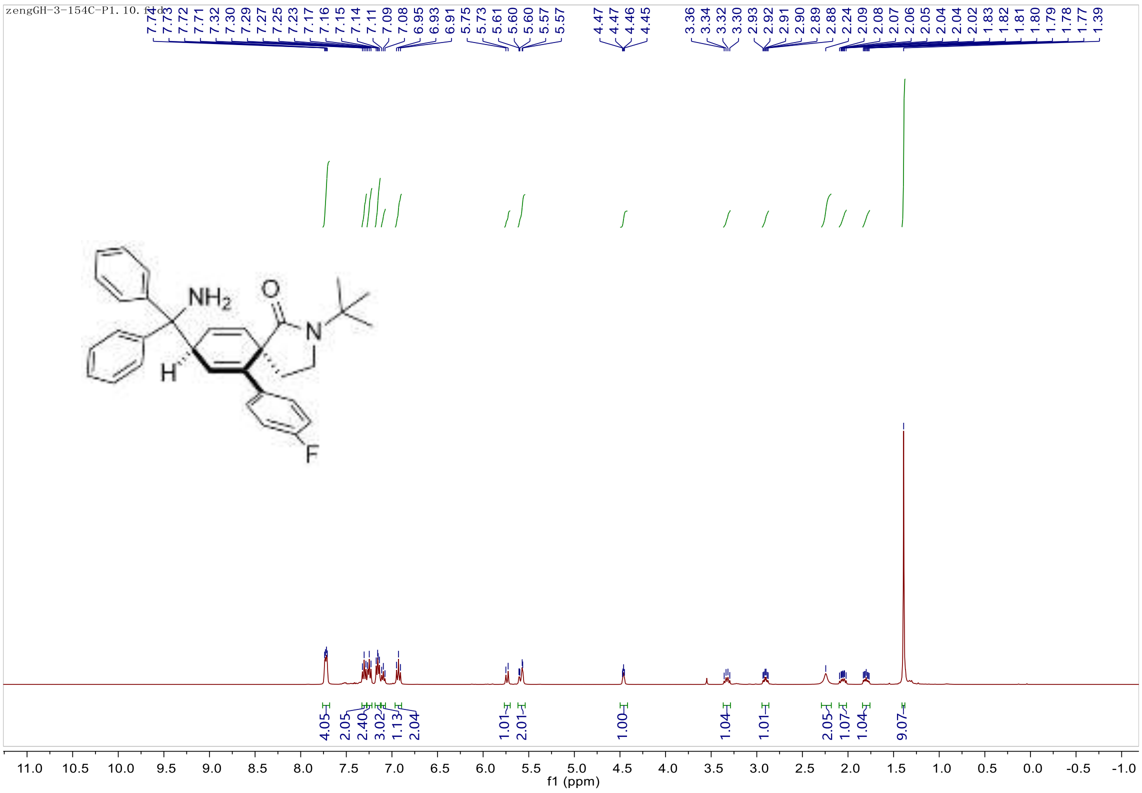

^{1}H-NMR for cis-**3-16**

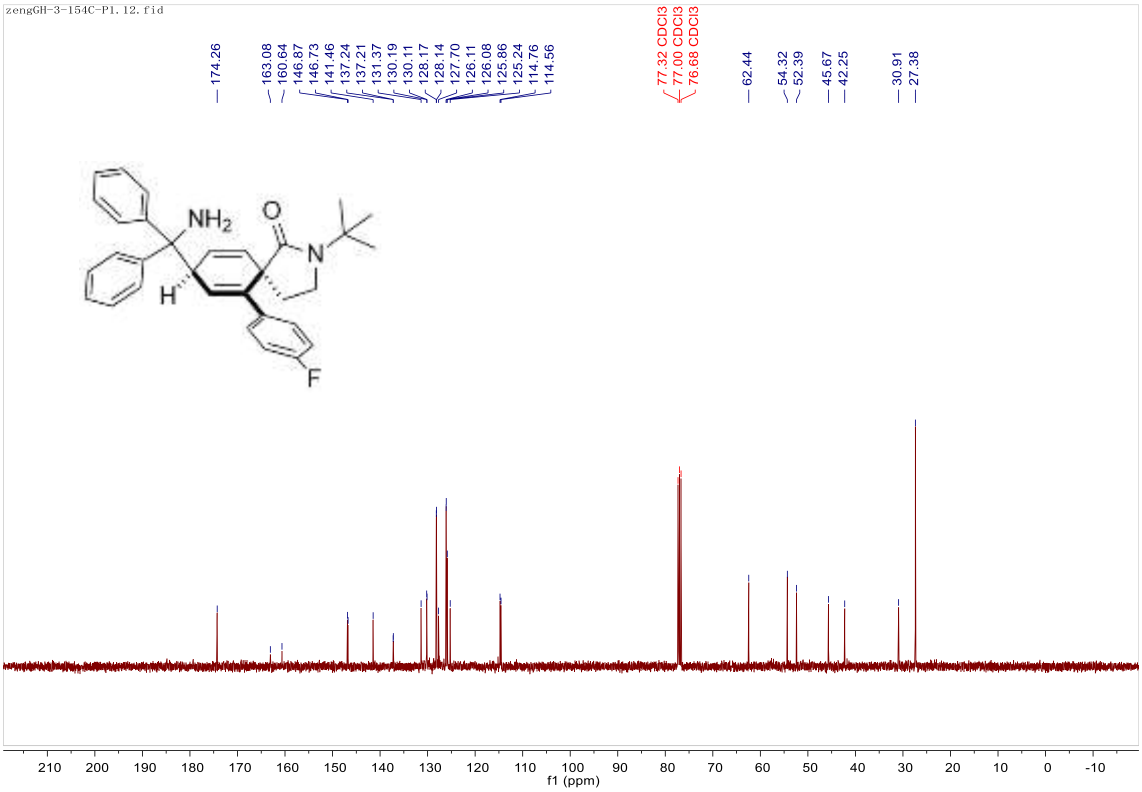

^{13}C−NMR for cis-**3-16**

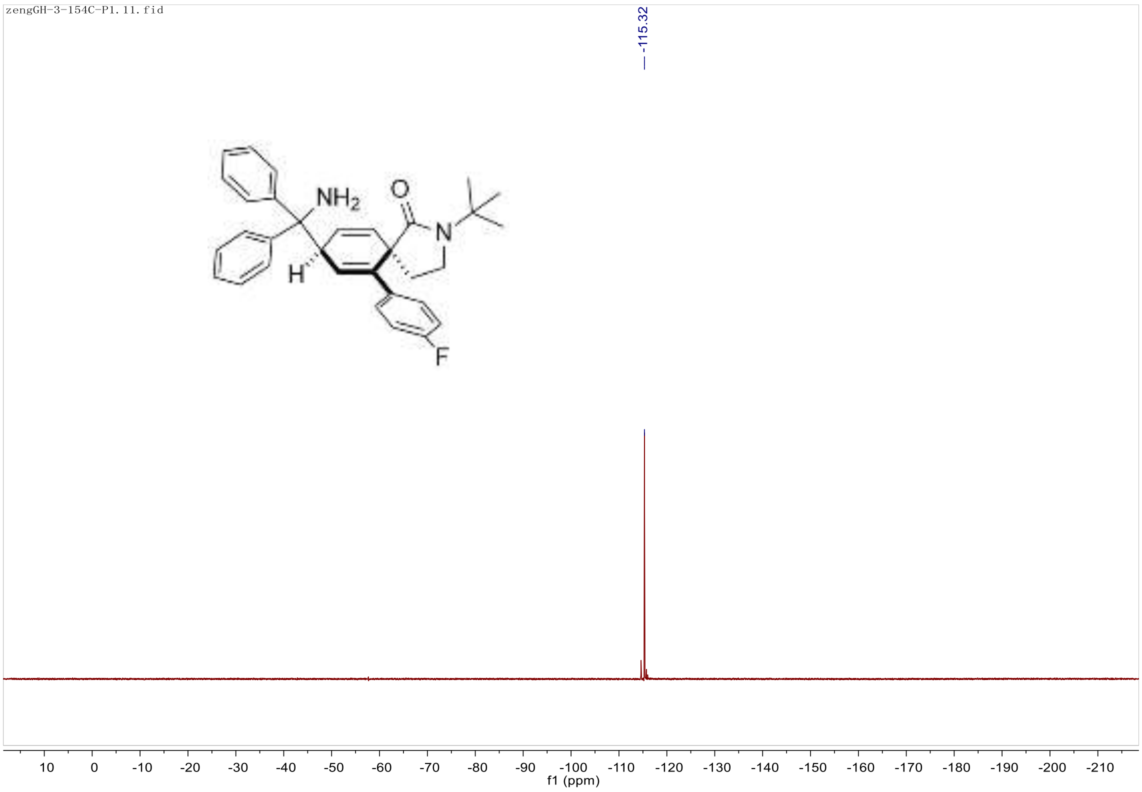

^{19}F-NMR for cis-**3-16**

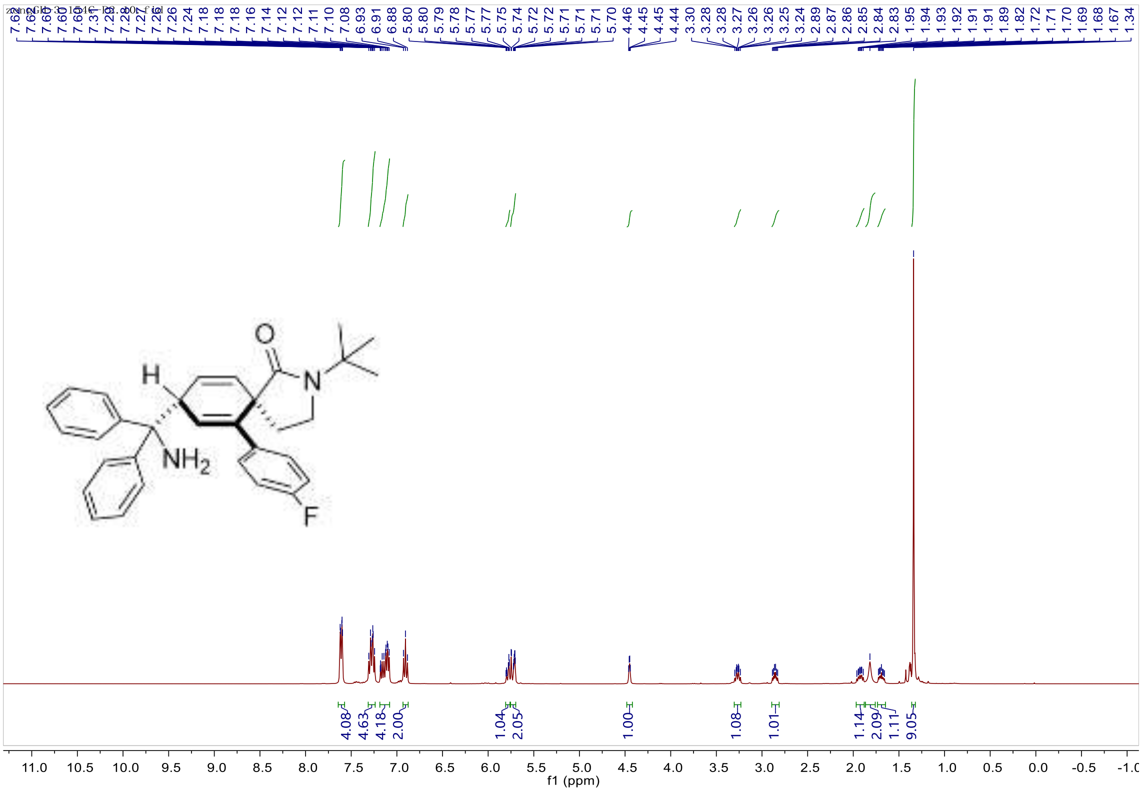

^{1}H-NMR for trans-**3-16**

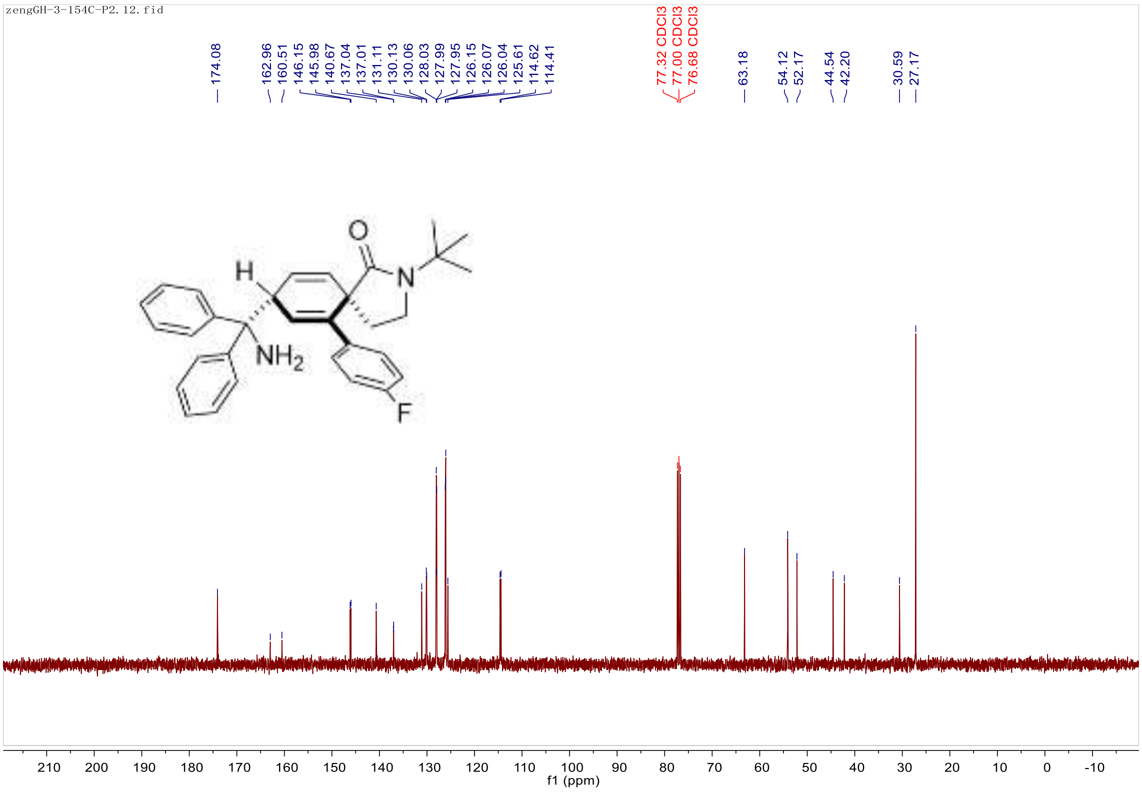

^{13}C−NMR for trans-**3-16**

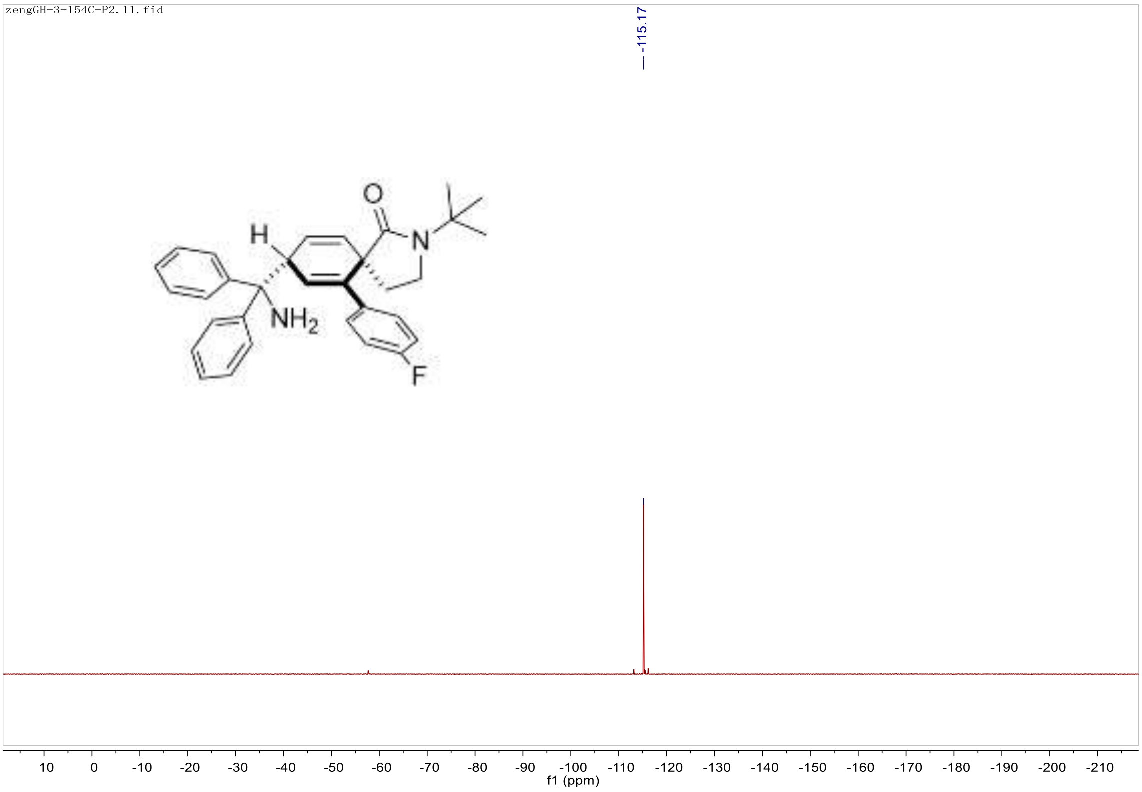

^{19}F-NMR for trans-**3-16**

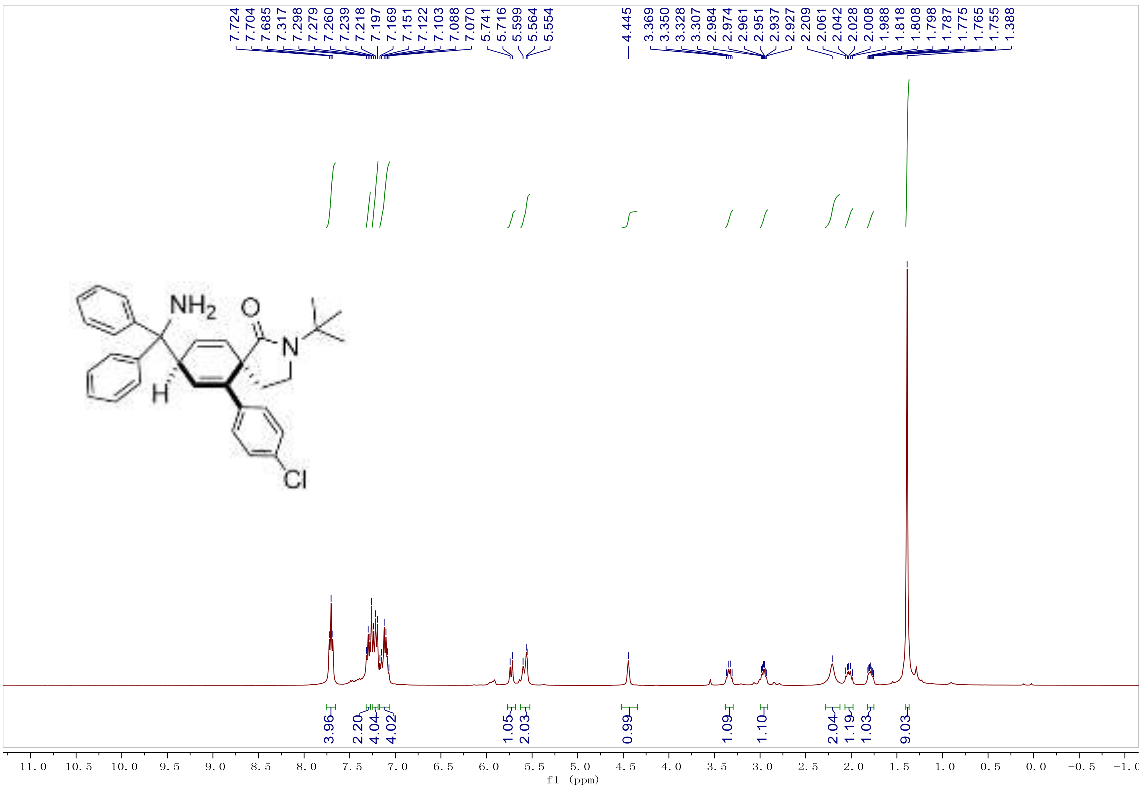

^{1}H-NMR for cis-**3-17**

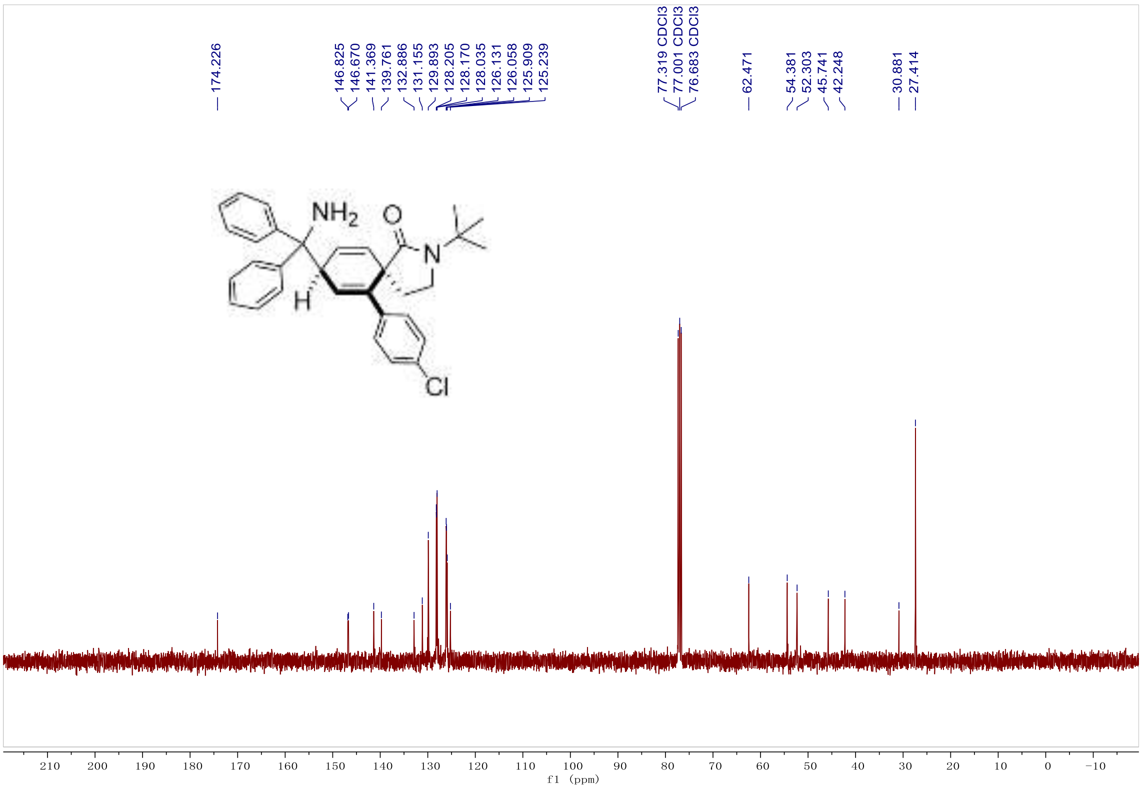

^{13}C−NMR for cis-**3-17**

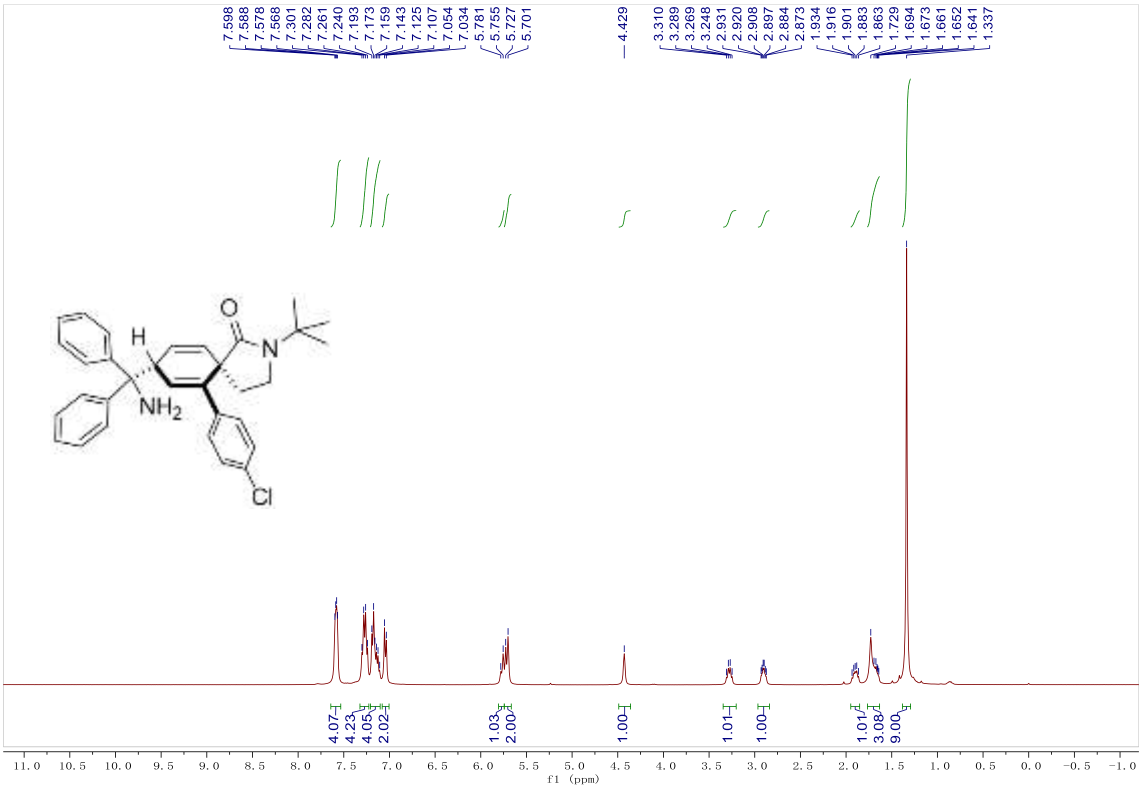

^{1}H-NMR for trans-**3-17**

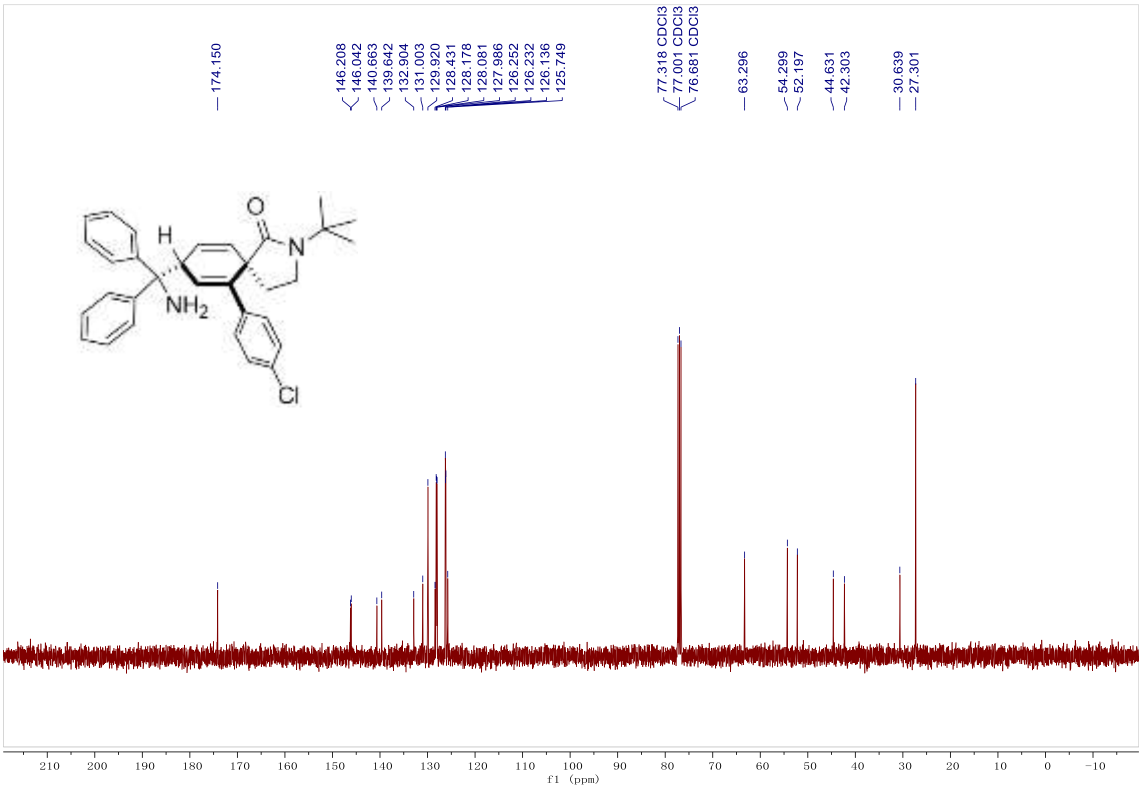

^{13}C−NMR for trans-**3-17**

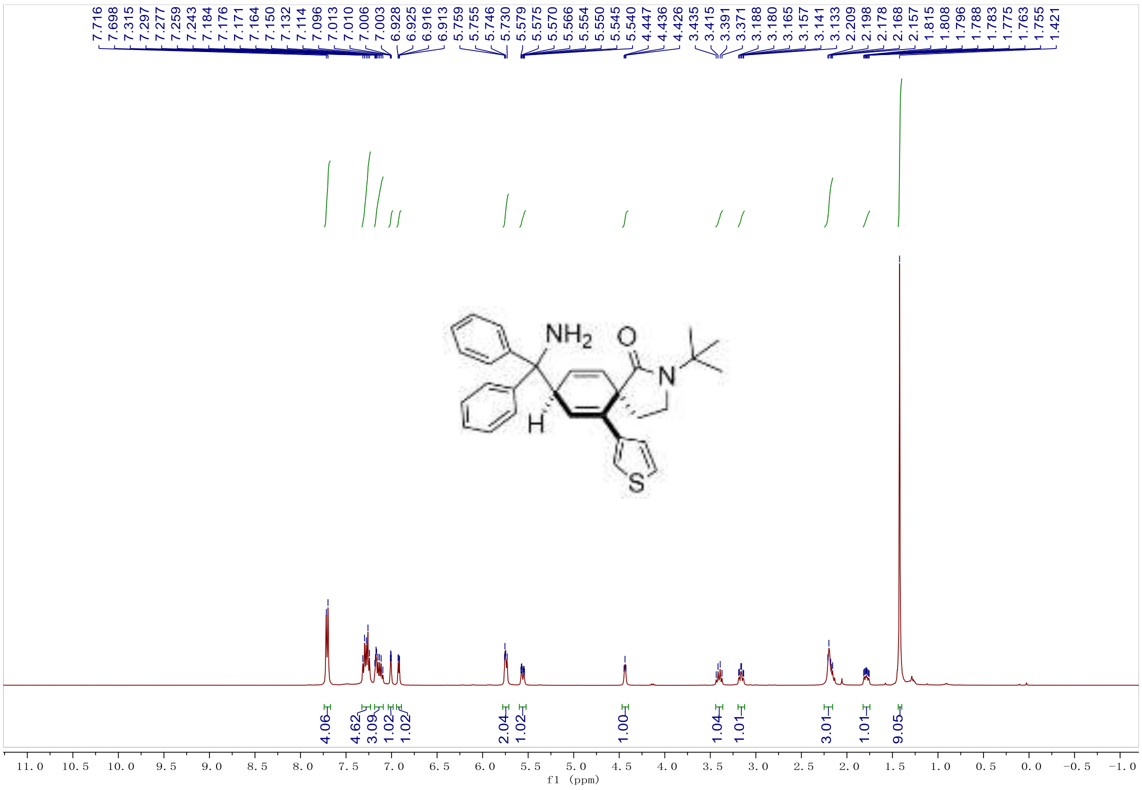

^{1}H-NMR for cis-**3-18**

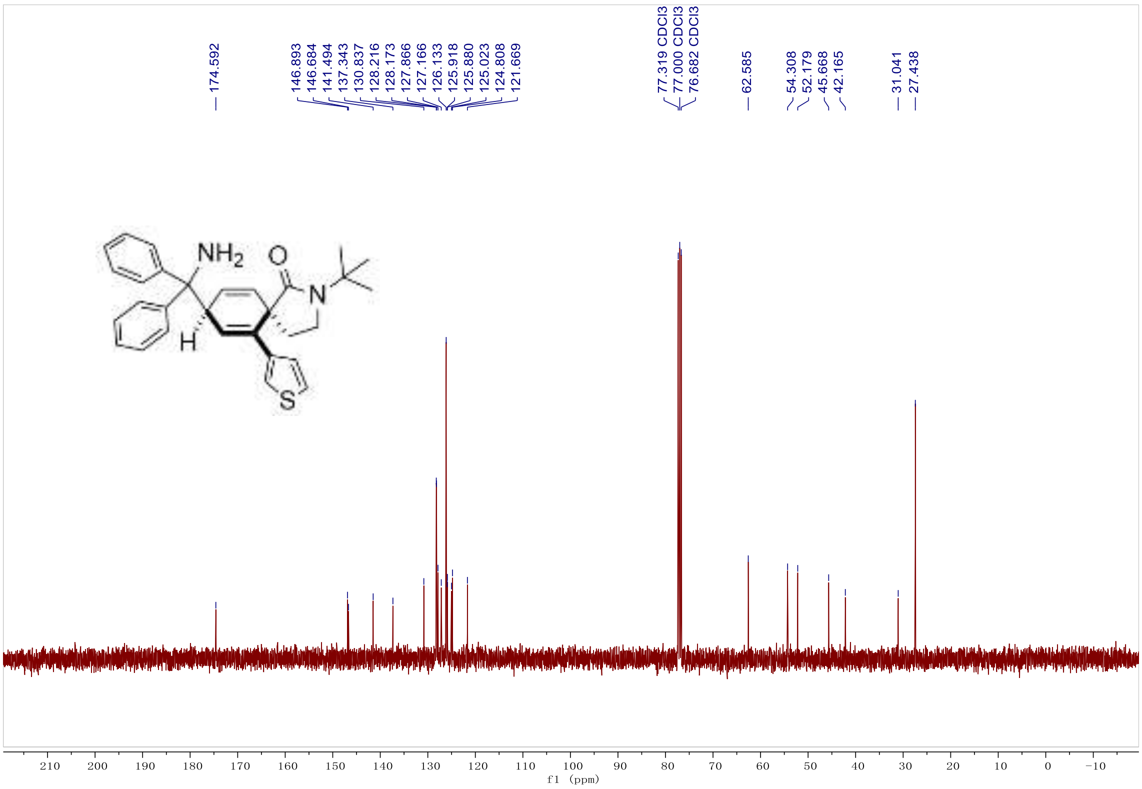

^{13}C−NMR for cis-**3-18**

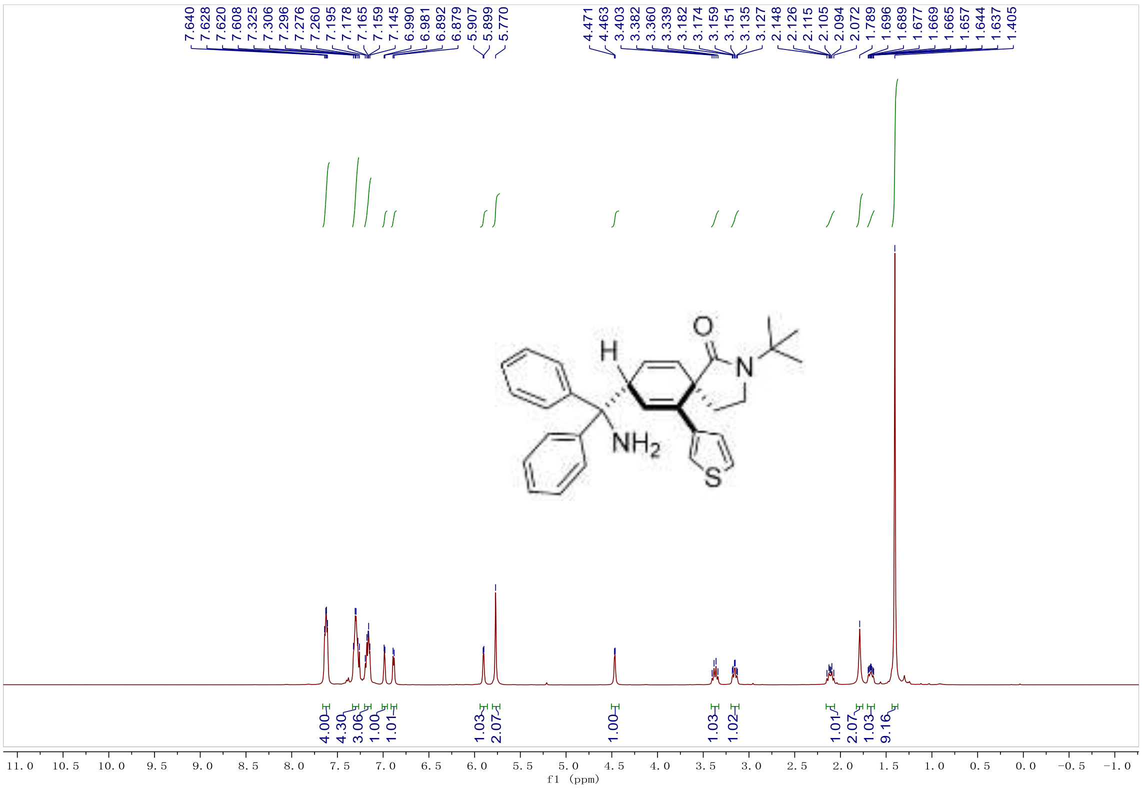

^{1}H-NMR for trans-**3-18**

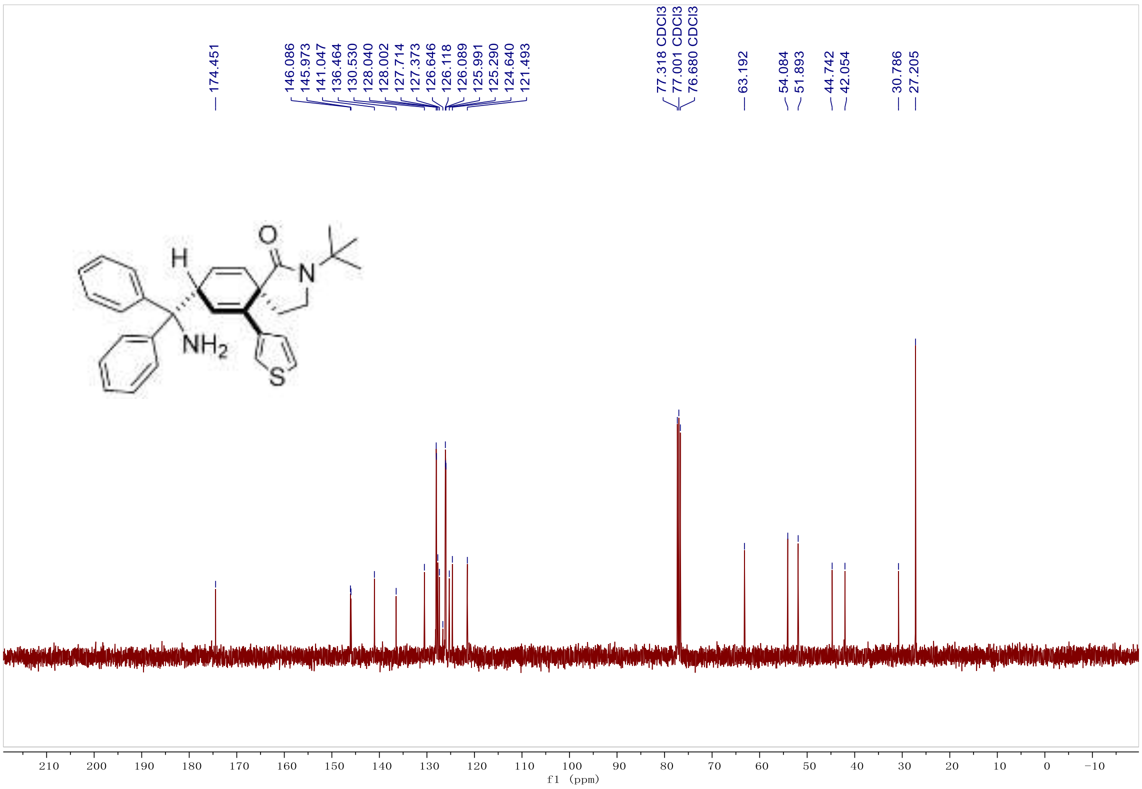

^{13}C−NMR for trans-**3-18**

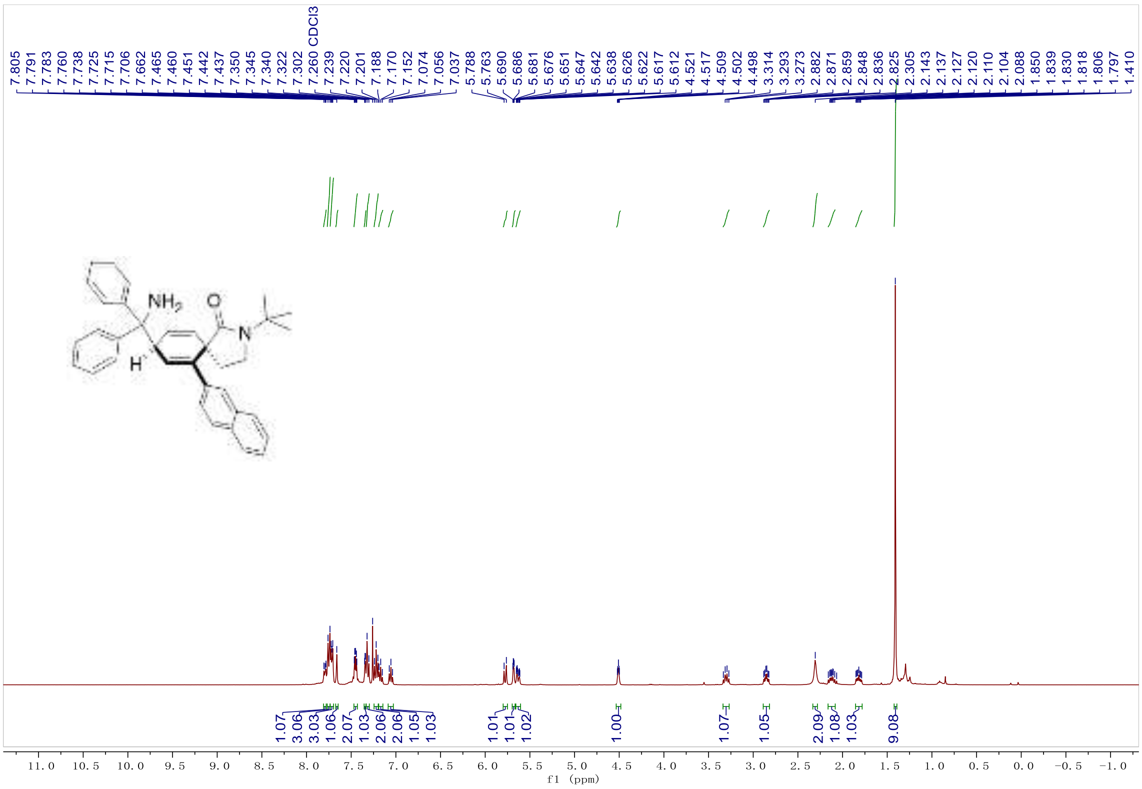

^{1}H-NMR for cis-**3-19**

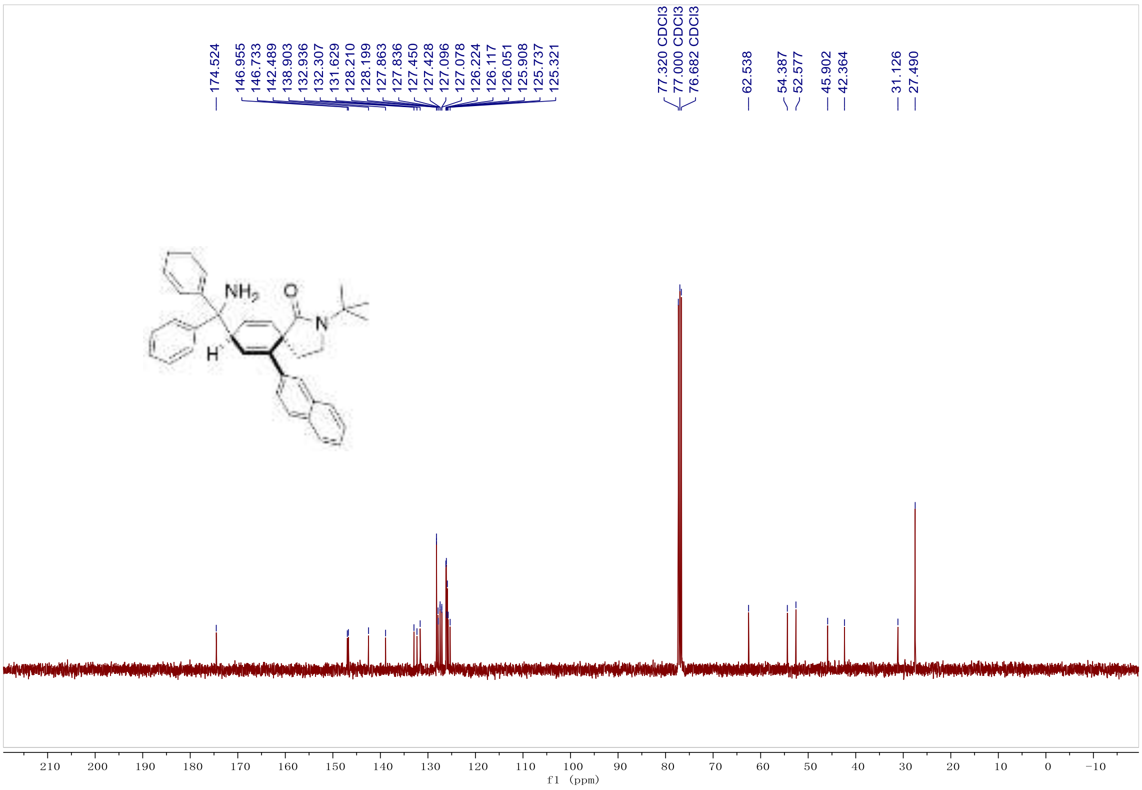

^{13}C−NMR for cis-**3-19**

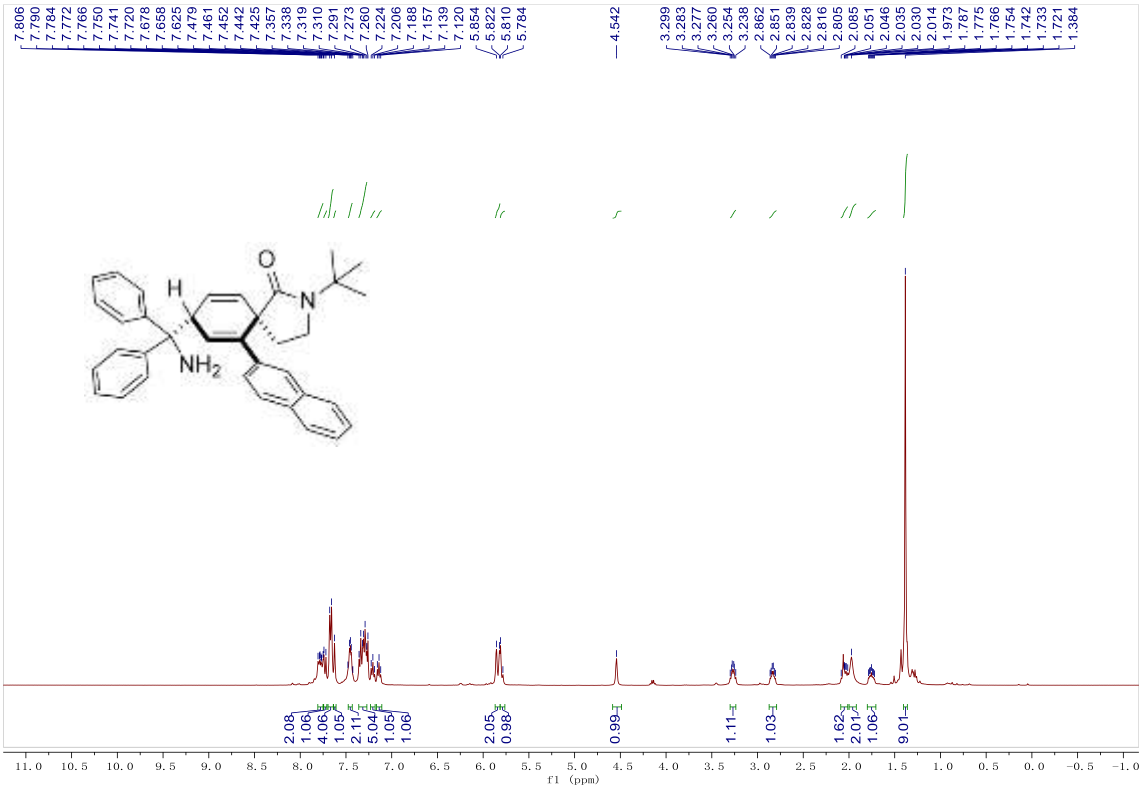

^{1}H-NMR for trans-**3-19**

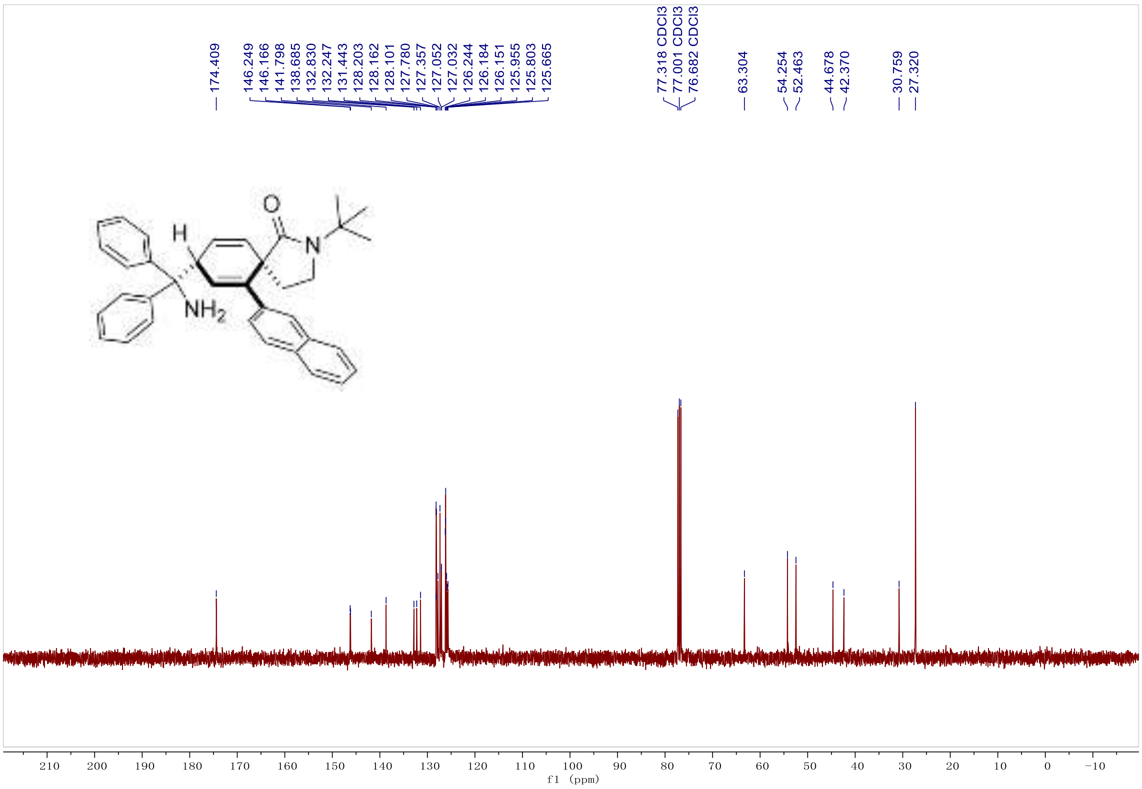

^{13}C−NMR for trans-**3-19**

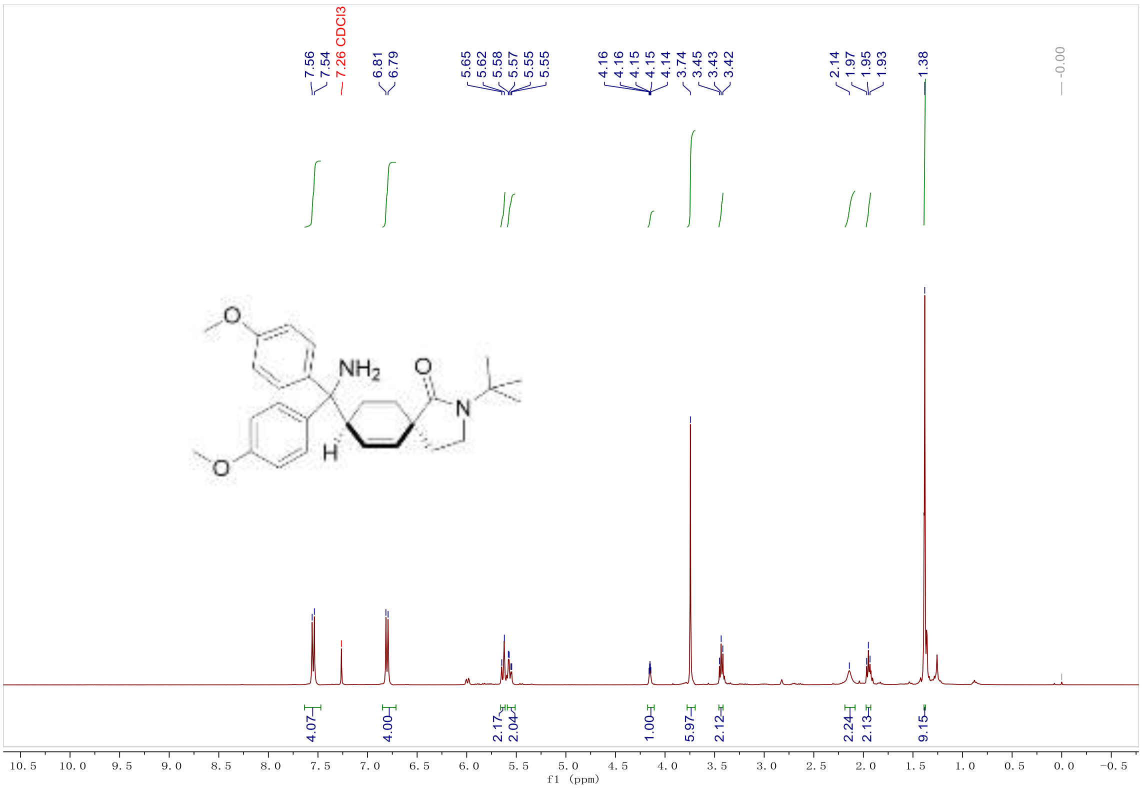

^{1}H-NMR for cis-**3-20**

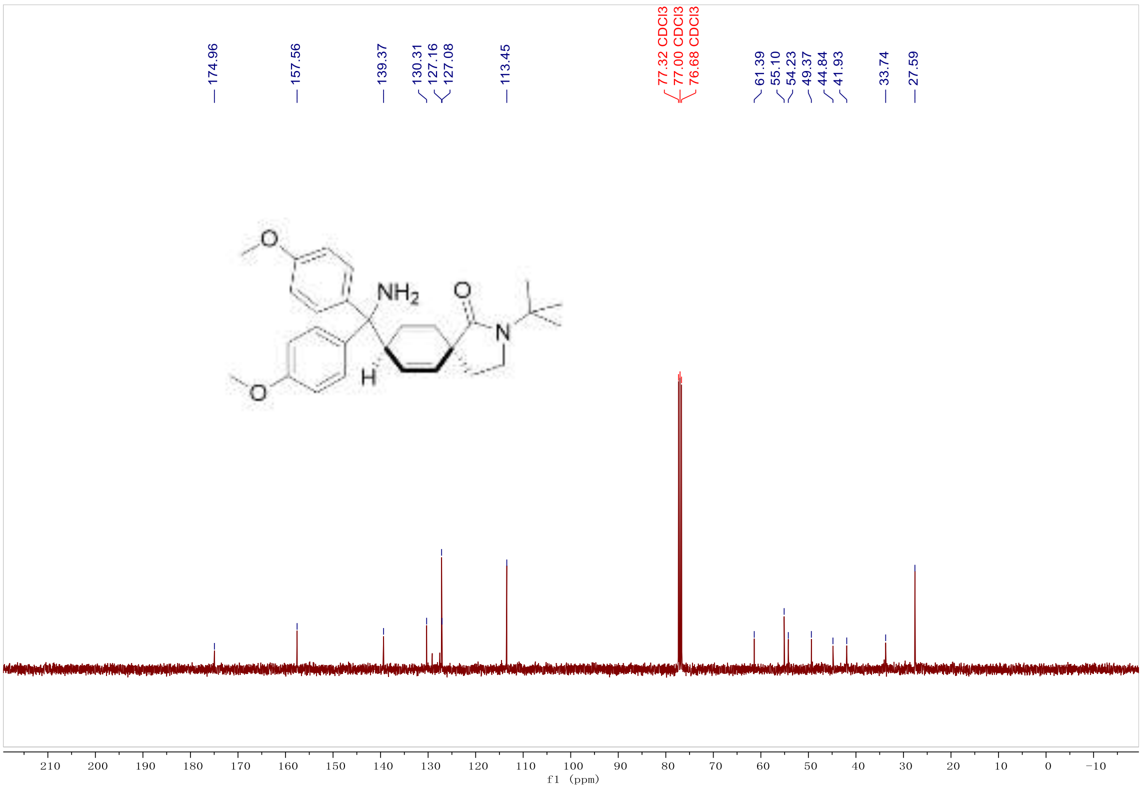

^{13}C−NMR for cis-**3-20**

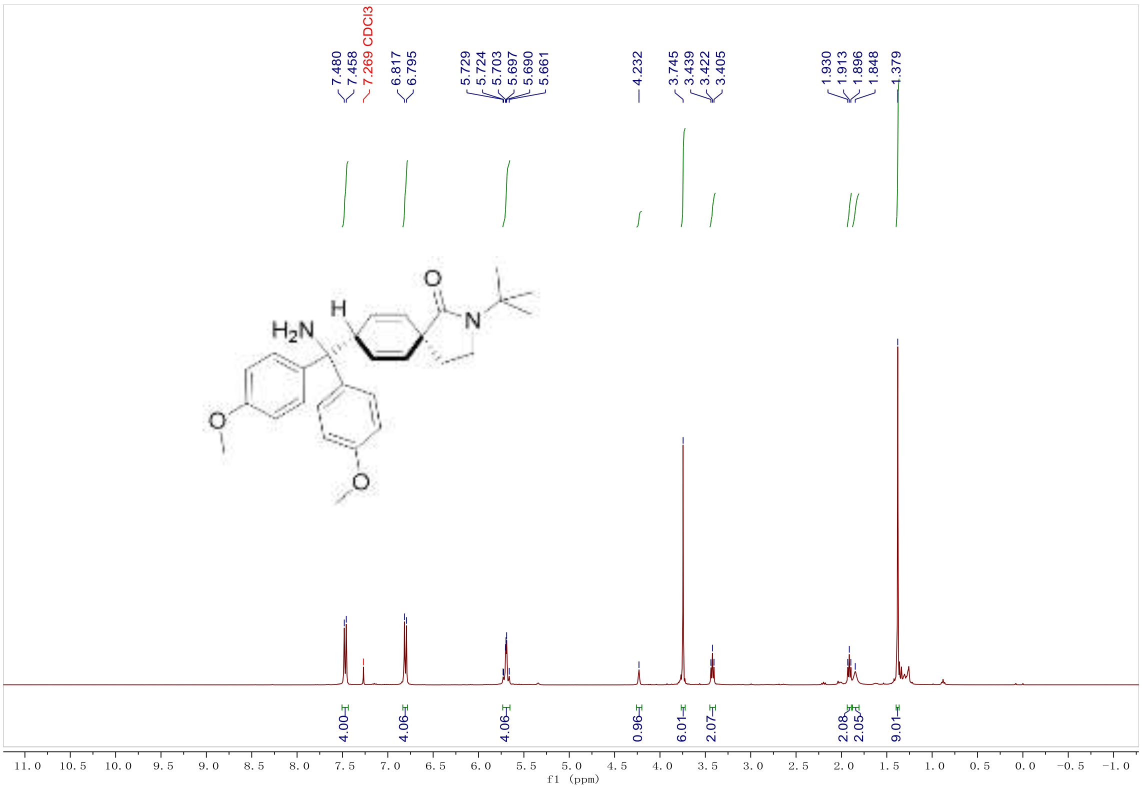

^{1}H-NMR for trans-**3-20**

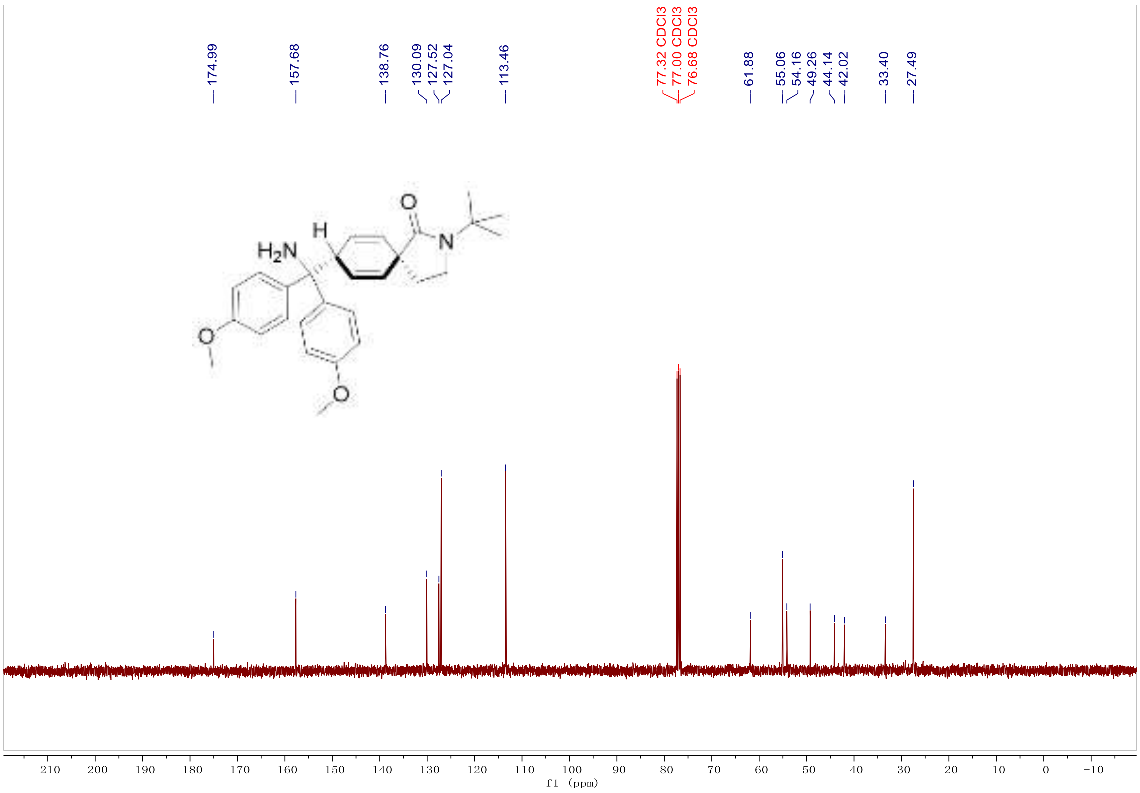

^{13}C−NMR for trans-**3-20**

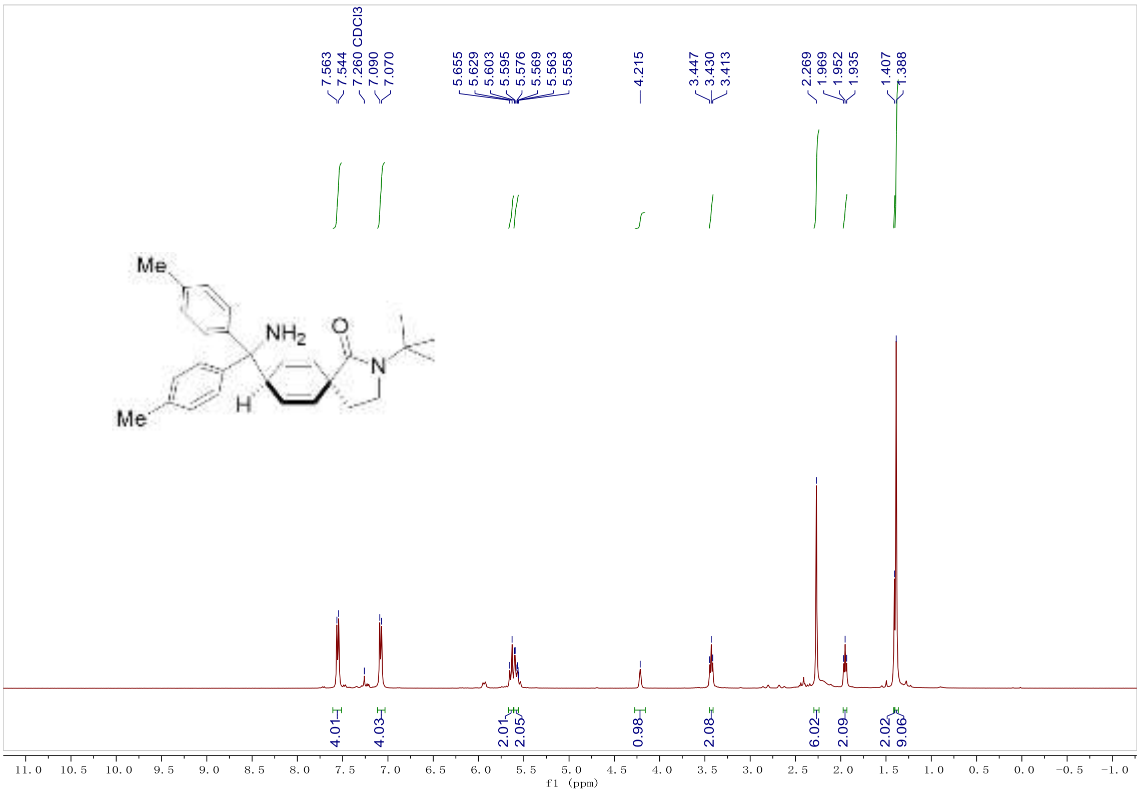

^{1}H-NMR for cis-**3-21**

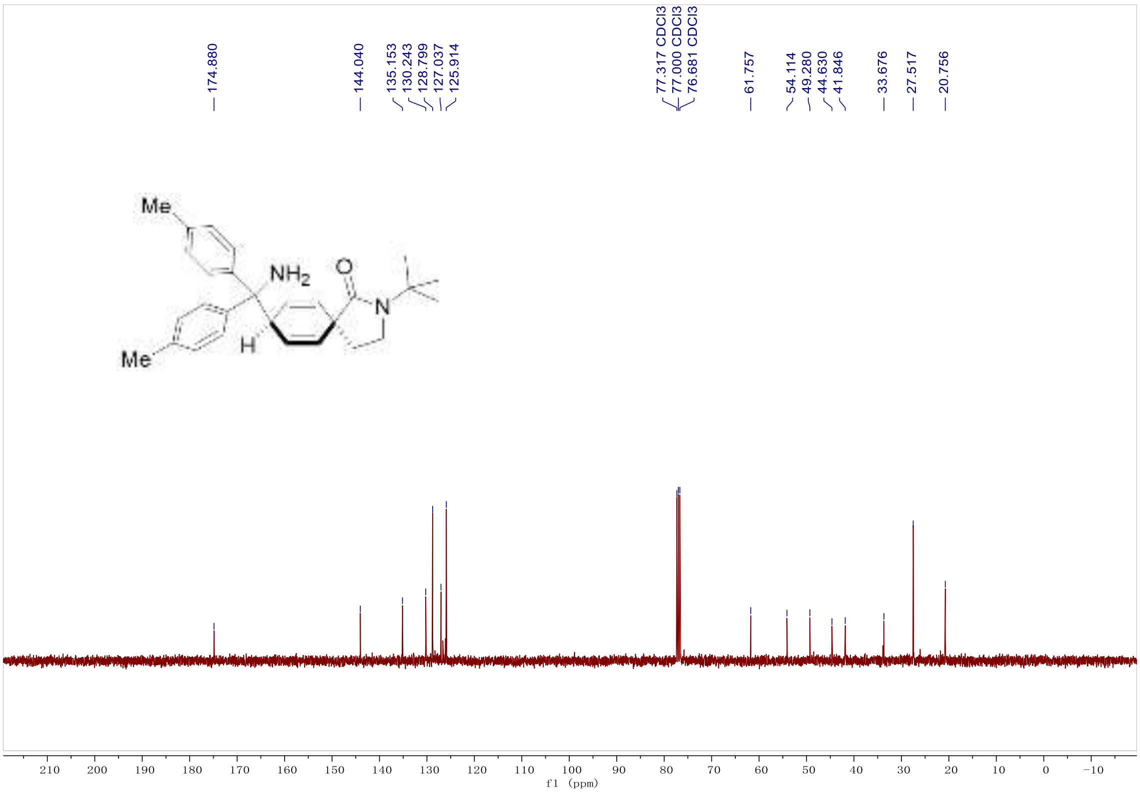

^{13}C−NMR for cis-**3-21**

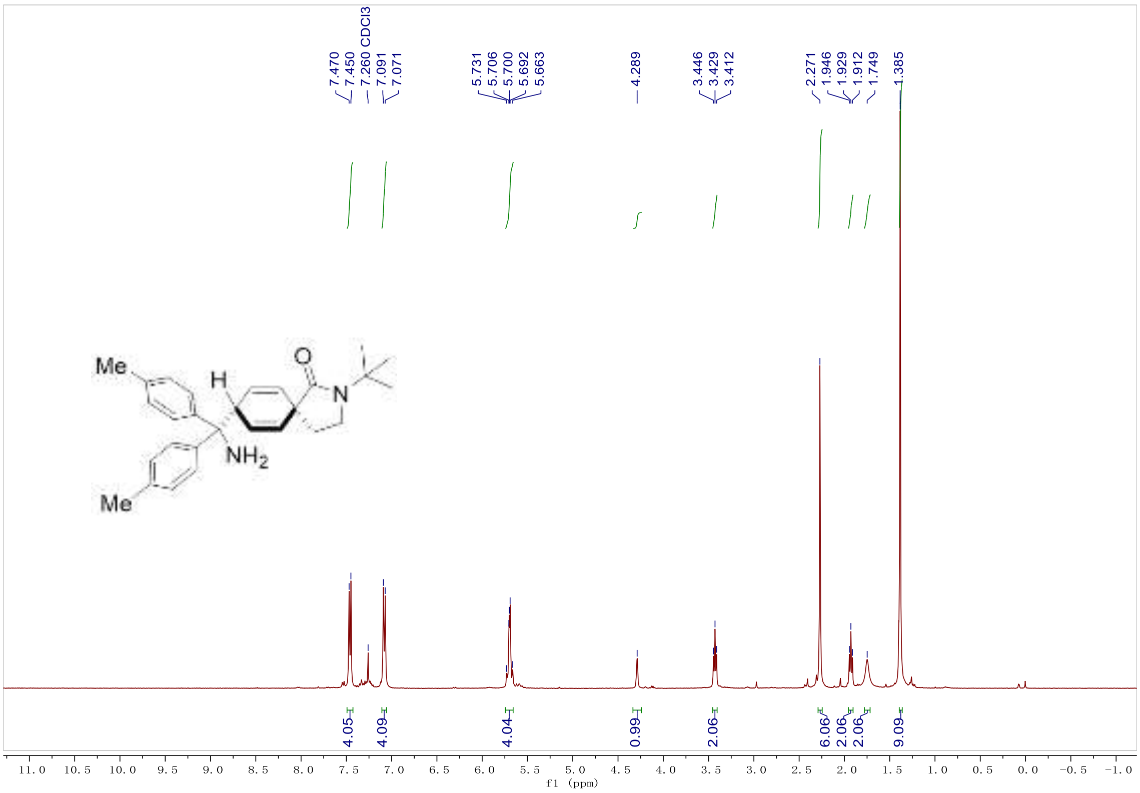

^{1}H-NMR for trans-**3-21**

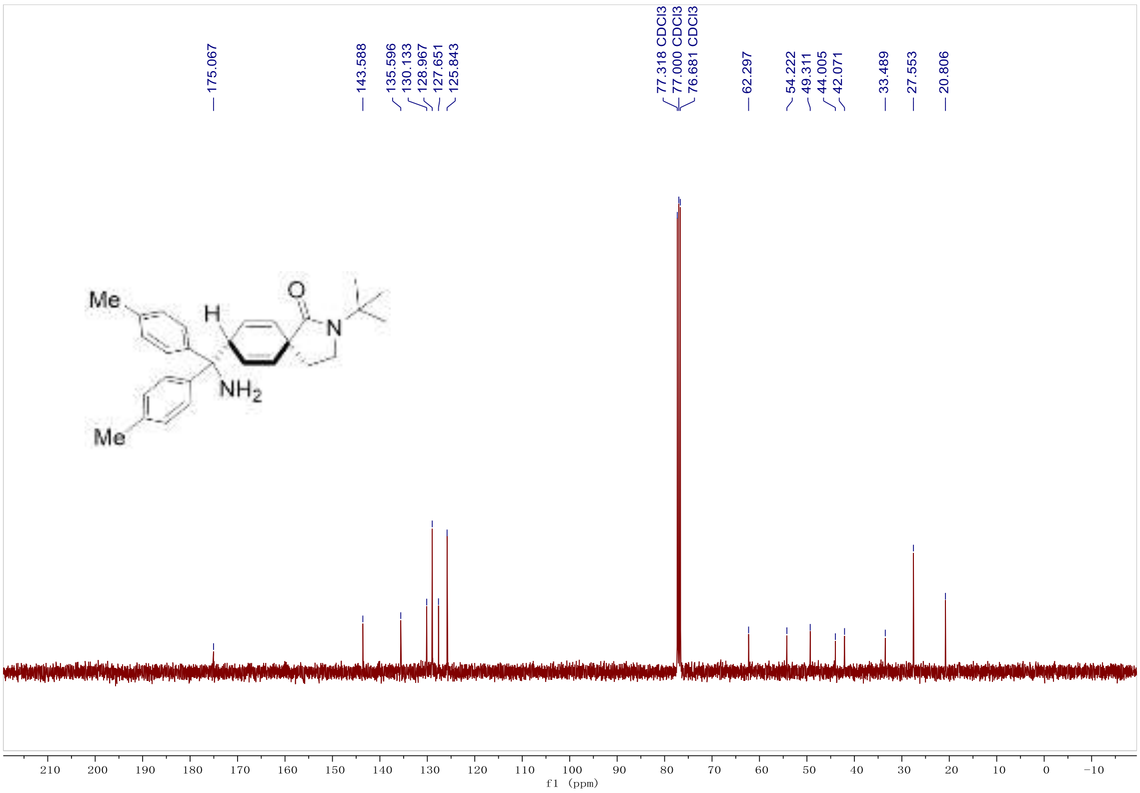

^{13}C−NMR for trans-**3-21**

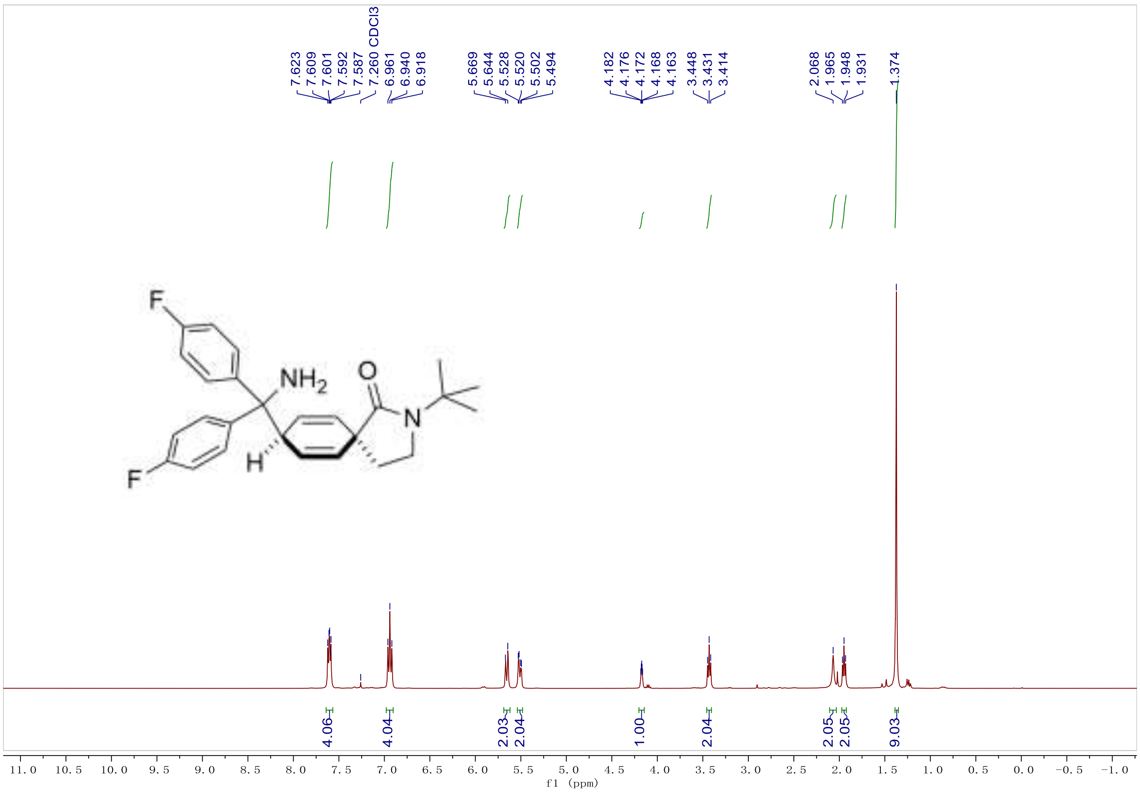

^{1}H-NMR for cis-**3-22**

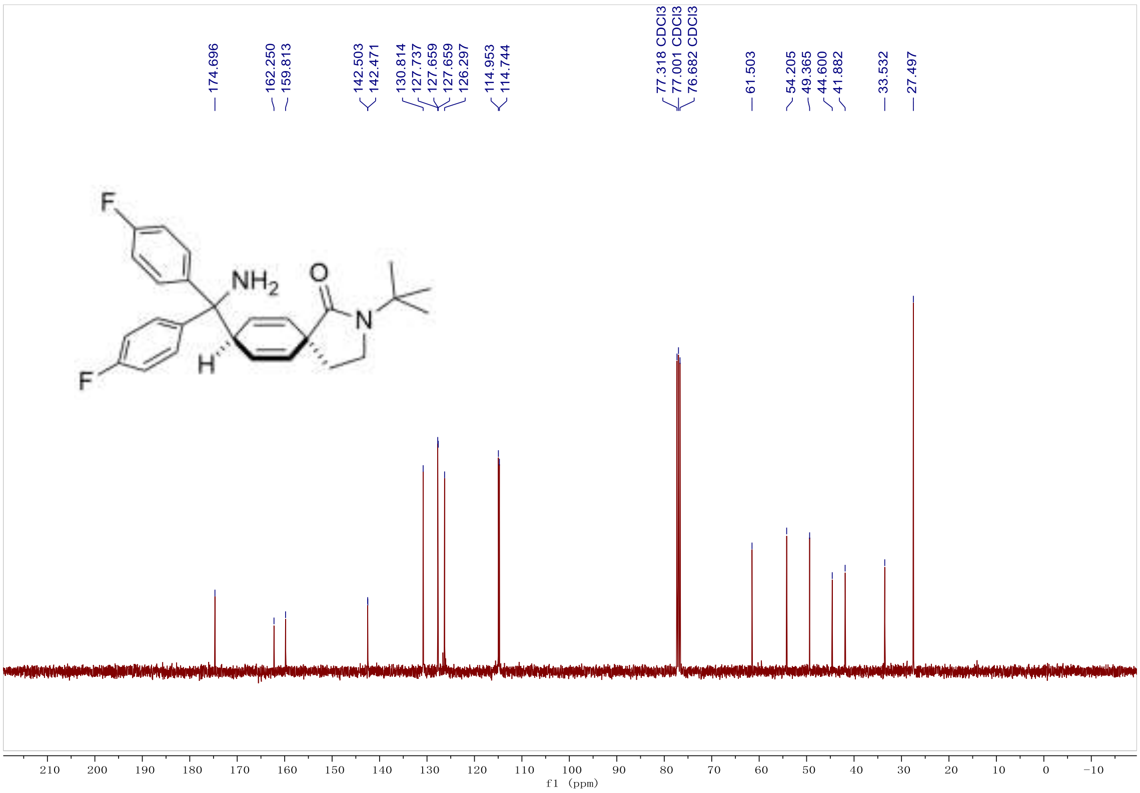

^{13}C−NMR for cis-**3-22**

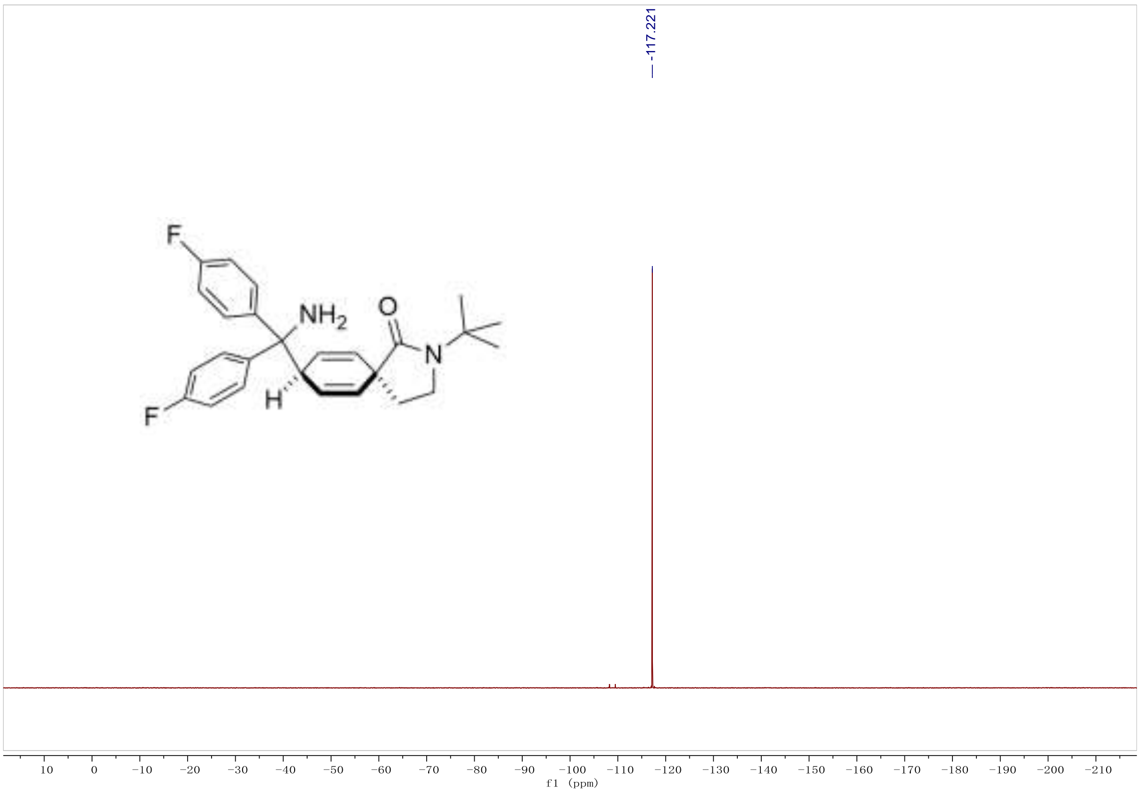

^{19}F-NMR for cis-**3-22**

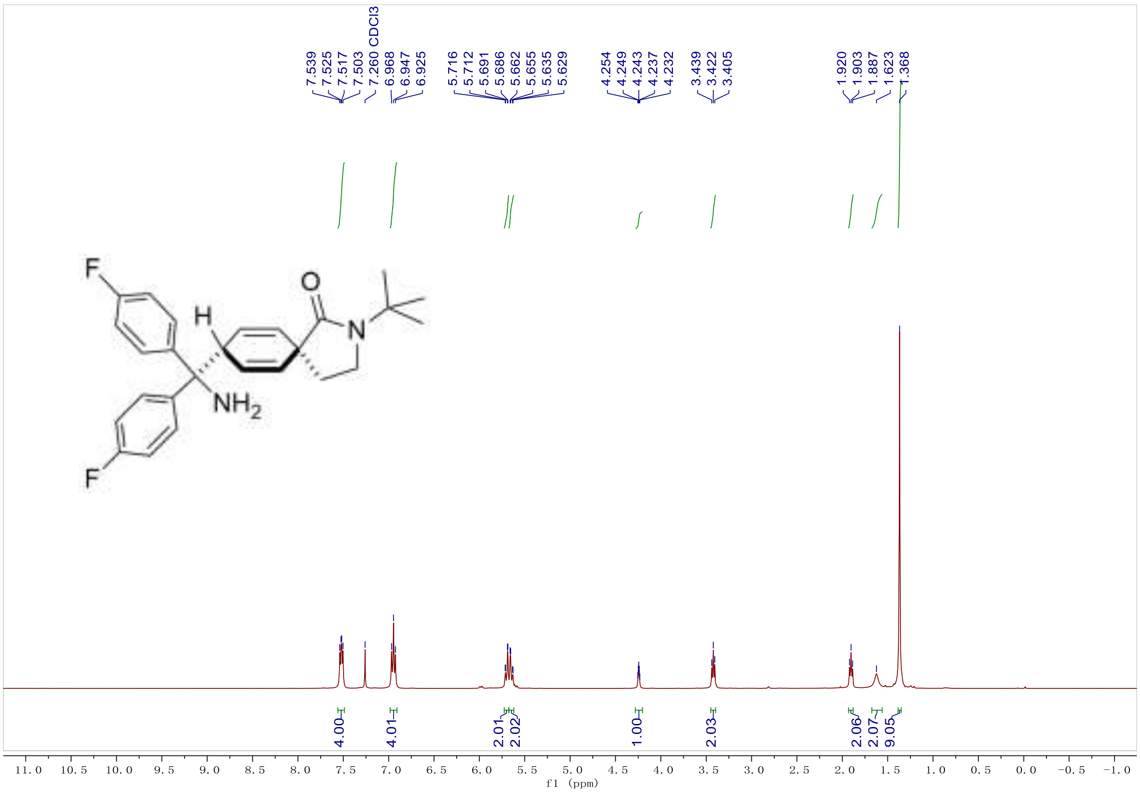

^{1}H-NMR for trans-**3-22**

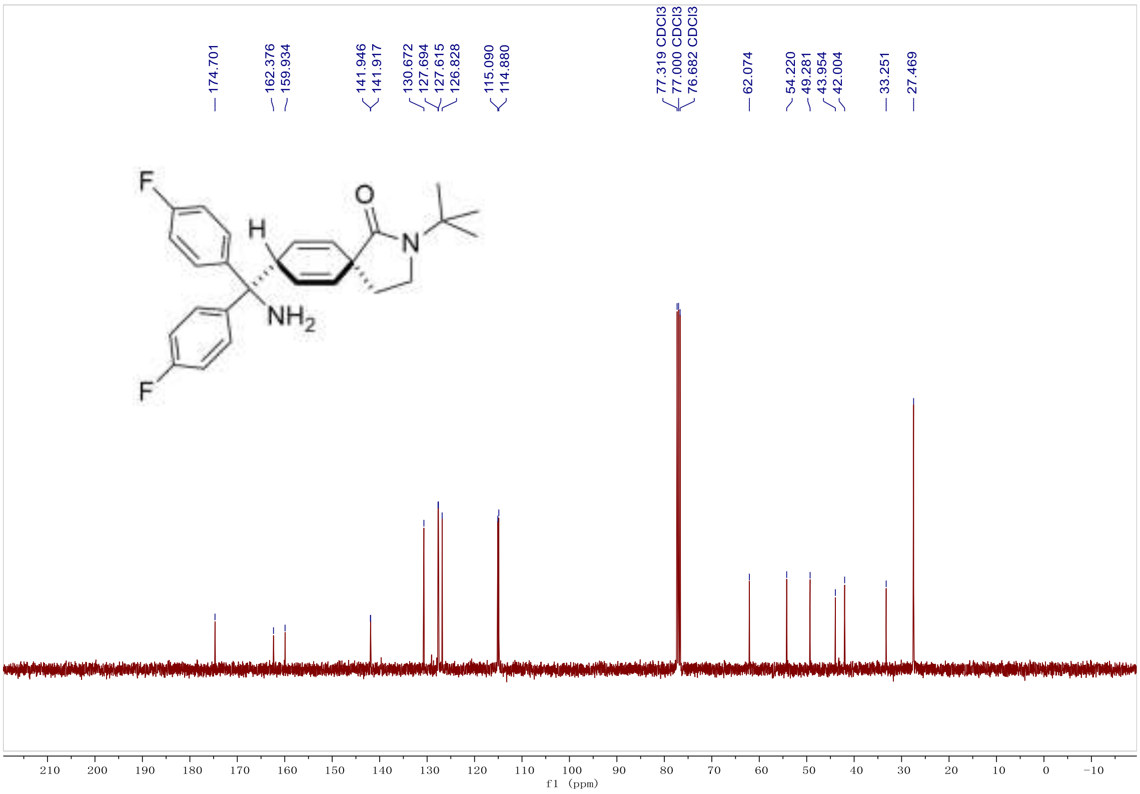

^{13}C−NMR for trans-**3-22**

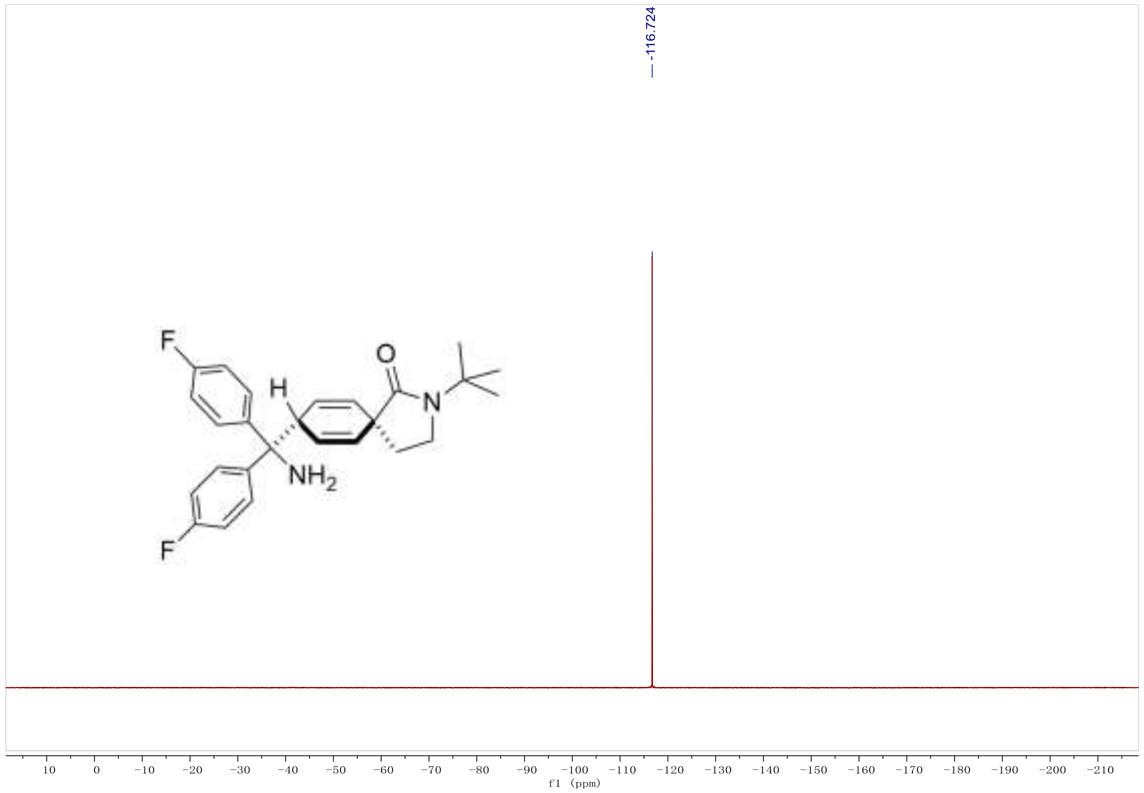

^{19}F-NMR for trans-**3-22**

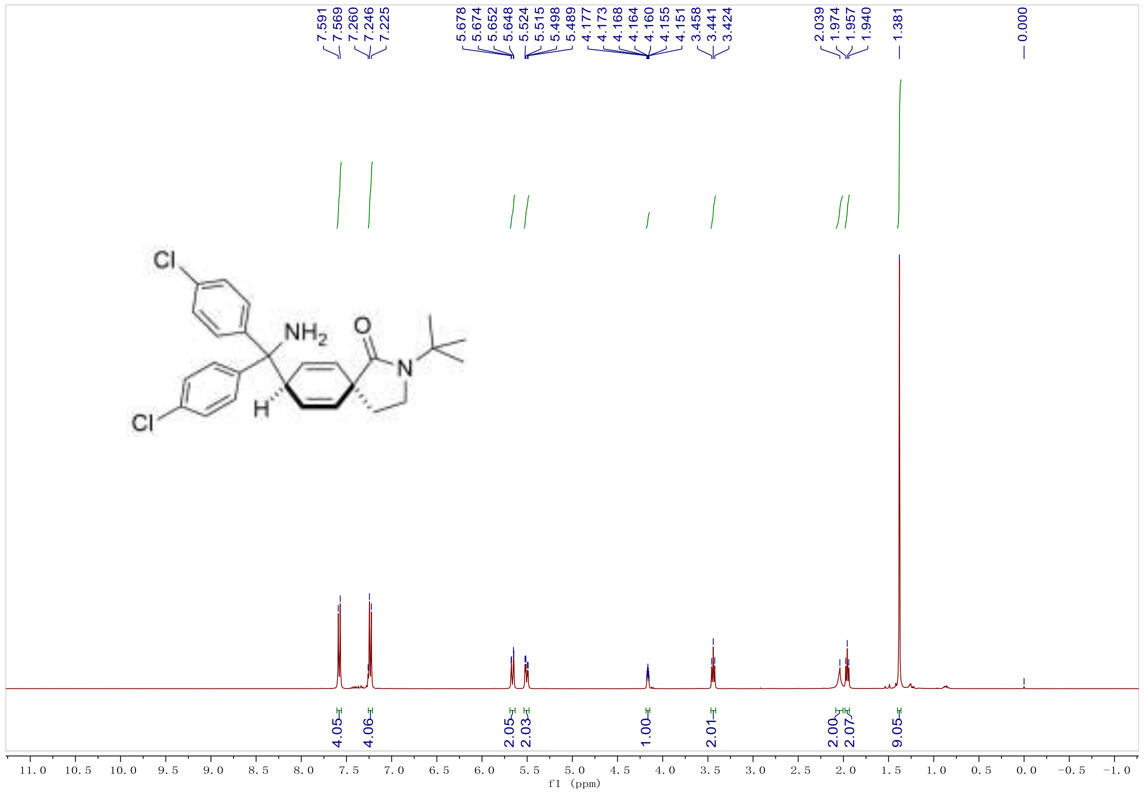

^{1}H-NMR for cis-**3-23**

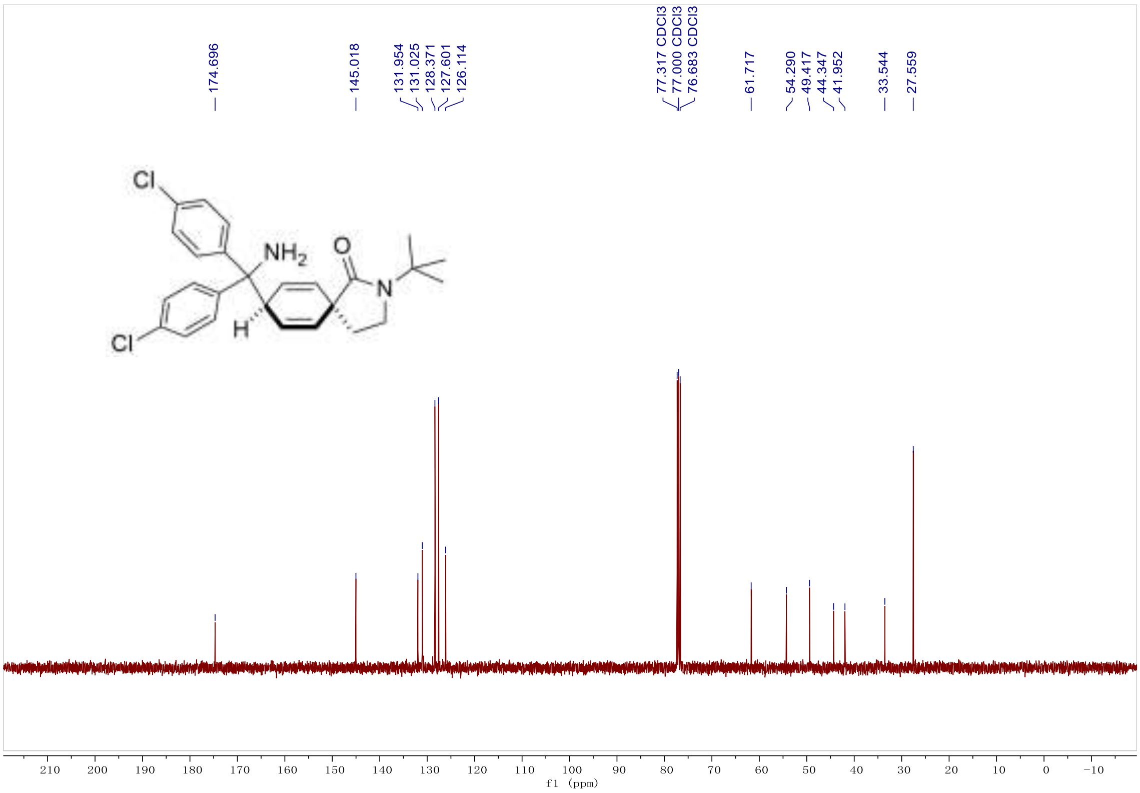

^{13}C−NMR for cis-**3-23**

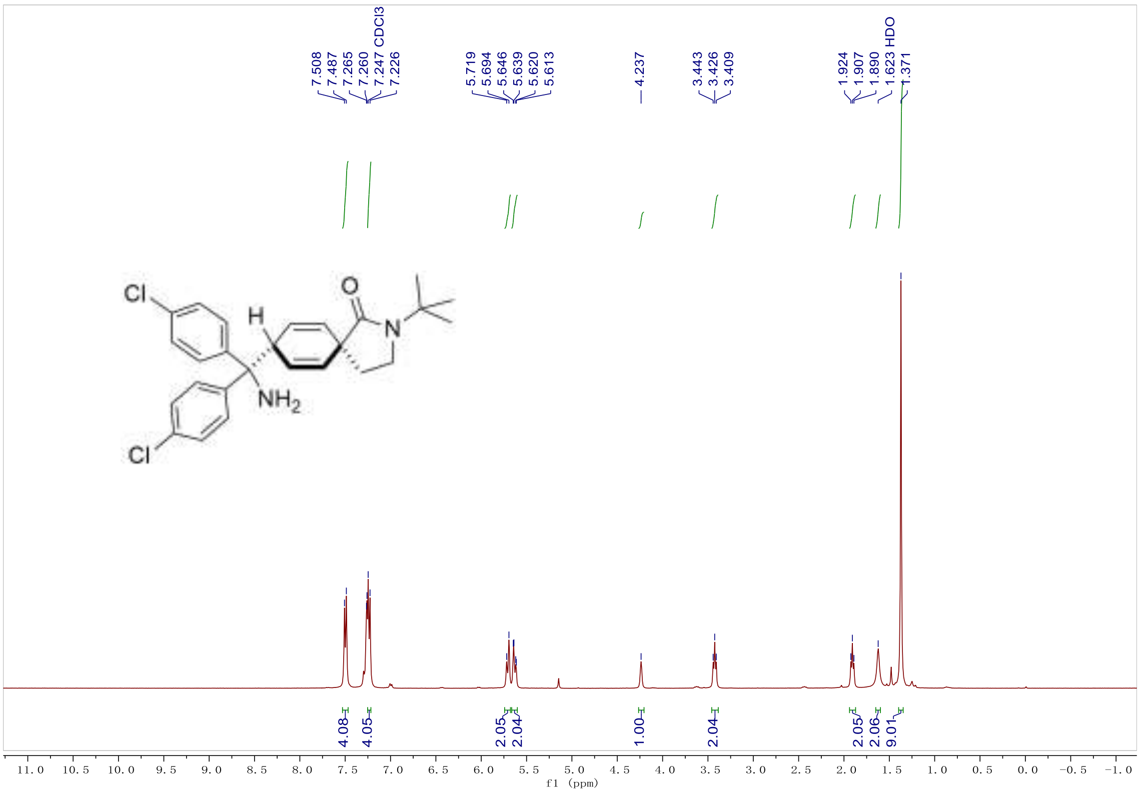

^{1}H-NMR for trans-**3-23**

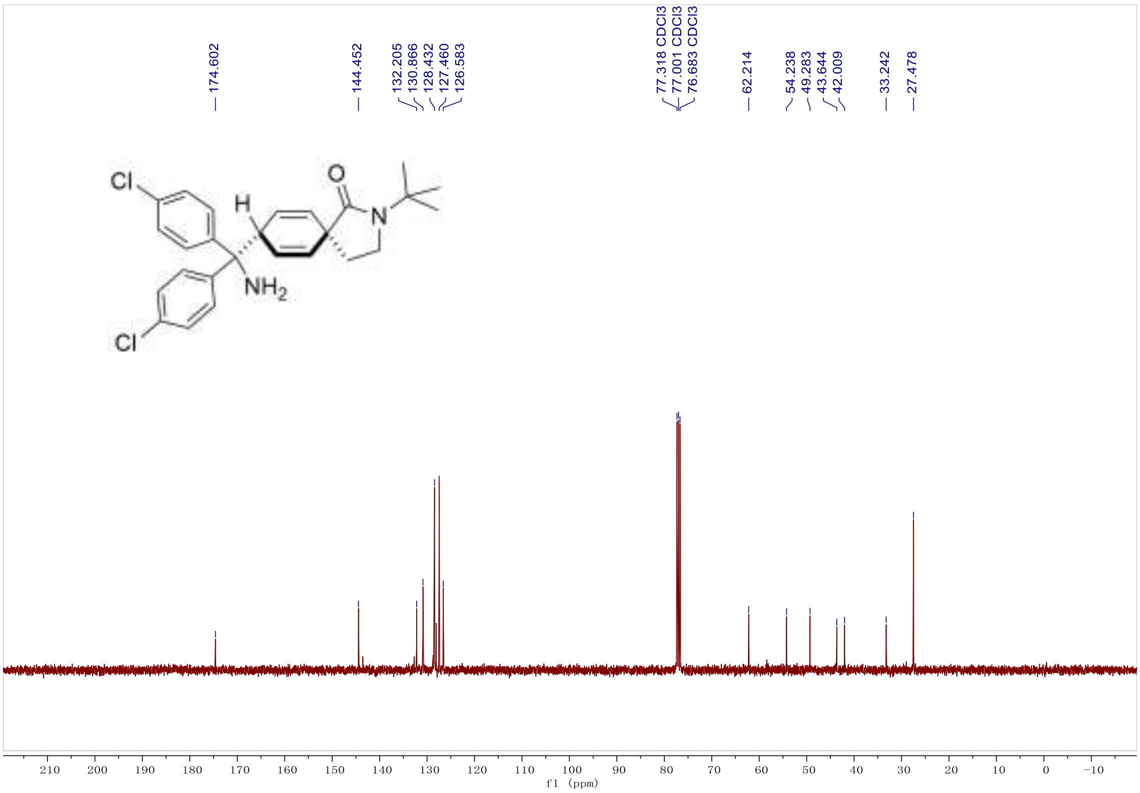

^{13}C−NMR for trans-**3-23**

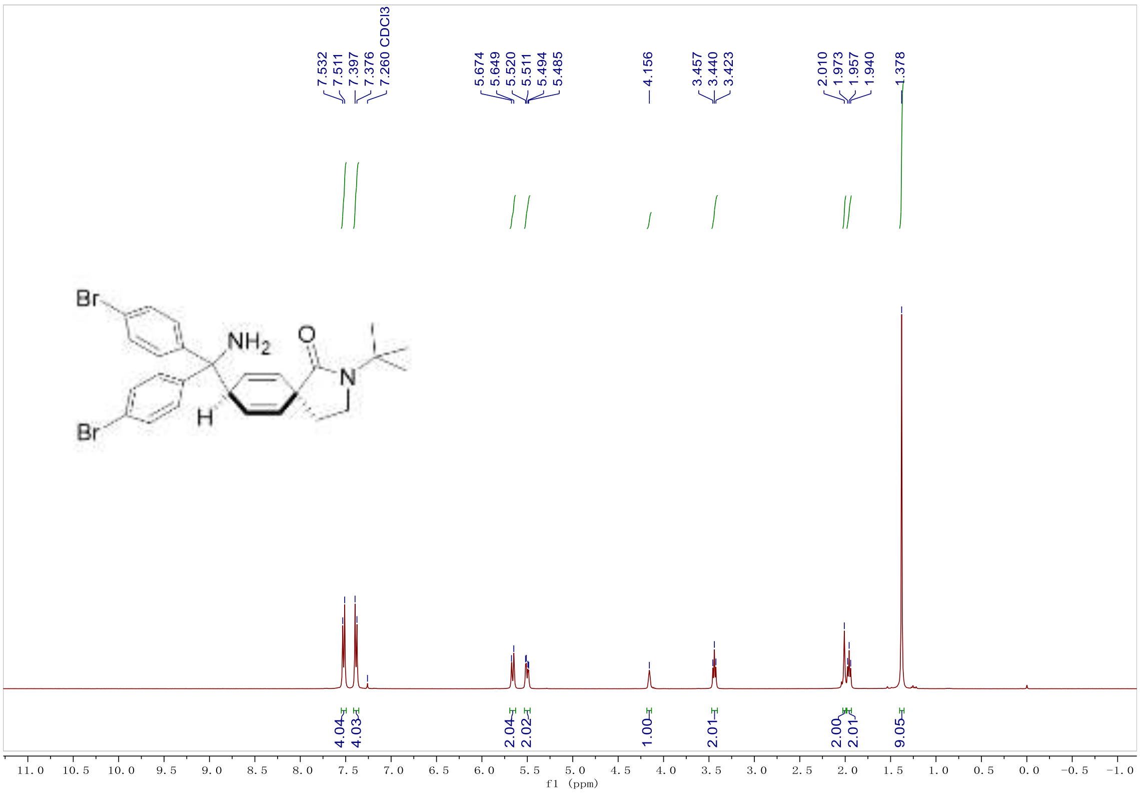

^{1}H-NMR for cis-**3-24**

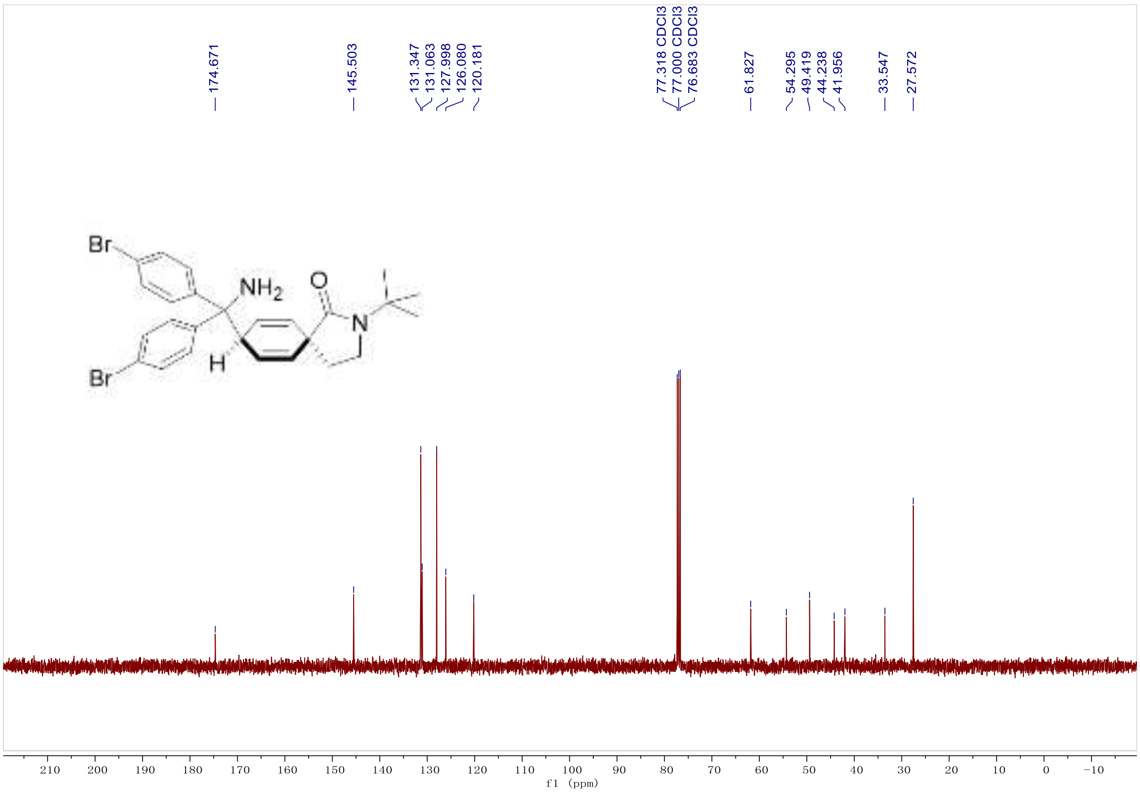

^{13}C−NMR for cis-**3-24**

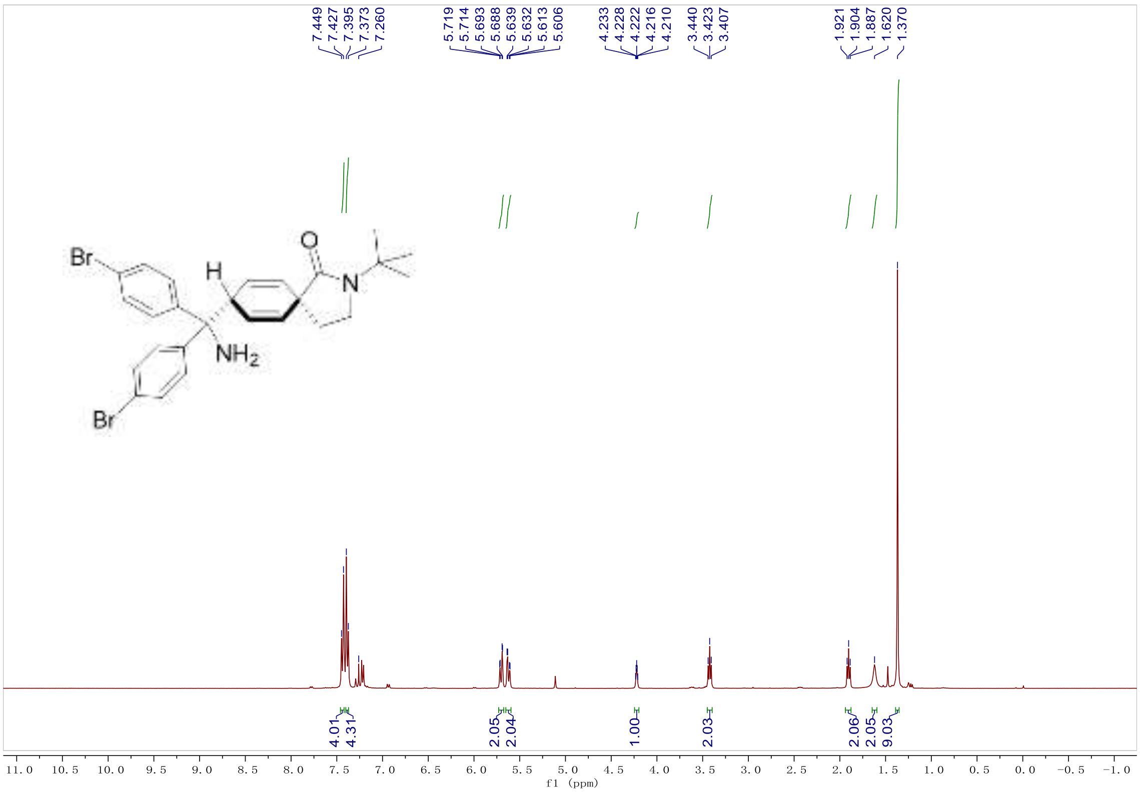

^{1}H-NMR for trans-**3-24**

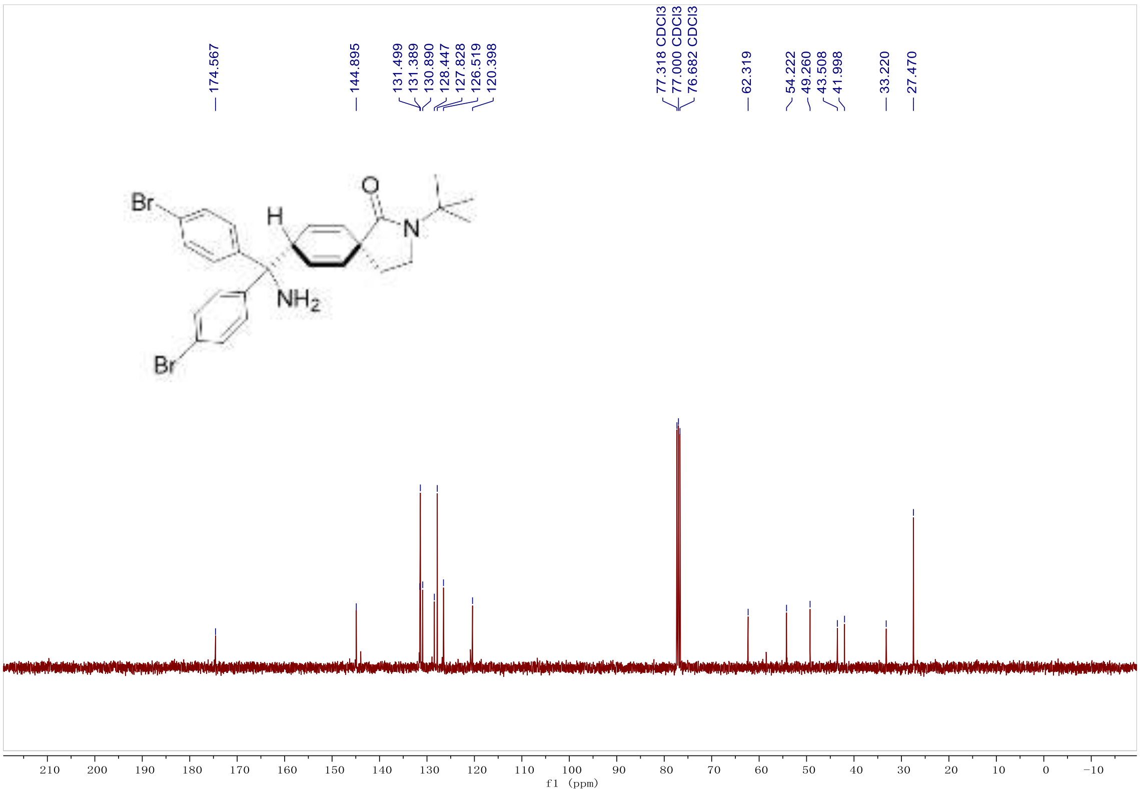

^{13}C−NMR for trans-**3-24**

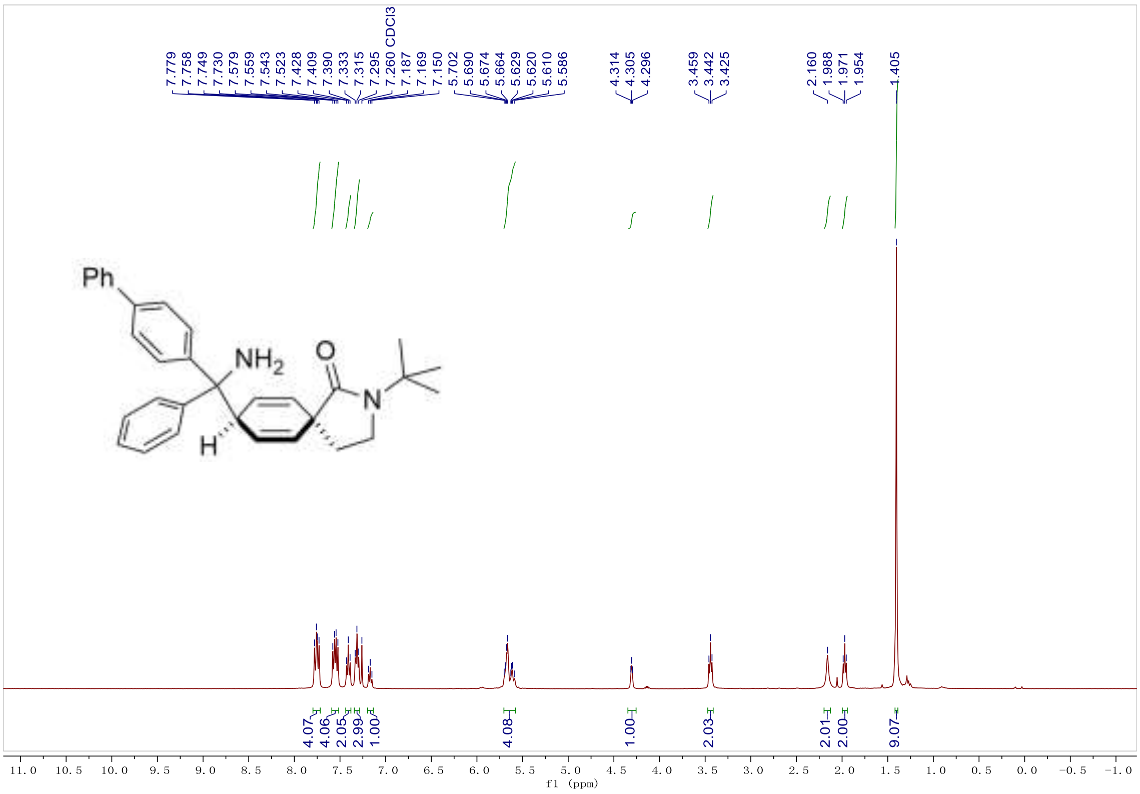

^{1}H-NMR for cis-**3-25**

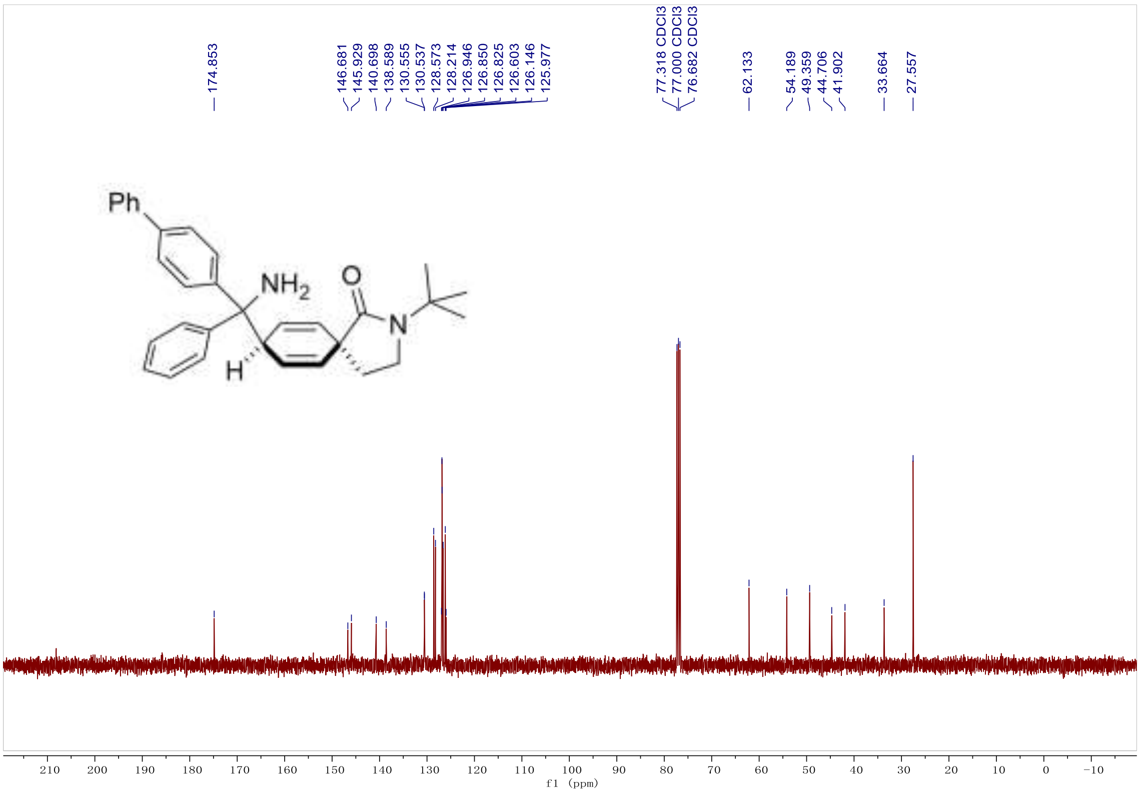

^{13}C−NMR for cis-**3-25**

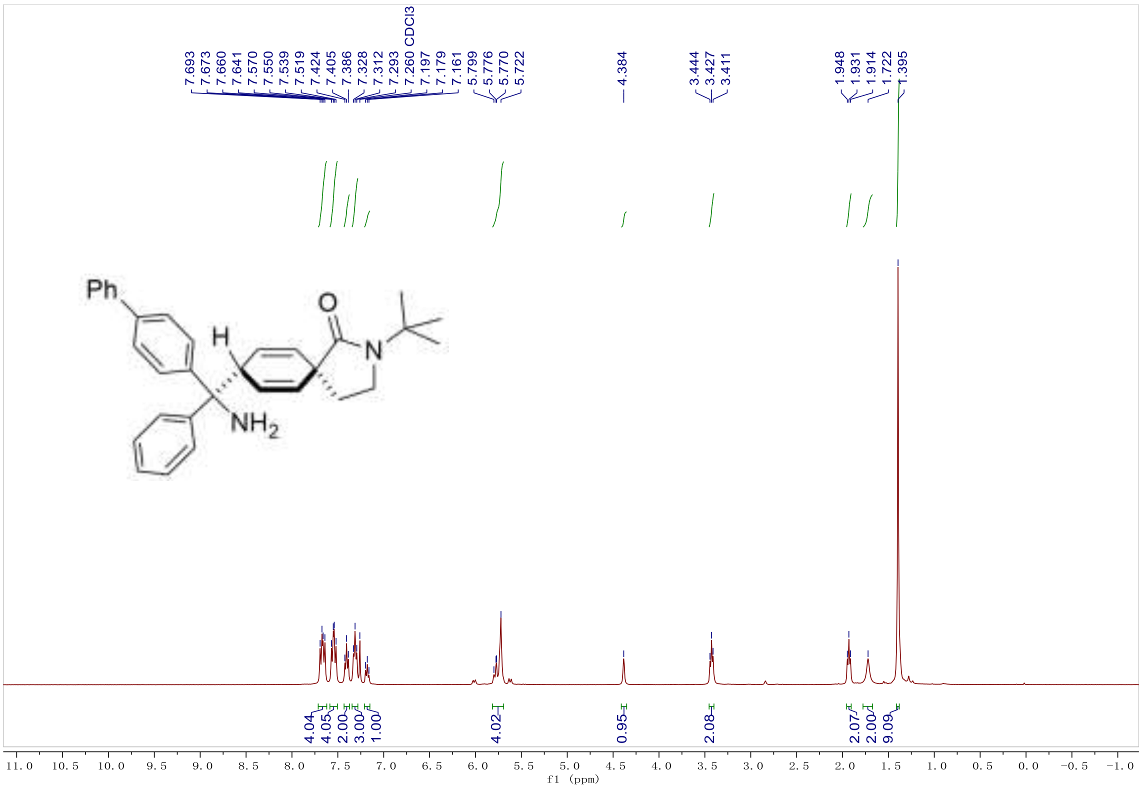

^{1}H-NMR for trans-**3-25**

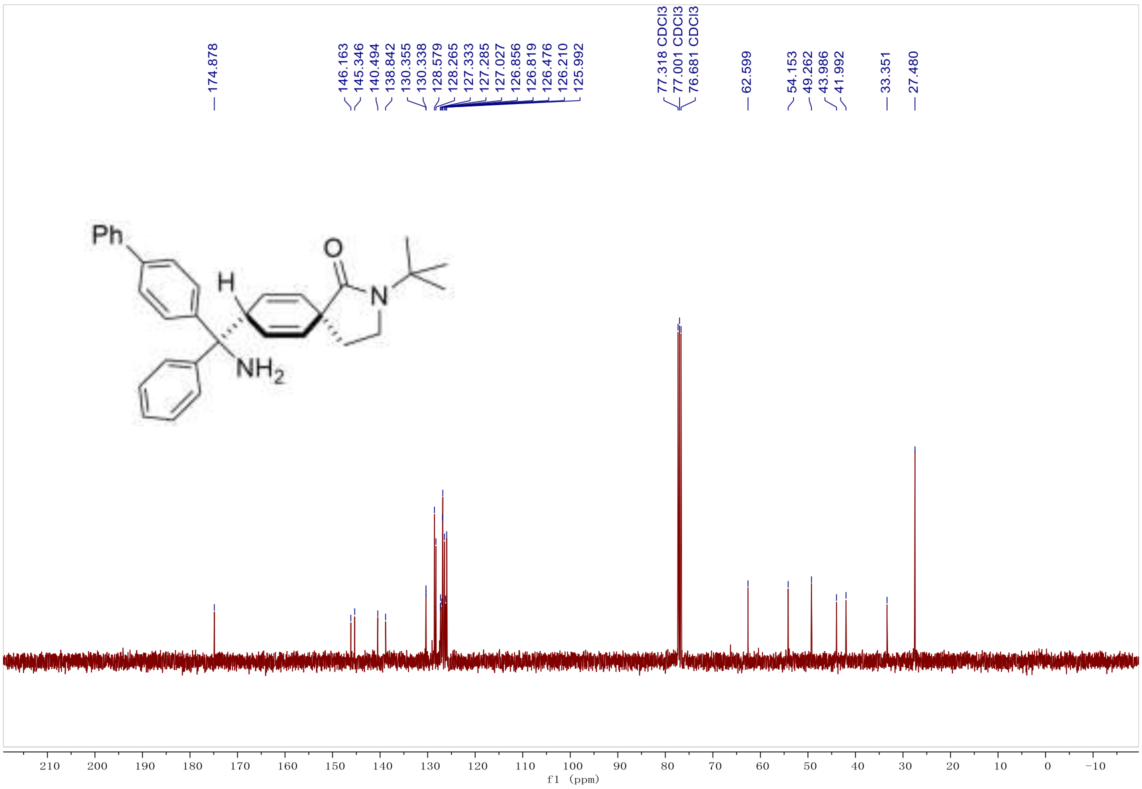

^{13}C−NMR for trans-**3-25**

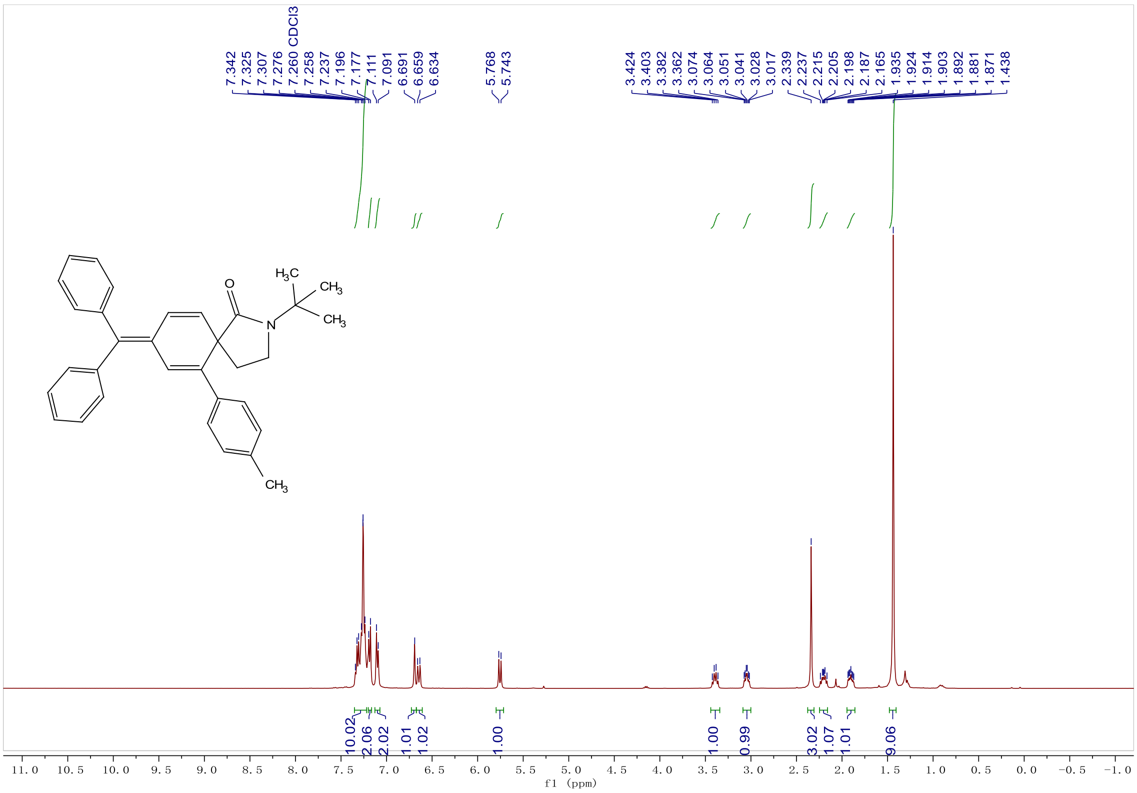

^{1}H-NMR for **7-1**

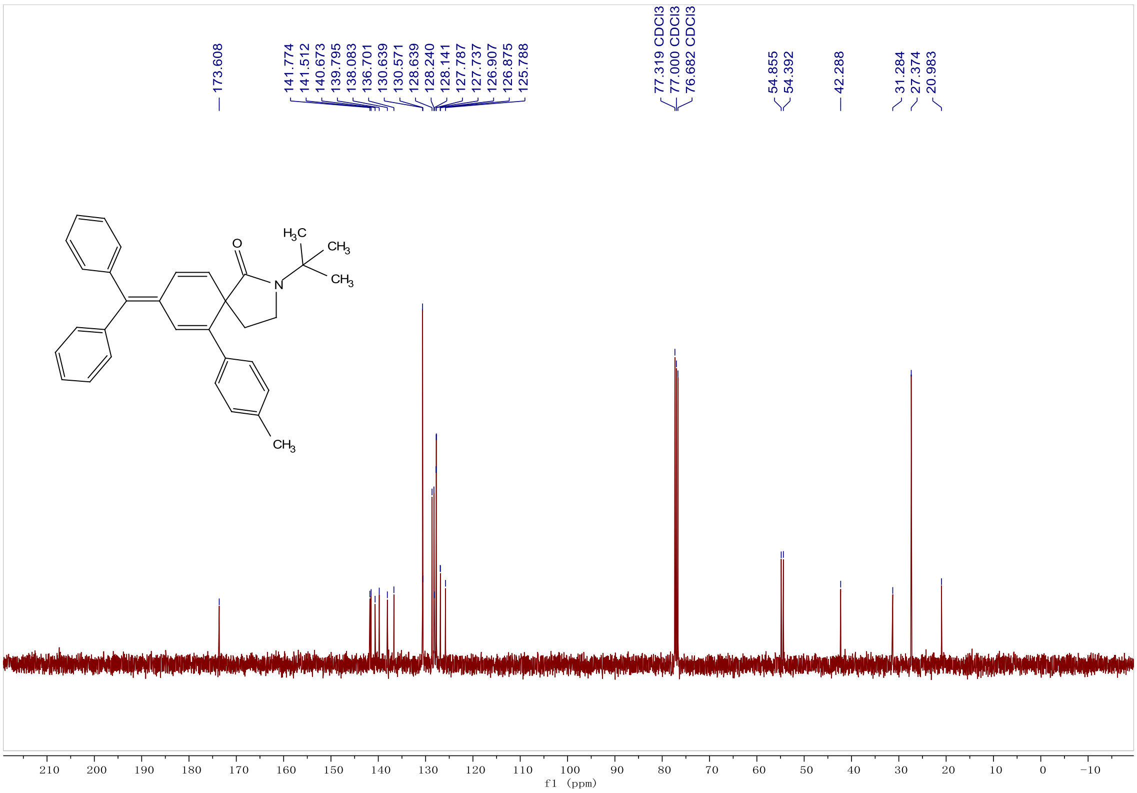

^{13}C−NMR for **7-1**

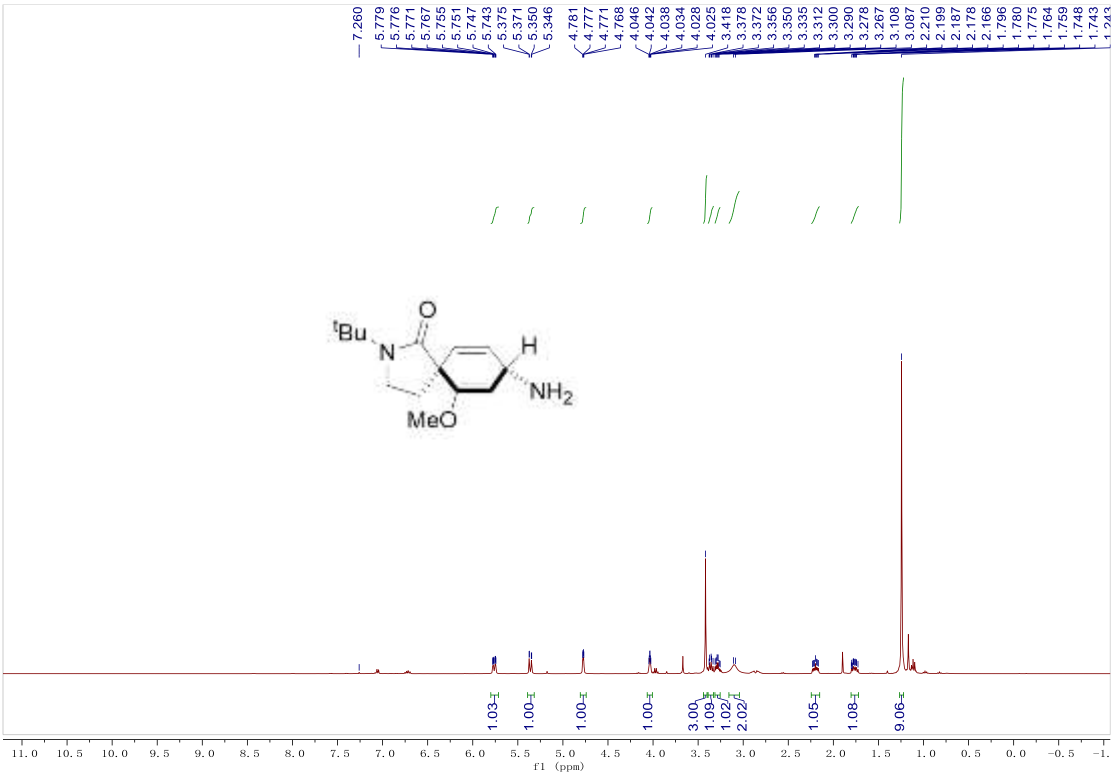

^{1}H-NMR for trans-**2-3-NH$_2$**

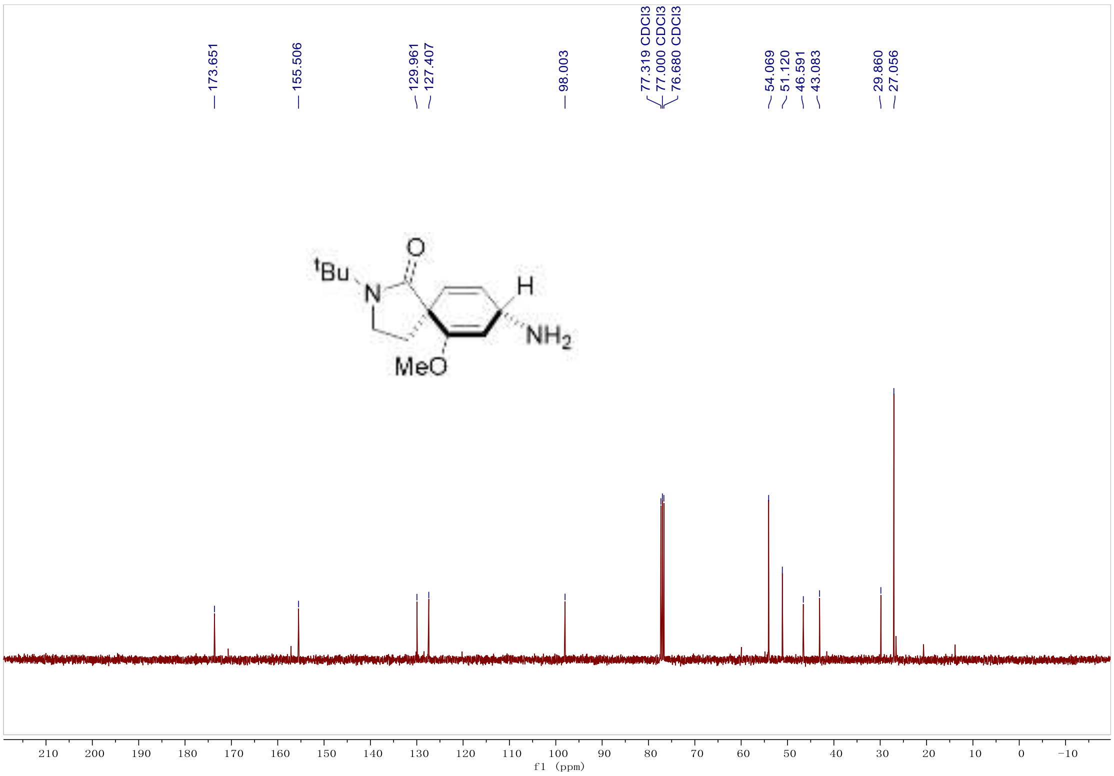

^{13}C-NMR for trans-**2-3-NH$_2$**

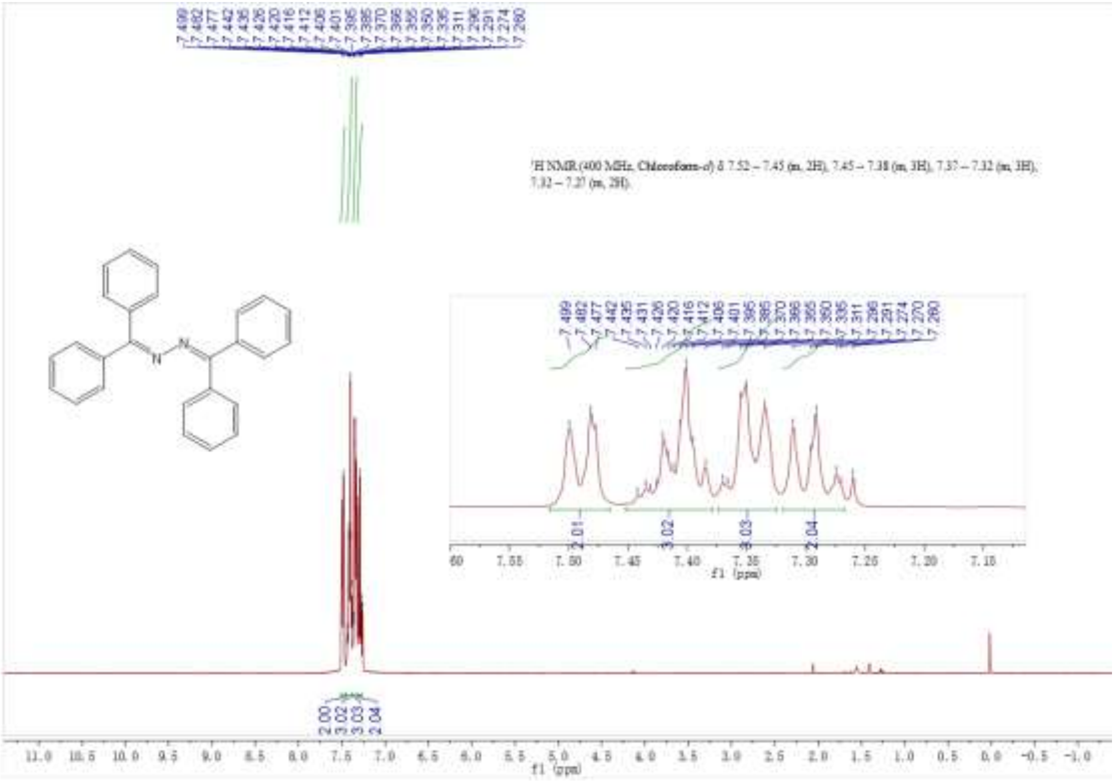

^{1}H-NMR for **Dimer-2**

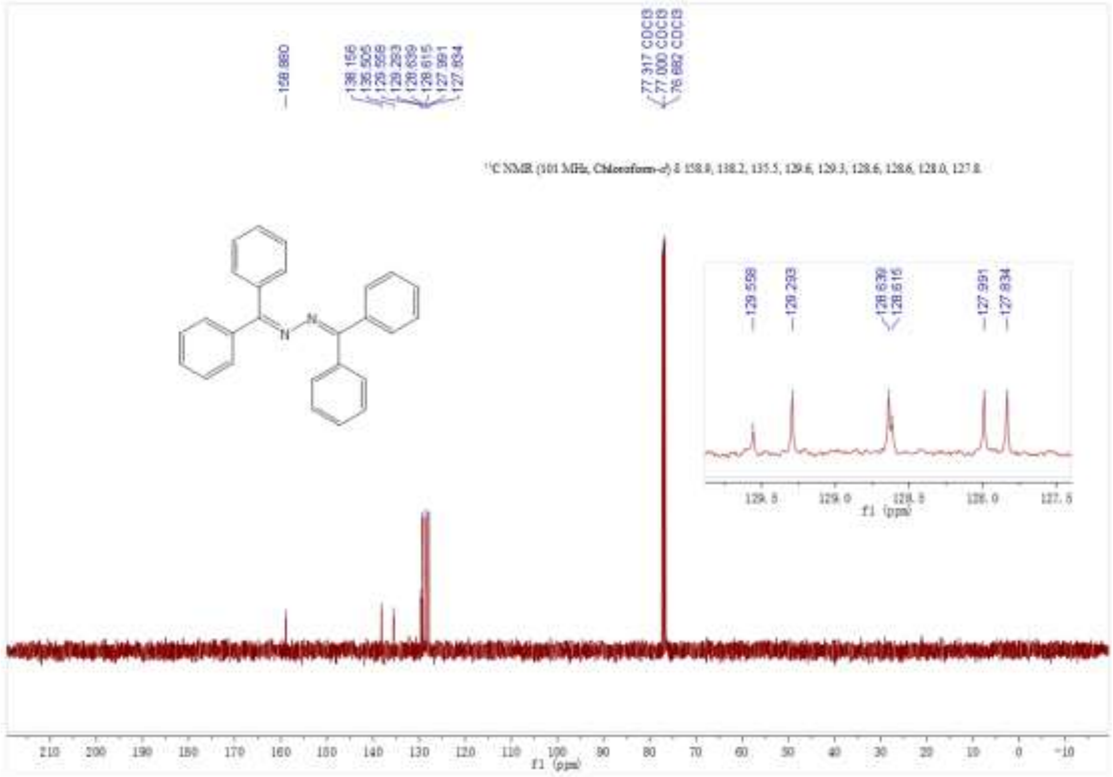

^{13}C−NMR for **Dimer-2**

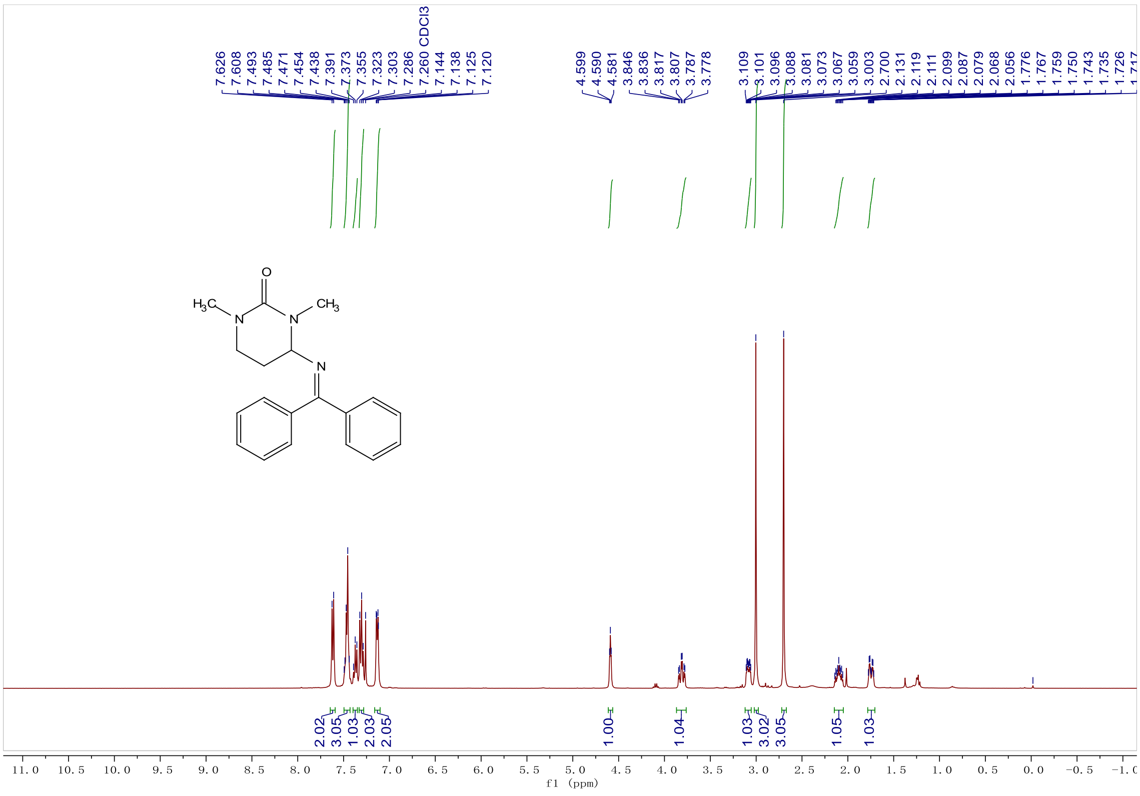

^{1}H-NMR for **DMPU-1**

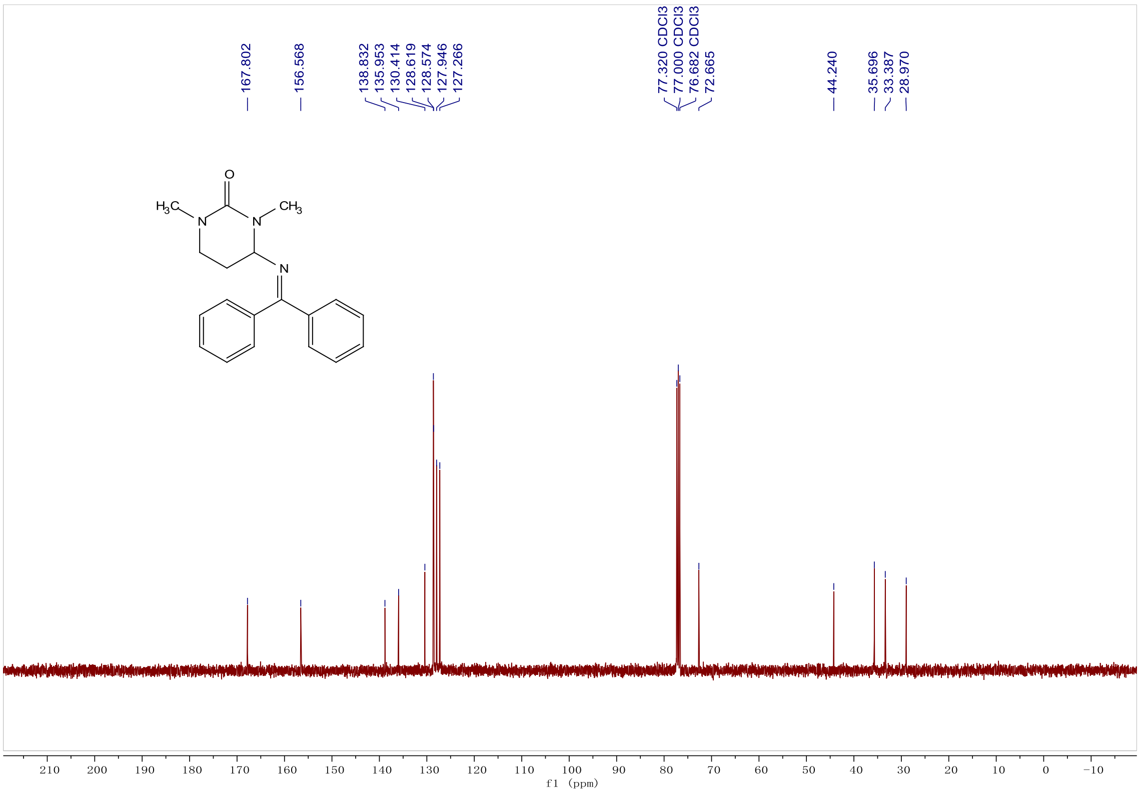

^{13}C−NMR for **DMPU-1**
